# Synthesis of a Systematic 64‐Membered Heparan Sulfate Tetrasaccharide Library

**DOI:** 10.1002/anie.202211985

**Published:** 2022-12-01

**Authors:** Kedar N. Baryal, Sherif Ramadan, Guowei Su, Changxin Huo, Yuetao Zhao, Jian Liu, Linda C. Hsieh‐Wilson, Xuefei Huang

**Affiliations:** ^1^ Department of Chemistry Michigan State University 578 S. Shaw Lane East Lansing MI 48824 USA; ^2^ Chemistry Department Faculty of Science Benha University Benha Qaliobiya 13518 Egypt; ^3^ Glycan Therapeutics 617 Hutton Street Raleigh NC 27606 USA; ^4^ School of Life Sciences Central South University Changsha Hunan 410013 China; ^5^ Division of Chemical Biology and Medicinal Chemistry Eshelman School of Pharmacy University of North Carolina Chapel Hill NC 27599 USA; ^6^ Division of Chemistry and Chemical Engineering California Institute of Technology Pasadena CA 91125 USA; ^7^ Institute for Quantitative Health Science and Engineering Michigan State University East Lansing MI 48824 USA; ^8^ Department of Biomedical Engineering Michigan State University East Lansing MI 48824 USA

**Keywords:** biological activity, carbohydrates, heparan sulfate, library synthesis, oligosaccharides

## Abstract

Heparan sulfate (HS) has multifaceted biological activities. To date, no libraries of HS oligosaccharides bearing systematically varied sulfation structures are available owing to the challenges in synthesizing a large number of HS oligosaccharides. To overcome the obstacles and expedite the synthesis, a divergent approach was designed, where 64 HS tetrasaccharides covering all possible structures of 2‐*O*‐, 6‐*O*‐ and *N*‐sulfation with the glucosamine‐glucuronic acid‐glucosamine‐iduronic acid backbone were successfully produced from a *single* strategically protected tetrasaccharide intermediate. This extensive library helped identify the structural requirements for HS sequences to have strong fibroblast growth factor‐2 binding but a weak affinity for platelet factor‐4. Such a strategy to separate out these two interactions could lead to new HS‐based potential therapeutics without the dangerous adverse effect of heparin‐induced thrombocytopenia.

## Graphical abstract

A library of 64 heparan sulfate tetrasaccharides containing all possible arrangements of 2‐*O*‐, 6‐*O*‐ and *N*‐sulfation with the glucosamine‐glucuronic acid‐glucosamine‐iduronic acid backbone was successfully produced from a *single* strategically protected tetrasaccharide intermediate. This library enabled the determination of heparan sulfate structures for strong binding with fibroblast growth factor‐2 but weak binding to platelet factor‐4.

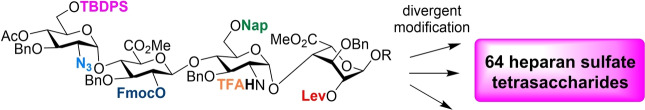

## Introduction

Heparan sulfate (HS) is a member of the glycosaminoglycan (GAG) family present on mammalian cell surfaces and extracellular matrices as proteoglycan, which regulates a wide range of biological functions, including blood coagulation, cell differentiation, inflammatory response, neurodegeneration, tumor metastasis and viral infections.[^1^, ^2^, ^3^] HS is a negatively charged linear polysaccharide with the repeating disaccharide units of glucosamine (GlcN)‐α‐1→4 linked to a uronic acid, which can be either D‐glucuronic acid (GlcA) or L‐iduronic acid (IdoA). Biosynthesis of HS starts at the Golgi apparatus with the assembly of the homopolymer backbone of *N*‐acetyl glucosamine (GlcNAc)‐α‐1→4‐GlcA‐ followed by enzyme mediated modification of GlcNAc to *N*‐sulfated glucosamine (GlcNS).[^2^, ^4^] This is followed by the epimerization of the GlcA unit at C5 to IdoA and finally *O*‐sulfation of the uronic acid and glucosamine. These enzyme‐mediated modifications are often incomplete, which result in highly heterogeneous HS biopolymers in nature. On the other hand, the biological functions of HS can be sequence dependent, as the length of the glycan, uronic acid structures and the number and location of sulfates can significantly influence their activities.[^5^, ^6^, ^7^, ^8^] For example, major alternations in the composition of HS were observed in the brains of Alzheimer's disease patients, which have been implicated in Alzheimer's disease development.^[9]^ Likewise, the interactions of viruses such as the SARS‐CoV‐2 virus,^[10]^ cytomegalovirus,^[11]^ and HIV‐1[^12^, ^13^] with host cells can be facilitated by selective HS structures.

To better understand the nature of HS‐protein interactions, and to establish the detailed structure activity relationship (SAR), well‐defined HS sequences are needed. As it is challenging to obtain pure HS in sufficient amounts from natural sources due to its structural heterogeneity, synthesis is critical. Over the past two decades, significant advances have been achieved in the preparation of HS oligosaccharides.[^14^, ^15^, ^16^, ^17^] Industrial scale synthesis of heparin like pentasaccharide Fondaparinux, an FDA approved drug for the treatment of deep vein thrombosis, is an elegant example of the power of modern synthetic chemistry.^[8]^ Many synthetic studies to date are focused on specific target structures, with HS sequences having the length approaching those of the HS polysaccharides successfully prepared.[^14^, ^18^, ^19^] However, to expedite the SAR studies, libraries of HS structures are needed. Building HS libraries is challenging with the diverse sulfation possibilities (*N*, 3‐*O* and 6‐*O* of GlcN and 2‐*O* position of GlcA/IdoA) as well as the variations of the backbone structures.[^20^, ^21^] Elegant chemical and chemoenzymatic synthesis of libraries of HS disaccharides[^22^, ^23^, ^24^] and tetrasaccharides^[25]^ have been reported. However, to date, no tetrasaccharide libraries are available yet with systematically varied sulfation patterns.

In this work, we report a divergent approach to produce a systematic library of 64 HS tetrasaccharides with all possibilities of *N*‐, 6‐*O*‐, and 2‐*O*‐ sulfations on the GlcN‐GlcA‐GlcN‐IdoA backbone. To expedite the synthesis, a key linchpin tetrasaccharide precursor was prepared, which bore strategically placed orthogonally removable protective groups at the positions to be potentially sulfated. From this single key intermediate, 64 HS tetrasaccharides were synthesized with systematically varied sulfation patterns, opening the door towards comprehensive HS oligosaccharide libraries.

## Results and Discussion

**Scheme 1:**
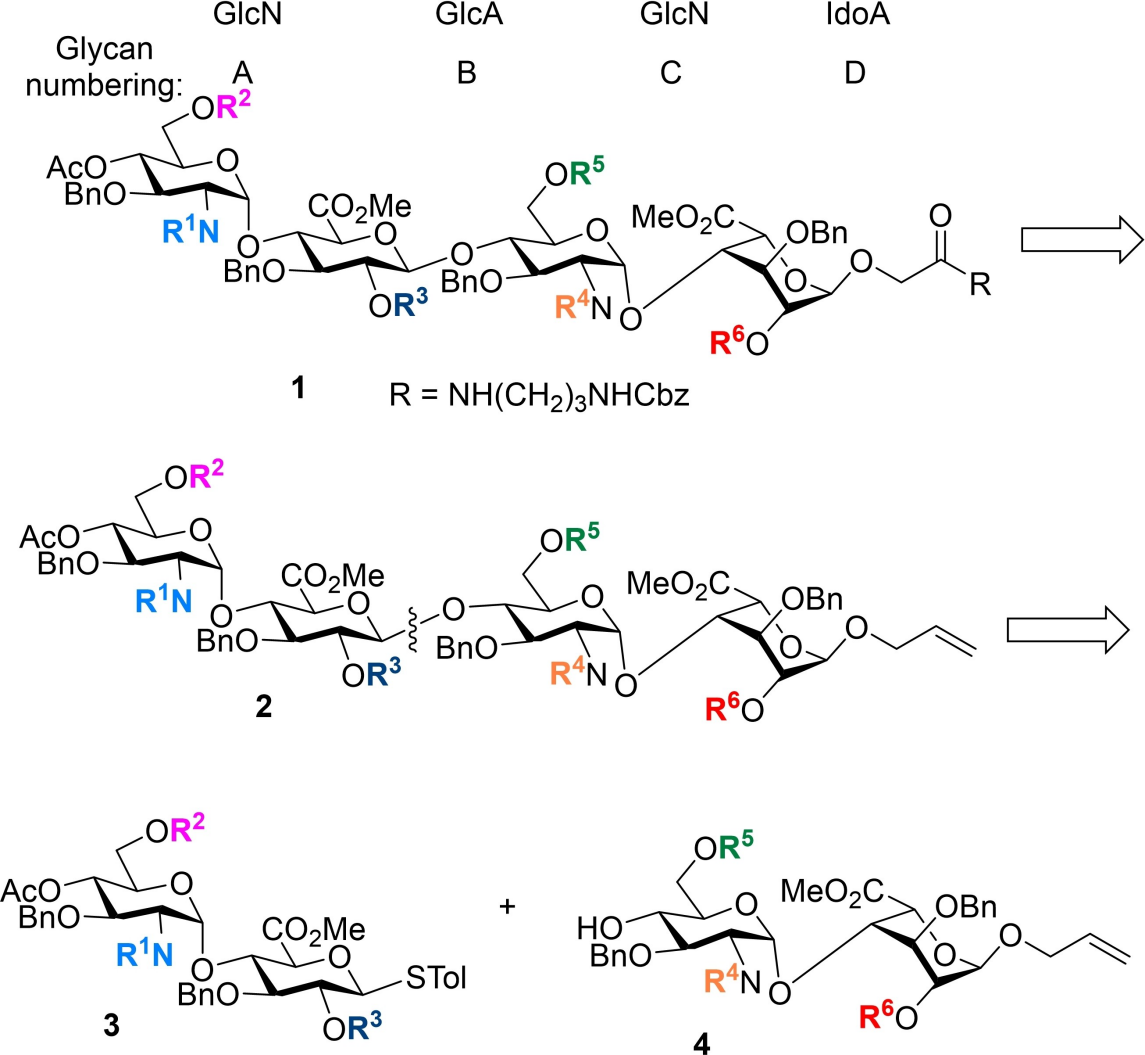
Structure of the key HS tetrasaccharide **1** and its retrosynthetic analysis.

To prepare a HS library, an important consideration is how to reduce the number of total synthetic steps needed. Rather than starting from a uniquely protected tetrasaccharide precursor for each of the 64 final HS tetrasaccharides, we envision the library preparation can be significantly expedited by divergent modifications of a *single* strategically protected key tetrasaccharide such as **1** (Scheme 1). The tetrasaccharide **1** has the GlcN‐GlcA‐GlcN‐IdoA backbone, selectively removable protective groups on hydroxyl group positions for future *O*‐sulfation, and differentially masked nitrogen moieties on the two GlcN units (rings A and C). The GlcN‐GlcA‐GlcN‐IdoA sequence was selected as it contains both GlcA and IdoA as a representative HS tetrasaccharide backbone. Protective group chemistry was to be established to orthogonally deprotect *O*‐protective groups to enable subsequent *O*‐sulfation and divergent generation of HS tetrasaccharides with systematically varied sulfation patterns.

Retrosynthetically, tetrasaccharide **1** could be obtained from allyl tetrasaccharide **2**, which in turn could be prepared by the reaction of disaccharide **3** with the allyl disaccharide acceptor **4** (Scheme 1). Through extensive screening of protective group chemistry, we selected fluorenylmethoxycarbonyl (Fmoc), levulinoyl (Lev), 2‐naphthylmethyl (Nap), and *tert*‐butyldiphenyl silyl (TBDPS) groups as potential protective groups for the *O*‐sulfation sites for our library design. The nitrogen moieties of the GlcNs in **3** and **4** would be differentiated as N_3_ and trifluoroacetamide (NHTFA) respectively.

**Figure 1:**
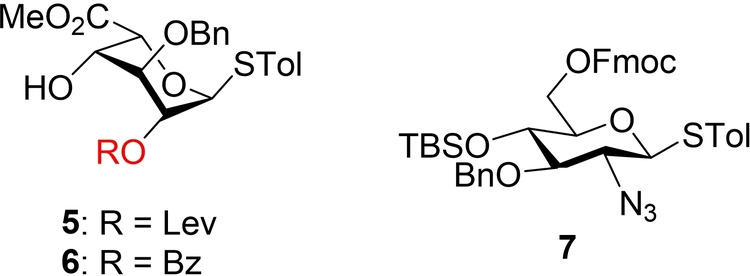
Structures of building blocks **5**–**7**.

**Scheme 2:**
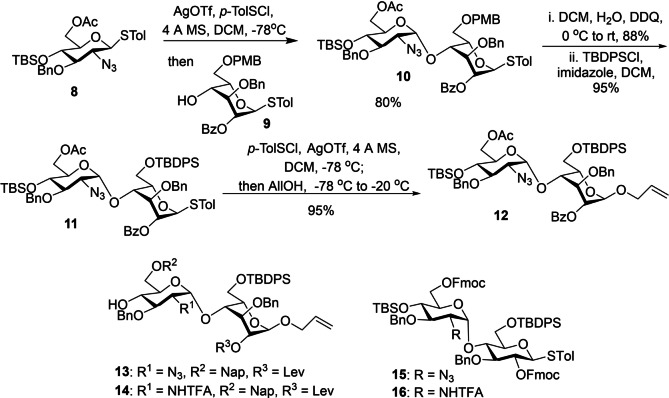
Synthesis of allyl disaccharide **12**.

In order to form the allyl disaccharide **4**, we first explored the IdoA thioglycoside **5** (Figure 1) as the acceptor. However, its glycosylation by the glucosamine donor **7** as well as the subsequent reaction with allyl alcohol gave low yields and stereoselectivities. Changing the 2‐*O*‐Lev to 2‐*O*‐Bz (IdoA **6**) did not help improve the stereoselectivities. To overcome this challenge, we investigate the alternative of using the hexose building block in place of the uronic ester. Preactivation of donor **8** ^[26]^ promoted by *p*‐toluene sulfenyl chloride (*p*‐TolSCl) and silver triflate (AgOTf)^[27]^ followed by its glycosylation of idoside (Ido) **9** ^[26]^ formed disaccharide **10** in 80 % yield (Scheme 2). To avoid the remote participation of the 6‐*O*‐PMB moiety in **10** upon its activation as a donor for further glycosylation,^[21]^ the 6‐*O*‐PMB of **10** was replaced with 6‐*O*‐TBDPS leading to disaccharide **11**. Preactivation of **11** with *p*‐TolSCl/AgOTf followed by the addition of allyl alcohol produced the allyl glycoside **12** in 95 % yield with the *α*‐anomer as the sole product isolated (^1^J(C_1_,H_1_)=170.5 and 170.5 Hz indicating two *α*‐linkages)^[28]^ (Scheme 2). **12** was transformed into disaccharides **13** and **14** bearing Nap, Lev and NHTFA moieties (Scheme S1).

**Scheme 3:**
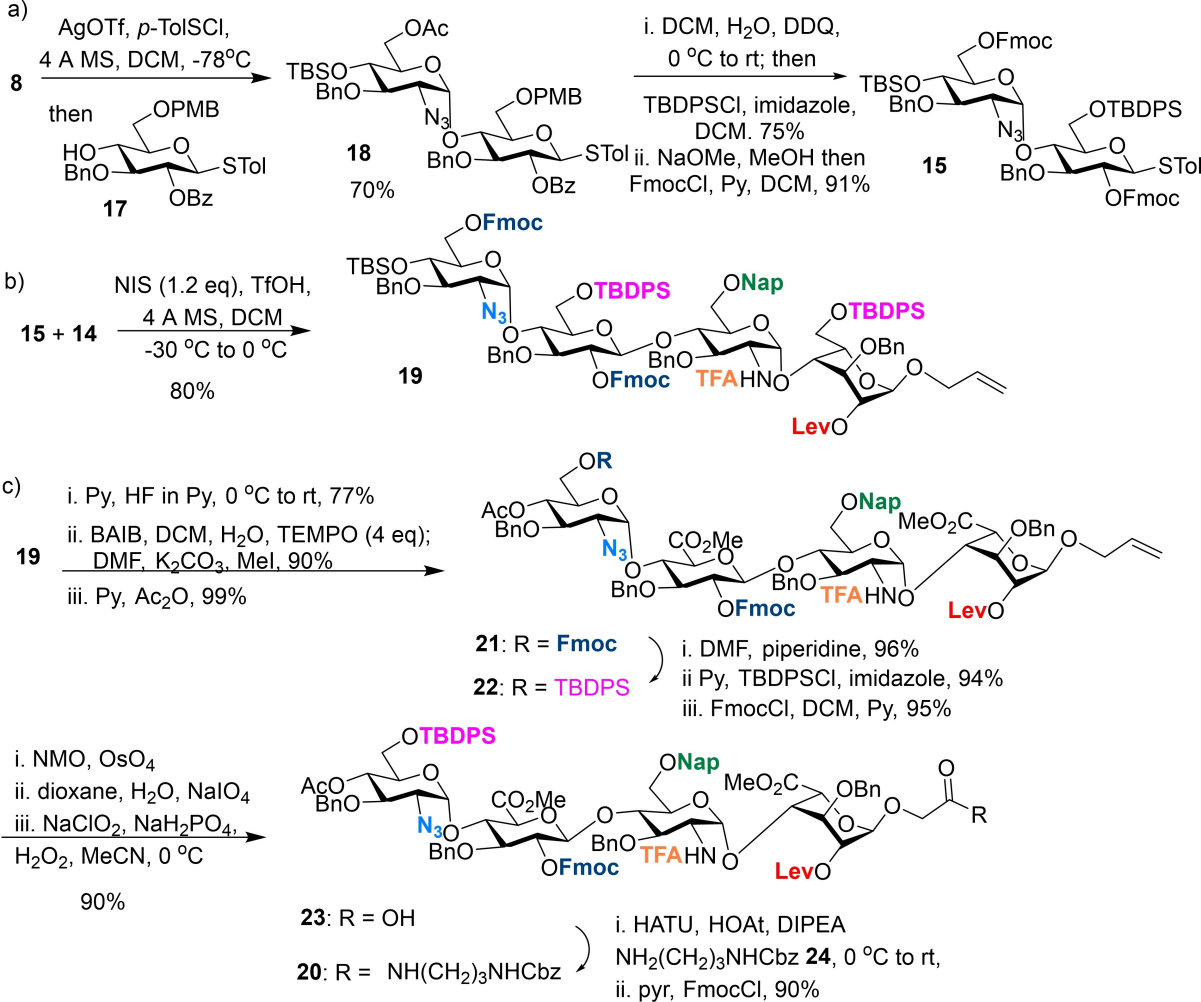
Synthesis of a) disaccharide **15**, b) tetrasaccharide **19**, and c) tetrasaccharide **20**.

With the GlcN‐Ido disaccharides **12**–**14** in hand, we moved onto the preparation of GlcN‐Glc disaccharide donors **15** and **16** (Scheme 3a). Glycosylation of glucosamine donor **8** and glucoside acceptor **17** ^[29]^ produced disaccharide **18** in 70 % yield. Protective group adjustment of **18** led to disaccharide donor **15** bearing the azide functionalized GlcN (Scheme 3a). Next, the formation of HS tetrasaccharide backbone was explored. The glycosylation of donor **16** with acceptor **13** gave no desired tetrasaccharide. Interestingly, swapping the N_3_ and the NHTFA groups in the two disaccharides, i.e., pairing donor **15** with acceptor **14**, generated tetrasaccharide **19** in 80 % yield with a newly formed *β*‐glycosyl linkage confirmed by NMR analysis (^1^J(C_1_,H_1_)=164.5 Hz) (Scheme 3b). These results suggest that it was detrimental to have the TFA amide in the donor.^[30]^ The high yield for the formation of **19** indicated the Fmoc moiety could serve well as a participating neighboring group to assist the 1,2‐*trans* glycoside formation for HS synthesis.

We next converted the tetrasaccharide **19** to the key intermediate **20** with orthogonal protective groups placed at strategic positions (Scheme 3c). The glucose and idose moieties of **19** were transformed into the uronic esters as in **21**, the 6‐*O*‐Fmoc group of which was subsequently exchanged to a TBDPS group (compound **22**). To facilitate future biological investigations, the allyl group at the reducing end of **22** was converted to a carboxylic acid. Ozonolysis was utilized first to oxidize the allyl moiety. However, the Nap group in **22** was cleaved during ozonolysis reaction.^[31]^ To solve this problem, we resorted to the alternative route of osmium tetroxide mediated *N*‐methylmorpholine *N*‐oxide (NMO) dihydroxylation to obtain a vicinal diol,[^32^, ^33^] which with subsequent sodium periodate and Pinnick oxidation^[34]^ gave carboxylic acid **23** in 90 % yield over three steps. This was followed by HATU‐HOAt‐mediated amidation of **23** with benzyl‐3‐aminopropyl carbamate **24**. We found the 2‐*O*‐Fmoc group was labile under the amidation condition presumably due to the excess (5 eq) amount of benzyl‐3‐aminopropyl carbamate **24** used to drive the amidation. The Fmoc was reintroduced easily after the amide formation affording the key tetrasaccharide **20**.

**Scheme 4:**
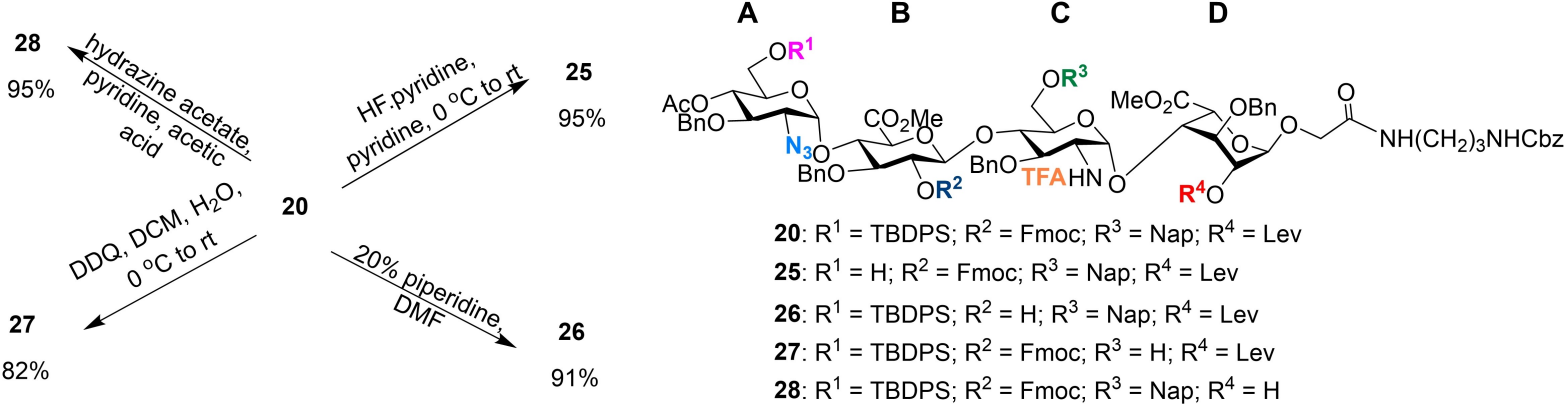
Orthogonal deprotection of tetrasaccharide **20**.

The common *O*‐sulfation locations of HS include the 6‐*O* position of the GlcN and 2‐*O* position of the uronic acid.[^14^, ^15^, ^16^, ^17^] We explored the orthogonal deprotection^[35]^ of the protective groups installed as these positions in **20** (Scheme 4). Treatment of **20** with HF.pyridine deprotected TBDPS selectively in 95 % yield leaving all other protective groups intact (compound **25**). The Fmoc group of **20** could be removed cleanly with 20 % piperidine in 91 % yield (tetrasaccharide **26**). The Nap moiety of **20** was deprotected in 82 % yield by 2,3‐dichloro‐5,6‐dicyano‐1,4‐benzoquinone (DDQ) oxidation (product **27**), and the Lev group could be cleaved selectively with hydrazine acetate in 95 % yield (tetrasaccharide **28**). Thus, all 6‐*O* of the GlcN and 2‐*O* of the uronic acid positions of **20** could be readily differentiated through orthogonal deprotections.

**Figure 2:**
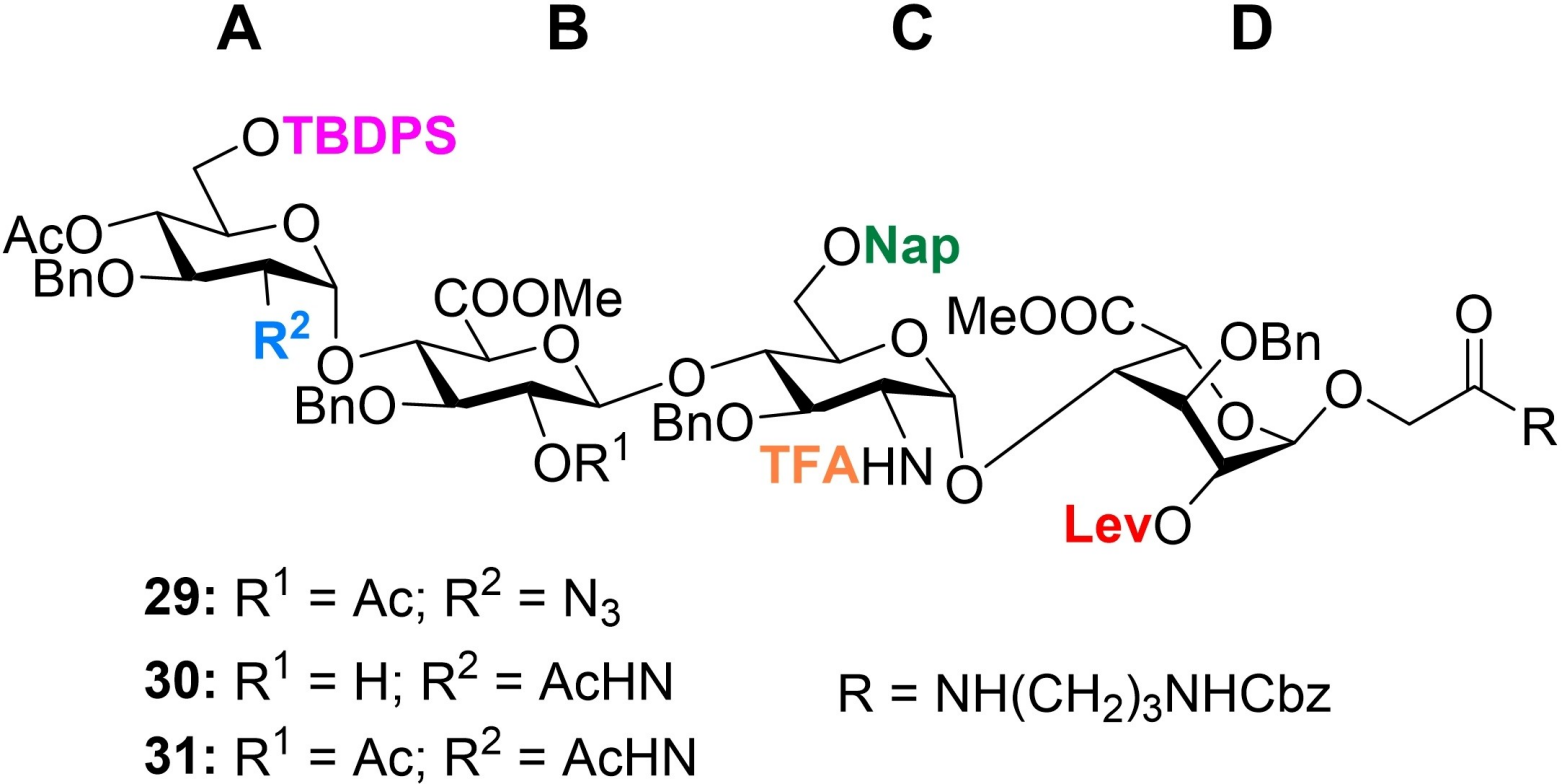
Structures of compounds **29**–**31**.

In tetrasaccharide **20**, six positions (*N*, 6‐*O* of ring A; 2‐*O* of ring B; *N*, 6‐*O* of ring C; 2‐*O* of ring D) can be potentially sulfated to create 2 6=64 HS tetrasaccharides. To accomplish the divergent sulfation, four disaccharide intermediates **26, 29**–**31** (Figure 2) were derived from **20** (Schemes S4 and S5). **26** serves as the precursor to 16 HS tetrasaccharides bearing *N*‐sulfation in ring A and 2‐*O*‐sulfation in ring B. Compound **29** can be converted into 16 HS tetrasaccharides bearing *N*‐sulfation in ring A and 2‐OH in ring B. Compound **30** is the precursor to 16 HS tetrasaccharides bearing NHAc (rather than *N*‐sulfation) in ring A and 2‐*O*‐sulfation in ring B, while **31** can lead to 16 HS tetrasaccharides bearing NHAc in ring A and 2‐OH in ring B.

**Scheme 5:**
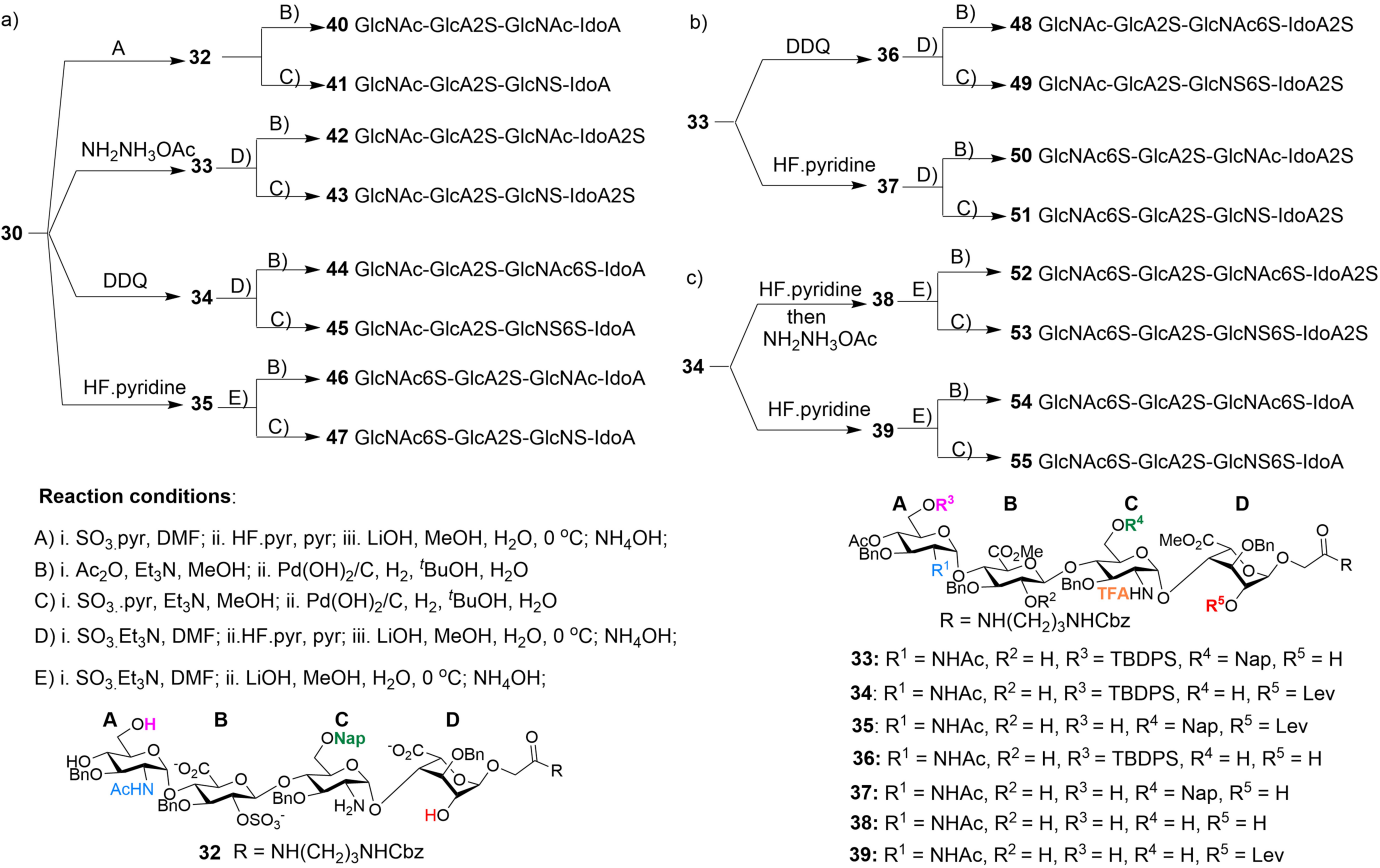
Divergent modification of tetrasaccharide **30** led to the production of 16 HS tetrasaccharides **40**–**55** bearing GlcNAc in ring A and GlcA2S in ring B.

As a representative example, the divergent transformation of **30** to 16 HS tetrasaccharides is shown in Scheme 5. Firstly, acetamide **30** was treated with SO_3_.pyr for 2‐*O*‐sulfation of ring B (Scheme 5a). The resulting monosulfate was then subjected for TBDPS deprotection with HF.pyridine followed by hydrolysis of all ester groups and the TFA amide to generate the free amine **32**. Careful control of the hydrolysis condition was needed to avoid the cleavage of the amide in the linker at the reducing end. The amine in **32** was acetylated with acetic anhydride in methanol in presence of triethylamine to access acetamide followed by palladium hydroxide mediated hydrogenation to produce compound **40** (GlcNAc‐GlcA2S‐GlcNAc‐IdoA) in 38 % overall yield for the 5 steps from **30**. Alternatively, rather than acetylation, the free amine bearing tetrasaccharide **32** was heated with SO_3_.pyr in methanol followed by hydrogenolysis, which resulted in the bis(*O*‐ and *N*‐)sulfated compound **41** (GlcNAc‐GlcA2S‐GlcNS‐IdoA) in 33 % yield from **30**.

**Figure 3:**
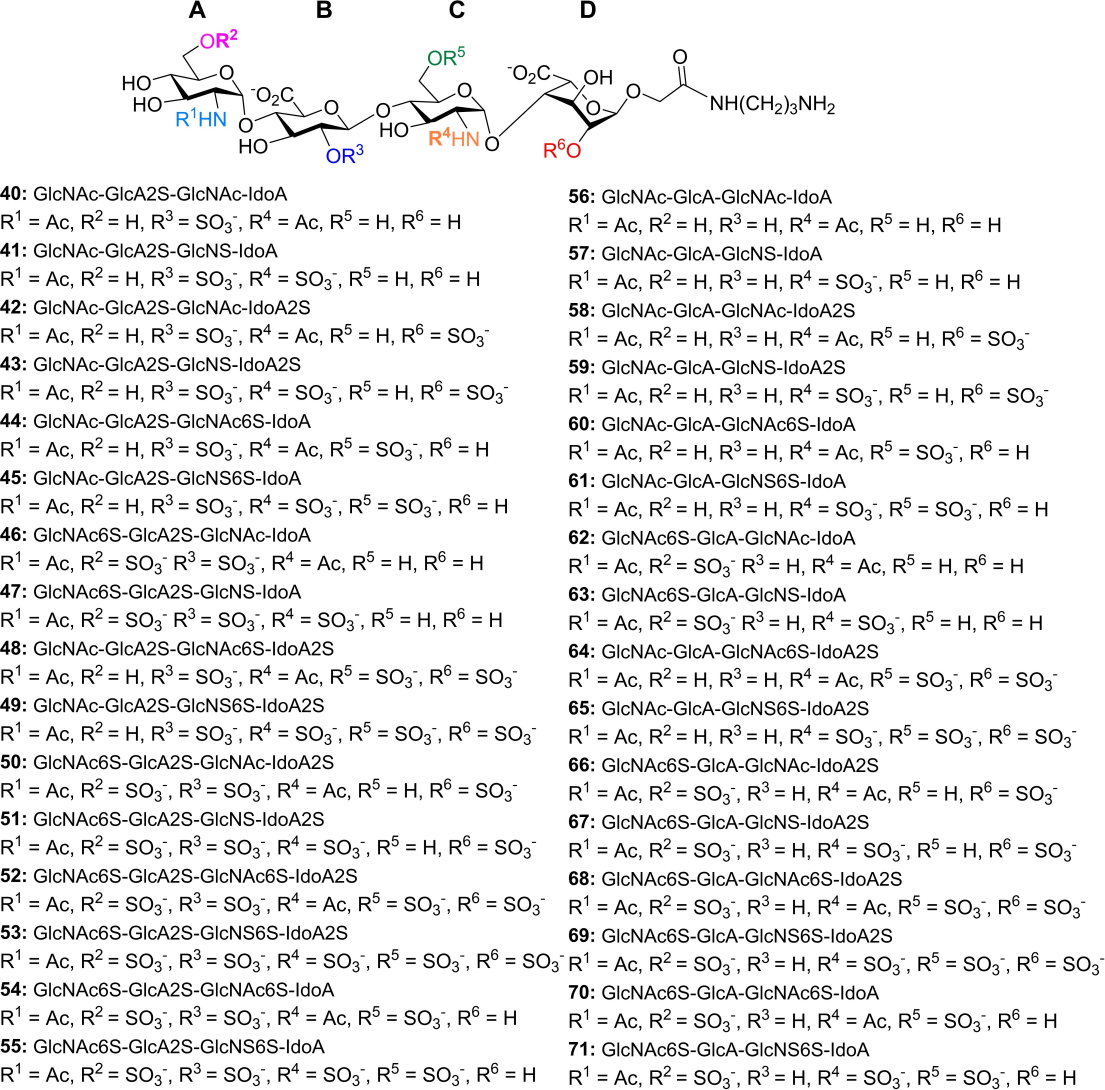
Structures of HS tetrasaccharides **40**–**71**.

To introduce 2‐*O*‐sulfation into IdoA (ring D) of the HS tetrasaccharides, hydrazine acetate mediated Lev group removal was performed on acetamide **30** to generate free 2‐OH on IdoA (compound **33**) (Scheme 5a). Parallel to removing the Lev group, the Nap group of **30** was deprotected selectively with DDQ to produce tetrasaccharide **34** with a free 6‐OH on ring C. The TBDPS group of **30** was removed selectively to generate tetrasaccharide **35** with a free 6‐OH on ring A. Modifications of **33**, **34** and **35** following the same reaction sequences as in the production of **40** and **41** (Figure 3) produced the HS tetrasaccharides **42** (GlcNAc‐GlcA2S‐GlcNAc‐IdoA2S) and **43** (GlcNAc‐GlcA2S‐GlcNS‐IdoA2S) bearing 2‐*O*‐sulfation on the ring D (IdoA), the HS tetrasaccharides **44** (GlcNAc‐GlcA2S‐GlcNAc6S‐IdoA) and **45** (GlcNAc‐GlcA2S‐GlcNS6S‐IdoA) bearing 6‐*O*‐sulfation on ring C, and the HS tetrasaccharides **46** (GlcNAc6S‐GlcA2S‐GlcNS‐IdoA) and **47** (GlcNAc6S‐GlcA2S‐GlcNS‐IdoA) bearing 6‐*O*‐sulfation on ring A respectively. Combination of the Lev, Nap and TBDPS removal from acetamide **33** (Scheme 5b) and **34** (Scheme 5c) followed by sulfation and subsequent deprotection produced HS tetrasaccharides **48**–**55**.

**Figure 4:**
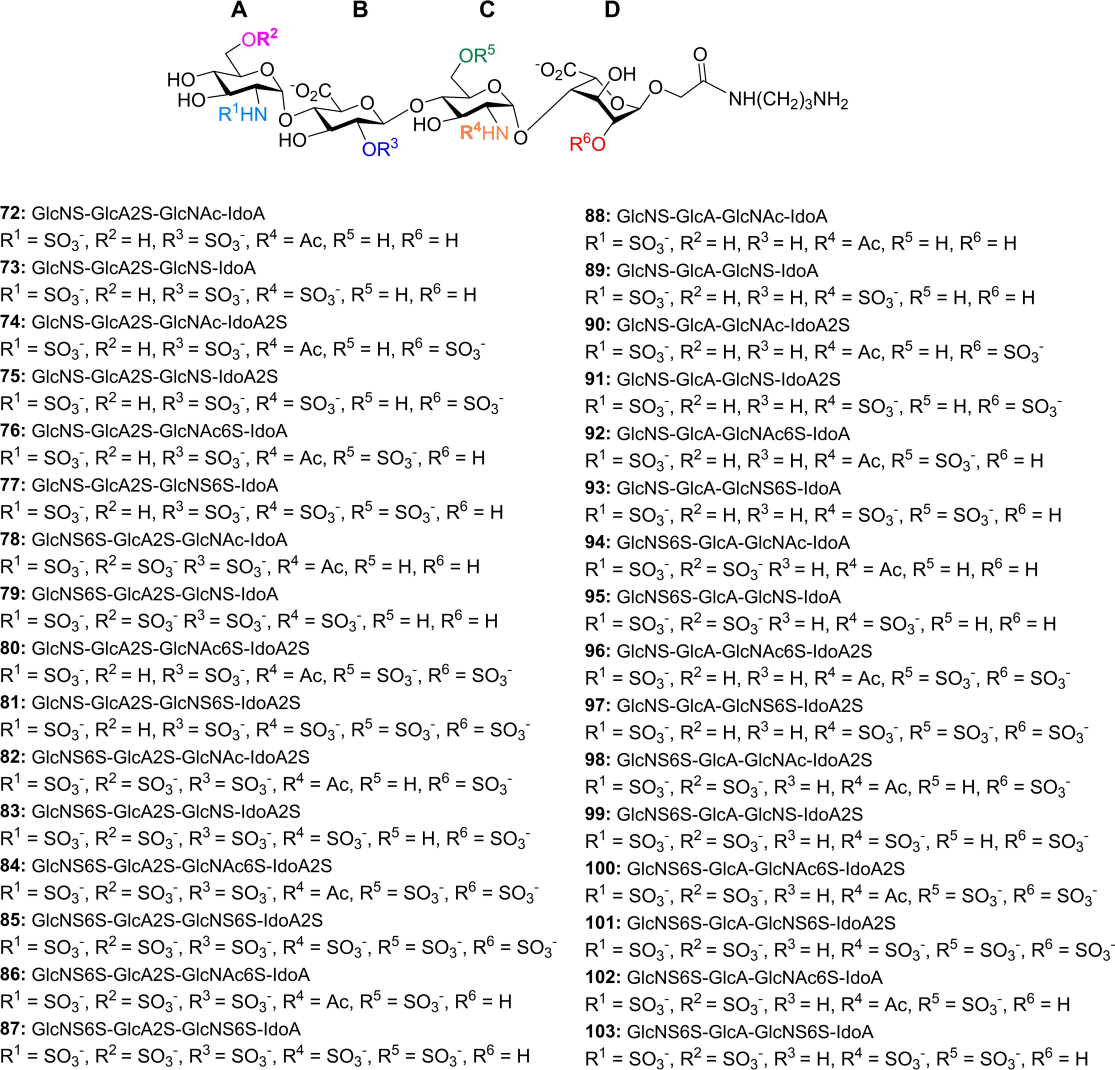
Structures of HS tetrasaccharides **72**–**103**.

With the series of 16 compounds synthesized starting from **30**, HS tetrasaccharides **56** to **103** (Figures 3 and 4) were prepared in a similar fashion from compounds **26**, **29**, and **31** respectively completing the full library of 64 HS tetrasaccharides bearing systematically varied *N*‐sulfation, 2‐*O*‐ and 6‐*O*‐sulfations on the GlcN‐GlcA‐GlcN‐IdoA backbone (Schemes S6–S9). All the final compounds were produced on a 2–5 mg scale. The NMR and MS data were consistent with the structures.

With the 64‐membered HS tetrasaccharide library in hand, their binding with fibroblast growth factor‐2 (FGF‐2) was investigated to establish the impacts of the fine structures of the HS tetrasaccharides. FGF‐2 plays important roles in regulating cell growth and proliferation.[^36^, ^37^] In nature, cell‐surface HS binds with FGF‐2 and helps regulate its function.^[38]^ While FGF‐2 interactions with HS is a common model used in HS studies,[^21^, ^25^, ^39^, ^40^, ^41^] this would be the first time that the FGF‐2 binding is probed with an extensive library of tetrasaccharides with systematically varied HS structures. The 64 HS tetrasaccharides were immobilized onto *N*‐hydroxysuccinimide (NHS) ester functionalized glass slides through the reducing end amines to produce a HS tetrasaccharide microarray, which is an attractive approach for high‐throughput analysis of HS binding.[^25^, ^39^, ^41^] Fluorescently labeled FGF‐2 was then incubated with the glycan microarray. Upon washing off unbound FGF‐2, the relative amounts of FGF‐2 bound by the HS tetrasaccharides were quantified from the fluorescence intensities on the microarray.

**Figure 5:**
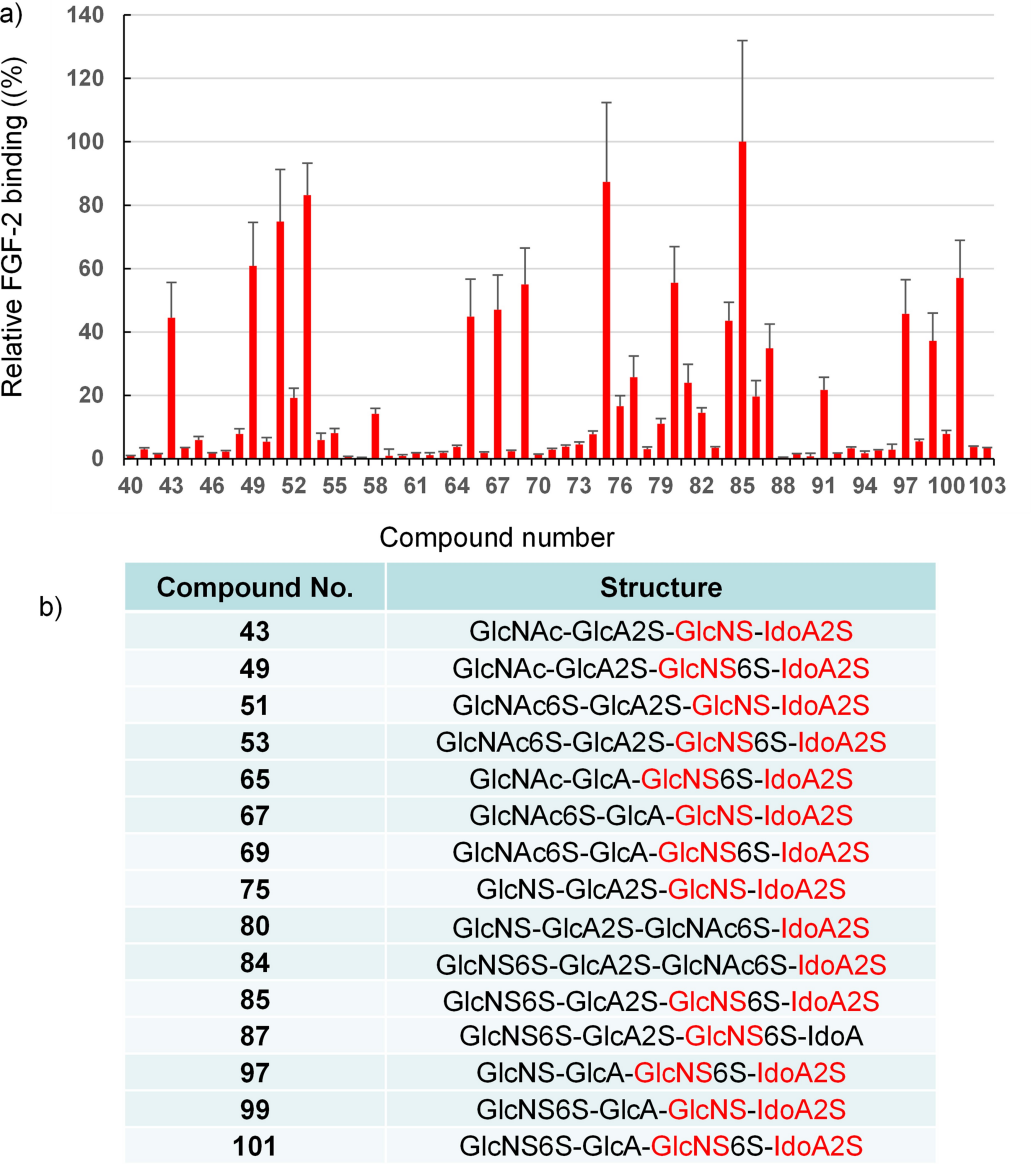
a) Relative FGF‐2 affinity calculated from the fluorescence intensities observed on the glycan microarray upon binding by FGF‐2 to the 64‐membered HS tetrasaccharides with the standard deviation of each microarray component plotted. All samples were printed in replicates of sixteen. The average intensity from the strongest binder (compound **85**) was set as 100 %. b) The sequences of HS tetrasaccharides that exhibited binding with FGF‐2 with the average fluorescence intensities over 30 % of that of the strongest binder.

The availability of the library with systematically varied sulfations enabled us to analyze in depth the impacts of the HS structures. The majority of the strong binders (compounds **43**, **49**, **51**, **54**, **65**, **67**, **69**, **75**, **85**, **97**, **99**, and **101**) bear the GlcNS‐IdoA2S disaccharide moieties at the reducing end (Figure 5b). The presence of 6S on unit C GlcN did not have significant impacts on binding as evident from the comparison of **43** versus **49** and **51** versus **54**. While compounds **80**, **84**, and **87** lacked the GlcNS‐IdoA2S disaccharide at the reducing ends, their strong binding with FGF‐2 was presumably due to the GlcNS‐GlcA2S or GlcNS6S‐GlcA2S moieties at their non‐reducing ends. The importance of 2‐*O*‐sulfation and *N*‐sulfation for HS binding with FGF‐2 is consistent with literature reports.[^41^, ^42^, ^43^] The preference of GlcNS‐IdoA2S can be rationalized by the structure of FGF‐2 with HS oligosaccharides.^[44]^ It is known that HS tetrasaccharide can bind with FGF‐2, with the main polar interactions (ion pair and hydrogen bonding) coming from the internal disaccharide with their 2‐*O*‐ and *N*‐sulfates interacting with FGF‐2 residues Asn‐28, Arg‐121, Lys‐126, and Gln‐135. The 6‐*O*‐sulfate groups point away from the binding site without much contact with FGF‐2.^[44]^

We next compared FGF‐2 binding by compounds with the same number of sulfates. As shown in Figure 5a, while almost all the lightly sulfated tetrasaccharides (total number of sulfates <3) bound FGF‐2 weakly, the total number of sulfate moieties does not closely correlate with FGF‐2 binding affinity. For example, tetrasaccharides **49**, **51**, **55**, **69**, **75**, **77**, **80**, **86**, **97**, **99**, **100**, and **103** all contain four sulfates. Yet, their FGF‐2 binding varied drastically, with **55**, **100**, and **103** having low binding (<10 % of the fluorescence intensity of the strongest binder compound **85**), **77**, **86**, **97**, and **99** with medium affinity (10–55 %), and **49**, **51**, **69**, **75**, and **80** with strong binding (all >55 % with **75** being one of the strongest binders at 87 %). The precise location of sulfate is important. **55** and **69** have identical structures except for the location of the 2‐*O*‐sulfate on the uronic acid (**55** has the 2‐*O*‐sulfate on the GlcA, while **69** has the 2‐*O*‐sulfate on the IdoA). Yet, **69** has significantly stronger binding to FGF‐2 than **55** (55.0 % vs. 8.1 %). Similar phenomena of precise structures rather than the number of sulfates dictating activities were also observed with compounds **53**, **81**, **83**, **84**, **87**, and **101** in the library. Despite that **53**, **81**, **83**, **84**, **87**, and **101** all contain five sulfates in each molecule, they differed significantly in FGF‐2 binding, with **83** being a weak binder (3.4 %), **81**, **84**, and **87** having medium affinities with relative FGF‐2 binding of 24.0 %, 43.6 %, and 34.8 % respectively, and **53** and **101** having strong binding (83.2 % and 57.0 %). These results highlighted that the binding of HS with FGF‐2 is highly sensitive to HS structures, which is determined by specific molecular interactions rather than the cumulative and nonspecific electrostatic effects. At the same time, this also suggests the availability of a comprehensive library can significantly aid in the deciphering of the fine characteristics of the structure and activity relationship of HS.

**Figure 6:**
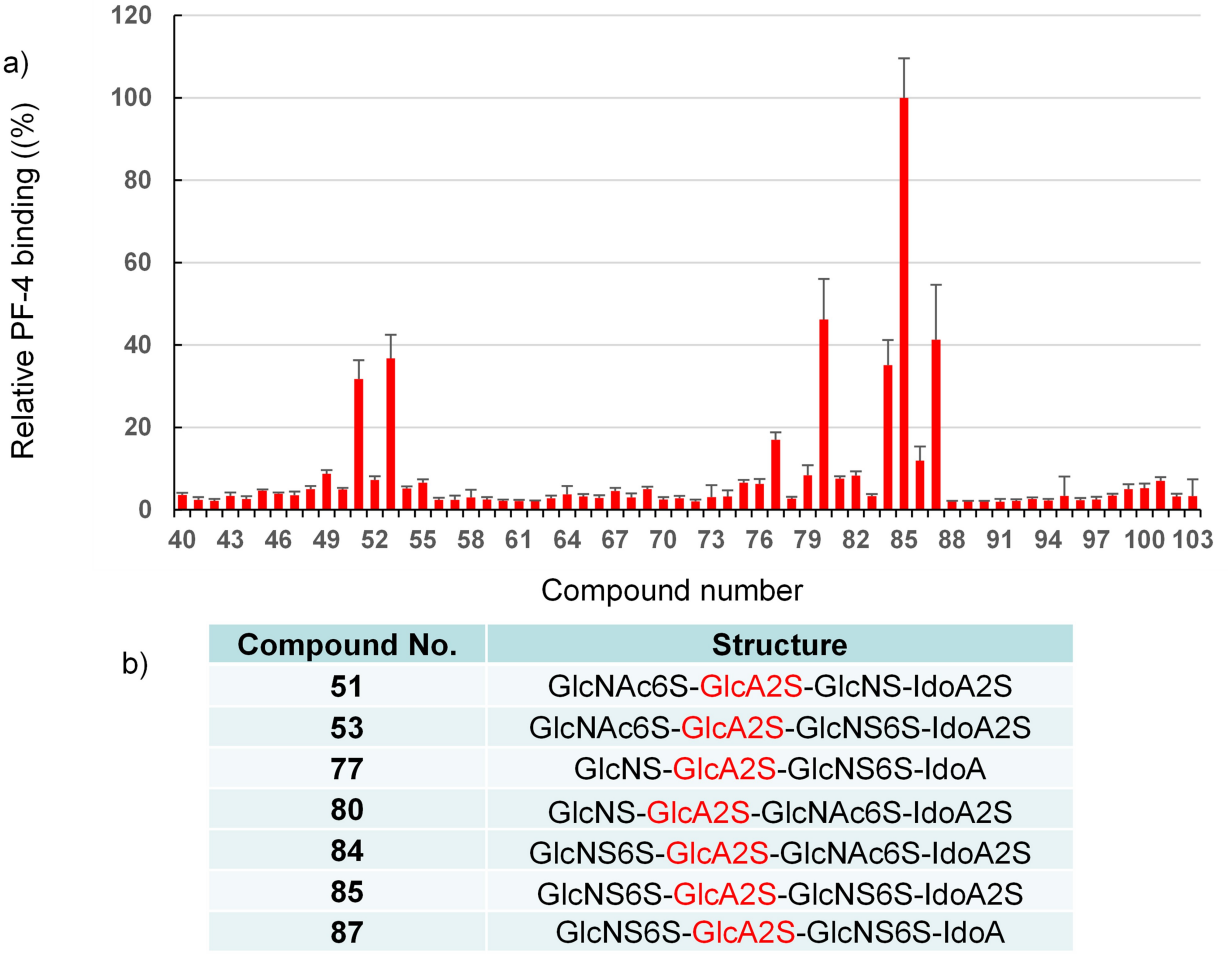
a) Relative PF‐4 affinity calculated from the fluorescence intensities observed on the glycan microarray upon binding by PF‐4 to the 64 membered HS tetrasaccharides with the standard deviation of each microarray component plotted. All samples were printed in replicates of sixteen. The average intensity from the strongest binder (compound **85**) was set as 100 %. b) The sequences of HS tetrasaccharides that bound with PF‐4 with the average fluorescence intensities near or above 20 % of that of the highest binder.

Platelet factor‐4 (PF‐4) is a 70 amino acid polypeptide that is released from the activated platelets and can bind with HS.^[45]^ The HS/PF‐4 complex is the antigen in heparin‐induced thrombocytopenia (HIT), a life‐threatening autoimmune reaction due to the administration of the anticoagulant heparin.[^46^, ^47^] For HS based therapeutics, the induction of HIT initiated through PF‐4 binding with HS needs to be avoided. Screening of PF‐4 on the HS tetrasaccharide microarray showed that PF‐4 is more selective towards HS structures as compared to FGF‐2 (Figure 6a). PF‐4 prefers highly sulfated HS sequences with all strong binders (compounds **51**, **53**, **77**, **80**, **84**, **85**, and **87**) (Figure 6b) containing GlcA2S and at least four sulfates per molecule. It has been proposed that the binding of HS with PF‐4 occurs generally through nonspecific electrostatic interactions^[48]^ with a series of basic amino acid residues in HS binding site of PF‐4 including Lys‐146, 161, 162, 165, 166 and Arg 122, 220 and 249 identified.^[49]^ However, we found that PF‐4 binding can be critically dependent on the location of the sulfate. For example, compounds **75** and **80** have the same number (4) of sulfates but differ in the location of one of the four sulfates (Figure 6b). **75** has the *N*‐sulfation at the C unit of GlcN, while **80** bears a sulfate on 6‐OH of the same GlcN. Yet, while **80** is one of the strongest binders of PF‐4 identified in the library (46.2 % of the strongest binder **85**), compound **75** interacts little (6.6 %) with PF‐4 despite that it contains GlcA2S and four sulfates. Thus, for potential FGF‐2 targeting HS based binder design, tetrasaccharide **75** can be a strong lead as it binds well with FGF‐2 (Figure 5b) but with a relatively low affinity with PF‐4. Similarly, compounds **43**, **49**, **65**, **67**, **69**, **97**, **99** and **101** can also serve as potential candidates for future development of FGF‐2 binders with high FGF‐2 binding and reduced possibilities of HIT.

## Conclusion

To expedite the biological understanding of HS, structurally diverse HS structures are critically needed. A systematic library of 64 HS tetrasaccharides with the GlcN‐GlcA‐GlcN‐IdoA backbone sequence has been successfully synthesized for the first time. The distinctive feature of the synthesis is that rather than starting from a uniquely protected tetrasaccharide for each of the final HS target, a single key strategically protected tetrasaccharide was designed, from which 64 HS tetrasaccharides were produced in good overall yields. Such a divergent strategy reduced the number of synthetic steps needed to access the 64‐compound library from the protected tetrasaccharide by 50 %, thus enhancing the overall synthetic efficiencies.

The availability of the 64 membered HS tetrasaccharide library enabled us to probe systematically the impacts of 2‐*O*‐, 6‐*O*‐ and *N*‐sulfation on HS binding with FGF‐2 and PF‐4. The new information generated gives us a more comprehensive understanding of the fine structural characteristics of HS‐receptor interactions. As the formation of HS/PF‐4 complex can lead to life‐threatening adverse reactions in human bodies, the binding of potential HS based therapeutics with PF‐4 should be avoided. From the microarray screening studies, several compounds with strong binding to FGF‐2 but relatively low PF‐4 affinity have been identified, which can serve as potential leads for further development.

In parallel to this work, a library of 64 HS tetrasaccharides with the GlcN‐IdoA‐GlcN‐IdoA backbone has been synthesized assisted by fluorous chemistry.^[50]^ It helped enhance the understanding of the roles of sulfation for modulating the activities of growth factors and chemokines with the GlcN‐IdoA‐GlcN‐IdoA sequence. Combined with the current work on HS tetrasaccharides with the GlcN‐GlcA‐GlcN‐IdoA backbone, these results highlight the utility of the systematic HS tetrasaccharide libraries, which can provide exciting new opportunities to target important biomedical events.

## Conflict of interest

JL is a founder for Glycan Therapeutics. GS is an employee of Glycan Therapeutics. The authors declare no other conflicts of interests.

1

## Supporting information

As a service to our authors and readers, this journal provides supporting information supplied by the authors. Such materials are peer reviewed and may be re‐organized for online delivery, but are not copy‐edited or typeset. Technical support issues arising from supporting information (other than missing files) should be addressed to the authors.

Supporting Information

## Data Availability

The data that support the findings of this study are available in the supplementary material of this article.
