# Supplementary material for: Synthesis of a Systematic 64‐Membered Heparan Sulfate Tetrasaccharide Library
Source: Angew Chem Int Ed Engl. 2022 Dec 1;62(1):e202211985. doi: 10.1002/anie.202211985 (PMC9933061; doi:10.1002/anie.202211985)
Supplement: Supplementary file 1 — Supporting Information [file ANIE-62-0-s001.pdf]

Angewandte Chemie
Eine Zeitschrift der Gesellschaft Deutscher Chemiker

Supporting Information

Synthesis of a Systematic 64-Membered Heparan Sulfate Tetrasaccharide Library

*K. N. Baryal, S. Ramadan, G. Su, C. Huo, Y. Zhao, J. Liu, L. C. Hsieh-Wilson, X. Huang**

Supporting Information

Synthesis of a Systematic 64 Membered Heparan Sulfate Tetrasaccharide Library

Kedar N. Baryal[a#], Sherif Ramadan[a,b#], Guowei Su[c], Changxin Huo[a], Yuetao Zhao[d,a], Jian Liu[e], Linda C. Hsieh-Wilson[f] and Xuefei Huang[a,g,h]*

[a]Department of Chemistry, Michigan State University, 578 S. Shaw Lane, East Lansing, Michigan 48824, USA

[b]Chemistry Department, Faculty of Science, Benha University, Benha, Qaliobiya 13518, Egypt

[c]Glycan Therapeutics, 617 Hutton Street, Raleigh, North Carolina 27606, USA

[d]School of Life Sciences, Central South University, Changsha, Hunan 410013, China

[e]Division of Chemical Biology and Medicinal Chemistry, Eshelman School of Pharmacy, University of North Carolina, Chapel Hill, North Carolina 27599, USA

[f]Division of Chemistry and Chemical Engineering, California Institute of Technology, Pasadena, California 91125, USA

[g]Institute for Quantitative Health Science and Engineering, [h]Department of Biomedical Engineering, Michigan State University, East Lansing, Michigan 48824, USA

[#]Equal contribution

Email: huangxu2@msu.edu

Table of Contents:

Experimental procedures and characterization data………………… **S3**

Scheme S1. Syntheses of disaccharides **13** and **14**. **S9**

Scheme S2. Synthesis of disaccharide **15**. **S15**

Scheme S3. Synthesis of tetrasaccharide **23**. **S17**

Scheme S4. Syntheses of tetrasaccharides **25 - 28**. **S23**

Scheme S5. Syntheses of tetrasaccharides **29 - 31**. **S26**

Scheme S6. Syntheses of tetrasaccharides **40 - 55**. **S29**

Scheme S7. Syntheses of tetrasaccharides **56 - 71**. **S37**

Scheme S8. Syntheses of tetrasaccharides **72 - 87**. **S43**

Scheme S9. Syntheses of tetrasaccharides **88 - 103**. **S48**

Procedures for microarray studies **S62**

References **S63**

NMR spectra **S64**

Experimental procedures and characterization data:

General experimental procedures:

All reactions were carried out under nitrogen with anhydrous solvents in flame-dried glassware, unless otherwise noted. Glycosylation reactions were performed in the presence of molecular sieves, which were flame–dried right before the reaction under high vacuum. Glycosylation solvents were dried using a solvent purification system and used directly without further drying. Chemicals used were reagent grade as supplied except where noted. Analytical thin-layer chromatography was performed using silica gel 60 F254 glass plates. Compounds were visualized by UV light (254 nm) and by staining with a yellow solution containing $Ce(NH_4)_2(NO_3)_6$ (0.5 g) and $(NH_4)_6Mo_7O_{24}$ $4H_2O$ (24.0 g) in 6% H_2SO_4 (500 mL). Flash column chromatography was performed on silica gel 60 (230-400 Mesh). Optical rotations were recorded on a Perkin Elmer 341 Polarimeter (λ= 589 nm, 1 dm cell).

General Procedure for Pre-activation Based Glycosylation.

A mixture of donor (60 μmol) and freshly activated 4 Å molecular sieves (200 mg) in CH_2Cl_2 (1 mL) was stirred at room temperature for 30 min and then cooled to−78 °C. AgOTf (31 mg, 120 μmol) dissolved in Et_2O (1 mL) was added directly to the solution making sure the solution did not touch the walls of the flask. After 5 min, orange-colored *p*-TolSCl (9.5 μL, 60 μmol) was added via a microsyringe directly to the flask, as the reaction temperature was lower than the freezing point of *p*-TolSCl and it would freeze on the walls of the flask. The color of p-TolSCl disappeared rapidly, indicating its consumption. After the donor was completely consumed, as verified by TLC analysis (about 5 min at −78 °C), a solution of acceptor (54 μmol) in CH_2Cl_2 (1 mL) along with 1 equiv of TTBP was slowly added dropwise along the walls of the flask. This was done to allow the acceptor solution to cool before mixing with the activated donor. The reaction mixture was stirred at -78 ^{o}C in around 2 h, then triethylamine (TEA) was added to quench the reaction. The mixture was diluted with CH_2Cl_2 and filtered through Celite. After washing the Celite with CH_2Cl_2 until all organic compounds were removed, as verified by TLC of the filtrate, the CH_2Cl_2 fractions were combined and washed twice with a saturated aqueous solution of $NaHCO_3$ (20 mL), and twice with water (10 mL). The organic layer was collected and dried over Na_2SO_4. After removal of the solvent, the product was purified by silica gel flash chromatography.

General Procedure for Non-Pre-activation Based Glycosylation.

A mixture of donor (60 μmol), acceptor (54 μmol) and freshly activated 4 Å molecular sieves (200 mg) in CH_2Cl_2 (1 mL) was stirred at room temperature for 30 min and then cooled to −40 °C covered by aluminum foil to shield from light. NIS (60 μmol) was added directly to the solution. After 5 min, TfOH (6 μmol) was added via a microsyringe directly to the flask. The reaction mixture was stirred in around 2 h. Then TEA was added to quench the reaction. The mixture was diluted with CH_2Cl_2 and filtered through Celite. After washing the Celite with CH_2Cl_2 until all organic compounds were removed, as verified by TLC of the filtrate, the CH_2Cl_2 fractions

were combined and washed twice with a saturated aqueous solution of $NaHCO_3$ (20 mL), and twice with water (10 mL). The organic layer was collected and dried over Na_2SO_4. After removal of the solvent, the product was purified by silica gel flash chromatography.

General Procedure for Naphthylmethyl (Nap) Formation.

To a solution of the oligosaccharide (58 μmol) and TMSCl (16 μL, 116 μmol) in CH_2Cl_2, TEA (29 μL, 232 μmol) was added at 0 ^oC, and the mixture was stirred at room temperature overnight. The solvent was removed, and the residue was dissolved by hexane and filtered. The filtrate was concentrated, and the crude product was used for next step reaction directly. A solution of the mixture from the last step and Et_3SiH (14 μL, 87 μmol) in CH_2Cl_2 (5.0 mL) was stirred at room temperature for 30 min over freshly activated 4 Å molecular sieves (100 mg) and then cooled to −78 °C. A solution of 2-naphthaldehyde (36 mg, 232 μmol) in CH_2Cl_2 (5.0 mL) was added, followed by addition of TMSOTf (2 μL, 12 μmol). The reaction was kept at -78 ^oC for 3 hours, then TEA was added to quench the reaction. The mixture was diluted with CH_2Cl_2 and filtered through Celite. After washing the Celite with CH_2Cl_2 until all organic compounds were removed, as verified by TLC of the filtrate, the CH_2Cl_2 fractions were combined. After removal of the solvent, the product was purified by silica gel flash chromatography.

General Procedure for Staudinger Reduction.

1 M PMe_3 solution in THF (5 equiv per N_3), 0.1 M aqueous solution of NaOH (3 equiv per N_3), and H_2O (2 mL) were added consecutively to a solution of azide-containing compound (for 50 mg of compound, 1 equiv) in THF (7 mL). The mixture was stirred at 50 ^oC for 3 hours and neutralized with 0.1M HCl until pH = 7. The mixture was concentrated to dryness, and the resulting residue was purified by silica gel flash chromatography.

General Procedure for TFA Protection of Amine.

A solution of the free amine bearing oligosaccharide (38 μmol), CF_3CO_2Me (50 μL, 400 μmol) and TEA (100 μL) in methanol was stirred at 50 ^oC overnight. After cooling to room temperature, the solvent was removed, and the residue was purified by silica gel flash chromatography.

General Procedure for *O*-Acylation:

To a solution of oligosaccharide (780 mg) in pyridine (2.3 mL) at 0°C was added acetic anhydride (1.2 mL). The reaction mixture was stirred for 2.5 h and diluted with ethyl acetate (50 mL) washed with 1 M HCl, water, followed by concentration and silica gel flash chromatography purification to afford the *O*-acylated product.

General Procedure for Converting Azide to Acetamide:

To a solution of oligosaccharide (2.06 g) in pyridine (10 mL) at 0°C was added thioacetic acid (10 mL). The reaction mixture was stirred at 40 °C for 24 h. It was concentrated, dissolved in

ethyl acetate (100 mL), washed with 1 M HCl, water, brine, dried over sodium sulfate, and purified by silica gel flash chromatography to afford the desired product.

General Procedure for *N*-Acylation Converting Amine to Acetamide:

To a solution of oligosaccharide (12 mg) in methanol (1 mL) was added triethyl amine (70 µL) and acetic anhydride (40 µL). The reaction mixture was stirred overnight, concentrated, and purified by silica gel flash chromatography to afford the desired product.

General Procedure for Deprotection of Naphthylmethyl (Nap) and *p*-Methoxybenzyl (PMB) Groups:

The Nap- or PMB-containing oligosaccharide (0.1 mmol) was dissolved in dichloromethane (DCM):water (10:1, 2.2 mL) and cooled down to 0 °C. Next, DDQ (0.15 mmol) was added and stirred overnight at rt. Mixture was then diluted with 30 mL water. The aqueous layer was extracted with DCM (3X 30 mL). The organic layers were combined, washed with brine (50 mL), dried over sodium sulfate, concentrated; and purified by silica gel flash column chromatography.

General Procedure for Deprotection of a Silyl Group.

The TBDPS- or TBS-containing oligosaccharide (0.54 mmol) was transferred to a 50 mL plastic centrifuge tube by three portions of 3.33 mL of pyridine. While stirring, the pyridine solution was cooled to 0 °C. Then, HF·pyridine (5 mL) was added dropwise to the stirring solution. The reaction was then allowed to warm to room temperature and kept overnight. After verifying that the reaction was complete by TLC, the reaction was diluted with CH_2Cl_2 and washed with a sat. aqueous solution of $NaHCO_3$. The organic layer was dried over Na_2SO_4, concentrated, and purified by silica gel flash chromatography.

General Procedure for TBDPS Ether Formation.

A solution of the oligosaccharide (50 µmol), TBDPSCl (20 µL, 80 µmol), and imidazole (11 mg, 150 µmol) in pyridine was stirred at room temperature overnight. After the starting material was completely consumed, the solvent was removed by co-evaporation with methanol. The residue was purified by silica gel flash chromatography.

General Procedure for Levulinoyl Ester Formation.

A mixture of the oligosaccharide (1 mmol), 1-ethyl-3-(3-(dimethylamino)propyl)-carbodiimide hydrochloride (EDC.HCl 3.3 equiv per OH), and *N*,*N*-dimethylaminopyridine (DMAP, 0.1 equiv per OH) was dissolved in dichloromethane (DCM, 30 mL). To this solution was added levulinic acid (3 equiv per OH), and the reaction was stirred at room temperature overnight. The mixture was then diluted with DCM, washed with sat. $NaHCO_3$, dried over Na_2SO_4, concentrated, and purified by flash silica gel chromatography.

General Procedure for Fmoc Carbonate Formation.

Fmoc chloride (280 mg, 5 equiv per OH) was added to the solution of the oligosaccharide (0.1 mmol) in pyridine (10 mL) at 0 ^{o}C, and the mixture was stirred overnight. The mixture was concentrated to dryness, and the resulting residue was purified by silica gel flash chromatography.

General Procedure for Fmoc Removal.

Piperidine (100 μL) was added to the solution of oligosaccharide (50 μmol) in DMF (1 mL) in an ice bath, and the mixture was stirred at 0 ^{o}C for 1 hour. After TLC showed that the starting material was consumed completely, the reaction mixture was diluted with ethyl acetate and washed by brine three times. After drying the mixture over Na_2SO_4, the solvent was removed, and the residue was purified by silica gel flash chromatography.

General Procedure for Oxidation of 6-OH.

The compound to be oxidized (45 μmol) was dissolved in a solution of DCM (2 mL), ^{t}-BuOH (2 mL), and H_2O (0.5 mL). To this solution was added TEMPO (26.5 μmol or 0.3 equiv per 6-OH), followed by iodobenzene diacetate (BAIB) (221 μmol or 2.5 equiv per 6-OH). The reaction was then stirred at room temperature overnight. After the reaction was complete as judged by TLC (1% acetic acid in ethyl acetate), the reaction was quenched by the addition of saturated aqueous $Na_2S_2O_3$ solution (2 mL) and allowed to stir at room temperature for 15 min. The mixture was then diluted with 10 mL of DCM and 3 mL of H_2O and separated. The aqueous layer was acidified with 1 M HCl solution and extracted three times with DCM. The organic layers were combined, dried over Na_2SO_4, and concentrated. The crude product was then be protected as a benzyl or methyl ester.

General Procedure for Methyl Ester Formation after Oxidation.

The crude product from oxidation was dissolved in DMF (2 mL for 15 μmol). To this solution was added K_2CO_3 (5 equiv per COOH), followed by CH_3I (2.5 equiv per COOH), and the reaction was allowed to stir overnight at room temperature. After verifying that the reaction was complete by TLC, the reaction was diluted with ethyl acetate and water. The mixture was then washed with 0.1 M HCl, followed by sat. $NaHCO_3$, dried over Na_2SO_4, concentrated, and purified by silica gel flash chromatography.

General Procedure for De-acylation.

A] By sodium methoxide: Anhydrous methanol was prepared by treating commercially available anhydrous methanol over freshly activated 4 Å molecular sieves for 24 h. The methyl ester containing oligosaccharide (10 μmol) was dissolved in anhydrous DCM (2 mL) and anhydrous methanol (2 mL). A sodium methoxide solution was prepared by adding sodium metal to a portion of anhydrous methanol. This fresh sodium methoxide solution was added to the oligosaccharide solution until the pH value reached 12. The reaction was maintained at pH = 12 and stirred at room temperature for 2 h. After the reaction was confirmed complete by TLC, it was

quenched to pH = 7 by a 1 M acetic acid solution in dry methanol. The quenched reaction was concentrated and purified by silica gel chromatography.

B] By magnesium methoxide for selective deprotection of acetate in presence of benzoyl-group. To the solution of disaccharide (4.5 g, 4.1 mmol) in DCM (50 mL) at -20 °C was added magnesium methoxide (28.5 mL, 6% to 10 wt% in methanol). It was stirred at 0 °C till the reaction was complete. It was then quenched with 1 N AcOH (in DCM), worked up and purified by silica gel chromatography.

General Procedure for Oxidation of Allyl Group to Carboxyl Group

To a solution of oligosaccharide (28 μmol) in 3:1 of acetone/water (3.5 mL) under N_2 atmosphere at 0 ^oC, *N*-methylmorpholine oxide (NMO, 36 mg, 31 μmol) and OsO_4 (0.84 mL, 9 μmol) were added. After the starting material was consumed completely, a saturated aqueous solution of Na_2SO_3 was added, and stirred for another 30 min. The mixture was diluted by CH_2Cl_2, and the water phase was separated. The organic phase was washed with brine three times, dried by Na_2SO_4, and concentrated. The residue was dissolved in 2/1/1/1 of Et_2O/THF/MeOH/H_2O, followed by the addition of $NaIO_4$ (72 mg, 37 μmol). The mixture was stirred at room temperature overnight. Then the solvent was removed, and the residue was dissolved in acetonitrile. A solution of NaH_2PO_4 (0.7 M, 1.6 mL) was added, followed by the addition of H_2O_2 (205 μL) and aqueous $NaClO_2$ (690 μL). After the reaction was completed, the mixture was diluted by CH_2Cl_2, and the water phase was separated. The organic phase was washed with brine three times, dried by Na_2SO_4, concentrated, and purified by silica gel flash chromatography.

General Procedure for Sulfation:

a] *O*-sulfation-To a solution of oligosaccharide with four hydroxy groups to be sulfated, (38 mg, 25 μmol) in DMF (1 mL) was added sulfur trioxide-pyridine complex (159 mg, 0.66 mmol). Reaction mixture was stirred at 55 °C. Once the reaction was completed, it was purified by size exclusion chromatography (LH-20) and passed through Na-resin for the ion-exchange to afford *O*-sulfated product.

b] *N*-sulfation only- To a solution of oligosaccharide (11 mg) in methanol (1.0 mL) and triethyl amine (0.3 mL), was added SO_3.pyr (34 mg). The reaction mixture was stirred at 55 °C. Once the reaction was completed, it was purified by size exclusion chromatography (LH-20) and passed through Na-resin for the ion-exchange to afford *N*-sulfated product.

General Procedure of Saponification:

To a solution of oligosaccharide (37 mg) in methanol (0.5 mL) and THF (0.5 mL) at 0°C was added hydrogen peroxide (125 µL) and 1 M aqueous solution of lithium hydroxide (250 µL). The reaction mixture was stirred overnight, which was then neutralized with Amberlite IR-120 acidic resin, filtered, concentrated and redissolved in methanol (0.5 mL). To this solution, ammonium hydroxide (2.5 mL) was added and stirred for another 20 h. All organic solvents were

then removed through rotary evaporation. The desired product was then purified by size exclusion column (LH-20).

General Procedure for Selective Deprotection of Levulinoyl Ester:

To the solution of oligosaccharide (350 mg) in pyridine:acetic acid (2.5 mL, 3:2) at 0°C was added hydrazine monohydrate (80 μL). The reaction mixture was stirred for 3h. After completion of the reaction, the mixture was concentrated to remove all organic solvents, and the residue was dissolved with ethyl acetate, washed with water, dried over sodium sulfate, and purified by silica gel flash chromatography.

General Procedure for Hydrogenation:

To a solution of oligosaccharide (7 mg) in 1.5 mL *t*-butanol:water (2:1) was added palladium hydroxide (12 mg). The reaction mixture was stirred under a hydrogen balloon overnight. It was then filtered through celite, and purified by size exclusion column (G-10) to afford the desired product.

Scheme S1. Syntheses of disaccharides **13** and **14**.

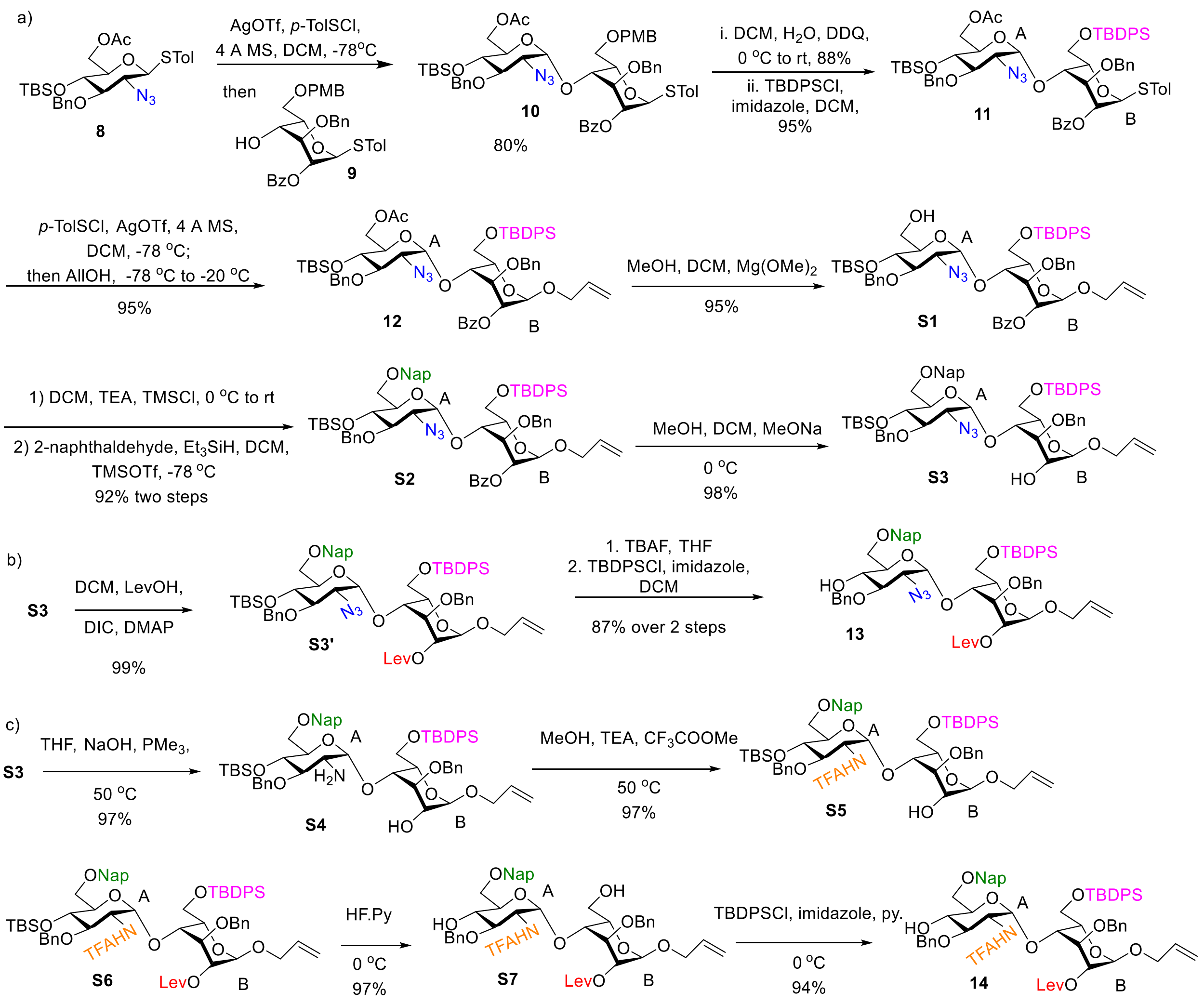

***p*-Tolyl 6-*O*-acetyl-2-azido-3-*O*-benzyl-4-*O*-tert-butyldimethylsilyl-2-deoxy-*α*-D-glucopyranosyl-(1→4)-2-*O*-benzoyl-3-*O*-benzyl-6-*O*-tert-butyldiphenylsilyl-1-thio-*α*-L-idopyranoside (11):** Compound **10** was prepared following the pre-activation based glycosylation method using donor **8** and acceptor **9** in 80% yield. The NMR and MS data of **10** matched the literature values.[1] Compound **11** was prepared from compound **10** (15.13 g, 16.6 mmol) using the general procedure for PMB deprotection followed by the general procedure for TBDPS ether formation, followed by silica gel flash chromatography (6:1 hexanes: EtOAc) to give 95% yield of compound **11**. $[\alpha]_D^{20}$ -1.5° (*c* 1.0, DCM); ^{1}H NMR (500 MHz, $CDCl_3$) δ 8.13 – 8.08 (m, 2H, Ar-H), 7.75 – 7.66 (m, 4H, Ar-H), 7.46 – 7.32 (m, 15H, Ar-H), 7.30 – 7.27 (m, 3H, Ar-H), 7.26 – 7.20 (m, 1H, Ar-H), 7.16 – 7.10 (m, 2H, Ar-H), 6.98 (d, *J* = 7.9 Hz, 2H, Ar-H), 5.58 (d, *J* = 2.4 Hz, 1H, H-1B), 5.36 (t, *J* = 3.0 Hz, 1H, H-2B), 4.94 (d, *J* = 11.7 Hz, 1H, $PhCH_2$), 4.81 – 4.73 (m, 2H, H-5B, $PhCH_2$), 4.68 (d, *J* = 3.6 Hz, 1H, H-1A), 4.26 (d, *J* = 11.3 Hz, 1H, $PhCH_2$), 4.18 (t, *J* = 3.7 Hz, 1H, H-3B), 4.09 (d, *J* = 11.3 Hz, 1H, $PhCH_2$), 4.05 – 3.90 (m, 3H, H-6aA, H-6B), 3.85 (dd, *J* =

12.1, 4.2 Hz, 1H, H-6bA), 3.72 (t, *J* = 3.3 Hz, 1H, H-4B), 3.61 (ddd, *J* = 9.6, 4.3, 2.3 Hz, 1H, H-5A), 3.49 (dd, *J* = 9.6, 8.3 Hz, 1H, H-4A), 3.30 (dd, *J* = 10.2, 8.3 Hz, 1H, H-3A), 3.25 (dd, *J* = 10.2, 3.6 Hz, 1H, H-2A), 2.28 (s, 3H, $SPhCH_3$), 1.92 (s, 3H, CH_3CO), 1.07 (s, 9H, $C(CH_3)_3$), 0.83 (s, 9H, $C(CH_3)_3$), -0.07 (s, 3H, $SiCH_3$), -0.14 (s, 3H, $SiCH_3$); ^{13}C NMR (125 MHz, $CDCl_3$) δ 170.47, 165.72, 137.78, 137.52, 137.50, 135.63, 135.58, 133.16, 133.05, 133.02, 132.63, 131.68, 129.99, 129.82, 129.77, 129.56, 128.41, 128.36, 128.04, 127.99, 127.85, 127.83, 127.81, 127.26, 126.97, 98.27, 86.65, 80.47, 74.86, 74.59, 72.71, 72.30, 71.25, 70.69, 70.53, 69.63, 64.61, 63.57, 62.78, 26.90, 25.90, 21.11, 20.75, 19.22, 17.95, -3.83, -5.09; HRMS (ESI) Anal. Calcd for $C_{64}H_{77}N_3O_{11}SSi_2$ $[M+NH_4]^+$: 1169.5156, found 1169.5107.

Allyl 6-*O*-acetyl-2-azido-3-*O*-benzyl-4-*O*-tert-butyldimethylsilyl-2-deoxy-*α*-D-glucopyranosyl-(1→4)-2-*O*-benzoyl-3-*O*-benzyl-6-*O*-tert-butyldiphenylsilyl-*α*-L-idopyranoside (12): Compound **12** was prepared from compound **11** (1.29 g, 1.12 mmol) using the general procedure for pre-activation based glycosylation, followed by silica gel chromatography (10:1 hexanes: EtOAc) to yield 95% of compound **12**. $[\boldsymbol{\alpha}]_{\mathbf{D}}^{\mathbf{20}}$ +25.3º (*c* 1.0, DCM); ^1H NMR (500 MHz, $CDCl_3$) δ 8.16 – 8.09 (m, 2H, Ar-H), 7.77 – 7.67 (m, 4H, Ar-H), 7.47 – 7.27 (m, 16H, Ar-H), 7.26 – 7.21 (m, 1H, Ar-H), 7.17 – 7.12 (m, 2H, Ar-H), 5.96 – 5.82 (m, 1H, $CH_2CH{=}CH_2$), 5.33 – 5.25 (m, 1H, $CH_2CH{=}CH_2$), 5.20 – 5.12 (m, 3H, $CH_2CH{=}CH_2$, H-1B, H-2B), 4.92 (d, *J* = 11.8 Hz, 1H, $PhCH_2$), 4.78 (d, *J* = 11.8 Hz, 1H, $PhCH_2$), 4.61 (d, *J* = 3.7 Hz, 1H, H-1A), 4.40 – 4.26 (m, 3H, $CH_2CH{=}CH_2$, H-5B, $PhCH_2$), 4.18 (d, *J* = 11.2 Hz, 1H, $PhCH_2$), 4.14 – 4.03 (m, 2H, $CH_2CH{=}CH_2$, H-3B), 4.01 – 3.80 (m, 4H, H-6A, H-6B), 3.67 (t, *J* = 3.0 Hz, 1H, H-4B), 3.58 (ddd, *J* = 9.7, 4.2, 2.2 Hz, 1H, H-5A), 3.49 (t, *J* = 9.0 Hz, 1H, H-4A), 3.35 (dd, *J* = 10.2, 8.5 Hz, 1H, H-3A), 3.22 (dd, *J* = 10.2, 3.7 Hz, 1H, H-2A), 1.91 (s, 3H, CH_3CO), 1.09 (s, 9H, $C(CH_3)_3$), 0.84 (s, 9H, $C(CH_3)_3$), -0.07 (s, 3H, $SiCH_3$), -0.13 (s, 3H, $SiCH_3$); ^{13}C NMR (125 MHz, $CDCl_3$) δ 170.49, 165.75, 137.79, 135.64, 135.52, 133.77, 133.14, 133.11, 133.02, 129.97, 129.86, 129.75, 128.36, 128.28, 128.05, 127.81, 127.79, 127.74, 127.28, 127.00, 117.49, 97.90, 97.19, 80.48, 74.68, 74.50, 72.74, 72.34, 71.13, 70.72, 69.57, 68.45, 67.87, 64.53, 63.78, 62.80, 26.87, 25.91, 20.73, 19.18, 17.95, -3.82, -5.07; HRMS (ESI) Anal. Calcd for $C_{60}H_{75}N_3O_{12}Si_2$ $[M+NH_4]^+$: 1103.5228, found 1103.5179.

Allyl 2-azido-3-*O*-benzyl-4-*O*-tert-butyldimethylsilyl-2-deoxy-*α*-D-glucopyranosyl-(1→4)-2-*O*-benzoyl-3-*O*-benzyl-6-*O*-tert-butyldiphenylsilyl-*α*-L-idopyranoside (S1): Compound **S1** was prepared from compound **12** (638 mg, 0.59 mmol) using the general procedure for transesterification, followed by silica gel chromatography (6:1 hexanes: EtOAc) to yield 95% of compound **S1**. $[\boldsymbol{\alpha}]_{\mathbf{D}}^{\mathbf{20}}$ +11.9º (*c* 1.0, DCM); ^1H NMR (500 MHz, $CDCl_3$) δ 8.15 – 8.11 (m, 2H, Ar-H), 7.78 – 7.67 (m, 4H, Ar-H), 7.51 – 7.28 (m, 16H, Ar-H), 7.26 – 7.20 (m, 1H, Ar-H), 7.16 – 7.11 (m, 2H, Ar-H), 5.96 – 5.82 (m, 1H, $CH_2CH{=}CH_2$), 5.34 – 5.26 (m, 1H, $CH_2CH{=}CH_2$), 5.21 – 5.16 (m, 1H, $CH_2CH{=}CH_2$), 5.15 – 5.12 (m, 2H, H-1B, H-2B), 4.94 (d, *J* = 11.8 Hz, 1H, $PhCH_2$), 4.78 (d, *J* = 11.8 Hz, 1H, $PhCH_2$), 4.51 (d, *J* = 3.8 Hz, 1H, H-1A), 4.38 – 4.23 (m, 3H, $CH_2CH{=}CH_2$, H-5B, $PhCH_2$), 4.16 – 4.02 (m, 3H, H-3B, $CH_2CH{=}CH_2$, $PhCH_2$), 3.96 (dd, *J* = 10.8, 6.8 Hz, 1H, H-6aB), 3.86 (dd, *J* = 10.9, 4.7 Hz, 1H, H-6bB), 3.62 (t, *J* = 3.0 Hz, 1H, H-4B), 3.53 – 3.38 (m,

3H, H-4A, H-5A, H-6aA), 3.38 – 3.28 (m, 2H, H-3A, H-6bA), 3.17 (dd, *J* = 10.2, 3.8 Hz, 1H, H-2A), 1.44 (brs, 1H, OH), 1.10 (s, 9H, $C(CH_3)_3$), 0.86 (s, 9H, $C(CH_3)_3$), 0.02 (s, 3H, $SiCH_3$), -0.12 (s, 3H, $SiCH_3$); ^{13}C NMR (125 MHz, $CDCl_3$) δ 165.76, 137.89, 137.84, 135.70, 135.56, 133.75, 133.24, 133.12, 133.11, 130.02, 129.83, 129.81, 129.75, 128.39, 128.29, 128.03, 127.81, 127.79, 127.77, 127.23, 127.02, 117.61, 98.35, 97.16, 80.61, 74.92, 74.67, 73.40, 72.64, 72.46, 70.25, 69.62, 68.22, 67.88, 64.69, 63.74, 60.65, 26.90, 25.96, 19.20, 17.98, -3.93, -4.91; HRMS (ESI) Anal. Calcd for $C_{58}H_{73}N_3O_{11}Si_2$ $[M+NH_4]^+$: 1061.5122, found 1061.5085.

Allyl 2-azido-3-*O*-benzyl-4-*O*-tert-butyldimethylsilyl-2-deoxy-6-*O*-(2-naphthylmethyl)-*α*-D-glucopyranosyl-(1→4)-2-*O*-benzoyl-3-*O*-benzyl-6-*O*-tert-butyldiphenylsilyl-*α*-L-idopyranoside (S2): Compound **S2** was prepared from compound **S1** (535 mg, 0.51 mmol) using the general procedure for 2-naphthylmethyl ether formation, followed by silica gel chromatography (15:1 hexanes: EtOAc) to yield 92% of compound **S2**. $[\alpha]_D^{20}$ +35.0º (*c* 1.0, DCM); ^{1}H NMR (500 MHz, $CDCl_3$) δ 8.16 – 8.11 (m, 2H, Ar-H), 7.85 – 7.66 (m, 9H, Ar-H), 7.48 – 7.29 (m, 17H, Ar-H), 7.26 – 7.22 (m, 1H, Ar-H), 7.18 – 7.15 (m, 2H, Ar-H), 5.95 – 5.84 (m, 1H, $CH_2CH{=}CH_2$), 5.34 – 5.26 (m, 1H, $CH_2CH{=}CH_2$), 5.21 – 5.14 (m, 3H, $CH_2CH{=}CH_2$, H-1B, H-2B), 4.91 (d, *J* = 11.7 Hz, 1H, $ArCH_2$), 4.79 (d, *J* = 11.7 Hz, 1H, $ArCH_2$), 4.65 (d, *J* = 3.7 Hz, 1H, H-1A), 4.51 (d, *J* = 12.3 Hz, 1H, $ArCH_2$), 4.47 (d, *J* = 12.3 Hz, 1H, $ArCH_2$), 4.41 – 4.28 (m, 3H, $CH_2CH{=}CH_2$, H-5B, $ArCH_2$), 4.21 (d, *J* = 11.3 Hz, 1H, $ArCH_2$), 4.14 (t, *J* = 3.6 Hz, 1H, H-3B), 4.10 (ddt, *J* = 13.3, 6.5, 1.3 Hz, 1H, $CH_2CH{=}CH_2$), 4.04 (dd, *J* = 11.0, 7.0 Hz, 1H, H-6aB), 3.90 (dd, *J* = 11.0, 3.8 Hz, 1H, H-6bB), 3.72 (t, *J* = 3.3 Hz, 1H, H-4B), 3.64 (t, *J* = 9.0 Hz, 1H, H-4A), 3.55 – 3.49 (m, 1H, H-5A), 3.43 (dd, *J* = 10.8, 3.5 Hz, 1H, H-6aA), 3.37 (dd, *J* = 10.2, 8.5 Hz, 1H, H-3A), 3.26 (dd, *J* = 10.2, 3.7 Hz, 1H, H-2A), 3.17 (dd, *J* = 10.9, 2.1 Hz, 1H, H-6bA), 1.09 (s, 9H, $C(CH_3)_3$), 0.81 (d, *J* = 1.1 Hz, 9H, $C(CH_3)_3$), -0.05 (s, 3H, $SiCH_3$), -0.12 (s, 3H, $SiCH_3$); ^{13}C NMR (125 MHz, $CDCl_3$) δ 165.77, 138.00, 137.85, 135.68, 135.58, 135.55, 133.82, 133.29, 133.21, 133.16, 133.07, 132.87, 130.07, 129.77, 129.76, 128.34, 128.28, 128.11, 128.03, 127.95, 127.83, 127.79, 127.77, 127.75, 127.64, 127.19, 126.99, 125.99, 125.71, 125.43, 117.48, 109.99, 98.10, 97.20, 80.73, 74.56, 74.43, 73.31, 73.05, 72.98, 72.44, 70.40, 69.81, 68.88, 68.09, 67.87, 64.57, 64.11, 26.88, 25.99, 19.20, 18.01, -3.86, -4.97; HRMS (ESI) Anal. Calcd for $C_{69}H_{81}N_3O_{11}Si_2$ $[M+Na]^+$: 1206.5302, found 1206.5251.

Allyl 2-azido-3-*O*-benzyl-4-*O*-tert-butyldimethylsilyl-2-deoxy-6-*O*-(2-naphthylmethyl)-*α*-D-glucopyranosyl-(1→4)-3-*O*-benzyl-6-*O*-tert-butyldiphenylsilyl-*α*-L-idopyranoside (S3): Compound **S3** was prepared from compound **S2** (552 mg, 0.47 mmol) using the general procedure for de-acylation using sodium methoxide, followed by silica gel chromatography (10:1 hexanes: EtOAc) to yield 98% of compound **S3**. $[\alpha]_D^{20}$ +31.3º (*c* 1.0, DCM); ^{1}H NMR (500 MHz, $CDCl_3$) δ 7.86 – 7.77 (m, 3H, Ar-H), 7.72 – 7.67 (m, 3H, Ar-H), 7.66 – 7.63 (m, 2H, Ar-H), 7.50 – 7.45 (m, 2H, Ar-H), 7.43 – 7.28 (m, 17H, Ar-H), 5.96 – 5.77 (m, 1H, $CH_2CH{=}CH_2$), 5.30 – 5.22 (m, 1H, $CH_2CH{=}CH_2$), 5.19 – 5.12 (m, 1H, $CH_2CH{=}CH_2$), 5.01 (d, *J* = 3.0 Hz, 1H, H-1A), 4.95 (s, 1H, H-1B), 4.85 (d, *J* = 11.5 Hz, 1H, $ArCH_2$), 4.81 – 4.74 (m, 2H, $ArCH_2$), 4.61 – 4.48 (m, 3H, $ArCH_2$), 4.33 (t, *J* = 6.3 Hz, 1H, H-4B), 4.24 – 4.14 (m, 1H, $CH_2CH{=}CH_2$), 4.00 – 3.75 (m, 7H,

CH_2CH=CH$_2$, H-4A, H-2B, H-3B, H-5B, H-6B), 3.66 – 3.54 (m, 4H, H-2A, H-3A, H-5A, OH), 3.51 (dd, J = 10.7, 3.3 Hz, 1H, H-6aA), 3.32 (dd, J = 10.7, 2.0 Hz, 1H, H-6bA), 1.04 (s, 9H, C(CH_3)$_3$), 0.83 (s, 9H, C(CH_3)$_3$), 0.00 (s, 3H, SiCH_3), -0.03 (s, 3H, SiCH_3); ^{13}C NMR (125 MHz, $CDCl_3$) δ 137.96, 137.82, 135.60, 135.46, 134.12, 133.38, 133.22, 133.11, 132.92, 129.77, 129.71, 128.37, 128.21, 128.02, 127.83, 127.76, 127.74, 127.67, 127.36, 127.02, 126.16, 126.05, 125.80, 125.49, 117.03, 99.75, 95.02, 81.18, 75.34, 73.41, 73.23, 71.99, 71.58, 70.65, 70.22, 67.99, 67.76, 67.20, 66.72, 64.73, 63.81, 26.88, 25.97, 19.19, 17.96, -3.83, -4.89; HRMS (ESI) Anal. Calcd for $C_{62}H_{77}N_3O_{10}Si_2$ $[M+NH_4]^+$: 1097.5486, found 1097.5453.

Allyl 2-azido-3-*O*-benzyl-4-*O*-tert-butyldimethylsilyl-2-deoxy-6-*O*-(2-naphthylmethyl)-*α*-D-glucopyranosyl-(1→4)-3-*O*-benzyl-6-*O*-tert-butyldiphenylsilyl-2-*O*-levulinyl-*α*-L-idopyranoside (S3'): Compound **S3'** was prepared from disaccharide compound **S3** (4.0 g, 3.7 mmol) using the general procedure for the levulinyl ester formation, followed by silica gel chromatography (1:1 hexanes: EtOAc) to yield 99% of product **S3'.** ^{1}H NMR (500 MHz, $CDCl_3$) δ 7.83 – 7.75 (m, 3H), 7.73 – 7.66 (m, 4H), 7.49 – 7.44 (m, 2H), 7.42 – 7.25 (m, 18H), 5.90 – 5.81 (m, 1H), 5.28 – 5.22 (m, 1H), 5.16 – 5.12 (m, 1H), 5.02 – 4.95 (m, 3H), 4.83 – 4.79 (m, 2H), 4.74 (d, J = 11.3 Hz, 1H), 4.68 (d, J = 11.8 Hz, 1H), 4.60 (d, J = 12.3 Hz, 1H), 4.54 (d, J = 12.3 Hz, 1H), 4.34 – 4.29 (m, 1H), 4.23 – 4.17 (m, 1H), 4.00 – 3.90 (m, 4H), 3.89 – 3.85 (m, 1H), 3.76 – 3.70 (m, 1H), 3.64 – 3.57 (m, 2H), 3.53 – 3.47 (m, 1H), 3.38 – 3.32 (m, 2H), 2.74 – 2.69 (m, 2H), 2.67 – 2.56 (m, 2H), 2.15 (s, 3H), 1.06 (s, 9H), 0.79 (s, 9H), -0.02 (s, 3H), -0.05 (s, 3H); ^{13}C NMR (125 MHz, $CDCl_3$) δ 206.22, 172.08, 138.17, 137.91, 135.64, 135.55, 135.51, 133.89, 133.30, 133.23, 133.11, 132.89, 129.73, 129.69, 129.03, 128.27, 128.22, 128.21, 128.01, 127.85, 127.80, 127.75, 127.74, 127.65, 127.64, 127.33, 127.04, 126.10, 126.01, 125.73, 125.47, 125.29, 117.16, 96.89, 96.28, 80.62, 74.75, 73.40, 72.73, 72.27, 72.22, 71.35, 70.69, 69.06, 68.49, 68.33, 67.83, 64.26, 63.51, 37.81, 29.79, 27.78, 26.88, 25.96, 19.18, 17.97, -3.85, -4.86; ESI MS Anal. Calcd for $C_{67}H_{84}N_3O_{12}Si_2$ $[M+H]^+$: 1178.56, found 1178.55.

Allyl 2-azido-3-*O*-benzyl-2-deoxy-6-*O*-(2-naphthylmethyl)-*α*-D-glucopyranosyl-(1→4)-3-*O*-benzyl-6-*O*-tert-butyldiphenylsilyl-2-*O*-levulinyl-*α*-L-idopyranoside (13): Compound **13** was prepared from disaccharide compound **S3'** (4.4 g, 3.7 mmol) using general procedure for the removal of silyl group, followed by TBDPS-group protection on primary alcohol, and purification by silica gel chromatography (1:1 hexanes: EtOAc) to yield 87% of product **13** over 2 steps**.** ^{1}H NMR (500 MHz, $CDCl_3$) δ 7.86 – 7.76 (m, 3H), 7.73 – 7.64 (m, 5H), 7.52 – 7.46 (m, 2H), 7.40 – 7.27 (m, 17H), 5.92 – 5.83 (m, 1H), 5.29 – 5.23 (m, 1H), 5.17 – 5.14 (m, 1H), 5.01 – 4.97 (m, 2H), 4.96 (d, J = 1.9 Hz, 1H), 4.85 – 4.76 (m, 3H), 4.68 (d, J = 11.8 Hz, 1H), 4.61 (d, J = 12.1 Hz, 1H), 4.52 (d, J = 12.1 Hz, 1H), 4.29 (td, J = 5.8, 2.4 Hz, 1H), 4.24 – 4.18 (m, 1H), 4.01 – 3.94 (m, 2H), 3.92 – 3.84 (m, 3H), 3.76 – 3.66 (m, 2H), 3.65 – 3.60 (m, 1H), 3.51 (dd, J = 10.3, 3.8 Hz, 1H), 3.39 (dd, J = 10.4, 4.1 Hz, 1H), 3.33 (dd, J = 9.8, 3.6 Hz, 1H), 2.78 – 2.58 (m, 4H), 2.35 (d, J = 2.9 Hz, 1H), 2.17 (s, 3H), 1.05 (s, 9H); ^{13}C NMR (125 MHz, $CDCl_3$) δ 206.27, 172.09, 138.12, 137.88, 135.67, 135.55, 135.03, 133.88, 133.25, 133.16, 133.00, 129.75, 129.71, 128.58, 128.29, 127.96, 127.94, 127.87, 127.76, 127.69, 127.66, 126.60, 126.18, 125.98, 125.55, 117.17, 96.96,

96.18, 80.06, 75.07, 73.76, 72.39, 72.27, 72.04, 71.04, 70.56, 69.30, 69.04, 68.33, 67.91, 63.30, 63.00, 37.84, 29.81, 27.75, 26.86, 19.14; ESI-MS Anal. Calcd for $C_{61}H_{70}N_3O_{12}Si$ $[M+H]^+$: 1064.47, found 1064.46.

Allyl 2-amino-3-*O*-benzyl-4-*O*-tert-butyldimethylsilyl-2-deoxy-6-*O*-(2-naphthylmethyl)-*α*-D-glucopyranosyl-(1→4)-3-*O*-benzyl-6-*O*-tert-butyldiphenylsilyl-*α*-L-idopyranoside (S4): Compound **S4** was prepared from compound **S3** (661 mg, 0.61 mmol) using the general procedure for Staudinger reduction, followed by silica gel chromatography (2:1 hexanes: EtOAc) to yield 96% of compound **S4**. $[\boldsymbol{\alpha}]_{\mathbf{D}}^{\mathbf{20}}$ +37.5º (*c* 1.0, DCM); ^{1}H NMR (500 MHz, $CDCl_3$) δ 7.84 – 7.74 (m, 3H, Ar-H), 7.71 – 7.63 (m, 5H, Ar-H), 7.49 – 7.43 (m, 2H, Ar-H), 7.40 – 7.27 (m, 17H, Ar-H), 5.94 – 5.82 (m, 1H, $CH_2CH{=}CH_2$), 5.30 – 5.21 (m, 1H, $CH_2CH{=}CH_2$), 5.18 – 5.11 (m, 1H, $CH_2CH{=}CH_2$), 4.94 – 4.90 (m, 2H, H-1A, H-1B), 4.87 (d, *J* = 12.0 Hz, 1H, $ArCH_2$), 4.71 (d, *J* = 11.7 Hz, 1H, $ArCH_2$), 4.58 – 4.47 (m, 4H, $ArCH_2$), 4.34 (ddd, *J* = 6.8, 4.9, 1.3 Hz, 1H, H-4B), 4.22 (ddt, *J* = 13.2, 4.8, 1.7 Hz, 1H, $CH_2CH{=}CH_2$), 4.01 – 3.91 (m, 2H, H-5B, $CH_2CH{=}CH_2$), 3.89 – 3.81 (m, 3H, H-3B, H-6B), 3.79 (dt, *J* = 2.5, 1.3 Hz, 1H, H-2B), 3.70 (dd, *J* = 9.5, 8.6 Hz, 1H, H-4A), 3.57 (ddd, *J* = 9.5, 3.9, 2.3 Hz, 1H, H-5A), 3.48 (dd, *J* = 10.6, 3.8 Hz, 1H, H-6aA), 3.42 – 3.30 (m, 2H, H-3A, H-6bA), 2.88 (dd, *J* = 10.3, 3.8 Hz, 1H, H-2A), 2.20 (brs, 3H, NH_2, OH), 1.03 (s, 9H, $C(CH_3)_3$), 0.83 (s, 9H, $C(CH_3)_3$), 0.04 (s, 3H, $SiCH_3$), 0.00 (s, 3H, $SiCH_3$); ^{13}C NMR (125 MHz, $CDCl_3$) δ 138.37, 137.99, 135.62, 135.60, 135.49, 134.27, 133.47, 133.28, 133.23, 132.90, 129.71, 129.68, 128.54, 128.33, 127.98, 127.83, 127.71, 127.66, 127.61, 127.33, 126.16, 126.00, 125.73, 125.55, 116.94, 99.89, 97.06, 83.44, 75.41, 73.37, 73.32, 72.04, 71.50, 70.73, 70.24, 68.33, 67.70, 67.54, 66.89, 64.15, 55.12, 26.86, 26.01, 19.20, 17.96, -3.77, -4.74; HRMS (ESI) Anal. Calcd for $C_{62}H_{79}NO_{10}Si_2$ $[M+H]^+$: 1054.5315, found 1054.5267.

Allyl 3-*O*-benzyl-4-*O*-tert-butyldimethylsilyl-2-deoxy-6-*O*-(2-naphthylmethyl)-2-trifluoroacetamido-*α*-D-glucopyranosyl-(1→4)-3-*O*-benzyl-6-*O*-*tert*-butyldiphenylsilyl-*α*-L-idopyranoside (S5): Compound **S5** was prepared from compound **S4** (619 mg, 0.59 mmol) using the general procedure for TFA protection of amine, followed by silica gel chromatography (6:1 hexanes: EtOAc) to yield 97% of compound **S5**. $[\boldsymbol{\alpha}]_{\mathbf{D}}^{\mathbf{20}}$ +24.4º (*c* 1.0, DCM); ^{1}H NMR (500 MHz, $CDCl_3$) δ 7.85 – 7.77 (m, 3H, Ar-H), 7.73 – 7.67 (m, 3H, Ar-H), 7.67 – 7.63 (m, 2H, Ar-H), 7.51 – 7.45 (m, 2H, Ar-H), 7.44 – 7.28 (m, 15H, Ar-H, NHCO), 7.27 – 7.19 (m, 3H, Ar-H), 5.99 – 5.83 (m, 1H, $CH_2CH{=}CH_2$), 5.35 – 5.26 (m, 1H, $CH_2CH{=}CH_2$), 5.24 – 5.17 (m, 1H, $CH_2CH{=}CH_2$), 4.92 (s, 1H, H-1B), 4.73 (d, *J* = 3.4 Hz, 1H, H-1A), 4.71 – 4.61 (m, 2H, $ArCH_2$), 4.57 – 4.45 (m, 4H, $ArCH_2$), 4.39 – 4.20 (m, 3H, H-2A, H-5B, $CH_2CH{=}CH_2$), 4.05 (ddt, *J* = 13.1, 6.5, 1.4 Hz, 1H, $CH_2CH{=}CH_2$), 3.96 (dd, *J* = 10.9, 7.5 Hz, 1H, H-6aB), 3.90 – 3.78 (m, 3H, H-4A, H-2B, H-6bB), 3.72 – 3.66 (m, 1H, H-4B), 3.65 – 3.56 (m, 2H, H-5A, H-3B), 3.52 (dd, *J* = 10.1, 8.1 Hz, 1H, H-3A), 3.45 (dd, *J* = 10.7, 3.7 Hz, 1H, H-6aA), 3.24 (dd, *J* = 10.6, 2.6 Hz, 1H, H-6bA), 2.30 (brs, 1H, OH), 1.05 (s, 9H, $C(CH_3)_3$), 0.83 (s, 9H, $C(CH_3)_3$), -0.02 (s, 3H, $SiCH_3$), -0.03 (s, 3H, $SiCH_3$); ^{13}C NMR (125 MHz, $CDCl_3$) δ 157.39 (q, *J* = 37.4 Hz), 137.49, 137.33, 135.57, 135.51, 135.47, 133.78, 133.30, 133.23, 133.14, 132.91, 129.78, 129.76, 128.47, 128.37, 128.02, 127.86, 127.77, 127.74, 127.66, 127.62, 127.56, 126.03, 126.01, 125.75, 125.42, 117.55, 115.69 (q, *J* = 286.8 Hz),

99.02, 96.44, 79.32, 74.99, 74.04, 73.29, 72.84, 72.76, 71.90, 70.25, 68.02, 67.87, 67.85, 67.25, 64.23, 52.89, 26.81, 25.95, 19.19, 17.96, -3.93, -4.99; HRMS (ESI) Anal. Calcd for $C_{64}H_{78}F_3NO_{11}Si_2$ $[M+H]^+$: 1167.5404, found 1167.5353.

Allyl 3-*O*-benzyl-4-*O*-tert-butyldimethylsilyl-2-deoxy-6-*O*-(2-naphthylmethyl)-2-trifluoroacetamido-*α*-D-glucopyranosyl-(1→4)-3-*O*-benzyl-6-*O*-tert-butyldiphenylsilyl-2-*O*-levulinyl-*α*-L-idopyranoside (S6): Compound **S6** was prepared from compound **S5** (632 mg, 0.55 mmol) using the general procedure for levulinyl ester formation, followed by silica gel chromatography (4:1 hexanes: EtOAc) to yield 99% of compound **S6**. $[\alpha]_D^{20}$ +18.6º (*c* 1.0, DCM); ^{1}H NMR (500 MHz, $CDCl_3$) δ 7.83 – 7.67 (m, 9H, Ar-H), 7.49 – 7.33 (m, 10H, Ar-H), 7.31 – 7.26 (m, 4H, Ar-H), 7.26 – 7.17 (m, 4H, Ar-H), 7.13 (d, *J* = 9.5 Hz, 1H, NHCO), 5.94 – 5.76 (m, 1H, $CH_2CH{=}CH_2$), 5.27 – 5.19 (m, 1H, $CH_2CH{=}CH_2$), 5.17 – 5.10 (m, 1H, $CH_2CH{=}CH_2$), 5.04 – 4.97 (m, 2H, H-1A, H-2B), 4.88 (d, *J* = 2.1 Hz, 1H, H-1B), 4.72 (d, *J* = 11.9 Hz, 1H, $ArCH_2$), 4.62 – 4.51 (m, 4H, $ArCH_2$), 4.45 (d, *J* = 11.4 Hz, 1H, $ArCH_2$), 4.39 – 4.29 (m, 2H, H-2A, H-5B), 4.20 (ddt, *J* = 13.2, 4.8, 1.7 Hz, 1H, $CH_2CH{=}CH_2$), 4.01 – 3.87 (m, 5H, $CH_2CH{=}CH_2$, H-3B, H-4B, H-6B), 3.78 (t, *J* = 8.5 Hz, 1H, H-4A), 3.71 (ddd, *J* = 9.0, 4.8, 2.6 Hz, 1H, H-5A), 3.56 – 3.47 (m, 2H, H-3A, H-6aA), 3.42 (dd, *J* = 10.5, 2.6 Hz, 1H, H-6bA), 2.70 (qdd, *J* = 18.5, 7.4, 5.0 Hz, 2H, $COCH_2$), 2.44 (ddd, *J* = 17.1, 6.6, 5.0 Hz, 1H, $COCH_2$), 2.39 – 2.28 (m, 1H, $COCH_2$), 2.13 (s, 3H, CH_3CO), 1.07 (s, 9H, $C(CH_3)_3$), 0.79 (s, 9H, $C(CH_3)_3$), -0.03 (s, 3H, $SiCH_3$), -0.07 (s, 3H, $SiCH_3$); ^{13}C NMR (125 MHz, $CDCl_3$) δ 206.41, 171.76, 156.95 (q, *J* = 36.6 Hz), 137.59, 137.56, 135.61, 135.54, 135.51, 133.78, 133.22, 133.13, 133.10, 132.91, 129.82, 128.34, 128.25, 128.05, 127.87, 127.80, 127.76, 127.75, 127.66, 127.63, 127.47, 127.15, 126.09, 126.02, 125.74, 125.45, 117.12, 115.70 (q, *J* = 286.6 Hz), 97.18, 95.45, 80.12, 74.51, 73.84, 73.40, 72.77, 72.30, 71.66, 70.36, 69.69, 68.56, 68.40, 67.96, 63.38, 52.91, 37.87, 29.63, 27.72, 26.91, 26.85, 25.91, 19.17, 17.97, -3.98, -4.85; HRMS (ESI) Anal. Calcd for $C_{69}H_{84}F_3NO_{13}Si_2$ $[M+H]^+$: 1270.5326, found 1270.5291.

Allyl 3-*O*-benzyl-6-*O*-(2-naphthylmethyl)-2-deoxy-2-trifluoroacetamido-*α*-D-glucopyranosyl-(1→4)-3-*O*-benzyl-2-*O*-levulinyl-*α*-L-idopyranoside (S7): Compound **S7** was prepared from compound **S6** (652 mg, 0.52 mmol) using the general procedure for removal of silyl group, followed by silica gel chromatography (1:1 hexanes: EtOAc) to yield 97% of compound **S7**. $[\alpha]_D^{20}$ -2.8º (*c* 1.0, DCM); ^{1}H NMR (500 MHz, $CDCl_3$) δ 7.88 – 7.82 (m, 3H, Ar-H), 7.78 (s, 1H, Ar-H), 7.53 – 7.44 (m, 4H, Ar-H, NHCO), 7.36 – 7.28 (m, 8H, Ar-H), 7.27 – 7.24 (m, 2H, Ar-H), 5.99 – 5.83 (m, 1H, $CH_2CH{=}CH_2$), 5.35 – 5.27 (m, 1H, $CH_2CH{=}CH_2$), 5.23 – 5.17 (m, 1H, $CH_2CH{=}CH_2$), 5.00 (d, *J* = 2.2 Hz, 1H, H-2B), 4.92 (d, *J* = 3.7 Hz, 1H, H-1A), 4.82 (s, 1H, H-1B), 4.80 – 4.70 (m, 4H, $ArCH_2$), 4.64 (d, *J* = 11.5 Hz, 1H, $ArCH_2$), 4.53 (d, *J* = 11.9 Hz, 1H, $ArCH_2$), 4.36 – 4.18 (m, 3H, $CH_2CH{=}CH_2$, H-2A, H-5B), 4.08 – 3.95 (m, 2H, $CH_2CH{=}CH_2$, H-5A), 3.86 – 3.65 (m, 9H, H-3A, H-4A, H-6A, H-3B, H-4B, H-6B, OH), 2.80 (ddd, *J* = 18.7, 8.1, 5.3 Hz, 1H, $COCH_2$), 2.68 (dt, *J* = 18.6, 5.6 Hz, 1H, $COCH_2$), 2.55 (brs, 1H, OH), 2.52 – 2.41 (m, 2H, $COCH_2$), 2.12 (s, 3H, CH_3CO); ^{13}C NMR (125 MHz, $CDCl_3$) δ 207.09, 171.76, 157.24 (q, *J* = 37.1 Hz), 137.97, 137.22, 134.76, 133.62, 133.19, 133.08, 128.56, 128.42, 128.41, 127.94, 127.92, 127.91, 127.74, 127.72, 127.55, 126.82, 126.25, 126.08, 125.63, 117.44, 115.83 (q, *J* =

286.6 Hz), 97.80, 97.11, 79.48, 74.17, 73.88, 73.38, 72.82, 72.06, 71.94, 71.54, 69.86, 68.85, 68.38, 67.00, 61.10, 52.95, 37.98, 29.53, 27.82; HRMS (ESI) Anal. Calcd for $C_{47}H_{52}F_3NO_{13}$ $[M+H]^+$: 913.3729, found 913.3707.

Allyl 3-*O*-benzyl-2-deoxy-6-*O*-(2-naphthylmethyl)-2-trifluoroacetamido-*α*-D-glucopyranosyl-(1→4)-3-*O*-benzyl-6-*O*-tert-butyldiphenylsilyl-2-*O*-levulinyl-*α*-L-idopyranoside (14): Compound **14** was prepared from compound **S7** (448 mg, 0.50 mmol) using the general procedure for TBDPS ether formation, followed by silica gel chromatography (3:1 hexanes: EtOAc) to yield 94% of compound **14**. $[\alpha]_D^{20}$ -0.2° (*c* 1.0, DCM); ^{1}H NMR (500 MHz, $CDCl_3$) δ 7.87 – 7.79 (m, 3H, Ar-H), 7.74 – 7.67 (m, 5H, Ar-H, NHCO), 7.52 – 7.47 (m, 3H, Ar-H), 7.42 – 7.28 (m, 14H, Ar-H), 7.27 – 7.22 (m, 3H, Ar-H), 5.95 – 5.77 (m, 1H, $CH_2CH{=}CH_2$), 5.31 – 5.23 (m, 1H, $CH_2CH{=}CH_2$), 5.19 – 5.13 (m, 1H, $CH_2CH{=}CH_2$), 5.04 – 4.97 (m, 2H, H-1A, H-2B), 4.89 (s, 1H, H-1B), 4.78 (d, *J* = 11.8 Hz, 1H, $ArCH_2$), 4.72 (d, *J* = 11.5 Hz, 1H, $ArCH_2$), 4.61 (d, *J* = 12.0 Hz, 1H, $ArCH_2$), 4.57 – 4.47 (m, 3H, $ArCH_2$), 4.32 (td, *J* = 5.9, 2.0 Hz, 1H, H-5B), 4.28 – 4.19 (m, 2H, $CH_2CH{=}CH_2$, H-2A), 3.99 (ddt, *J* = 13.2, 6.2, 1.4 Hz, 1H, $CH_2CH{=}CH_2$), 3.91 – 3.73 (m, 6H, H-4A, H-5A, H-3B, H-4B, H-6B), 3.63 (dd, *J* = 10.6, 8.7 Hz, 1H, H-3A), 3.53 (dd, *J* = 10.3, 3.9 Hz, 1H, H-6aA), 3.39 (dd, *J* = 10.2, 4.6 Hz, 1H, H-6bA), 2.81 (dt, *J* = 18.6, 6.8 Hz, 1H, $COCH_2$), 2.68 – 2.58 (m, 2H, $COCH_2$, OH), 2.42 (dd, *J* = 6.8, 5.3 Hz, 2H, $COCH_2$), 2.09 (s, 3H, CH_3CO), 1.06 (s, 9H, $C(CH_3)_3$); ^{13}C NMR (125 MHz, $CDCl_3$) δ 207.13, 171.87, 157.21 (q, *J* = 37.0 Hz), 138.14, 137.55, 135.63, 135.57, 134.98, 133.76, 133.18, 133.09, 133.06, 133.02, 129.83, 129.82, 128.48, 128.35, 128.33, 127.89, 127.83, 127.78, 127.74, 127.72, 127.61, 127.45, 126.59, 126.21, 126.01, 125.53, 117.20, 115.84 (q, *J* = 286.6 Hz), 97.21, 96.71, 79.44, 74.09, 73.82, 73.01, 72.65, 72.30, 72.21, 70.61, 69.77, 69.01, 67.91, 67.84, 63.41, 53.00, 37.99, 29.48, 27.81, 26.80, 19.12; HRMS (ESI) Anal. Calcd for $C_{63}H_{70}F_3NO_{13}Si$ $[M+NH_4]^+$: 1151.4907, found 1151.4869.

Scheme S2. Synthesis of disaccharide **15**.

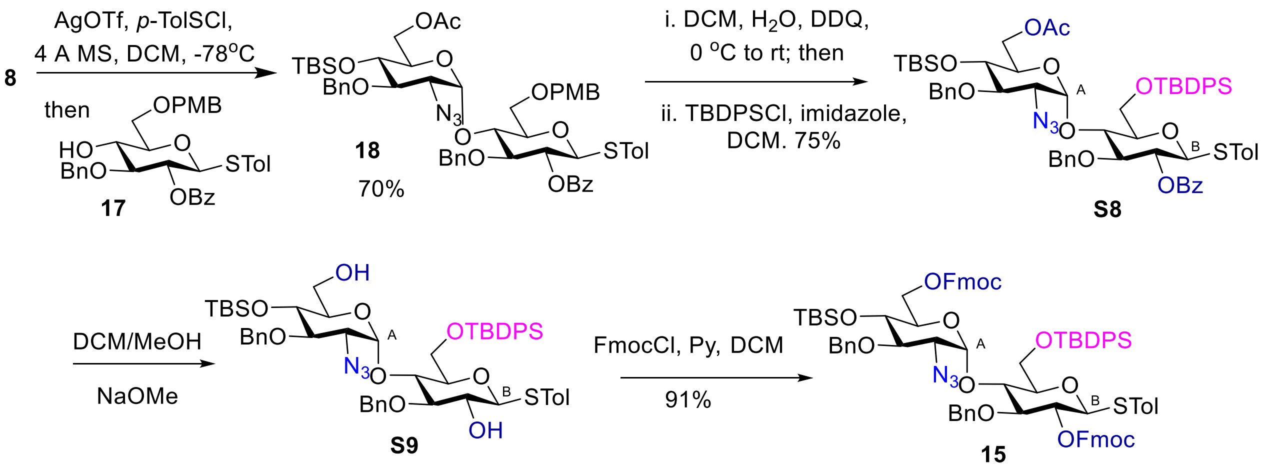

***p*-Tolyl 6-*O*-acetyl-2-azido-3-*O*-benzyl-4-*O*-tert-butyldimethylsilyl-2-deoxy-*α*-D-glucopyranosyl-(1→4)-2-*O*-benzoyl-3-*O*-benzyl-6-*O*-*O*-tert-butyldiphenylsilyl-1-thio-*β*-D-**

glucopyranoside S8: Compound **18** was prepared following the pre-activation based glycosylation method using donor **8** and acceptor **17**[1] in 70% yield. To a solution of compound **18** (2.51 g, 2.4 mmol) in DCM/H_2O (10:1, 30 mL), DDQ (1.1 g, 4.8 mmol) was added. The reaction mixture was stirred at room temperature and after completion (≈3 h), it was quenched with saturated aqueous $NaHCO_3$ solution and diluted with DCM. The organic phase was washed with H_2O until the solution became colorless. The solvent was dried over Na_2SO_4, concentrated *in vacuo* and the residue was purified by silica gel column chromatography (hexanes: EtOAc, 6:1 to 1:2) to afford *p*-tolyl 6-*O*-acetyl-2-azido-3-*O*-benzyl-4-*O*-tert-butyldimethylsilyl-2-deoxy-*α*-D-glucopyranosyl-(1→4)-2-*O*-benzoyl-3-*O*-benzyl-1-thio-*β*-D-glucopyranoside as white solid (1.77 g, 1.9 mmol, 80%). Then the product was dissolved in DCM and subjected to the general procedure for TBDPS ether formation, followed by silica gel chromatography (6:1 hexanes: EtOAc) to yield 75% of compound **S8**. The NMR and MS data of **18** and **S8** matched the literature values.[2]

***p*-Tolyl 2-azido-3-*O*-benzyl-4-*O*-tert-butyldimethylsilyl-2-deoxy-α-D-glucopyranosyl-(1→4)-3-*O*-benzyl-6-*O*-tert-butyldiphenylsilyl-1-thio-β-D-glucopyranoside (S9):** Compound **S9** was prepared from **S8** (250 mg, 0.22 mmol) using the general procedure for deacylation using sodium methoxide, followed by silica gel chromatography (8:1 hexanes:EtOAc) to yield 95% of compound **S9**. $[\alpha]_D^{20}$ = +14.0. ^{1}H NMR δH (500 MHz, $CDCl_3$) 7.76 – 7.72 (m, 4H), 7.48 – 7.31 (m, 16H), 7.31 – 7.27 (m, 2H), 7.06 – 7.02 (m, 2H), 5.66 (d, *J* = 4.0 Hz, 1H), 5.12 (d, *J* = 10.8 Hz, 1H), 4.87 – 4.77 (m, 3H, Bn), 4.55 (d, *J* = 10.0 Hz, 1H, H1B), 4.05 (dd, *J* = 11.0, 2.0 Hz, 1H), 3.89 (dd, *J* = 11.0, 6.0 Hz, 1H), 3.80 – 3.72 (m, 2H), 3.61 – 3.51 (m, 4H), 3.41 – 3.30 (m, 3H), 3.14 (dd, *J* = 10.0, 4.0 Hz, 1H), 2.33 (s, 3H, STol-CH_3), 1.11 (s, 9H, $(CH_3)_3CSi$-CH_3), 0.89 (s, 9H, $(CH_3)_3CSi$-CH_3), 0.06 (s, 3H, CH_3Si-CH_3), -0.02 (s, 3H, CH_3Si-CH_3).^{13}C NMR δC (125 MHz, $CDCl_3$) 138.4, 138.1, 138.0, 135.8, 135.6, 133.6, 133.3, 132.6, 129.9, 129.7, 128.7, 128.45, 128.3, 127.8, 127.7, 127.6, 127.5, 127.3, 98.0, 89.2, 86.2, 80.0, 79.7, 75.0, 74.6, 74.2, 73.4, 70.4, 64.1, 63.7, 60.4, 27.0, 26.0, 21.2, 19.4, 18.0, -3.9, -4.8. HRMS $[M + NH^4]^+$ $C_{55}H_{75}N_4O_9SSi_2^+$ calcd. 1023.4878, obsd.1023.4863.

***p*-Tolyl 2-azido-3-*O*-benzyl-4-*O*-tert-butyldimethylsilyl-6-*O*-fluorenylmethyloxycarbonyl-2-deoxy-α-D-glucopyranosyl-(1→4)-3-*O*-benzyl-6-*O*-tert-butyldiphenylsilyl-2-*O*-fluorenylmethyloxycarbonyl-1-thio-β-D-glucopyranoside (15):** Compound **15** was prepared from compound **S9** (220 mg, 0.20 mmol) using the general procedure for Fmoc protection, followed by silica gel chromatography (12:1 hexanes: EtOAc) to yield 94% of compound **15**. ^{1}H NMR δH (500 MHz, $CDCl_3$) 7.78 – 7.70 (m, 8H), 7.65 – 7.54 (m, 5H), 7.44 – 7.33 (m, 16H), 7.30 – 7.28 (m, 3H), 7.26-7.22 (m, 6H), 6.98 (d, *J* = 8.0 Hz, 2H), 5.63 (d, *J* = 4.0 Hz, 1H, H1A), 4.94 – 4.73 (m, 6H), 4.61 – 4.55 (m, 1H), 4.35 – 4.30 (m, 2H), 4.29 – 4.23 (m, 2H), 4.17 (t, *J* = 7.0 Hz, 1H), 4.07 – 3.84 (m, 7H), 3.65 – 3.50 (m, 4H), 3.21 (dd, *J* = 10.0, 4.0 Hz, 1H), 2.29 (s, 3H, STol-CH_3), 1.11 (s, 9H, $(CH_3)_3CSi$-CH_3), 0.86 (s, 9H, $(CH_3)_3CSi$-CH_3), -0.02 (s, 6H, CH_3Si-CH_3).^{13}C NMR δC (125 MHz, $CDCl_3$) 154.7, 154.3, 143.4, 143.3, 143.2, 143.1, 141.3, 141.2, 137. 9, 137.7, 137.6, 135.7, 135.5, 133.3, 133.2, 130.1, 129.8, 129.7, 128.4, 128.3, 127.9, 127.8, 127.6, 127.5,

127.4, 127.2, 127.1, 125.3, 125.2, 125.1, 120.0, 98.0, 87.2, 84.7, 80.0, 79.7, 75.0, 74.6, 74.3, 71.3, 70.6, 70.3, 69.9, 65.6, 64.1, 63.5, 46.7, 46.6, 29.7, 26.9, 25.9, 21.1, 19.3, 18.0, 14.1, -3.9, -5.0. HRMS $[M + NH_4]^+$ $C_{85}H_{95}N_4O_{12}SSi_2^+$ calcd. 1468.6183, obsd.1468.6195.

Scheme S3. Synthesis of tetrasaccharide **23**.

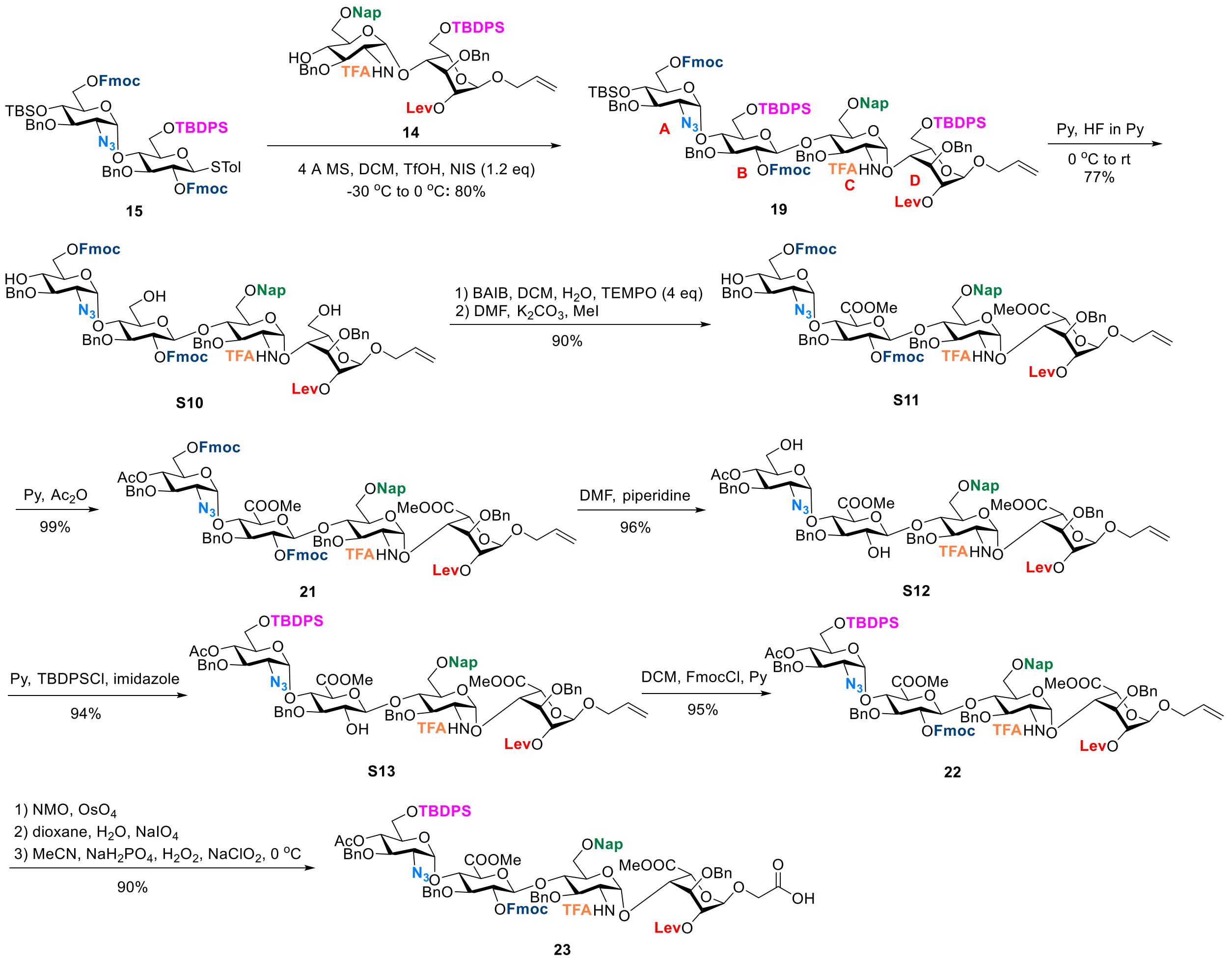

Allyl 2-azido-3-*O*-benzyl-4-*O*-tert-butyldimethylsilyl-2-deoxy-6-*O*-fluorenylmethoxycarbonyl-*α*-D-glucopyranosyl-(1→4)-3-*O*-benzyl-6-*O*-tert-butyldiphenylsilyl-2-*O*-fluorenylmethoxycarbonyl-*β*-D-glucopyranosyl-(1→4)-3-*O*-benzyl-2-deoxy-2-trifluoroacetamido-6-*O*-(2-naphthylmethyl)-*α*-D-glucopyranosyl-(1→4)-3-*O*-benzyl-6-*O*-tert-butyldiphenylsilyl-2-*O*-levulinyl-*α*-L-idopyranoside (19): Compound **19** was prepared from compound **15** (3.41 g, 2.35 mmol) and compound **14** (2.22 g, 1.96 mmol) using the general procedure for non-pre-activation based glycosylation, followed by silica gel chromatography (4:1 hexanes: EtOAc) to yield 80% of compound **19**. $[\alpha]_D^{20}$ +19.2° (*c* 1.0, DCM); ^{1}H NMR (500 MHz, $CDCl_3$) δ 7.82 (d, J = 8.1 Hz, 1H, NHCO), 7.79 – 7.65 (m, 10H, Ar-H), 7.64 – 7.27 (m, 42H, Ar-H), 7.26 – 7.14 (m, 4H, Ar-H), 7.10 – 6.98 (m, 7H, Ar-H), 5.84 – 5.72 (m, 1H,

$CH_2CH=CH_2$), 5.37 (d, *J* = 4.1 Hz, 1H, H-1A), 5.17 (dq, *J* = 17.2, 1.7 Hz, 1H, $CH_2CH=CH_2$), 5.10 (d, *J* = 3.7 Hz, 1H, H-1C), 5.06 (dq, *J* = 10.5, 1.5 Hz, 1H, $CH_2CH=CH_2$), 4.97 (t, *J* = 2.2 Hz, 1H, H-1D), 4.90 – 4.78 (m, 3H, $ArCH_2$), 4.77 – 4.70 (m, 3H, H-2D, $ArCH_2$), 4.55 (dd, *J* = 9.4, 8.1 Hz, 1H, H-2B), 4.50 (d, *J* = 11.7 Hz, 1H, $ArCH_2$), 4.41 – 4.08 (m, 12H, $CH_2CH=CH_2$, H-1B, H-2C, H-5D, $ArCH_2$), 4.08 – 4.03 (m, 2H), 4.01 – 3.92 (m, 3H, H-4C), 3.91 – 3.86 (m, 2H), 3.85 – 3.77 (m, 2H, $CH_2CH=CH_2$, H-3D), 3.76 – 3.58 (m, 7H, H-4A, H-6aB, H-3C, H-4D, H-6D), 3.58 – 3.43 (m, 3H, H-3A, H-4B, H-6bB), 3.28 – 3.22 (m, 2H, H-2A), 3.11 (ddd, *J* = 9.2, 5.4, 3.4 Hz, 1H, H-5B), 2.89 (t, *J* = 9.0 Hz, 1H, H-3B), 2.79 (ddd, *J* = 18.5, 10.0, 4.4 Hz, 1H, $COCH_2$), 2.57 – 2.47 (m, 1H, $COCH_2$), 2.41 (ddd, *J* = 17.0, 10.0, 4.0 Hz, 1H, $COCH_2$), 2.31 (dt, *J* = 17.0, 4.9 Hz, 1H, $COCH_2$), 1.87 (s, 3H, CH_3CO), 1.04 (s, 9H, $C(CH_3)_3$), 1.00 (s, 9H, $C(CH_3)_3$), 0.81 (s, 9H, $C(CH_3)_3$), 0.00 – -0.05 (m, 6H, $SiCH_3$); ^{13}C NMR (125 MHz, $CDCl_3$) δ 207.84, 172.09, 157.27 (q, *J* = 36.9 Hz), 154.83, 154.06, 143.42, 143.28, 143.22, 143.15, 141.33, 141.32, 141.24, 141.21, 138.94, 137.93, 137.83, 137.78, 135.56, 135.47, 134.66, 133.80, 133.23, 133.21, 133.20, 133.11, 132.95, 129.87, 129.85, 129.83, 128.49, 128.31, 128.26, 128.20, 128.19, 127.98, 127.93, 127.91, 127.87, 127.84, 127.83, 127.79, 127.62, 127.58, 127.45, 127.37, 127.31, 127.17, 127.14, 127.12, 127.09, 126.92, 126.85, 126.68, 126.29, 126.00, 125.21, 125.04, 124.68, 124.65, 120.14, 120.12, 119.99, 119.96, 116.92, 115.84 (q, *J* = 286.5 Hz), 99.37, 98.09, 97.65, 97.31, 82.62, 80.33, 78.47, 76.85, 75.64, 75.59, 75.37, 75.21, 75.08, 74.42, 74.13, 73.84, 72.52, 72.16, 71.45, 70.95, 70.70, 69.88, 69.36, 68.58, 67.93, 67.71, 67.18, 65.50, 64.20, 63.90, 63.48, 53.33, 46.69, 46.59, 38.15, 29.14, 27.89, 26.95, 26.78, 25.91, 19.13, 19.11, 17.95, -3.89, -5.09; HRMS (ESI) Anal. Calcd for $C_{141}H_{153}F_3N_4O_{26}Si_3$ $[M+2NH_4]^{2+}$: 1247.5355, found 1247.5320.

Allyl 2-azido-3-*O*-benzyl-2-deoxy-6-*O*-fluorenylmethoxycarbonyl-*α*-D-glucopyranosyl-(1→4)-3-*O*-benzyl-2-*O*-fluorenylmethoxycarbonyl-*β*-D-glucopyranosyl-(1→4)-3-*O*-benzyl-2-deoxy-6-*O*-(2-naphthylmethyl)-2-trifluoroacetamido-*α*-D-glucopyranosyl-(1→4)-3-*O*-benzyl-2-*O*-levulinyl-*α*-L-idopyranoside (S10): Compound **S10** was prepared from compound **19** (1.98 g, 0.80 mmol) using the general procedure for removal of the silyl group, followed by silica gel chromatography (2:1 hexanes: EtOAc) to yield 77% of compound **S10**. $[\alpha]_D^{20}$ -3.3° (*c* 1.0, DCM); ^1H NMR (500 MHz, $CDCl_3$) δ 7.85 – 7.77 (m, 5H, Ar-H), 7.77 – 7.71 (m, 3H, Ar-H), 7.62 – 7.27 (m, 28H, NHCO, Ar-H), 7.26 – 7.07 (m, 8H, Ar-H), 5.98 – 5.82 (m, 1H, $CH_2CH=CH_2$), 5.40 (d, *J* = 3.9 Hz, 1H, H-1A), 5.33 – 5.26 (m, 1H, $CH_2CH=CH_2$), 5.19 (dq, *J* = 10.5, 1.4 Hz, 1H, $CH_2CH=CH_2$), 5.08 (d, *J* = 3.6 Hz, 1H, H-1C), 5.00 (s, 1H, H-2D), 4.94 (d, *J* = 11.0 Hz, 1H, $ArCH_2$), 4.91 – 4.74 (m, 5H, H-1D, $ArCH_2$), 4.57 – 4.47 (m, 4H, H-2B, $ArCH_2$), 4.46 – 4.26 (m, 8H, H-2C, $ArCH_2$), 4.23 – 4.12 (m, 5H, $CH_2CH=CH_2$), 4.08 (d, *J* = 8.0 Hz, 1H, H-1B), 4.01 – 3.93 (m, 1H, H-4A), 3.93 – 3.88 (m, 2H, $CH_2CH=CH_2$), 3.82 (d, *J* = 9.9 Hz, 1H, H-5A), 3.79 – 3.56 (m, 9H, H-3A, H-6aA, H-4B, H-3C, H-3D, H-4D), 3.55 – 3.43 (m, 2H, H-6aB), 3.41 (d, *J* = 10.9 Hz, 1H, H-6bA), 3.28 – 3.17 (m, 2H, H-2A, H-6bB), 2.97 (dd, *J* = 9.4, 3.5 Hz, 1H, H-5B), 2.87 (t, *J* = 9.2 Hz, 1H, H-3B), 2.84 – 2.76 (m, 1H, $COCH_2$), 2.75 (brs, 1H, OH), 2.63 (dt, *J* = 18.7, 5.1 Hz, 1H, $COCH_2$), 2.44 – 2.38 (m, 2H, $COCH_2$), 2.06 (s, 3H, CH_3CO); ^{13}C NMR (125 MHz, $CDCl_3$) δ 207.33, 171.96, 157.31 (q, *J* = 37.1 Hz), 155.92, 154.21, 143.33, 143.31, 143.22, 143.16, 141.45, 141.27, 138.17, 137.86, 137.79, 137.43, 134.33, 133.67, 133.20, 133.00, 128.69, 128.50, 128.48,

128.40, 128.30, 128.24, 128.19, 128.13, 128.07, 127.92, 127.89, 127.69, 127.67, 127.52, 127.43, 127.20, 127.03, 126.69, 126.57, 126.33, 126.16, 125.20, 125.11, 124.66, 124.60, 120.27, 120.22, 120.07, 120.05, 117.40, 115.80 (q, J = 286.4 Hz), 100.03, 97.44, 97.24, 97.14, 82.74, 79.32, 78.35, 77.02, 77.00, 75.36, 74.36, 74.27, 74.22, 73.72, 73.03, 72.67, 72.16, 71.64, 70.60, 70.25, 70.20, 69.34, 68.39, 68.17, 66.83, 66.60, 66.01, 62.77, 61.39, 61.27, 53.23, 46.73, 46.64, 38.01, 29.44, 27.81; HRMS (ESI) Anal. Calcd for $C_{103}H_{103}F_3N_4O_{26}$ $[M+NH_4]^+$: 1886.7151, found 1886.7112.

Allyl 2-azido-3-*O*-benzyl-2-deoxy-6-*O*-fluorenylmethoxycarbonyl-*α*-D-glucopyranosyl-(1→4)-methyl-3-*O*-benzyl-2-*O*-fluorenylmethoxycarbonyl-*β*-D-glucopyranosyluronate-(1→4)-3-*O*-benzyl-2-deoxy-6-*O*-(2-naphthylmethyl)-2-trifluoroacetamido-*α*-D-glucopyranosyl-(1→4)-methyl-3-*O*-benzyl-2-*O*-levulinyl-*α*-L-idopyranosyluronate (S11): Compound **S11** was prepared from compound **S10** (1.05 g, 0.56 mmol) using the general procedure for oxidation of 6-OH and methyl ester formation after oxidation, followed by silica gel chromatography (3:2 hexanes: EtOAc) to yield 90% of compound **S11**. $[\alpha]_D^{20}$ -20.2° (c 1.0, DCM); ^{1}H NMR (500 MHz, $CDCl_3$) δ 8.05 (d, J = 8.4 Hz, 1H, NHCO), 7.89 – 7.83 (m, 4H, Ar-H), 7.75 (ddt, J = 7.6, 1.7, 0.8 Hz, 2H, Ar-H), 7.69 – 7.65 (m, 1H, Ar-H), 7.63 – 7.27 (m, 27H, Ar-H), 7.26 – 7.02 (m, 9H, Ar-H), 5.90 – 5.76 (m, 1H, $CH_2CH{=}CH_2$), 5.29 – 5.19 (m, 2H, $CH_2CH{=}CH_2$, H-1A), 5.15 (d, J = 3.7 Hz, 1H, H-1C), 5.12 (dq, J = 10.4, 1.4 Hz, 1H, $CH_2CH{=}CH_2$), 5.01 – 4.88 (m, 7H, H-1D, H-2D, $ArCH_2$), 4.84 – 4.79 (m, 2H, H-5D, $ArCH_2$), 4.60 – 4.50 (m, 4H, H-6aA, H-2B, $ArCH_2$), 4.49 – 4.34 (m, 4H, $ArCH_2$, $CH_2CH(C_6H_4)_2$), 4.28 – 4.15 (m, 7H, $CH_2CH{=}CH_2$, H-6bA, H-1B, H-2C, $CH_2CH(C_6H_4)_2$, $CH_2CH(C_6H_4)_2$), 4.12 – 4.07 (m, 2H, H-4D, $CH_2CH(C_6H_4)_2$), 4.00 (dd, J = 9.9, 8.9 Hz, 1H, H-4C), 3.95 – 3.87 (m, 2H, $CH_2CH{=}CH_2$, H-4B), 3.86 – 3.65 (m, 8H, H-3A, H-3C, H-5C, H-6aC, H-3D, OCH_3), 3.64 – 3.49 (m, 3H, H-4A, H-5B, H-6bC), 3.49 – 3.39 (m, 4H, H-5A, OCH_3), 3.30 (dd, J = 10.2, 3.7 Hz, 1H, H-2A), 3.02 – 2.91 (m, 1H, $COCH_2$), 2.77 (brs, 1H, OH-4A), 2.67 (t, J = 9.2 Hz, 1H, H-3B), 2.54 (td, J = 11.8, 11.1, 4.9 Hz, 2H, $COCH_2$), 2.38 – 2.29 (m, 1H, $COCH_2$), 1.92 (s, 3H, CH_3CO); ^{13}C NMR (125 MHz, $CDCl_3$) δ 208.77, 172.27, 169.22, 167.76, 157.43 (q, J = 37.3 Hz), 155.97, 153.91, 143.40, 143.28, 143.18, 143.12, 141.47, 141.39, 141.28, 138.70, 137.83, 137.71, 137.44, 134.74, 133.38, 133.26, 132.97, 128.68, 128.45, 128.33, 128.31, 128.26, 128.21, 128.19, 128.17, 128.05, 128.01, 127.95, 127.92, 127.82, 127.71, 127.69, 127.59, 127.45, 127.28, 127.20, 127.17, 127.08, 126.98, 126.94, 126.35, 126.15, 125.14, 125.08, 124.94, 124.59, 120.21, 120.06, 120.05, 117.33, 115.90 (q, J = 286.9 Hz), 100.10, 98.23, 97.71, 97.47, 81.51, 79.29, 77.52, 77.49, 75.40, 75.34, 74.41, 73.99, 73.91, 73.65, 73.03, 72.05, 71.09, 70.60, 70.21, 70.05, 69.25, 68.58, 67.13, 66.68, 66.27, 65.69, 63.00, 53.53, 52.53, 52.28, 46.78, 46.63, 38.37, 29.12, 27.95; HRMS (ESI) Anal. Calcd for $C_{105}H_{103}F_3N_4O_{28}$ $[M+NH_4]^+$: 1942.7049, found 1942.7074.

Allyl 4-*O*-acetyl-2-azido-3-*O*-benzyl-2-deoxy-6-*O*-fluorenylmethoxycarbonyl-*α*-D-glucopyranosyl-(1→4)-methyl-3-*O*-benzyl-2-*O*-fluorenylmethoxycarbonyl-*β*-D-glucopyranosyluronate-(1→4)-3-*O*-benzyl-2-deoxy-6-*O*-(2-naphthylmethyl)-2-trifluoroacetamido-*α*-D-glucopyranosyl-(1→4)-methyl-3-*O*-benzyl-2-*O*-levulinyl-*α*-L-idopyranosyluronate (21): Compound **21** was prepared from compound **S11** (790 mg, 0.41

mmol) using the general procedure for acetylation, followed by silica gel chromatography (2:1 hexanes: EtOAc) to yield 99% of compound **21**. $[\alpha]_D^{20}$ -10.1° (*c* 1.0, DCM); ^{1}H NMR (500 MHz, $CDCl_3$) δ 8.07 (d, *J* = 8.4 Hz, 1H, NHCO), 7.90 – 7.83 (m, 4H, Ar-H), 7.74 (d, *J* = 7.6 Hz, 2H, Ar-H), 7.67 (d, *J* = 7.2 Hz, 1H, Ar-H), 7.63 – 7.27 (m, 26H, Ar-H), 7.26 – 7.01 (m, 10H, Ar-H), 5.89 – 5.76 (m, 1H, CH_2CH=CH_2), 5.30 (d, *J* = 4.0 Hz, 1H, H-1A), 5.24 (dt, *J* = 17.2, 1.8 Hz, 1H, CH_2CH=CH_2), 5.18 – 5.06 (m, 3H, CH_2CH=CH_2, H-4A, H-1C), 4.98 (d, *J* = 11.9 Hz, 1H, $ArCH_2$), 4.95 – 4.89 (m, 3H, H-1D, H-2D, $ArCH_2$), 4.88 – 4.79 (m, 3H, $ArCH_2$), 4.71 (d, *J* = 11.0 Hz, 1H, $ArCH_2$), 4.62 – 4.51 (m, 3H, H-2B, $ArCH_2$), 4.51 – 4.29 (m, 4H, H-3C, H-3D, $ArCH_2$), 4.27 – 4.16 (m, 7H, CH_2CH=CH_2, H-6aA, H-1B, H-2C, $ArCH_2$), 4.13 – 4.06 (m, 3H, H-6bA), 4.00 (t, *J* = 9.4 Hz, 1H), 3.96 – 3.73 (m, 10H, CH_2CH=CH_2, H-3A, H-4B, OCH_3), 3.72 – 3.57 (m, 4H, H-5A, H-5B), 3.43 (ddd, *J* = 10.3, 3.7, 1.7 Hz, 1H, H-2A), 3.38 (s, 3H, OCH_3), 3.01 – 2.89 (m, 1H, $COCH_2$), 2.68 (t, *J* = 9.1 Hz, 1H, H-3B), 2.58 – 2.47 (m, 2H, $COCH_2$), 2.38 – 2.30 (m, 1H, $COCH_2$), 1.97 (s, 3H, CH_3CO), 1.89 (s, 3H, CH_3CO); ^{13}C NMR (125 MHz, $CDCl_3$) δ 208.81, 172.28, 169.42, 169.24, 167.80, 157.47 (q, *J* = 37.3 Hz), 154.90, 153.91, 143.47, 143.42, 143.30, 143.27, 141.51, 141.44, 141.27, 141.24, 138.80, 137.68, 137.46, 137.39, 134.80, 133.41, 133.31, 132.98, 128.61, 128.46, 128.43, 128.34, 128.29, 128.19, 128.16, 128.09, 128.05, 127.93, 127.86, 127.84, 127.72, 127.63, 127.53, 127.31, 127.23, 127.18, 127.16, 126.90, 126.81, 126.33, 126.11, 125.27, 125.21, 124.94, 124.57, 120.24, 119.98, 117.37, 115.92 (q, *J* = 285.6 Hz), 100.09, 98.27, 97.51, 97.36, 81.52, 77.62, 77.58, 77.49, 75.43, 75.02, 74.45, 74.33, 74.04, 73.96, 73.67, 73.03, 72.08, 71.10, 70.10, 70.04, 69.24, 68.61, 68.43, 67.14, 66.69, 66.28, 65.38, 65.35, 62.99, 53.57, 52.52, 52.29, 46.81, 46.63, 38.38, 29.09, 27.96, 20.81; HRMS (ESI) Anal. Calcd for $C_{107}H_{105}F_3N_4O_{29}$ $[M+NH_4]^+$: 1984.7155, found 1984.7102.

Allyl 4-*O*-acetyl-2-azido-3-*O*-benzyl-2-deoxy-*α*-D-glucopyranosyl-(1→4)-methyl-3-*O*-benzyl-*β*-D-glucopyranosyluronate-(1→4)-3-*O*-benzyl-6-*O*-(2-naphthylmethyl)-2-deoxy-2-trifluoroacetamido-*α*-D-glucopyranosyl-(1→4)-methyl-3-*O*-benzyl-2-*O*-levulinyl-*α*-L-idopyranosyluronate (S12): Compound **S12** was prepared from compound **21** (796 mg, 0.40 mmol) using the general procedure for Fmoc removal, followed by silica gel chromatography (1:1 hexanes: EtOAc) to yield 96% of compound **S12**. $[\alpha]_D^{20}$ +4.5° (*c* 1.0, DCM); ^{1}H NMR (500 MHz, $CDCl_3$) δ 8.49 (d, *J* = 8.0 Hz, 1H, NHCO), 7.94 – 7.84 (m, 4H, Ar-H), 7.58 – 7.46 (m, 3H, Ar-H), 7.40 – 7.27 (m, 15H, Ar-H), 7.26 – 7.24 (m, 2H, Ar-H), 7.23 – 7.14 (m, 3H, Ar-H), 5.99 – 5.83 (m, 1H, CH_2CH=CH_2), 5.52 (d, *J* = 3.8 Hz, 1H, H-1A), 5.31 (dt, *J* = 17.2, 1.4 Hz, 1H, CH_2CH=CH_2), 5.19 (dt, *J* = 10.5, 1.4 Hz, 1H, CH_2CH=CH_2), 5.08 (d, *J* = 3.6 Hz, 1H, H-1C), 5.00 (s, 1H, H-1D), 4.96 (d, *J* = 11.4 Hz, 1H, $ArCH_2$), 4.93 – 4.77 (m, 7H, H-4A, H-2D, H-5D, $ArCH_2$), 4.74 – 4.66 (m, 3H, $ArCH_2$), 4.62 – 4.52 (m, 2H, $ArCH_2$), 4.48 (d, *J* = 7.8 Hz, 1H, H-1B), 4.31 – 4.18 (m, 2H, CH_2CH=CH_2, H-2C), 4.17 – 4.02 (m, 5H, CH_2CH=CH_2, H-4C, H-4D, H-6C), 3.97 (t, *J* = 9.2 Hz, 1H, H-4B), 3.93 – 3.82 (m, 3H, H-3A, H-3C, H-5C), 3.77 (dd, *J* = 9.8, 1.0 Hz, 1H, H-5B), 3.72 (d, *J* = 2.4 Hz, 1H, H-3D), 3.67 (s, 3H, OCH_3), 3.57 (d, *J* = 12.4 Hz, 1H, H-6aA), 3.49 (t, *J* = 8.4 Hz, 1H, H-2B), 3.46 – 3.33 (m, 6H, H-2A, H-5A, H-6bA, OCH_3), 3.29 (t, *J* = 8.8 Hz, 1H, H-3B), 3.03 (ddd, *J* = 18.7, 12.0, 3.4 Hz, 1H, $COCH_2$), 2.85 (brs, 1H, OH), 2.63 – 2.42 (m,

3H, $COCH_2$, OH), 2.35 (dt, *J* = 17.3, 4.3 Hz, 1H, $COCH_2$), 1.99 (s, 3H, CH_3CO), 1.92 (s, 3H, CH_3CO); ^{13}C NMR (125 MHz, cdcl$_3$) δ 209.43, 172.11, 170.85, 169.35, 168.43, 157.62 (q, *J* = 37.3 Hz), 138.64, 138.36, 137.56, 137.44, 134.99, 133.40, 133.28, 133.07, 128.54, 128.47, 128.40, 128.32, 128.22, 128.10, 128.02, 127.83, 127.82, 127.78, 127.66, 127.61, 127.43, 127.25, 126.68, 126.44, 126.31, 126.10, 117.42, 115.95 (q, *J* = 286.4 Hz), 102.56, 99.21, 98.27, 97.55, 83.61, 77.77, 77.38, 77.23, 76.43, 76.42, 75.33, 75.29, 75.01, 74.77, 74.48, 73.87, 73.84, 73.35, 72.07, 71.22, 70.86, 70.55, 68.62, 67.61, 67.22, 66.30, 63.00, 60.77, 53.96, 52.43, 52.13, 38.43, 29.00, 28.18, 20.79; HRMS (ESI) Anal. Calcd for $C_{77}H_{85}F_3N_4O_{25}$ $[M+NH_4]^+$: 1540.5793, found 1540.5747.

Allyl 4-*O*-acetyl-2-azido-3-*O*-benzyl-6-*O*-tert-butyldiphenylsilyl-2-deoxy-*α*-D-glucopyranosyl-(1→4)-methyl-3-*O*-benzyl-*β*-D-glucopyranosyluronate-(1→4)-3-*O*-benzyl-2-deoxy-6-*O*-(2-naphthylmethyl)-2-trifluoroacetamido-*α*-D-glucopyranosyl-(1→4)-methyl-3-*O*-benzyl-2-*O*-levulinyl-*α*-L-idopyranosyluronate (S13): Compound **S13** was prepared from compound **S12** (539 mg, 0.35 mmol) using the general procedure for TBDPS ether formation, followed by silica gel chromatography (2:1 hexanes: EtOAc) to yield 94% of compound **S13**. $[\alpha]_D^{20}$ +17.1° (*c* 1.0, DCM); ^1H NMR (500 MHz, $CDCl_3$) δ 8.44 (d, *J* = 8.0 Hz, 1H, NHCO), 7.91 – 7.83 (m, 5H, Ar-H), 7.66 – 7.60 (m, 4H, Ar-H), 7.54 (dd, *J* = 8.4, 1.6 Hz, 1H, Ar-H), 7.51 – 7.44 (m, 2H, Ar-H), 7.41 – 7.27 (m, 19H, Ar-H), 7.25 – 7.10 (m, 6H, Ar-H), 5.96 – 5.80 (m, 1H, $CH_2CH{=}CH_2$), 5.47 (d, *J* = 3.6 Hz, 1H, H-1A), 5.34 – 5.26 (m, 2H, $CH_2CH{=}CH_2$, H-4A), 5.18 (dq, *J* = 10.5, 1.5 Hz, 1H, $CH_2CH{=}CH_2$), 5.06 (d, *J* = 3.7 Hz, 1H, H-1C), 4.99 (s, 1H, H-1D), 4.96 – 4.86 (m, 3H, H-2D, $ArCH_2$), 4.84 – 4.76 (m, 3H, H-5D, $ArCH_2$), 4.72 – 4.66 (m, 2H, $ArCH_2$), 4.64 – 4.51 (m, 3H, $ArCH_2$), 4.45 (d, *J* = 7.8 Hz, 1H, H-1B), 4.26 (ddt, *J* = 13.1, 4.9, 1.6 Hz, 1H, $CH_2CH{=}CH_2$), 4.20 (ddd, *J* = 11.3, 8.0, 3.6 Hz, 1H, H-2C), 4.16 – 4.00 (m, 6H, $CH_2CH{=}CH_2$, H-4C, H-6C, H-4D, $ArCH_2$), 3.93 (dd, *J* = 9.7, 8.7 Hz, 1H, H-4B), 3.88 – 3.79 (m, 3H, H-3A, H-3C, H-5C), 3.75 (d, *J* = 9.7 Hz, 1H, H-5B), 3.71 (dt, *J* = 2.8, 1.4 Hz, 1H, H-3D), 3.65 (s, 3H, OCH_3), 3.61 (dd, *J* = 11.6, 2.2 Hz, 1H, H-6aA), 3.57 – 3.53 (m, 1H, H-6bA), 3.50 – 3.42 (m, 2H, H-5A, H-2B), 3.37 (dd, *J* = 10.3, 3.7 Hz, 1H, H-2A), 3.27 (t, *J* = 8.9 Hz, 1H, H-3B), 3.21 (s, 3H, OCH_3), 3.00 (ddd, *J* = 18.8, 12.4, 3.5 Hz, 1H, $COCH_2$), 2.85 (brs, 1H, OH), 2.62 – 2.49 (m, 2H, $COCH_2$), 2.36 – 2.28 (m, 1H, $COCH_2$), 1.91 (s, 3H, CH_3CO), 1.87 (s, 3H, CH_3CO), 1.01 (s, 9H, $C(CH_3)_3$); ^{13}C NMR (125 MHz, $CDCl_3$) δ 209.39, 172.12, 169.31, 168.98, 168.23, 157.57 (q, *J* = 37.3 Hz), 138.64, 138.43, 137.48, 137.44, 135.77, 135.72, 134.93, 133.40, 133.27, 133.20, 133.13, 133.08, 129.61, 129.52, 128.52, 128.48, 128.42, 128.31, 128.23, 128.18, 128.08, 128.00, 127.82, 127.65, 127.62, 127.56, 127.49, 127.46, 127.18, 126.67, 126.48, 126.31, 126.11, 117.41, 115.94 (q, *J* = 286.4 Hz), 102.58, 99.21, 98.27, 97.31, 83.60, 77.83, 77.68, 76.44, 75.16, 74.57, 74.49, 74.33, 73.90, 73.87, 73.30, 72.06, 71.15, 70.78, 69.50, 68.60, 67.62, 67.23, 66.29, 63.02, 61.66, 53.96, 52.12, 38.42, 29.01, 28.17, 26.73, 20.85, 19.22; HRMS (ESI) Anal. Calcd for $C_{93}H_{103}F_3N_4O_{25}Si$ $[M+NH_4]^+$: 1778.6971, found 1778.6986.

Allyl 4-*O*-acetyl-2-azido-3-*O*-benzyl-6-*O*-tert-butyldiphenylsilyl-2-deoxy-*α*-D-glucopyranosyl-(1→4)-methyl-3-*O*-benzyl-2-*O*-fluorenylmethoxycarbonyl-*β*-D-

glucopyranosyluronate-(1→4)-3-*O*-benzyl-2-deoxy-6-*O*-(2-naphthylmethyl)-2-trifluoroacetamido-*α*-D-glucopyranosyl-(1→4)-methyl-3-*O*-benzyl-2-*O*-levulinyl-*α*-L-idopyranosyluronate (22): Compound **22** was prepared from compound **S13** (588 mg, 0.33 mmol) using the general procedure for Fmoc carbonate formation, followed by silica gel chromatography (4:1 hexanes: EtOAc) to yield 95% of compound **22**. $[\alpha]_D^{20}$ -0.3º (*c* 1.0, DCM); ^{1}H NMR (500 MHz, $CDCl_3$) δ 7.98 (d, *J* = 8.4 Hz, 1H, NHCO), 7.89 – 7.83 (m, 4H, Ar-H), 7.68 (d, *J* = 7.2 Hz, 1H, Ar-H), 7.65 – 7.58 (m, 5H, Ar-H), 7.55 – 7.28 (m, 25H, Ar-H), 7.26 – 7.09 (m, 8H, Ar-H), 7.08 – 7.04 (m, 2H, Ar-H), 5.87 – 5.76 (m, 1H, $CH_2CH{=}CH_2$), 5.32 (t, *J* = 9.7 Hz, 1H, H-4A), 5.27 – 5.21 (m, 2H, $CH_2CH{=}CH_2$, H-1A), 5.15 (d, *J* = 3.7 Hz, 1H, H-1C), 5.12 (dq, *J* = 10.5, 1.6 Hz, 1H, $CH_2CH{=}CH_2$), 4.99 (d, *J* = 12.0 Hz, 1H, $ArCH_2$), 4.95 – 4.89 (m, 3H, H-1D, H-2D, $ArCH_2$), 4.87 – 4.79 (m, 3H, H-5D, $ArCH_2$), 4.67 (d, *J* = 11.0 Hz, 1H, $ArCH_2$), 4.63 – 4.50 (m, 3H, H-2B, $ArCH_2$, $CH_2CH(C_6H_4)_2$), 4.46 (dd, *J* = 10.5, 6.0 Hz, 1H, $CH_2CH(C_6H_4)_2$), 4.37 (d, *J* = 12.2 Hz, 1H, $ArCH_2$), 4.25 – 4.13 (m, 5H, $CH_2CH{=}CH_2$, H-1B, H-2C, $ArCH_2$, $CH_2CH(C_6H_4)_2$), 4.13 – 4.05 (m, 2H, H-4D, $ArCH_2$), 3.97 (t, *J* = 9.4 Hz, 1H, H-4C), 3.94 – 3.72 (m, 11H, $CH_2CH{=}CH_2$, H-3A, H-4B, H-3D, H-5C, H-6C, $ArCH_2$, OCH_3), 3.68 (dd, *J* = 10.8, 8.8 Hz, 1H, H-3C), 3.63 – 3.58 (m, 2H, H-6aA, H-5B), 3.55 (dd, *J* = 11.6, 3.1 Hz, 1H, H-6bA), 3.46 (dt, *J* = 10.1, 2.7 Hz, 1H, H-5A), 3.40 (dd, *J* = 10.3, 3.6 Hz, 1H, H-2A), 3.16 (s, 3H, OCH_3), 2.97 (ddd, *J* = 18.3, 11.5, 3.7 Hz, 1H, $COCH_2$), 2.66 (t, *J* = 9.1 Hz, 1H, H-3B), 2.60 – 2.50 (m, 2H, $COCH_2$), 2.35 (dt, *J* = 17.7, 4.4 Hz, 1H, $COCH_2$), 1.93 – 1.88 (m, 6H, CH_3CO), 1.01 (s, 9H, $C(CH_3)_3$); ^{13}C NMR (125 MHz, $CDCl_3$) δ 208.71, 172.26, 169.20, 169.01, 167.68, 157.42 (q, *J* = 37.0 Hz), 153.93, 143.42, 143.32, 141.50, 141.43, 138.84, 137.75, 137.45, 135.76, 135.73, 134.69, 133.39, 133.28, 133.14, 133.08, 132.99, 129.66, 129.56, 128.58, 128.50, 128.39, 128.33, 128.28, 128.27, 128.13, 128.09, 128.03, 127.92, 127.83, 127.70, 127.64, 127.58, 127.47, 127.30, 127.16, 127.05, 126.86, 126.81, 126.32, 126.15, 124.95, 124.58, 120.24, 120.22, 117.35, 115.90 (q, *J* = 286.6 Hz), 100.00, 98.25, 97.48, 97.26, 81.52, 77.86, 77.67, 77.39, 74.76, 74.63, 74.43, 74.24, 73.94, 73.69, 73.05, 72.07, 71.09, 70.91, 69.57, 69.21, 68.60, 67.17, 66.70, 66.18, 63.13, 61.62, 53.54, 52.28, 52.19, 46.79, 38.38, 29.10, 27.96, 26.74, 20.88, 19.21; HRMS (ESI) Anal. Calcd for $C_{108}H_{113}F_3N_4O_{27}Si$ $[M+NH_4]^+$: 2000.7652, found 2000.7639.

Methylcarboxy 4-*O*-acetyl-2-azido-3-*O*-benzyl-6-*O*-tert-butyldiphenylsilyl-2-deoxy-*α*-D-glucopyranosyl-(1→4)-methyl-3-*O*-benzyl-2-*O*-fluorenylmethoxycarbonyl-*β*-D-glucopyranosyluronate-(1→4)-3-*O*-benzyl-2-deoxy-6-*O*-(2-naphthylmethyl)-2-trifluoroacetamido-*α*-D-glucopyranosyl-(1→4)-methyl-3-*O*-benzyl-2-*O*-levulinyl-*α*-L-idopyranosyluronate (23): Compound **23** was prepared from compound **22** (631 mg, 0.32 mmol) using the general procedure for oxidation of allyl group to carboxyl group, followed by silica gel chromatography (30:1 DCM: MeOH) to yield 90% of compound **23**. $[\alpha]_D^{20}$ +2.6º (*c* 1.0, DCM); ^{1}H NMR (500 MHz, $CDCl_3$) δ 8.05 (d, *J* = 8.3 Hz, 1H, NHCO), 7.90 – 7.83 (m, 4H, Ar-H), 7.69 – 7.57 (m, 6H, Ar-H), 7.56 – 7.28 (m, 25H, Ar-H), 7.26 – 7.11 (m, 8H, Ar-H), 7.11 – 7.06 (m, 2H, Ar-H), 5.34 (t, *J* = 9.7 Hz, 1H, H-4A), 5.27 (d, *J* = 3.6 Hz, 1H, H-1A), 5.16 (d, *J* = 3.7 Hz, 1H, H-1C), 5.04 – 4.91 (m, 4H, H-1D, H-2D, $ArCH_2$), 4.89 – 4.79 (m, 3H, H-5D, $ArCH_2$), 4.69 (d, *J* =

11.0 Hz, 1H, $ArCH_2$), 4.63 – 4.50 (m, 3H, H-2B, $ArCH_2$, $CH_2CH(C_6H_4)_2$), 4.44 (dd, J = 10.6, 5.9 Hz, 1H, $CH_2CH(C_6H_4)_2$), 4.37 (d, J = 12.2 Hz, 1H, $ArCH_2$), 4.27 – 4.07 (m, 8H, H-1B, H-2C, H-4D, $ArCH_2$, $CH_2CH(C_6H_4)_2$, OCH_2COOH), 3.99 (t, J = 9.4 Hz, 1H, H-4C), 3.93 – 3.73 (m, 9H, H-3A, H-4B, H-5C, H-6aC, H-3D, OCH_3, OCH_2COOH), 3.69 (dd, J = 10.7, 8.8 Hz, 1H, H-3C), 3.65 – 3.54 (m, 4H, H-6A, H-5B,H-6bC), 3.47 (dt, J = 10.1, 2.7 Hz, 1H, H-5A), 3.42 (dd, J = 10.3, 3.6 Hz, 1H, H-2A), 3.18 (s, 3H, OCH_3), 2.98 (ddd, J = 19.3, 12.1, 3.8 Hz, 1H, $COCH_2$), 2.68 (t, J = 9.1 Hz, 1H, H-3B), 2.60 – 2.50 (m, 2H, $COCH_2$), 2.36 (dt, J = 16.4, 4.1 Hz, 1H, $COCH_2$), 1.92 (s, 3H, CH_3CO), 1.91 (s, 3H, CH_3CO), 1.03 (s, 9H, $C(CH_3)_3$); ^{13}C NMR (125 MHz, $CDCl_3$) δ 208.93, 173.05, 172.16, 169.07, 168.81, 167.67, 157.44 (q, J = 37.0 Hz), 153.94, 143.40, 143.29, 141.44, 141.41, 138.78, 137.74, 137.43, 137.13, 135.76, 135.73, 134.63, 133.27, 133.14, 133.09, 133.00, 129.66, 129.56, 128.58, 128.51, 128.48, 128.43, 128.39, 128.28, 128.16, 128.12, 128.10, 128.06, 128.01, 127.91, 127.79, 127.64, 127.58, 127.49, 127.33, 127.13, 127.10, 126.86, 126.82, 126.33, 126.17, 124.86, 124.56, 120.28, 115.88 (q, J = 286.4 Hz), 99.99, 99.02, 97.55, 97.27, 81.56, 77.84, 77.52, 77.40, 77.03, 74.75, 74.64, 74.24, 74.13, 73.99, 73.92, 73.68, 72.96, 72.43, 71.18, 70.92, 69.59, 69.23, 67.21, 67.08, 66.14, 64.13, 63.12, 61.62, 53.52, 52.38, 52.20, 46.77, 38.39, 29.06, 27.93, 26.74, 20.88, 19.21; HRMS (ESI) Anal. Calcd for $C_{107}H_{111}F_3N_4O_{29}Si$ $[M+NH_4]^+$: 2018.7394, found 2018.7390.

Scheme S4. Syntheses of tetrasaccharides **25-28**.

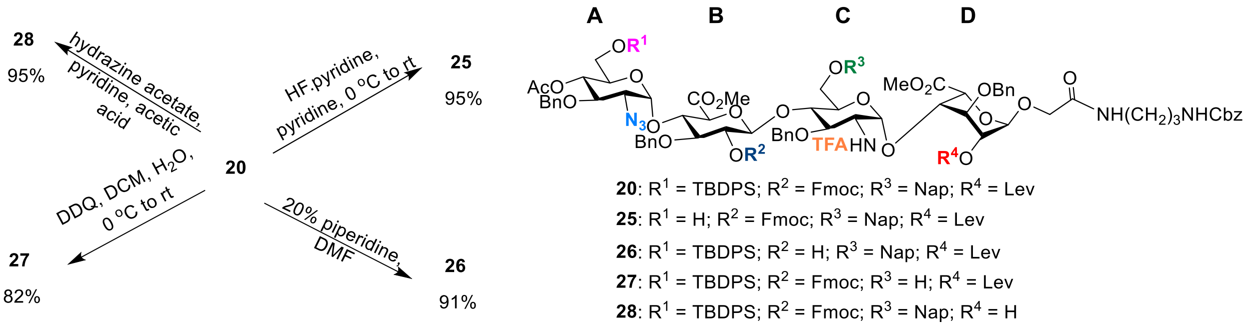

Compound 20: Compound **20** was prepared from compound **23** (6.9 g, 3.46 mmol) using the general procedure for amidation followed by Fmoc-protection (since some of the Fmoc-group fall off during amidation). The desired product was purified by silica gel chromatography (10:1 hexanes: ethyl acetate with 10% MeOH) to afford compound **20** (6.92 g, 3.13 mmol) with 90% yield. $[\boldsymbol{\alpha}]_{\mathbf{D}}^{\mathbf{20}}$ +4.0° (c 1.0, $CHCl_3$); ^1H NMR (500 MHz, $CDCl_3$) δ 8.09 (d, J = 8.0 Hz, 1H), 7.86 (dd, J = 11.2, 6.9 Hz, 3H), 7.62 (t, J = 7.3 Hz, 6H), 7.58 – 7.27 (m, 29H), 7.26 – 7.14 (m, 7H), 7.12 (d, J = 7.8 Hz, 2H), 7.08 (d, J = 7.0 Hz, 2H), 6.66 – 6.61 (m, 1H), 5.31 (t, J = 9.7 Hz, 1H), 5.27 – 5.18 (m, 3H), 5.06 (s, 2H), 4.94 (dd, J = 20.9, 12.0 Hz, 3H), 4.86 – 4.79 (m, 2H), 4.73 (d, J = 11.1 Hz, 1H), 4.67 (d, J = 11.0 Hz, 1H), 4.61 – 4.49 (m, 4H), 4.38 (dd, J = 11.4, 4.8 Hz, 2H), 4.26 – 4.09 (m, 8H), 4.00 – 3.95 (m, 1H), 3.90 – 3.73 (m, 10H), 3.68 – 3.52 (m, 6H), 3.45 (d, J = 10.0 Hz, 1H), 3.40 (dd, J = 10.3, 3.5 Hz, 1H), 3.16 (s, 3H), 3.04 – 2.89 (m, 5H), 2.69 (t, J = 9.2 Hz, 1H), 2.62 – 2.46 (m, 3H), 2.34 (dd, J = 14.1, 5.6 Hz, 1H), 1.92 (s, 3H), 1.90 (s, 3H), 1.00 (s,

9H); ^{13}C NMR (125 MHz, $CDCl_3$) δ 208.80, 172.23, 168.99, 168.90, 168.53, 167.63, 157.59, 157.30, 156.53, 153.94, 143.39, 143.21, 141.40, 138.68, 137.71, 137.40, 136.98, 136.63, 135.73, 135.70, 134.55, 133.26, 133.10, 133.05, 133.00, 129.63, 129.53, 128.62, 128.55, 128.47, 128.34, 128.29, 128.26, 128.16, 128.11, 128.08, 128.02, 127.90, 127.79, 127.61, 127.55, 127.49, 127.44, 127.31, 127.15, 127.08, 126.92, 126.83, 126.37, 126.20, 124.80, 124.56, 120.31, 114.71, 99.97, 97.87, 97.47, 97.28, 81.59, 77.81, 77.35, 77.23, 76.92, 74.72, 74.63, 74.23, 74.04, 73.83, 73.69, 73.39, 72.93, 71.15, 70.91, 69.53, 69.38, 67.17, 66.98, 66.53, 66.06, 65.77, 63.09, 61.59, 53.51, 52.52, 52.19, 46.74, 38.30, 37.43, 35.34, 29.77, 29.11, 27.86, 26.71, 20.85, 19.18; HRMS (ESI) Anal. Calcd for $C_{118}H_{129}F_3N_7O_{30}Si^+$ $[M+NH_4]^+$: 2208.8500, found 2208.8259.

Compound **25:** The solution of compound **20** (110 mg, 50 µmol) in pyridine (2.0 mL) was treated with HF-pyridine (1.0 mL) for the deprotection of TBDPS-group according to general procedure to afford compound **25** (96 mg, 91% yield). $[\boldsymbol{\alpha}]_{\mathbf{D}}^{\mathbf{20}}$ -13.3º (*c* 1.0, $CHCl_3$); ^1H NMR (500 MHz, $CDCl_3$) δ 8.16 (d, *J* = 8.0 Hz, 1H), 7.89 – 7.81 (m, 4H), 7.65 – 7.57 (m, 3H), 7.55 – 7.27 (m, 24H), 7.26 – 7.12 (m, 8H), 7.05 (d, *J* = 6.3 Hz, 2H), 6.62 (t, *J* = 6.2 Hz, 1H), 5.28 (d, *J* = 3.8 Hz, 1H), 5.23 – 5.18 (m, 2H), 5.06 (s, 2H), 4.98 (d, *J* = 11.9 Hz, 1H), 4.96 – 4.84 (m, 4H), 4.79 (s, 1H), 4.73 (d, *J* = 11.2 Hz, 2H), 4.61 – 4.50 (m, 4H), 4.43 – 4.35 (m, 2H), 4.25 (d, *J* = 11.1 Hz, 1H), 4.24 – 4.15 (m, 4H), 4.14 – 4.07 (m, 2H), 4.03 – 3.96 (m, 1H), 3.93 – 3.84 (m, 2H), 3.84 – 3.72 (m, 8H), 3.70 – 3.64 (m, 1H), 3.64 -3.59 (m, 2H), 3.58 – 3.52 (m, 1H), 3.46 – 3.34 (m, 6H), 3.05 – 2.90 (m, 5H), 2.70 (t, *J* = 9.1 Hz, 1H), 2.61 – 2.47 (m, 3H), 2.37 – 2.29 (m, 1H), 2.00 (s, 3H), 1.90 (s, 3H); ^{13}C NMR (125 MHz, $CDCl_3$) δ 208.99, 172.21, 170.78, 168.89, 168.49, 167.84, 156.53, 153.92, 143.40, 143.15, 141.40, 141.38, 138.68, 137.61, 137.47, 136.97, 136.62, 134.63, 133.27, 132.97, 128.62, 128.58, 128.49, 128.46, 128.37, 128.29, 128.25, 128.20, 128.10, 128.02, 127.87, 127.82, 127.80, 127.52, 127.45, 127.31, 127.20, 127.06, 126.86, 126.77, 126.35, 126.14, 124.82, 124.55, 120.31, 99.95, 97.86, 97.79, 97.42, 81.59, 77.50, 77.47, 77.22, 76.84, 75.52, 75.10, 74.49, 74.15, 74.04, 73.80, 73.69, 72.93, 71.17, 70.82, 70.77, 69.44, 67.04, 66.53, 65.70, 63.05, 53.61, 52.50, 46.75, 38.33, 37.42, 35.32, 29.78, 29.70, 29.08, 27.89, 20.80; HRMS (ESI) Anal. Calcd for $C_{102}H_{108}F_3N_6O_{30}^+$ $[M+H]^+$: 1953.7056, found 1953.7154.

Compound **26**: Compound **20** (1.60 g, 0.73 mmol) in DMF (14 mL) was treated with piperidine to deprotect Fmoc-group following the general procedure for Fmoc removal to afford the desired product **26** (1.31 g, 0.66 mmol) in 91% yield. ^1H NMR (500 MHz, $CDCl_3$) δ 8.41 (d, *J* = 7.6 Hz, 1H), 7.92 – 7.83 (m, 4H), 7.67 – 7.59 (m, 4H), 7.56 – 7.44 (m, 4H), 7.40 – 7.26 (m, 23H), 7.24 – 7.10 (m, 6H), 6.65 (t, *J* = 6.3 Hz, 1H), 5.46 (d, *J* = 3.6 Hz, 1H), 5.31 (t, *J* = 9.7 Hz, 1H), 5.22 (t, *J* = 6.3 Hz, 1H), 5.15 (d, *J* = 3.5 Hz, 1H), 5.09 – 4.92 (m, 6H), 4.88 (d, *J* = 12.1 Hz, 1H), 4.81 (d, *J* = 11.0 Hz, 1H), 4.74 – 4.52 (m, 8H), 4.43 (d, *J* = 7.9 Hz, 1H), 4.24 – 4.03 (m, 7H), 4.01 – 3.89 (m, 4H), 3.88 – 3.73 (m, 6H), 3.66 (s, 3H), 3.58 (ddd, *J* = 28.4, 11.6, 2.5 Hz, 3H), 3.48 – 3.41 (m, 2H), 3.38 (dd, *J* = 10.3, 3.6 Hz, 1H), 3.28 – 3.19 (m, 4H), 3.04 – 2.95 (m, 5H), 2.82 – 2.77 (m, 1H), 2.59 – 2.51 (m, 2H), 2.37 – 2.30 (m, 1H), 1.92 (s, 3H), 1.87 (s, 3H), 1.01 (s, 9H); ^{13}C NMR (125 MHz, $CDCl_3$) δ 209.36, 172.10, 168.97, 168.57, 168.21, 138.55, 138.38, 137.44, 136.96, 136.61,

135.75, 135.70, 134.79, 133.25, 133.17, 133.11, 133.07, 129.59, 129.50, 128.61, 128.50, 128.47, 128.42, 128.30, 128.21, 128.19, 128.07, 128.03, 128.01, 127.80, 127.60, 127.54, 127.42, 127.24, 126.76, 126.48, 126.36, 126.15, 102.50, 97.30, 83.63, 77.66, 77.50, 77.22, 74.99, 74.54, 74.32, 72.88, 71.28, 70.79, 69.50, 66.53, 63.01, 53.91, 52.12, 38.33, 37.42, 35.32, 31.42, 29.77, 29.03, 28.08, 20.83, 19.20; ; HRMS (ESI) Anal. Calcd for $C_{103}H_{116}F_3N_6O_{28}Si^+$ $[M+H]^+$: 1969.7553, found 1969.7454.

Compound **27:** The solution of compound **20** (220 mg, 100 µmol) in DCM:water (2.2 mL, 10:1) was treated with DDQ (34 mg, 150 µmol) for the deprotection of Nap-group according to general procedure to afford compound **27** (169 mg, 82% yield). $[\boldsymbol{\alpha}]_{\mathbf{D}}^{\mathbf{20}}$ +12.5º (*c* 1.0, $CHCl_3$); ^{1}H NMR (500 MHz, $CDCl_3$) δ 8.26 (d, *J* = 8.0 Hz, 1H), 7.77 (dd, *J* = 7.5, 3.7 Hz, 2H), 7.67 – 7.54 (m, 6H), 7.44 – 7.13 (m, 35H), 6.58 (t, *J* = 6.1 Hz, 1H), 5.43 (d, *J* = 3.6 Hz, 1H), 5.30 (t, *J* = 9.7 Hz, 1H), 5.22 (t, *J* = 5.6 Hz, 1H), 5.11 (d, *J* = 3.7 Hz, 1H), 5.08 – 5.02 (m, 3H), 4.97 – 4.90 (m, 2H), 4.87 (s, 1H), 4.86 – 4.76 (m, 3H), 4.70 (d, *J* = 11.0 Hz, 1H), 4.66 (s, 1H), 4.61 (d, *J* = 11.0 Hz, 1H), 4.58 – 4.51 (m, 2H), 4.45 (dd, *J* = 10.2, 7.0 Hz, 1H), 4.30 – 4.25 (m, 1H), 4.22 – 4.09 (m, 5H), 4.05 – 3.76 (m, 12H), 3.74 (s, 3H), 3.69 (d, *J* = 15.1 Hz, 1H), 3.64 – 3.55 (m, 2H), 3.52 – 3.47 (m, 1H), 3.38 (dd, *J* = 10.4, 3.6 Hz, 1H), 3.20 (s, 3H), 3.05 – 2.90 (m, 5H), 2.57 (ddd, *J* = 20.0, 14.7, 3.5 Hz, 2H), 2.37 – 2.29 (m, 1H), 1.92 (s, 3H), 1.86 (s, 3H), 1.02 (s, 9H);^{13}C NMR (125 MHz, $CDCl_3$) δ 209.32, 172.15, 168.98, 168.87, 168.62, 167.82, 157.66, 157.37, 156.53, 154.27, 143.28, 142.95, 141.23, 141.13, 138.66, 137.48, 137.39, 136.91, 136.62, 135.76, 135.71, 133.14, 133.07, 129.64, 129.56, 128.61, 128.52, 128.47, 128.39, 128.30, 128.25, 128.21, 128.11, 128.03, 127.84, 127.75, 127.62, 127.57, 127.33, 127.25, 127.21, 126.76, 125.12, 124.93, 120.22, 120.19, 117.03, 114.74, 100.45, 97.85, 97.79, 97.56, 82.22, 77.55, 77.50, 77.23, 77.12, 76.87, 74.88, 74.68, 74.63, 74.49, 74.30, 73.80, 73.43, 72.88, 72.21, 71.04, 70.46, 69.44, 66.91, 66.76, 66.53, 65.66, 62.95, 61.66, 60.13, 53.81, 52.46, 52.27, 46.66, 38.41, 37.41, 35.30, 29.79, 29.70, 29.14, 27.93, 26.72, 20.81, 19.20; HRMS (ESI) Anal. Calcd for $C_{107}H_{118}F_3N_6O_{30}Si^+$ $[M+H]^+$: 2051.7608, found 2051.7550.

Compound **28:** The solution of compound **20** (350 mg, 159 µmol) in pyridine:acetic acid (2.5 mL, 3:2) was treated with hydrazine monohydrate (80µL, 1.6 mmol) for the deprotection of Lev-group according to general procedure to afford compound **28** (316 mg, 95% yield). ^{1}H NMR (500 MHz, $CDCl_3$) δ 7.89 – 7.82 (m, 4H), 7.70 (d, *J* = 7.5 Hz, 1H), 7.63 – 7.56 (m, 4H), 7.52 – 7.41 (m, 7H), 7.40 – 7.04 (m, 35H), 6.95 (t, *J* = 6.2 Hz, 1H), 5.28 (t, *J* = 9.7 Hz, 1H), 5.22 (d, *J* = 3.5 Hz, 1H), 5.13 – 4.96 (m, 5H), 4.94 – 4.82 (m, 3H), 4.71 – 4.65 (m, 1H), 4.68 (dd, *J* = 13.7, 11.2 Hz, 2H), 4.65 – 4.57 (m, 2H), 4.54 – 4.48 (m, 2H), 4.39 (d, *J* = 12.4 Hz, 1H), 4.24 – 4.05 (m, 7H), 3.96 – 3.88 (m, 3H), 3.86 – 3.80 (m, 2H), 3.79 – 3.76 (m, 1H), 3.69 – 3.58 (m, 6H), 3.58 – 3.51 (m, 3H), 3.49 – 3.40 (m, 3H), 3.37 (dd, *J* = 10.2, 3.4 Hz, 1H), 3.31 – 3.26 (m, 1H), 3.23 (s, 3H), 3.20 – 3.13 (m, 2H), 3.12 – 2.99 (m, 3H), 2.48 (t, *J* = 9.2 Hz, 1H), 1.89 (s, 3H), 0.98 (s, 9H);^{13}C NMR (125 MHz, $CDCl_3$) δ 169.64, 169.07, 168.99, 167.68, 157.33, 153.79, 143.48, 143.38, 141.55, 141.47, 138.36, 137.72, 137.42, 136.93, 136.03, 135.69, 134.42, 133.20, 133.08, 133.01, 132.99, 129.61, 129.53, 128.70, 128.55, 128.48, 128.34, 128.25, 128.07, 128.02, 127.90, 127.81, 127.71, 127.59,

127.54, 127.47, 127.36, 127.29, 127.11, 126.79, 126.39, 126.26, 124.79, 124.55, 120.28, 120.23, 100.53, 100.37, 98.23, 97.04, 94.96, 81.48, 77.90, 77.70, 74.96, 74.65, 74.08, 74.00, 73.78, 73.53, 72.97, 72.83, 71.14, 70.90, 69.54, 69.05, 68.54, 66.98, 66.32, 63.02, 61.62, 52.93, 52.68, 52.16, 46.75, 37.31, 34.90, 29.70, 29.49, 26.69, 20.84, 19.16; HRMS (ESI) Anal. Calcd for $C_{113}H_{120}F_3N_6O_{28}Si^+$ $[M+H]^+$: 2093.7866, found 2051.7550.

Scheme S5. Syntheses of tetrasaccharides **29 - 31**.

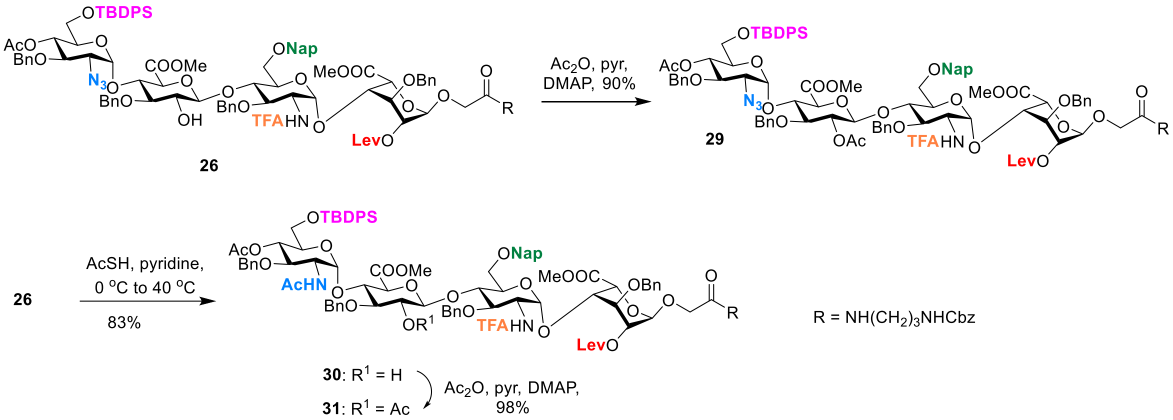

Compound 29: Compound **26** (0.72 g, 0.33 mmol) was dissolved in pyridine and *O*-acylated following the general procedure to produce product **29** (0.60 g, 0.298 mmol) in 90% yield. .$[\alpha]_D^{20}$ -2.0° (*c* 1.0, $CHCl_3$); ^{1}H NMR (500 MHz, $CDCl_3$) δ 8.39 (d, *J* = 7.7 Hz, 1H), 7.98 (d, *J* = 8.4 Hz, 1H), 7.94 – 7.89 (m, 2H), 7.88 (s, 1H), 7.66 – 7.57 (m, 5H), 7.57 – 7.50 (m, 2H), 7.40 – 7.26 (m, 23H), 7.22 – 7.09 (m, 7H), 6.67 (t, *J* = 6.1 Hz, 1H), 5.37 – 5.30 (m, 2H), 5.21 (t, *J* = 6.2 Hz, 1H), 5.17 (d, *J* = 3.6 Hz, 1H), 5.06 (s, 2H), 5.01 (d, *J* = 12.2 Hz, 2H), 4.96 (s, 2H), 4.89 – 4.82 (m, 2H), 4.70 (dd, *J* = 27.6, 11.0 Hz, 2H), 4.63 – 4.52 (m, 4H), 4.48 (d, *J* = 11.2 Hz, 1H), 4.34 (d, *J* = 8.1 Hz, 1H), 4.23 (d, *J* = 15.0 Hz, 1H), 4.17 (dd, *J* = 13.0, 5.4 Hz, 2H), 4.04 (dd, *J* = 15.3, 6.0 Hz, 2H), 4.00 – 3.94 (m, 3H), 3.92 – 3.68 (m, 11H), 3.64 – 3.52 (m, 3H), 3.48 (dt, *J* = 10.2, 2.3 Hz, 1H), 3.42 (dd, *J* = 10.3, 3.6 Hz, 1H), 3.20 (s, 3H), 3.09 (t, *J* = 9.2 Hz, 1H), 3.05 – 2.94 (m, 5H), 2.59 – 2.51 (m, 2H), 2.34 (dt, *J* = 16.7, 3.6 Hz, 1H), 1.90 (s, 3H), 1.87 (s, 3H), 1.78 (s, 3H), 1.00 (s, 9H); ^{13}C NMR (125 MHz, $CDCl_3$) δ 209.42, 172.26, 168.98, 168.93, 168.89, 168.59, 167.77, 156.57, 138.78, 137.70, 137.39, 137.01, 136.61, 135.74, 135.70, 134.68, 133.28, 133.13, 133.05, 129.62, 129.54, 128.81, 128.60, 128.55, 128.47, 128.43, 128.28, 128.25, 128.14, 128.11, 128.07, 128.03, 128.02, 127.82, 127.66, 127.61, 127.55, 127.13, 127.00, 126.92, 126.83, 126.62, 126.37, 99.79, 98.35, 97.42, 82.14, 77.22, 74.84, 74.70, 74.46, 74.12, 74.03, 73.68, 72.95, 72.88, 71.18, 70.89, 69.52, 66.55, 65.79, 63.08, 61.53, 53.79, 52.50, 52.22, 38.39, 37.42, 35.31, 29.80, 29.05, 27.94, 26.71, 20.85, 20.76, 19.19. HRMS (ESI) Anal. Calcd for $C_{105}H_{118}F_3N_6O_{29}Si^+$ $[M+H]^+$: 2011.7659, found 2011.7598.

Compound 30: To the solution of compound **26** (434 mg, 0.22 mmol) in pyridine (3.0 mL) was added thioacetic acid (3.0 mL) at 0 °C. The reaction mixture was then heated to 40 °C and stirred at 40 °C overnight. Once the reaction was completed, it was concentrated and purified by flash column chromatography (toluene : ethyl acetate 10:1 to 1:1) to afford the desired product (364 mg, 0.183 mmol) with 83% yield. ^{1}H NMR (500 MHz, $CDCl_3$) δ 8.47 (d, *J* = 7.5 Hz, 1H), 7.90 – 7.83 (m, 4H), 7.72 – 7.65 (m, 4H), 7.58 – 7.45 (m, 4H), 7.45 – 7.18 (m, 27H), 7.14 (d, *J* = 7.0 Hz, 2H), 6.68 (t, *J* = 6.3 Hz, 1H), 6.21 (d, *J* = 9.7 Hz, 1H), 5.45 (t, *J* = 9.6 Hz, 1H), 5.25 – 5.15 (m, 2H), 5.07 (s, 2H), 5.04 – 4.90 (m, 4H), 4.90 – 4.85 (m, 2H), 4.74 (d, *J* = 11.0 Hz, 1H), 4.65 – 4.52 (m, 6H), 4.40 – 4.33 (m, 1H), 4.26 – 4.18 (m, 3H), 4.18 – 4.07 (m, 4H), 4.04 – 3.94 (m, 3H), 3.87 – 3.76 (m, 5H), 3.74 (s, 3H), 3.72 – 3.66 (m, 3H), 3.51 (d, *J* = 9.4 Hz, 1H), 3.43 – 3.37 (m, 1H), 3.27 (s, 3H), 3.08 – 2.88 (m, 6H), 2.68 – 2.50 (m, 4H), 2.37 – 2.31 (m, 1H), 1.99 (s, 3H), 1.97 (s, 3H), 1.27 (s, 3H), 1.07 (s, 9H); ^{13}C NMR (125 MHz, $CDCl_3$) δ 209.56, 172.07, 170.09, 169.01, 168.91, 168.68, 167.66, 157.43, 156.59, 138.29, 138.25, 137.28, 136.95, 136.61, 135.78, 134.95, 133.30, 133.20, 133.15, 133.10, 129.71, 129.66, 129.03, 128.67, 128.64, 128.61, 128.49, 128.34, 128.25, 128.22, 128.06, 128.02, 127.92, 127.88, 127.84, 127.68, 127.65, 127.60, 127.46, 127.08, 126.98, 126.50, 126.34, 125.29, 102.68, 99.94, 99.26, 98.02, 81.68, 77.86, 77.73, 77.24, 76.88, 75.78, 75.18, 75.12, 75.08, 74.03, 73.87, 73.05, 72.91, 72.21, 71.28, 70.51, 68.45, 66.96, 66.56, 65.81, 62.38, 53.87, 52.48, 52.42, 51.36, 38.36, 37.44, 35.35, 29.79, 29.13, 28.09, 26.82, 22.27, 20.98, 19.24. HRMS (ESI) Anal. Calcd for $[M+H]^+$: $C_{105}H_{119}F_3N_4O_{29}Si^+$ 1985.7754, found 1985.7173.

Compound 31: Compound **30** (240 mg, 0.12 mmol) was acylated in pyridine (1.0 mL) with acetic anhydride (0.5 mL) according to the general procedure of *N*-acylation to afford the desired product **31** (237 mg 0.117 mmol) with 98% yield. ^{1}H NMR (500 MHz, $CDCl_3$) δ 8.43 (d, *J* = 7.8 Hz, 1H), 7.96 – 7.86 (m, 4H), 7.72 – 7.58 (m, 5H), 7.57 – 7.51 (m, 2H), 7.47 – 7.26 (m, 19H), 7.26 – 7.11 (m, 9H), 7.08 (d, *J* = 7.0 Hz, 2H), 6.70 (t, *J* = 6.3 Hz, 1H), 5.91 (d, *J* = 9.6 Hz, 1H), 5.47 (t, *J* = 9.5 Hz, 1H), 5.22 – 5.16 (m, 2H), 5.09- 5.03 (m, 3H), 5.00 - 4.94 (m, 2H), 4.91 (d, *J* = 11.2 Hz, 1H), 4.87 (d, *J* = 3.0 Hz, 1H), 4.85 – 4.80 (m, 1H), 4.75 (d, *J* = 11.0 Hz, 1H), 4.64 – 4.44 (m, 6H), 4.39 – 4.33 (m, 1H), 4.29 – 4.13 (m, 5H), 4.08 – 4.00 (m, 2H), 4.00 – 3.67 (m, 15H), 3.55 (d, *J* = 9.5 Hz, 1H), 3.30 (s, 3H), 3.07 – 2.93 (m, 5H), 2.59 – 2.51 (m, 2H), 2.43 (t, *J* = 9.5 Hz, 1H), 2.39 – 2.32 (m, 1H), 2.03 (s, 3H), 1.93 (s, 3H), 1.91 (s, 3H), 1.38 – 1.30 (m, 2H), 1.20 (s, 3H), 1.08 (s, 9H); ^{13}C NMR (125 MHz, $CDCl_3$) δ 209.57, 172.25, 170.05, 169.01, 168.86, 168.60, 167.19, 156.59, 138.44, 138.12, 136.99, 136.59, 136.47, 135.76, 135.74, 135.12, 133.36, 133.14, 133.07, 129.75, 129.73, 128.77, 128.61, 128.47, 128.41, 128.35, 128.30, 128.21, 128.05, 128.02, 127.98, 127.90, 127.83, 127.80, 127.70, 127.68, 127.66, 127.61, 127.39, 127.16, 126.76, 126.55, 99.98, 98.29, 80.44, 78.18, 77.63, 76.17, 74.95, 74.75, 73.87, 73.53, 72.97, 72.86, 72.49, 71.13, 70.39, 68.47, 66.67, 65.81, 62.40, 53.63, 52.55, 51.24, 38.44, 37.42, 35.32, 29.81, 29.16, 27.93, 26.82, 22.14, 21.00, 20.83, 19.25, HRMS (ESI) Anal. Calcd for $C_{107}H_{122}F_3N_4O_{30}Si$ $[M+H]^+$: 2027.7860, found 2027.7249.

Compound 32: The solution of compound **30** (55 mg, 27 µmol) in pyridine was subjected for the *O*-sulfation, TBDPS-deprotection and saponification according to the general procedures to afford

compound **32** (25 mg, 59% over 3 steps). ^{1}H NMR (500 MHz, CD_3OD) δ 7.89 – 7.76 (m, 4H), 7.50 – 7.15 (m, 25H), 5.39 (s, 1H), 5.26 (d, *J* = 3.7 Hz, 1H), 5.19 – 5.00 (m, 5H), 4.88 – 4.57 (m, 10H), 4.53 – 4.43 (m, 2H), 4.35 (d, *J* = 10.1 Hz, 1H), 4.31 – 3.94 (m, 12H), 3.90 (d, *J* = 8.8 Hz, 1H), 3.86 – 3.51 (m, 9H), 3.22 (s, 1H), 3.14 – 2.87 (m, 5H), 1.84 (s, 3H), 1.46 – 1.36 (m, 2H). ^{13}C NMR (126 MHz, CD_3OD) δ 128.55, 128.21, 128.15, 128.06, 127.90, 127.83, 127.76, 127.71, 127.55, 127.32, 127.01, 126.85, 125.75, 97.04, 73.56, 72.85, 72.46, 65.99, 60.86, 52.57, 48.10, 47.93, 47.76, 47.59, 47.42, 47.25, 47.08, 37.54, 35.61, 29.27. HRMS (ESI) Anal. Calcd for $C_{78}H_{89}N_4O_{28}S^-$ $[M-H]^-$: 1561.5390, found 1561.5411.

Compound 33: To the solution of compound **30** (200 mg, 0.10 mmol) in pyridine : acetic acid mixture was subjected to Lev-deprotection according to the general procedure to afford compound **33** (190 mg, 98% yield). ^{1}H NMR (500 MHz, $CDCl_3$) δ 7.89 – 7.81 (m, 4H), 7.67 (dd, *J* = 10.6, 7.6 Hz, 4H), 7.50 (dt, *J* = 9.8, 7.3 Hz, 4H), 7.45 – 7.27 (m, 20H), 7.25 – 7.14 (m, 10H), 6.89 (t, *J* = 5.9 Hz, 1H), 6.17 (d, *J* = 9.7 Hz, 1H), 5.45 (t, *J* = 9.5 Hz, 1H), 5.10 – 4.92 (m, 7H), 4.88 (s, 1H), 4.80 (d, *J* = 11.4 Hz, 1H), 4.74 (s, 1H), 4.68 (d, *J* = 11.4 Hz, 1H), 4.64 – 4.52 (m, 6H), 4.37 (ddd, *J* = 12.9, 12.3, 7.3 Hz, 3H), 4.24 – 4.04 (m, 9H), 3.95 (d, *J* = 6.0 Hz, 1H), 3.79 (t, *J* = 9.6 Hz, 3H), 3.77 – 3.61 (m, 10H), 3.51 (d, *J* = 9.4 Hz, 1H), 3.44 – 3.39 (m, 1H), 3.32 (s, 3H), 3.26 – 3.18 (m, 2H), 3.05 – 2.98 (m, 2H), 2.75 (t, *J* = 9.0 Hz, 1H), 1.99 (s, 3H), 1.31 (s, 3H), 1.06 (s, 9H). ^{13}C NMR (125 MHz, $CDCl_3$) δ 170.06, 169.09, 168.98, 138.23, 135.77, 134.75, 133.23, 133.09, 129.65, 128.70, 128.67, 128.60, 128.50, 128.32, 128.12, 127.95, 127.85, 127.81, 127.65, 127.63, 127.58, 126.63, 126.49, 126.34, 102.62, 100.62, 99.74, 96.77, 75.05, 74.54, 73.93, 72.73, 72.15, 70.50, 67.03, 66.04, 52.91, 52.59, 52.41, 37.26, 29.41, 26.79, 22.35, 20.96, 19.22. HRMS (ESI) Anal. Calcd for $C_{100}H_{114}F_3N_4O_{27}S^+$ $[M+H]^+$: 1887.7386, found 1887.7054.

Scheme S6. Syntheses of tetrasaccharides **40** - **55**.

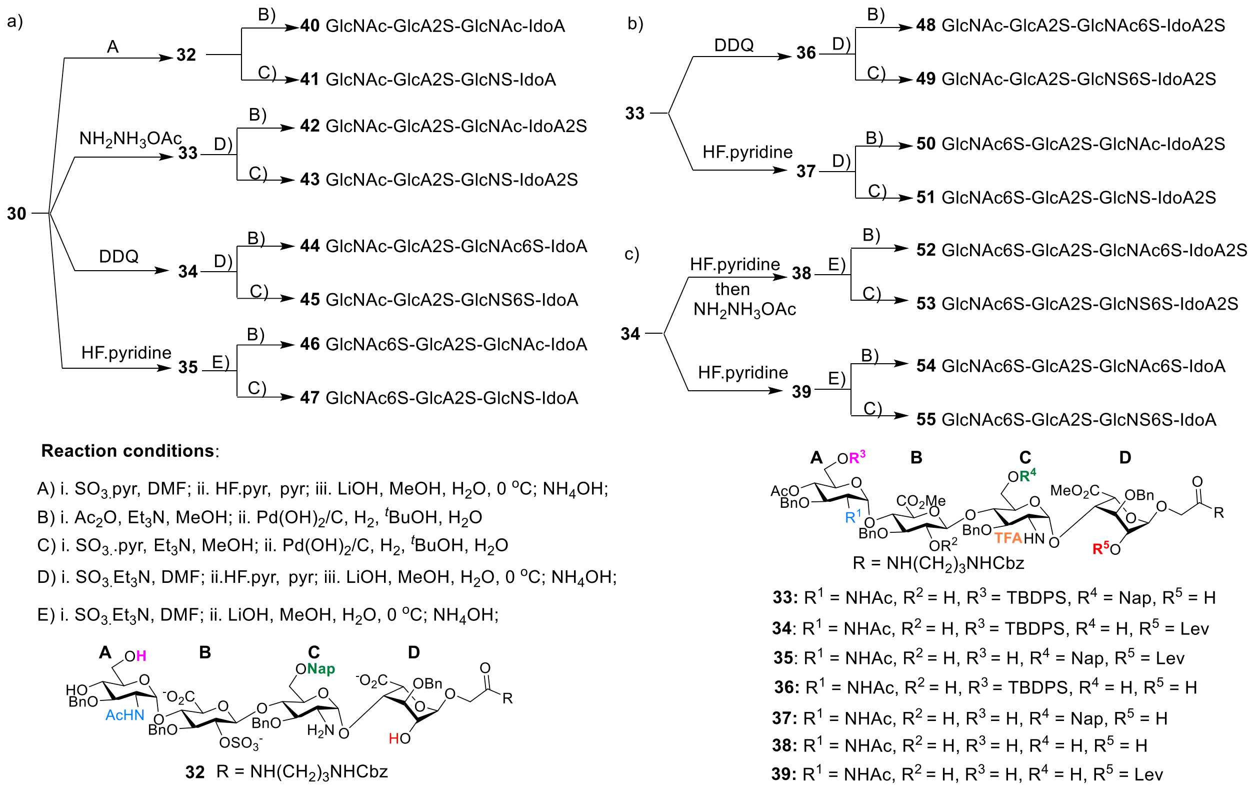

Compound **34:** The solution of compound **30** (70 mg, 35 μmol) in DCM:water (1.1 mL) was subjected to the general procedure for the deprotection of Nap-group to afford compound **34** (63 mg, 34 μmol) in 97% yield. ^{1}H NMR (500 MHz, $CDCl_3$) δ 7.70 – 7.62 (m, 4H), 7.44 – 7.28 (m, 22H), 7.27 – 7.14 (m, 9H), 6.69 (t, *J* = 6.2 Hz, 1H), 6.20 (d, *J* = 9.2 Hz, 1H), 5.37 (t, *J* = 9.5 Hz, 1H), 5.19 (d, *J* = 5.9 Hz, 1H), 5.12 (d, *J* = 3.6 Hz, 1H), 5.10 – 4.92 (m, 8H), 4.76 (t, *J* = 10.0 Hz, 2H), 4.66 – 4.57 (m, 3H), 4.55 (s, 2H), 4.42 – 4.35 (m, 1H), 4.26 – 4.12 (m, 5H), 4.05 – 3.85 (m, 9H), 3.82 – 3.68 (m, 7H), 3.65 (d, *J* = 2.6 Hz, 2H), 3.60 (t, *J* = 7.8 Hz, 1H), 3.30 (s, 3H), 3.08 – 2.97 (m, 5H), 2.59 (dd, *J* = 15.7, 10.2 Hz, 2H), 2.37 (dd, *J* = 14.0, 3.9 Hz, 1H), 1.95 (s, 3H), 1.94 (s, 3H), 1.58 (s, 3H), 1.05 (s, 9H); ^{13}C NMR (125 MHz, $CDCl_3$) δ 209.73, 172.08, 170.13, 169.04, 168.87, 168.22, 156.62, 138.34, 138.15, 137.34, 136.94, 135.77, 135.73, 133.21, 133.13, 129.64, 129.60, 128.67, 128.63, 128.49, 128.37, 128.33, 128.26, 128.07, 128.01, 127.98, 127.84, 127.65, 127.62, 127.60, 127.41, 126.77, 102.81, 98.96, 98.60, 98.16, 82.01, 77.62, 77.34, 76.20, 75.30, 74.80, 74.46, 73.80, 73.70, 72.85, 71.96, 70.86, 68.67, 66.57, 65.89, 62.33, 54.16, 52.55, 52.33, 51.38, 38.44, 37.45, 35.35, 29.79, 29.71, 29.11, 28.14, 26.76, 22.68, 20.89, 19.20; HRMS (ESI) Anal. Calcd for $C_{94}H_{112}F_3N_4O_{29}Si^+$ $[M+H]^+$: 1845.7128, found 1845.7115.

Compound **35:** The solution of compound **30** (30 mg, 15 μmol) in pyridine (0.6 mL) was subjected to the general procedue for deprotection of TBDPS-group to afford compound **35** (24 mg, 13.7 μmol) in 91% yield. ^{1}H NMR (500 MHz, $CDCl_3$) δ 8.51 (d, *J* = 7.5 Hz, 1H), 7.94 – 7.87 (m, 4H),

7.59 (dd, *J* = 8.4, 1.2 Hz, 1H), 7.53 (dd, *J* = 9.4, 4.9 Hz, 2H), 7.39 – 7.27 (m, 15H), 7.24 (ddd, *J* = 15.3, 9.2, 4.5 Hz, 7H), 7.17 (d, *J* = 7.1 Hz, 2H), 6.68 (t, *J* = 6.1 Hz, 1H), 5.95 (d, *J* = 9.7 Hz, 1H), 5.23 – 5.17 (m, 2H), 5.07 (s, 2H), 5.04 – 4.93 (m, 4H), 4.89 (d, *J* = 11.2 Hz, 1H), 4.81 (d, *J* = 3.5 Hz, 1H), 4.75 (d, *J* = 11.0 Hz, 1H), 4.71 – 4.55 (m, 7H), 4.35 – 4.27 (m, 2H), 4.26 – 4.19 (m, 2H), 4.17 – 4.09 (m, 5H), 4.00 (t, *J* = 12.5 Hz, 2H), 3.90 – 3.81 (m, 2H), 3.81 – 3.66 (m, 7H), 3.59 (s, 1H), 3.57 – 3.50 (m, 5H), 3.01 (dd, *J* = 25.3, 11.1 Hz, 6H), 2.75 (t, *J* = 9.1 Hz, 1H), 2.66 (s, 1H), 2.63 – 2.53 (m, 3H), 2.36 (d, *J* = 16.3 Hz, 1H), 2.11 (s, 3H), 1.96 (s, 3H), 1.27 (s, 3H).; ^{13}C NMR (125 MHz, $CDCl_3$) δ 209.65, 172.06, 170.40, 170.13, 168.88, 168.64, 167.80, 157.77, 156.59, 138.30, 138.11, 137.42, 136.96, 135.03, 133.31, 133.10, 128.72, 128.64, 128.49, 128.45, 128.43, 128.39, 128.34, 128.29, 128.25, 128.06, 128.02, 127.83, 127.73, 127.53, 127.48, 126.90, 126.83, 126.46, 126.27, 102.68, 99.96, 99.46, 98.05, 81.56, 78.54, 77.68, 76.04, 75.46, 75.37, 75.31, 74.09, 73.88, 73.03, 72.90, 72.44, 72.33, 71.30, 69.83, 67.42, 66.94, 66.66, 66.56, 65.78, 61.47, 53.94, 52.83, 52.46, 51.85, 38.39, 37.43, 35.33, 29.79, 29.71, 29.09, 28.12, 22.28, 20.93; HRMS (ESI) Anal. Calcd for $C_{89}H_{102}F_3N_4O_{29}$ $[M+H]^+$: 1748.6576, found 1748.6422.

Compound **36:** The solution of compound **33** (125 mg, 66 µmol) in DCM:water (3.0 mL) was subjected to the general condition for deprotection of Nap-group to afford compound **36** (95 mg, 54 µmol) in 82% yield. ^{1}H NMR (500 MHz, $CDCl_3$) δ 7.78 (d, *J* = 8.7 Hz, 1H), 7.64 (ddd, *J* = 13.7, 7.9, 1.3 Hz, 4H), 7.47 – 7.16 (m, 30H), 6.88 (t, *J* = 6.0 Hz, 1H), 6.18 (d, *J* = 9.5 Hz, 1H), 5.36 (t, *J* = 9.4 Hz, 1H), 5.13 – 5.08 (m, 1H), 5.07 – 4.97 (m, 4H), 4.90 – 4.86 (m, 2H), 4.81 (d, *J* = 11.4 Hz, 1H), 4.75 (s, 1H), 4.69 (d, *J* = 8.3 Hz, 2H), 4.56 (dt, *J* = 17.6, 6.3 Hz, 5H), 4.39 – 4.33 (m, 1H), 4.28 – 4.22 (m, 1H), 4.18 (t, *J* = 12.7 Hz, 2H), 4.12 (d, *J* = 6.3 Hz, 2H), 4.05 – 3.92 (m, 5H), 3.86 (d, *J* = 8.3 Hz, 1H), 3.79 – 3.62 (m, 11H), 3.54 (t, *J* = 8.2 Hz, 2H), 3.39 (s, 3H), 3.28 – 3.18 (m, 2H), 3.12 – 2.95 (m, 3H), 1.93 (s, 3H), 1.52 (s, 3H), 1.04 (s, 9H); ^{13}C NMR (125 MHz, $CDCl_3$) δ 170.12, 169.43, 169.17, 169.01, 168.22, 157.47, 138.14, 137.43, 137.17, 136.78, 135.91, 135.75, 135.73, 133.20, 133.10, 129.64, 129.62, 128.71, 128.68, 128.60, 128.42, 128.39, 128.36, 128.33, 128.28, 128.00, 127.83, 127.77, 127.74, 127.64, 127.62, 127.61, 102.58, 100.56, 98.90, 96.39, 82.00, 78.00, 77.39, 77.22, 76.21, 75.38, 75.01, 74.52, 73.99, 73.09, 72.73, 72.29, 72.23, 72.03, 70.94, 68.59, 67.65, 67.02, 65.98, 62.26, 60.63, 53.33, 52.64, 52.48, 51.34, 37.29, 34.96, 29.70, 29.36, 26.75, 22.67, 20.88, 19.19. HRMS (ESI) Anal. Calcd for $C_{89}H_{105}F_3N_4O_{27}SiNa_2$ $[M+2Na]^{2+}$: 896.3236, found 896.3268.

Compound **37:** The solution of compound **33** (47 mg, 25 µmol) in pyridine was subjected to silyl-group deprotection according to the general procedure to afford compound **37** (35.1 mg, 21.2 µmol) in 85% yield. ^{1}H NMR (500 MHz, $CDCl_3$) δ 7.94 – 7.85 (m, 4H), 7.64 (d, *J* = 8.8 Hz, 1H), 7.57 (d, *J* = 8.4 Hz, 1H), 7.53 (dd, *J* = 6.3, 3.2 Hz, 2H), 7.37 – 7.27 (m, 14H), 7.23 (ddd, *J* = 23.6, 16.2, 5.8 Hz, 10H), 6.90 (t, *J* = 6.2 Hz, 1H), 5.98 (d, *J* = 9.7 Hz, 1H), 5.13 – 5.09 (m, 1H), 5.09 – 4.96 (m, 5H), 4.94 (d, *J* = 5.7 Hz, 2H), 4.84 – 4.79 (m, 2H), 4.75 (d, *J* = 1.6 Hz, 1H), 4.70 (dd, *J* = 15.1, 11.2 Hz, 2H), 4.64-4.55 (m, 5H), 4.43 (d, *J* = 11.3 Hz, 1H), 4.35 – 4.07 (m, 10H), 3.96 (d, *J* = 7.3 Hz, 2H), 3.80 – 3.65 (m, 11H), 3.58 (s, 3H), 3.45 (t, *J* = 8.4 Hz, 1H), 3.31 (s, 1H), 3.23 (td,

J = 13.4, 6.7 Hz, 2H), 3.09 (dd, J = 14.1, 5.8 Hz, 1H), 3.01 (d, J = 5.6 Hz, 2H), 2.84 (t, J = 9.1 Hz, 1H), 2.69 (s, 1H), 2.10 (s, 3H), 1.29 (s, 3H); ^{13}C NMR (125 MHz, $CDCl_3$) δ 170.38, 170.20, 169.48, 169.16, 167.84, 138.12, 137.90, 137.38, 136.77, 134.81, 133.26, 133.12, 128.72, 128.60, 128.56, 128.42, 128.40, 128.37, 128.34, 128.16, 127.87, 127.85, 127.83, 127.72, 127.66, 127.57, 127.53, 127.49, 126.60, 126.47, 126.29, 102.62, 100.60, 99.91, 96.80, 81.69, 78.40, 77.67, 77.61, 77.23, 76.88, 75.47, 75.31, 75.26, 74.37, 73.97, 73.33, 72.83, 72.72, 72.47, 72.29, 71.29, 69.81, 67.70, 67.03, 65.99, 61.45, 52.91, 52.84, 52.60, 51.87, 37.28, 34.94, 29.71, 29.40, 22.31, 20.92; HRMS (ESI) Anal. Calcd for $C_{84}H_{96}F_3N_4O_{27}$ $[M+H]^{2+}$: 1649.6209, found 1649.6112.

Compound **38:** The solution of compound **34** (49 mg, 28 µmol) in pyridine was subjected for silyl-group and Lev-group deprotection according to the general procedures to afford compound **38** (38.5 mg, 25.5 µmol) in 91% yield. ^{1}H NMR (500 MHz, $CDCl_3$) δ 7.91 (d, J = 8.6 Hz, 1H), 7.38 – 7.16 (m, 25H), 6.87 (t, J = 5.8 Hz, 1H), 6.03 (d, J = 9.2 Hz, 1H), 5.19 – 5.12 (m, 1H), 5.07 – 4.92 (m, 6H), 4.89 (s, 1H), 4.82 (d, J = 11.2 Hz, 1H), 4.74 (s, 1H), 4.67 (dd, J = 9.5, 5.0 Hz, 2H), 4.62 – 4.49 (m, 5H), 4.34 – 4.08 (m, 7H), 4.05 – 3.84 (m, 7H), 3.81 – 3.66 (m, 8H), 3.61 (s, 3H), 3.24 – 3.12 (m, 2H), 3.11 – 2.93 (m, 3H), 2.77 (s, 2H), 2.02 (s, 3H), 1.45 (s, 3H); ^{13}C NMR (125 MHz, $CDCl_3$) δ 170.47, 170.30, 169.52, 169.26, 168.19, 157.39, 138.06, 137.61, 137.31, 136.83, 136.01, 128.74, 128.70, 128.59, 128.43, 128.40, 128.37, 128.30, 127.85, 127.83, 127.75, 127.69, 127.65, 127.63, 116.94, 114.65, 102.92, 100.48, 99.21, 96.50, 81.94, 78.09, 77.41, 75.97, 75.70, 75.08, 74.76, 74.65, 73.16, 72.80, 72.70, 72.35, 72.09, 72.02, 69.99, 67.62, 66.96, 66.78, 65.95, 61.27, 60.80, 53.27, 52.89, 52.67, 51.93, 37.32, 35.03, 29.71, 29.40, 22.57, 20.85; HRMS (ESI) Anal. Calcd for $C_{73}H_{87}F_3N_4O_{27}Na^+$ $[M+Na]^+$: 1531.5402, found 1531.5394.

Compound **39:** The solution of compound **34** (80 mg, 43.3 µmol) in pyridine was subjected to silyl-group deprotection according to the general procedure to afford compound **39** (57.5 mg, 35.9 µmol) in 83% yield. ^{1}H NMR (500 MHz, $CDCl_3$) δ 8.51 (d, J = 7.8 Hz, 1H), 7.39 – 7.13 (m, 25H), 6.77 – 6.71 (m, 1H), 6.76 – 6.70 (m, 1H), 6.06 (d, J = 9.5 Hz, 1H), 5.24 (d, J = 5.8 Hz, 1H), 5.15 – 4.94 (m, 9H), 4.77 (d, J = 11.1 Hz, 1H), 4.71 (d, J = 7.7 Hz, 1H), 4.67 – 4.51 (m, 5H), 4.37 – 4.29 (m, 1H), 4.25 – 4.13 (m, 4H), 4.04 – 3.84 (m, 8H), 3.79 (s, 3H), 3.75 – 3.68 (m, 2H), 3.67 – 3.54 (m, 4H), 3.51 (s, 3H), 3.02 (s, 5H), 2.63 – 2.54 (m, 2H), 2.37 (d, J = 16.2 Hz, 1H), 2.05 (d, J = 3.6 Hz, 3H), 1.96 (s, 3H), 1.49 (s, 3H), 1.36 (s, 2H); ^{13}C NMR (125 MHz, $CDCl_3$) δ 209.68, 172.09, 170.50, 170.21, 168.93, 168.88, 168.19, 157.81, 157.52, 156.65, 138.31, 138.08, 137.45, 136.96, 136.58, 128.71, 128.62, 128.49, 128.42, 128.31, 128.07, 128.00, 127.84, 127.73, 127.64, 127.46, 127.23, 126.71, 117.05, 114.76, 103.20, 99.06, 98.81, 98.20, 82.04, 77.69, 77.37, 75.65, 75.32, 75.16, 74.99, 73.72, 73.60, 72.82, 72.73, 72.37, 71.94, 70.03, 66.57, 65.92, 61.24, 60.72, 54.06, 52.75, 52.55, 51.89, 38.45, 37.47, 35.39, 29.77, 29.70, 29.11, 28.13, 22.60, 20.86; HRMS (ESI) Anal. Calcd for $C_{78}H_{94}F_3N_4O_{29}$ $[M+H]^+$: 1607.5950, found 1607.5985.

Compound **40:** The solution of compound **32** (11 mg, 7 µmol) in acetic acid and triethylamine was subjected for *N*-acylation followed by hydrogenation according to the general procedures to afford

compound **40** (4.6 mg, 67% over 2 steps). $[\alpha]_D^{20}$ +4.6º (*c* 0.1, H_2O); ^{1}H NMR (500 MHz, D_2O) δ 5.25 (d, *J* = 3.7 Hz, 1H), 5.00 (d, *J* = 3.7 Hz, 1H), 4.71 (d, *J* = 3.6 Hz, 1H), 4.54 (d, *J* = 7.7 Hz, 1H), 4.35 (d, *J* = 3.0 Hz, 1H), 4.10 – 4.01 (m, 2H), 3.95 (t, *J* = 8.2 Hz, 1H), 3.89 (t, *J* = 3.4 Hz, 1H), 3.85 – 3.65 (m, 10H), 3.63 (d, *J* = 2.8 Hz, 2H), 3.58 – 3.52 (m, 3H), 3.49 (t, *J* = 9.4 Hz, 1H), 3.31 (t, *J* = 9.6 Hz, 1H), 3.24 – 3.17 (m, 1H), 3.15 – 3.08 (m, 1H), 2.79 (t, *J* = 7.7 Hz, 2H), 1.88 (s, 3H), 1.84 (s, 3H), 1.69 (td, *J* = 14.4, 7.3 Hz, 2H); ^{13}C NMR (125 MHz, D_2O) δ 101.69, 100.32, 96.86, 94.56, 79.98, 79.25, 75.75, 75.25, 74.75, 70.74, 69.71, 69.59, 69.56, 69.55, 69.55, 69.54, 66.92, 59.89, 59.70, 59.01, 58.98, 53.37, 37.00, 35.76, 35.71, 26.97, 21.84, 21.80; HRMS (ESI) Anal. Calcd for $C_{33}H_{53}N_4O_{27}S^-$ $[M-H]^-$: 969.2623, found 969.2670.

Compound **41:** The solution of compound **32** (7 mg, 4.4 µmol) in methanol was subjected to *N*-sulfation followed by hydrogenation according to the general procedures to afford compound **41** (2.5 mg, 56% over 2 steps). $[\alpha]_D^{20}$ +4.1° (*c* 0.1, H_2O); ^{1}H NMR (500 MHz, D_2O) δ 5.25 (d, *J* = 3.7 Hz, 1H), 5.17 (dd, *J* = 13.1, 3.7 Hz, 1H), 4.75 (dd, *J* = 10.6, 2.4 Hz, 1H), 4.54 (d, *J* = 7.7 Hz, 1H), 4.32 (dd, *J* = 6.3, 2.5 Hz, 1H), 4.13 – 3.91 (m, 4H), 3.89 (d, *J* = 2.9 Hz, 1H), 3.84 – 3.75 (m, 2H), 3.74 – 3.61 (m, 8H), 3.61 – 3.52 (m, 4H), 3.47 (t, *J* = 9.5 Hz, 1H), 3.31 (t, *J* = 9.6 Hz, 1H), 3.22 – 3.10 (m, 2H), 3.06 (dd, *J* = 10.4, 3.6 Hz, 1H), 2.78 (dd, *J* = 14.7, 7.0 Hz, 1H), 2.57 (t, *J* = 6.6 Hz, 1H), 1.87 (s, 3H), 1.75 – 1.63 (m, 2cH). ^{13}C NMR (125 MHz, D_2O) δ 101.38, 100.40, 96.83, 95.73, 80.03, 79.33, 75.86, 75.52, 75.16, 71.68, 70.35, 69.68, 69.58, 68.86, 68.82, 68.42, 66.29, 66.80, 67.75, 59.91, 59.28, 59.24, 57.45, 53.53, 37.07, 35.85, 34.62, 17.61, 21.81, 27.27. HRMS (ESI) Anal. Calcd for $C_{31}H_{50}N_4O_{29}S_2^{2-}$ $[M-2H]^{2-}$: 503.1006, found 503.1011.

Compound **42:** The solution of compound **33** (15 mg, 7.6 µmol) in DMF was subjected to *O*-sulfation followed by silyl-deprotection, saponification, *N*-acylation and hydrogenation according to the general procedures to afford compound **42** (4.2 mg, 50% yield over 5 steps). $[\alpha]_D^{20}$ = +1.9° (*c* = 0.1, H_2O); ^{1}H NMR (500 MHz, D_2O) δ 5.24 (d, *J* = 3.3 Hz, 1H), 4.98 (d, *J* = 3.1 Hz, 1H), 4.92 (s, 1H), 4.56 (s, 1H), 4.36 (s, 1H), 4.23 – 4.14 (m, 1H), 4.12 – 3.76 (m, 9H), 3.77 – 3.60 (m, 8H), 3.59 – 3.52 (m, 2H), 3.51 – 3.43 (m, 1H), 3.30 (t, *J* = 9.4 Hz, 1H), 3.18 (t, *J* = 6.4 Hz, 1H), 2.83 (t, *J* = 7.6 Hz, 1H), 2.57 (t, *J* = 6.5 Hz, 1H), 1.87 (s, 5H), 1.73 (dt, *J* = 13.2, 6.6 Hz, 1H); ^{13}C NMR (125 MHz, D_2O) δ 100.42, 93.87, 96.85, 99.45, 80.04, 79.51, 75.58, 75.48, 74.99, 74.51, 72.36, 71.78, 70.59, 70.54, 69.49, 66.58, 65.62, 66.15, 59.89, 59.36, 59.36, 53.50, 52.92, 36.89, 35.62, 26.45, 21.77, 17.42; HRMS (ESI) Anal. Calcd for $C_{33}H_{52}N_4O_{30}S_2^{2-}$ $[M-2H]^{2-}$: 524.1059, found 524.1068.

Compound **43:** The solution of compound **33** (8 mg, 4 µmol) in DMF was subjected to *O*-sulfation followed by silyl-deprotection, saponification, *N*-sulfation and hydrogenation according to the general procedures to afford compound **43** (1.6 mg, 37% yield over 5 steps). $[\alpha]_D^{20}$ = +2.5° (*c* = 0.05, H_2O); ^{1}H NMR (500 MHz, D_2O) δ 5.34 (d, *J* = 3.5 Hz, 1H), 5.25 (d, *J* = 3.4 Hz, 1H), 4.89 (d, *J* = 10.2 Hz, 1H), 4.55 (d, *J* = 7.7 Hz, 1H), 4.35 (dd, *J* = 5.6, 3.7 Hz, 1H), 4.20 (dd, *J* = 7.9, 4.5 Hz, 1H), 4.13 – 3.91 (m, 6H), 3.84 – 3.74 (m, 3H), 3.74 – 3.65 (m, 6H), 3.63 (s, 3H), 3.57 – 3.51 (m, 2H), 3.50 – 3.43 (m, 1H), 3.31 (t, *J* = 9.6 Hz, 1H), 3.08 (dd, *J* = 10.4, 3.3 Hz, 1H), 2.84 (t, *J* =

7.8 Hz, 1H), 2.58 (t, *J* = 6.6 Hz, 1H), 1.88 (s, 3H), 1.80 – 1.68 (m, 1H); ^{13}C NMR (125 MHz, D_2O) ^{13}C NMR (125 MHz, D_2O) δ 100.27, 99.91, 96.82, 95.75, 80.25, 79.49, 78.12, 76.39, 75.78, 75.56, 70.86, 70.48, 70.32, 70.31, , 69.60, 68.90, 66.45, 59.85, 59.46, 59.42, 59.19, 57.61, 53.50, 37.02, 26.72. 21.86; HRMS (ESI) Anal. Calcd for $C_{31}H_{49}N_4O_{32}S_3^{3-}$ [M-3H]$^{3-}$: 361.7169, found 361.7166.

Compound **44:** The solution of compound **34** (20.7 mg, 11.2 µmol) in pyridine was subjected to *O*-sulfation followed by silyl-deprotection, saponification, *N*-acylation and hydrogenation according to the general procedures to afford compound **44** (7.1 mg 61% over 5 steps). $[\boldsymbol{\alpha}]_{\mathbf{D}}^{\mathbf{20}}$ = +2.4° (*c* = 0.2, H_2O); ^{1}H NMR (500 MHz, D_2O) δ 5.24 (d, *J* = 3.7 Hz, 1H), 5.00 (d, *J* = 3.7 Hz, 1H), 4.71 (d, *J* = 3.2 Hz, 1H), 4.57 (d, *J* = 7.8 Hz, 1H), 4.38 (d, *J* = 10.9 Hz, 1H), 4.35 (d, *J* = 2.7 Hz, 1H), 4.03 (dd, *J* = 19.0, 5.8 Hz, 3H), 3.95 (d, *J* = 8.5 Hz, 1H), 3.92 – 3.89 (m, 1H), 3.86 – 3.78 (m, 2H), 3.77 – 3.64 (m, 6H), 3.64 – 3.59 (m, 2H), 3.58 – 3.50 (m, 3H), 3.33 – 3.27 (m, 1H), 3.24 – 3.16 (m, 2H), 3.14 – 3.07 (m, 1H), 2.81 (t, *J* = 7.7 Hz, 1H), 2.62 – 2.51 (m, 1H), 2.27 – 2.20 (m, 1H), 1.87 (s, 3H), 1.83 (s, 3H), 1.72 – 1.65 (m, 1H); ^{13}C NMR (125 MHz, D_2O) δ 101.64, 99.78, 96.89, 94.71, 79.91, 76.26, 75.58, 75.22, 74.92, 71.72, 69.54, 69.51, 69.43, 69.27, 68.95, 66.88, 65.63, 52.96, 53.50, 59.91, 36.93, 36.23, 35.63, 31.04, 23.24, 21.78, 21.78; HRMS (ESI) Anal. Calcd for $C_{33}H_{52}N_4O_{30}S_2^{2-}$- [M]$^{2-}$: 524.1059 , found 524.1068.

Compound **45:** The solution of compound **34** (16.6 mg, 9 µmol) in pyridine was subjected to *O*-sulfation followed by silyl-deprotection, saponification, *N*-sulfation and hydrogenation according to the general procedures to afford compound **45** (4.9 mg 50% over 5 steps). $[\boldsymbol{\alpha}]_{\mathbf{D}}^{\mathbf{20}}$ = +5.0° (*c* = 0.1, H_2O); ^{1}H NMR (500 MHz, D_2O) δ 5.24 (d, *J* = 3.7 Hz, 1H), 5.19 (d, *J* = 3.7 Hz, 1H), 4.74 (d, *J* = 2.8 Hz, 1H), 4.58 (d, *J* = 7.9 Hz, 1H), 4.37 (d, *J* = 11.1 Hz, 1H), 4.33 (d, *J* = 2.5 Hz, 1H), 4.09 – 3.97 (m, 4H), 3.97 – 3.89 (m, 2H), 3.83 – 3.65 (m, 6H), 3.63 (d, *J* = 2.6 Hz, 2H), 3.61 – 3.52 (m, 5H), 3.30 (t, *J* = 9.6 Hz, 1H), 3.20 (dd, *J* = 13.9, 7.0 Hz, 1H), 3.12 (ddd, *J* = 11.1, 10.1, 5.2 Hz, 2H), 2.81 (t, *J* = 7.8 Hz, 2H), 1.88 (s, 3H), 1.74 – 1.67 (m, 1H); ^{13}C NMR (125 MHz, D_2O) δ 101.50, 99.73, 96.85, 95.82, 79.95, 77.35, 76.04, 75.34, 75.33, 71.72, 69.58, 69.10, 68.93, 68.82, 68.69, 68.45, 66.86, 65.68, 65.67, 59.92, 57.32, 53.53, 36.98, 35.69, 35.68, 26.72, 21.79; HRMS (ESI) Anal. Calcd for $C_{31}H_{49}N_4O_{32}S_3^{3-}$ [M-3H]$^{3-}$: 361.7169, found 361.7169.

Compound **46:** The solution of compound **35** (9 mg, 4.9 µmol) in DMF was subjected for *O*-sulfation followed by saponification, *N*-acylation and hydrogenation according to the general procedures to afford compound **46** (3.0 mg 58% over 4 steps). $[\boldsymbol{\alpha}]_{\mathbf{D}}^{\mathbf{20}}$ = +2.6° (*c* = 0.1, H_2O); ^{1}H NMR (500 MHz, D_2O) δ 5.24 (d, *J* = 3.7 Hz, 1H), 5.00 (d, *J* = 3.6 Hz, 1H), 4.70 (d, *J* = 3.6 Hz, 1H), 4.54 (d, *J* = 7.8 Hz, 1H), 4.35 (d, *J* = 2.9 Hz, 1H), 4.20 (d, *J* = 9.8 Hz, 1H), 4.06 (q, *J* = 15.8 Hz, 2H), 3.97 (dd, *J* = 18.1, 9.8 Hz, 2H), 3.91 – 3.88 (m, 1H), 3.82 (d, *J* = 9.7 Hz, 1H), 3.79 – 3.66 (m, 10H), 3.59 – 3.52 (m, 3H), 3.51 – 3.46 (m, 1H), 3.42 (t, *J* = 9.6 Hz, 1H), 3.23 – 3.17 (m, 1H), 3.15 – 3.09 (m, 1H), 2.81 (t, *J* = 7.7 Hz, 2H), 1.87 (s, 3H), 1.84 (s, 3H), 1.75 – 1.66 (m, 1H); ^{13}C NMR (125 MHz, D_2O) δ 101.50, 100.37, 97.10, 94.60, 80.00, 79.37, 75.75, 75.52, 74.91, 70.67,

69.77, 69.77, 69.57, 68.87, 66.97, 66.22, 66.16, 59.33, 59.24, 53.28, 37.04, 35.76, 35.73, 26.76, 21.93, 21.82; HRMS (ESI) Anal. Calcd for $C_{33}H_{52}N_4O_{30}S_2^{2-}$ $[M-2H]^{2-}$: 524.1059, found 524.1062.

Compound 47: The solution of compound **35** (17 mg, 9.9 μmol) in DMF was subjected to *O*-sulfation followed by saponification, *N*-sulfation and hydrogenation according to the general procedures to afford compound **47** (4.4 mg, 41% over 4 steps). $[\alpha]_D^{20}$ = +3.8° (*c* = 0.1, H_2O); ^{1}H NMR (500 MHz, D_2O) δ 5.25 (d, *J* = 3.7 Hz, 1H), 5.18 (d, *J* = 3.6 Hz, 1H), 4.74 (d, *J* = 2.8 Hz, 1H), 4.54 (d, *J* = 7.8 Hz, 1H), 4.33 (d, *J* = 2.3 Hz, 1H), 4.20 (d, *J* = 9.8 Hz, 1H), 4.07 (t, *J* = 11.3 Hz, 2H), 4.02 – 3.93 (m, 3H), 3.91 – 3.88 (m, 1H), 3.84 – 3.72 (m, 5H), 3.70 – 3.63 (m, 4H), 3.60 – 3.53 (m, 3H), 3.47 (t, *J* = 9.5 Hz, 1H), 3.42 (t, *J* = 9.6 Hz, 1H), 3.18 (qd, *J* = 14.0, 7.1 Hz, 2H), 3.06 (dd, *J* = 10.4, 3.7 Hz, 1H), 2.82 (t, *J* = 7.8 Hz, 2H), 1.87 (s, 3H), 1.71 (td, *J* = 13.6, 7.0 Hz, 1H); ^{13}C NMR (125 MHz, D_2O) δ 174.29, 172.54, 101.49, 100.40, 97.02, 95.76, 80.03, 79.28, 76.11, 75.75, 75.53, 75.30, 70.43, 69.96, 69.28, 69.01, 68.86, 68.81, 68.50, 66.91, 66.11, 59.28, 57.41, 53.37, 36.96, 35.69, 26.64, 21.78; HRMS (ESI) Anal. Calcd for $C_{31}H_{49}N_4O_{32}S_3^{3-}$ $[M-3H]^{3-}$: 361.7169, found 361.7195.

Compound **48:** The solution of compound **36** (10 mg, 5.7 μmol) in DMF was subjected for *O*-sulfation followed by silyl-deprotection, saponification, *N*-acylation and hydrogenation according to the general procedures to afford compound **48** (2.1 mg 32% over 5 steps). $[\alpha]_D^{20}$ = +2.5° (*c* = 0.1, H_2O); ^{1}H NMR (500 MHz, D_2O) δ 5.25 (d, *J* = 3.7 Hz, 1H), 5.01 (d, *J* = 3.8 Hz, 1H), 4.94 (d, *J* = 2.6 Hz, 1H), 4.58 (d, *J* = 7.8 Hz, 1H), 4.39 (dd, *J* = 10.7, 6.3 Hz, 2H), 4.18 (dd, *J* = 5.0, 2.7 Hz, 1H), 4.11 – 4.03 (m, 3H), 4.02 – 3.99 (m, 1H), 3.98 – 3.94 (m, 1H), 3.92 (t, *J* = 3.1 Hz, 1H), 3.85 (dd, *J* = 10.6, 3.6 Hz, 2H), 3.77 (t, *J* = 8.4 Hz, 1H), 3.74 – 3.63 (m, 6H), 3.60 – 3.54 (m, 3H), 3.31 (t, *J* = 9.6 Hz, 1H), 3.24 – 3.13 (m, 2H), 2.84 (t, *J* = 7.7 Hz, 2H), 1.89 (d, *J* = 1.5 Hz, 6H), 1.77 – 1.70 (m, 2H); ^{13}C NMR (125 MHz, D_2O) δ 99.66, 96.86, 99.48, 94.19, 80.02, 77.57, 75.98, 75.30, 75.07, 72.84, 71.70, 70.53, 69.54, 68.91, 68.90, 68.71, 66.58, 66.41, 65.72, 65.64, 59.87, 53.43, 52.77, 36.91, 35.62, 26.52, 21.87; HRMS (ESI) Anal. Calcd for $C_{33}H_{51}N_4O_{33}S_3^{3-}$ $[M]^{3-}$: 375.7205, found 375.7238.

Compound **49:** The solution of compound **36** (10 mg, 5.7 μmol) in DMF was subjected for *O*-sulfation followed by TBDPS-deprotection, saponification, *N*-sulfation and hydrogenation according to the general procedures to afford compound **49** (2.4 mg, 36% over 5 steps). $[\alpha]_D^{20}$ = +2.2° (*c* = 0.1, H_2O); ^{1}H NMR (500 MHz, D_2O) δ 5.34 (d, *J* = 3.6 Hz, 1H), 5.26 (d, *J* = 3.7 Hz, 1H), 4.92 (d, *J* = 4.1 Hz, 1H), 4.60 (d, *J* = 7.9 Hz, 1H), 4.39 (t, *J* = 7.1 Hz, 2H), 4.22 (dd, *J* = 7.8, 4.3 Hz, 1H), 4.13 – 3.95 (m, 6H), 3.87 (d, *J* = 9.3 Hz, 1H), 3.77 (t, *J* = 8.6 Hz, 1H), 3.75 – 3.67 (m, 3H), 3.67 – 3.62 (m, 2H), 3.62 – 3.50 (m, 5H), 3.36 – 3.29 (m, 1H), 3.23 – 3.11 (m, 3H), 2.81 (t, *J* = 7.6 Hz, 1H), 1.90 (s, 3H), 1.76 – 1.68 (m, 2H); ^{13}C NMR (125 MHz, D_2O) δ 99.94, 99.59, 96.80, 96.06, 79.94, 77.55, 77.37, 76.71, 75.99, 75.37, 71.76, 71.54, 70.48, 70.30, 69.59, 68.84, 68.77, 66.49, 66.22, 65.68, 59.89, 57.52, 57.35, 53.47, 37.02, 35.72, 27.06, 21.79; HRMS (ESI) Anal. Calcd for $C_{31}H_{49}N_4O_{35}S_4^{3-}$ $[M-3H]^{3-}$: 388.3692, found 388.3708.

Compound **50:** The solution of compound **37** (8.6 mg, 5.2 µmol) in DMF was subjected for *O*-sulfation followed by saponification, *N*-acylation and hydrogenation according to the general procedures to afford compound **50** (3.1 mg, 53% over 4 steps). $[\boldsymbol{\alpha}]_{\mathbf{D}}^{\mathbf{20}}$ = +2.9° (*c* = 0.1, H_2O); ^{1}H NMR (500 MHz, D_2O) δ 5.24 (d, *J* = 3.7 Hz, 1H), 4.99 (d, *J* = 3.7 Hz, 1H), 4.92 (d, *J* = 2.4 Hz, 1H), 4.54 (d, *J* = 7.8 Hz, 1H), 4.36 (d, *J* = 2.6 Hz, 1H), 4.23 – 4.15 (m, 2H), 4.06 (d, *J* = 3.1 Hz, 2H), 4.03 – 3.94 (m, 3H), 3.92 – 3.88 (m, 1H), 3.83 – 3.72 (m, 6H), 3.71 – 3.64 (m, 4H), 3.59 – 3.54 (m, 1H), 3.48 (t, *J* = 9.4 Hz, 1H), 3.42 (t, *J* = 9.6 Hz, 1H), 3.18 (t, *J* = 6.5 Hz, 2H), 2.83 (t, *J* = 7.7 Hz, 2H), 1.87 (s, 6H), 1.77 – 1.69 (m, 2H); ^{13}C NMR (125 MHz, D_2O) δ 100.43, 99.46, 97.07, 93.89, 80.05, 79.48, 75.91, 75.48, 75.11, 72.47, 70.76, 70.45, 70.01, 69.94, 69.19, 68.85, 68.79, 66.38, 66.61, 66.16, 66.12, 59.41, 59.38, 53.36, 53.36, 53.02, 53.02, 36.98, 35.67, 26.63, 21.91; HRMS (ESI) Anal. Calcd for $C_{33}H_{51}N_4O_{33}S_3^{3-}$ $[M]^{3-}$: 375.7205, found 375.7205.

Compound **51:** The solution of compound **37** (9.4 mg, 5.7 µmol) in DMF was subjected to *O*-sulfation followed by saponification, *N*-sulfation and hydrogenation according to the general procedures to afford compound **48** (5.2 mg 42% over 4 steps). $[\boldsymbol{\alpha}]_{\mathbf{D}}^{\mathbf{20}}$ = +2.1° (*c* = 0.1, H_2O); ^{1}H NMR (500 MHz, D_2O) δ 5.33 (d, *J* = 3.3 Hz, 1H), 5.25 (d, *J* = 3.7 Hz, 1H), 4.89 (d, *J* = 4.4 Hz, 1H), 4.54 (d, *J* = 7.9 Hz, 1H), 4.36 (d, *J* = 2.9 Hz, 1H), 4.20 (dd, *J* = 7.9, 3.9 Hz, 2H), 4.12 – 3.94 (m, 6H), 3.82 – 3.66 (m, 8H), 3.55 (dd, *J* = 21.2, 10.4 Hz, 2H), 3.44 (dt, *J* = 22.6, 9.6 Hz, 2H), 3.24 – 3.12 (m, 2H), 3.08 (dd, *J* = 10.4, 3.6 Hz, 1H), 2.87 (ddd, *J* = 15.4, 11.1, 6.5 Hz, 2H), 1.88 (s, 3H), 1.80 – 1.70 (m, 2H); ^{13}C NMR (125 MHz, D_2O) δ 100.35, 100.01, 97.03, 95.67, 80.07, 79.47, 77.84, 76.38, 75.96, 75.57, 70.76, 70.54, 70.53, 70.45, 70.03, 69.95, 69.08, 68.89, 66.59, 66.16, 66.14, 59.46, 59.45, 57.56, 53.40, 46.09, 37.01, 35.67, 26.55, 21.82; HRMS (ESI) Anal. Calcd for $C_{31}H_{48}N_4O_{35}S_4^{4-}$ $[M]^{4-}$: 291.0251, found 291.0245.

Compound **52:** The solution of compound **38** (9.5 mg, 6.2 µmol) in DMF was subjected for *O*-sulfation followed by saponification, *N*-acylation and hydrogenation according to general procedure to afford compound **52** (2.5 mg, 40% over 4 steps). $[\boldsymbol{\alpha}]_{\mathbf{D}}^{\mathbf{20}}$ = +1.2° (*c* = 0.05, H_2O); ^{1}H NMR (500 MHz, D_2O) δ 5.25 (d, *J* = 3.7 Hz, 1H), 5.01 (d, *J* = 3.8 Hz, 1H), 4.93 (d, *J* = 2.6 Hz, 1H), 4.58 (d, *J* = 7.9 Hz, 1H), 4.39 (dd, *J* = 13.1, 6.3 Hz, 2H), 4.25 – 4.16 (m, 2H), 4.11 – 4.03 (m, 3H), 4.02 – 3.94 (m, 3H), 3.92 (t, *J* = 3.1 Hz, 1H), 3.85 (dd, *J* = 10.6, 3.7 Hz, 2H), 3.79 – 3.72 (m, 3H), 3.67 (dt, *J* = 10.6, 6.8 Hz, 3H), 3.60 – 3.54 (m, 2H), 3.42 (t, *J* = 9.6 Hz, 1H), 3.24 – 3.14 (m, 2H), 2.82 (dd, *J* = 9.9, 5.5 Hz, 2H), 1.89 (s, 6H), 1.77 – 1.70 (m, 2H); ^{13}C NMR (125 MHz, D_2O) δ 99.83, 99.48, 97.05, 94.25, 79.91, 77.73, 76.07, 75.43, 75.13, 72.98, 70.40, 70.01, 69.95, 68.97, 68.95, 68.87, 68.81, 66.79, 66.59, 66.01, 65.55, 52.82, 52.78, 53.28, 36.99, 35.68, 26.71, 21.86; HRMS (ESI) Anal. Calcd for $C_{33}H_{50}N_4O_{36}S_4^{4-}$ $[M]^{4-}$: 301.5277, found 301.5290.

Compound **53:** The solution of compound **38** (10.5 mg, 6.9 µmol) in DMF was subjected for *O*-sulfation followed by saponification, *N*-sulfation and hydrogenation according to the general procedures to afford compound **53** (3.0 mg, 36% over 4 steps). $[\boldsymbol{\alpha}]_{\mathbf{D}}^{\mathbf{20}}$ = +3.9° (*c* = 0.1, H_2O); ^{1}H

NMR (500 MHz, D_2O) δ 5.33 (d, *J* = 3.6 Hz, 1H), 5.25 (d, *J* = 3.7 Hz, 1H), 4.90 (d, *J* = 4.2 Hz, 1H), 4.59 (d, *J* = 7.9 Hz, 1H), 4.42 – 4.34 (m, 2H), 4.20 (dd, *J* = 7.9, 4.1 Hz, 2H), 4.13 – 3.93 (m, 7H), 3.86 (d, *J* = 9.0 Hz, 1H), 3.79 – 3.72 (m, 3H), 3.71 – 3.65 (m, 2H), 3.60 – 3.51 (m, 3H), 3.42 (t, *J* = 9.7 Hz, 1H), 3.18 (dd, *J* = 13.3, 6.6 Hz, 2H), 3.15 – 3.10 (m, 1H), 2.84 (t, *J* = 7.7 Hz, 2H), 1.88 (s, 3H), 1.73 (td, *J* = 13.7, 7.1 Hz, 2H); ^{13}C NMR (125 MHz, D_2O) δ 100.06, 99.76, 97.05, 96.12, 79.98, 77.58, 77.40, 76.75, 76.11, 75.38, 70.60, 70.42, 70.35, 69.96, 68.96, 68.89, 68.83, 66.66, 66.50, 66.17, 65.64, 57.44, 53.41, 36.99, 35.70, 26.57, 21.85; HRMS (ESI) Anal. Calcd for $C_{31}H_{47}N_4O_{38}S_5Na_2^{3-}$ [M+2Na]$^{-3}$: 429.6761, found 429.6767.

Compound **54:** The solution of compound **39** (12.5 mg, 7.7 µmol) in DMF was subjected for *O*-sulfation followed by saponification, *N*-acylation and hydrogenation according to the general procedures to afford compound **54** (3.2 mg, 36% over 4 steps). $[\boldsymbol{\alpha}]_{\mathbf{D}}^{\mathbf{20}}$ = +5.1° (*c* = 0.1, H_2O); ^{1}H NMR (500 MHz, D_2O) δ 5.24 (d, *J* = 3.7 Hz, 1H), 5.02 (d, *J* = 3.6 Hz, 1H), 4.70 (d, *J* = 3.4 Hz, 1H), 4.57 (d, *J* = 7.8 Hz, 1H), 4.39 (d, *J* = 10.8 Hz, 1H), 4.35 (dd, *J* = 5.1, 2.8 Hz, 1H), 4.22 – 4.18 (m, 1H), 4.11 – 3.93 (m, 5H), 3.91 (d, *J* = 3.1 Hz, 1H), 3.84 (d, *J* = 10.1 Hz, 1H), 3.80 (dd, *J* = 10.8, 3.7 Hz, 1H), 3.77 – 3.63 (m, 7H), 3.58 – 3.52 (m, 3H), 3.41 (t, *J* = 9.6 Hz, 1H), 3.26 – 3.18 (m, 1H), 3.14 – 3.07 (m, 1H), 2.87 – 2.78 (m, 2H), 1.88 (s, 3H), 1.84 (s, 3H), 1.78 – 1.64 (m, 2H); ^{13}C NMR (125 MHz, D_2O) δ 101.77, 99.80, 97.08, 94.75, 79.90, 76.07, 77.50, 75.06, 75.32, 70.45, 69.87, 69.75, 69.54, 68.98, 68.85, 68.80, 68.79, 66.93, 66.13, 65.97, 65.66, 53.40, 53.00, 45.72, 36.96, 35.71, 35.64, 26.16, 25.52, 21.83, 21.78; HRMS (ESI) Anal. Calcd for $C_{33}H_{51}N_4O_{33}S_3^{3-}$ [M$^-$]: 1127.1614, found1127.1665.

Compound **55:** The solution of compound **39** (12.5 mg, 7.7 µmol) in DMF was subjected to *O*-sulfation followed by saponification, *N*-sulfation and hydrogenation according to the general procedures to afford compound **55** (3.0 mg, 34% over 4 steps). $[\boldsymbol{\alpha}]_{\mathbf{D}}^{\mathbf{20}}$ = +4.6° (*c* = 0.1, H_2O); ^{1}H NMR (500 MHz, D_2O) δ 5.25 (d, *J* = 3.8 Hz, 1H), 5.19 (d, *J* = 3.7 Hz, 1H), 4.74 (d, *J* = 2.9 Hz, 1H), 4.57 (d, *J* = 7.9 Hz, 1H), 4.37 (d, *J* = 10.2 Hz, 1H), 4.33 (d, *J* = 2.6 Hz, 1H), 4.20 (dd, *J* = 11.1, 1.9 Hz, 1H), 4.05 (d, *J* = 6.9 Hz, 2H), 4.00 (dd, *J* = 10.5, 7.2 Hz, 3H), 3.97 – 3.92 (m, 1H), 3.92 – 3.90 (m, 1H), 3.82 – 3.72 (m, 4H), 3.70 – 3.64 (m, 2H), 3.61 – 3.51 (m, 4H), 3.41 (t, *J* = 9.7 Hz, 1H), 3.23 – 3.09 (m, 3H), 2.81 (t, *J* = 7.8 Hz, 2H), 1.88 (s, 3H), 1.76 – 1.65 (m, 2H); ^{13}C NMR (125 MHz, D_2O) δ 101.52, 99.74, 97.05, 95.91, 79.92, 77.39, 76.25, 75.50, 75.39, 69.90, 69.06, 69.00, 68.81, 68.74, 68.50, 66.92, 66.14, 66.13, 57.29, 36.97, 35.71, 35.70, 26.67, 26.66, 21.77; HRMS (ESI) Anal. Calcd for $C_{31}H_{49}N_4O_{35}S_4^{3-}$ [M-3H]$^{3-}$: 388.3692, found 388.3689.

Scheme S7. Syntheses of tetrasaccharides **56** - **71**.

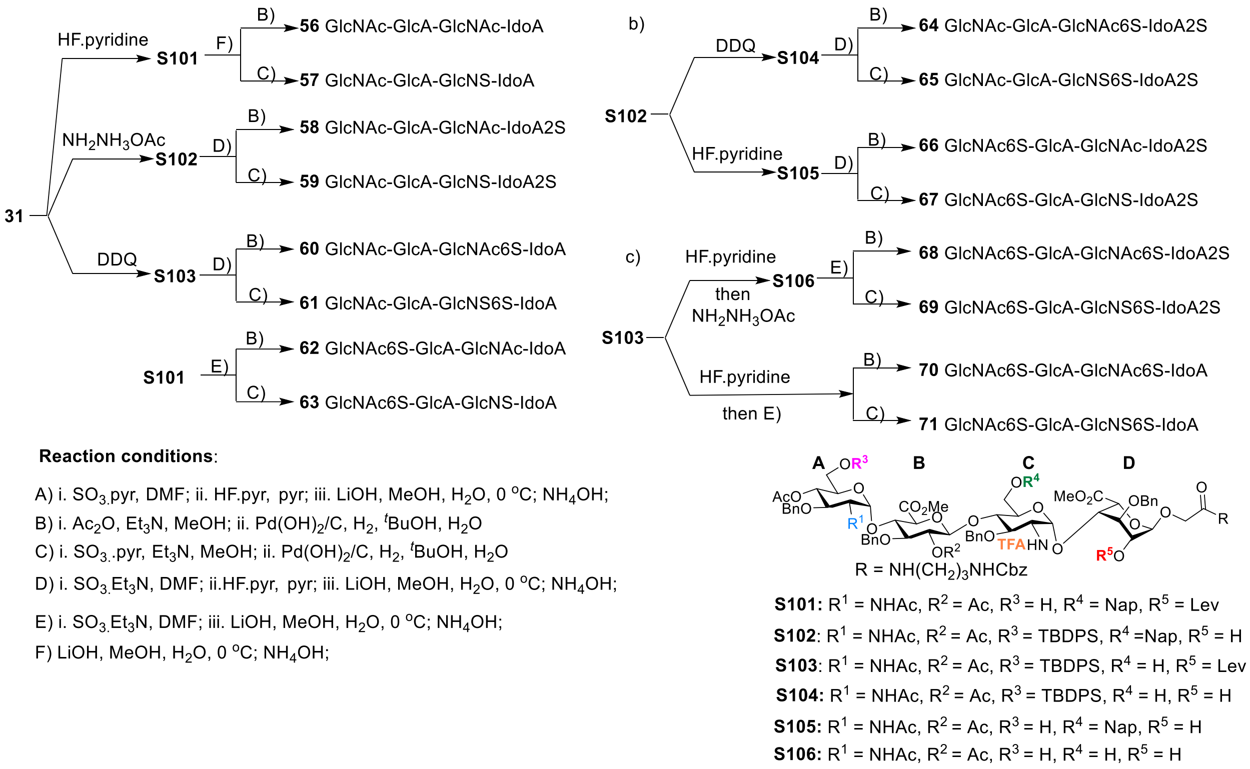

Compound **56:** To the solution of compound **S101** (14 mg, 7.8 µmol) in methanol and water was subjected for saponification followed by *N*-acylation and hydrogenation according to the general procedures to afford compound **56** (3.2 mg, 46% over 3 steps). $[\alpha]_D^{20}$ = +4.8° (*c* = 0.1, H_2O); ^{1}H NMR (500 MHz, D_2O) δ 5.25 (d, *J* = 3.8 Hz, 1H), 4.99 (d, *J* = 3.7 Hz, 1H), 4.73 (d, *J* = 3.0 Hz, 1H), 4.34 (dd, *J* = 7.9, 5.3 Hz, 2H), 4.10 – 4.01 (m, 2H), 3.89 (t, *J* = 3.1 Hz, 1H), 3.78 – 3.68 (m, 6H), 3.67 – 3.62 (m, 4H), 3.61 – 3.50 (m, 6H), 3.31 (t, *J* = 9.6 Hz, 1H), 3.23 – 3.10 (m, 3H), 2.76 (t, *J* = 7.5 Hz, 2H), 1.88 (s, 3H), 1.84 (s, 3H), 1.72 – 1.64 (m, 2H); ^{13}C NMR (125 MHz, D_2O) δ 102.22, 101.29, 96.78, 94.19, 76.39, 76.14, 73.87, 73.34, 71.78, 70.59, 70.57, 69.53, 69.35, 69.11, 68.94, 68.67, 66.60, 59.94, 53.55, 53.54, 53.01, 37.03, 35.76, 27.17,21.82, 21.80; HRMS (ESI) Anal. Calcd for $C_{33}H_{53}N_4O_{24}^-$ [M-H]$^-$: 889.3055, found 889.3011.

Compound 57: The solution of compound **S101** (16 mg, 8.9 µmol) in methanol and water was subjected to saponification followed by *N*-sulfation and hydrogenation according to the general procedures to afford compound **57** (4.6 mg, 52% over 3 steps). $[\alpha]_D^{20}$ = +0.4° (*c* = 0.05, H_2O); ^{1}H NMR (500 MHz, D_2O) δ 5.25 (d, *J* = 3.5 Hz, 1H), 5.18 (d, *J* = 3.4 Hz, 1H), 4.75 (d, *J* = 2.5 Hz, 1H), 4.32 (dd, *J* = 14.9, 12.0 Hz, 2H), 4.09 – 4.01 (m, 2H), 4.00 – 3.97 (m, 1H), 3.90 – 3.88 (m, 1H), 3.74 – 3.60 (m, 8H), 3.60 – 3.50 (m, 6H), 3.31 (t, *J* = 9.7 Hz, 1H), 3.24 – 3.14 (m, 3H), 3.08 – 3.05 (m, 1H), 2.83 (t, *J* = 7.7 Hz, 2H), 1.88 (s, 3H), 1.75 – 1.69 (m, 2H); ^{13}C NMR (125 MHz, D_2O) δ 102.53, 101.07, 96.73, 95.36, 76.43, 76.11, 75.71, 74.41, 73.35, 71.73, 70.59, 69.60, 68.66,

68.46, 67.86, 66.47, 59.93, 59.29, 59.26, 57.35, 53.56, 36.89, 35.63, 26.71, 21.81; HRMS (ESI) Anal. Calcd for $C_{31}H_{51}N_4O_{26}S^-$ $[M]^-$: 927.2518, found 927.2546.

Compound **58:** The solution of compound **S102** (12 mg, 6.2 µmol) in DMF was subjected to *O*-sulfation followed by silyl-group deprotection, saponification, *N*-acylation and hydrogenation according to the general procedures to afford compound **58** (2.5 mg, 38% over 5 steps). $[\alpha]_D^{20}$ = -0.4° (c = 0.1, H_2O); ^1H NMR (500 MHz, D_2o) δ 5.25 (d, *J* = 3.8 Hz, 1H), 4.98 (s, 1H), 4.94 (d, *J* = 2.4 Hz, 1H), 4.36 (d, *J* = 2.6 Hz, 1H), 4.32 (d, *J* = 7.9 Hz, 1H), 4.18 (dd, *J* = 4.5, 2.4 Hz, 1H), 4.06 (s, 2H), 4.02 (dt, *J* = 5.5, 2.6 Hz, 1H), 3.90 (d, *J* = 3.0 Hz, 1H), 3.81 (dd, *J* = 10.6, 3.6 Hz, 1H), 3.76 – 3.69 (m, 4H), 3.66 – 3.49 (m, 10H), 3.30 (t, *J* = 9.6 Hz, 1H), 3.24 – 3.13 (m, 3H), 2.93 – 2.79 (m, 3H), 2.64 (s, 1H), 2.54 (s, 1H), 1.88 (s, 6H), 1.79 – 1.71 (m, 2H); ^{13}C NMR (125 MHz, D_2o) δ 102.10, 99.17, 96.83, 93.70, 78.08, 74.56, 73.39, 71.83, 71.63, 70.66, 69.48, 68.31, 65.77, 55.23, 53.47, 52.88, 46.24, 42.73, 36.87, 35.70, 31.63, 25.35, 22.03; HRMS (ESI) Anal. Calcd for $C_{33}H_{54}N_4O_{27}S$ $[M-H]^-$: 969.2623, found 969.2611.

Compound **59:** The solution of compound **S102** (32 mg, 16.5 µmol) in DMF was subjected to *O*-sulfation followed by silyl-group deprotection, saponification, *N*-sulfation and hydrogenation according to the general procedures to afford compound **59** (6.5 mg, 36% over 5 steps). $[\alpha]_D^{20}$ = +4.1° (c = 0.1, H_2O); ^1H NMR (500 MHz, D_2O) δ 5.34 (s, 1H), 5.25 (d, *J* = 3.8 Hz, 1H), 4.90 (d, *J* = 4.4 Hz, 1H), 4.36 (d, *J* = 3.1 Hz, 1H), 4.33 (d, *J* = 7.9 Hz, 1H), 4.09 – 3.96 (m, 5H), 3.78 – 3.68 (m, 5H), 3.66 – 3.46 (m, 12H), 3.31 (t, *J* = 9.6 Hz, 2H), 3.26 – 3.07 (m, 6H), 3.00 – 2.83 (m, 4H), 2.70 (d, *J* = 6.2 Hz, 2H), 1.88 (s, 3H), 1.80 – 1.72 (m, 2H); ^{13}C NMR (125 MHz, D_2o) δ 102.10, 99.76, 96.63, 95.46, 70.46, 77.49, 76.13, 75.93, 73.39, 71.63, 70.46, 69.68, 66.36, 57.38, 53.47, 36.87, 35.70, 35.50, 42.73, 26.52, 21.83; HRMS (ESI) Anal. Calcd for $C_{31}H_{52}N_4O_{29}S_2$ $[M-H]^-$: 1007.2086, found 1007.2076.

Compound **60:** The solution of compound **S103** (25 mg, 13 µmol) in DMF was subjected to *O*-sulfation, followed by silyl-group deprotection, saponification, *N*-acylation and hydrogenation according to the general procedures to afford compound **60** (7.0 mg, 55% over 5 steps). $[\boldsymbol{\alpha}]_{\mathbf{D}}^{\mathbf{20}}$ = +5.3° (*c* = 0.1, H_2O); ^1H NMR (500 MHz, D_2O) δ 5.24 (d, *J* = 3.7 Hz, 1H), 5.00 (d, *J* = 3.4 Hz, 1H), 4.74 (d, *J* = 2.4 Hz, 1H), 4.40 (d, *J* = 7.9 Hz, 1H), 4.35 (d, *J* = 2.0 Hz, 1H), 4.30 – 4.25 (m, 1H), 4.11 – 4.02 (m, 3H), 3.94 – 3.89 (m, 2H), 3.78 (ddd, *J* = 8.8, 7.8, 3.4 Hz, 2H), 3.71 (dd, *J* = 10.7, 3.7 Hz, 1H), 3.67 – 3.60 (m, 5H), 3.56 (ddd, *J* = 12.9, 8.8, 3.8 Hz, 5H), 3.30 (t, *J* = 9.6 Hz, 1H), 3.27 – 3.10 (m, 3H), 2.90 – 2.81 (m, 2H), 1.88 (s, 3H), 1.83 (s, 3H), 1.78 – 1.71 (m, 2H). ^{13}C NMR (125 MHz, D_2O) δ 101.88, 101.27, 96.78, 94.11, 76.38, 75.89, 75.35, 73.79, 73.40, 71.82, 70.92, 69.58, 69.22, 69.12, 68.93, 68.74, 68.64, 66.50, 65.73, 59.95, 57.48, 54.75, 53.58, 53.58, 52.85, 52.82, 45.98, 35.67, 35.65, 25.49, 21.83, 21.80; HRMS (ESI) Anal. Calcd for $C_{33}H_{53}N_4O_{27}S^-$ $[M-H]^-$: 969.2623, found 969.2607.

Compound **61:** The solution of compound **S103** (20 mg, 10.4 µmol) in DMF was subjected to *O*-sulfation, followed by silyl-group deprotection, saponification, *N*-sulfation and hydrogenation

according to the general procedures to afford compound **61** (3.7 mg, 36% over 5 steps). $[\alpha]_D^{20}$ = +2.8° (*c* = 0.1, H_2O); ^1H NMR (500 MHz, D_2O) δ 5.25 (d, *J* = 3.7 Hz, 1H), 5.20 (d, *J* = 3.4 Hz, 1H), 4.75 (d, *J* = 2.3 Hz, 1H), 4.41 (d, *J* = 7.9 Hz, 1H), 4.33 (s, 1H), 4.29 (d, *J* = 9.4 Hz, 1H), 4.13 – 3.96 (m, 4H), 3.91 (s, 1H), 3.86 (d, *J* = 9.8 Hz, 1H), 3.71 (dd, *J* = 10.7, 3.7 Hz, 1H), 3.63 (q, *J* = 9.9 Hz, 5H), 3.54 (ddd, *J* = 24.7, 15.3, 6.0 Hz, 6H), 3.30 (t, *J* = 9.6 Hz, 1H), 3.16 (t, *J* = 8.4 Hz, 2H), 3.10 (dd, *J* = 10.3, 3.4 Hz, 1H), 2.82 (t, *J* = 7.6 Hz, 2H), 1.88 (s, 3H), 1.77 – 1.66 (m, 2H); ^{13}C NMR (125 MHz, D_2O) δ 101.77, 101.08, 96.73, 95.25, 76.42, 75.97, 74.45, 73.43, 71.61, 69.58, 69.42, 68.64, 68.60, 68.43, 67.90, 66.51, 65.72, 59.91, 57.15, 53.56, 36.89, 35.60, 35.60, 26.70, 21.78; HRMS (ESI) Anal. Calcd for $C_{31}H_{50}N_4O_{29}S_2^{2-}$ $[M]^{2-}$: 1006.2013, found 1006.2048.

Compound **62:** The solution of compound **S101** (16 mg, 8.9 µmol) in DMF was subjected to *O*-sulfation followed by saponification, *N*-acylation and hydrogenation according to the general procedures to afford compound **62** (5.0 mg, 58% over 4 steps). $[\alpha]_D^{20}$ = +6.3° (*c* = 0.1, H_2O); ^1H NMR (500 MHz, D_2O) δ 5.27 (d, *J* = 3.7 Hz, 1H), 4.99 (dd, *J* = 6.8, 3.7 Hz, 1H), 4.74 (dd, *J* = 10.0, 2.9 Hz, 1H), 4.36 – 4.32 (m, 2H), 4.20 (d, *J* = 11.0 Hz, 1H), 4.11 – 4.02 (m, 2H), 4.02 – 3.98 (m, 1H), 3.90 (s, 1H), 3.80 – 3.71 (m, 6H), 3.69 – 3.61 (m, 4H), 3.61 – 3.50 (m, 5H), 3.42 (t, *J* = 9.7 Hz, 2H), 3.23 – 3.18 (m, 2H), 2.80 (t, *J* = 7.5 Hz, 1H), 1.88 (s, 3H), 1.84 (s, 3H), 1.75 – 1.66 (m, 2H);^{13}C NMR (125 MHz, D_2O) δ 174.79, 174.42, 174.28, 174.21, 172.40, 102.22, 101.31, 96.80, 94.22, 78.04, 76.28, 76.17, 75.67, 73.94, 73.33, 70.57, 70.46, 69.92, 69.30, 68.90, 68.74, 66.62, 66.16, 59.28, 53.40, 53.01, 36.99, 35.72, 34.52, 27.09, 21.80, 17.48. HRMS (ESI) Anal. Calcd for $C_{33}H_{53}N_4O_{27}S^-$ $[M]^-$: 969.2623, found 969.2653.

Compound **63:** The solution of compound **S101** (13 mg, 7.2 µmol) in DMF was subjected to *O*-sulfation followed by saponification, *N*-sulfation and hydrogenation according to the general procedures to afford compound **63** (3.0 mg, 40% over 4 steps). $[\alpha]_D^{20}$ = +5.4° (*c* = 0.1, H_2O); ^1H NMR (500 MHz, D_2O) δ 5.27 (d, *J* = 3.7 Hz, 1H), 5.19 (d, *J* = 3.6 Hz, 1H), 4.76 (d, *J* = 2.5 Hz, 1H), 4.34 (t, *J* = 4.9 Hz, 2H), 4.20 (dd, *J* = 11.1, 2.0 Hz, 1H), 4.06 (s, 2H), 4.03 – 3.97 (m, 2H), 3.92 – 3.87 (m, 1H), 3.78 – 3.71 (m, 3H), 3.71 – 3.50 (m, 9H), 3.42 (t, *J* = 9.6 Hz, 1H), 3.24 – 3.13 (m, 3H), 3.07 (dd, *J* = 10.0, 3.8 Hz, 1H), 2.83 (t, *J* = 7.6 Hz, 2H), 1.88 (s, 3H), 1.79 – 1.66 (m, 2H); ^{13}C NMR (125 MHz, D_2O) δ 102.12, 101.16, 96.79, 95.42, 77.88, 76.17, 76.12, 75.86,74.59, 73.29, 70.48, 70.41, 69.91, 69.55, 68.88, 68.65, 68.49, 67.98, 66.56, 66.31, 66.20, 66.16, 59.41, 59.37, 57.33, 53.43, 36.94, 35.68, 26.68, 21.83; HRMS (ESI) Anal. Calcd for $C_{31}H_{50}N_4O_{29}S_2^{2-}$ $[M]^{2-}$: 503.1006, found 503.1014.

Compound **64:** The solution of compound **S104** (5.0 mg, 2.5 µmol) in DMF was subjected to *O*-sulfation followed by silyl-group deprotection, saponification, *N*-acylation and hydrogenation according to the general procedures to afford compound **64** (1.0 mg, 31% over 5 steps). $[\alpha]_D^{20}$ = +1.8° (c = 0.1, H_2O); ^1H NMR (500 MHz, D_2O) δ 5.25 (d, *J* = 3.9 Hz, 1H), 4.98 (d, *J* = 3.6 Hz, 1H), 4.95 (s, 1H), 4.43 – 4.36 (m, 2H), 4.27 (d, *J* = 11.0 Hz, 1H), 4.19 (s, 1H), 4.11 – 4.02 (m, 5H), 3.95 – 3.90 (m, 2H), 3.87 – 3.82 (m, 1H), 3.71 (dd, *J* = 10.6, 3.9 Hz, 1H), 3.66 – 3.53 (m,

13H), 3.30 (t, *J* = 9.5 Hz, 1H), 3.21 – 3.17 (m, 10H), 2.83 (t, *J* = 7.7 Hz, 2H), 1.88 (s, 3H), 1.75 – 1.72 (m, 2H); ^{13}C NMR (151 MHz, D_2O) δ 101.90, 99.16, 96.90, 93.84, 78.53, 77.24, 76.60, 75.95, 74.50, 73.38, 71.93, 71.76, 70.80, 69.67, 69.51, 68.70, 66.45, 65.64, 60.32, 60.00, 55.33, 53.72, 52.75, 46.63, 36.96, 35.67, 26.49, 21.97; HRMS (ESI) Anal. Calcd for $C_{33}H_{54}N_4O_{30}S_2$ $[M-2H]^{2-}$: 524.1059, found 524.1047.

Compound **65:** The solution of compound **S104** (14.0 mg, 7.5 µmol) in DMF was subjected to *O*-sulfation followed by silyl-group deprotection, saponification, *N*-sulfation and hydrogenation according to the general procedures to afford compound **65** (3.0 mg, 36% over 5 steps). $[\alpha]_D^{20}$ = +3.5° (c = 0.1, H_2O); ^{1}H NMR (500 MHz, D_2O) δ 5.35 (d, *J* = 3.6 Hz, 1H), 5.25 (d, *J* = 3.8 Hz, 1H), 4.91 (d, *J* = 4.3 Hz, 1H), 4.42 (d, *J* = 7.9 Hz, 1H), 4.36 (d, *J* = 2.9 Hz, 1H), 4.28 (dd, *J* = 11.2, 2.5 Hz, 1H), 4.21 (dd, *J* = 7.5, 4.3 Hz, 1H), 4.11 – 4.02 (m, 3H), 4.02 – 3.92 (m, 3H), 3.71 (dd, *J* = 10.6, 3.8 Hz, 1H), 3.66 – 3.45 (m, 10H), 3.32 – 3.09 (m, 6H), 2.83 (t, *J* = 7.7 Hz, 2H), 1.88 (s, 3H), 1.78 – 1.70 (m, 2H); ^{13}C NMR (125 MHz, D_2o) δ 101.71, 99.76, 96.63, 95.46, 77.30, 76.13, 75.93, 73.39, 71.05, 70.66, 70.46, 66.36, 59.92, 57.18, 37.06, 35.70, 26.52, 21.83; HRMS (ESI) Anal. Calcd for $C_{31}H_{52}N_4O_{32}S_3$ $[M-2H]^{2-}$: 543.0790, found 543.0799.

Compound **66:** The solution of compound **S105** (7.0 mg, 3.6 µmol) in DMF was subjected to *O*-sulfation followed by saponification, *N*-acylation and hydrogenation according to the general procedures to afford compound **66** (1.5 mg, 40% over 4 steps). $[\alpha]_D^{20}$ = -0.6° (c = 0.1, H_2O); ^{1}H NMR (500 MHz, D_2O) δ 5.27 (d, *J* = 3.8 Hz, 1H), 4.98 (d, *J* = 3.5 Hz, 1H), 4.94 (s, 1H), 4.37 (s, 1H), 4.32 (d, *J* = 8.0 Hz, 1H), 4.25 – 4.17 (m, 2H), 4.06 (s, 1H), 4.04 – 3.97 (m, 2H), 3.92 (d, *J* = 21.1 Hz, 2H), 3.81 (dd, *J* = 10.9, 3.6 Hz, 1H), 3.78 – 3.70 (m, 5H), 3.57 (ddt, *J* = 43.0, 15.8, 9.1 Hz, 8H), 3.41 (t, *J* = 9.7 Hz, 2H), 3.24 – 3.15 (m, 3H), 2.85 (q, *J* = 9.3, 7.7 Hz, 2H), 1.88 (s, 6H), 1.78 – 1.72 (m, 2H); ^{13}C NMR (151 MHz, D_2O) δ 102.28, 99.38, 96.96, 93.90, 78.27, 76.34, 76.18, 74.89, 73.44, 72.15, 70.70, 69.57, 69.09, 68.60, 66.67, 66.35, 66.03, 59.58, 55.39, 53.62, 52.97, 37.02, 35.73, 26.55, 22.04; HRMS (ESI) Anal. Calcd for $C_{33}H_{54}N_4O_{30}S_2$ $[M-3H]^{3-}$: 349.0682, found 349.0677.

Compound **67:** The solution of compound **S105** (20.0 mg, 11.4 µmol) in DMF was subjected to *O*-sulfation followed by saponification, *N*-sulfation and hydrogenation according to the general procedures to afford compound **67** (5.8 mg, 47% over 4 steps). $[\alpha]_D^{20}$ = +3.3° (c = 0.1, H_2O); ^{1}H NMR (500 MHz, D_2O) δ 5.34 (d, *J* = 3.6 Hz, 1H), 5.27 (d, *J* = 3.8 Hz, 1H), 4.91 (d, *J* = 4.3 Hz, 1H), 4.36 (d, *J* = 3.3 Hz, 1H), 4.34 (d, *J* = 7.9 Hz, 1H), 4.21 (td, *J* = 7.9, 3.5 Hz, 2H), 4.07 (d, *J* = 6.1 Hz, 1H), 4.05 – 3.91 (m, 4H), 3.78 – 3.67 (m, 5H), 3.67 – 3.47 (m, 7H), 3.42 (t, *J* = 9.6 Hz, 2H), 3.28 – 3.07 (m, 5H), 2.86 (t, *J* = 7.7 Hz, 2H), 1.88 (s, 3H), 1.75 (p, *J* = 6.9 Hz, 2H); ^{13}C NMR (125 MHz, D_2O) δ 102.10, 99.76, 96.83, 95.56, 77.88, 77.39, 76.22, 75.93, 75.83, 73.29, 70.46, 70.27, 69.97, 69.39, 68.90, 66.16, 59.43, 57.47, 36.97, 35.70, 35.60, 53.47, 26.52, 21.83; HRMS (ESI) Anal. Calcd for $C_{31}H_{52}N_4O_{32}S_3$ $[M-2H]^{2-}$: 543.0790, found 543.0799.

Compound **68:** The solution of compound **S106** (5.6 mg, 3.6 µmol) in DMF was subjected to *O*-sulfation followed by saponification, *N*-acylation and hydrogenation according to the general procedures to afford compound **68** (2.0 mg, 51% over 4 steps). $[\alpha]_D^{20}$ = +2.8° (*c* = 0.1, H_2O); ^{1}H NMR (500 MHz, D_2O) δ 5.28 (d, *J* = 3.7 Hz, 1H), 4.99 (d, *J* = 3.6 Hz, 1H), 4.96 (d, *J* = 2.0 Hz, 1H), 4.41 (d, *J* = 7.9 Hz, 1H), 4.38 (d, *J* = 2.4 Hz, 1H), 4.28 (dd, *J* = 11.1, 2.7 Hz, 1H), 4.20 (dt, *J* = 5.7, 2.9 Hz, 2H), 4.13 – 4.03 (m, 4H), 4.00 (dd, *J* = 11.1, 1.8 Hz, 1H), 3.93 (dd, *J* = 9.6, 6.3 Hz, 2H), 3.85 (dd, *J* = 10.1, 3.5 Hz, 1H), 3.75 (dd, *J* = 10.6, 3.7 Hz, 2H), 3.66 – 3.54 (m, 6H), 3.42 (t, *J* = 9.6 Hz, 2H), 3.20 (dd, *J* = 10.6, 3.8 Hz, 3H), 2.85 – 2.78 (m, 2H), 1.89 (d, *J* = 2.6 Hz, 6H), 1.77 – 1.69 (m, 2H); ^{13}C NMR (125 MHz, D_2O) δ 101.83, 99.10, 96.78, 93.71, 77.01, 76.25, 75.96,74.45, 73.33, 71.78, 70.49, 69.85, 69.49, 68.85, 68.69, 68.19, 66.32, 66.11, 66.10, 65.79, 65.56, 53.40, 52.70, 36.98, 35.69, 26.99, 21.88; HRMS (ESI) Anal. Calcd for $C_{33}H_{52}N_4O_{33}S_3^{2-}$ $[M+H]^{2-}$: 564.0843, found 564.0844.

Compound **69:** The solution of compound **S106** (6.5 mg, 4.2 µmol) in DMF was subjected to *O*-sulfation followed by saponification, *N*-sulfation and hydrogenation according to the general procedures to afford compound **68** (2.0 mg, 41% over 4 steps). $[\alpha]_D^{20}$ = +2.6° (*c* = 0.1, H_2O); ^{1}H NMR (500 MHz, D_2O) δ 5.36 (d, *J* = 2.9 Hz, 1H), 5.28 (d, *J* = 3.6 Hz, 1H), 4.92 (d, *J* = 4.3 Hz, 1H), 4.42 (d, *J* = 7.9 Hz, 1H), 4.38 (d, *J* = 2.4 Hz, 1H), 4.30 (dd, *J* = 11.1, 2.4 Hz, 1H), 4.22 (dd, *J* = 10.9, 6.4 Hz, 2H), 4.08 (d, *J* = 6.2 Hz, 3H), 4.01 (dt, *J* = 5.4, 3.0 Hz, 3H), 3.95 (d, *J* = 9.7 Hz, 1H), 3.75 (dd, *J* = 10.6, 3.7 Hz, 2H), 3.65 (dd, *J* = 12.1, 6.8 Hz, 2H), 3.61 – 3.54 (m, 4H), 3.49 (t, *J* = 9.6 Hz, 1H), 3.42 (t, *J* = 9.7 Hz, 1H), 3.26 – 3.15 (m, 3H), 3.13 (dd, *J* = 10.5, 3.5 Hz, 1H), 2.85 (t, *J* = 7.6 Hz, 1H), 1.89 (s, 3H), 1.81 – 1.71 (m, 2H); ^{13}C NMR (125 MHz, D_2O) δ 101.66, 99.78, 96.87, 95.45, 76.36, 75.97, 75.91, 73.38, 70.45, 69.93, 68.85, 68.63, 66.18, 66.18, 66.17, 65.89, 57.27, 37.10, 35.69, 26.68, 21.83; HRMS (ESI) Anal. Calcd for $C_{31}H_{48}N_4O_{35}S_4^{3-}$ $[M-3H]^{3-}$: 291.0251, found 291.0292.

Compound **70:** The solution of compound **S103** (26 mg, 14 µmol) in pyridine was subjected to silyl group removal, followed by *O*-sulfation, saponification, *N*-acylation and hydrogenation according to the general procedures to afford compound **70** (5.5 mg, 38% over 5 steps). $[\alpha]_D^{20}$ = +4.0° (*c* = 0.1, H_2O); ^{1}H NMR (500 MHz, D_2O) δ 5.26 (d, *J* = 3.7 Hz, 1H), 5.00 (d, *J* = 3.6 Hz, 1H), 4.73 (d, *J* = 3.0 Hz, 1H), 4.40 (d, *J* = 7.9 Hz, 1H), 4.35 (d, *J* = 2.6 Hz, 1H), 4.27 (dd, *J* = 11.0, 2.2 Hz, 1H), 4.19 (dd, *J* = 11.1, 2.1 Hz, 1H), 4.05 (d, *J* = 7.2 Hz, 3H), 4.01 – 3.97 (m, 1H), 3.91 (dd, *J* = 6.6, 3.0 Hz, 2H), 3.79 (dd, *J* = 10.5, 3.7 Hz, 1H), 3.77 – 3.71 (m, 3H), 3.63 (t, *J* = 10.3 Hz, 2H), 3.61 – 3.50 (m, 5H), 3.40 (t, *J* = 9.6 Hz, 1H), 3.25 – 3.10 (m, 3H), 2.87 – 2.80 (m, 2H), 1.88 (s, 3H), 1.83 (s, 3H), 1.77 – 1.68 (m, 2H); ^{13}C NMR (125 MHz, D_2O) δ 101.89, 101.31, 96.85, 94.15, 77.65, 76.28, 75.96, 75.96, 73.87, 73.39, 69.81, 69.31, 69.17, 68.92, 68.92, 68.79, 68.69, 66.57, 66.18, 66.15, 65.80, 53.42, 53.40, 52.84, 46.32, 36.91, 35.66, 35.63, 26.70,21.85, 21.43; HRMS (ESI) Anal. Calcd for $C_{33}H_{52}N_4O_{30}S_2^{2-}$ $[M]^{2-}$: 1048.2119, found 1048.2123.

Compound **71:** The solution of compound **S103** (13 mg, 6.8 μmol) in pyridine was subjected to silyl group removal, followed by *O*-sulfation, saponification, *N*-sulfation and hydrogenation according to the general procedures to afford compound **71** (2.5 mg, 34% over 5 steps). $[\alpha]_D^{20}$ = +3.3° (*c* = 0.1, H_2O); ^{1}H NMR (500 MHz, D_2O) δ 5.27 (d, *J* = 3.5 Hz, 1H), 5.20 (d, *J* = 3.5 Hz, 1H), 4.75 (d, *J* = 2.4 Hz, 1H), 4.41 (d, *J* = 8.0 Hz, 1H), 4.35 – 4.28 (m, 2H), 4.20 (d, *J* = 10.7 Hz, 1H), 4.15 – 3.94 (m, 6H), 3.91 (d, *J* = 2.7 Hz, 1H), 3.86 (d, *J* = 9.7 Hz, 1H), 3.78 – 3.72 (m, 2H), 3.66 – 3.45 (m, , 8H), 3.40 (t, *J* = 9.7 Hz, 1H), 3.19 (tt, *J* = 13.4, 6.6 Hz, 3H), 3.10 (dd, *J* = 10.3, 3.3 Hz, 1H), 2.82 (t, *J* = 7.7 Hz, 1H), 1.88 (s, 3H), 1.77 – 1.67 (m, 1H); ^{13}C NMR (125 MHz, D_2O) δ 101.80, 101.16, 96.81, 95.28, 76.31, 76.29, 74.49, 73.40, 70.44, 69.91, 69.45, 69.38, 68.93, 68.65, 68.55, 68.44, 67.84, 66.54, 66.18, 66.07, 65.72, 57.21, 53.40, 36.93, 35.70, 26.61, 21.81; HRMS (ESI) Anal. Calcd for $C_{31}H_{49}N_4O_{32}S_3^{3-}$ $[M-3H]^{3-}$: 361.7169, found 361.7167.

Scheme S8. Syntheses of tetrasaccharides **72** – **87**.

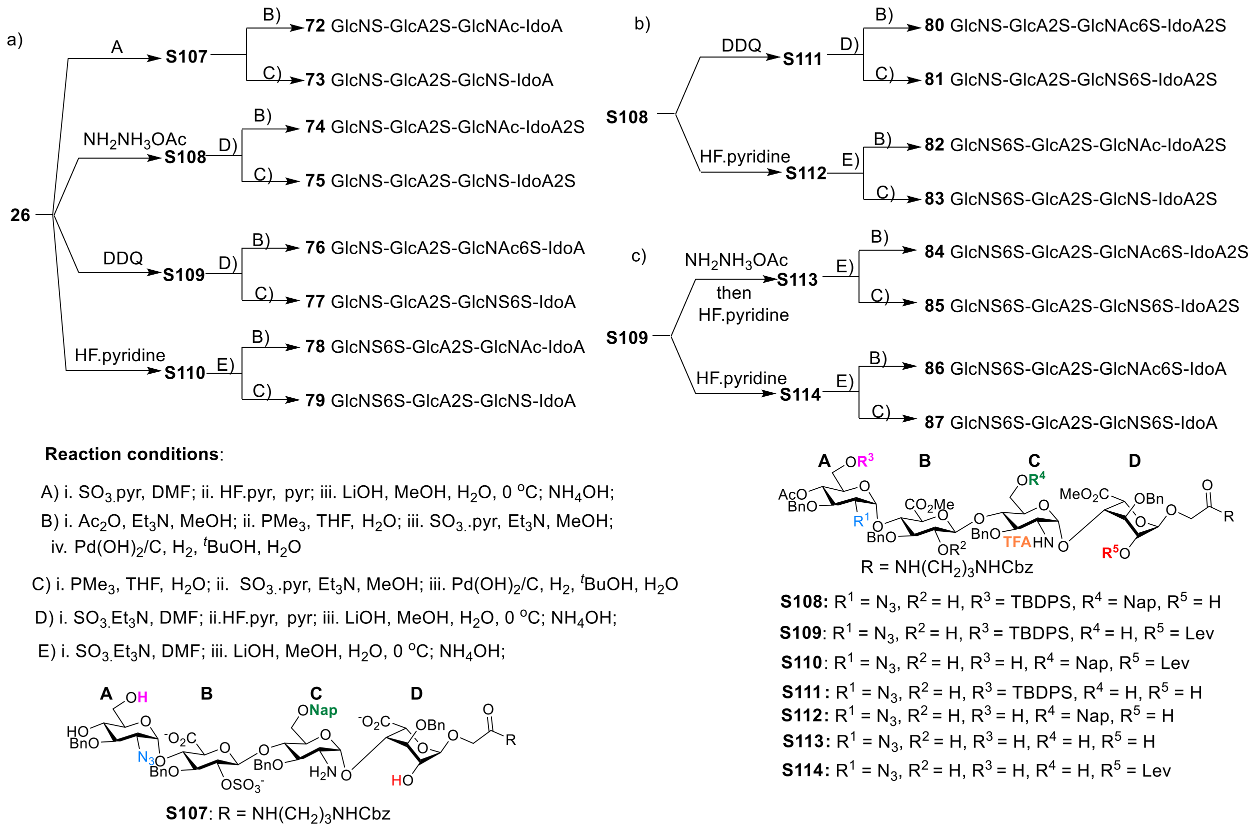

Compound **72:** The solution of compound **S107** (7.5 mg, 5.0 µmol) in methanol was subjected to *N*-acylation, followed by azide reduction, *N*-sulfation, and hydrogenation according to the general procedures to afford compound **72** (1.6 mg, 32% over 4 steps). $[\alpha]_D^{20}$ = +2.8° (c = 0.1, H_2O); ^1H NMR (500 MHz, D_2O) δ 5.52 (s, 1H), 5.00 (d, J = 14.2 Hz, 1H), 4.73 (d, J = 14.5 Hz, 1H), 4.55 (d, J = 7.4 Hz, 1H), 4.35 (d, J = 7.4 Hz, 1H), 4.15 – 4.02 (m, 2H), 3.97 (t, J = 6.8 Hz, 1H), 3.87 (dd, J = 19.9, 10.7 Hz, 4H), 3.79 – 3.65 (m, 8H), 3.61 (d, J = 20.1 Hz, 2H), 3.57 – 3.38 (m, 5H), 3.30 (t, J = 8.8 Hz, 1H), 3.05 (d, J = 10.3 Hz, 1H), 2.82 (t, J = 7.3 Hz, 1H), 1.85 (s, 3H), 1.76 – 1.66 (m, 2H); ^{13}C NMR (125 MHz, D_2O) δ 101.50, 100.47, 97.08, 94.46, 79.69, 75.82, 74.74, 71.58, 71.31, 69.55, 69.54, 69.46, 69.45, 69.30, 68.91, 66.87, 59.99, 59.18, 59.17, 57.89, 57.80, 53.09, 36.92, 26.78, 21.85; HRMS (ESI) Anal. Calcd for $C_{31}H_{50}N_4O_{29}S_2^{2-}$ $[M-2H]^{2-}$: 503.1006, found 503.1016.

Compound **73:** The solution of compound **S107** (5.0 mg, 3.2 µmol) in THF and water was subjected to azide reduction, followed by *N*-sulfation, and hydrogenation according to the general procedures to afford compound **72** (1.7 mg, 51% over 3 steps). $[\alpha]_D^{20}$ = +1.9° (c = 0.1, H_2O); ^1H NMR (500 MHz, D_2O) δ 5.51 (d, J = 3.6 Hz, 1H), 5.19 (d, J = 3.7 Hz, 1H), 4.74 (d, J = 2.9 Hz, 1H), 4.54 (d, J = 7.7 Hz, 1H), 4.33 (d, J = 2.5 Hz, 1H), 4.11 – 3.93 (m, 4H), 3.92 – 3.81 (m, 3H), 3.72 – 3.43 (m, 13H), 3.30 (t, J = 9.7 Hz, 1H), 3.23 – 3.12 (m, 3H), 3.05 (ddd, J = 14.1, 10.4, 3.7

Hz, 2H), 2.81 (t, J = 7.7 Hz, 2H), 1.80 – 1.59 (m, 1H); ^{13}C NMR (125 MHz, D_2O) δ 101.48, 100.48, 97.20, 95.80, 79.69, 79.34, 75.89, 75.30, 71.62, 71.28, 70.47, 70.33, 69.55, 68.96, 68.92, 68.59, 66.88, 60.04, 59.28, 57.65, 37.01, 35.66, 26.72; HRMS (ESI) Anal. Calcd for $C_{29}H_{47}N_4O_{31}S_3^{3-}$ $[M]^{3-}$: 347.7134, found 347.7123.

Compound **74:** The solution of compound **S108** (29.0 mg, 15.3 µmol) in DMF was subjected to *O*-sulfation, followed by silyl group deprotection, saponification, *N*-acylation, azide reduction, *N*-sulfation, and hydrogenation according to the general procedures to afford compound **74** (5.3 mg, 32% over 7 steps). $[\alpha]_D^{20}$ = -2.0° (c = 0.1, H_2O); ^{1}H NMR (500 MHz, D_2O) δ 5.51 (d, J = 3.7 Hz, 1H), 4.99 (d, J = 3.7 Hz, 1H), 4.92 (d, J = 2.7 Hz, 1H), 4.54 (d, J = 7.8 Hz, 1H), 4.37 (d, J = 2.8 Hz, 1H), 4.17 (dd, J = 5.0, 2.7 Hz, 1H), 4.07 (d, J = 2.1 Hz, 2H), 4.04 – 3.95 (m, 2H), 3.92 – 3.79 (m, 5H), 3.73 – 3.60 (m, 8H), 3.55 – 3.42 (m, 4H), 3.30 (t, J = 9.6 Hz, 1H), 3.18 (t, J = 6.6 Hz, 2H), 3.04 (dd, J = 10.3, 3.7 Hz, 1H), 2.81 (t, J = 7.7 Hz, 1H), 2.53 (d, J = 4.7 Hz, 1H), 1.87 (s, 3H), 1.75 – 1.68 (m, 2H); ^{13}C NMR (125 MHz, D_2O) δ 100.54, 99.37, 97.22, 93.90, 79.84, 79.64, 75.15, 72.41, 71.63, 70.66, 69.48, , 69.09 68.70, 66.56, 66.16, 59.92, 59.33, 57.96, 52.88, 35.70, 37.06, 26.91, 22.03; HRMS (ESI) Anal. Calcd for $C_{31}H_{52}N_4O_{32}S_3$ $[M-3H]^{3-}$: 361.7169, found 361.7169.

Compound **75:** The solution of compound **S108** (27.0 mg, 14.5 µmol) in DMF was subjected to *O*-sulfation, followed by silyl group deprotection, saponification, azide reduction, *N*-sulfation, and hydrogenation according to the general procedures to afford compound **75** (5.2 mg, 34% over 6 steps). $[\alpha]_D^{20}$ = +1.6° (c = 0.1, H_2O); ^{1}H NMR (500 MHz, D_2O) δ 5.51 (d, J = 2.7 Hz, 1H), 5.34 (d, J = 3.0 Hz, 1H), 4.90 (dd, J = 4.6, 1.9 Hz, 1H), 4.55 (dd, J = 7.8, 1.9 Hz, 1H), 4.37 (t, J = 2.6 Hz, 1H), 4.21 (ddd, J = 6.8, 4.7, 1.9 Hz, 1H), 4.12 – 3.95 (m, 5H), 3.88 (ddd, J = 9.0, 5.8, 2.7 Hz, 1H), 3.80 (ddd, J = 16.0, 13.2, 2.8 Hz, 1H), 3.74 – 3.68 (m, 3H), 3.68 3.58 (m, 3H), 3.57 – 3.51 (m, 2H), 3.50 – 3.43 (m, 2H), 3.30 (td, J = 9.7, 1.9 Hz, 1H), 3.23 – 3.14 (m, 2H), 3.09 (dt, J = 10.9, 2.2 Hz, 1H), 3.04 (ddd, J = 10.3, 3.8, 1.7 Hz, 1H), 2.87 – 2.83 (m, 1H), 1.75 (p, J = 7.3 Hz, 2H); ^{13}C NMR (125 MHz, D_2O) δ 100.54, 99.95, 97.22, 95.66, 79.44, 77.69, 76.32, 75.93, 71.63, 70.65, 70.46, 69.29, 66.55, 60.11, 59.33, 57.57, 37.06, 35.69, 26.71; HRMS (ESI) Anal. Calcd for $C_{29}H_{50}N_4O_{34}S_4$ $[M-2H]^{2-}$: 562.0522, found 562.0531.

Compound **76:** The solution of compound **S109** (14.0 mg, 7.9 µmol) in DMF was subjected to *O*-sulfation, followed by silyl group deprotection, saponification, *N*-acylation, azide reduction, *N*-sulfation, and hydrogenation according to the general procedures to afford compound **76** (2.5 mg, 29% over 7 steps). $[\boldsymbol{\alpha}]_{\mathbf{D}}^{\mathbf{20}}$ = +2.1° (c = 0.1, H_2O); ^{1}H NMR (500 MHz, D_2O) δ 5.48 (d, J = 3.6 Hz, 1H), 5.03 (d, J = 3.8 Hz, 1H), 4.72 (d, J = 3.8 Hz, 1H), 4.58 (d, J = 7.8 Hz, 1H), 4.44 – 4.34 (m, 2H), 4.13 – 4.01 (m, 3H), 4.00 – 3.95 (m, 1H), 3.94 – 3.91 (m, 1H), 3.88 – 3.78 (m, 3H), 3.74 – 3.52 (m, 10H), 3.47 (t, J = 9.8 Hz, 1H), 3.30 (t, J = 9.7 Hz, 1H), 3.27 – 3.20 (m, 1H), 3.15 – 3.07 (m, 1H), 3.04 (dd, J = 10.3, 3.7 Hz, 1H), 2.82 (t, J = 7.8 Hz, 1H), 1.85 (s, 3H), 1.78 – 1.67 (m, 2H); ^{13}C NMR (125 MHz, D_2O) δ 101.73, 99.90, 97.43, 94.63, 79.60, 77.68, 76.11, 75.01, 74.87, 71.55, 71.47, 71.19, 69.79, 69.78, 69.52, 69.52, 66.94, 68.87, 68.83, 65.69, 65.60, 59.99, 57.94,

53.00, 36.92, 35.65, 35.64, 26.69, 21.81; HRMS (ESI) Anal. Calcd for $C_{31}H_{49}N_4O_{32}S_3^{3-}$ $[M-3H]^{3-}$: 361.7169, found 361.7282.

Compound **77:** The solution of compound **S109** (16.0 mg, 8.6 μmol) in DMF was subjected to *O*-sulfation, followed by silyl group deprotection, saponification, azide reduction, *N*-sulfation, and hydrogenation according to the general procedures to afford compound **77** (2.7 mg, 28% over 6 steps). $[\alpha]_D^{20}$ = +2.3° (*c* = 0.1, H_2O); ^{1}H NMR (500 MHz, D_2O) δ 5.48 (d, *J* = 3.6 Hz, 1H), 5.21 (d, *J* = 3.7 Hz, 1H), 4.75 (d, *J* = 3.0 Hz, 1H), 4.58 (d, *J* = 7.9 Hz, 1H), 4.38 (d, *J* = 11.1 Hz, 1H), 4.36 – 4.33 (m, 1H), 4.11 – 3.98 (m, 4H), 3.95 (d, *J* = 8.0 Hz, 1H), 3.92 (t, *J* = 3.0 Hz, 1H), 3.86 – 3.79 (m, 2H), 3.70 – 3.66 (m, 2H), 3.66 – 3.58 (m, 3H), 3.57 – 3.51 (m, 3H), 3.49 – 3.44 (m, 1H), 3.32 – 3.27 (m, 1H), 3.25 – 3.18 (m, 1H), 3.17 – 3.09 (m, 2H), 3.03 (dd, *J* = 10.3, 3.7 Hz, 1H), 2.87 – 2.79 (m, 2H), 1.78 – 1.67 (m, 2H); ^{13}C NMR (125 MHz, D_2O) δ 101.51, 99.83, 97.47, 95.80, 79.64, 77.44, 76.16, 75.45, 74.99, 71.54, 71.49, 71.22, 69.52, 69.09, 69.03, 68.77, 68.71, 68.56, 66.88, 65.70, 65.64, 59.94, 57.95, 57.28, 36.98, 35.64, 35.63, 26.66; HRMS (ESI) Anal. Calcd for $C_{29}H_{47}N_4O_{34}S_4^{3-}$ $[M-3H]^{3-}$: 374.3657, found 374.3694.

Compound **78:** The solution of compound **S110** (12.0 mg, 6.9 μmol) in DMF was subjected to *O*-sulfation, followed by saponification, *N*-acylation, azide reduction, *N*-sulfation, and hydrogenation according to general procedure to afford compound **78** (2.4 mg, 31% over 6 steps). $[\alpha]_D^{20}$ = +0.3° (c = 0.1, H_2O); ^{1}H NMR (500 MHz, D_2O) δ 5.50 (d, *J* = 3.7 Hz, 1H), 5.01 (d, *J* = 3.8 Hz, 1H), 4.54 (d, *J* = 7.7 Hz, 1H), 4.35 (d, *J* = 3.2 Hz, 1H), 4.18 (d, *J* = 10.8 Hz, 1H), 4.06 (d, *J* = 7.4 Hz, 2H), 3.98 (dd, *J* = 15.8, 9.3 Hz, 2H), 3.92 – 3.82 (m, 3H), 3.78 – 3.64 (m, 9H, 3.57 – 3.38 (m, 6H), 3.14 – 3.06 (m, 2H), 2.78 (t, *J* = 7.5 Hz, 1H), 1.84 (s, 3H), 1.72 – 1.64 (m, 2H); ^{13}C NMR (125 MHz, D_2O) δ 101.71, 100.73, 97.41, 94.68, 79.64, 79.25, 75.93, 74.95, 69.68, 69.48, 68.90, 66.95, 66.16, 59.33, 57.96, 53.27, 37.06, 27.30, 21.83; HRMS (ESI) Anal. Calcd for $C_{31}H_{52}N_4O_{32}S_3$ $[M-2H]^{2-}$: 543.0790, found 543.0800.

Compound **79:** The solution of compound **S110** (7.0 mg, 4.2 μmol) in DMF was subjected to *O*-sulfation, followed by saponification, azide reduction, *N*-sulfation, and hydrogenation according to the general procedures to afford compound **79** (1.4 mg, 30% over 5 steps). $[\alpha]_D^{20}$ = +1.3° (c = 0.1, H_2O); ^{1}H NMR (500 MHz, D_2O) δ 5.51 (d, *J* = 3.7 Hz, 1H), 5.20 (d, *J* = 3.7 Hz, 1H), 4.74 (d, *J* = 3.1 Hz, 1H), 4.54 (d, *J* = 7.8 Hz, 1H), 4.34 (d, *J* = 2.6 Hz, 1H), 4.19 (d, *J* = 10.7 Hz, 1H), 4.07 – 3.95 (m, 5H), 3.90 – 3.80 (m, 3H), 3.71 – 3.63 (m, 5H), 3.61 – 3.53 (m, 3H), 3.50 – 3.38 (m, 4H), 3.20 – 3.13 (m, 2H), 3.07 (dd, *J* = 10.0, 3.8 Hz, 2H), 2.87 – 2.80 (m, 2H), 1.75 – 1.66 (m, 2H); ^{13}C NMR (125 MHz, D_2O) δ 101.51, 100.54, 97.22, 95.66, 79.84, 76.13, 75.34, 71.05, 70.07, 69.09, 68.90, 68.51, 66.95, 66.56, 66.16, 59.33, 59.13, 57.57, 37.06, 35.70, 26.71; HRMS (ESI) Anal. Calcd for $C_{29}H_{50}N_4O_{34}S_4$ $[M-2H]^{2-}$: 522.0738, found 522.0740.

Compound **80:** The solution of compound **S111** (20.0 mg, 11.0 μmol) in DMF was subjected to *O*-sulfation, followed by silyl group deprotection, saponification, *N*-acylation, azide reduction, *N*-sulfation, and hydrogenation according to the general procedures to afford compound **80** (5.3 mg,

42% over 7 steps). $[\alpha]_D^{20}$ = -5.6° (c = 0.1, H_2O); ^1H NMR (500 MHz, D_2O) δ 5.47 (d, *J* = 3.7 Hz, 1H), 5.00 (d, *J* = 3.8 Hz, 1H), 4.93 (d, *J* = 2.7 Hz, 1H), 4.57 (d, *J* = 7.7 Hz, 1H), 4.42 – 4.36 (m, 2H), 4.17 (dd, *J* = 4.9, 2.7 Hz, 1H), 4.08 – 4.04 (m, 3H), 4.02 – 3.95 (m, 2H), 3.92 (t, *J* = 3.1 Hz, 1H), 3.87 – 3.82 (m, 3H), 3.70 – 3.67 (m, 2H), 3.66 – 3.61 (m, 3H), 3.58 – 3.52 (m, 2H), 3.48 – 3.43 (m, 1H), 3.29 (t, *J* = 9.7 Hz, 1H), 3.18 (dt, *J* = 8.6, 6.6 Hz, 2H), 3.03 (dd, *J* = 10.3, 3.7 Hz, 1H), 2.83 (t, *J* = 7.7 Hz, 2H), 1.88 (s, 3H), 1.76 – 1.69 (m, 2H); ^{13}C NMR (125 MHz, D_2O) δ 100.15, 99.37, 97.41, 94.09, 79.84, 77.88, 76.13, 74.95, 72.81, 71.63, 71.44, 69.48, 68.90, 68.70, 66.56, 65.58, 60.11, 58.49, 52.88, 37.06, 36.32, 26.71, 22.03; HRMS (ESI) Anal. Calcd for $C_{31}H_{52}N_4O_{35}S_4$ $[M-2H]^{2-}$: 583.0574, found 583.0566.

Compound **81:** The solution of compound **S111** (20.0 mg, 11.0 μmol) in DMF was subjected to *O*-sulfation, followed by silyl group deprotection, saponification, azide reduction, *N*-sulfation, and hydrogenation according to the general procedures to afford compound **81** (5.9 mg, 45% over 6 steps). $[\alpha]_D^{20}$ = +0.6° (c = 0.1, H_2O); ^1H NMR (500 MHz, D_2O) δ 5.47 (d, *J* = 3.7 Hz, 1H), 5.33 (d, *J* = 3.8 Hz, 1H), 4.91 (d, *J* = 4.3 Hz, 1H), 4.58 (d, *J* = 7.9 Hz, 1H), 4.41 – 4.35 (m, 2H), 4.21 (dd, *J* = 7.8, 4.3 Hz, 1H), 4.12 – 4.01 (m, 4H), 4.00 – 3.95 (m, 2H), 3.88 – 3.81 (m, 2H), 3.69 – 3.68 (m, 1H), 3.62 (dd, *J* = 6.3, 3.2 Hz, 1H), 3.57 – 3.51 (m, 3H), 3.49 – 3.44 (m, 1H), 3.29 (t, *J* = 9.7 Hz, 1H), 3.18 (q, *J* = 6.3 Hz, 2H), 3.13 (dd, *J* = 10.2, 3.5 Hz, 1H), 3.03 (dd, *J* = 10.3, 3.7 Hz, 1H), 2.88 – 2.81 (m, 2H), 1.78 – 1.69 (m, 2H); ^{13}C NMR (125 MHz, D_2O) δ 99.95, 97.61, 96.05, 79.64, 77.69, 77.49, 76.71, 74.95, 71.63, 71.24, 70.46, 70.27, 69.48, 69.09, 65.97, 65.77, 60.11, 57.96, 57.38, 46.24, 37.06, 35.70, 26.52; HRMS (ESI) Anal. Calcd for $C_{29}H_{50}N_4O_{37}S_5$ $[M-3H]^{3-}$: 401.0180, found 401.0109.

Compound **82:** The solution of compound **S108** (15.3 mg, 8.2 μmol) in pyridine (2 mL) was subjected for silyl group deprotection according to the general procedure to afford compound **S112**, which was followed by *O*-sulfation, saponification, *N*-acylation, azide reduction, *N*-sulfation, and hydrogenation according to the general procedures to afford compound **82** (4.3 mg, 45% over 7 steps). $[\alpha]_D^{20}$ = +2.1° (c = 0.1, H_2O); ^1H NMR (500 MHz, D_2O) δ 5.50 (d, *J* = 3.9 Hz, 1H), 4.99 (d, *J* = 3.8 Hz, 1H), 4.92 (d, *J* = 2.7 Hz, 1H), 4.54 (d, *J* = 7.8 Hz, 1H), 4.37 (d, *J* = 2.8 Hz, 1H), 4.21 – 4.15 (m, 2H), 4.07 (d, *J* = 3.9 Hz, 2H), 4.04 – 3.96 (m, 3H), 3.92 – 3.78 (m, 5H), 3.75 – 3.62 (m, 6H), 3.58 – 3.38 (m, 5H), 3.22 – 3.15 (m, 3H), 3.07 (dd, *J* = 9.9, 3.6 Hz, 1H), 2.84 (t, *J* = 7.7 Hz, 2H), 1.87 (s, 3H), 1.78 – 1.71 (m, 2H); ^{13}C NMR (125 MHz, D_2O) δ 100.80, 100.54, 97.41, 93.90, 79.64, 79.45, 75.15, 71.05, 69.68, 68.90, 68.70, 66.56, 59.33, 58.68, 52.88, 36.87, 35.70, 26.52, 22.73; HRMS (ESI) Anal. Calcd for $C_{31}H_{52}N_4O_{35}S_4Na_2$ $[M-4H+2Na]^{2-}$: 605.0394, found 605.0381.

Compound **83:** The solution of compound **S108** (21 mg, 11.2 μmol) in pyridine (2 mL) was subjected for silyl group deprotection according to the general procedure to afford compound **S112**, which was followed by *O*-sulfation, saponification, azide reduction, *N*-sulfation, and hydrogenation according to the general procedures to afford compound **83** (6.9 mg, 51% over 6 steps). $[\alpha]_D^{20}$ = +2.0° (c = 0.1, H_2O); ^1H NMR (500 MHz, D_2O) δ 5.50 (d, *J* = 3.8 Hz, 1H), 5.32

(d, J = 3.9 Hz, 1H), 4.92 – 4.87 (m, 1H), 4.36 (d, J = 3.3 Hz, 1H), 4.23 – 4.15 (m, 2H), 4.11 – 3.94 (m, 6H), 3.86 (t, J = 7.5 Hz, 1H), 3.83 – 3.75 (m, 2H), 3.73 – 3.67 (m, 4H), 3.63 (d, J = 11.7 Hz, 1H), 3.55 – 3.40 (m, 4H), 3.23 – 3.04 (m, 4H), 2.97 – 2.89 (m, 1H), 2.81 (t, J = 7.5 Hz, 2H), 1.71 (d, J = 6.8 Hz, 2H).; ^{13}C NMR (125 MHz, D_2O) δ 100.54, 99.95, 97.41, 95.85, 77.49, 76.32, 75.93, 70.66, 70.27, 66.56, 70.27, 69.48, 59.33, 57.77, 55.42, 37.06, 35.89, 26.91; HRMS (ESI) Anal. Calcd for $C_{29}H_{50}N_4O_{37}S_5$ $[M-2H]^{2-}$: 602.0306, found 602.0357.

Compound **84:** The solution of compound **S113** (6.0 mg, 3.5 µmol) in DMF was subjected for *O*-sulfation, followed by saponification, *N*-acylation, azide reduction, *N*-sulfation, and hydrogenation according to the general procedures to afford compound **84** (1.5 mg, 34% over 6 steps). $[\alpha]_D^{20}$ = +1.1° (c = 0.1, H_2O); ^{1}H NMR (500 MHz, D_2O) δ 5.48 (d, J = 3.6 Hz, 1H), 5.01 (d, J = 3.7 Hz, 1H), 4.93 (d, J = 2.6 Hz, 1H), 4.57 (d, J = 7.9 Hz, 1H), 4.39 (dd, J = 15.5, 7.0 Hz, 2H), 4.22 – 4.15 (m, 2H), 4.09 – 4.03 (m, 3H), 4.02 – 3.95 (m, 3H), 3.94 – 3.89 (m, 1H), 3.88 – 3.80 (m, 3H), 3.74 – 3.62 (m, 4H), 3.56 (t, J = 9.5 Hz, 1H), 3.49 – 3.38 (mi, 2H), 3.24 – 3.12 (m, 2H), 3.06 (dd, J = 10.0, 3.7 Hz, 1H), 2.86 – 2.78 (m, 2H), 1.88 (s, 3H), 1.78 – 1.68 (m, 2H); ^{13}C NMR (125 MHz, D_2O) δ 99.96, 99.49, 97.61, 94.10, 79.66, 76.17, 75.10, 72.94, 71.09, 69.74, 68.85, 68.78, 68.77, 66.56, 65.97, 65.87, 65.74, 57.85, 52.70, 36.97, 35.63, 26.67, 21.97; HRMS (ESI) Anal. Calcd for $C_{31}H_{47}N_4O_{38}S_5Na_2{}^{3-}$ $[M-5H+2Na]^{3-}$: 429.6761, found 429.6764.

Compound **85:** The solution of compound **S113** (9.0 mg, 6.0 µmol) in DMF was subjected for *O*-sulfation, followed by saponification, azide reduction, *N*-sulfation, and hydrogenation according to the general procedures to afford compound **85** (3.1 mg, 40% over 5 steps). $[\alpha]_D^{20}$ = +2.0° (c = 0.1, H_2O); ^{1}H NMR (500 MHz, D_2O) δ 5.48 (d, J = 3.6 Hz, 1H), 5.34 (d, J = 3.6 Hz, 1H), 4.89 (d, J = 4.5 Hz, 1H), 4.58 (d, J = 7.9 Hz, 1H), 4.40 – 4.35 (m, 2H), 4.22 – 4.17 (m, 2H), 4.11 – 3.96 (m, 8H), 3.89 – 3.79 (m, 2H), 3.74 – 3.66 (m, 3H), 3.56 – 3.51 (m, 2H), 3.49 – 3.37 (m, 2H), 3.18 (td, J = 6.6, 2.8 Hz, 2H), 3.13 (dd, J = 10.2, 3.8 Hz, 1H), 3.06 (dd, J = 10.1, 3.7 Hz, 1H), 2.83 (t, J = 7.7 Hz, 2H), 1.77 – 1.68 (m, 2H); ^{13}C NMR (125 MHz, D_2O) δ 100.05, 99.82, 97.66, 96.09, 80.66, 79.58, 78.21, 77.54, 76.66, 76.15, 70.96, 70.73, 70.51, 69.61, 69.00, 68.58, 66.91, 66.65, 66.22, 57.77, 57.33, 36.89, 35.57, 26.46; HRMS (ESI) Anal. Calcd for $C_{29}H_{46}N_4O_{40}S_6Na_2{}^{2-}$ $[M+2Na+2H]^{2-}$: 663.9906, found 663.9940.

Compound **86:** The solution of compound **S114** (13.0 mg, 8.4 µmol) in DMF was subjected to *O*-sulfation, followed by saponification, *N*-acylation, azide reduction, *N*-sulfation, and hydrogenation according to general procedure to afford compound **86** (3.0 mg, 31% over 6 steps). $[\alpha]_D^{20}$ = -1.5° (c = 0.05, H_2O); ^{1}H NMR (500 MHz, D_2O) δ 5.48 (d, J = 3.6 Hz, 1H), 5.03 (d, J = 3.8 Hz, 1H), 4.71 (d, J = 3.9 Hz, 1H), 4.57 (d, J = 7.9 Hz, 1H), 4.45 – 4.33 (m, 2H), 4.19 (d, J = 11.2 Hz, 1H), 4.11 – 3.94 (m, 6H), 3.93 – 3.90 (m, 1H), 3.87 – 3.77 (m, 3H), 3.75 – 3.64 (m, 5H), 3.60 – 3.51 (m, 2H), 3.44 (dt, J = 26.3, 9.4 Hz, 2H), 3.26 – 3.03 (m, 3H), 2.80 (dt, J = 15.6, 7.3 Hz, 1H), 1.85 (s, 3H), 1.76 – 1.64 (m, 2H); ^{13}C NMR (125 MHz, D_2O) δ 102.13, 99.95, 97.50, 94.65, 79.53, 76.14, 75.08, 70.99, 69.71, 69.71, 69.00, 68.77, 66.90, 65.97, 66.15, 65.77, 65.52, 57.62, 53.07,

37.14, 26.92, 21.83; HRMS (ESI) Anal. Calcd for $C_{31}H_{49}N_4O_{35}S_4^{3-}$ $[M\text{-}H]^{3-}$: 388.3692, found 388.3693.

Compound **87:** The solution of compound **S114** (14.0 mg, 9.3 μmol) in DMF was subjected to *O*-sulfation, followed by saponification, azide reduction, *N*-sulfation, and hydrogenation according to the general procedures to afford compound **87** (3.7 mg, 33% over 5 steps). $[\boldsymbol{\alpha}]_{\mathbf{D}}^{\mathbf{20}}$ = +2.6° (*c* = 0.1, H_2O); ^1H NMR (500 MHz, D_2O) δ 5.49 (d, *J* = 3.6 Hz, 1H), 5.21 (d, *J* = 3.7 Hz, 1H), 4.75 (d, *J* = 3.1 Hz, 1H), 4.58 (d, *J* = 7.9 Hz, 1H), 4.39 (d, *J* = 10.6 Hz, 1H), 4.35 (d, *J* = 2.7 Hz, 1H), 4.20 (dd, *J* = 11.1, 2.0 Hz, 1H), 4.10 – 3.96 (m, 5H), 3.93 (t, *J* = 3.1 Hz, 1H), 3.87 – 3.78 (m, 2H), 3.74 – 3.64 (m, 5H), 3.62 – 3.53 (m, 2H), 3.45 (dt, *J* = 29.5, 9.5 Hz, 2H), 3.25 – 3.18 (m, 1H), 3.17 – 3.10 (m, 2H), 3.07 (dd, *J* = 10.1, 3.7 Hz, 1H), 2.83 (t, *J* = 7.8 Hz, 2H), 1.80 – 1.60 (m, 2H); ^{13}C NMR (125 MHz, D_2O) δ 101.59, 99.88, 97.66, 95.84, 79.58, 77.50, 76.21, 75.51, 75.02, 71.14, 69.69, 69.12, 69.03, 68.88, 68.86, 68.74, 68.61, 66.96, 66.09, 65.92, 65.69, 59.81, 57.76, 57.30, 36.96, 35.69, 35.65, 26.66; HRMS (ESI) Anal. Calcd for $C_{29}H_{45}N_4O_{37}S_5Na_2^{3-}$ $[M\text{-}5H\text{+}2Na]^{3-}$: 415.6726, found 415.6764.

Scheme S9. Syntheses of tetrasaccharides **88** - **103**.

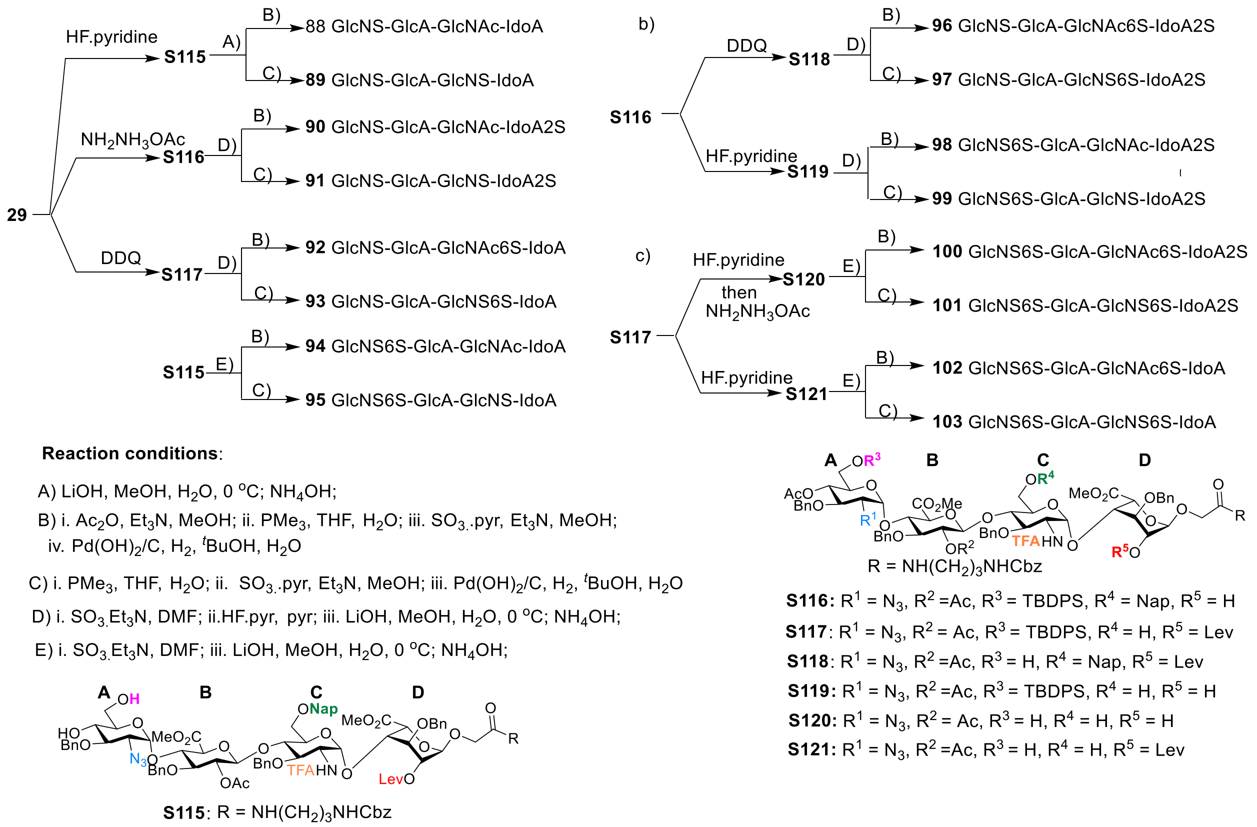

Compound **88:** The solution of compound **S115** (13.0 mg, 7.1 µmol) in methanol and water was subjected for saponification, followed by *N*-acylation, azide reduction, *N*-sulfation, and hydrogenation according to the general procedures to afford compound **88** (2.9 mg, 44% over 5 steps). $[\alpha]_D^{20}$ = +3.4° (c = 0.1, H_2O); ^{1}H NMR (500 MHz, D_2O) δ 5.47 (d, *J* = 3.8 Hz, 1H), 4.99 (d, *J* = 3.4 Hz, 1H), 4.72 (d, *J* = 3.2 Hz, 1H), 4.36 (dd, *J* = 7.9, 1.5 Hz, 2H), 4.06 (s, 2H), 3.89 (t, *J* = 3.3 Hz, 1H), 3.78 – 3.50 (m, 16H), 3.46 – 3.39 (m, 1H), 3.30 (t, *J* = 9.6 Hz, 1H), 3.24 – 3.12 (m, 3H), 3.06 (dd, *J* = 10.4, 3.8 Hz, 1H), 2.87 – 2.79 (m, 3H), 1.83 (s, 3H), 1.77 – 1.69 (m, 2H); ^{13}C NMR (125 MHz, D_2O) δ 102.10, 101.32, 97.02, 94.09, 77.88, 76.13, 73.98, 72.81, 71.44, 69.48, 69.29, 69.09, 68.90, 66.75, 59.92, 57.96, 57.38, 53.08, 46.05, 36.87, 35.70, 26.71, 21.83; HRMS (ESI) Anal. Calcd for $C_{31}H_{52}N_4O_{26}S$ $[M-H]^-$: 927.2518, found 927.2550.

Compound **89:** The solution of compound **S115** (19.0 mg, 10.5 µmol) in methanol and water was subjected for saponification, followed by azide reduction, *N*-sulfation, and hydrogenation according to the general procedures to afford compound **89** (4.9 mg, 48% over 4 steps). $[\boldsymbol{\alpha}]_{\mathbf{D}}^{\mathbf{20}}$ = +3.0° (*c* = 0.1, H_2O); ^{1}H NMR (500 MHz, D_2O) δ 5.47 (d, *J* = 3.7 Hz, 1H), 5.19 (d, *J* = 3.2 Hz, 1H), 4.75 (d, *J* = 2.5 Hz, 1H), 4.36 (d, *J* = 8.0 Hz, 1H), 4.32 (d, *J* = 2.4 Hz, 1H), 4.11 – 4.02 (m, 2H), 3.99 (dd, *J* = 7.9, 3.7 Hz, 1iH), 3.93 – 3.86 (m, 1H), 3.79 – 3.58 (m, 8H), 3.57 – 3.49 (m, 3H), 3.46 – 3.40 (m, 1H), 3.34 – 3.27 (m, 1H), 3.25 – 3.14 (m, 3H), 3.09 – 3.03 (m, 2H), 2.83 (t, *J* = 7.7 Hz, 1H), 1.78 – 1.67 (m, 2H); ^{13}C NMR (125 MHz, D_2O) δ 102.07, 101.18, 97.07, 95.32, 77.71, 76.35, 75.94, 74.46, 72.69, 71.45, 71.07, 70.40, 69.52, 69.50, 68.63, 68.45, 67.98, 66.56, 59.97, 59.28, 59.28, 57.94, 57.31, 36.91, 35.63, 26.61; HRMS (ESI) Anal. Calcd for $C_{29}H_{48}N_4O_{28}S_2^{2-}$ $[M-2H]^{2-}$: 482.0953, found 482.0942.

Compound **90:** The solution of compound **S116** (22.0 mg, 11.5 µmol) in DMF was subjected to *O*-sulfation, followed by silyl group deprotection, saponification, *N*-acylation, azide reduction, *N*-sulfation, and hydrogenation according to the general procedures to afford compound **90** (5.5 mg, 47% over 7 steps). $[\alpha]_D^{20}$ = -5.5° (c = 0.1, H_2O); ^{1}H NMR (500 MHz, D_2O) δ 5.48 (d, *J* = 3.8 Hz, 1H), 4.98 (d, *J* = 3.6 Hz, 1H), 4.93 (d, *J* = 2.6 Hz, 1H), 4.37 – 4.33 (m, 2H), 4.18 (dd, *J* = 4.7, 2.5 Hz, 1H), 4.06 (s, 2H), 4.02 (t, *J* = 4.1 Hz, 1H), 3.90 (t, *J* = 3.0 Hz, 1H), 3.81 (dd, *J* = 10.6, 3.6 Hz, 1H), 3.76 – 3.59 (m, 10H), 3.52 (dd, *J* = 12.3, 6.1 Hz, 2H), 3.43 (t, *J* = 9.8 Hz, 1H), 3.30 (t, *J* = 9.6 Hz, 1H), 3.25 – 3.13 (m, 4H), 3.06 (dd, *J* = 10.3, 3.8 Hz, 1H), 3.00 (q, *J* = 7.3 Hz, 3H), 2.95 – 2.90 (m, 2H), 1.87 (s, 3H), 1.80 – 1.75 (m, 2H); ^{13}C NMR (125 MHz, D_2O) δ 102.10, 99.17, 97.02, 94.78, 77.88, 76.13, 74.76, 72.61, 72.02, 71.44, 71.24, 70.66, 69.48, 68.51, 65.97, 59.92, 59.33, 57.96, 52.88, 49.17, 47.02, 35.89, 23.39, 22.03; HRMS (ESI) Anal. Calcd for $C_{31}H_{52}N_4O_{29}S_2$ $[M-2H]^{2-}$: 503.1006, found 503.1010.

Compound **91:** The solution of compound **S116** (24.0 mg, 12.5 µmol) in DMF was subjected to *O*-sulfation, followed by silyl group deprotection, saponification, azide reduction, *N*-sulfation, and hydrogenation according to the general procedures to afford compound **91** (6.6 mg, 51% over 6 steps). $[\alpha]_D^{20}$ = +2.0° (c = 0.1, H_2O); ^{1}H NMR (500 MHz, D_2O) δ 5.47 (d, *J* = 3.8 Hz, 1H), 5.33 (t, *J* = 3.1 Hz, 1H), 4.91 (d, *J* = 4.2 Hz, 1H), 4.35 (dd, *J* = 5.7, 2.3 Hz, 2H), 4.21 (dd, *J* = 7.7, 4.3

Hz, 1H), 4.06 (d, *J* = 4.4 Hz, 2H), 4.03 – 3.96 (m, 2H), 3.77 – 3.60 (m, 10H), 3.55 – 3.41 (m, 5H), 3.30 (t, *J* = 9.6 Hz, 1H), 3.22 (q, *J* = 7.3, 6.0 Hz, 2H), 3.17 – 3.04 (m, 4H), 2.89 – 2.82 (m, 2H), 2.54 (s, 1H), 1.79 – 1.73 (m, 2H); ^{13}C NMR (125 MHz, D_2O) δ 102.10, 99.76, 97.02, 95.46, 77.88, 77.30, 76.13, 75.93, 72.81, 71.44, 70.46, 70.27, 71.05, 69.48, 66.36, 59.92, 59.52, 57.77, 57.57, 46.24, 37.06, 35.70, 26.52; HRMS (ESI) Anal. Calcd for $C_{29}H_{50}N_4O_{31}S_3$ [M-H]$^-$: 1045.1548, found 1045.1511.

Compound **92:** The solution of compound **S117** (19.0 mg, 10.0 µmol) in DMF was subjected to *O*-sulfation, followed by silyl group deprotection, saponification, *N*-acylation, azide reduction, *N*-sulfation, and hydrogenation according to the general procedures to afford compound **92** (3.5 mg, 35% over 7 steps). $[\alpha]_D^{20}$ = +2.6° (*c* = 0.1, H_2O); ^{1}H NMR (500 MHz, D_2O) δ 5.44 (d, *J* = 3.7 Hz, 1H), 5.00 (dd, *J* = 8.8, 3.7 Hz, 1H), 4.73 (dd, *J* = 8.3, 2.9 Hz, 1H), 4.41 (d, *J* = 7.9 Hz, 1H), 4.35 (s, 1H), 4.25 (t, *J* = 8.3 Hz, 1H), 4.13 – 4.02 (m, 3H), 3.95 – 3.89 (m, 2H), 3.81 – 3.73 (m, 2H), 3.71 – 3.52 (m, 11H), 3.44 (t, *J* = 9.8 Hz, 1H), 3.30 (t, *J* = 9.6 Hz, 1H), 3.23 – 3.17 (m, 2H), 3.05 (dd, *J* = 10.4, 3.7 Hz, 1H), 2.81 (t, *J* = 7.6 Hz, 1H), 1.83 (s, 3H), 1.76 – 1.62 (m, 2H); ^{13}C NMR (125 MHz, D_2O) δ 101.44, 101.10, 97.33, 94.07, 76.39, 76.38, 75.42, 75.73, 72.75, 71.44, 71.42, 69.51, 69.28, 69.26, 68.75, 66.93, 66.65, 65.76, 59.94, 59.94, 57.68, 37.24, 26.76, 21.81; HRMS (ESI) Anal. Calcd for $C_{31}H_{50}N_4O_{29}S_2^{2-}$ [M-2H]$^{2-}$: 503.1006, found 503.0996.

Compound **93:** The solution of compound **S117** (19.0 mg, 10.0 µmol) in DMF was subjected to *O*-sulfation, followed by silyl group deprotection, saponification, azide reduction, *N*-sulfation, and hydrogenation according to the general procedures to afford compound **92** (4.3 mg, 39% over 6 steps). $[\alpha]_D^{20}$ = +1.1° (c = 0.1, H_2O); ^{1}H NMR (500 MHz, D_2O) δ 5.44 (d, *J* = 3.7 Hz, 1H), 5.20 (d, *J* = 3.6 Hz, 1H), 4.75 (d, *J* = 2.8 Hz, 1H), 4.42 (d, *J* = 7.8 Hz, 1H), 4.33 (d, *J* = 2.6 Hz, 1H), 4.27 (dd, *J* = 11.3, 2.5 Hz, 1H), 4.08 – 3.96 (m, 5H), 3.91 (t, *J* = 3.1 Hz, 1H), 3.86 (d, *J* = 9.8 Hz, 1H), 3.70 – 3.41 (m, 14H), 3.28 (d, *J* = 9.6 Hz, 1H), 3.22 – 3.03 (m, 6H), 2.85 – 2.78 (m, 2H), 1.75 – 1.67 (m, 2H); ^{13}C NMR (125 MHz, D_2O) δ 101.71, 101.12, 97.41, 95.27, 76.91, 75.93, 74.56, 72.81, 71.44, 71.05, 69.48, 68.70, 68.51, 68.12, 66.56, 65.77, 59.92, 57.96, 57.18, 37.06, 35.70, 26.91; HRMS (ESI) Anal. Calcd for $C_{29}H_{50}N_4O_{31}S_3$ [M-2H]$^{2-}$: 522.0738, found 522.0701.

Compound **94:** The solution of compound **S115** (19.0 mg, 8.5 µmol) in DMF was subjected to *O*-sulfation, followed by saponification, *N*-acylation, azide reduction, *N*-sulfation, and hydrogenation according to the general procedures to afford compound **94** (2.0 mg, 24% over 6 steps). $[\alpha]_D^{20}$ = +1.6° (*c* = 0.1, H_2O); ^{1}H NMR (500 MHz, D_2O) δ 5.50 (d, *J* = 3.7 Hz, 1H), 5.00 (d, *J* = 3.5 Hz, 1H), 4.73 (d, *J* = 3.3 Hz, 1H), 4.36 (d, *J* = 8.0 Hz, 2H), 4.18 (dd, *J* = 11.0, 2.2 Hz, 1H), 4.06 (s, 2H), 3.99 (dd, *J* = 11.1, 1.9 Hz, 1H), 3.90 (t, *J* = 3.0 Hz, 1H), 3.79 – 3.60 (m, 11H), 3.58 – 3.51 (m, 2H), 3.46 – 3.40 (m, 2H), 3.26 – 3.09 (m, 4H), 2.83 – 2.78 (m, 1H), 1.84 (s, 3H), 1.75 – 1.65 (m, 2H); ^{13}C NMR (125 MHz, D_2O) δ 102.21, 101.31, 97.04, 94.13, 76.02, 73.96, 72.65, 70.90, 69.49, 69.18, 68.99, 68.85, 68.85, 66.63, 66.15, 66.11, 59.28, 59.19, 57.74, 36.94, 27.03, 21.78; HRMS (ESI) Anal. Calcd for $C_{31}H_{50}N_4O_{29}S_2^{2-}$ [M-2H]$^{2-}$: 503.1006, found 503.1046.

Compound **95:** The solution of compound **S115** (8.5 mg, 5 µmol) in DMF was subjected to *O*-sulfation, followed by saponification, azide reduction, *N*-sulfation, and hydrogenation according to the general procedures to afford compound **95** (1.4 mg, 27% over 5 steps). $[\alpha]_D^{20}$ = +2.1º (c = 0.1, H_2O); ^1H NMR (500 MHz, D_2O) δ 5.49 (d, *J* = 4.0 Hz, 1H), 5.23 – 5.13 (m, 1H), 4.81 – 4.71 (m, 1H), 4.70 – 4.62 (m, 2H), 4.40 – 4.28 (m, 3H), 4.23 – 4.13 (m, 1H), 4.04 (d, *J* = 9.9 Hz, 2H), 3.99 (dd, *J* = 8.3, 3.8 Hz, 2H), 3.93 – 3.86 (m, 1H), 3.76 – 3.59 (m, 9H), 3.56 – 3.49 (m, 2H), 3.46 – 3.35 (m, 3H), 3.26 – 3.13 (m, 2H), 3.12 – 3.04 (m, 2H), 2.82 (dt, *J* = 15.3, 7.7 Hz, 1H), 2.60 – 2.51 (m, 1H), 1.77 – 1.62 (m, 2H).; ^{13}C NMR (151 MHz, D_2O) δ 102.23, 101.26, 97.24, 95.46, 77.90, 76.29, 75.97, 74.68, 72.74, 71.13, 70.49, 69.68, 69.68, 69.04, 68.88, 68.72, 68.23, 66.78, 66.30, 59.37, 36.97, 35.85, 26.98, 17.48; HRMS (ESI) Anal. Calcd for $C_{29}H_{50}N_4O_{34}S_4$ $[M-2H]^{2-}$: 522.0738, found 522.0853.

Compound **96:** The solution of compound **S118** (17 mg, 9.5 µmol) in DMF was subjected to *O*-sulfation, followed by silyl group deprotection, saponification, *N*-acylation, azide reduction, *N*-sulfation, and hydrogenation according to the general procedures to afford compound **96** (3.0 mg, 29% over 7 steps). $[\alpha]_D^{20}$ = +2.2º (c = 0.1, H_2O); ^1H NMR (500 MHz, D_2O) δ 5.45 (d, *J* = 3.2 Hz, 1H), 4.98 (d, *J* = 3.3 Hz, 1H), 4.95 (s, 1H), 4.43 – 4.40 (m, 1H), 4.38 (d, *J* = 2.8 Hz, 1H), 4.25 (d, *J* = 11.0 Hz, 1H), 4.18 (d, *J* = 3.5 Hz, 1H), 4.12 – 4.03 (m, 4H), 3.93 (d, *J* = 13.5 Hz, 2H), 3.87 – 3.83 (m, 1H), 3.71 – 3.53 (m, 9H), 3.44 (td, *J* = 10.0, 9.5, 2.4 Hz, 1H), 3.33 – 3.28 (m, 1H), 3.24 – 3.17 (m, 3H), 3.08 – 3.03 (m, 1H), 2.87 – 2.80 (m, 2H), 1.88 (s, 3H), 1.76 – 1.72 (m, 2H); ^{13}C NMR (125 MHz, D_2O) δ 101.91, 99.17, 97.22, 93.70, 75.93, 74.37, 72.81, 71.63, 71.44, 71.05, 70.39, 69.48, 68.70, 68.12, 66.36, 65.77, 59.92, 57.96, 52.69, 36.87, 35.70, 26.71, 22.03; HRMS (ESI) Anal. Calcd for $C_{31}H_{52}N_4O_{32}S_3$ $[M-H]^-$: 1087.1654, found 1087.1769.

Compound **97:** The solution of compound **S118** (10 mg, 5.8 µmol) in DMF was subjected to *O*-sulfation, followed by silyl group deprotection, saponification, azide reduction, *N*-sulfation, and hydrogenation according to the general procedures to afford compound **97** (1.7 mg, 26% over 6 steps). $[\alpha]_D^{20}$ = +2.6º (c = 0.1, H_2O); ^1H NMR (500 MHz, D_2O) δ 5.45 (d, *J* = 3.7 Hz, 1H), 5.36 (d, *J* = 3.6 Hz, 1H), 4.91 (d, *J* = 4.5 Hz, 1H), 4.43 (d, *J* = 7.9 Hz, 1H), 4.37 (d, *J* = 2.7 Hz, 1H), 4.28 – 4.19 (m, 2H), 4.12 – 4.03 (m, 4H), 4.02 – 3.92 (m, 3H), 3.73 – 3.53 (m, 7H), 3.47 (ddd, *J* = 15.4, 10.5, 9.1 Hz, 2H), 3.30 (t, *J* = 9.6 Hz, 1H), 3.25 – 3.15 (m, 3H), 3.12 (dd, *J* = 10.5, 3.7 Hz, 1H), 3.05 (dd, *J* = 10.4, 3.7 Hz, 1H), 2.85 (t, *J* = 7.7 Hz, 2H), 1.77 – 1.71 (m, 2H) ^{13}C NMR (125 MHz, D_2O) δ 101.71, 99.95, 97.41, 95.46, 77.69, 76.32, 75.93, 72.81, 71.44, 71.05, 70.66, 69.48, 69.29, 66.36, 65.97, 59.92, 57.96, 57.38, 37.06, 35.50, 26.52; HRMS (ESI) Anal. Calcd for $C_{29}H_{50}N_4O_{34}S_4$ $[M-H]^-$: 1125.1117, found 1125.1120.

Compound **98:** The solution of compound **S119** (25 mg, 14.8 µmol) in DMF was subjected to *O*-sulfation, followed by saponification, *N*-acylation, azide reduction, *N*-sulfation, and hydrogenation according to the general procedures to afford compound **98** (6.6 mg, 41% over 6 steps). $[\alpha]_D^{20}$ = +1.6º (c = 0.1, H_2O); ^1H NMR (500 MHz, D_2O) δ 5.49 (d, *J* = 3.8 Hz, 1H), 4.98 (d, *J* = 3.6 Hz, 1H), 4.94 (s, 1H), 4.35 (d, *J* = 10.1 Hz, 2H), 4.18 (d, *J* = 7.8 Hz, 2H), 4.08 3.97 (m, 4H), 3.90 (s,

1H), 3.81 (dd, *J* = 10.7, 3.5 Hz, 1H), 3.77 – 3.50 (m, 12H), 3.42 (p, *J* = 9.5 Hz, 2H), 3.23 (q, *J* = 10.3, 9.4 Hz, 2H), 3.13 – 3.07 (m, 1H), 2.80 (t, *J* = 7.7 Hz, 1H), 1.88 (s, 2H), 1.73 – 1.67 (m, 2H); ^{13}C NMR (125 MHz, D_2O) δ 102.10, 99.17, 97.22, 93.70, 77.88, 76.13, 74.56, 72.61, 71.83, 71.05, 70.66, 69.48, 68.90, 68.31, 66.36, 66.16, 65.77, 59.33, 57.77, 52.88, 48.78, 37.06, 35.70, 26.91, 22.03; HRMS (ESI) Anal. Calcd for $C_{31}H_{52}N_4O_{32}S_3$ $[M-H]^-$: 1087.1654, found 1087.2069.

Compound **99:** The solution of compound **S119** (34 mg, 20.0 μmol) in DMF was subjected to *O*-sulfation, followed by saponification, azide reduction, *N*-sulfation, and hydrogenation according to the general procedures to afford compound **99** (11.5 mg, 51% over 5 steps). $[\alpha]_D^{20}$ = +3.5° (c = 0.19, H_2O); ^{1}H NMR (500 MHz, D_2O) δ 5.49 (d, *J* = 3.8 Hz, 1H), 5.33 (d, *J* = 3.5 Hz, 1H), 4.92 (d, *J* = 4.2 Hz, 1H), 4.38 – 4.35 (m, 2H), 4.20 (ddd, *J* = 13.6, 9.3, 3.4 Hz, 2H), 4.07 (d, *J* = 4.6 Hz, 2H), 4.02 (dd, *J* = 7.6, 3.5 Hz, 2H), 3.98 (q, *J* = 3.8, 3.0 Hz, 1H), 3.77 – 3.74 (m, 1H), 3.72 – 3.69 (m, 3H), 3.67 (d, *J* = 4.8 Hz, 1H), 3.64 (d, *J* = 8.3 Hz, 2H), 3.54 – 3.49 (m, 2H), 3.43 (t, *J* = 8.8 Hz, 2H), 3.23 (dt, *J* = 11.0, 7.3 Hz, 3H), 3.16 (d, *J* = 6.6 Hz, 1H), 3.10 (dd, *J* = 10.0, 3.7 Hz, 2H), 2.84 (t, *J* = 7.8 Hz, 2H), 1.77 – 1.71 (m, 2H); ^{13}C NMR (125 MHz, D_2O) δ 102.10, 99.76, 97.22, 95.46, 77.88, 77.30, 76.13, 75.93, 72.61, 70.46, 71.05, 70.27, 69.68, 69.29, 68.90, 66.36, 66.16, 59.52, 57.57, 37.06, 35.70, 26.71; HRMS (ESI) Anal. Calcd for $C_{29}H_{50}N_4O_{34}S_4$ $[M-H]^-$: 1125.1117, found 1125.1102.

Compound **100:** The solution of compound **S120** (18 mg, 11.7 μmol) in DMF was subjected to *O*-sulfation, followed by saponification, *N*-acylation, azide reduction, *N*-sulfation, and hydrogenation according to the general procedures to afford compound **100** (6.8 mg, 50% over 6 steps). $[\alpha]_D^{20}$ = -2.5° (c = 0.1, H_2O); ^{1}H NMR (500 MHz, D_2O) δ 5.45 (d, *J* = 3.8 Hz, 1H), 4.97 (d, *J* = 3.7 Hz, 1H), 4.94 (d, *J* = 2.3 Hz, 1H), 4.40 (d, *J* = 7.9 Hz, 1H), 4.37 (d, *J* = 2.6 Hz, 1H), 4.24 (dd, *J* = 11.1, 3.1 Hz, 1H), 4.20 – 4.15 (m, 2H), 4.11 – 3.89 (m, 8H), 3.83 (dd, *J* = 10.2, 3.6 Hz, 1H), 3.72 – 3.54 (m, 7H), 3.46 – 3.36 (m, 2H), 3.23 – 3.16 (m, 3H), 3.08 (dd, *J* = 9.8, 3.8 Hz, 1H), 2.88 – 2.81 (m, 2H), 1.86 (s, 3H), 1.77 – 1.69 (m, 2H); ^{13}C NMR (125 MHz, D_2O) δ 101.91, 99.17, 97.41, 93.70, 77.10, 74.37, 72.61, 69.68, 69.48, 68.90, 68.70, 68.31, 66.36, 65.77, 57.77, 52.69, 36.87, 35.70, 26.52, 22.03; HRMS (ESI) Anal. Calcd for $C_{31}H_{52}N_4O_{35}S_4$ $[M-H]^-$: 1167.1222, found 1167.1267.

Compound **101:** The solution of compound **S120** (11 mg, 7.0 μmol) in DMF was subjected to *O*-sulfation, followed by saponification, azide reduction, *N*-sulfation, and hydrogenation according to the general procedures to afford compound **101** (3.9 mg, 46% over 5 steps). $[\alpha]_D^{20}$ = -3.2° (c = 0.1, H_2O); ^{1}H NMR (500 MHz, D_2O) δ 5.45 (d, *J* = 3.7 Hz, 1H), 5.34 (d, *J* = 3.5 Hz, 1H), 4.89 (d, *J* = 4.4 Hz, 1H), 4.42 (d, *J* = 7.9 Hz, 1H), 4.36 (d, *J* = 2.7 Hz, 1H), 4.26 (dd, *J* = 11.1, 2.7 Hz, 1H), 4.21 – 4.16 (m, 2H), 4.10 – 4.04 (m, 3H), 4.02 – 3.91 (m, 5H), 3.72 – 3.54 (m, 6H), 3.50 3.37 (m, 3H), 3.22 – 3.06 (m, 6H), 2.85 – 2.78 (m, 2H), 2.55 – 2.50 (m, 1H), 1.76 – 1.68 (m, 2H); ^{13}C NMR (125 MHz, D_2O) δ 101.71, 97.41, 95.46, 99.95, 77.69, 76.32, 75.93, 75.73, 72.61, 70.85, 70.66, 70.46, 69.68, 69.29, 68.90, 68.70, 66.36, 66.16, 65.77, 57.77, 35.70, 37.06, 26.71; HRMS (ESI) Anal. Calcd for $C_{29}H_{50}N_4O_{37}S_5$ $[M-4H+2Na]^{2-}$: 624.0125, found 624.0131.

Compound **102:** The solution of compound **S121** (29 mg, 18 µmol) in DMF was subjected to *O*-sulfation, followed by saponification, *N*-acylation, azide reduction, *N*-sulfation, and hydrogenation according to the general procedures to afford compound **101** (6.0 mg, 31% over 6 steps). $[\alpha]_D^{20}$ = +7.2° (*c* = 0.2, H_2O); ^1H NMR (500 MHz, D_2O) δ 5.46 (d, *J* = 3.9 Hz, 1H), 5.01 (d, *J* = 3.8 Hz, 1H), 4.73 (d, *J* = 3.4 Hz, 1H), 4.42 (d, *J* = 7.9 Hz, 1H), 4.36 (d, *J* = 2.9 Hz, 1H), 4.26 (dd, *J* = 11.1, 2.8 Hz, 1H), 4.18 (dd, *J* = 11.1, 2.4 Hz, 1H), 4.06 (d, *J* = 8.1 Hz, 3H), 4.01 – 3.95 (m, 1H), 3.92 (dd, *J* = 7.3, 3.5 Hz, 2H), 3.82 – 3.54 (m, 9H), 3.46 – 3.37 (m, 2H), 3.25 – 3.17 (m, 2H), 3.16 – 3.07 (m, 2H), 2.82 (t, *J* = 7.5 Hz, 2H), 1.83 (s, 3H), 1.76 – 1.65 (m, 2H); ^{13}C NMR (125 MHz, D_2O) δ 101.85, 101.35, 97.35, 94.13, 77.21, 76.28, 75.78, 73.91, 72.72, 70.96, 69.60, 69.26, 69.23, 68.98, 68.82, 68.78, 68.74, 66.66, 66.16, 66.16, 65.80, 57.80, 52.81, 36.92, 35.64, 35.63, 26.71, 21.83; HRMS (ESI) Anal. Calcd for $C_{31}H_{49}N_4O_{32}S_3^{3-}$ $[M-3H]^{3-}$: 361.7169, found 361.7167.

Compound **103:** The solution of compound **S121** (18 mg, 10.8 µmol) in DMF was subjected to *O*-sulfation, followed by saponification, azide reduction, *N*-sulfation, and hydrogenation according to the general procedures to afford compound **101** (5.2 mg, 43% over 5 steps). $[\alpha]_D^{20}$ = +1.1° (c = 0.1, H_2O); ^1H NMR (500 MHz, D_2O) δ 5.47 (d, *J* = 3.7 Hz, 1H), 5.22 (d, *J* = 3.7 Hz, 1H), 4.76 (s, 1H), 4.45 – 4.40 (m, 1H), 4.36 – 4.26 (m, 2H), 4.20 (d, *J* = 11.1 Hz, 1H), 4.12 – 3.97 (m, 5H), 3.94 – 3.85 (m, 2H), 3.73 – 3.38 (m, 9H), 3.25 – 3.07 (m, 4H), 2.83 (td, *J* = 7.8, 1.7 Hz, 1H), 1.80 – 1.67 (m, 2H); ^{13}C NMR (125 MHz, D_2O) δ 101.71, 101.12, 97.41, 95.27, 76.32, 72.81, 74.56, 69.68, 69.48, 68.90, 68.70, 68.12, 66.56, 65.97, 59.92, 57.57, 36.87, 35.70, 26.71; HRMS (ESI) Anal. Calcd for $C_{29}H_{50}N_4O_{34}S_4$ $[M-H]^-$: 1125.1117, found 1125.1102.

Compound **S101:** The solution of compound **31** (67 mg, 33 µmol) in pyridine was subjected to silyl-group deprotection according to the general procedure to afford compound **S101** (57.7 mg, 28.9 µmol) in 88% yield. ^1H NMR (500 MHz, $CDCl_3$) δ 8.47 (d, *J* = 7.7 Hz, 1H), 8.01 – 7.90 (m, 4H), 7.66 (dd, *J* = 8.4, 1.5 Hz, 1H), 7.64 – 7.56 (m, 2H), 7.41 – 7.18 (m, 22H), 7.17 – 7.07 (m, 4H), 6.70 (t, *J* = 6.1 Hz, 1H), 5.66 (d, *J* = 9.6 Hz, 1H), 5.24 – 5.17 (m, 2H), 5.06 (dd, *J* = 21.8, 10.1 Hz, 4H), 4.98 (d, *J* = 6.0 Hz, 2H), 4.93 (d, *J* = 11.4 Hz, 1H), 4.85 (dd, *J* = 9.5, 8.2 Hz, 1H), 4.79 (d, *J* = 3.4 Hz, 1H), 4.75 (d, *J* = 11.0 Hz, 1H), 4.64 – 4.50 (m, 5H), 4.34 – 4.28 (m, 1H), 4.27 – 4.16 (m, 4H), 4.09 – 3.95 (m, 4H), 3.92 – 3.86 (m, 3H), 3.83 (d, *J* = 6.4 Hz, 2H), 3.80 (s, 3H), 3.78 – 3.70 (m, 3H), 3.70 – 3.54 (m, 4H), 3.51 (s, 3H), 3.07 – 2.95 (m, 4H), 2.73 (dd, *J* = 9.1, 3.9 Hz, 1H), 2.60 – 2.52 (m, 2H), 2.48 (t, *J* = 9.5 Hz, 1H), 2.35 (dd, *J* = 12.9, 3.6 Hz, 1H), 2.15 (s, 3H), 1.93 (s, 3H), 1.92 (s, 3H), 1.37 – 1.31 (m, 2H), 1.19 (s, 3H); ^{13}C NMR (125 MHz, $CDCl_3$) δ 209.57, 172.24, 170.23, 170.11, 168.91, 168.87, 168.63, 167.40, 156.60, 138.52, 137.98, 137.00, 136.62, 135.29, 133.40, 133.16, 128.84, 128.62, 128.48, 128.46, 128.31, 128.29, 128.22, 128.05, 128.02, 127.97, 127.84, 127.82, 127.61, 127.56, 127.36, 126.86, 126.71, 126.47, 99.94, 99.87, 98.42, 98.28, 80.44, 78.98, 77.58, 76.13, 75.33, 75.30, 74.89, 73.86, 73.68, 73.45, 73.01, 72.88, 72.72, 72.18, 71.18, 69.71, 66.71, 66.57, 66.40, 65.81, 61.57, 53.67, 52.97, 52.54, 51.71, 38.44, 37.43, 35.33, 29.81, 29.11, 27.96, 22.13, 20.97, 20.86; ESI -MS Anal. Calcd for $C_{91}H_{103}F_3N_4O_{30}Na$ $[M+Na]^+$: 1811.65, found 1811.63.

Compound **S102:** The solution of compound **31** (70 mg, 34.5 µmol) in pyridine:acetic acid (1.0 mL, 3:2) was subjected for Lev-group deprotection according to the general procedure to afford compound **S103** (60 mg, 31.1 µmol) in 90% yield. ^{1}H NMR (500 MHz, $CDCl_3$) δ 8.04 (dt, *J* = 8.4, 1.7 Hz, 1H), 7.95 – 7.87 (m, 4H), 7.71 – 7.67 (m, 4H), 7.61 – 7.53 (m, 4H), 7.45 – 7.37 (m, 8H), 7.32 (q, *J* = 6.3, 5.8 Hz, 14H), 7.24 – 7.21 (m, 5H), 7.17 – 7.15 (m, 2H), 7.08 (dd, *J* = 7.9, 1.7 Hz, 2H), 6.93 (t, *J* = 6.4 Hz, 1H), 5.93 (d, *J* = 9.7 Hz, 1H), 5.46 (t, *J* = 9.5 Hz, 1H), 5.21 (t, *J* = 6.7 Hz, 1H), 5.12 – 5.02 (m, 5H), 4.99 (s, 1H), 4.88 – 4.82 (m, 3H), 4.73 (d, *J* = 2.6 Hz, 1H), 4.67 (d, *J* = 11.2 Hz, 1H), 4.60 – 4.53 (m, 4H), 4.50 (dd, *J* = 3.9, 2.2 Hz, 1H), 4.47 (d, *J* = 12.5 Hz, 1H), 4.40 (d, *J* = 11.7 Hz, 1H), 4.37 – 4.33 (m, 1H), 4.22 – 4.17 (m, 3H), 4.07 – 4.00 (m, 3H), 3.94 – 3.87 (m, 7H), 3.85 – 3.82 (m, 1H), 3.81 – 3.72 (m, 10H), 3.68 – 3.61 (m, 2H), 3.55 – 3.47 (m, 3H), 3.31 (s, 3H), 3.06 (ddt, *J* = 36.1, 21.4, 6.4 Hz, 4H), 2.40 – 2.34 (m, 1H), 2.03 (s, 3H), 1.93 (s, 3H), 1.51 – 1.45 (m, 1H), 1.38 – 1.33 (m, 1H), 1.09 (s, 9H). ^{13}C NMR (125 MHz, $CDCl_3$) δ 135.80, 129.75, 128.28, 128.09, 127.99, 127.79, 126.82, 100.45, 100.35, 99.86, 94.98, 77.01, 74.86, 70.47, 68.32, 66.95, 66.17, 66.07, 64.02, 62.46, 52.89, 52.69, 52.60, 37.26, 35.11, 29.65, 26.81, 22.13, 21.05, 20.66. ESI-MS Anal. Calcd for $C_{102}H_{115}F_3N_4O_{28}Si$ $[M+H]^+$: 1929.75, found 1929.72.

Compound **S103:** The solution of compound **31** (62 mg, 30 µmol) in DCM:water (3.3 mL, 10:1) was subjected to Nap-group deprotection according to the general procedure to afford compound **S103** (55 mg, 29 µmol) in 97% yield. ^{1}H NMR (500 MHz, $CDCl_3$) δ 8.44 (d, *J* = 7.8 Hz, 1H), 7.64 (dt, *J* = 18.4, 9.2 Hz, 4H), 7.43 – 7.28 (m, 20H), 7.28 – 7.22 (m, 7H), 7.22 – 7.13 (m, 4H), 6.73 – 6.67 (m, 1H), 5.95 (d, *J* = 9.2 Hz, 1H), 5.37 (t, *J* = 9.4 Hz, 1H), 5.20 – 5.04 (m, 7H), 4.98 (d, *J* = 7.0 Hz, 2H), 4.83 (d, *J* = 8.2 Hz, 1H), 4.77 (d, *J* = 11.2 Hz, 1H), 4.68 (d, *J* = 10.9 Hz, 1H), 4.64 – 4.50 (m, 5H), 4.40 – 4.34 (m, 1H), 4.24 – 4.06 (m, 6H), 4.01 – 3.93 (m, 3H), 3.88 (t, *J* = 7.5 Hz, 2H), 3.85 – 3.57 (m, 12H), 3.26 (d, *J* = 12.2 Hz, 3H), 3.01 (dd, *J* = 20.2, 9.0 Hz, 5H), 2.59 (dd, *J* = 22.0, 7.0 Hz, 2H), 2.42 – 2.36 (m, 1H), 2.05 (s, 3H), 1.94 (s, 3H), 1.91 (s, 3H), 1.59 (s, 3H), 1.04 (s, 9H); ^{13}C NMR (125 MHz, $CDCl_3$) δ 209.84, 172.24, 169.99, 169.18, 169.03, 168.81, 168.75, 167.81, 157.77, 157.47, 156.62, 138.64, 138.04, 136.96, 136.74, 136.59, 135.74, 135.72, 133.16, 133.06, 129.66, 129.62, 129.02, 128.70, 128.60, 128.47, 128.40, 128.29, 128.21, 128.20, 128.05, 128.00, 127.88, 127.85, 127.73, 127.63, 127.61, 127.27, 127.02, 126.77, 125.28, 100.23, 98.39, 98.16, 98.03, 80.61, 77.24, 77.15, 77.12, 75.53, 75.35, 74.86, 74.01, 73.54, 73.03, 72.79, 72.40, 72.01, 70.98, 68.75, 66.74, 66.56, 65.85, 62.25, 60.27, 53.90, 52.50, 52.37, 51.31, 38.54, 37.40, 35.29, 29.82, 29.70, 29.14, 28.03, 26.75, 22.70, 20.92, 20.88, 19.20; ESI-MS Anal. Calcd for $C_{96}H_{114}F_3N_4O_{30}Si^+$ $[M+H]^+$: 1887.72, found 1887.70.

Compound **S104:** The solution of compound **S102** (60 mg, 31.1 µmol) in DCM:water was subjected to Nap-group deprotection according to the general procedure to afford compound **S104** (55 mg, 29.1 µmol) in 93.5% yield. ^{1}H NMR (500 MHz, $CDCl_3$) δ 7.67 – 7.61 (m, 4H), 7.56 (d, *J* = 8.8 Hz, 1H), 7.39 (td, *J* = 7.1, 4.9 Hz, 3H), 7.35 – 7.28 (m, 18H), 7.25 – 7.19 (m, 8H), 6.90 (t, *J* = 6.4 Hz, 1H), 5.97 (d, *J* = 9.3 Hz, 1H), 5.35 (t, *J* = 9.3 Hz, 1H), 5.21 – 5.17 (m, 2H), 5.12 (d, *J* = 3.5 Hz, 1H), 5.04 (s, 1H), 5.00 (dd, *J* = 13.3, 10.4 Hz, 3H), 4.93 (d, *J* = 3.5 Hz, 1H), 4.75 (dd, *J* = 17.4, 5.3 Hz, 2H), 4.67 (dd, *J* = 13.0, 11.0 Hz, 2H), 4.57 – 4.53 (m, 3H), 4.51 – 4.48 (m, 1H),

4.36 (dt, *J* = 10.1, 5.0 Hz, 1H), 4.19 (d, *J* = 15.6 Hz, 1H), 4.16 – 4.09 (m, 3H), 4.06 (d, *J* = 15.7 Hz, 1H), 3.97 (d, *J* = 8.3 Hz, 1H), 3.93 (d, *J* = 4.5 Hz, 2H), 3.79 (t, *J* = 4.4 Hz, 2H), 3.76 (s, 3H), 3.73 – 3.69 (m, 2H), 3.67 (d, *J* = 10.0 Hz, 2H), 3.60 – 3.52 (m, 2H), 3.48 – 3.44 (m, 1H), 3.32 (s, 3H), 3.15 (dt, *J* = 12.0, 6.2 Hz, 2H), 3.09 – 2.99 (m, 2H), 2.03 (s, 2H), 1.50 (dt, *J* = 12.8, 6.4 Hz, 1H), 1.39 (dd, *J* = 13.3, 6.8 Hz, 1H), 1.04 (s, 7H). ^{13}C NMR (125 MHz, $CDCl_3$) δ 135.71, 129.65, 128.28, 100.55, 98.20, 95.27, 81.02, 77.30, 76.52, 75.16, 74.96, 72.81, 72.23, 71.05, 68.71, 72.81, 68.32, 66.95, 66.76, 62.26, 60.12, 52.89, 52.50, 51.33, 37.26, 35.11, 29.45, 26.72, 22.61, 20.86. ESI-MS Anal. Calcd for $C_{91}H_{107}F_3N_4O_{28}Si$ $[M+Na]^+$: 1811.67, found 1811.61.

Compound **S105:** The solution of compound S**102** (40 mg, 20.7 µmol) in pyridine was subjected to silyl group deprotection according to the general procedure to afford compound **S105** (30 mg, 17.7 µmol) in 85.5% yield. ^{1}H NMR (500 MHz, $CDCl_3$) δ 8.00 – 7.90 (m, 4H), 7.65 (dd, *J* = 8.4, 1.7 Hz, 1H), 7.60 – 7.57 (m, 2H), 7.39 (d, *J* = 8.7 Hz, 1H), 7.36 – 7.28 (m, 14H), 7.25 – 7.19 (m, 6H), 7.16 (d, *J* = 7.5 Hz, 2H), 7.09 (dd, *J* = 7.0, 2.3 Hz, 2H), 6.90 (t, *J* = 6.3 Hz, 1H), 5.67 (d, *J* = 9.6 Hz, 1H), 5.19 (t, *J* = 6.6 Hz, 1H), 5.10 (d, *J* = 12.4 Hz, 1H), 5.07 – 5.01 (m, 4H), 4.99 (d, *J* = 6.0 Hz, 1H), 4.84 (dd, *J* = 10.5, 6.9 Hz, 2H), 4.73 (dd, *J* = 7.3, 2.9 Hz, 2H), 4.66 (d, *J* = 11.2 Hz, 1H), 4.62 – 4.53 (m, 4H), 4.48 (d, *J* = 12.4 Hz, 1H), 4.40 (d, *J* = 11.7 Hz, 1H), 4.29 (td, *J* = 10.1, 3.4 Hz, 1H), 4.24 – 4.16 (m, 3H), 4.07 – 4.02 (m, 3H), 4.01 – 3.97 (m, 1H), 3.93 – 3.86 (m, 4H), 3.83 (d, *J* = 10.8 Hz, 1H), 3.79 – 3.76 (m, 1H), 3.74 (s, 3H), 3.72 (d, *J* = 2.8 Hz, 1H), 3.70 – 3.64 (m, 3H), 3.54 (d, *J* = 11.5 Hz, 2H), 3.51 (s, 3H), 3.47 (dd, *J* = 8.9, 1.8 Hz, 1H), 3.14 – 2.97 (m, 4H), 2.80 (dd, *J* = 9.7, 4.6 Hz, 1H), 2.37 (t, *J* = 9.6 Hz, 1H), 2.14 (s, 3H), 1.94 (s, 3H), 1.47 (dt, *J* = 13.4, 6.9 Hz, 1H), 1.36 (td, *J* = 13.8, 12.3, 5.2 Hz, 1H), 1.13 (s, 3H). ^{13}C NMR (125 MHz, $CDCl_3$) δ 170.27, 170.19, 169.94, 169.20, 168.74, 167.34, 157.29, 138.19, 137.98, 136.91, 136.50, 136.15, 135.18, 133.40, 133.22, 128.87, 128.70, 128.57, 128.55, 128.47, 128.36, 128.25, 128.07, 127.90, 127.84, 127.76, 127.69, 127.64, 127.57, 127.39, 126.80, 126.56, 100.44, 100.21, 99.98, 95.01, 80.39, 79.03, 77.70, 77.26, 75.30, 74.66, 73.85, 72.93, 72.80, 72.19, 70.97, 69.65, 68.24, 66.90, 66.60, 66.16, 66.06, 61.58, 52.97, 52.83, 52.59, 51.76, 37.32, 34.98, 29.49, 22.01, 20.97, 20.63. HRMS (ESI) Anal. Calcd for $C_{86}H_{97}F_3N_4O_{28}$ $[M+Na]^+$: 1691.6314, found 1691.6457.

Compound **S106:** The solution of compound **S103** (38.5 mg, 21 µmol) in pyridine:acetic acid (1.0 mL, 3:2) was subjected for Lev-group deprotection followed by silyl-group deprotection according to the general procedures to afford compound **S106** (26 mg, 16.7 µmol) in 80% yield over 2 steps. ^{1}H NMR (500 MHz, $CDCl_3$) δ 7.44 – 7.27 (m, 16H), 7.27 – 7.15 (m, 9H), 6.91 (t, *J* = 6.4 Hz, 1H), 5.77 (d, *J* = 9.3 Hz, 1H), 5.17 (t, *J* = 8.3 Hz, 1H), 5.14 – 5.09 (m, 1H), 5.09 – 4.91 (m, 7H), 4.76 (dd, *J* = 13.0, 5.0 Hz, 2H), 4.69 (dd, *J* = 15.0, 9.1 Hz, 2H), 4.63 – 4.47 (m, 5H), 4.36 – 4.28 (m, 1H), 4.23 – 4.06 (m, 5H), 4.01 (d, *J* = 8.4 Hz, 1H), 3.98 – 3.91 (m, 2H), 3.86 – 3.66 (m, 10H), 3.62 – 3.45 (m, 7H), 3.22 (dd, *J* = 13.4, 6.7 Hz, 2H), 3.15 – 2.98 (m, 3H), 2.66 (dd, *J* = 9.3, 4.8 Hz, 1H), 2.07 (s, 3H), 2.03 (s, 3H), 1.46 (s, 3H); ^{13}C NMR (125 MHz, $CDCl_3$) δ 170.36, 170.05, 169.75, 169.04, 169.00, 167.82, 138.17, 137.99, 136.85, 136.66, 129.03, 129.02, 128.82, 128.69, 128.59, 128.46, 128.37, 128.31, 128.22, 127.87, 127.82, 127.80, 127.75, 127.66, 127.44, 125.29, 100.57, 98.85, 95.50, 80.86, 77.38, 77.22, 77.13, 76.60, 75.65, 74.92, 74.84, 73.12, 72.77, 72.61,

72.22, 71.89, 70.03, 67.00, 66.26, 61.32, 60.10, 52.89, 52.78, 51.79, 37.30, 34.90, 29.44, 22.51, 21.46, 20.87, 20.71; ESI-MS Anal. Calcd for $C_{75}H_{90}F_3N_4O_{28}$ $[M+H]^+$: 1551.57, found 1551.57.

Compound **S107:** The solution of compound **26** (47 mg, 24 µmol) in DMF was subjected to *O*-sulfation followed by silyl group deprotection and saponification according to the general procedures to afford compound **S107** (25 mg, 16 µmol) in 67% yield over 3 steps. ^{1}H NMR (500 MHz, CD_3OD) δ 7.95 – 7.78 (m, 4H), 7.56 (d, *J* = 8.6 Hz, 1H), 7.51 – 7.14 (m, 27H), 5.50 (d, *J* = 3.2 Hz, 1H), 5.37 (s, 1H), 5.14 – 5.00 (m, 4H), 4.90 (s, 1H), 4.84 – 4.62 (m, 6H), 4.53 (d, *J* = 9.8 Hz, 1H), 4.49 – 4.38 (m, 3H), 4.32 – 4.18 (m, 4H), 4.13 – 3.98 (m, 4H), 3.92 (t, *J* = 9.5 Hz, 2H), 3.88 – 3.78 (m, 4H), 3.74 – 3.58 (m, 3H), 3.56 – 3.49 (m, 1H), 3.19 (dd, *J* = 10.3, 3.2 Hz, 2H), 3.11 – 2.90 (m, 4H), 1.50 – 1.39 (m, 2H), ^{13}C NMR (125 MHz, CD_3OD) δ 174.57, 170.77, 157.50, 138.55, 138.10, 137.78, 136.97, 135.44, 133.41, 133.13, 128.86, 128.40, 128.16, 128.10, 128.06, 127.94, 127.85, 127.83, 127.72, 127.62, 127.55, 127.46, 127.42, 127.20, 126.96, 126.88, 126.10, 125.72, 125.49, 101.28, 100.43, 97.04, 91.16, 83.42, 80.04, 79.63, 77.44, 74.80, 74.67, 74.09, 73.01, 72.56, 72.28, 71.21, 67.00, 66.00, 65.65, 63.16, 60.65, 37.59, 35.63, 29.26; HRMS (ESI) Anal. Calcd for $C_{76}H_{84}N_6O_{27}S^{2-}$ $[M-2H]^{2-}$: 772.2558, found 772.2415.

Compound **S108:** The solution of compound **26** (75 mg, 38.1 µmol) in pyridine:acetic acid was subjected to Lev-group deprotection according to the general procedure to afford compound **S103** (70 mg, 37.4 µmol) in 98% yield. ^{1}H NMR (500 MHz, $CDCl_3$) δ 7.94 – 7.85 (m, 4H), 7.69 – 7.63 (m, 5H), 7.53 (ddd, *J* = 22.2, 8.0, 2.8 Hz, 3H), 7.40 – 7.30 (m, 27H), 7.26 – 7.17 (m, 7H), 6.93 (t, *J* = 6.4 Hz, 1H), 5.49 (d, *J* = 3.6 Hz, 1H), 5.33 (t, *J* = 9.6 Hz, 1H), 5.15 (t, *J* = 6.6 Hz, 1H), 5.09 – 4.97 (m, 4H), 4.91 (d, *J* = 11.9 Hz, 2H), 4.83 (d, *J* = 11.0 Hz, 1H), 4.75 (t, *J* = 2.2 Hz, 1H), 4.71 (d, *J* = 5.1 Hz, 1H), 4.69 (d, *J* = 2.1 Hz, 1H), 4.67 – 4.62 (m, 3H), 4.57 (d, *J* = 11.3 Hz, 1H), 4.44 (t, *J* = 10.6 Hz, 2H), 4.26 (ddd, *J* = 15.5, 7.9, 3.4 Hz, 1H), 4.20 – 4.15 (m, 1H), 4.14 – 4.07 (m, 3H), 4.00 – 3.97 (m, 1H), 3.95 (t, *J* = 6.8 Hz, 1H), 3.87 (t, *J* = 9.8 Hz, 1H), 3.80 – 3.74 (m, 2H), 3.72 – 3.57 (m, 9H), 3.49 (ddd, *J* = 12.7, 6.8, 3.1 Hz, 2H), 3.39 (dd, *J* = 10.4, 3.5 Hz, 1H), 3.29 (d, *J* = 9.7 Hz, 4H), 3.15 – 2.96 (m, 4H), 1.89 (s, 3H), 1.52 – 1.46 (m, 1H), 1.36 (dt, *J* = 13.5, 6.9 Hz, 1H), 1.04 (s, 9H). ^{13}C NMR (125 MHz, $CDCl_3$) δ 169.45, 169.33, 169.05, 168.27, 157.33, 138.40, 138.23, 137.51, 136.87, 136.12, 135.78, 135.75, 134.78, 133.27, 133.21, 133.13, 129.65, 129.57, 128.73, 128.61, 128.57, 128.54, 128.47, 128.43, 128.30, 128.26, 128.08, 128.03, 127.92, 127.88, 127.84, 127.68, 127.65, 127.60, 127.43, 127.39, 127.32, 126.40, 126.28, 126.22, 102.69, 100.50, 97.24, 96.33, 83.87, 77.68, 77.52, 77.32, 74.87, 74.59, 74.33, 73.90, 73.32, 72.78, 72.28, 71.41, 70.87, 69.55, 67.75, 67.66, 66.94, 66.83, 65.94, 63.05, 61.74, 52.98, 52.58, 52.17, 37.37, 35.11, 29.46, 26.76, 20.87, 19.23. ESI-MS Anal. Calcd for $C_{98}H_{109}F_3N_6O_{26}Si$ $[M+H]^+$: 1871.72, found 1871.70.

Compound **S109:** The solution of compound **26** (165 mg, 83.7 µmol) in DCM:water was subjected to Nap-group deprotection according to the general procedure to afford compound **S109** (150 mg, 82 µmol) in 98% yield. ^{1}H NMR (500 MHz, $CDCl_3$) δ 8.42 (d, *J* = 8.4 Hz, 1H), 7.70 – 7.59 (m, 4H), 7.43 – 7.29 (m, 25H), 7.26 – 7.12 (m, 6H), 6.70 (t, *J* = 7.2 Hz, 1H), 5.52 (d, *J* = 3.6 Hz, 1H),

5.31 (dd, J = 10.5, 8.8 Hz, 1H), 5.21 (t, J = 6.8 Hz, 1H), 5.12 (d, J = 3.7 Hz, 1H), 5.05 (dd, J = 12.3, 7.8 Hz, 3H), 5.00 (d, J = 10.2 Hz, 1H), 4.95 (d, J = 11.5 Hz, 1H), 4.87 (d, J = 11.0 Hz, 1H), 4.79 (dd, J = 21.1, 11.0 Hz, 2H), 4.65 (d, J = 7.4 Hz, 2H), 4.60 (dd, J = 11.0, 5.2 Hz, 2H), 4.26 – 4.13 (m, 4H), 4.06 – 3.91 (m, 7H), 3.89 – 3.83 (m, 2H), 3.80 (d, J = 5.1 Hz, 3H), 3.76 – 3.70 (m, 2H), 3.66 – 3.54 (m, 4H), 3.47 (d, J = 10.0 Hz, 1H), 3.38 (dd, J = 10.4, 3.6 Hz, 2H), 3.26 (s, 3H), 3.01 (dd, J = 16.1, 11.0 Hz, 4H), 2.66 – 2.53 (m, 2H), 2.40 – 2.33 (m, 2H), 1.97 (s, 3H), 1.86 (s, 3H), 1.02 (s, 9H); ^{13}C NMR (125 MHz, $CDCl_3$) δ 209.43, 168.98, 168.35, 138.29, 138.20, 137.44, 135.77, 135.71, 133.18, 133.12, 129.61, 129.54, 128.63, 128.54, 128.50, 128.33, 128.28, 128.24, 128.06, 128.01, 127.97, 127.85, 127.67, 127.61, 127.56, 127.38, 126.76, 103.04, 98.45, 98.12, 97.31, 83.78, 77.51, 77.22, 74.82, 74.72, 74.53, 74.41, 72.86, 72.51, 70.91, 69.47, 66.57, 62.93, 61.72, 60.76, 53.92, 52.58, 52.22, 38.62, 37.46, 35.37, 29.78, 29.12, 28.10, 26.72, 20.82, 19.22; ESI-MS Anal. Calcd for $C_{92}H_{108}F_3N_6O_{28}Si^+$ [M+H]$^+$: 1829.69, found 1829.67.

Compound **S110:** The solution of compound **26** (60 mg, 30.5 µmol) in pyridine was subjected to silyl group deprotection according to the general procedure to afford compound **S110** (50 mg, 28.9 µmol) in 94.9% yield. ^{1}H NMR (500 MHz, $CDCl_3$) δ 8.47 (d, J = 7.6 Hz, 1H), 7.93 – 7.82 (m, 4H), 7.57 – 7.46 (m, 3H), 7.36 – 7.25 (m, 23H), 7.19 (dd, J = 21.2, 7.3 Hz, 3H), 6.66 (t, J = 6.4 Hz, 1H), 5.50 (d, J = 3.8 Hz, 1H), 5.23 – 5.14 (m, 2H), 5.06 (s, 2H), 5.02 – 4.95 (m, 3H), 4.91 – 4.83 (m, 3H), 4.75 – 4.67 (m, 4H), 4.61 – 4.55 (m, 3H), 4.45 (d, J = 7.9 Hz, 1H), 4.24 – 4.11 (m, 5H), 4.06 (dd, J = 11.5, 2.2 Hz, 1H), 4.01 – 3.94 (m, 3H), 3.89 (t, J = 9.7 Hz, 1H), 3.80 (ddd, J = 25.4, 10.5, 3.9 Hz, 4H), 3.67 (s, 3H), 3.56 (dd, J = 13.1, 5.5 Hz, 1H), 3.47 – 3.40 (m, 5H), 3.36 (dd, J = 10.3, 3.8 Hz, 1H), 3.25 (t, J = 8.8 Hz, 1H), 3.00 (dt, J = 21.6, 6.4 Hz, 5H), 2.77 (d, J = 3.4 Hz, 1H), 2.60 – 2.49 (m, 3H), 2.34 (dt, J = 16.0, 4.2 Hz, 1H), 1.98 (s, 3H), 1.92 (s, 3H), 1.36 – 1.29 (m, 2H). ^{13}C NMR (125 MHz, $CDCl_3$) δ 209.54, 172.10, 170.85, 168.93, 168.59, 168.42, 156.59, 138.58, 138.33, 137.55, 137.00, 136.64, 134.87, 133.28, 133.08, 128.64, 128.55, 128.51, 128.49, 128.41, 128.32, 128.26, 128.12, 128.05, 128.03, 127.83, 127.79, 127.64, 127.55, 127.41, 127.33, 126.73, 126.49, 126.37, 126.15, 102.47, 99.28, 98.03, 97.56, 83.62, 77.50, 77.39, 76.94, 75.78, 75.38, 75.16, 75.01, 74.79, 74.47, 74.06, 73.84, 73.22, 72.90, 71.35, 70.86, 70.58, 67.32, 66.95, 66.70, 66.54, 65.78, 63.01, 60.77, 53.99, 52.44, 38.36, 37.44, 35.34, 29.78, 29.70, 29.01, 28.11, 20.79. ESI-MS Anal. Calcd for $C_{87}H_{97}F_3N_6O_{28}$ [M+Na]$^+$: 1753.62, found 1753.61.

Compound **S111:** The solution of compound **S108** (190 mg, 0.10 mmol) in DCM:water mixture was subjected to Nap deprotection according to the general procedure to afford compound **S111** (150 mg, 0.087 mmol) in 87% yield. ^{1}H NMR (500 MHz, $CDCl_3$) δ 7.70 – 7.59 (m, 5H), 7.41 – 7.17 (m, 30H), 6.89 (t, J = 6.2 Hz, 1H), 5.51 (d, J = 3.4 Hz, 1H), 5.30 (t, J = 9.7 Hz, 1H), 5.11 – 4.98 (m, 4H), 4.90 (dd, J = 23.4, 7.1 Hz, 2H), 4.81 (dd, J = 10.6, 6.2 Hz, 3H), 4.76 (s, 1H), 4.69 (d, J = 11.5 Hz, 1H), 4.64 (t, J = 7.4 Hz, 1H), 4.63 – 4.57 (m, 2H), 4.53 (d, J = 11.5 Hz, 1H), 4.26 – 4.20 (m, 1H), 4.15 (dd, J = 25.0, 7.0 Hz, 3H), 4.05 – 3.95 (m, 4H), 3.91 (dd, J = 12.6, 7.8 Hz, 2H), 3.85 (t, J = 9.8 Hz, 1H), 3.77 (d, J = 14.2 Hz, 4H), 3.75 – 3.52 (m, 8H), 3.45 (d, J = 10.0 Hz, 1H), 3.40 – 3.34 (m, 4H), 3.31 – 3.20 (m, 3H), 3.10 (dd, J = 14.1, 6.0 Hz, 1H), 3.02 (d, J = 5.4 Hz, 1H), 1.86 (s, 3H), 1.02 (s, 9H); ^{13}C NMR (125 MHz, $CDCl_3$) δ 169.39, 169.13, 168.97, 168.49,

157.49, 138.05, 137.44, 136.77, 135.74, 135.71, 133.15, 133.04, 129.62, 129.57, 128.72, 128.61, 128.55, 128.50, 128.40, 128.34, 128.25, 127.97, 127.85, 127.73, 127.68, 127.61, 127.58, 102.73, 100.57, 97.22, 96.33, 83.69, 78.02, 77.50, 77.22, 75.09, 74.76, 74.52, 74.44, 74.07, 73.05, 72.75, 72.37, 72.16, 70.95, 69.44, 67.67, 67.04, 66.00, 62.90, 61.71, 60.61, 53.34, 52.69, 52.37, 37.28, 34.93, 29.71, 29.37, 26.72, 20.82, 19.20; MS-ESI Anal. Calcd for $C_{87}H_{102}F_3N_6O_{26}Si^+$ $[M+NH_4]^+$: 1748.68, found 1748.70.

Compound **S113:** The solution of compound **S109** (65 mg, 35.5 µmol) in pyridine: acetic acid was subjected for Lev-group deprotection followed by silyl group deprotection according to the general procedures to afford compound **S113** (44 mg, 29 µmol) in 82% yield over 2 steps. ^{1}H NMR (500 MHz, $CDCl_3$) δ 7.86 (d, *J* = 8.3 Hz, 1H), 7.42 – 7.13 (m, 25H), 6.88 (t, *J* = 5.9 Hz, 1H), 5.54 (d, *J* = 3.7 Hz, 1H), 5.12 (t, *J* = 6.1 Hz, 1H), 5.04 (q, *J* = 12.4 Hz, 3H), 4.96 (d, *J* = 10.9 Hz, 1H), 4.93 – 4.80 (m, 5H), 4.75 (s, 1H), 4.67 (dd, *J* = 15.5, 7.9 Hz, 3H), 4.57 (d, *J* = 11.2 Hz, 1H), 4.53 (d, *J* = 11.4 Hz, 1H), 4.28 – 4.18 (m, 2H), 4.13 (d, *J* = 10.0 Hz, 2H), 4.03 (dt, *J* = 18.7, 9.1 Hz, 4H), 3.94 (d, *J* = 9.4 Hz, 2H), 3.92 – 3.86 (m, 1H), 3.83 – 3.71 (m, 6H), 3.70 – 3.62 (m, 3H), 3.56 (d, *J* = 17.2 Hz, 5H), 3.44 (t, *J* = 11.1 Hz, 2H), 3.34 (dd, *J* = 10.3, 3.7 Hz, 1H), 3.19 (d, *J* = 7.0 Hz, 2H), 3.12 – 2.97 (m, 3H), 2.70 (broad, 1H), 2.62 (broad, 1H), 1.96 (s, 3H); ^{13}C NMR (125 MHz, $CDCl_3$) δ 170.78, 169.51, 169.22, 168.58, 157.36, 138.00, 137.70, 137.50, 136.81, 136.02, 129.03, 128.72, 128.59, 128.52, 128.43, 128.39, 128.30, 128.22, 127.99, 127.87, 127.85, 127.81, 127.67, 127.63, 102.99, 100.42, 97.44, 96.38, 83.72, 77.91, 77.22, 75.01, 74.95, 74.88, 74.69, 74.49, 73.08, 72.76, 72.21, 70.73, 67.64, 66.97, 66.01, 62.89, 60.79, 53.28, 52.72, 52.62, 37.35, 35.04, 29.43, 20.75; HRMS (ESI) Anal. Calcd for $C_{71}H_{84}F_3N_6O_{26}$ $[M+Na]^+$: 1515.5201, found 1515.5248.

Compound **S114:** The solution of compound **S109** (30 mg, 16.4 µmol) in pyridine was subjected for silyl group deprotection according to the general procedure to afford compound **S114** (24 mg, 15.0 µmol) in 82% yield. ^{1}H NMR (500 MHz, $CDCl_3$) δ 8.48 (d, *J* = 7.9 Hz, 1H), 7.41 – 7.27 (m, 21H), 7.26 – 7.14 (m, 4H), 6.71 (t, *J* = 6.2 Hz, 1H), 5.56 (d, *J* = 3.8 Hz, 1H), 5.22 (t, *J* = 6.1 Hz, 1H), 5.12 (d, *J* = 3.7 Hz, 1H), 5.09 – 4.94 (m, 6H), 4.91 – 4.82 (m, 3H), 4.77 (d, *J* = 11.0 Hz, 1H), 4.66 (dd, *J* = 9.5, 6.7 Hz, 3H), 4.59 (d, *J* = 11.1 Hz, 1H), 4.26 – 4.14 (m, 4H), 4.10 – 4.04 (m, 1H), 4.03 – 3.92 (m, 6H), 3.91 – 3.84 (m, 2H), 3.80 (d, *J* = 7.4 Hz, 4H), 3.77 – 3.70 (m, 2H), 3.68 – 3.62 (m, 1H), 3.57 (d, *J* = 4.1 Hz, 2H), 3.46 (s, 3H), 3.45 – 3.39 (m, 2H), 3.35 (dd, *J* = 10.0, 3.5 Hz, 2H), 3.07 – 2.94 (m, 4H), 2.64 – 2.52 (m, 3H), 1.97 (s, 3H), 1.95 (s, 3H); ^{13}C NMR (125 MHz, $CDCl_3$) δ 209.60, 172.12, 170.87, 168.94, 168.89, 168.48, 156.63, 138.37, 138.18, 137.51, 136.95, 136.59, 129.03, 128.63, 128.52, 128.49, 128.39, 128.30, 128.22, 128.07, 128.01, 128.00, 127.85, 127.80, 127.66, 127.61, 127.40, 126.68, 125.29, 114.76, 103.12, 98.69, 98.13, 97.59, 83.80, 77.54, 77.24, 75.36, 75.19, 75.06, 75.03, 74.99, 74.64, 73.66, 73.49, 72.84, 72.53, 70.80, 70.64, 67.06, 66.67, 66.57, 65.89, 62.90, 60.76, 60.69, 53.96, 52.59, 52.51, 38.44, 37.46, 35.37, 29.78, 29.09, 28.12, 20.77; HRMS (ESI) Anal. Calcd for $C_{76}H_{90}F_3N_6O_{28}^+$ $[M+H]^+$: 1591.5750, found 1591.5637.

Compound **S115:** The solution of compound **29** (50.0 mg, 25.0 µmol) in pyridine was subjected for silyl group deprotection according to the general procedure to afford compound **S115** (41.0 mg, 23.0 µmol) in 92% yield. ^{1}H NMR (500 MHz, $CDCl_3$) δ 8.46 (d, *J* = 7.7 Hz, 1H), 8.01 – 7.86 (m, 4H), 7.62 – 7.51 (m, 3H), 7.42 – 7.22 (m, 18H), 7.23 – 7.08 (m, 5H), 6.68 (t, *J* = 6.2 Hz, 1H), 5.36 (d, *J* = 3.7 Hz, 1H), 5.19 (dd, *J* = 13.1, 4.9 Hz, 2H), 5.06 (s, 2H), 5.02 (dd, *J* = 12.0, 4.3 Hz, 2H), 4.97 (s, 2H), 4.88 (dt, *J* = 18.0, 9.7 Hz, 3H), 4.73 (d, *J* = 11.2 Hz, 2H), 4.59 (t, *J* = 13.8 Hz, 3H), 4.46 (d, *J* = 11.1 Hz, 1H), 4.36 (d, *J* = 8.1 Hz, 1H), 4.26 – 4.14 (m, 3H), 4.09 – 4.03 (m, 2H), 4.00 (dd, *J* = 10.2, 6.2 Hz, 2H), 3.96 – 3.91 (m, 2H), 3.88 – 3.70 (m, 10H), 3.56 (d, *J* = 7.5 Hz, 1H), 3.40 (dt, *J* = 10.2, 4.5 Hz, 6H), 3.10 (t, *J* = 9.1 Hz, 1H), 3.06 – 2.92 (m, 6H), 2.55 (dd, *J* = 16.4, 10.1 Hz, 3H), 2.34 (d, *J* = 16.6 Hz, 1H), 2.00 (s, 3H), 1.87 (s, 3H), 1.79 (s, 3H), 1.32 (d, *J* = 4.4 Hz, 2H); ^{13}C NMR (125 MHz, $CDCl_3$) δ 209.53, 172.26, 170.81, 168.89, 168.86, 168.61, 168.00, 157.74, 156.57, 138.77, 137.59, 137.47, 137.01, 136.61, 134.73, 133.29, 133.11, 128.78, 128.61, 128.57, 128.47, 128.42, 128.29, 128.19, 128.09, 128.04, 128.02, 127.98, 127.82, 127.81, 127.68, 127.21, 127.00, 126.88, 126.77, 126.62, 126.35, 117.09, 114.80, 99.79, 98.48, 98.17, 97.50, 82.13, 77.44, 76.19, 75.60, 75.08, 74.90, 74.58, 74.36, 74.03, 73.69, 73.13, 72.88, 71.18, 70.86, 70.71, 66.76, 66.55, 66.35, 65.77, 63.03, 60.73, 53.84, 52.55, 38.40, 37.41, 35.31, 29.79, 29.70, 29.04, 27.96, 20.80, 20.78; MS-ESI Anal. Calcd for $C_{89}H_{100}F_3N_6O_{29}$ $[M+H]^+$: 1773.65, found 1773.66.

Compound **S116:** The solution of compound **29** (360 mg, 0.18 mmol) in pyridine:acetic acid was subjected for Lev-group deprotection according to the general procedure to afford compound **S115** (319 mg, 0.17 mmol) in 93% yield. ^{1}H NMR (500 MHz, $CDCl_3$) δ 8.00 (d, *J* = 8.4 Hz, 1H), 7.96 – 7.90 (m, 2H), 7.86 (s, 1H), 7.60 (ddd, *J* = 7.0, 4.9, 1.7 Hz, 4H), 7.58 – 7.51 (m, 2H), 7.41 – 7.26 (m, 20H), 7.26 – 7.10 (m, 8H), 6.91 (t, *J* = 6.2 Hz, 1H), 5.32 (dt, *J* = 12.8, 6.3 Hz, 2H), 5.13 – 5.09 (m, 1H), 5.04 (dt, *J* = 19.3, 7.8 Hz, 4H), 4.97 (s, 1H), 4.92 – 4.83 (m, 3H), 4.72 (d, *J* = 2.2 Hz, 1H), 4.70 – 4.64 (m, 2H), 4.59 – 4.52 (m, 2H), 4.44 (dd, *J* = 17.8, 11.7 Hz, 2H), 4.24 (d, *J* = 8.1 Hz, 1H), 4.22 – 4.12 (m, 3H), 4.10 – 3.85 (m, 7H), 3.83 – 3.74 (m, 3H), 3.73 (d, *J* = 9.2 Hz, 3H), 3.54 (dddd, *J* = 26.4, 19.4, 10.6, 6.4 Hz, 7H), 3.40 (dd, *J* = 10.3, 3.6 Hz, 1H), 3.28 (s, 3H), 3.20 – 2.96 (m, 6H), 1.90 (s, 3H), 1.78 (s, 3H), 1.70 (s, 3H), 1.51 (dd, *J* = 16.3, 10.1 Hz, 1H), 1.41 – 1.35 (m, 1H), 0.99 (s, 9H); ^{13}C NMR (125 MHz, $CDCl_3$) δ 169.79, 169.07, 169.00, 168.71, 167.80, 157.29, 138.31, 137.61, 137.43, 136.89, 136.08, 135.71, 135.69, 134.53, 133.27, 133.19, 133.11, 132.99, 129.62, 129.55, 128.91, 128.68, 128.57, 128.56, 128.44, 128.34, 128.28, 128.26, 128.15, 128.10, 128.07, 127.99, 127.92, 127.73, 127.66, 127.60, 127.55, 127.37, 126.97, 126.87, 126.74, 126.50, 100.46, 100.11, 97.24, 95.03, 82.13, 77.69, 76.28, 74.73, 74.64, 74.43, 74.30, 74.05, 73.88, 72.96, 72.82, 71.12, 70.98, 70.91, 69.52, 68.40, 66.95, 66.21, 63.01, 61.58, 52.85, 52.71, 52.25, 37.32, 34.91, 29.50, 26.70, 20.85, 20.52, 19.17; MS-ESI Anal. Calcd for $C_{100}H_{112}F_3N_6O_{27}Si^+$ $[M+H]^+$: 1913.72, found 1913.56.

Compound **S117:** To the solution of compound **29** (180 mg, 89 µmol) in DCM:water was subjected for Nap-group deprotection according to the general procedure to afford compound **S115** (156 mg, 84 µmol) in 93% yield. ^{1}H NMR (500 MHz, $CDCl_3$) δ 8.37 (d, *J* = 8.0 Hz, 1H),

7.69 – 7.58 (m, 4H), 7.42 – 7.27 (m, 25H), 7.18 (ddd, *J* = 16.8, 13.9, 7.0 Hz, 6H), 6.70 (t, *J* = 6.1 Hz, 1H), 5.43 (d, *J* = 3.6 Hz, 1H), 5.30 (t, *J* = 9.7 Hz, 1H), 5.21 (t, *J* = 6.1 Hz, 1H), 5.16 – 5.11 (m, 1H), 5.07 (dd, *J* = 19.3, 9.3 Hz, 4H), 4.98 (s, 2H), 4.88 (d, *J* = 10.9 Hz, 1H), 4.80 (d, *J* = 11.0 Hz, 1H), 4.78 – 4.67 (m, 3H), 4.67 – 4.56 (m, 3H), 4.22 (d, *J* = 15.1 Hz, 1H), 4.18 – 4.07 (m, 4H), 4.01 – 3.91 (m, 3H), 3.90 – 3.69 (m, 11H), 3.59 (ddd, *J* = 14.5, 11.6, 2.5 Hz, 2H), 3.49 (d, *J* = 10.0 Hz, 1H), 3.38 (dd, *J* = 10.4, 3.6 Hz, 1H), 3.22 (s, 3H), 3.08 – 2.96 (m, 5H), 2.64 – 2.54 (m, 2H), 2.42 – 2.34 (m, 1H), 2.11 – 2.06 (m, 1H), 2.05 (d, *J* = 7.0 Hz, 3H), 1.92 (s, 3H), 1.86 (s, 3H), 1.01 (s, 9H); ^{13}C NMR (125 MHz, $CDCl_3$) δ 209.62, 172.29, 169.38, 168.98, 168.86, 167.90, 157.73, 157.44, 156.61, 138.66, 137.58, 137.39, 136.96, 136.60, 135.75, 135.69, 133.14, 133.04, 129.63, 129.55, 128.60, 128.55, 128.51, 128.47, 128.30, 128.24, 128.20, 128.04, 128.00, 127.87, 127.61, 127.56, 127.40, 127.21, 126.78, 117.06, 114.77, 100.70, 98.33, 97.78, 97.52, 82.30, 77.54, 77.23, 77.14, 76.97, 74.82, 74.69, 74.60, 74.39, 74.21, 73.89, 73.36, 72.98, 72.79, 72.54, 71.01, 69.45, 66.83, 66.56, 65.90, 62.93, 61.65, 60.38, 53.74, 52.58, 52.26, 38.51, 37.42, 35.31, 29.81, 29.15, 27.98, 26.71, 20.91, 20.81, 19.19; MS-ESI Anal. Calcd for $C_{94}H_{11}0F_3N_6O_{29}Si$ $[M+H]^+$: 1871.70, found 1871.67.

Compound **S118:** The solution of compound **S116** (180 mg, 93 µmol) in DCM:water was subjected for Nap-group deprotection according to the general procedure to afford compound **S118** (146 mg, 83 µmol) in 88% yield. ^1H NMR (500 MHz, $CDCl_3$) δ 7.67 – 7.55 (m, 4H), 7.43 – 7.27 (m, 26H), 7.26 – 7.15 (m, 5H), 6.92 (t, *J* = 6.0 Hz, 1H), 5.43 (d, *J* = 3.5 Hz, 1H), 5.30 (t, *J* = 9.7 Hz, 1H), 5.19 – 5.14 (m, 1H), 5.10 (t, *J* = 6.3 Hz, 1H), 5.05 (d, *J* = 12.2 Hz, 1H), 5.02 – 4.98 (m, 2H), 4.95 (d, *J* = 12.0 Hz, 1H), 4.92 (d, *J* = 3.2 Hz, 1H), 4.86 (d, *J* = 10.9 Hz, 1H), 4.80 (d, *J* = 11.0 Hz, 1H), 4.72 (d, *J* = 3.0 Hz, 2H), 4.68 (dd, *J* = 9.7, 4.3 Hz, 2H), 4.61 (d, *J* = 11.0 Hz, 1H), 4.55 (d, *J* = 11.3 Hz, 1H), 4.49 (d, *J* = 12.0 Hz, 1H), 4.18 (dd, *J* = 22.2, 12.5 Hz, 2H), 4.13 – 4.06 (m, 3H), 3.99 (d, *J* = 9.3 Hz, 1H), 3.91 (d, *J* = 9.2 Hz, 2H), 3.89 – 3.82 (m, 3H), 3.82 – 3.70 (m, 6H), 3.62 (d, *J* = 10.0 Hz, 1H), 3.56 (t, *J* = 9.8 Hz, 2H), 3.49 (dd, *J* = 10.0, 7.7 Hz, 2H), 3.37 (dd, *J* = 10.4, 3.6 Hz, 1H), 3.28 (s, 3H), 3.19 (s, 2H), 3.14 – 2.99 (m, 3H), 2.03 (s, 3H), 1.93 (s, 1H), 1.86 (s, 3H), 1.00 (s, 9H); ^{13}C NMR (125 MHz, $CDCl_3$) δ 169.70, 169.15, 169.05, 168.99, 167.90, 157.40, 138.25, 137.49, 137.42, 136.82, 135.99, 135.73, 135.69, 133.14, 132.98, 129.63, 129.57, 128.69, 128.58, 128.56, 128.50, 128.36, 128.28, 128.23, 127.98, 127.88, 127.80, 127.60, 127.56, 127.43, 127.36, 120.29, 116.86, 114.52, 100.88, 100.57, 97.37, 95.38, 82.39, 77.55, 77.34, 76.48, 74.88, 74.65, 74.55, 74.30, 73.94, 73.05, 72.81, 71.97, 71.67, 71.02, 69.42, 68.27, 67.00, 66.27, 62.89, 61.62, 52.90, 52.27, 37.30, 34.88, 29.45, 26.71, 20.81, 20.73, 19.18; MS-ESI Anal. Calcd for $C_{89}H_{104}F_3N_6O_{27}Si$ $[M+H]^+$: 1773.66, found 1773.99.

Compound **S119:** The solution of compound **S116** (70 mg, 37 µmol) in pyridine was subjected for silyl-group deprotection according to the general procedure to afford compound **S118** (61 mg, 36 µmol) in 88% yield. ^1H NMR (500 MHz, $CDCl_3$) δ 8.00 (d, *J* = 8.4 Hz, 1H), 7.93 (dd, *J* = 9.1, 5.0 Hz, 2H), 7.87 (s, 1H), 7.61 (d, *J* = 8.3 Hz, 1H), 7.58 – 7.52 (m, 2H), 7.43 – 7.27 (m, 20H), 7.25 – 7.15 (m, 5H), 7.11 (d, *J* = 7.0 Hz, 2H), 6.92 (t, *J* = 6.1 Hz, 1H), 5.35 (d, *J* = 3.7 Hz, 1H), 5.10 (t, *J* = 6.4 Hz, 1H), 5.08 – 4.96 (m, 5H), 4.94 – 4.85 (m, 4H), 4.73 (dd, *J* = 10.5, 6.7 Hz, 2H), 4.67 (d,

J = 11.2 Hz, 1H), 4.56 (dd, J = 11.7, 6.2 Hz, 2H), 4.44 (dd, J = 19.3, 11.6 Hz, 2H), 4.27 (d, J = 8.1 Hz, 1H), 4.23 – 4.16 (m, 3H), 4.12 – 3.88 (m, 7H), 3.79 (dd, J = 19.7, 9.9 Hz, 3H), 3.71 (s, 3H), 3.63 (d, J = 9.4 Hz, 2H), 3.60 – 3.40 (m, 8H), 3.37 (dd, J = 10.3, 3.7 Hz, 1H), 3.21 – 2.95 (m, 6H), 2.49 (s, 1H), 2.00 (s, 3H), 1.78 (s, 3H); ^{13}C NMR (125 MHz, $CDCl_3$) δ 170.75, 169.80, 169.08, 168.65, 168.02, 157.32, 138.31, 137.51, 137.49, 136.89, 136.06, 134.59, 133.28, 133.16, 128.86, 128.68, 128.57, 128.42, 128.34, 128.30, 128.27, 128.09, 128.07, 127.99, 127.91, 127.83, 127.80, 127.73, 127.68, 127.38, 126.95, 126.85, 126.72, 126.46, 116.81, 100.48, 100.08, 97.45, 95.14, 82.08, 77.38, 77.22, 76.93, 76.48, 75.33, 75.03, 74.69, 74.41, 74.36, 74.05, 73.03, 72.81, 71.21, 71.00, 70.81, 70.72, 66.96, 66.19, 62.99, 60.74, 52.72, 52.57, 37.31, 34.90, 29.49, 20.80, 20.53; HRMS (ESI) Anal. Calcd for $C_{84}H_{94}F_3N_6O_{27}$ [M+H]$^+$: 1675.6114, found 1675.6144.

Compound **S120:** The solution of compound **S117** (74 mg, 39 µmol) in pyridine:acetic acid was subjected for Lev-group deprotection, followed by silyl-group deprotection according to the general procedures to afford compound **S120** (46 mg, 30 µmol) in 76% yield over 2 steps. ^{1}H NMR (500 MHz, $CDCl_3$) δ 7.46 (d, J = 8.6 Hz, 2H), 7.40 – 7.28 (m, 20H), 7.22 (t, J = 6.0 Hz, 3H), 6.91 (t, J = 6.1 Hz, 1H), 5.43 (d, J = 3.7 Hz, 1H), 5.17 – 5.12 (m, 2H), 5.08 – 4.94 (m, 5H), 4.95 – 4.91 (m, 1H), 4.89 (d, J = 9.1 Hz, 1H), 4.85 (d, J = 6.1 Hz, 1H), 4.82 (s, 1H), 4.71 (dt, J = 17.2, 6.9 Hz, 5H), 4.56 (d, J = 11.3 Hz, 1H), 4.49 (d, J = 11.9 Hz, 1H), 4.24 – 4.06 (m, 6H), 4.02 (d, J = 9.3 Hz, 1H), 3.97 – 3.87 (m, , 4H), 3.87 – 3.72 (m, 9H), 3.58 (dd, J = 17.4, 8.2 Hz, 2H), 3.54 – 3.41 (m, 6H), 3.35 (dd, J = 10.3, 3.7 Hz, 1H), 3.19 (dd, J = 13.5, 6.3 Hz, 2H), 3.05 (ddd, J = 30.8, 13.0, 6.4 Hz, 3H), 2.03 (s, 3H), 1.97 (s, 3H); ^{13}C NMR (125 MHz, $CDCl_3$) δ 170.78, 169.73, 169.10, 169.08, 168.13, 157.71, 157.37, 138.26, 137.47, 136.85, 128.70, 128.58, 128.53, 128.37, 128.31, 128.02, 127.89, 127.80, 127.67, 127.39, 127.35, 116.86, 114.57, 100.82, 100.51, 97.66, 95.36, 82.27, 77.23, 76.59, 75.69, 75.00, 74.81, 74.74, 74.62, 73.14, 72.94, 72.79, 71.97, 71.57, 70.86, 70.73, 68.22, 66.97, 66.27, 62.87, 60.77, 60.14, 52.85, 52.62, 37.31, 34.92, 29.46, 20.77, 20.73; HRMS (ESI) Anal. Calcd for $C_{73}H_{86}F_3N_6O_{27}$ [M+H]$^+$: 1535.5488, found 1535.5501.

Compound **S121:** The solution of compound **S117** (82 mg, 44 µmol) in pyridine was subjected to silyl-group deprotection according to the general procedure to afford compound **S121** (63 mg, 39 µmol) in 88% yield. ^{1}H NMR (500 MHz, $CDCl_3$) δ 8.40 (d, J = 7.9 Hz, 1H), 7.40 – 7.24 (m, 22H), 7.22 – 7.14 (m, 3H), 6.69 (t, J = 6.2 Hz, 1H), 5.45 (d, J = 3.8 Hz, 1H), 5.19 (t, J = 6.0 Hz, 1H), 5.15 – 5.10 (m, 1H), 5.09 (d, J = 3.7 Hz, 1H), 5.05 (d, J = 11.5 Hz, 3H), 4.98 (s, 2H), 4.91 – 4.81 (m, 3H), 4.76 (d, J = 11.1 Hz, 1H), 4.74 – 4.61 (m, 4H), 4.59 (d, J = 11.1 Hz, 1H), 4.25 – 4.07 (m, 6H), 4.01 – 3.93 (m, 3H), 3.92 – 3.78 (m, 9H), 3.76 – 3.70 (m, 1H), 3.60 – 3.54 (m, 1H), 3.48 – 3.41 (m, 5H), 3.35 (dd, J = 10.3, 3.8 Hz, 1H), 3.07 – 2.95 (m, 5H), 2.64 – 2.54 (m, 2H), 2.50 (dd, J = 8.8, 4.9 Hz, 1H), 2.42 – 2.35 (m, 1H), 2.10 – 2.06 (m, 1H), 2.05 (s, 3H), 1.97 (s, 3H), 1.91 (s, 3H); ^{13}C NMR (125 MHz, $CDCl_3$) δ 210.39, 209.73, 172.29, 170.82, 169.27, 168.85, 168.07, 157.48, 156.60, 138.65, 137.51, 137.46, 136.95, 136.59, 128.60, 128.53, 128.52, 128.47, 128.30, 128.23, 128.04, 128.01, 127.86, 127.78, 127.31, 127.26, 126.69, 114.77, 100.72, 98.33, 97.94, 97.69, 82.28, 77.22, 77.13, 76.93, 75.83, 75.03, 74.86, 74.64, 73.89, 73.42, 73.24, 72.79, 72.56, 70.80, 66.76, 66.56, 65.86, 62.89, 60.74, 60.38, 53.77, 52.58, 38.53, 37.41, 35.30, 29.81, 29.14,

28.00, 20.92, 20.76; HRMS (ESI) Anal. Calcd for $C_{78}H_{92}F_3N_6O_{29}$ $[M+H]^+$: 1633.5855, found 1633.5880.

Procedures for microarray studies

Immobilization of disaccharides on the array slides.

All 64 tetrasaccharides were dissolved in sodium phosphate buffer (pH 8.5, 50 mM) in concentrations of 50 µM, 25 µM and 12.5 µM. The solution was spatially arrayed onto NHS-activated slides (Nexterion® Slide H from SCHOTT, Jena, Germany) under ~50% relative humidity at 20°C. The robotic arrayer SX (from Scienion, Berlin, Germany) delivered 440 pL of the solution containing disaccharides to the array slide. The array spots had an average diameter of about 80 µm with a distance of 280 µm between the centers of adjacent spots. The slides were incubated overnight in a saturated $(NH_4)_2SO_4$ chamber (81% relative humidity). The slides were then washed with water to remove the unreacted oligosaccharides from the surface. The remaining *N*-hydroxysuccinimidyl groups were blocked by placing slides in a solution that contained 50 mM ethanolamine in PBST (137 mM NaCl, 13.2 mM Na_2HPO_4, 1.56 mM NaH_2PO_4, 2.68 mM KCl, 0.01% Tween 20) at 50°C for at least 1.5 h. Slides were rinsed several times with deionized water, and the residual liquid was dried by centrifugation.

Preparation of fluorescently labeled FGF-2

Fluorescently labeled FGF-2 was prepared by direct labeling with Alexa Fluor 488 NHS Ester (Thermo Scientific, Waltham, MA, USA). FGF-2 (100 µg) was mixed with three equivalents of heparin and 100 mM $NaHCO_3$. The mixture was allowed to equilibrate for 15 min at room temperature followed by the addition of 3–10 equivalents of Alexa Fluor 488 NHS Ester. The reaction was left to proceed at room temperature for 1 h. The reaction mixture was purified by heparin column and buffer exchanged [phosphate-buffered saline (PBS)] using a centrifugal filter to get Alexa Fluor 488 labeled FGF-2.

Hybridization of array slides with Alexa Fluor 488 labeled FGF-2.

The hybridization solution contained 10 µg mL^{-1} of Alexa Fluor 488 labeled FGF-2 PBST (137 mM NaCl, 2.7 mM KCl, 4.3 mM Na_2HPO_4, 1.4 mM KH_2PO_4, 0.05% Tween 20), 20 mM Tris (pH 7.5) and 10% bovine serum albumin (BSA). The solution was placed between array slide and cover slip and incubated for 1 h at room temperature in a saturated $(NH_4)_2SO_4$ chamber (81% relative humidity). The slide was then washed with 45 mL of PBST solution containing BSA (1%) and Tris (20 mM) for 30 min in a clean 50 mL conical tube. Slides were rinsed several times with deionized water, and the residual liquid was dried by centrifugation before analyzing the slide with the array scanner.

Preparation of fluorescently labeled human Platelet Factor 4 (PF4).

Fluorescently labeled PF4 was prepared by direct labeling with Alexa Fluor 488 NHS Ester (Thermo Scientific, Waltham, MA, USA). Human PF4 (100 µg) was mixed with three equivalents

of heparin and 100 mM $NaHCO_3$. The mixture was allowed to equilibrate for 15 min at room temperature followed by the addition of 3–10 equivalents of Alexa Fluor 488 NHS Ester. The reaction was left to proceed at room temperature for 1 h. The reaction mixture was purified by heparin column and buffer exchanged [phosphate-buffered saline (PBS)] using a centrifugal filter to get Alexa Fluor 488 labeled human PF4.

Hybridization of array slides with Alexa Fluor 488 labeled PF4.

The hybridization solution contained 10 μg mL^{-1} of Alexa Fluor 488 labeled PF4, PBST (137 mM NaCl, 2.7 mM KCl, 4.3 mM Na_2HPO_4, 1.4 mM KH_2PO_4, 0.05% Tween 20), 20 mM Tris (pH 7.5) and 10% bovine serum albumin (BSA). The solution (100 µL) was placed between the array slide and the cover slip, and incubated for 1 h at room temperature in a saturated $(NH_4)_2SO_4$ aqueous solution chamber (81% relative humidity). The slide was then washed with 45 mL of PBST solution containing BSA (1%) and Tris (20 mM) for 30 min in a clean 50 mL conical tube. Slides were rinsed several times with deionized water, and the residual liquid was dried by centrifugation before analyzing the slide with the array scanner.

Array data analysis

The array slides were scanned by a GenePix 4400 scanner (Molecular Dynamics). Scanning wavelength was 488 nm. Resolution was set at 5 μm. The array images were analyzed by GenePix Pro 7.2.29.002 software. Spots were automatically found, and spot deviations were manually fit to correct. Mean median fluorescence intensities of arrays were obtained by Array Quality Control of software. Some thresholds were listed as follows: median signal-to-background, >10; mean of median background, < 500; median signal-to-noise, > 10; The intensity data are the mean value ± S.D. of 27 individual spots.

References

1. Dulaney, S. B.; Xu, Y.; Wang, P.; Tiruchinapally, G.; Wang, Z.; Kathawa, J.; El-Dakdouki, M. H.; Yang, B.; Huang, X. Divergent synthesis of heparan sulfate oligosaccharides. *J. Org. Chem.* **2015**, *80*, 12265−12279.
2. Huang, L.; Huang, X., Highly Efficienct Syntheses of Hyaluronic Acid Oligosaccharides. *Chem. Eur. J.* **2007,** *13*, 529-540.
3. Ramadan, S.; Su, G.; Baryal, K.; Hsieh-Wilson, L. C.; Liu, J.; Huang, X., Automated solid phase assisted synthesis of a heparan sulfate disaccharide library. *Org. Chem. Front.* **2022,** *9*, 2910-2920.

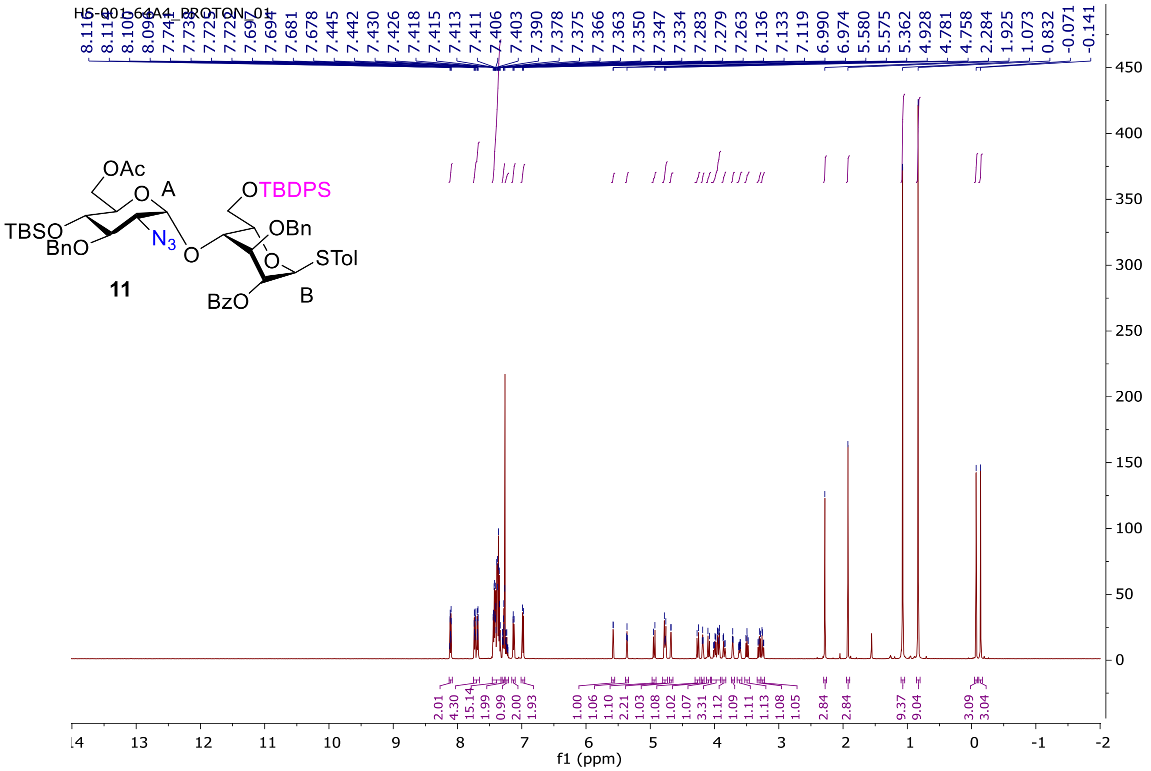

^{1}H NMR spectrum of compound **11**

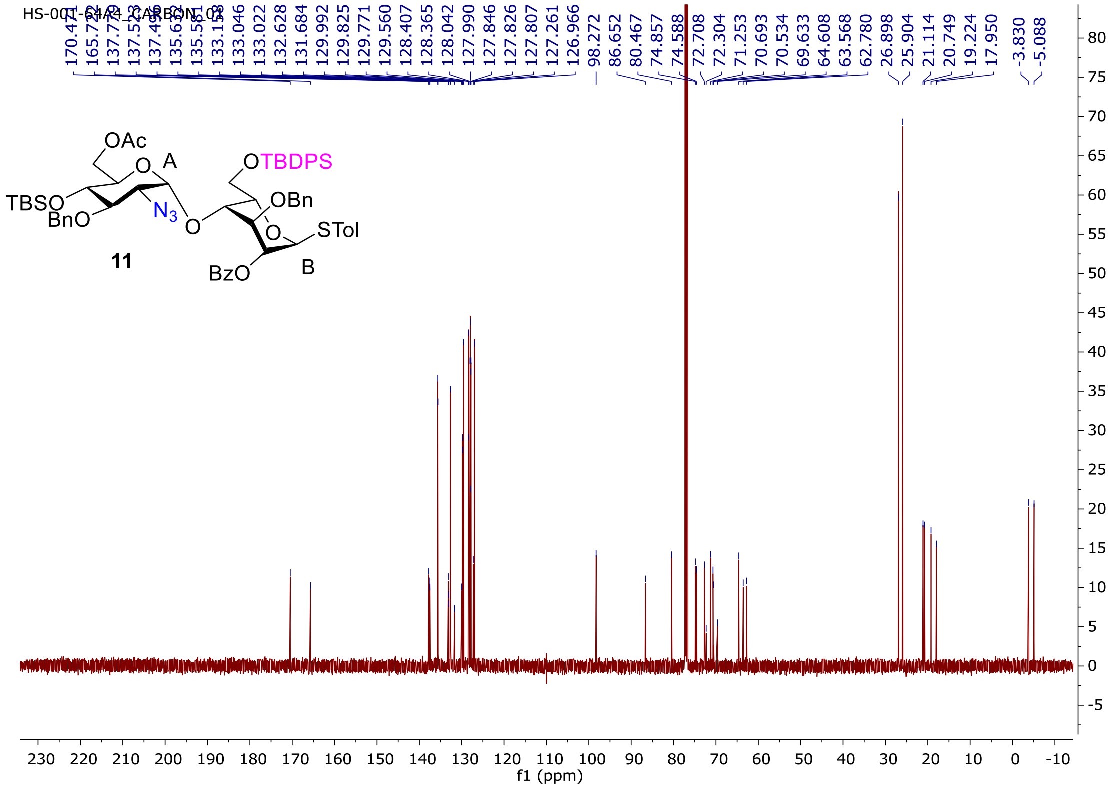

^{13}C NMR spectrum of compound **11**

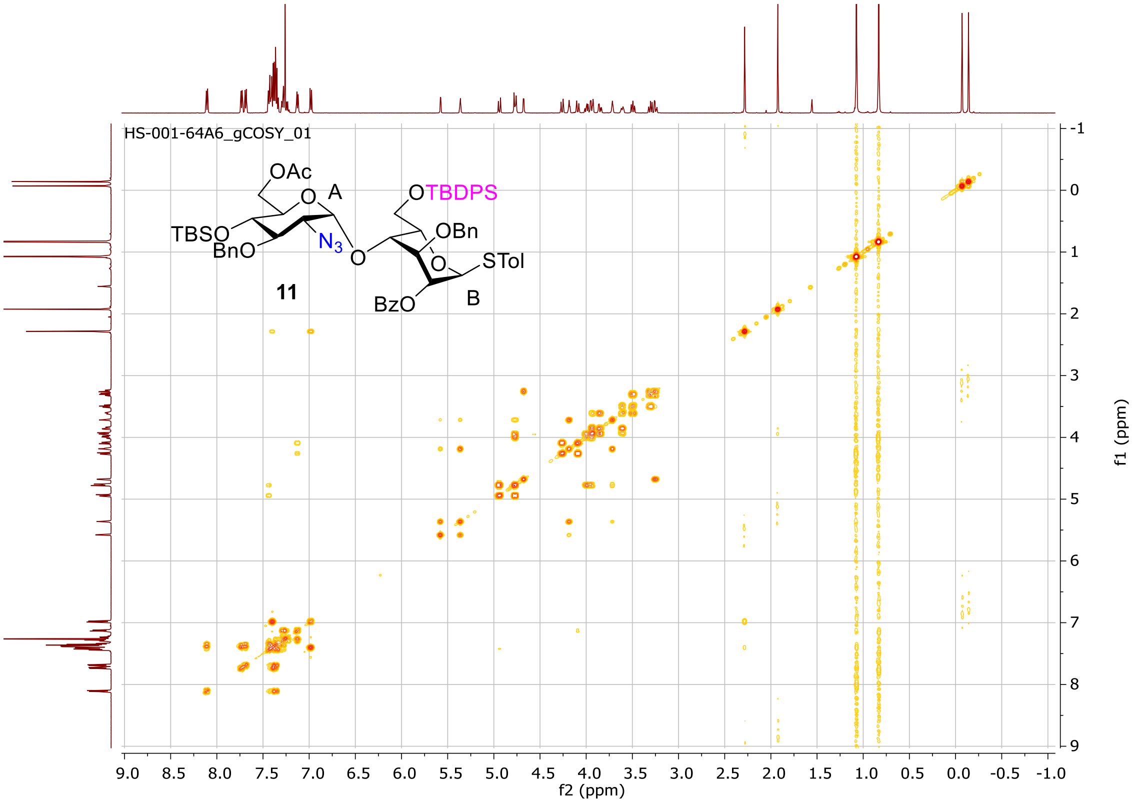

gCOSY NMR spectrum of compound **11**

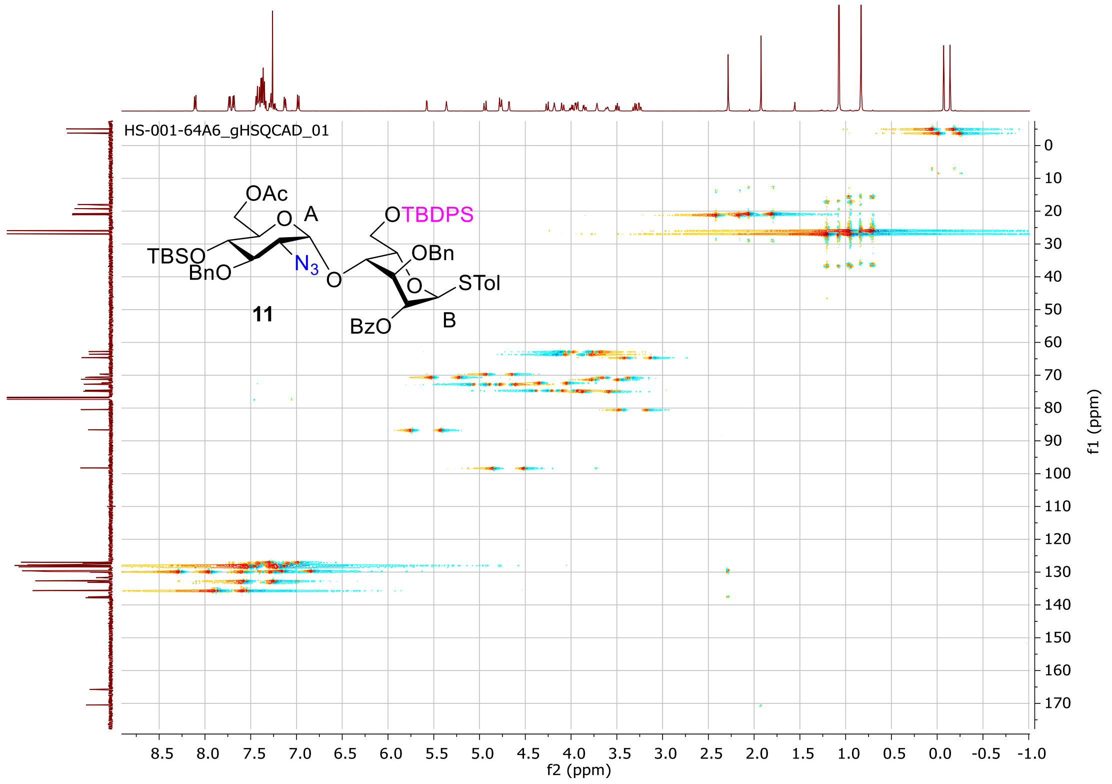

^{1}H-Coupled gHSQC NMR spectrum of compound **11**

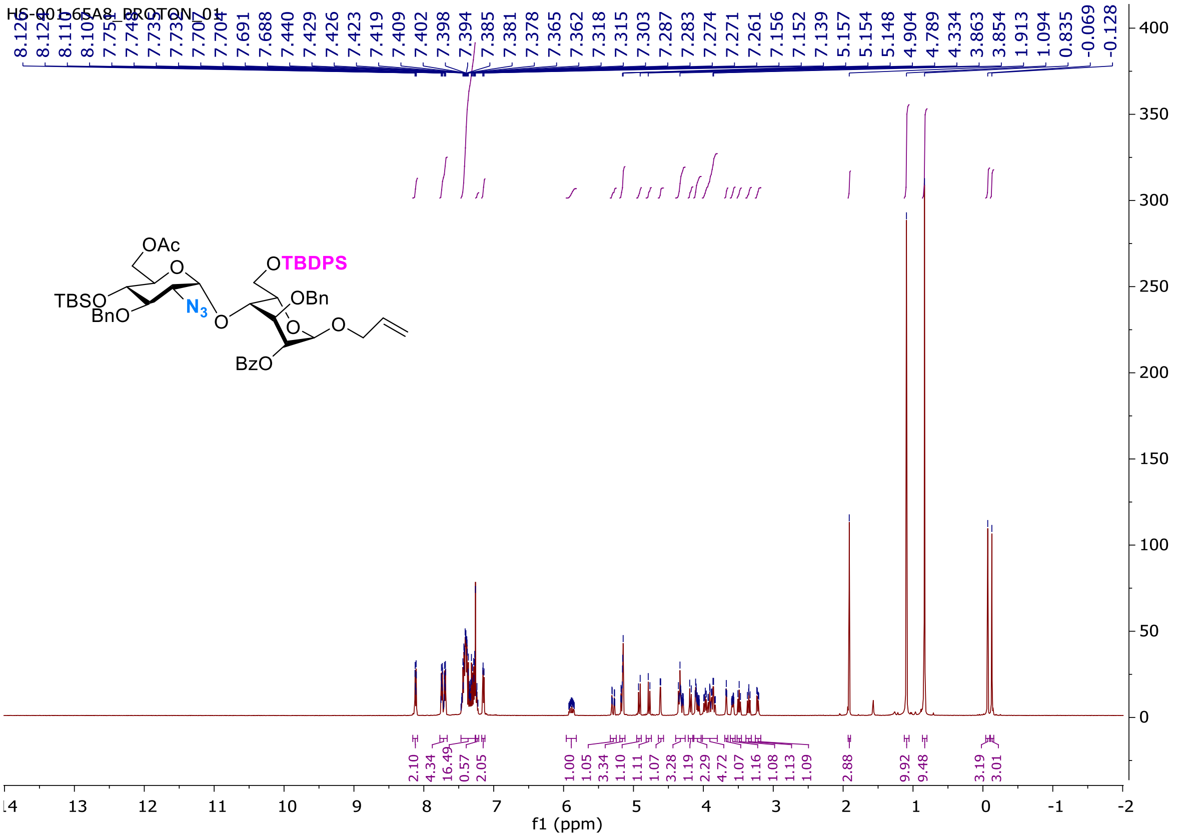

^{1}H NMR spectrum of compound **12**

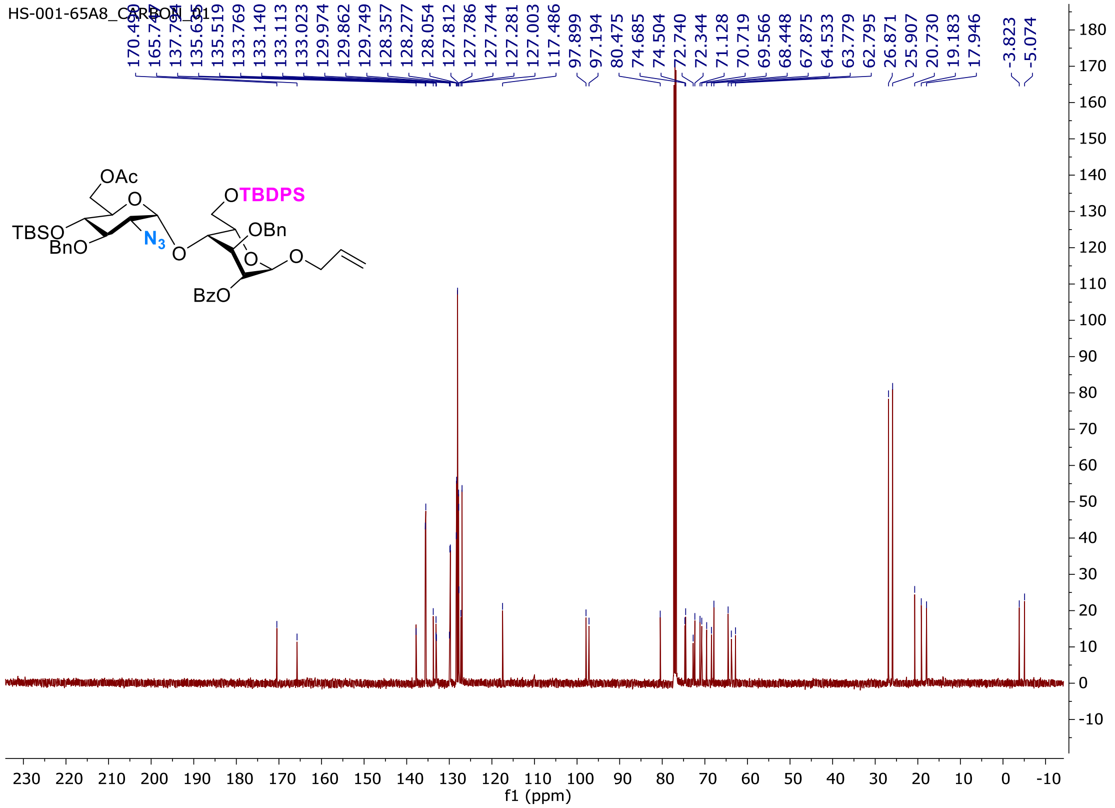

^{13}C NMR spectrum of compound **12**

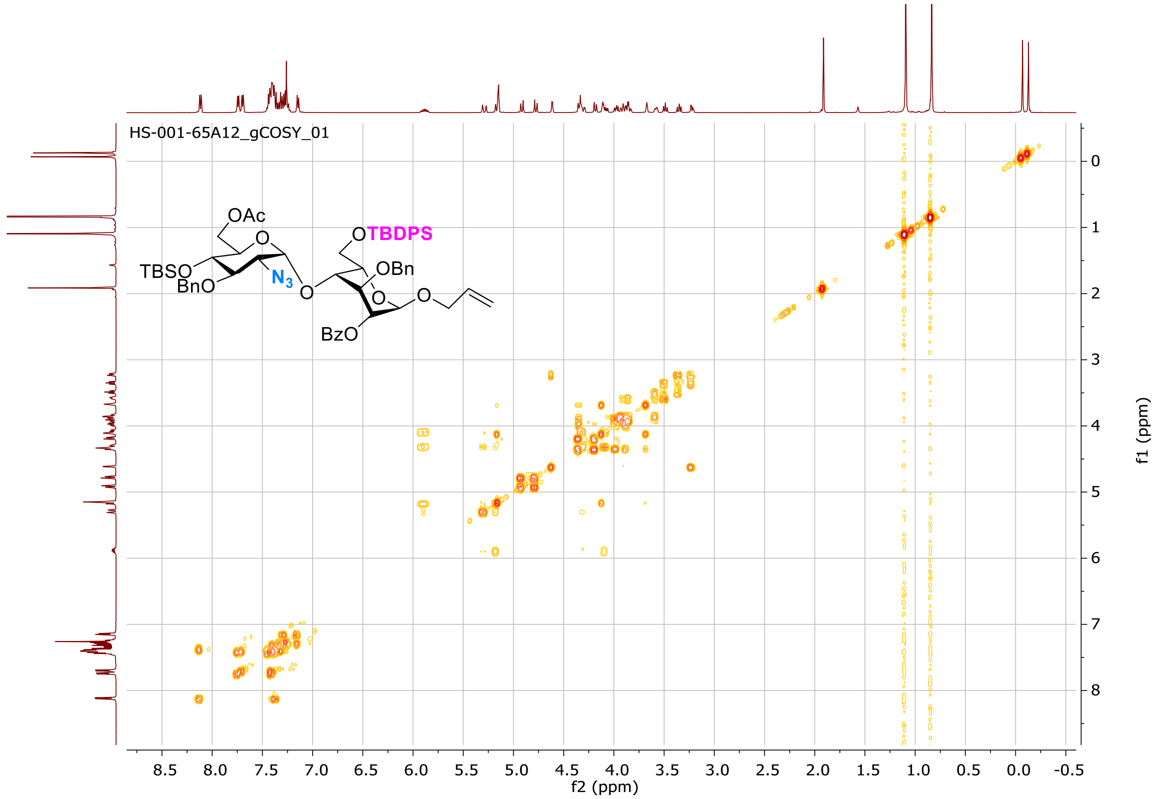

gCOSY NMR spectrum of compound **12**

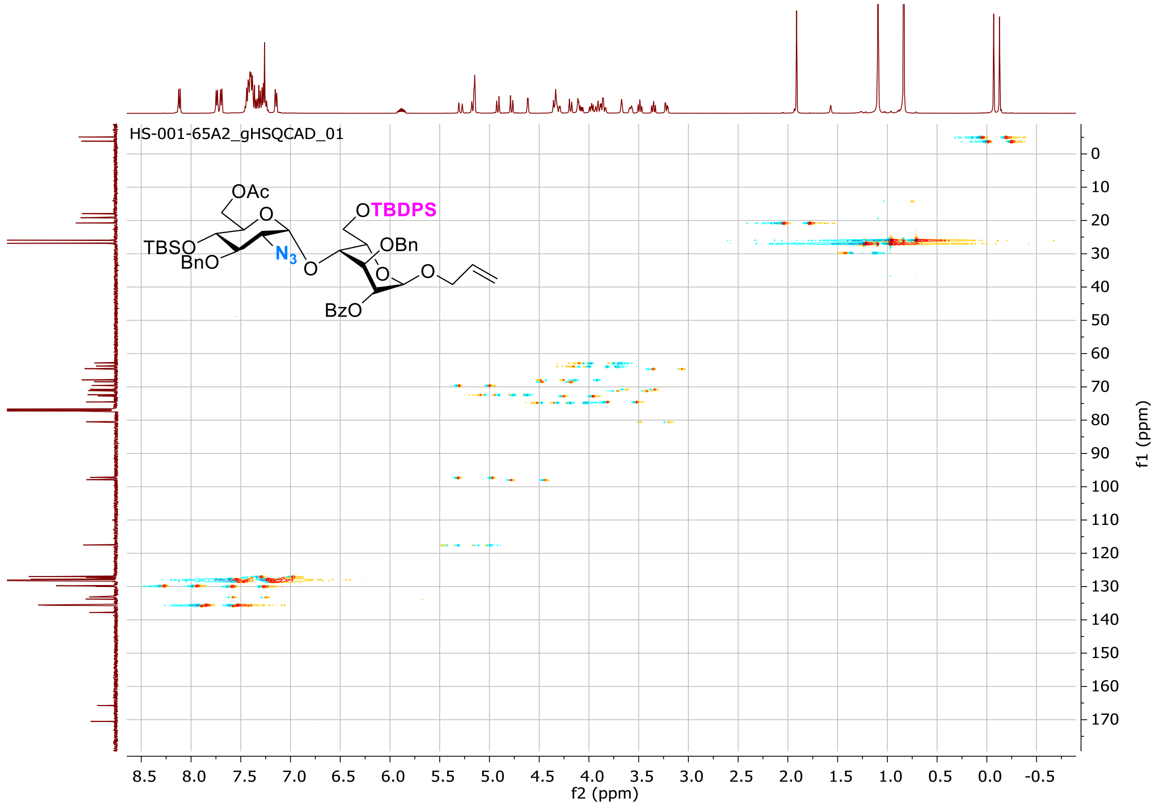

^{1}H-Coupled gHSQC NMR spectrum of compound **12**

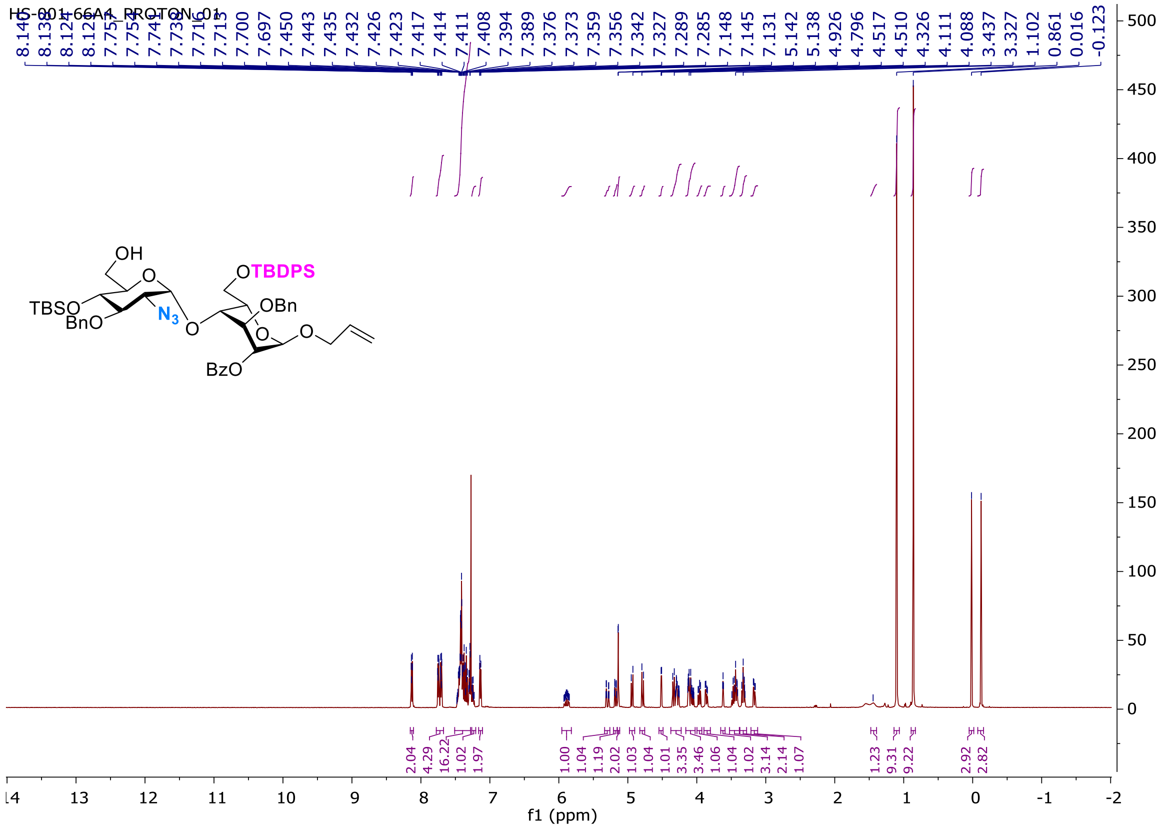

^{1}H NMR spectrum of compound **S1**

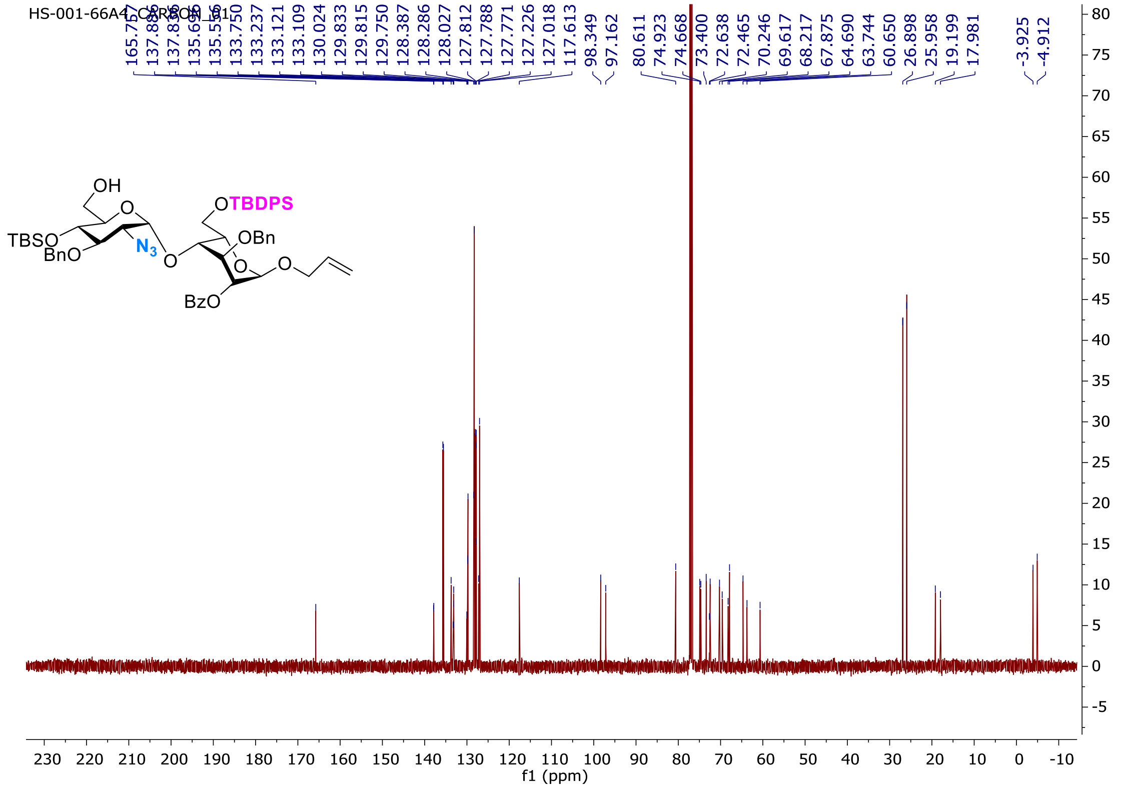

^{13}C NMR spectrum of compound **S1**

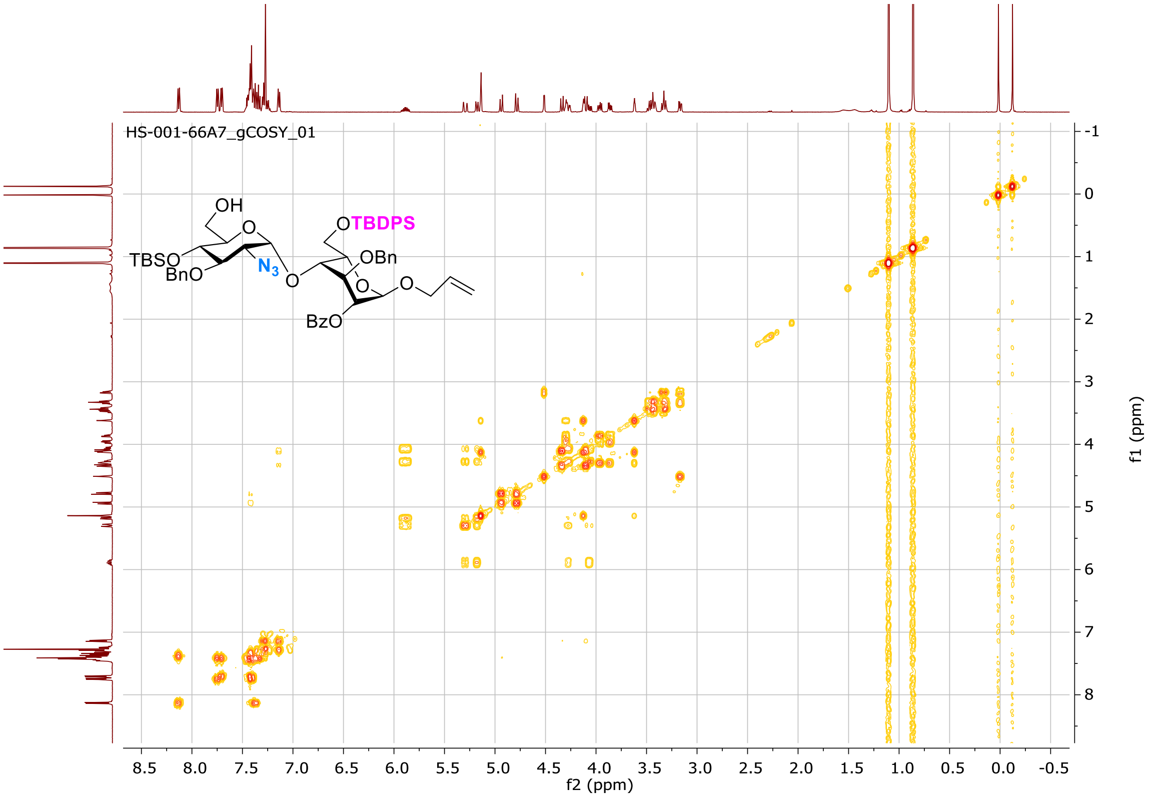

gCOSY NMR spectrum of compound **S1**

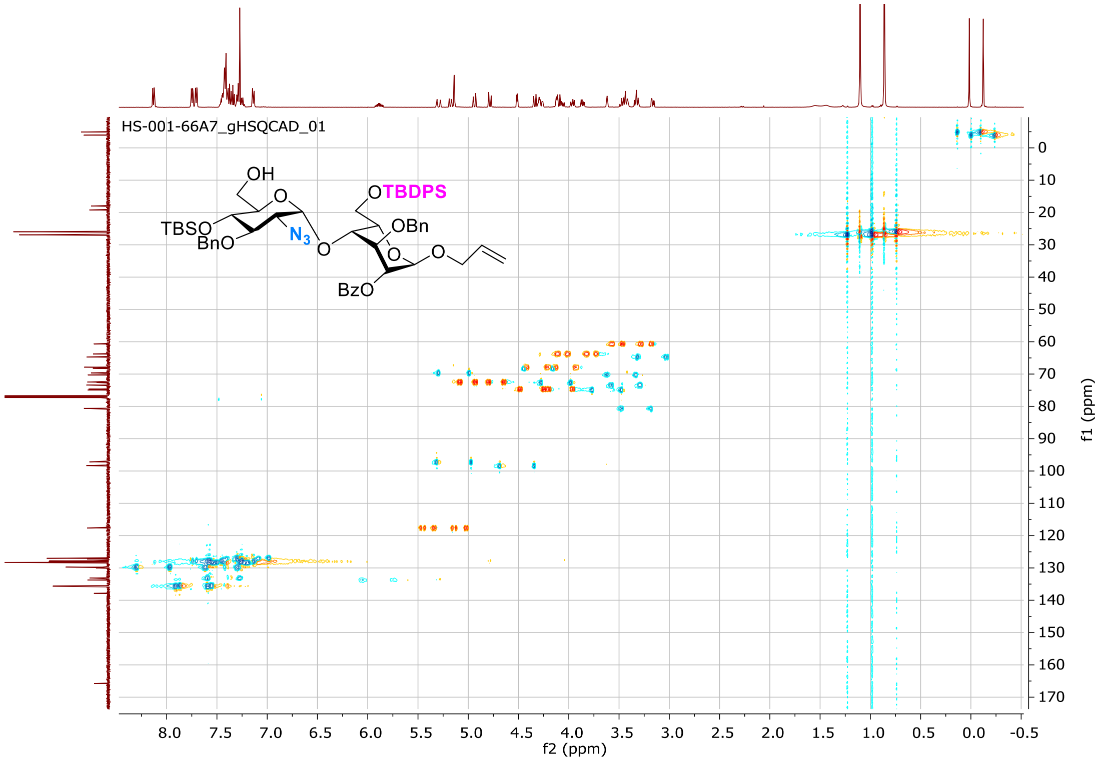

^{1}H-Coupled gHSQC NMR spectrum of compound **S1**

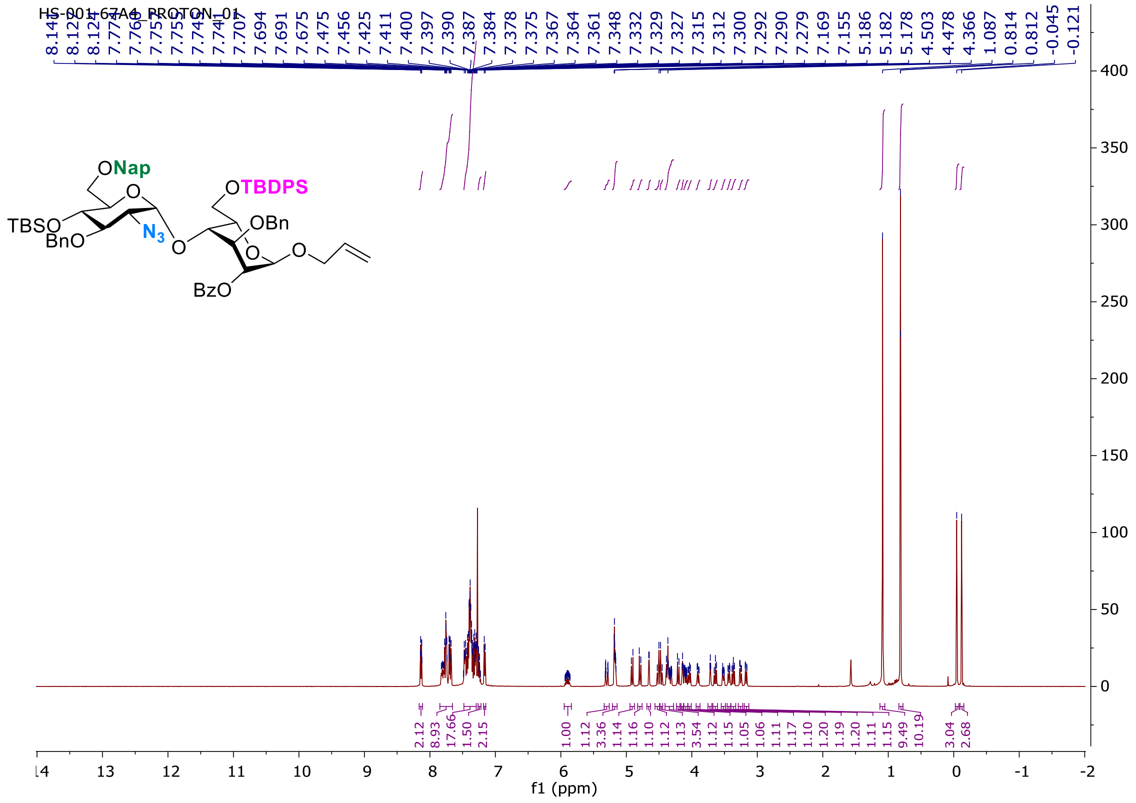

^{1}H NMR spectrum of compound **S2**

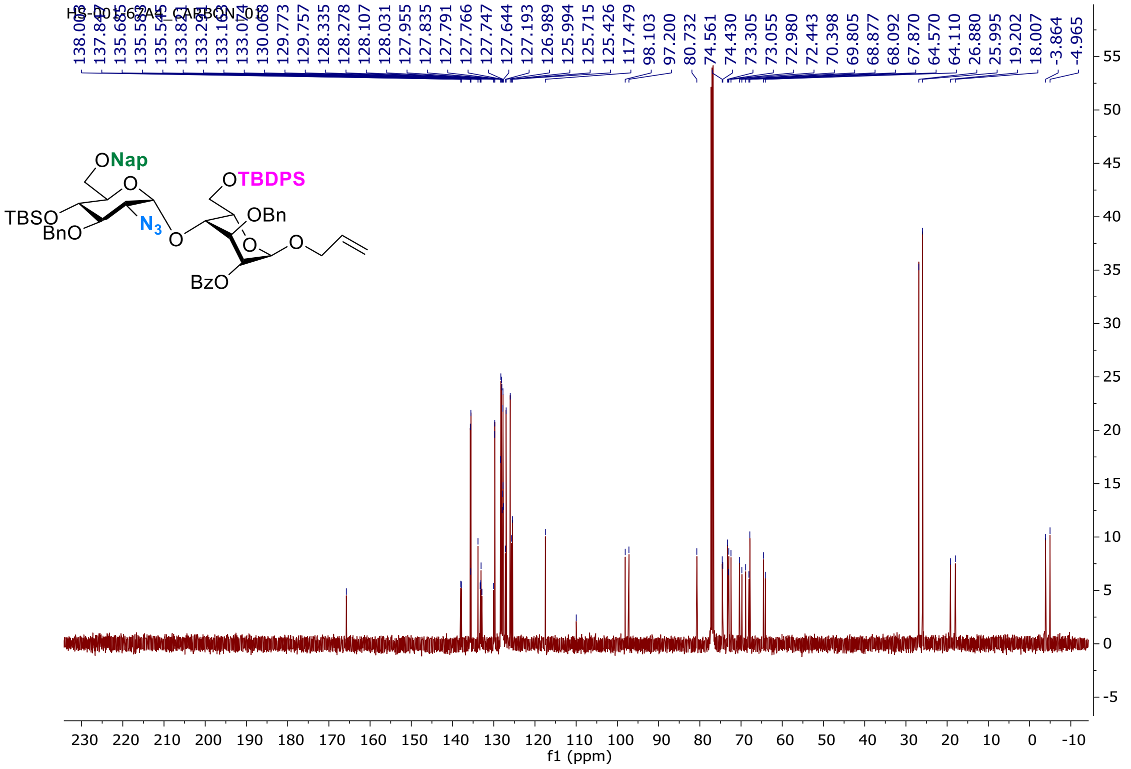

^{13}C NMR spectrum of compound **S2**

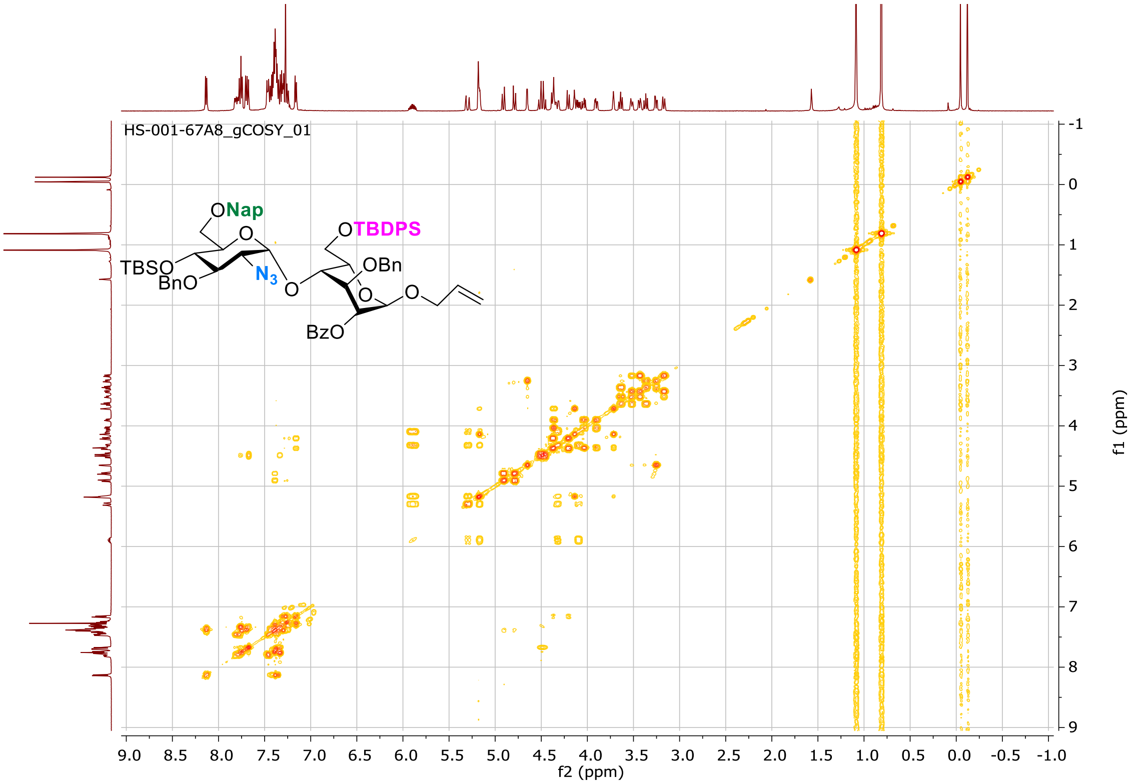

gCOSY NMR spectrum of compound **S2**

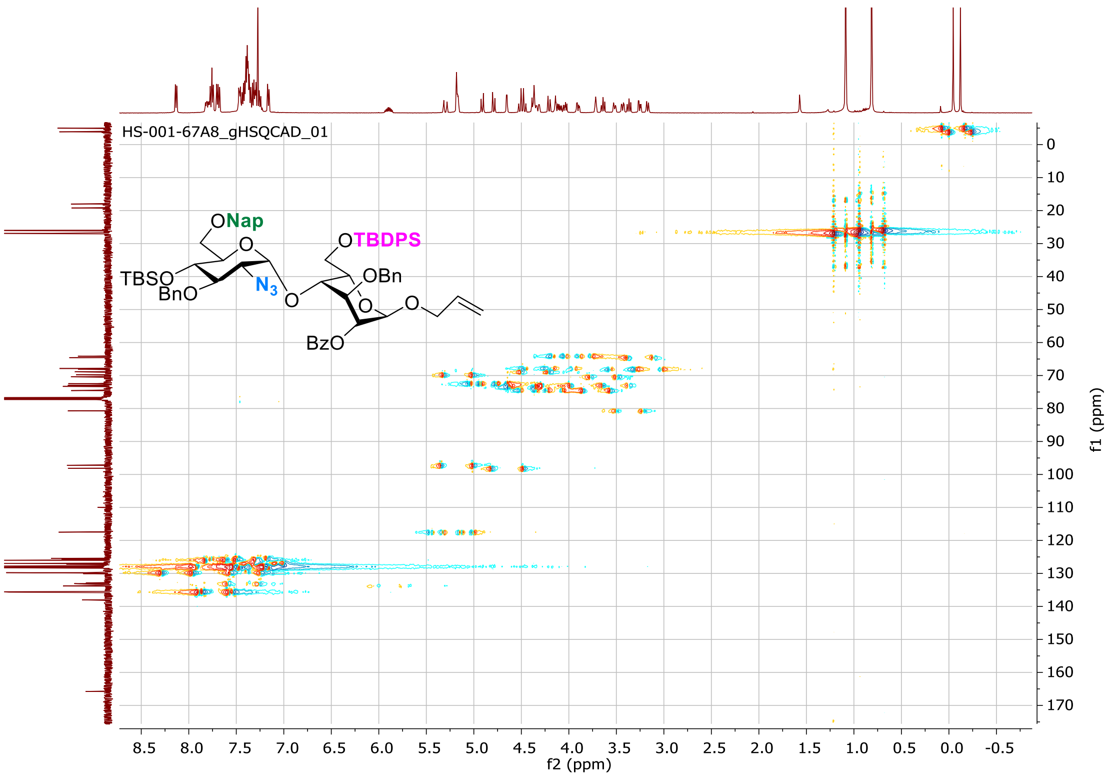

^{1}H-Coupled gHSQC NMR spectrum of compound **S2**

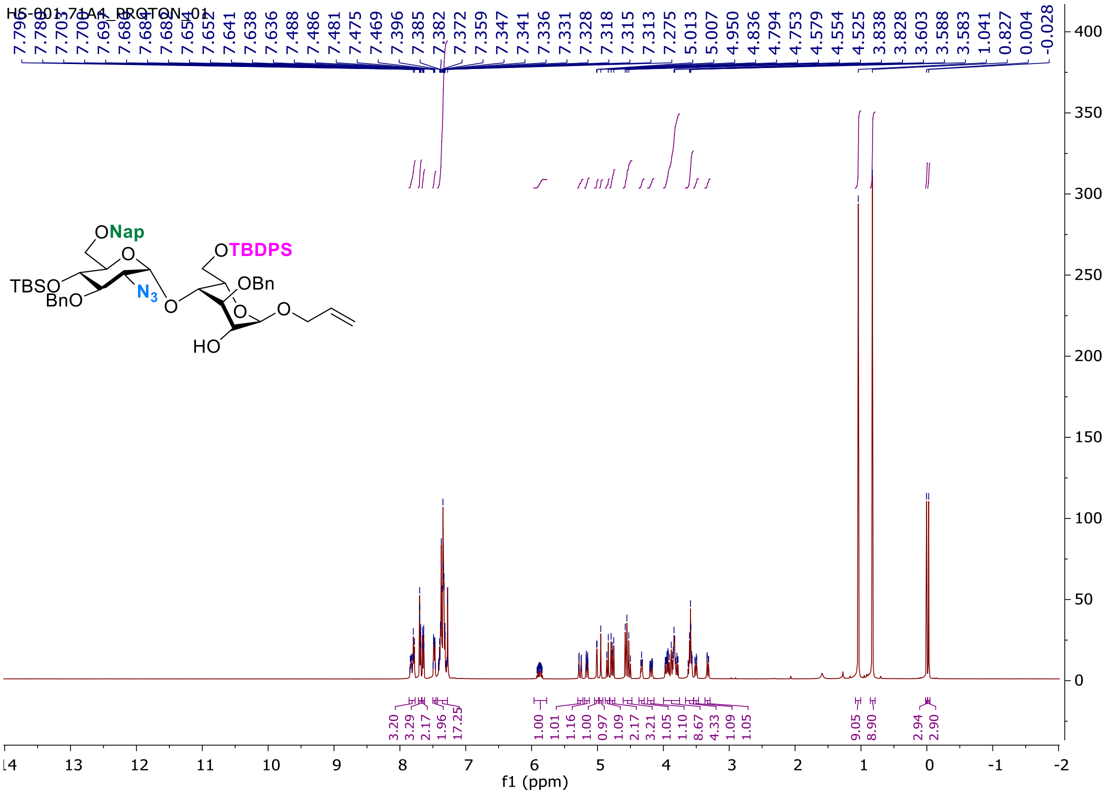

^{1}H NMR spectrum of compound **S3**

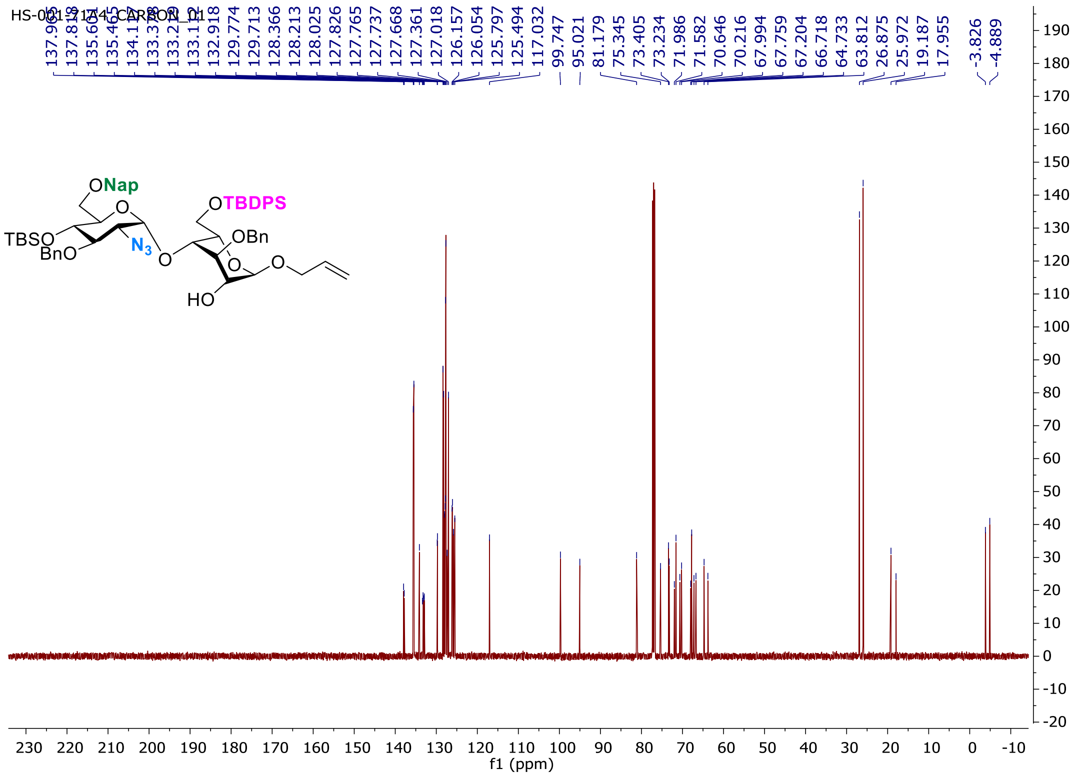

^{13}C NMR spectrum of compound **S3**

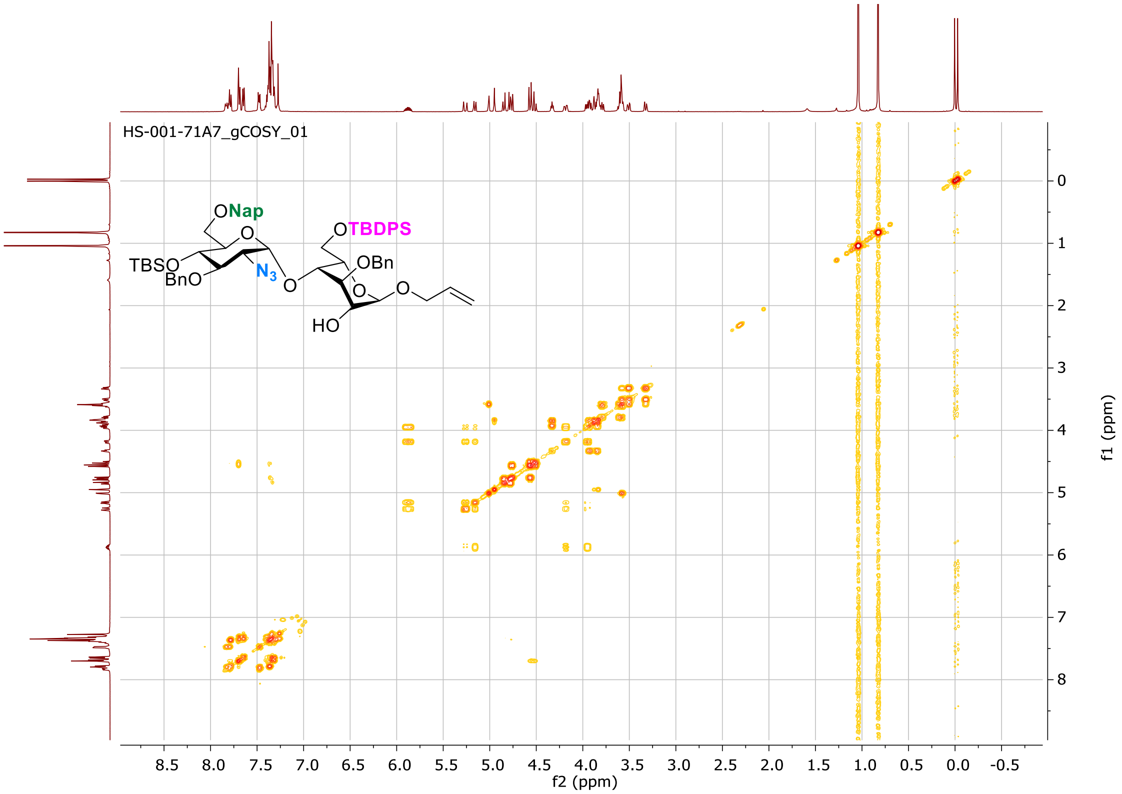

gCOSY NMR spectrum of compound **S3**

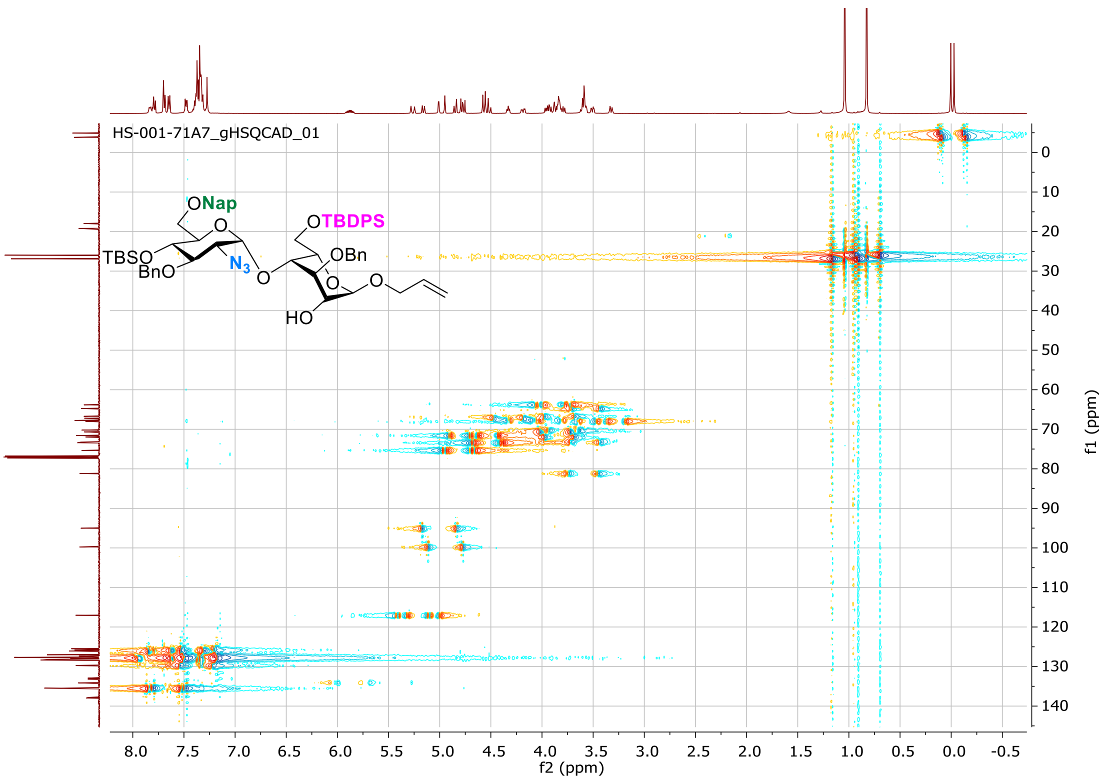

^{1}H-Coupled gHSQC NMR spectrum of compound **S3**

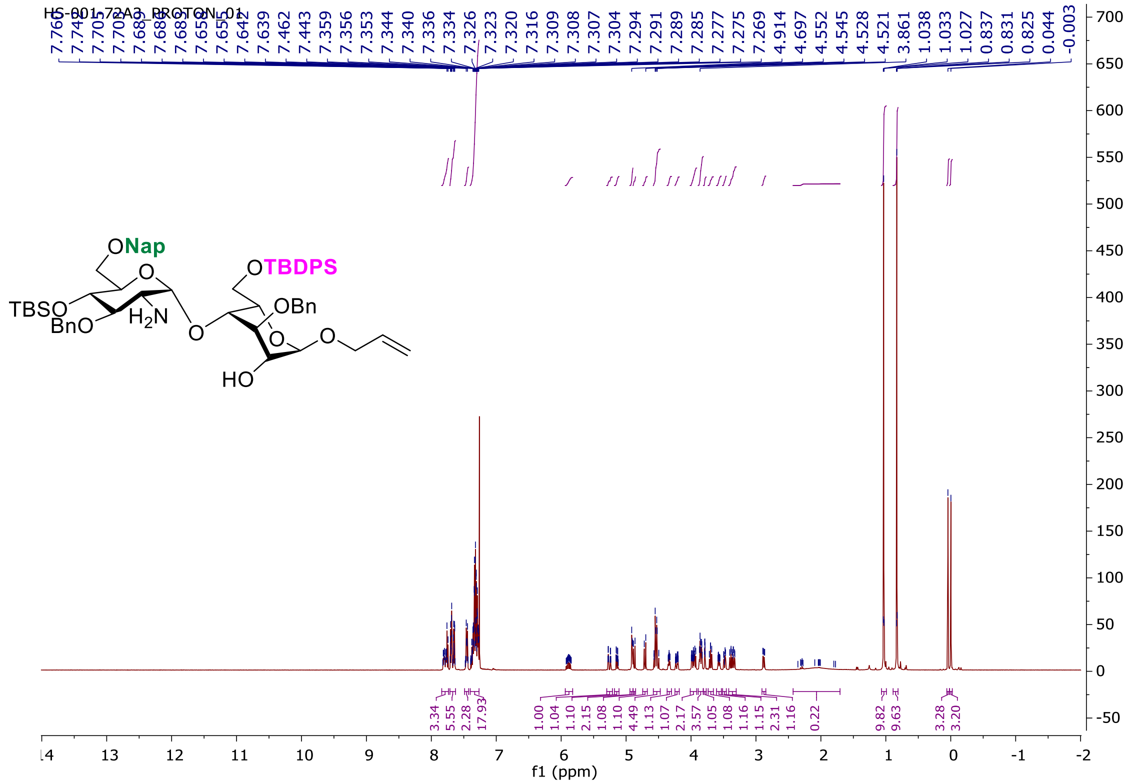

^1H NMR spectrum of compound **S4**

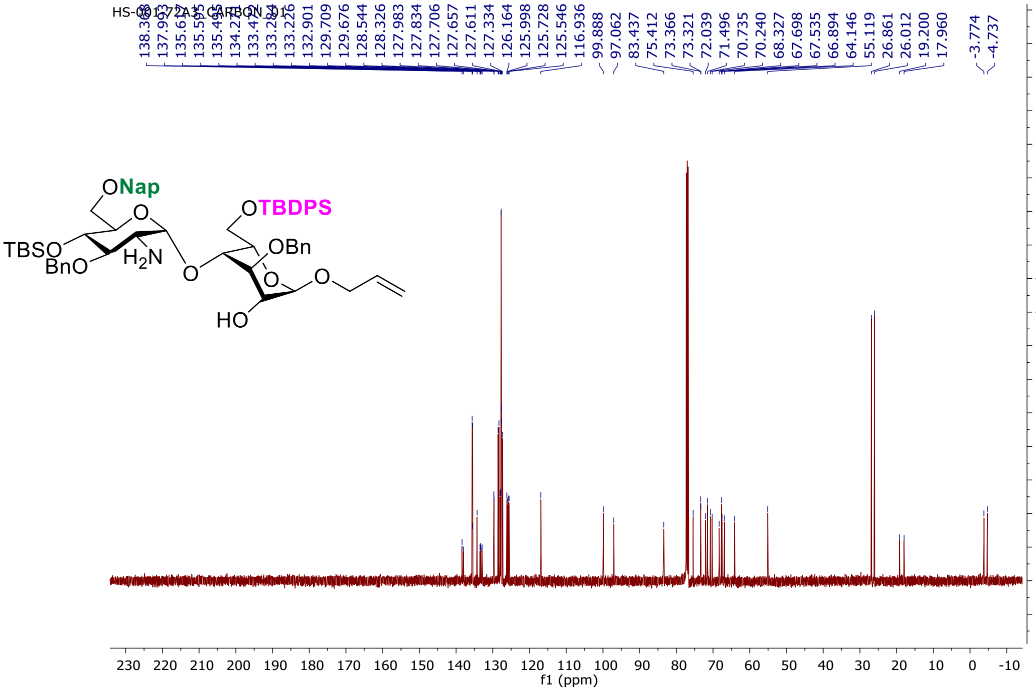

^{13}C NMR spectrum of compound **S4**

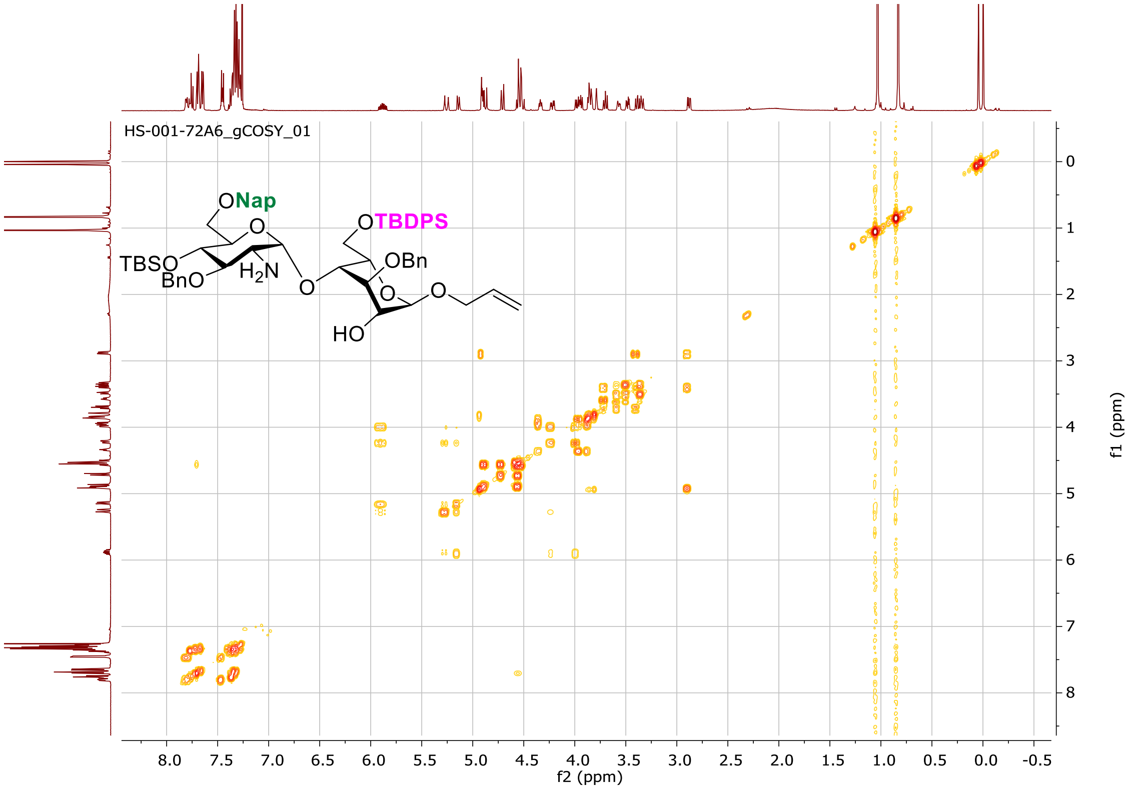

gCOSY NMR spectrum of compound **S4**

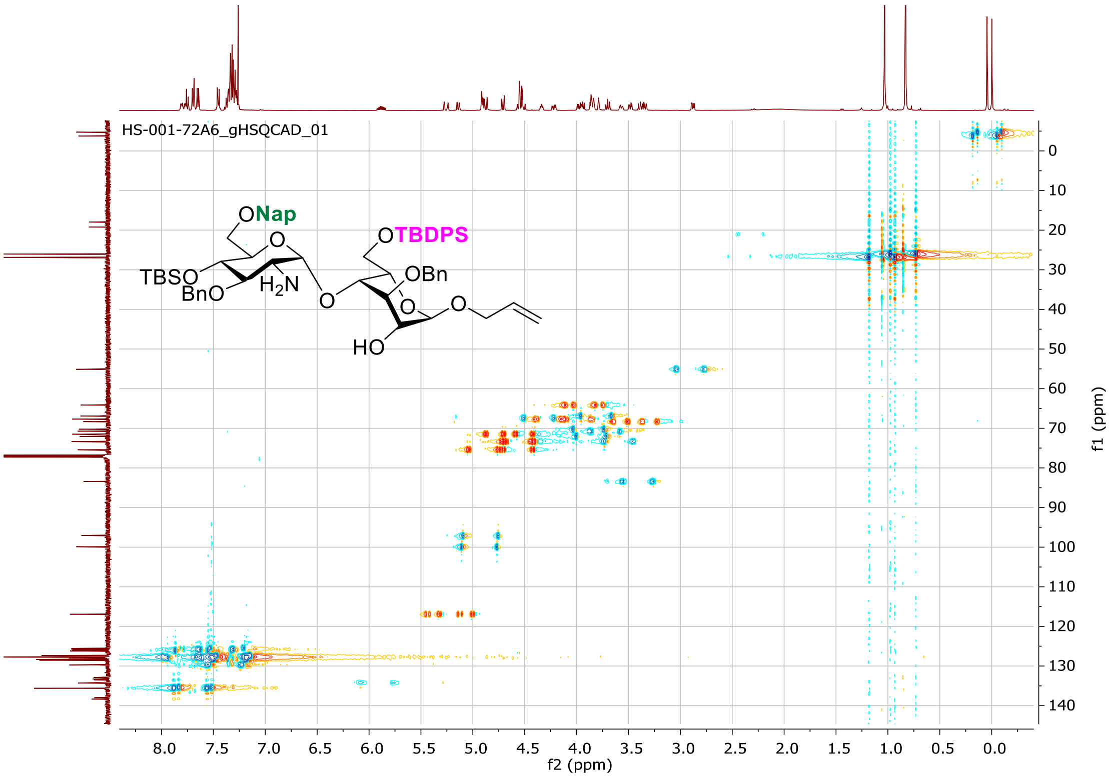

^{1}H-Coupled gHSQC NMR spectrum of compound **S4**

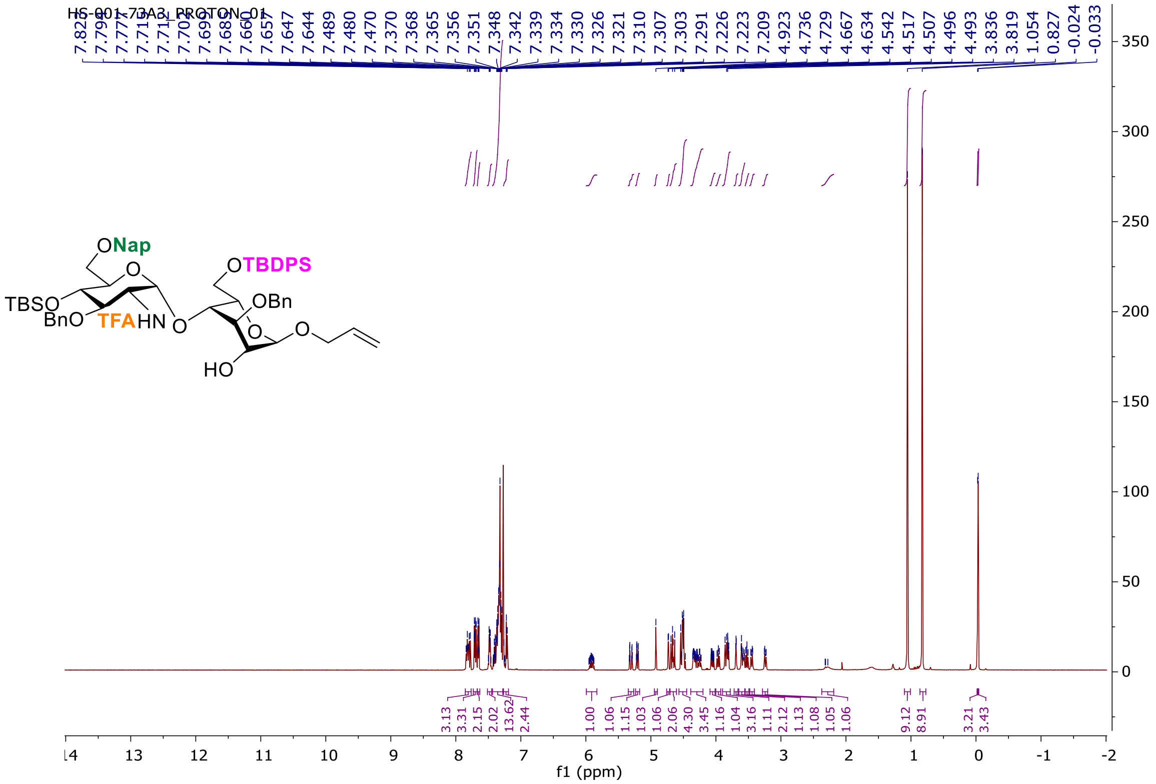

^{1}H NMR spectrum of compound **S5**

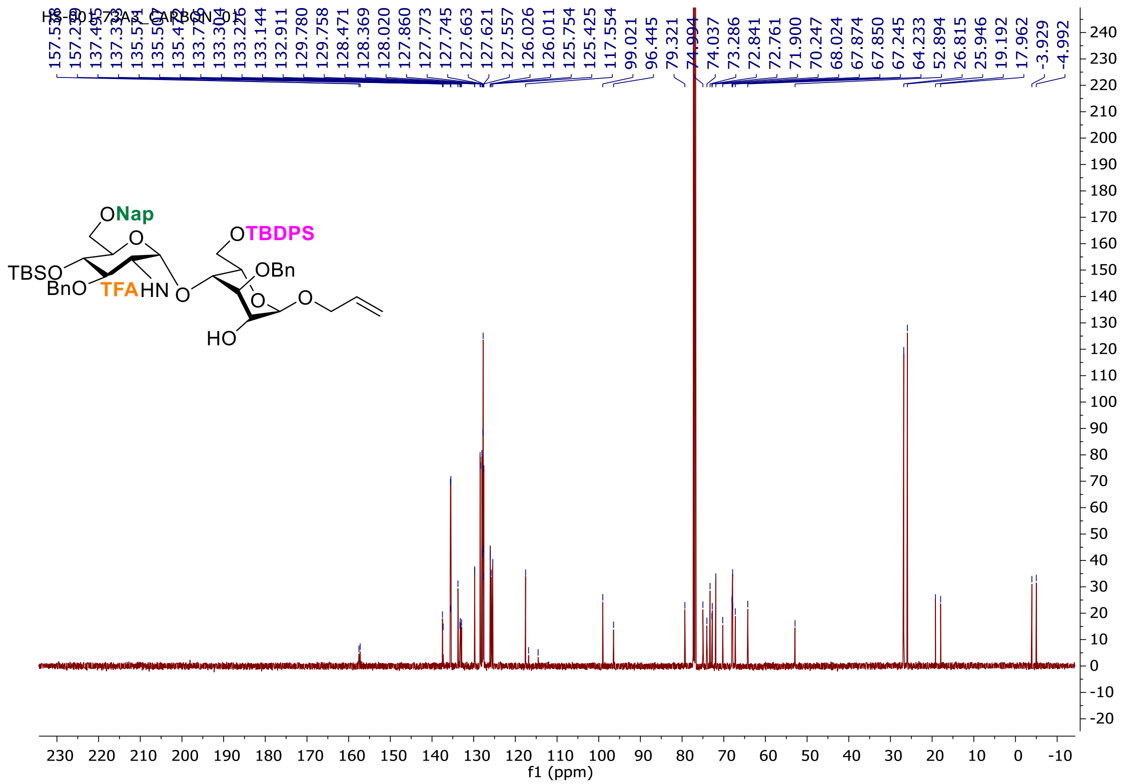

^{13}C NMR spectrum of compound **S5**

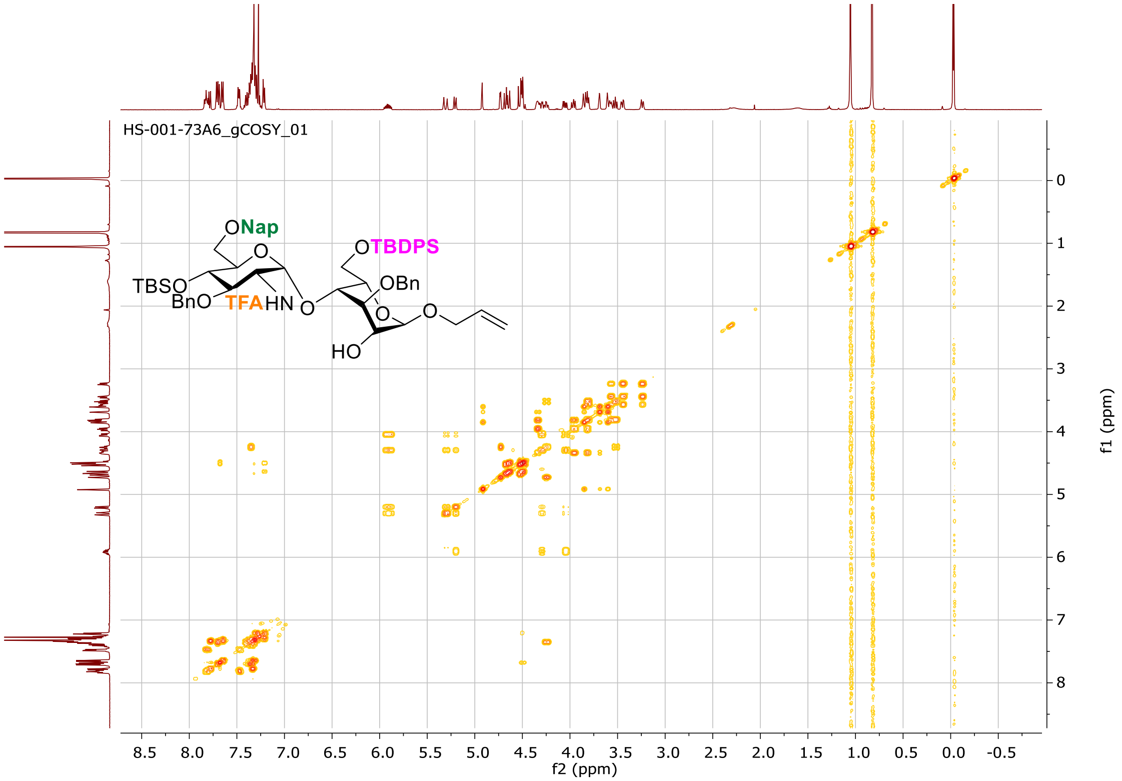

gCOSY NMR spectrum of compound **S5**

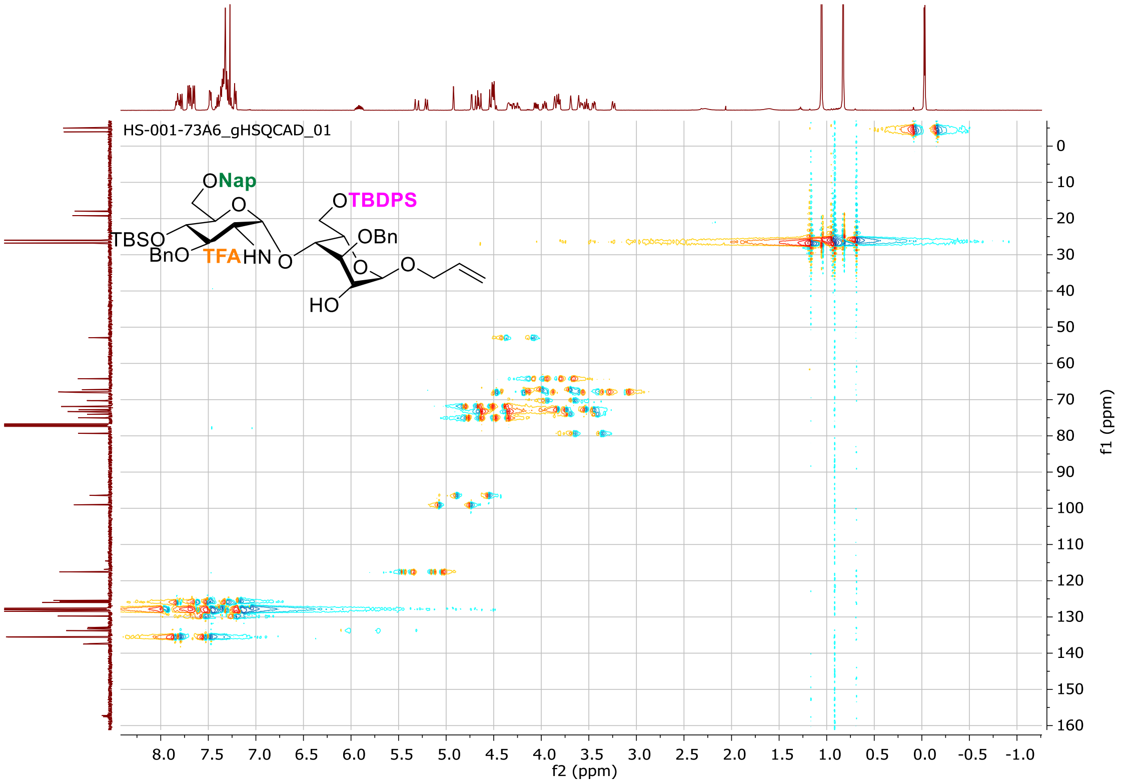

^{1}H-Coupled gHSQC NMR spectrum of compound **S5**

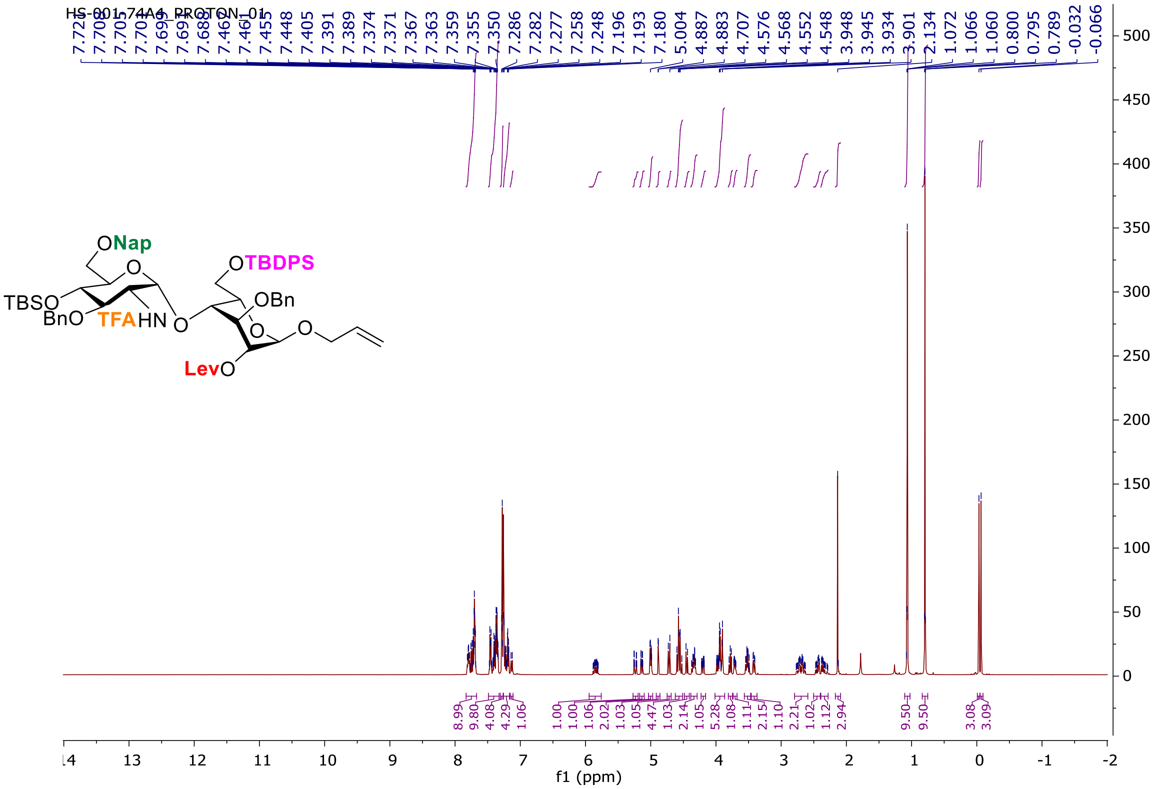

^{1}H NMR spectrum of compound **S6**

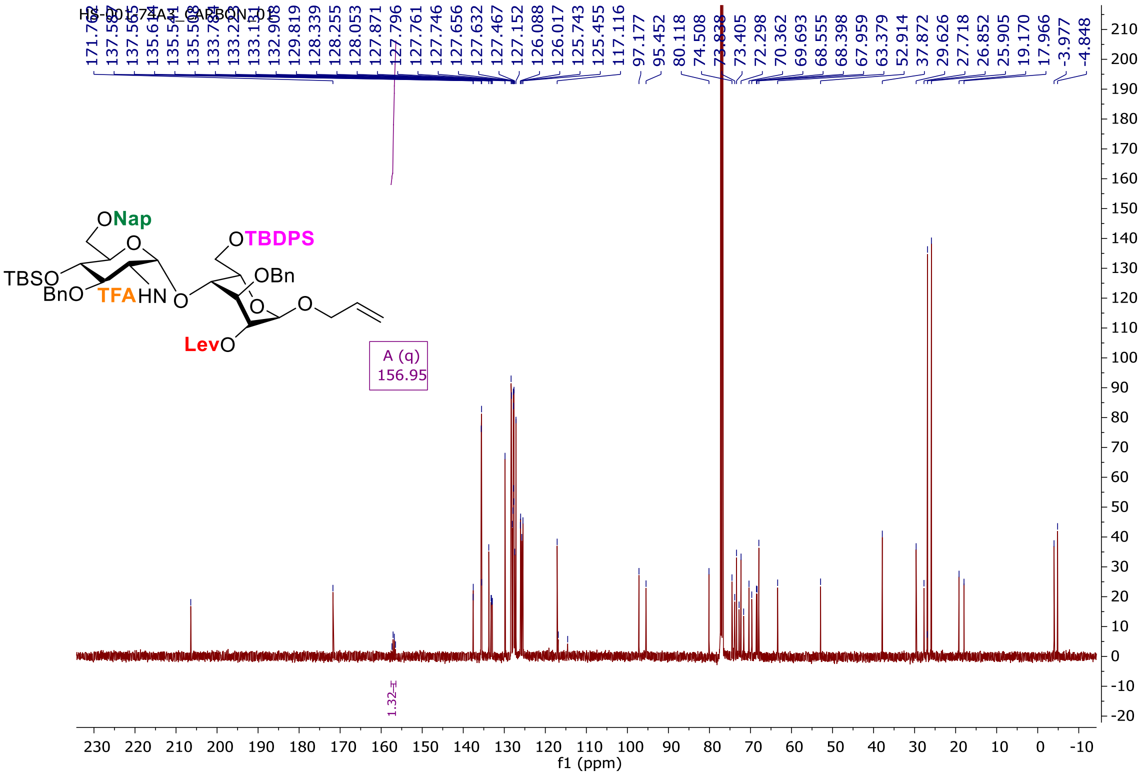

^{13}C NMR spectrum of compound **S6**

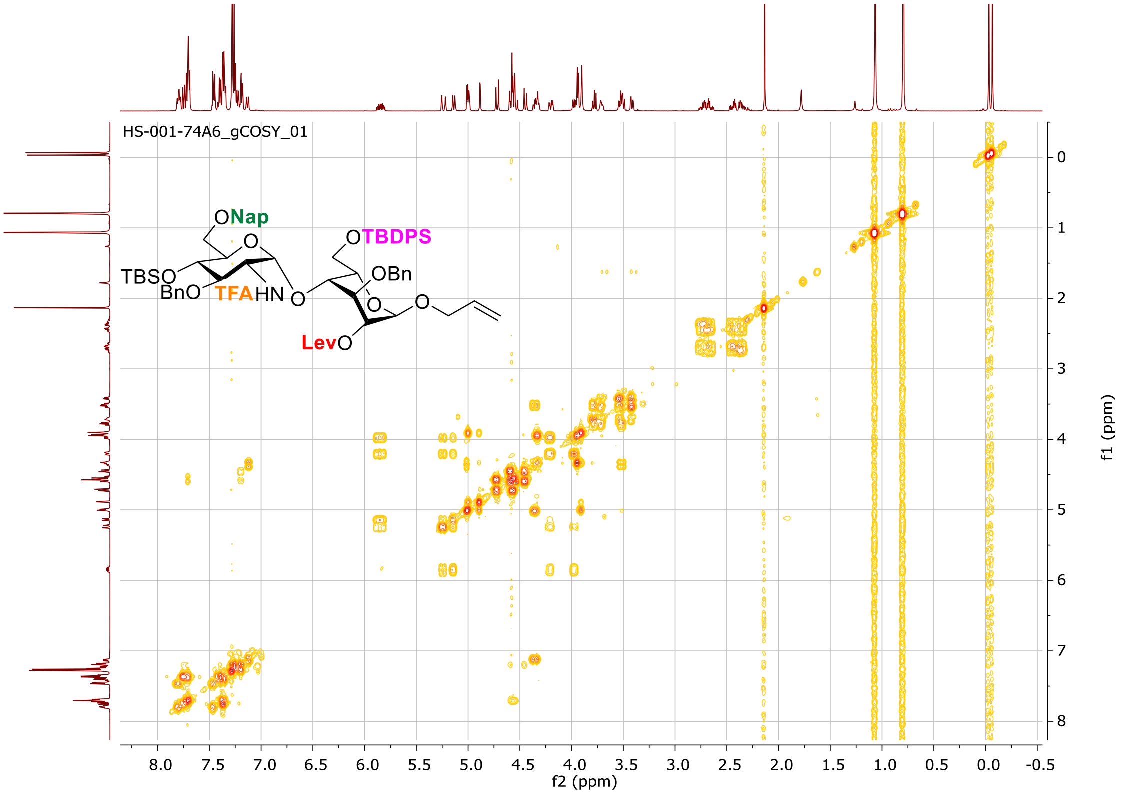

gCOSY NMR spectrum of compound **S6**

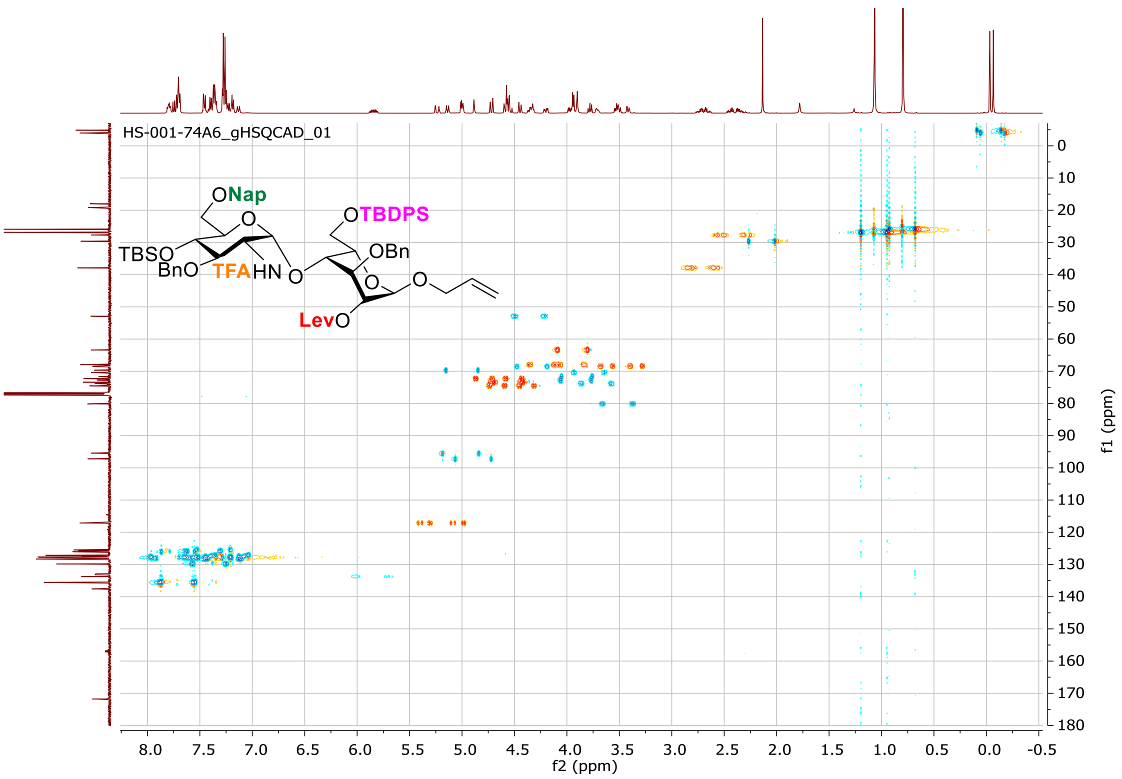

^{1}H-Coupled gHSQC NMR spectrum of compound **S6**

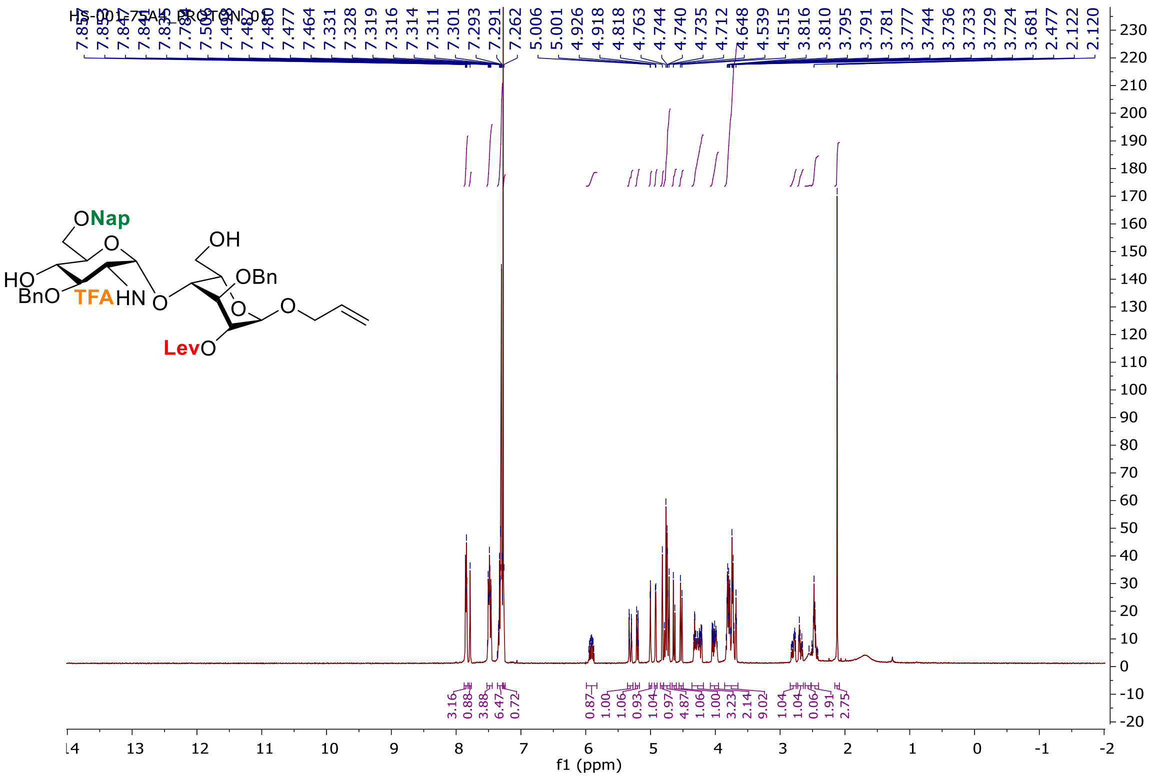

^{1}H NMR spectrum of compound **S7**

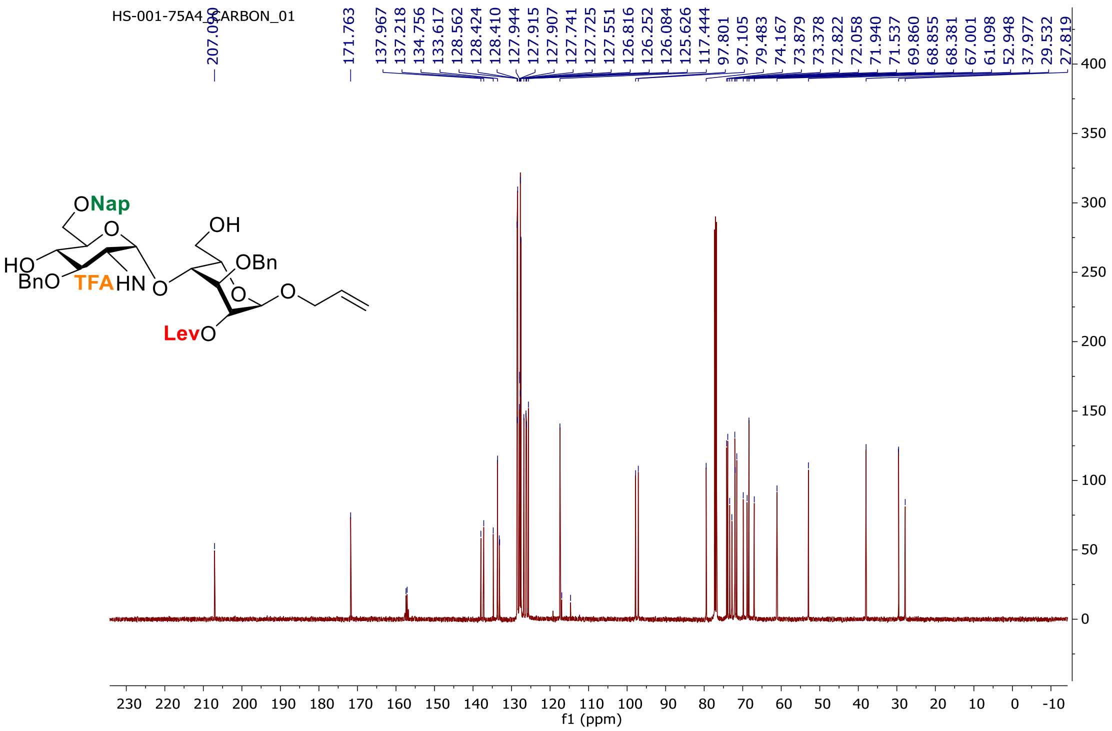

^{13}C NMR spectrum of compound **S7**

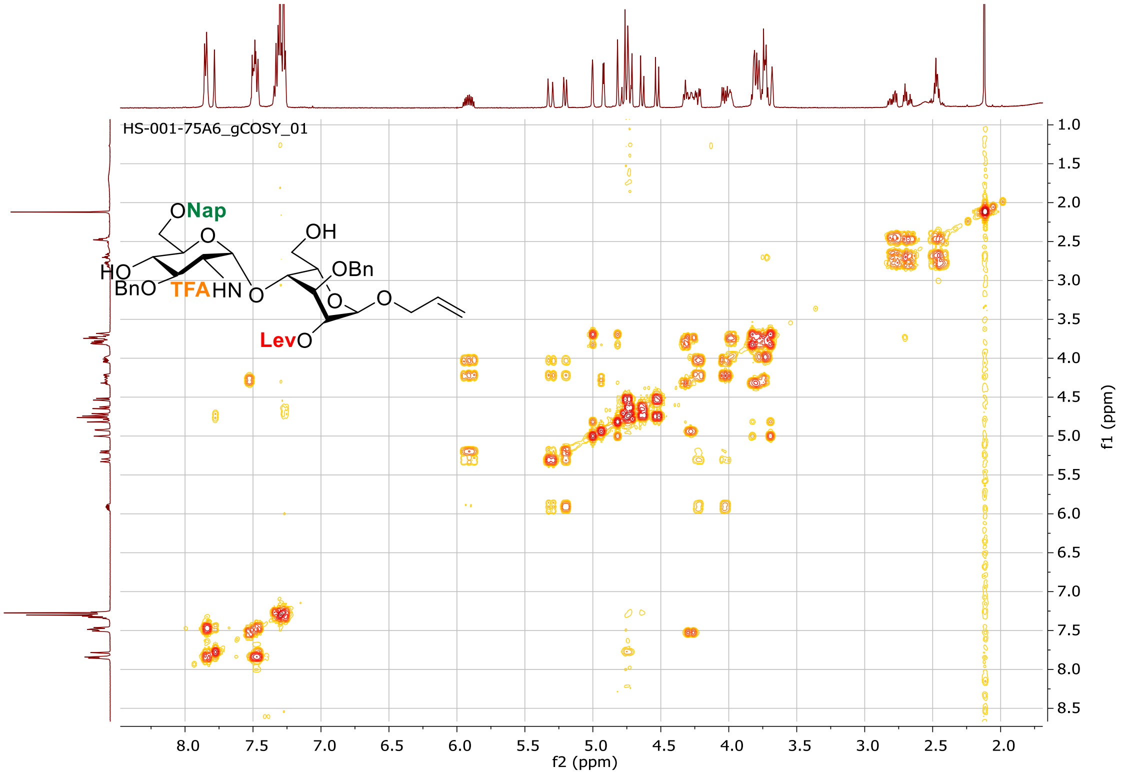

gCOSY NMR spectrum of compound **S7**

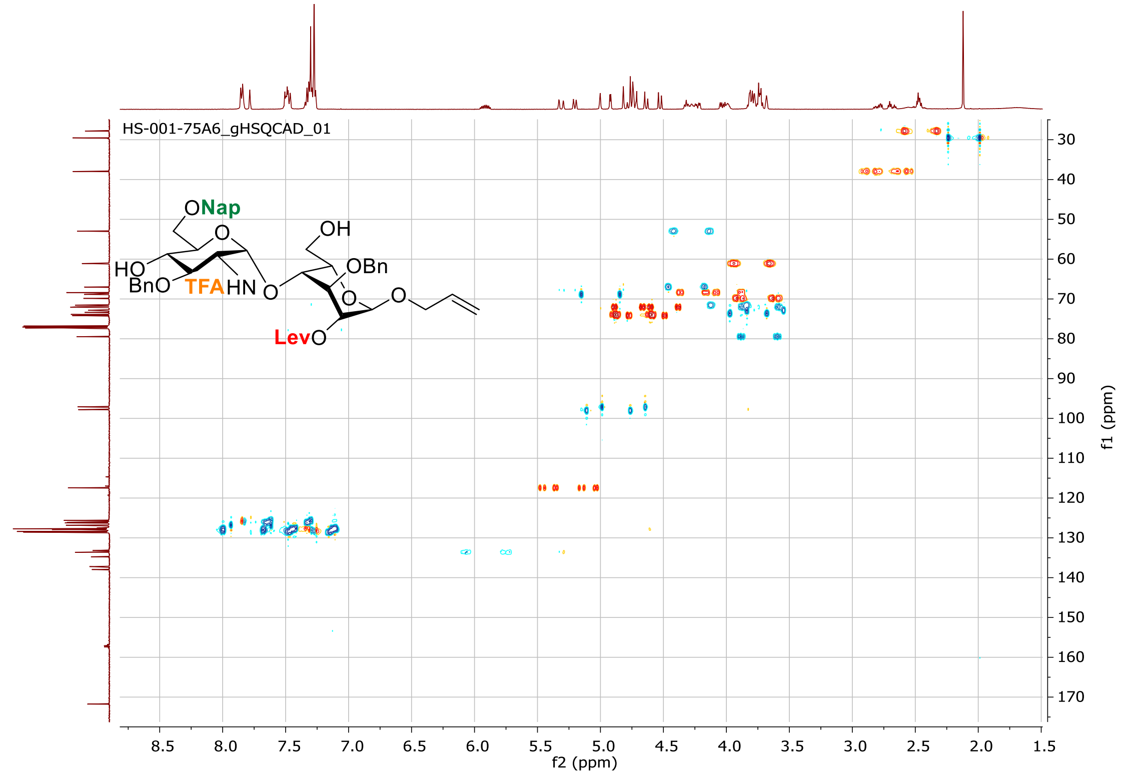

^{1}H-Coupled gHSQC NMR spectrum of compound **S7**

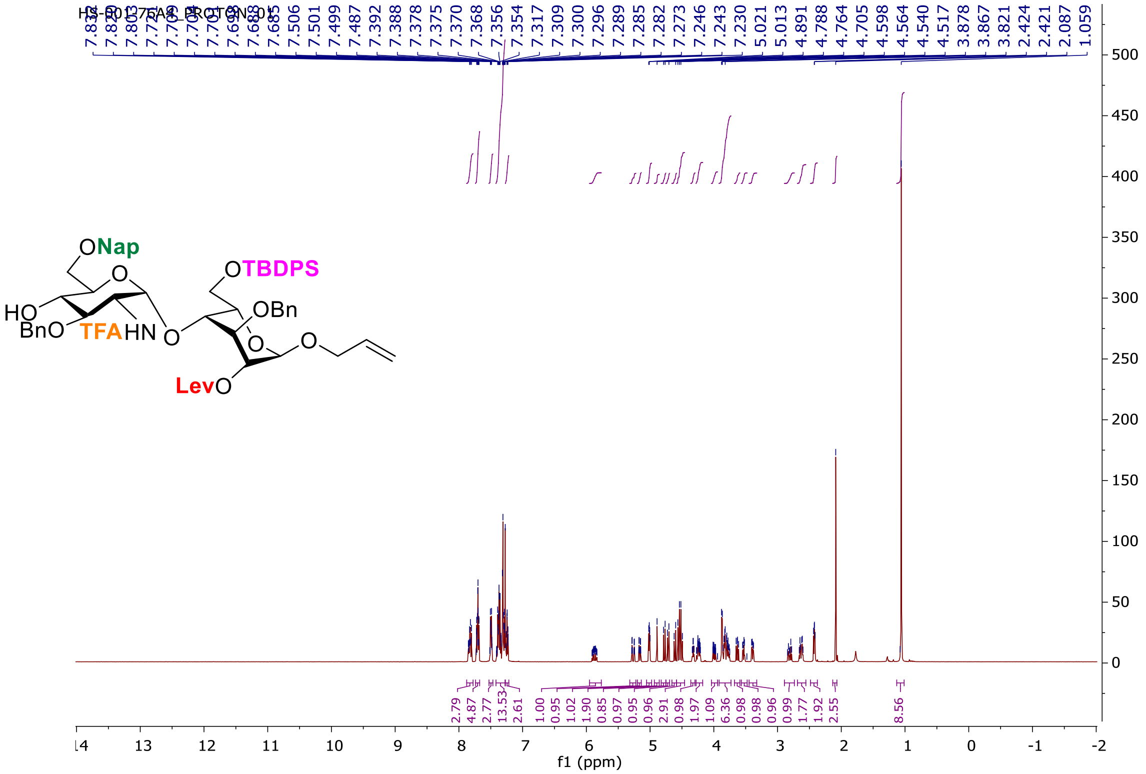

^{1}H NMR spectrum of compound **14**

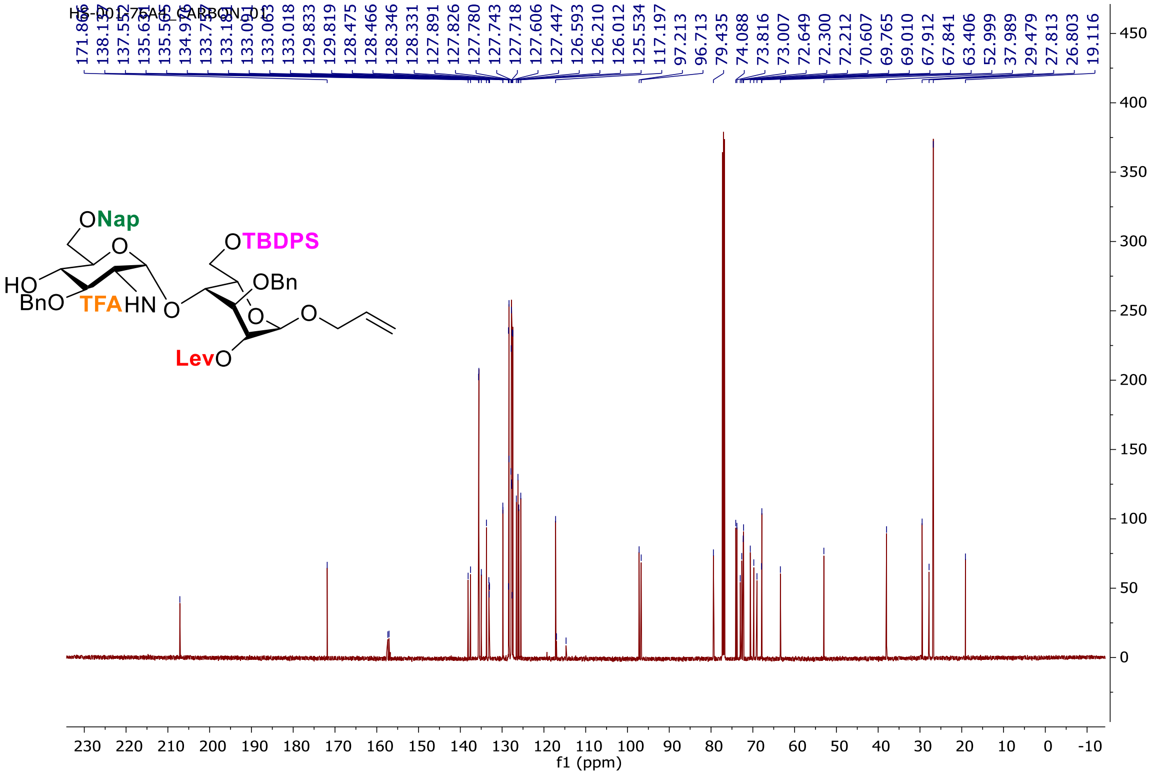

^{13}C NMR spectrum of compound **14**

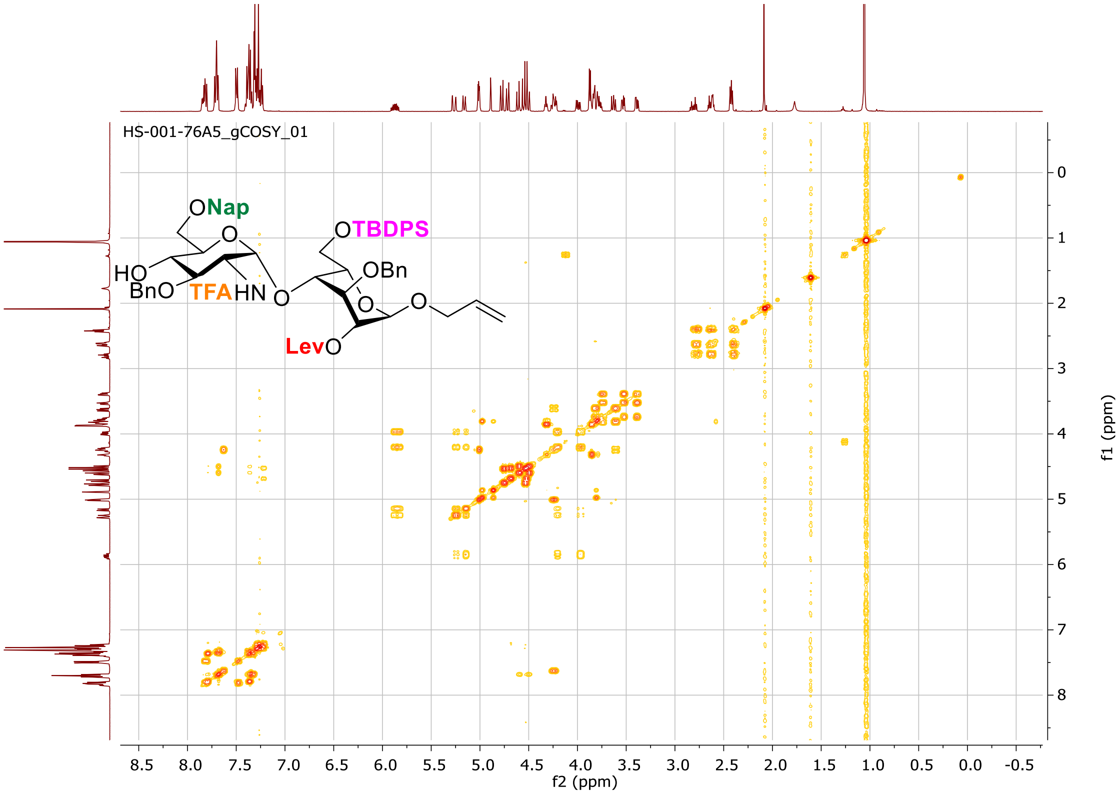

gCOSY NMR spectrum of compound **14**

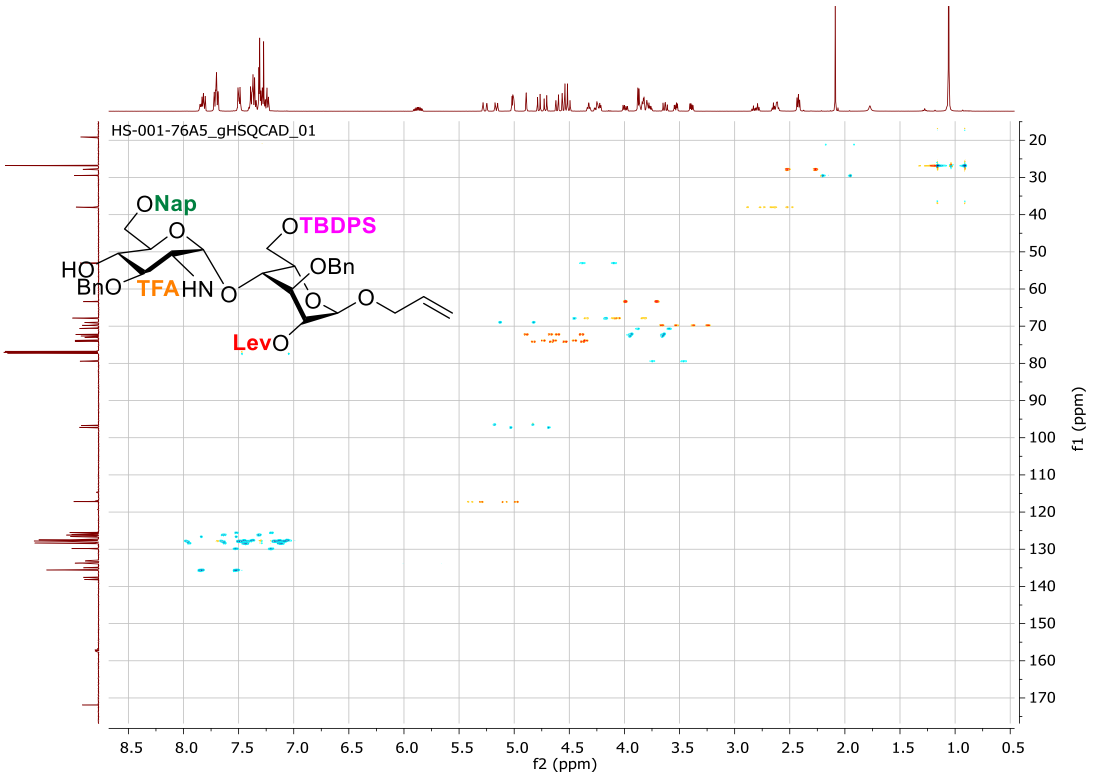

^{1}H-Coupled gHSQC NMR spectrum of compound **14**

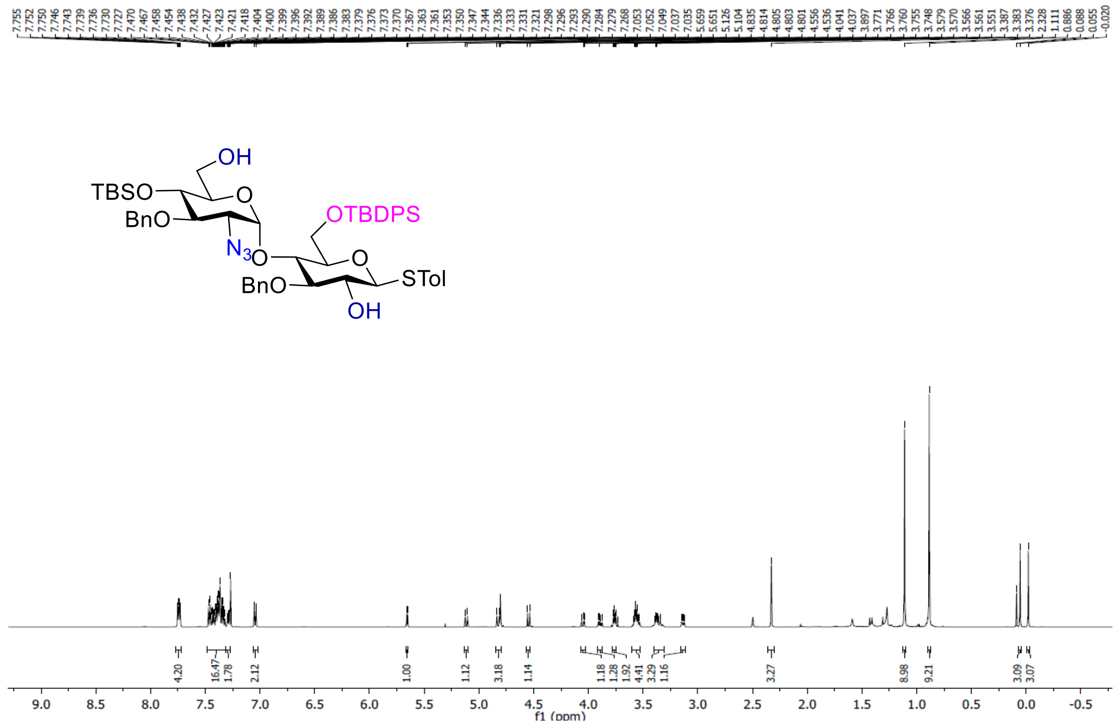

^{1}H NMR spectrum of compound **S9**

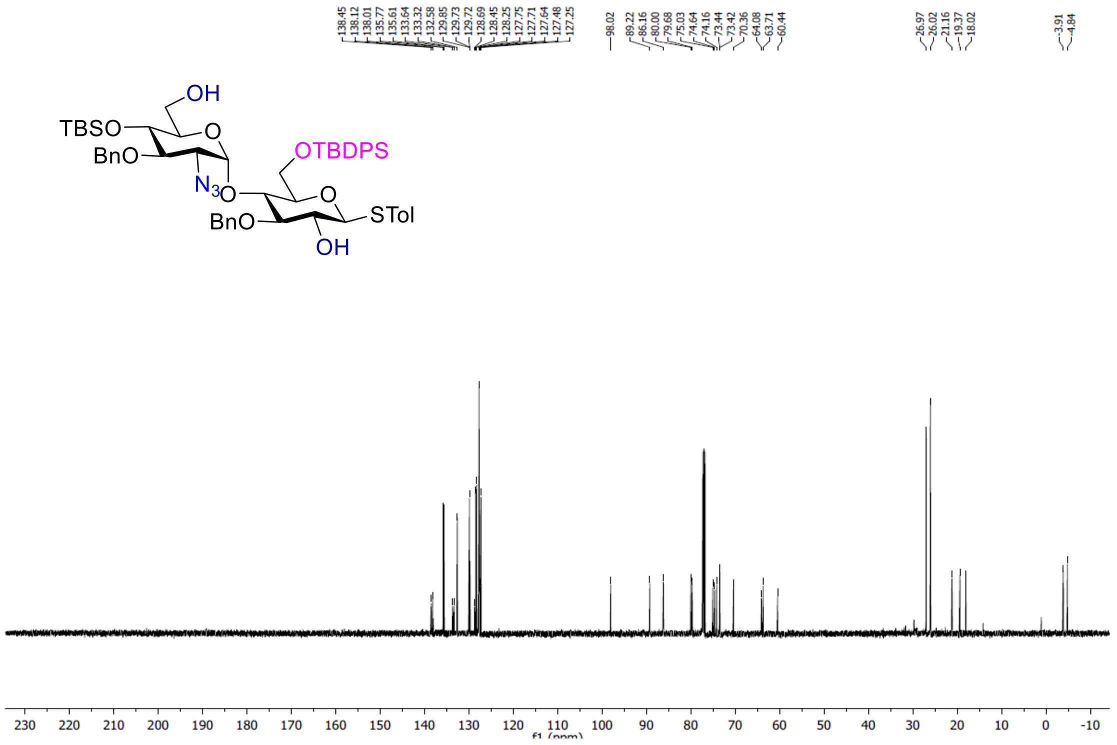

^{13}C NMR spectrum of compound **S9**

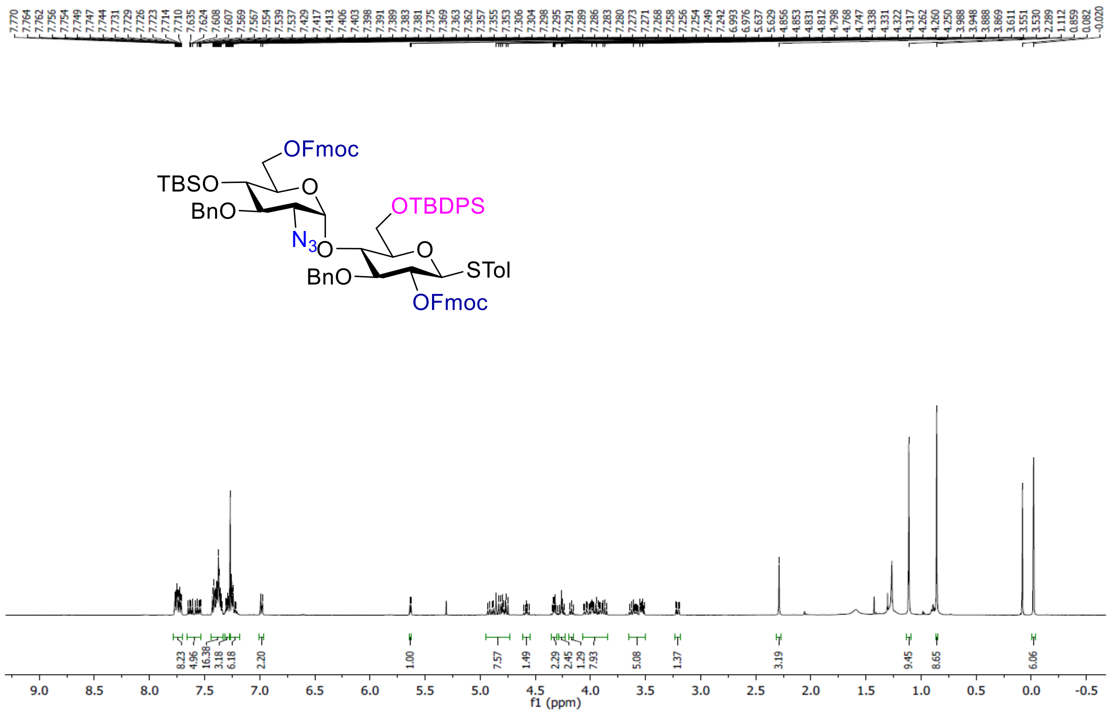

^{1}H NMR spectrum of compound **15**

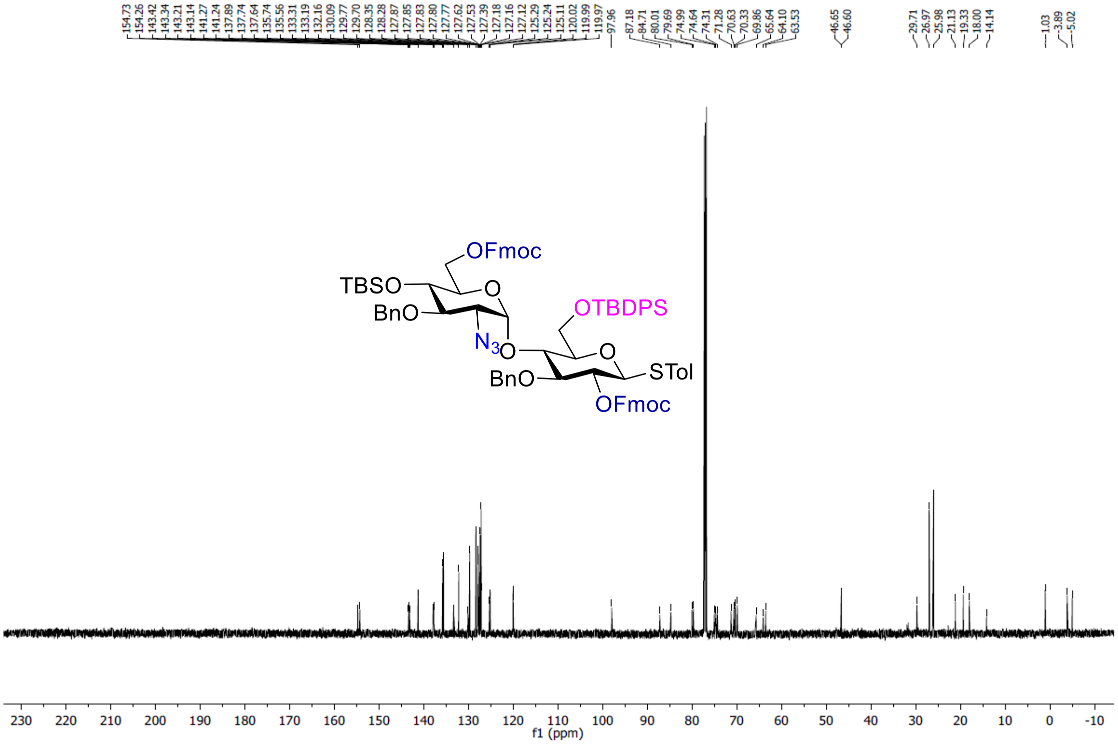

^{13}C NMR of compound **15**

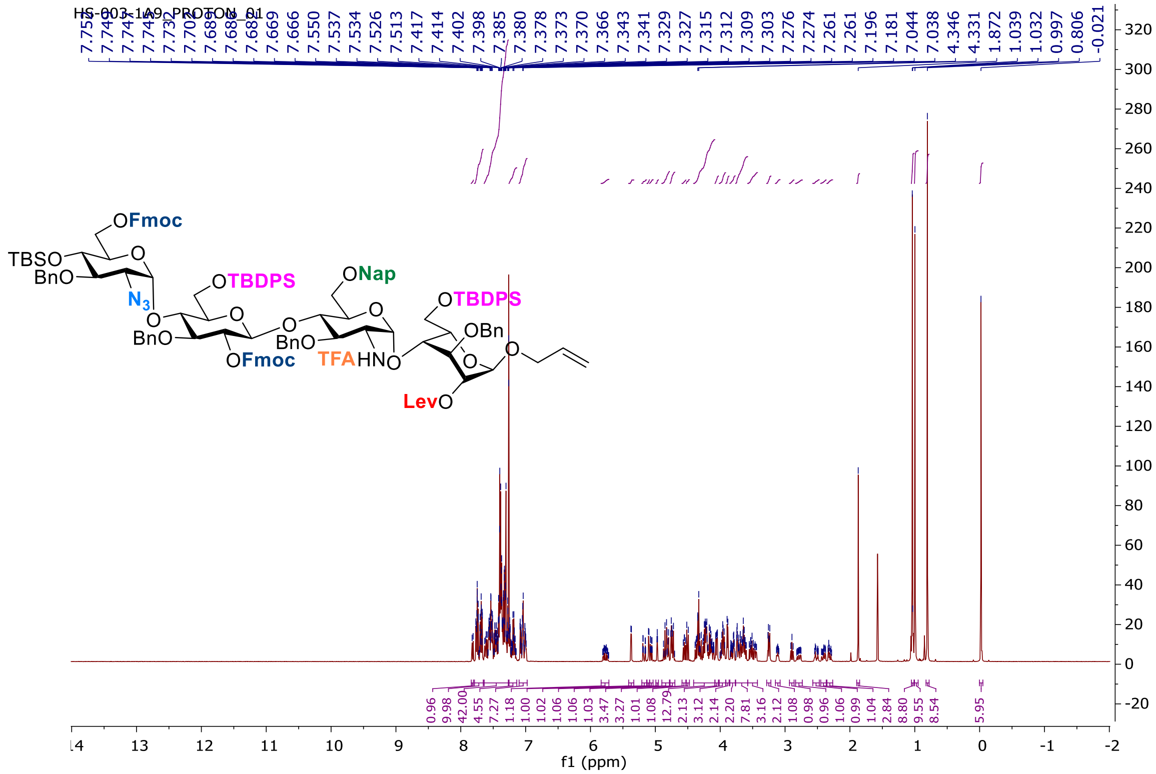

^{1}H NMR spectrum of compound **19**

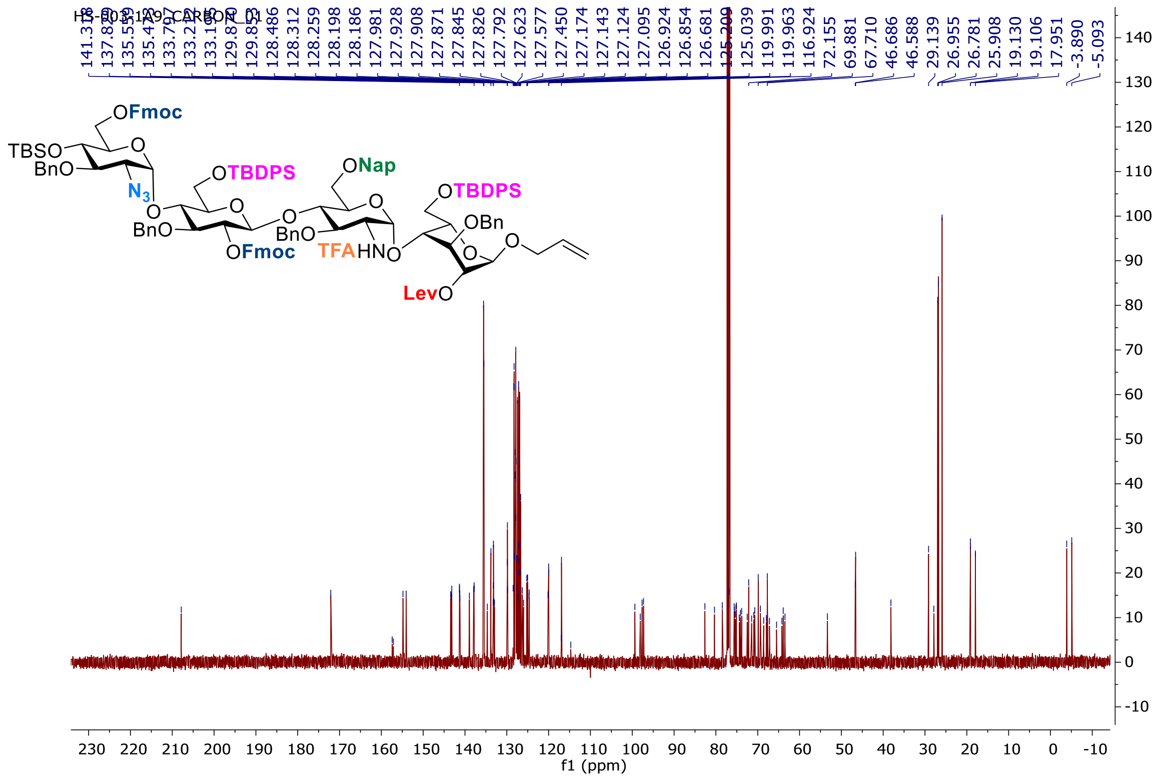

^{13}C NMR spectrum of compound **19**

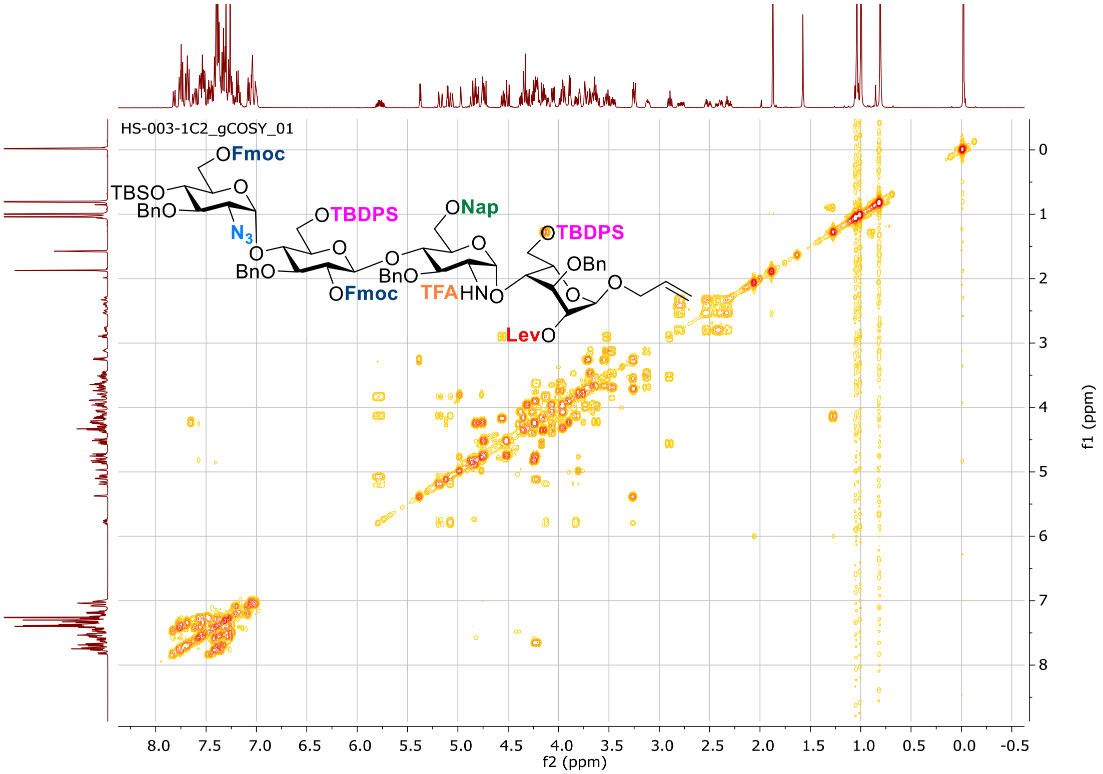

gCOSY NMR spectrum of compound **19**

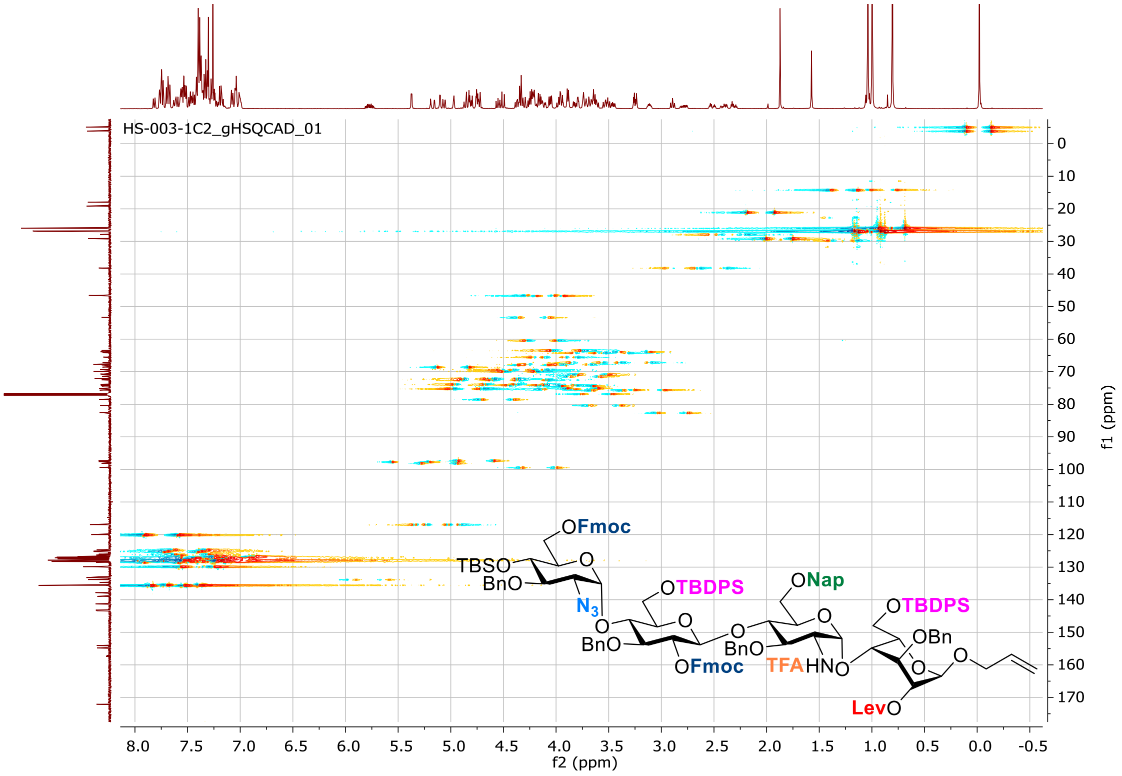

^{1}H-Coupled gHSQC NMR spectrum of compound **19**

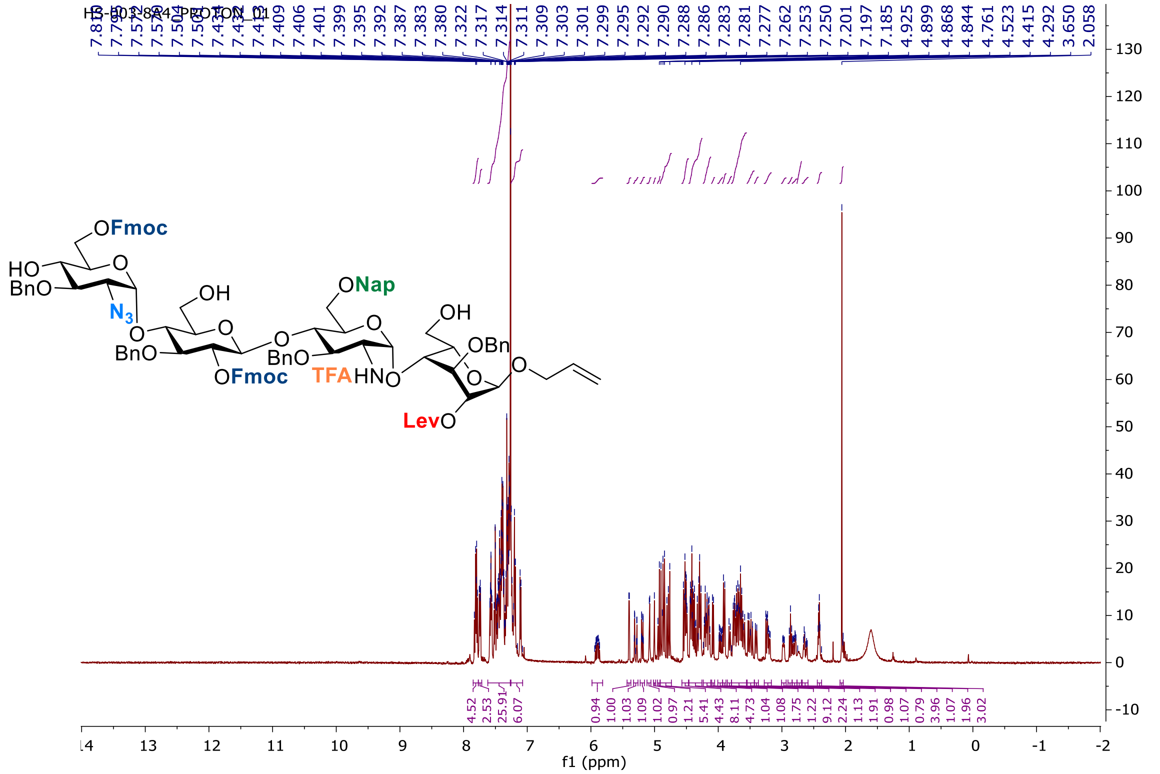

^{1}H NMR spectrum of compound **S10**

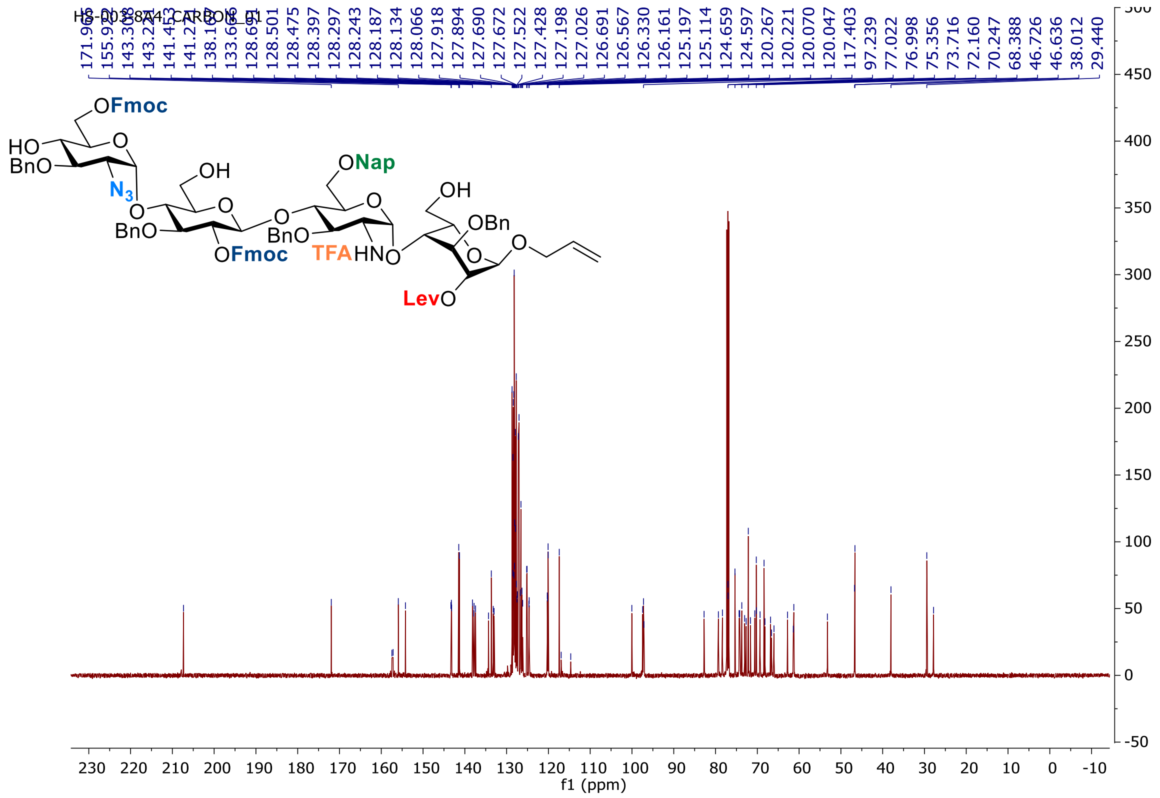

^{13}C NMR spectrum of compound **S10**

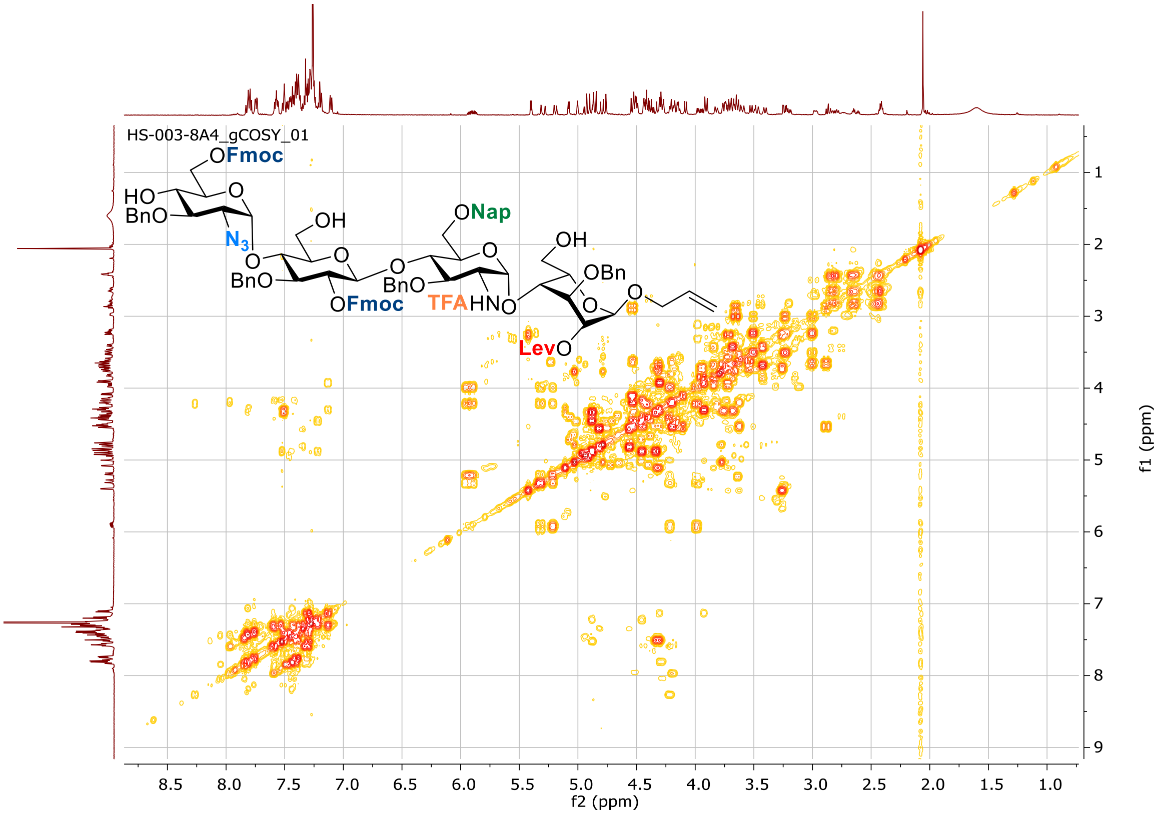

gCOSY NMR spectrum of compound **S10**

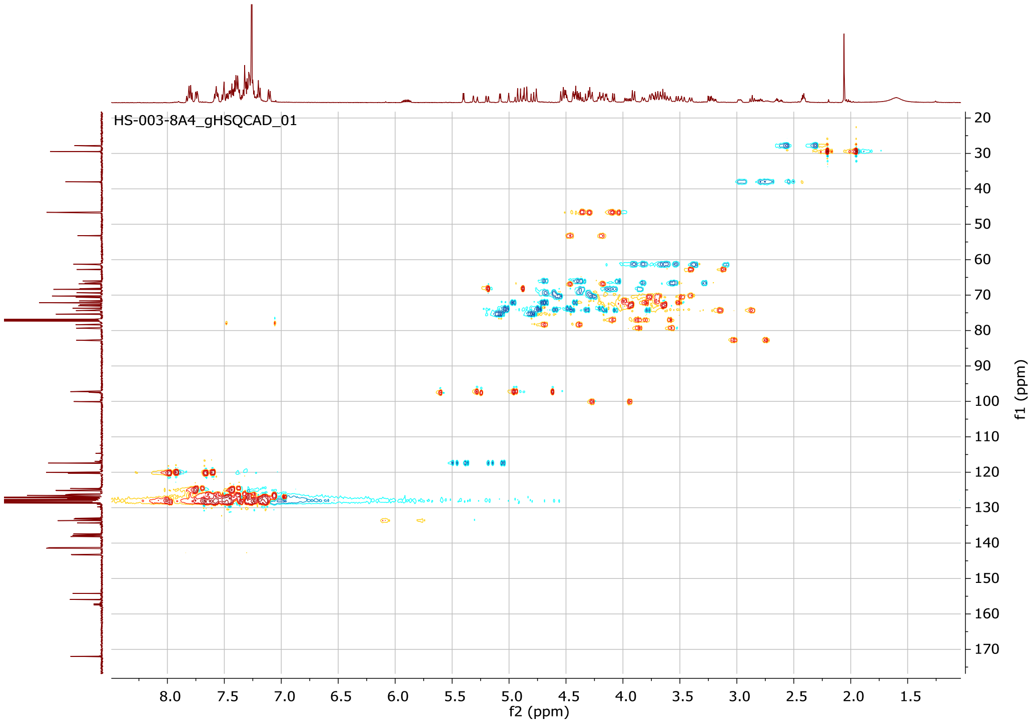

^{1}H-Coupled gHSQC NMR spectrum of compound **S10**

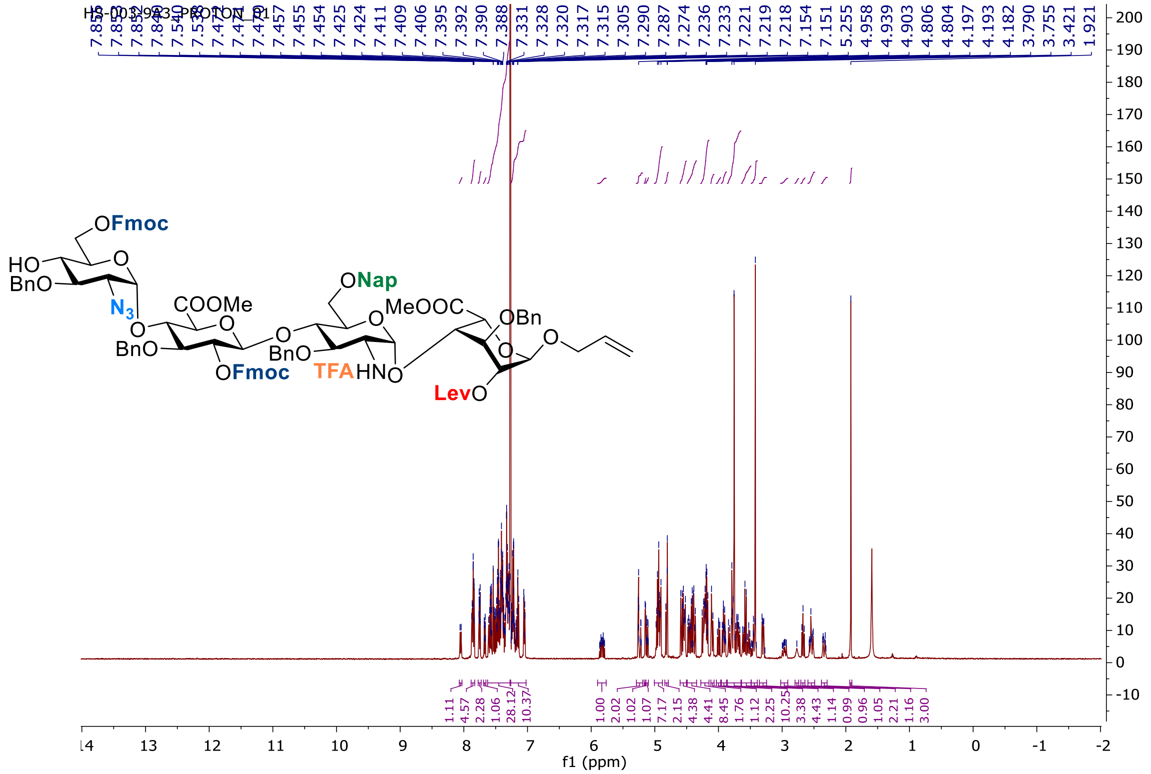

^{1}H NMR spectrum of compound **S11**

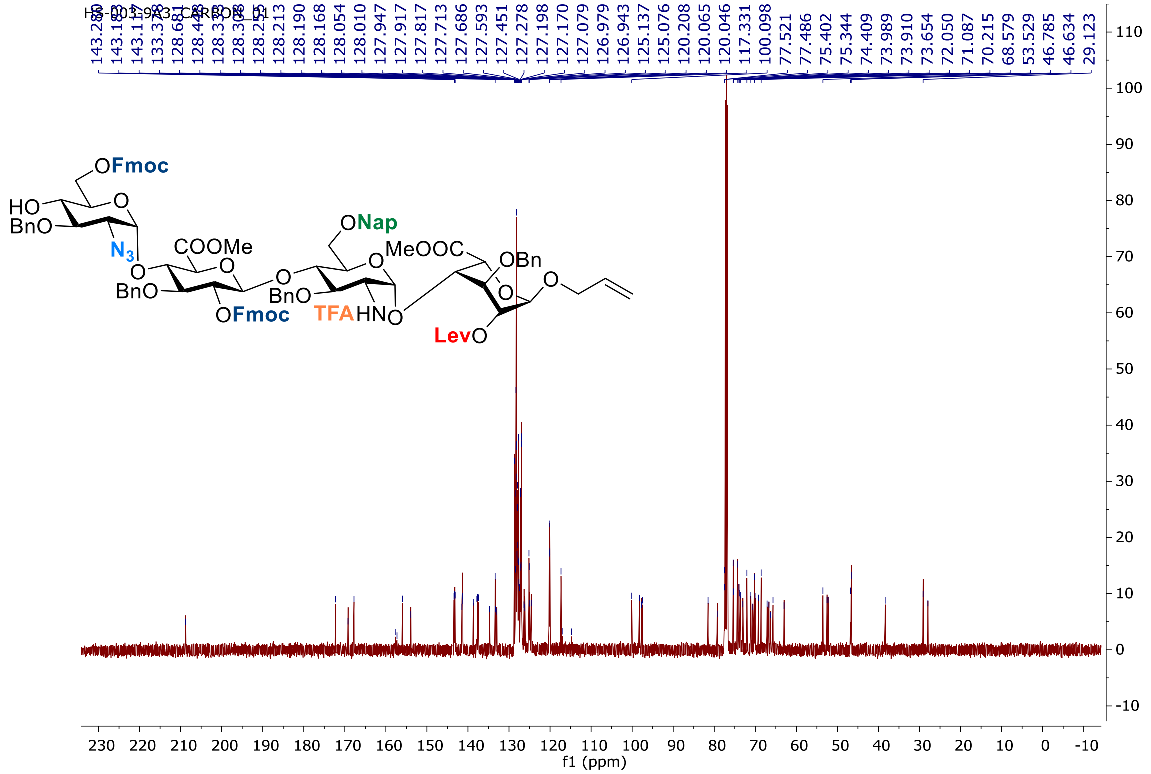

^{13}C NMR spectrum of compound **S11**

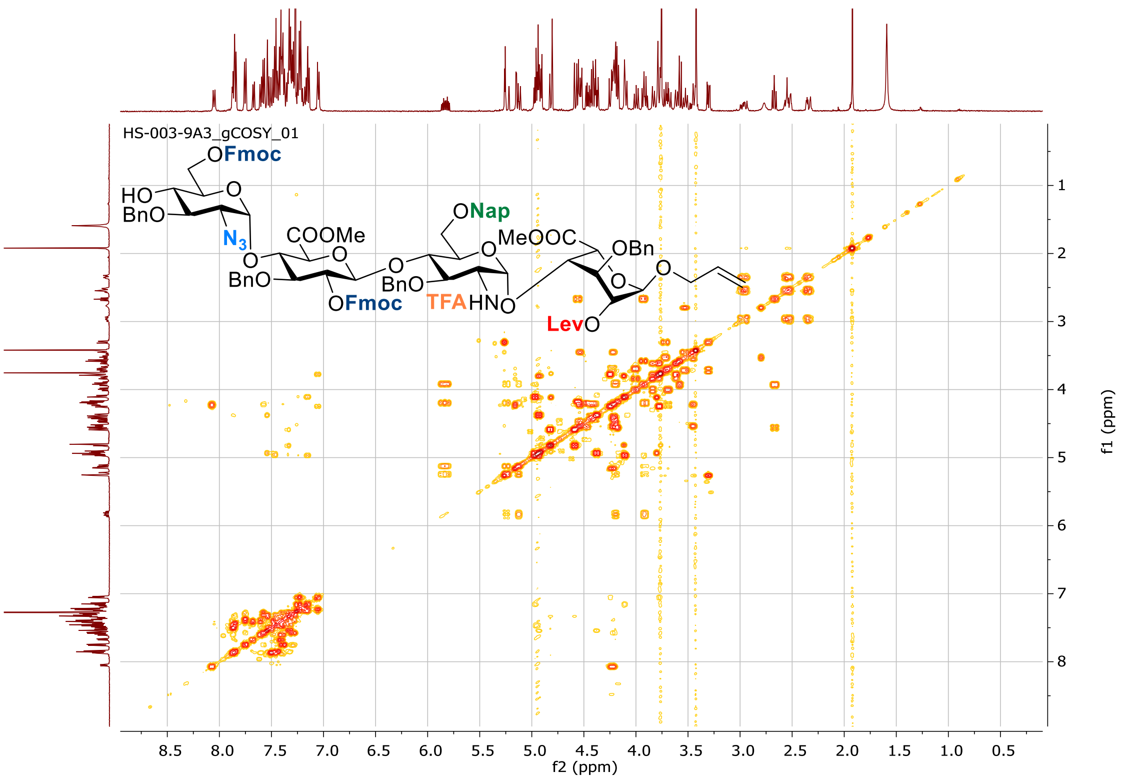

gCOSY NMR spectrum of compound **S11**

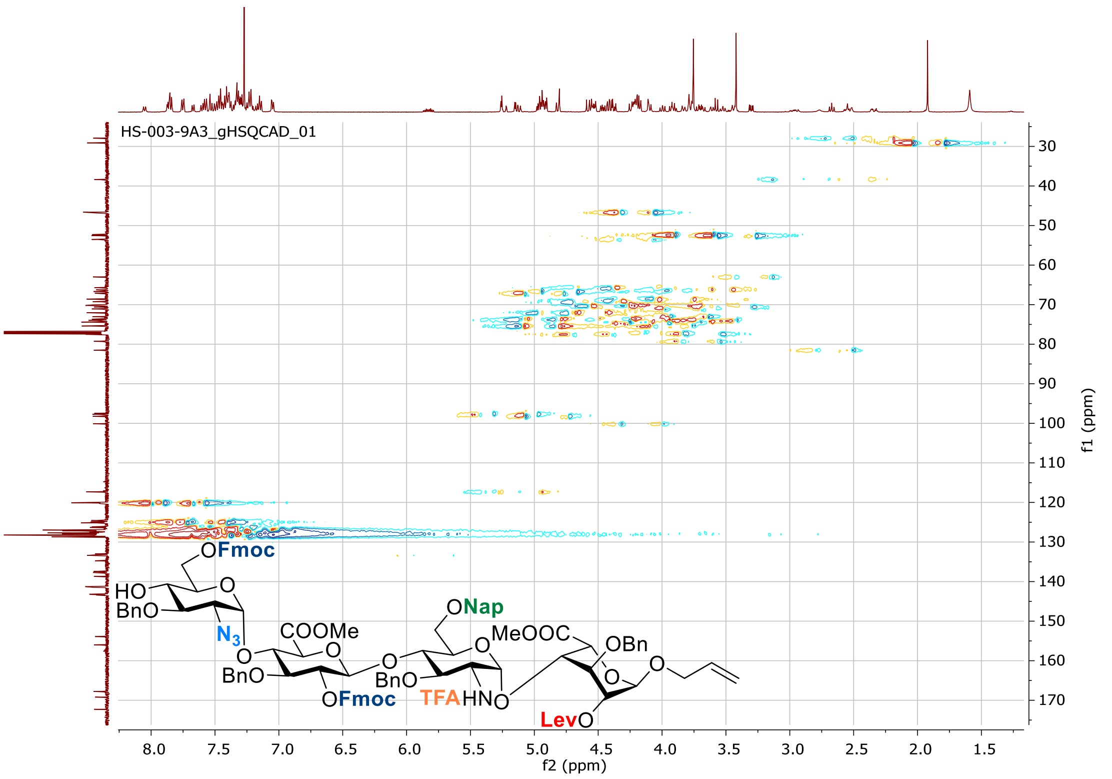

^{1}H-Coupled gHSQC NMR spectrum of compound **S11**

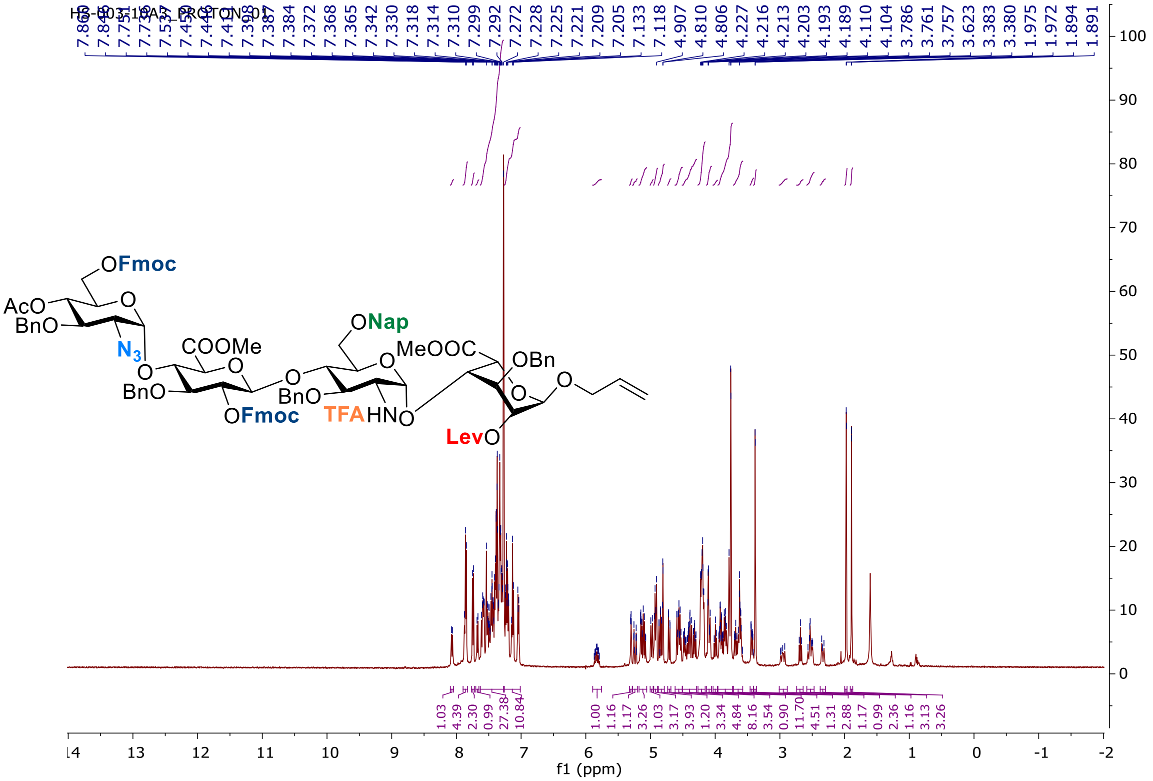

^{1}H NMR spectrum of compound **21**

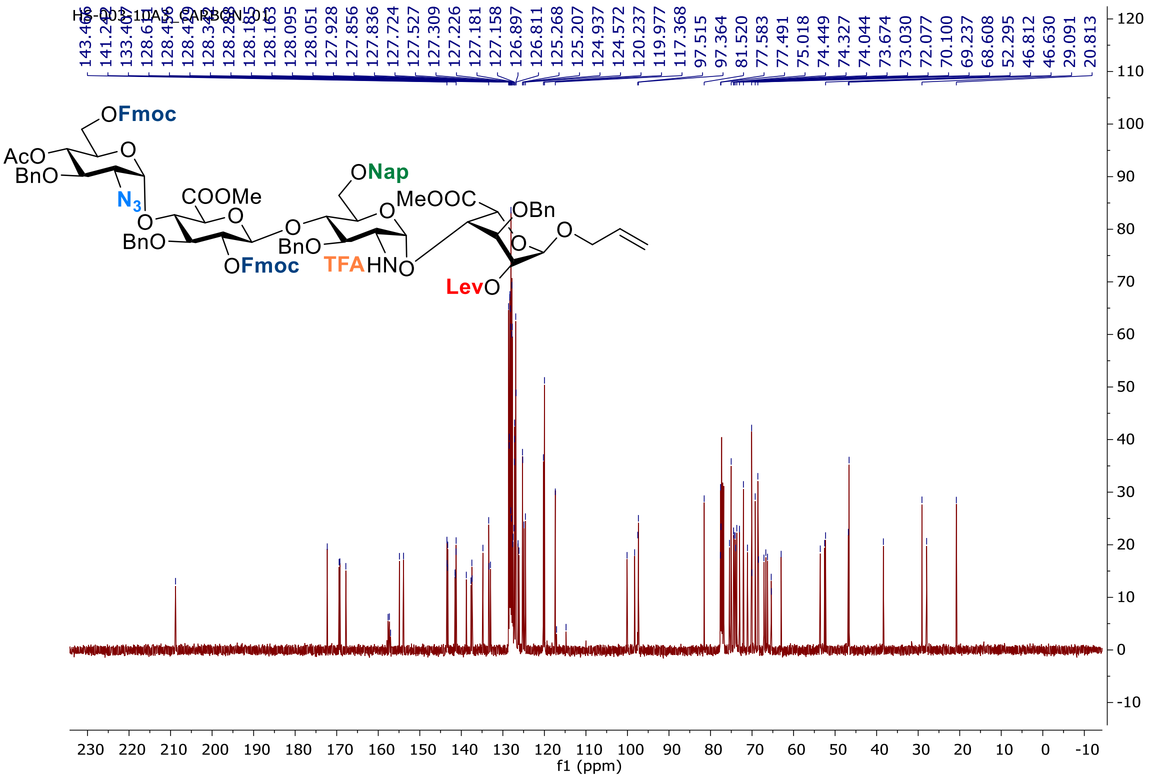

^{13}C NMR spectrum of compound **21**

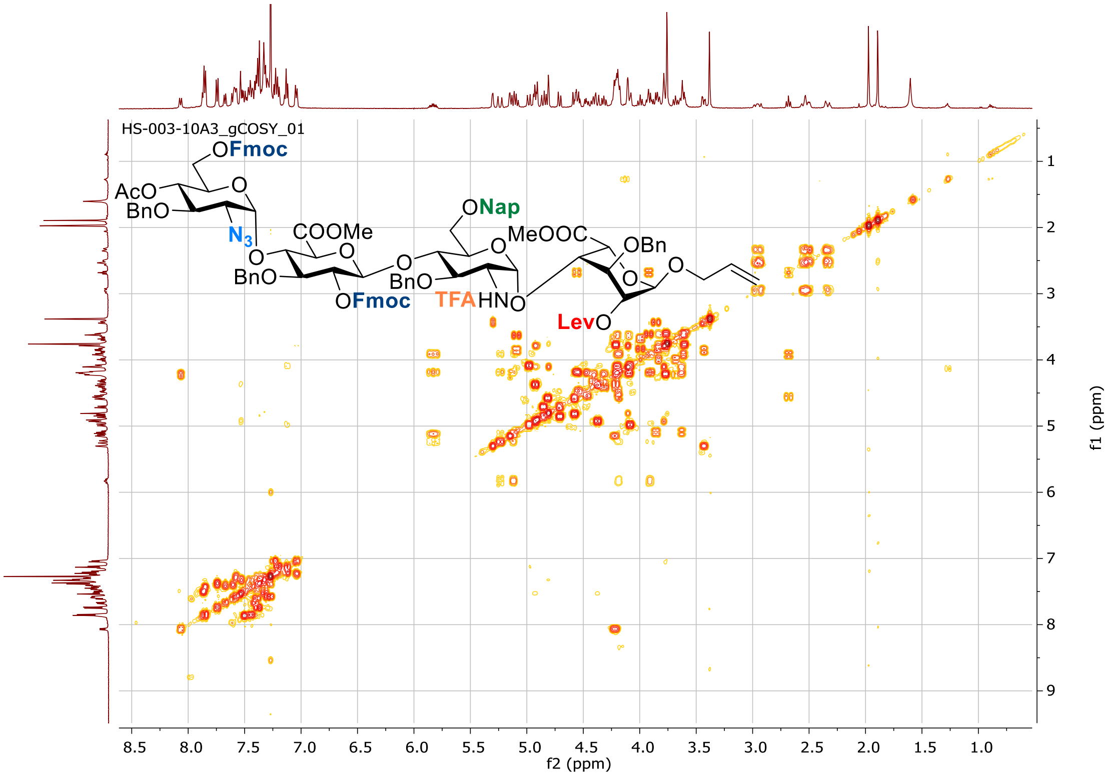

gCOSY NMR spectrum of compound **21**

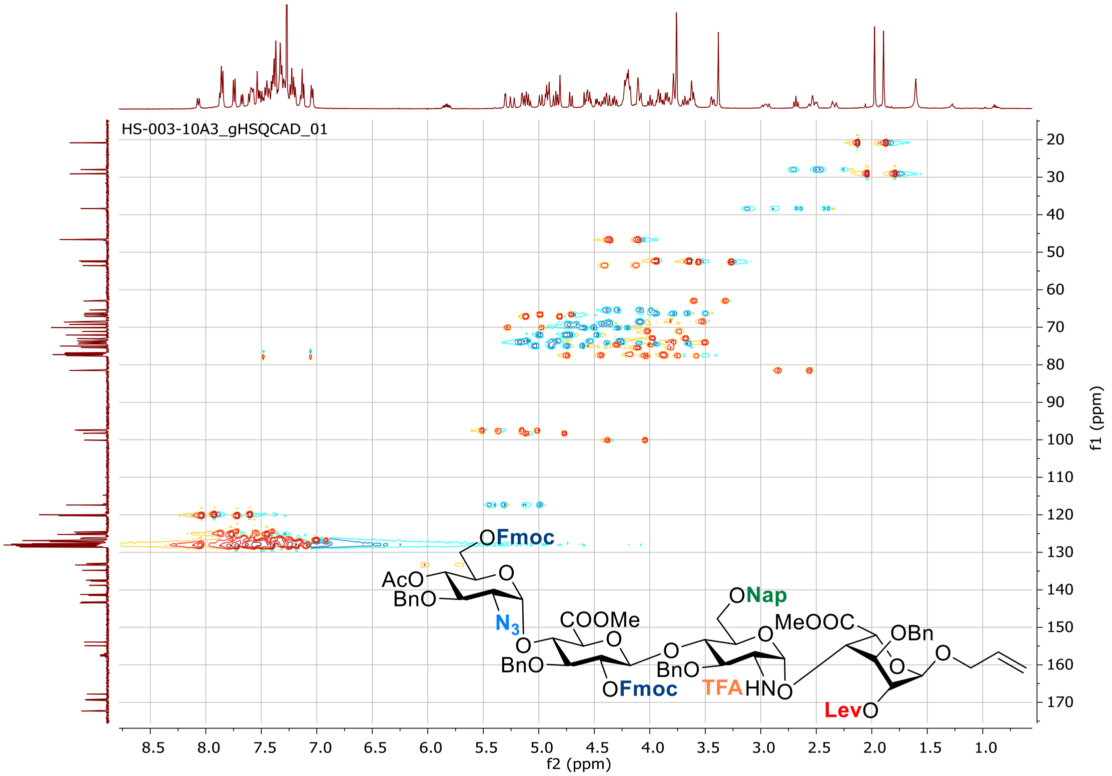

^{1}H-Coupled gHSQC NMR spectrum of compound **21**

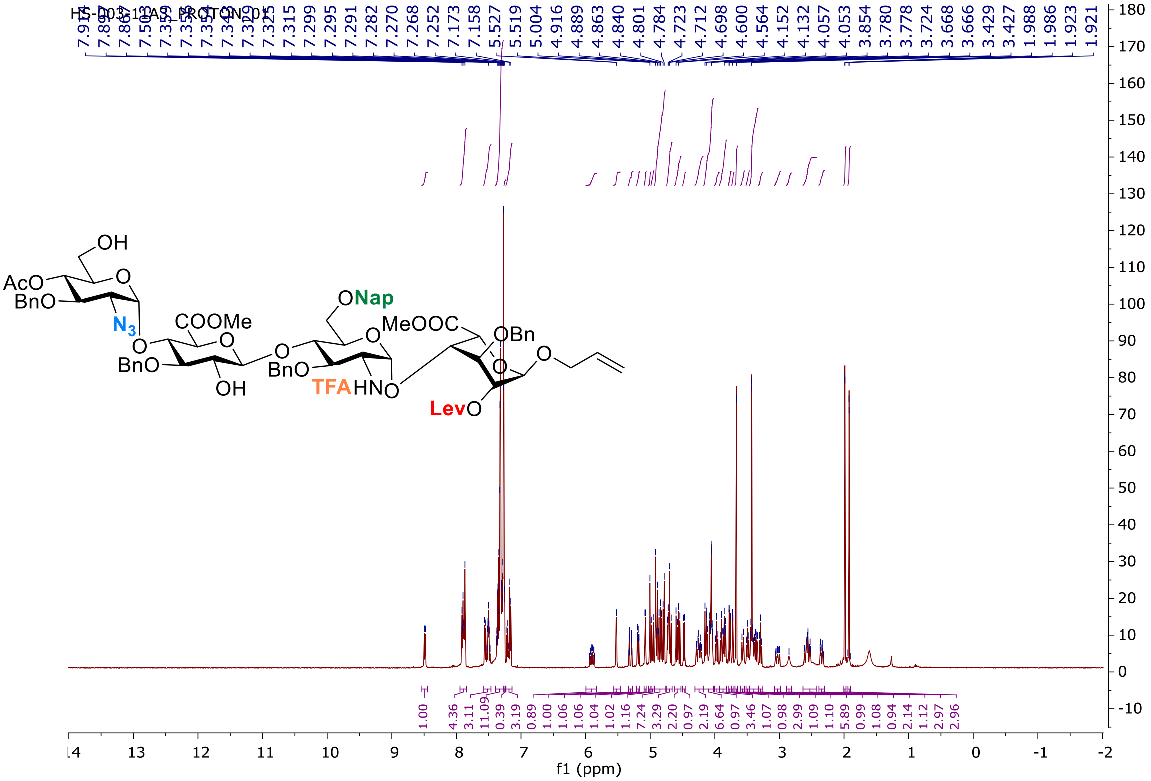

^{1}H NMR spectrum of compound **S12**

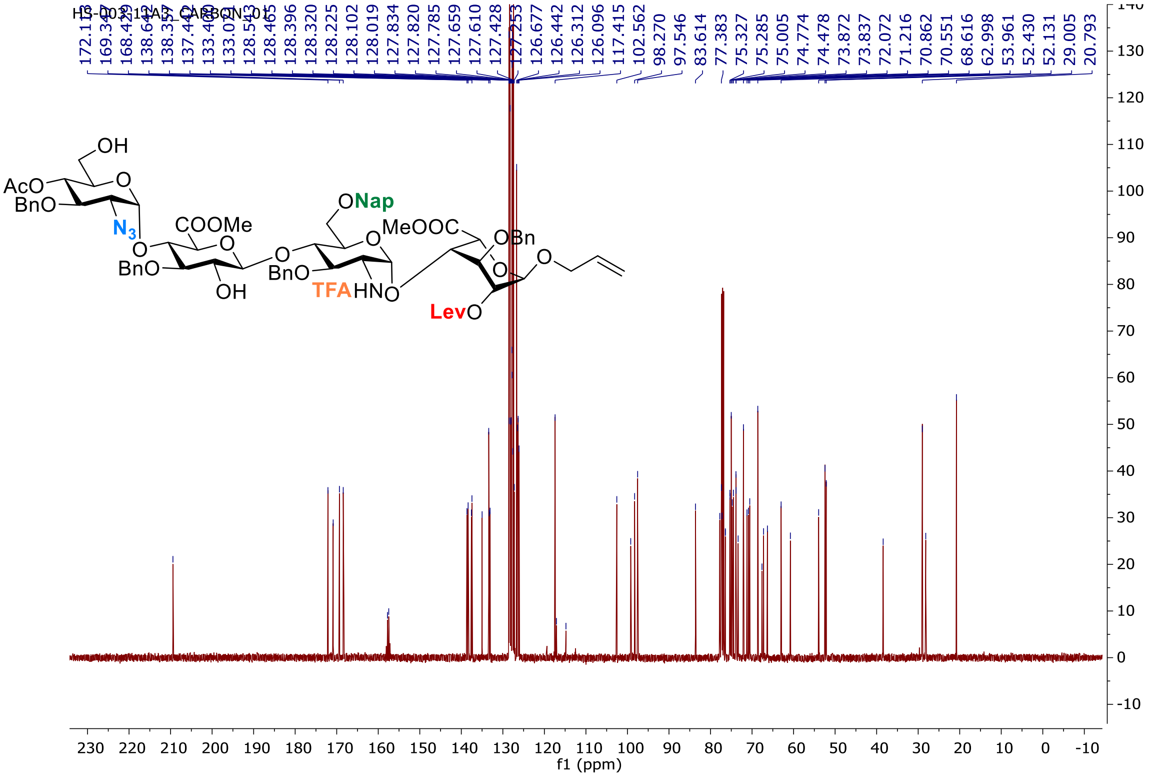

^{13}C NMR spectrum of compound **S12**

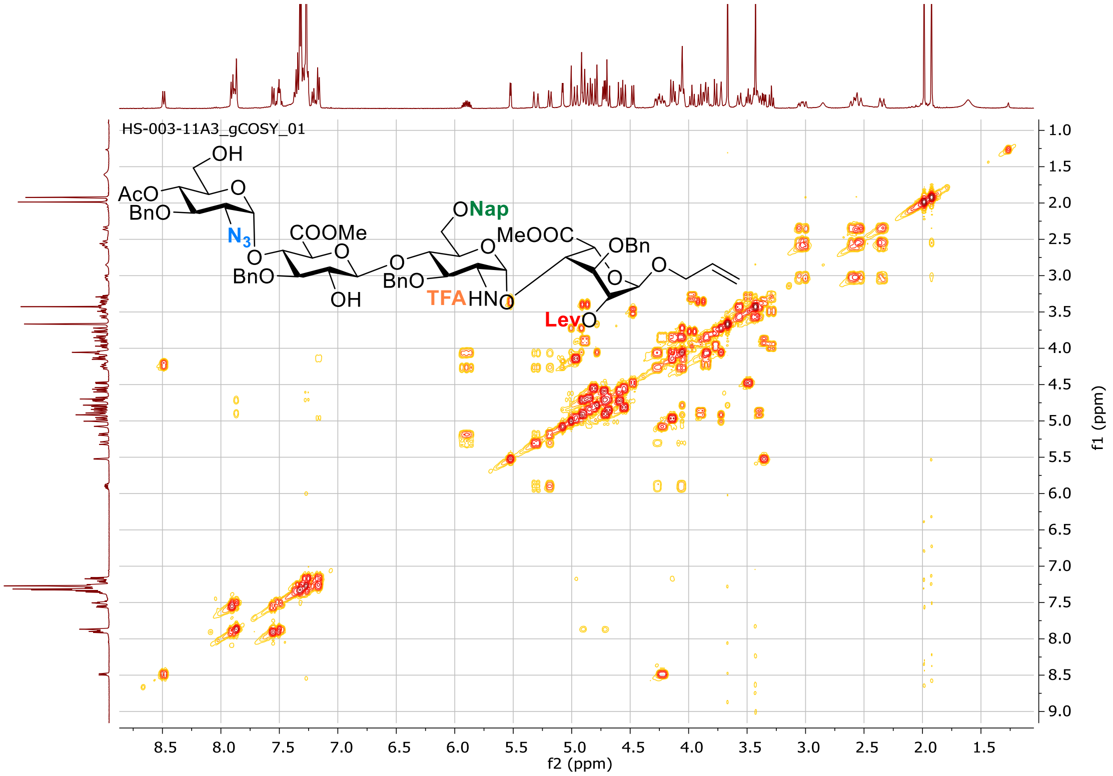

gCOSY NMR spectrum of compound **S12**

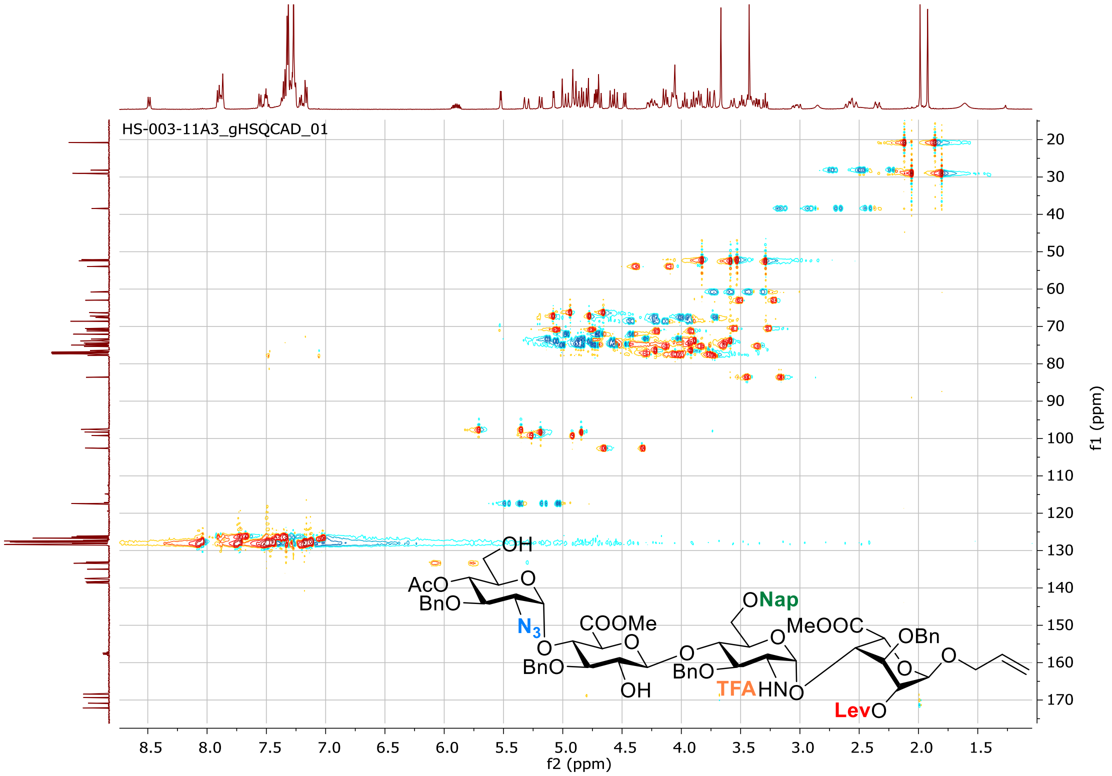

^{1}H-Coupled gHSQC NMR spectrum of compound **S12**

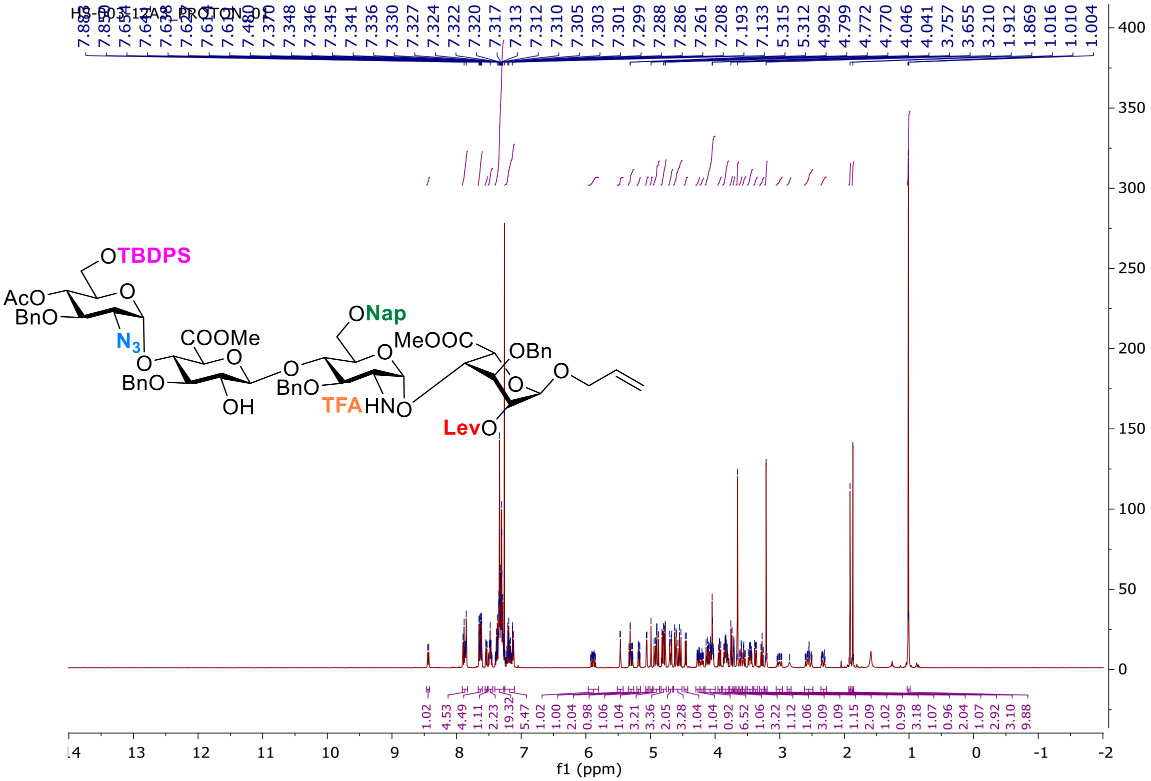

^{1}H NMR spectrum of compound **S13**

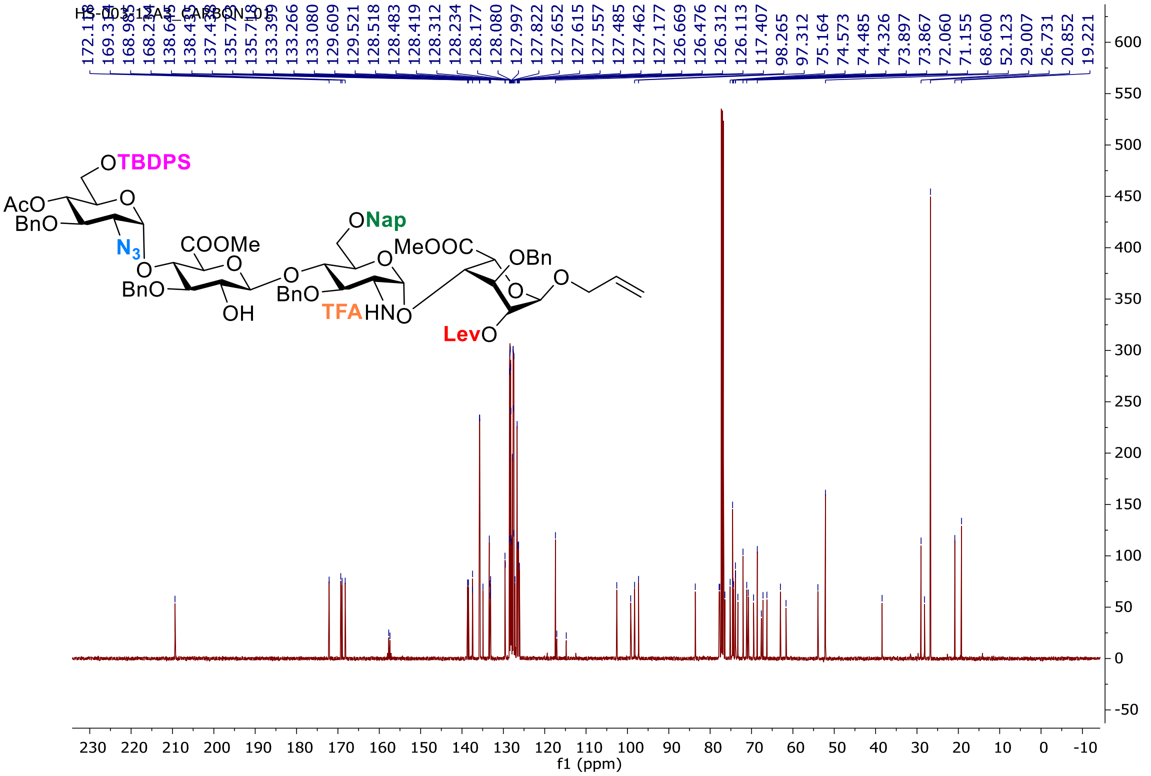

^{13}C NMR spectrum of compound **S13**

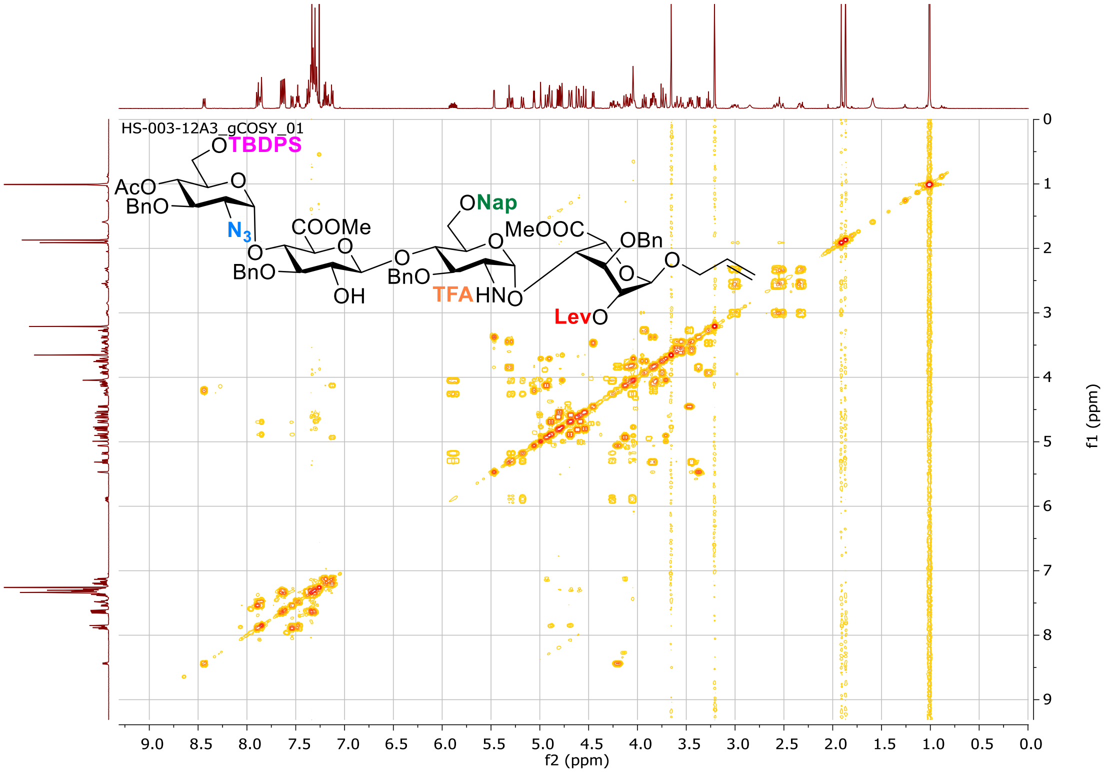

gCOSY NMR spectrum of compound **S13**

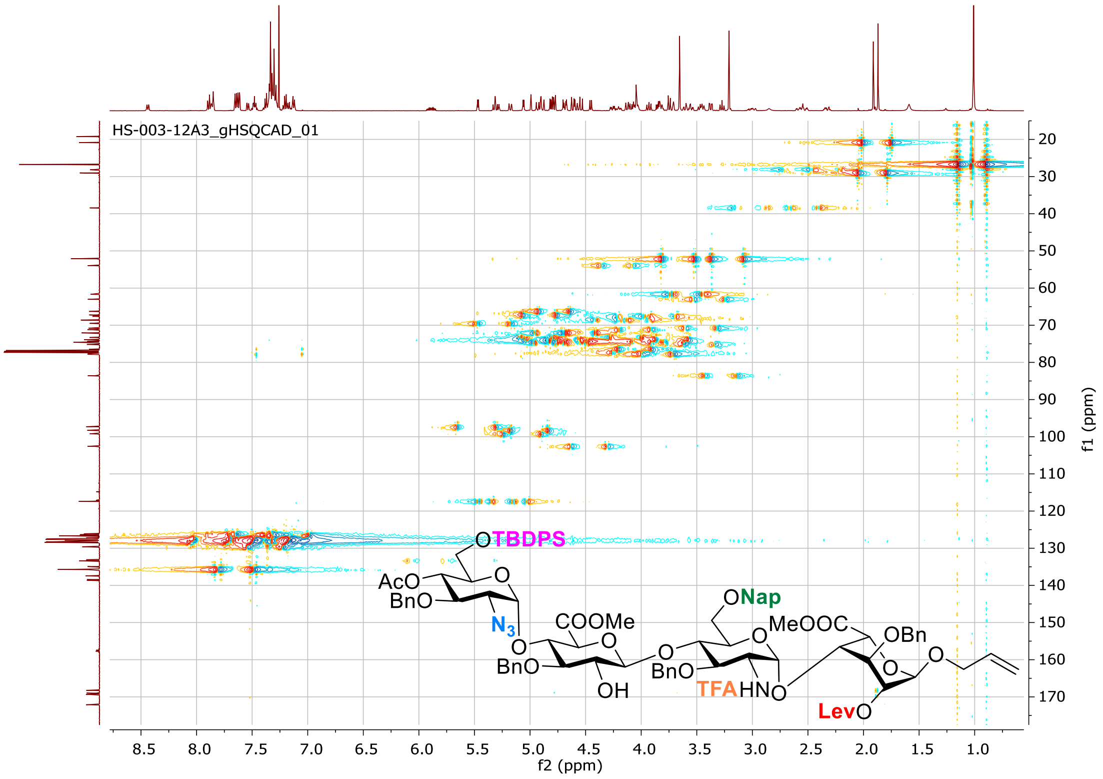

^{1}H-Coupled gHSQC NMR spectrum of compound **S13**

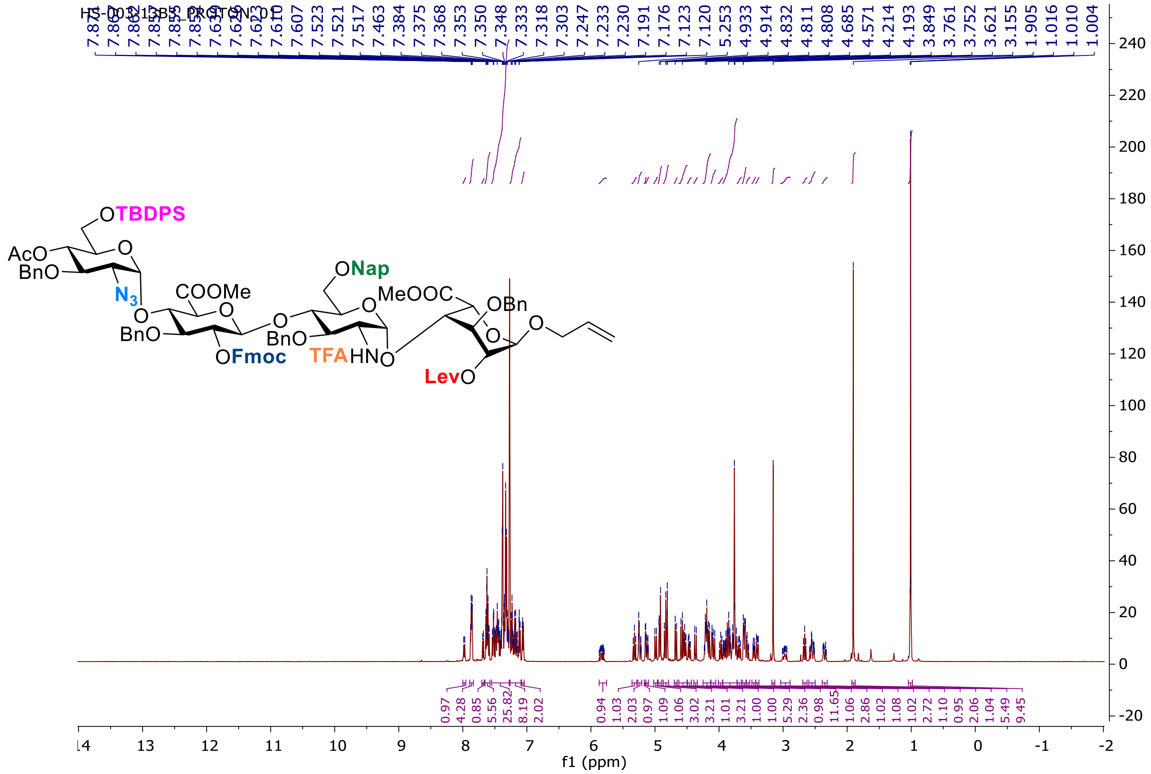

^{1}H NMR spectrum of compound **22**

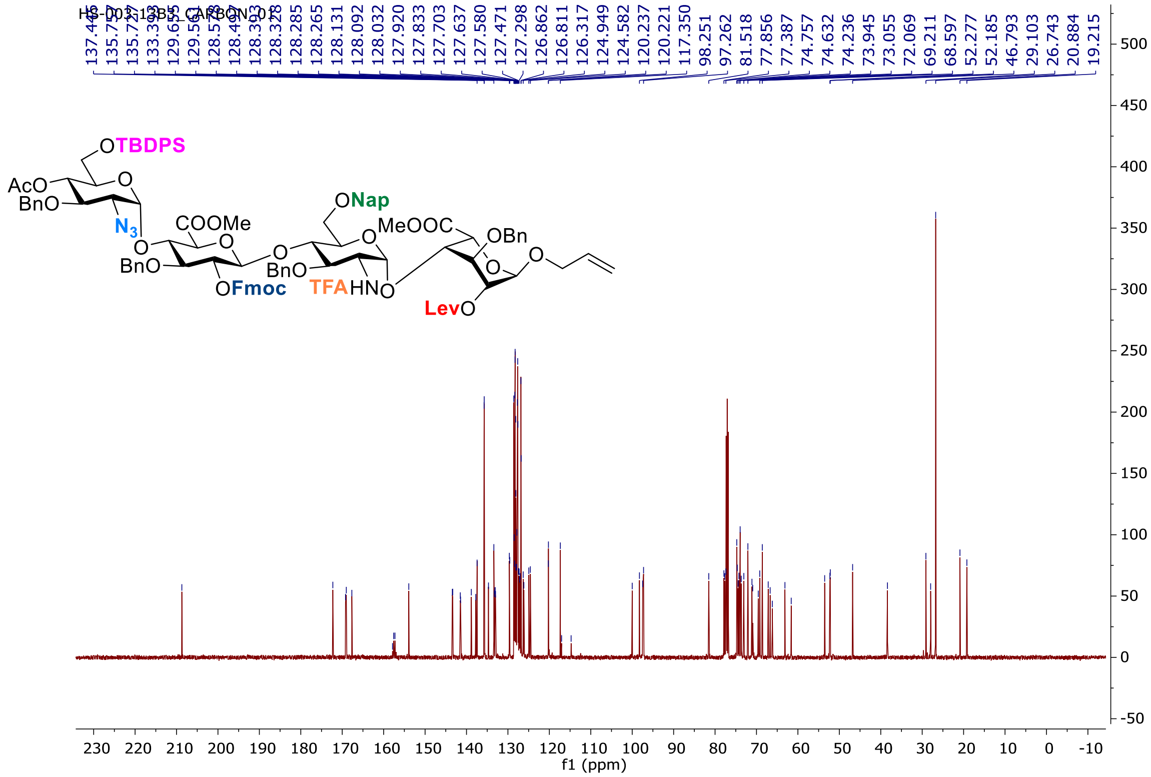

^{13}C NMR spectrum of compound **22**

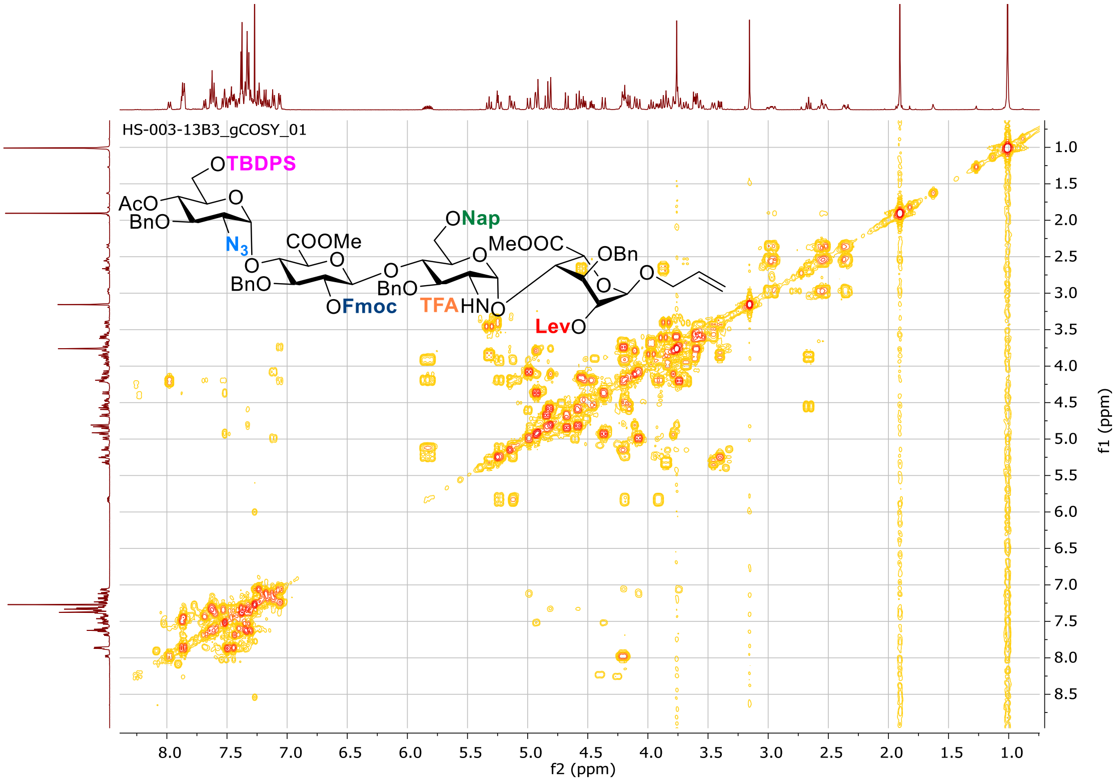

gCOSY NMR spectrum of compound **22**

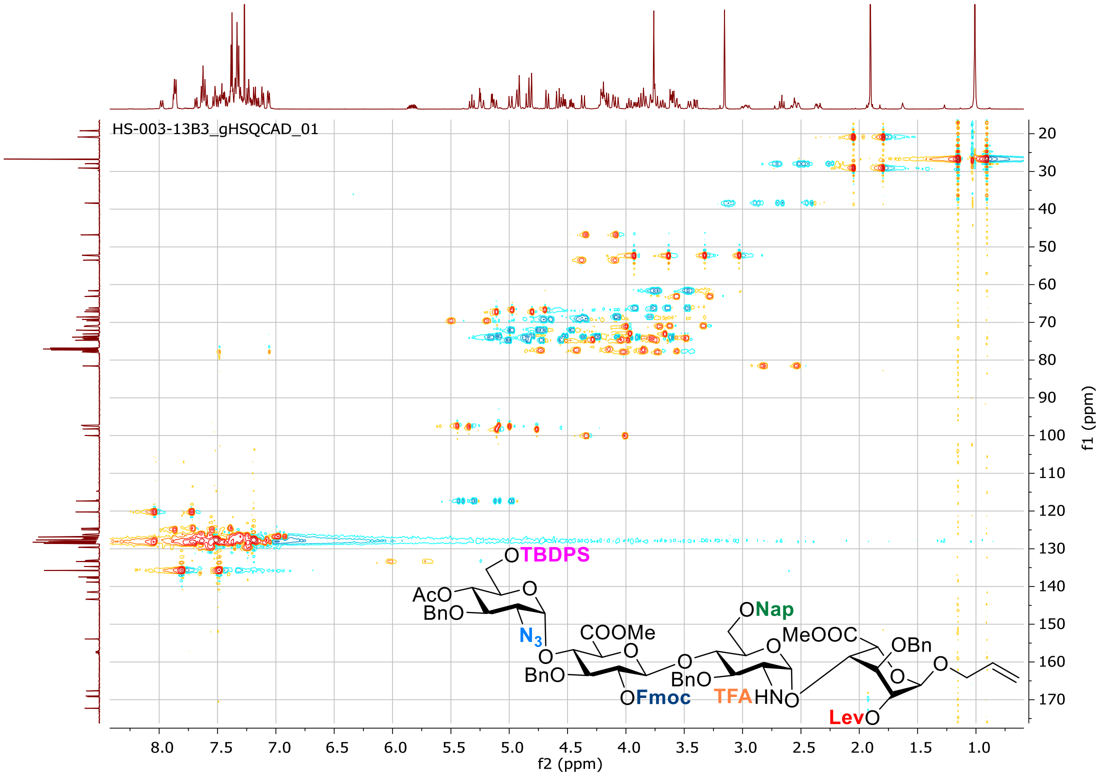

^{1}H-Coupled gHSQC NMR spectrum of compound **22**

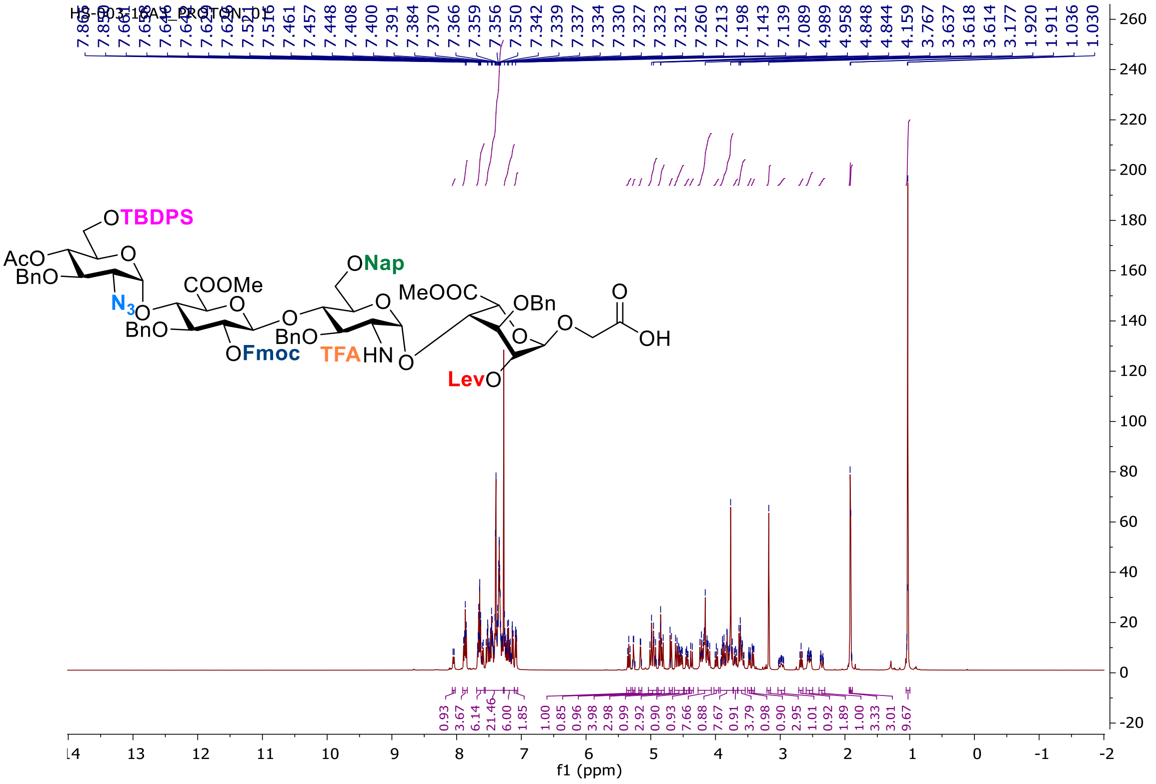

^{1}H NMR spectrum of compound **23**

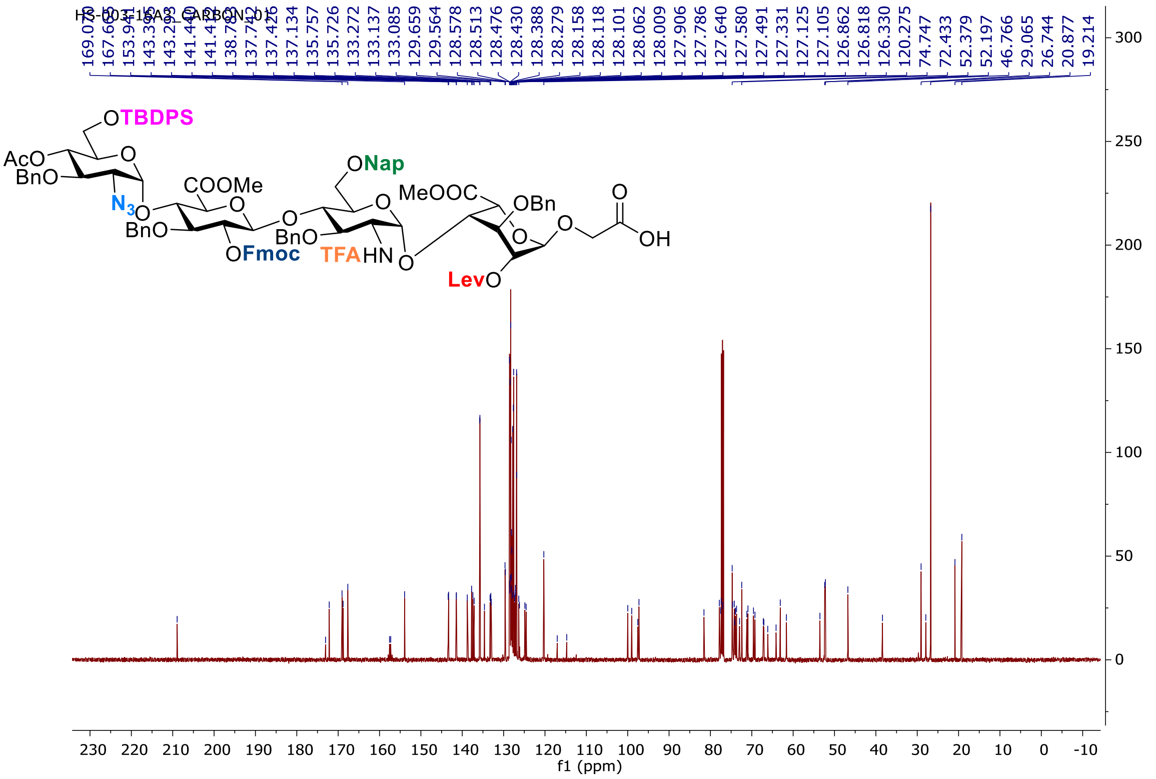

^{13}C NMR spectrum of compound **23**

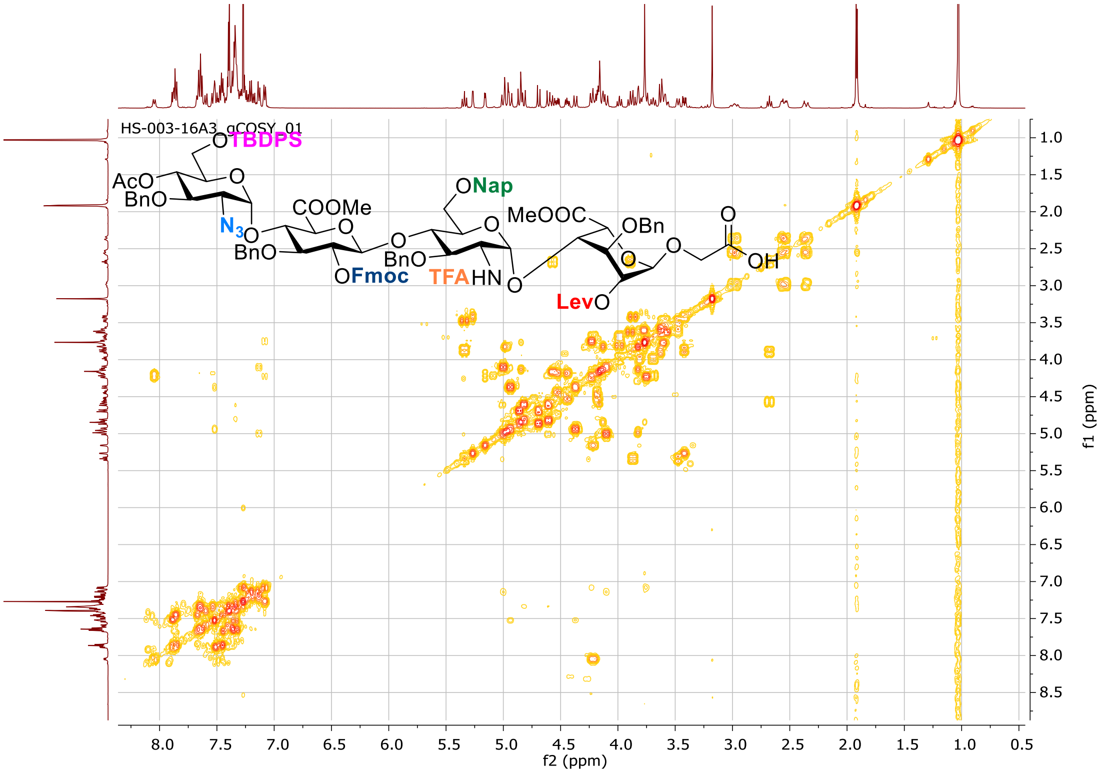

gCOSY NMR spectrum of compound **23**

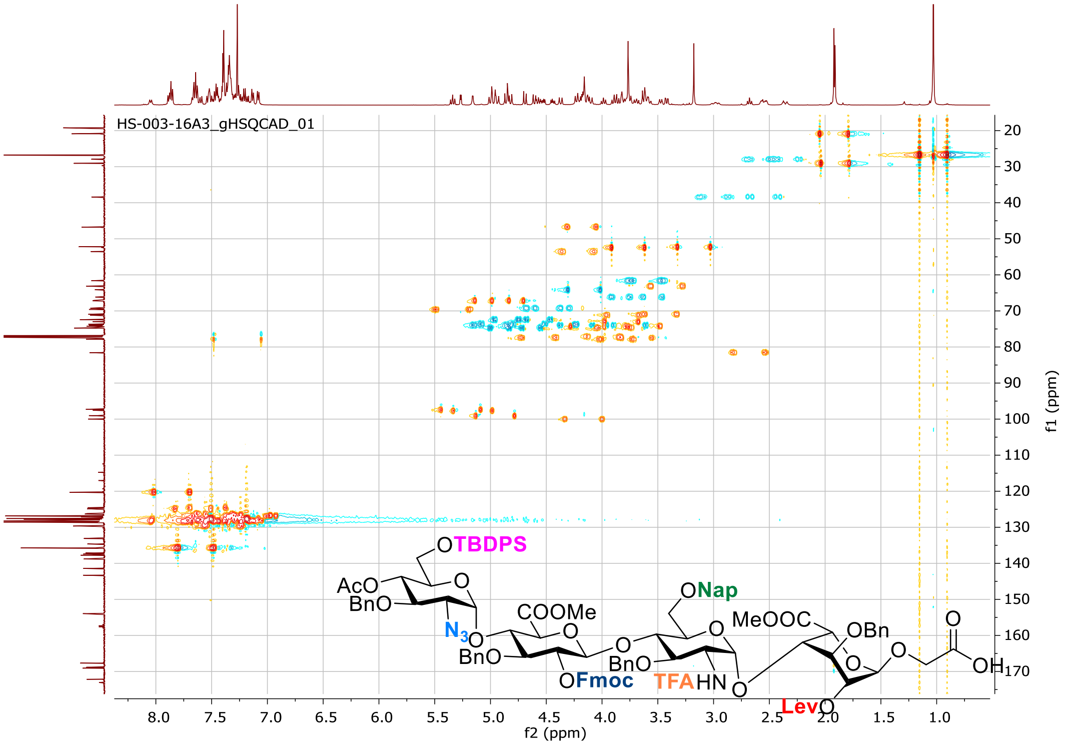

^{1}H-Coupled gHSQC NMR spectrum of compound **23**

NMR of the library of tetrasaccharides **40-103**

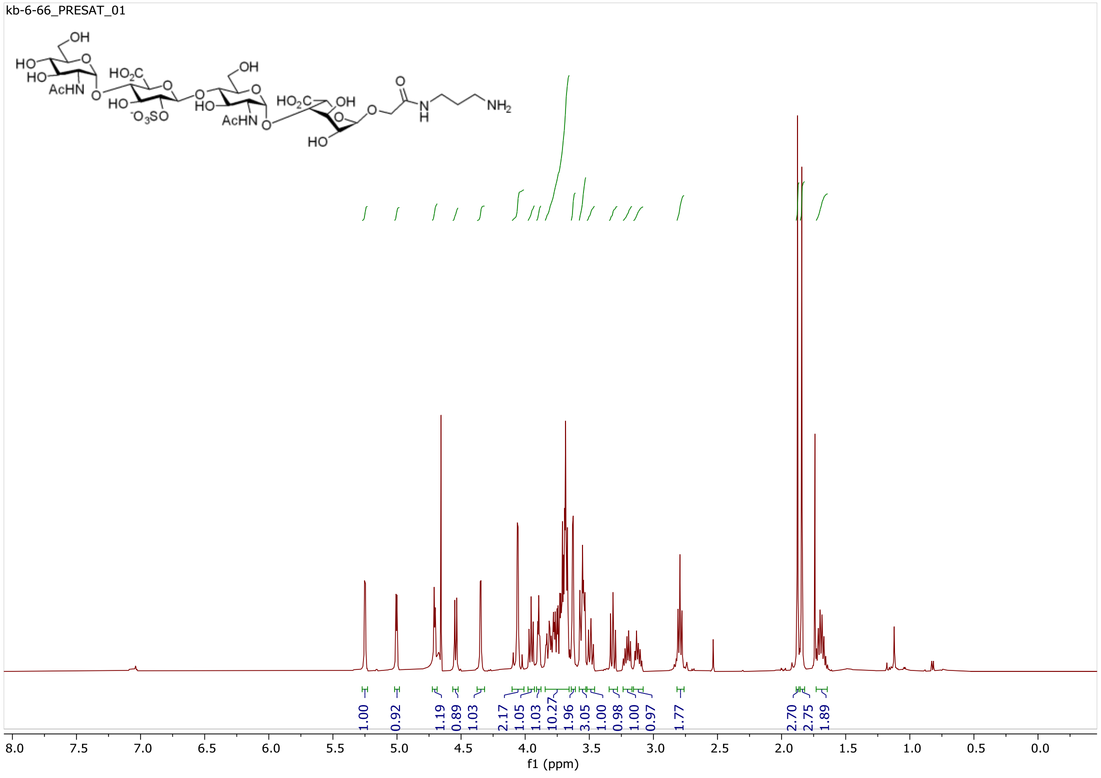

^{1}HNMR spectrum of compound **40**

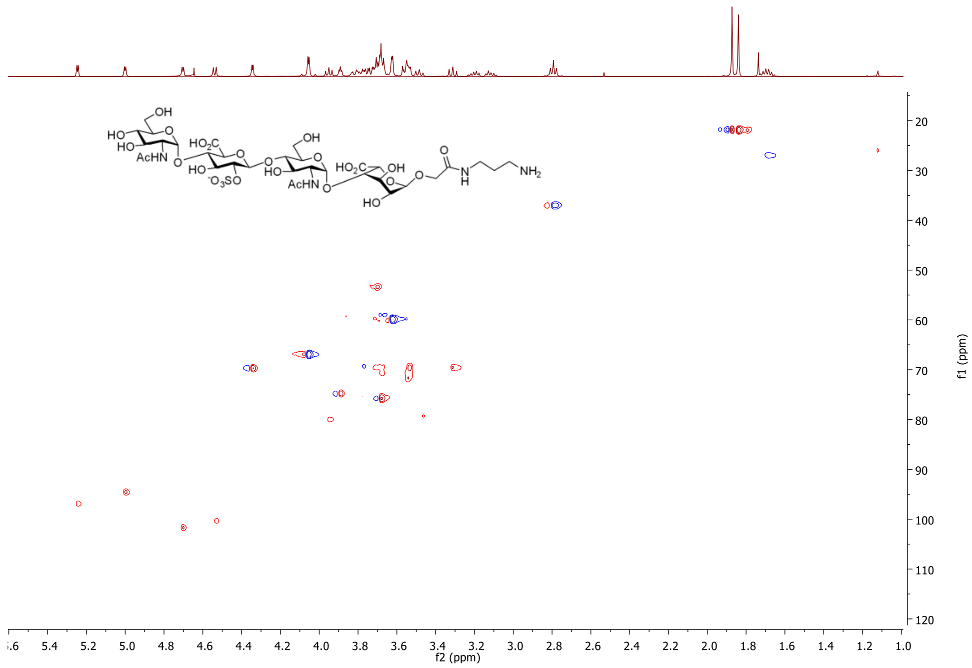

^{1}H-decoupled HSQC of compound **40**

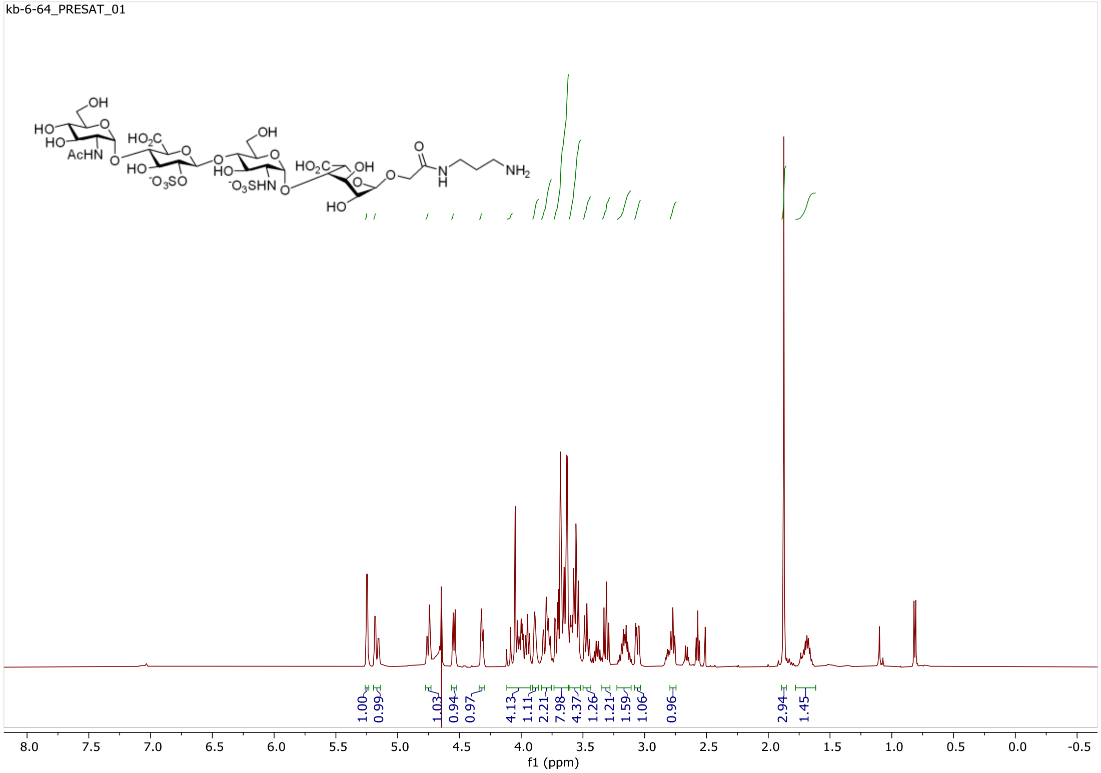

^{1}HNMR spectrum of compound **41**

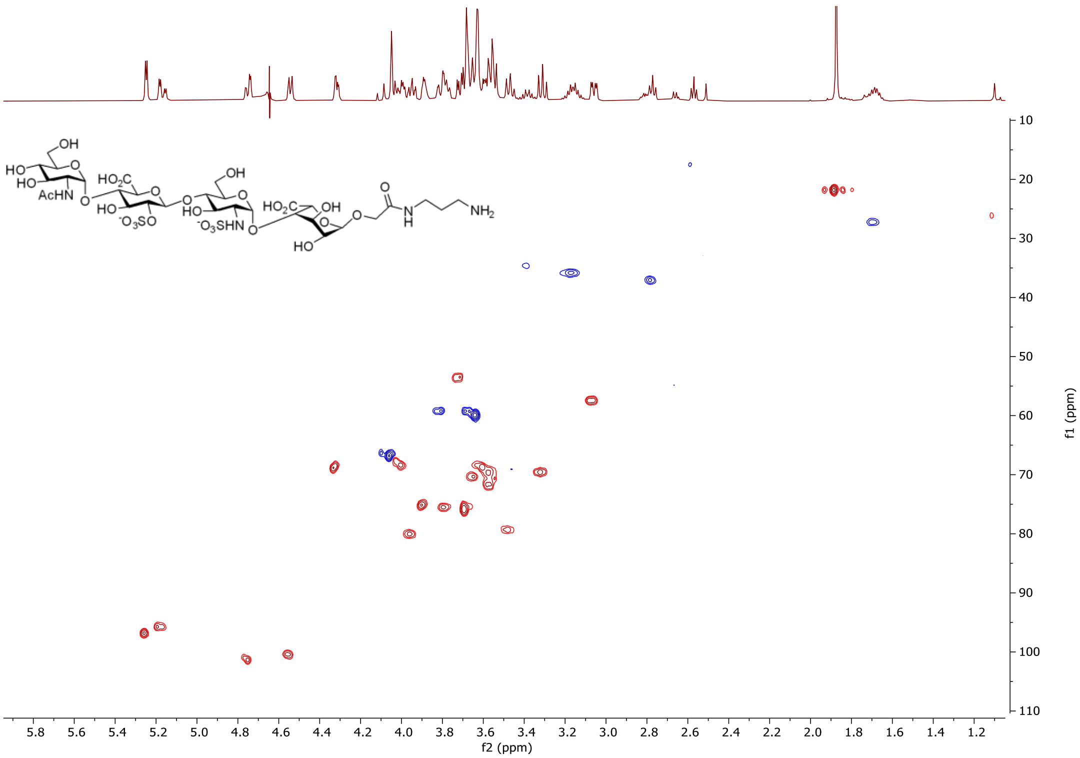

^{1}H-decoupled HSQC of compound **41**

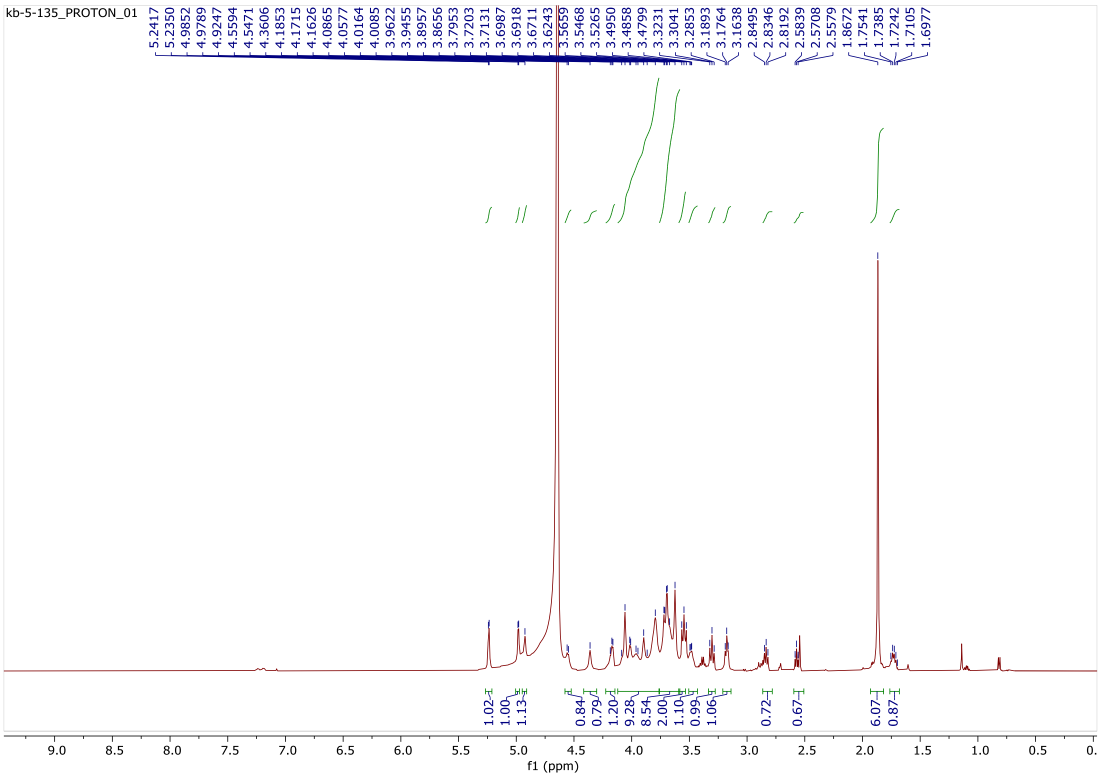

^{1}HNMR spectrum of compound **42**

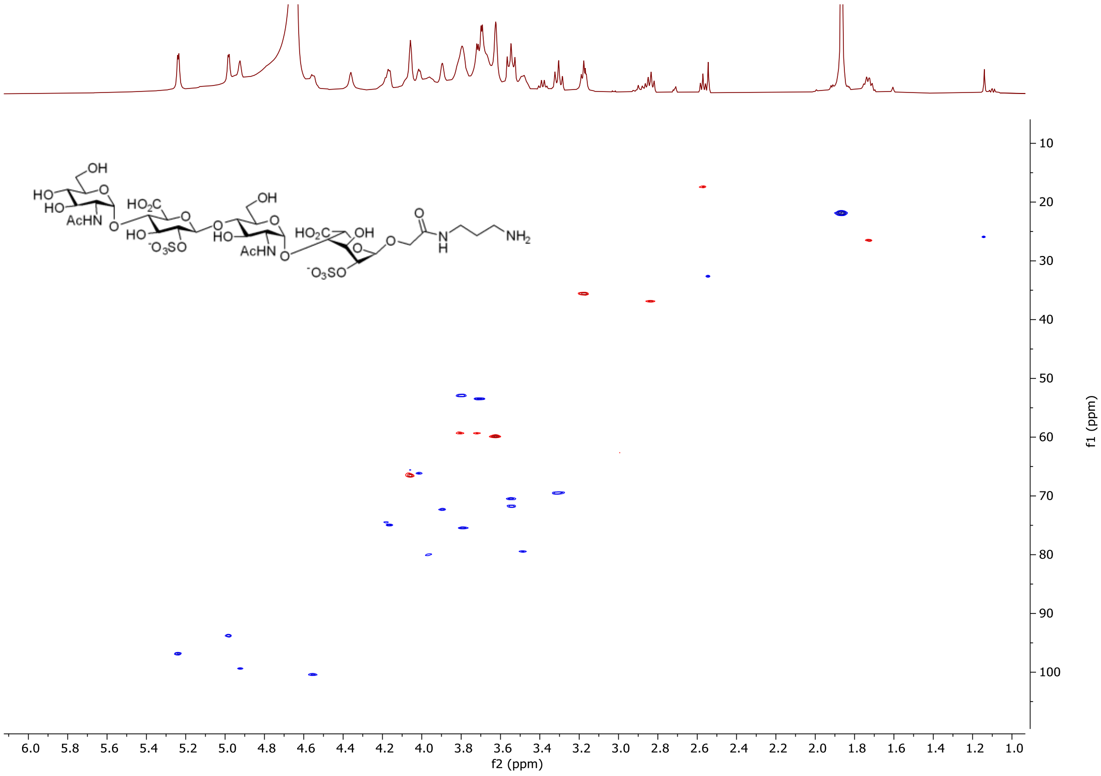

^{1}H-decoupled HSQC of compound **42**

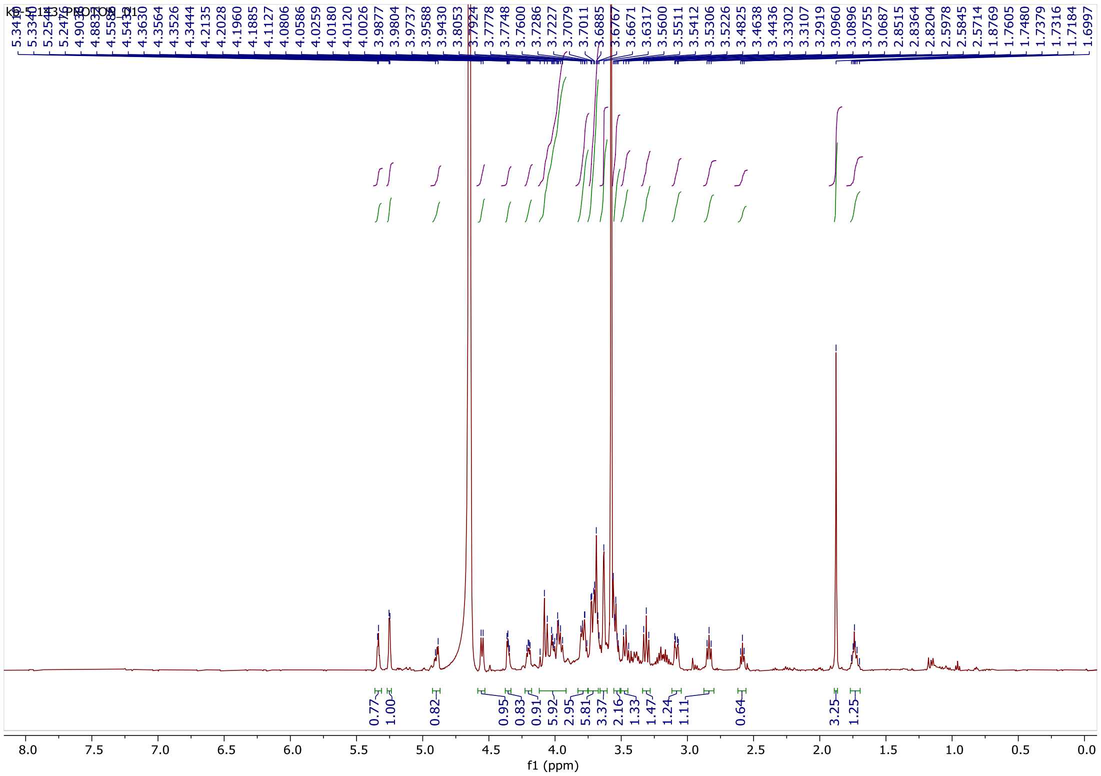

^{1}HNMR spectrum of compound **43**

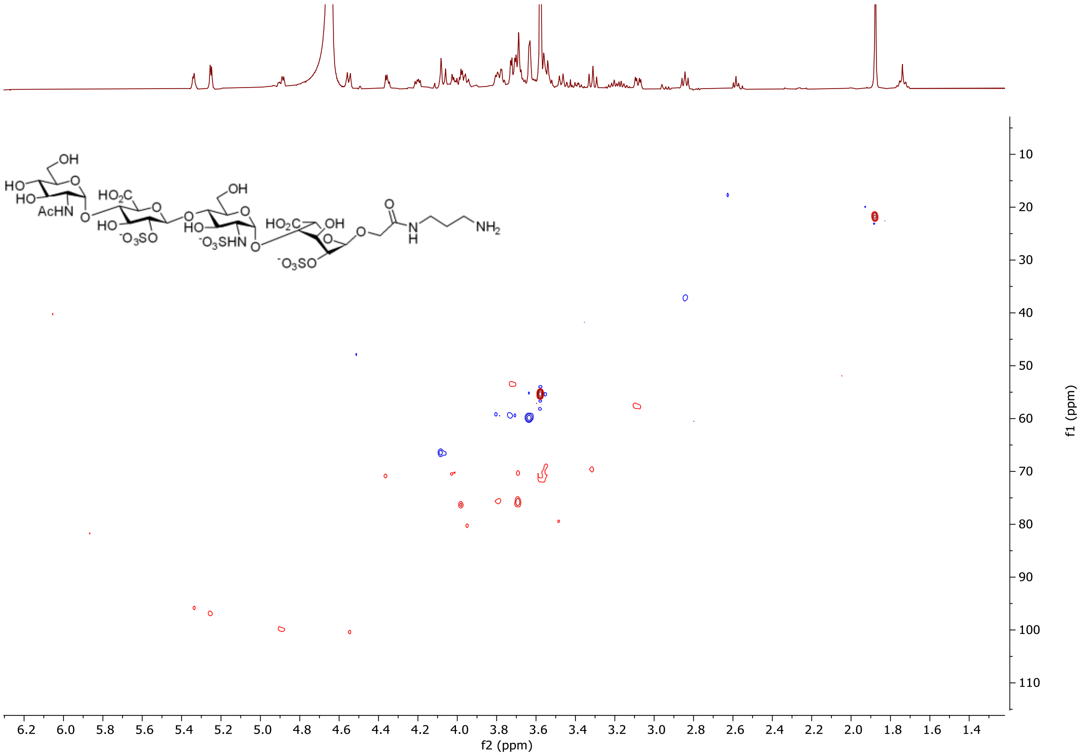

^{1}H-decoupled HSQC of compound **43**

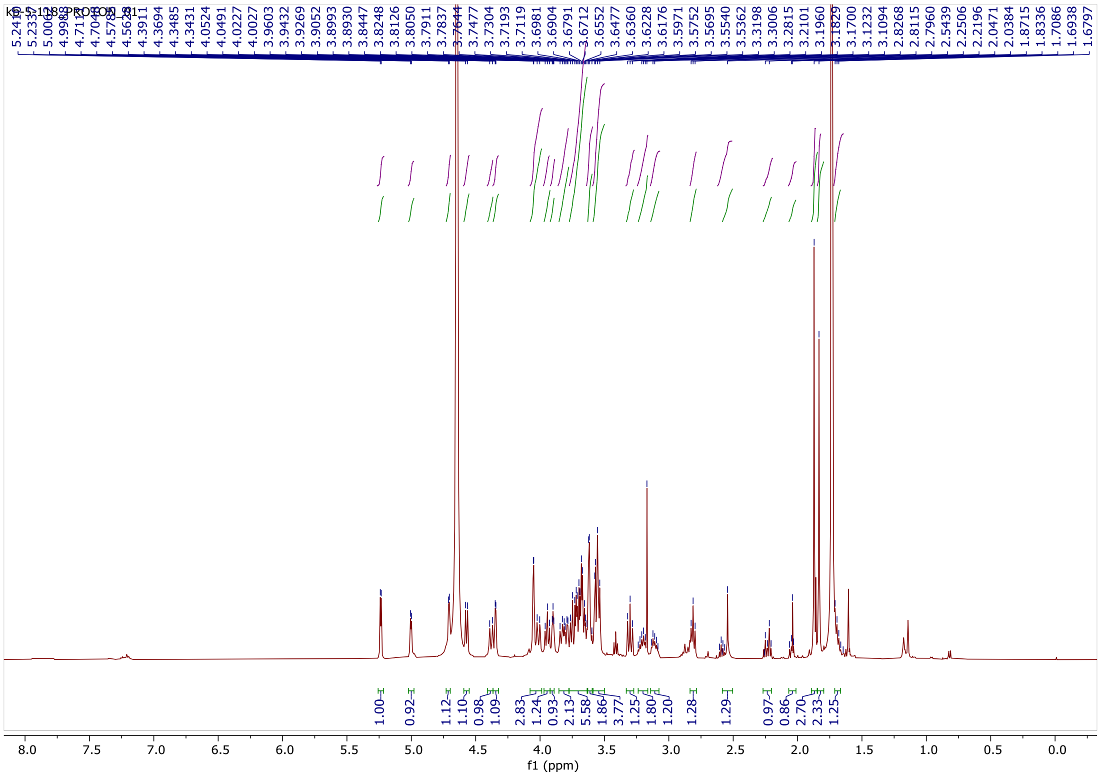

^{1}HNMR spectrum of compound **44**

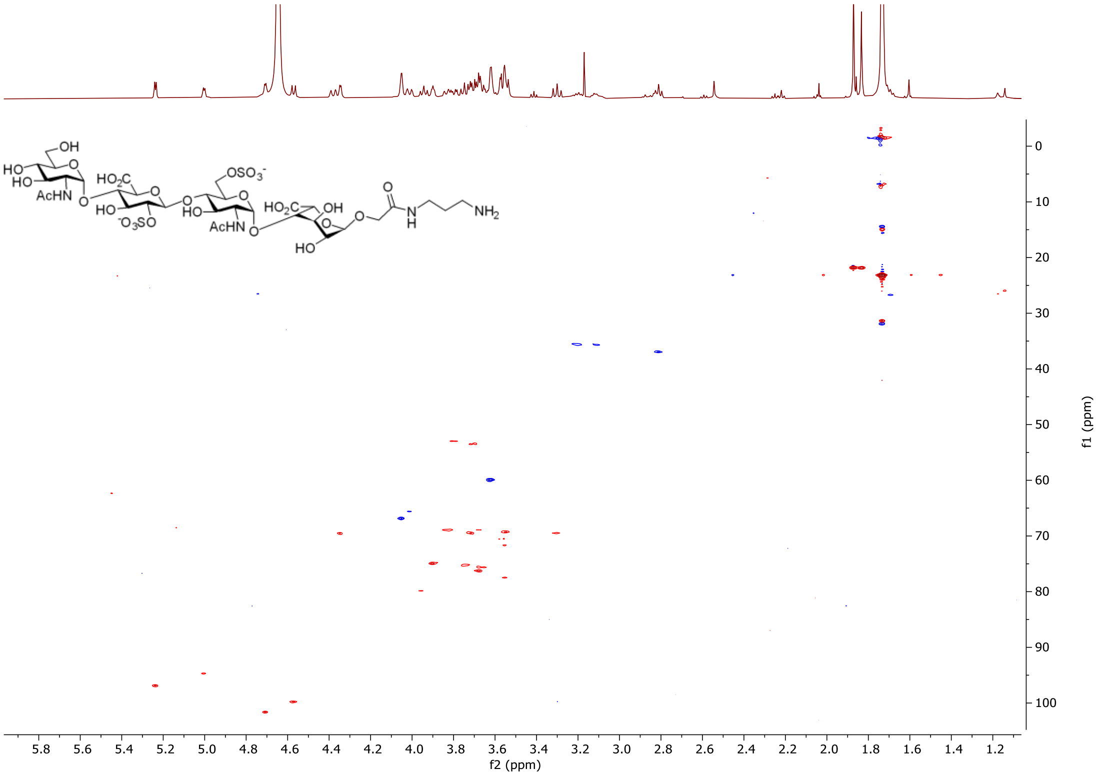

^{1}H-decoupled HSQC of compound **44**

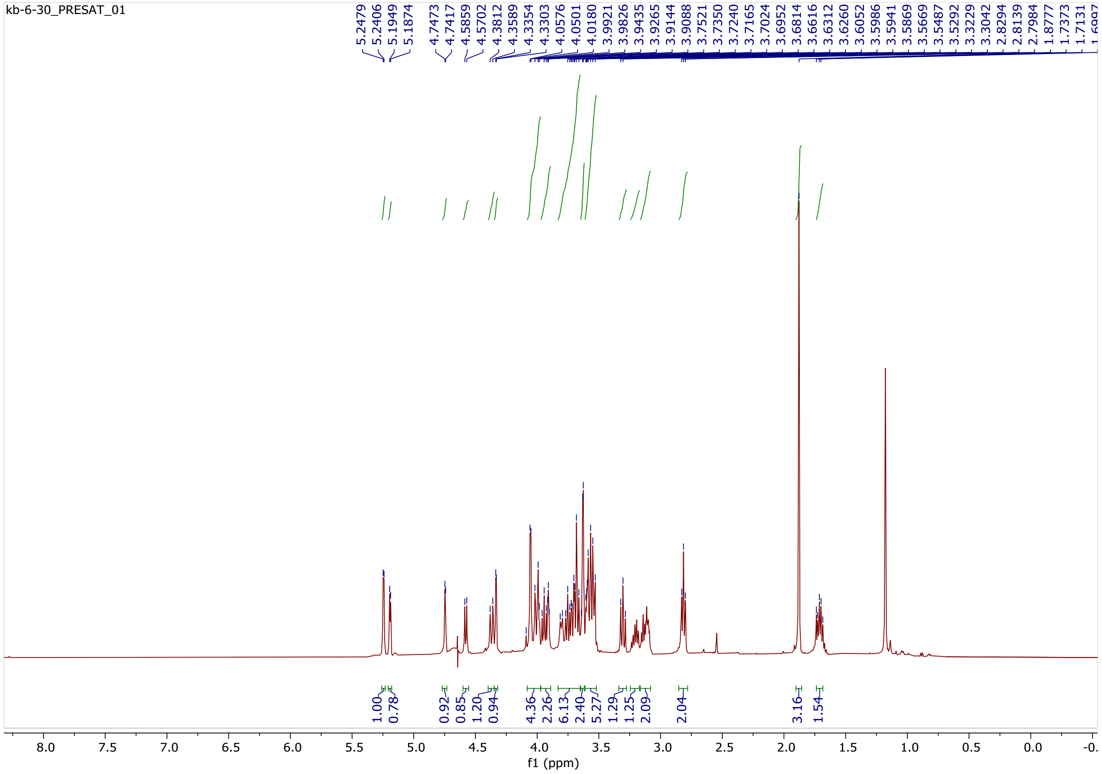

^{1}HNMR spectrum of compound **45**

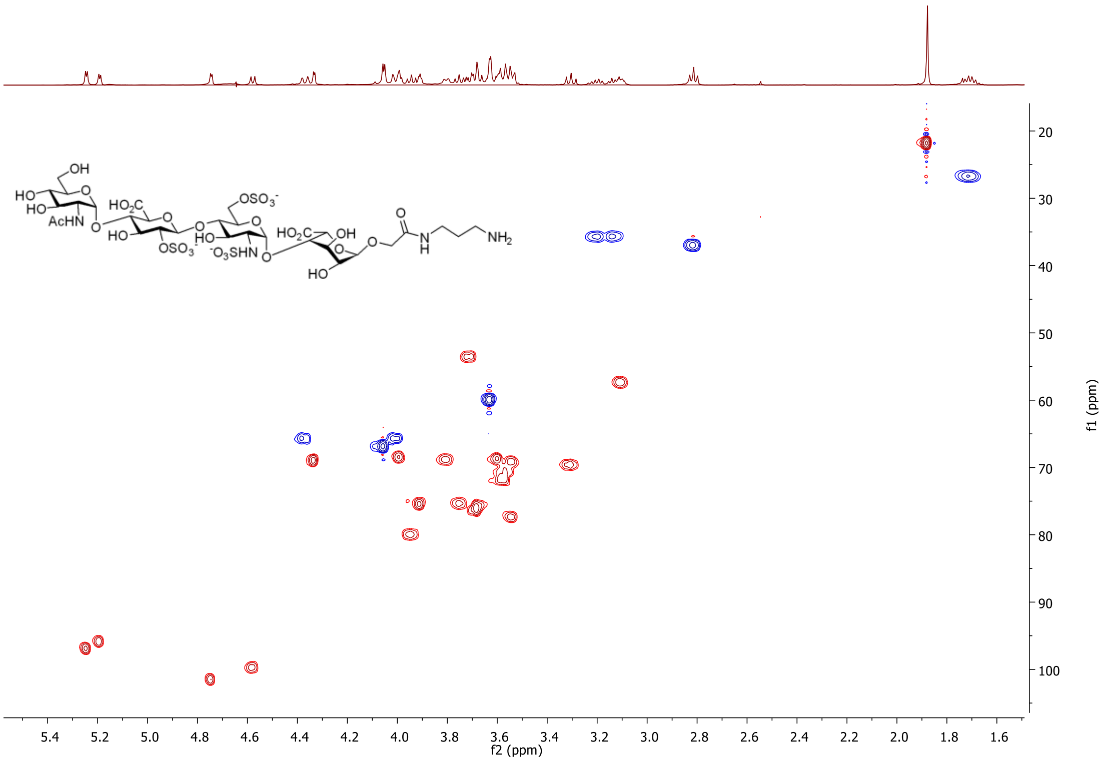

^{1}H-decoupled HSQC of compound **45**

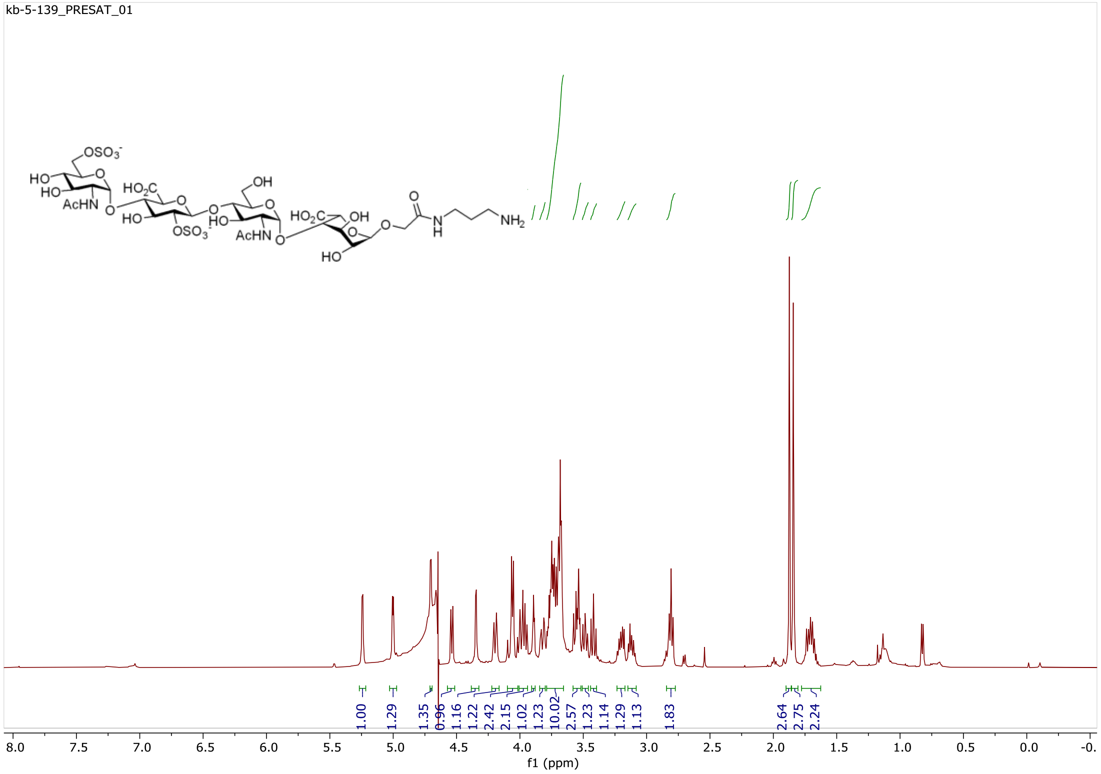

^{1}HNMR spectrum of compound **46**

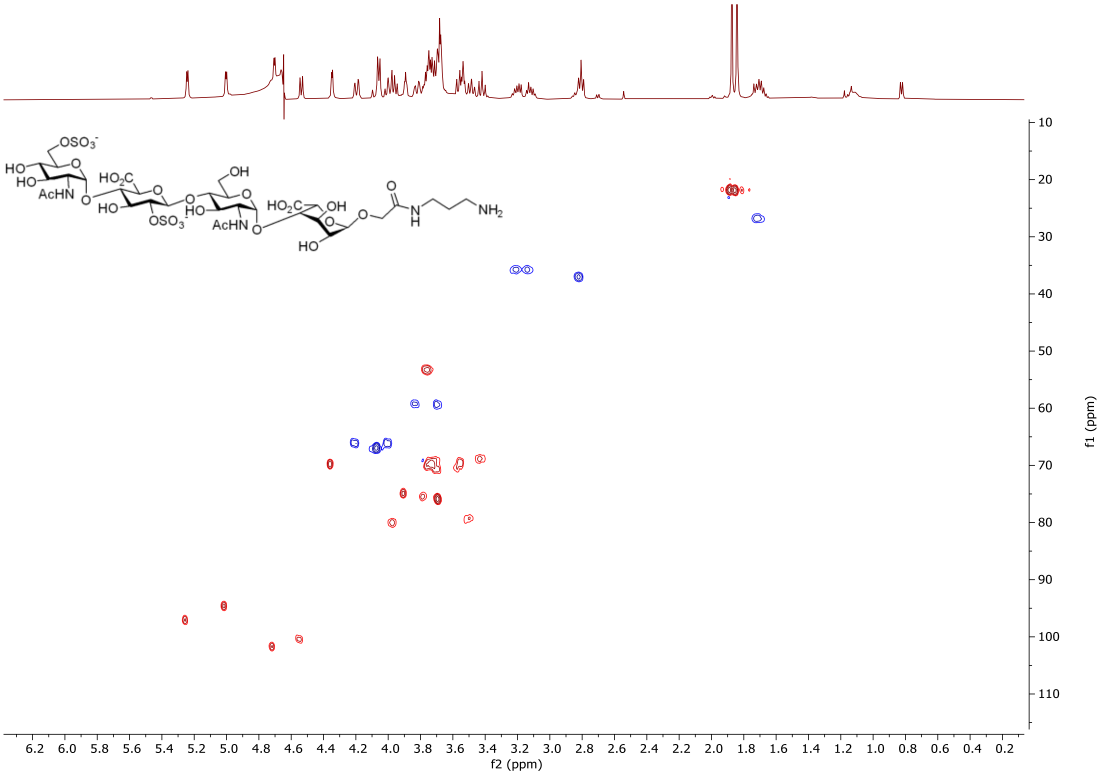

^{1}H-decoupled HSQC of compound **46**

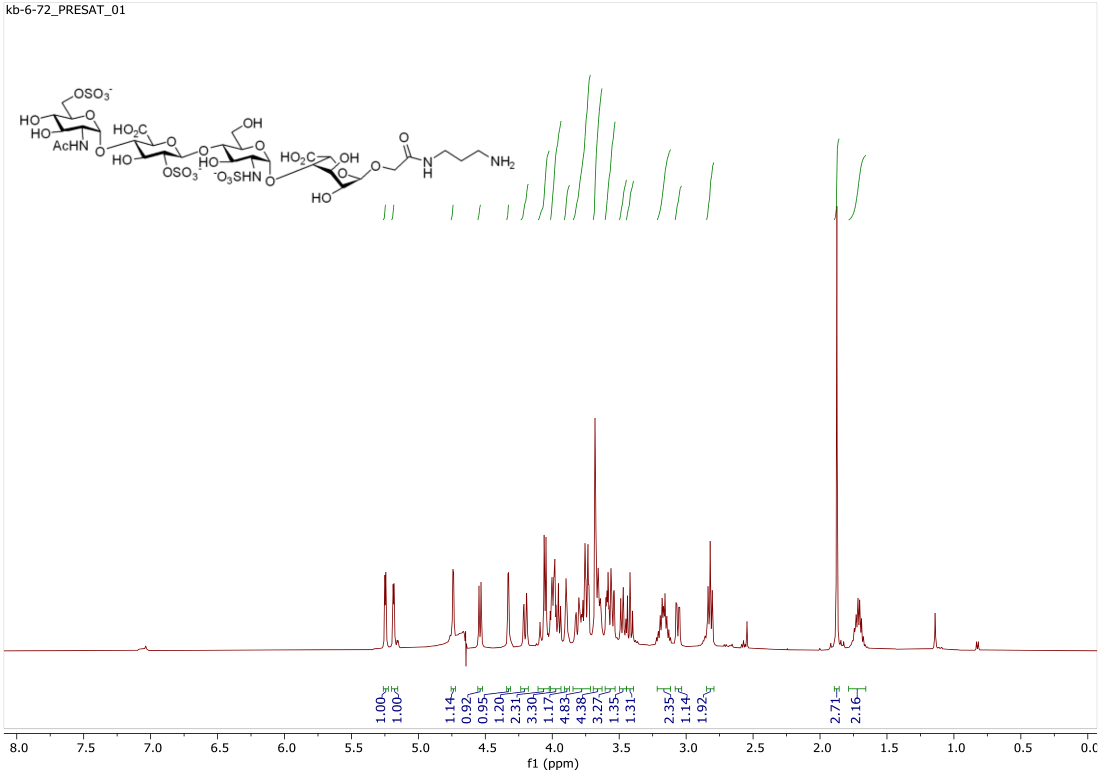

^{1}HNMR spectrum of compound **47**

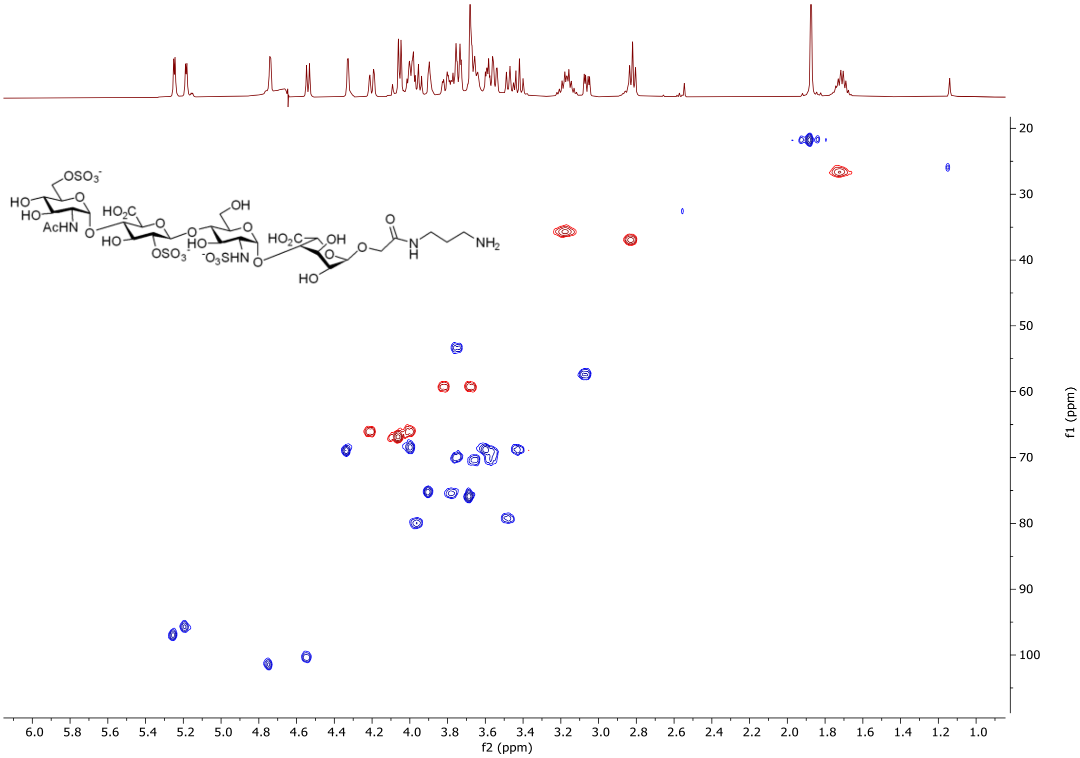

^{1}H-decoupled HSQC of compound **47**

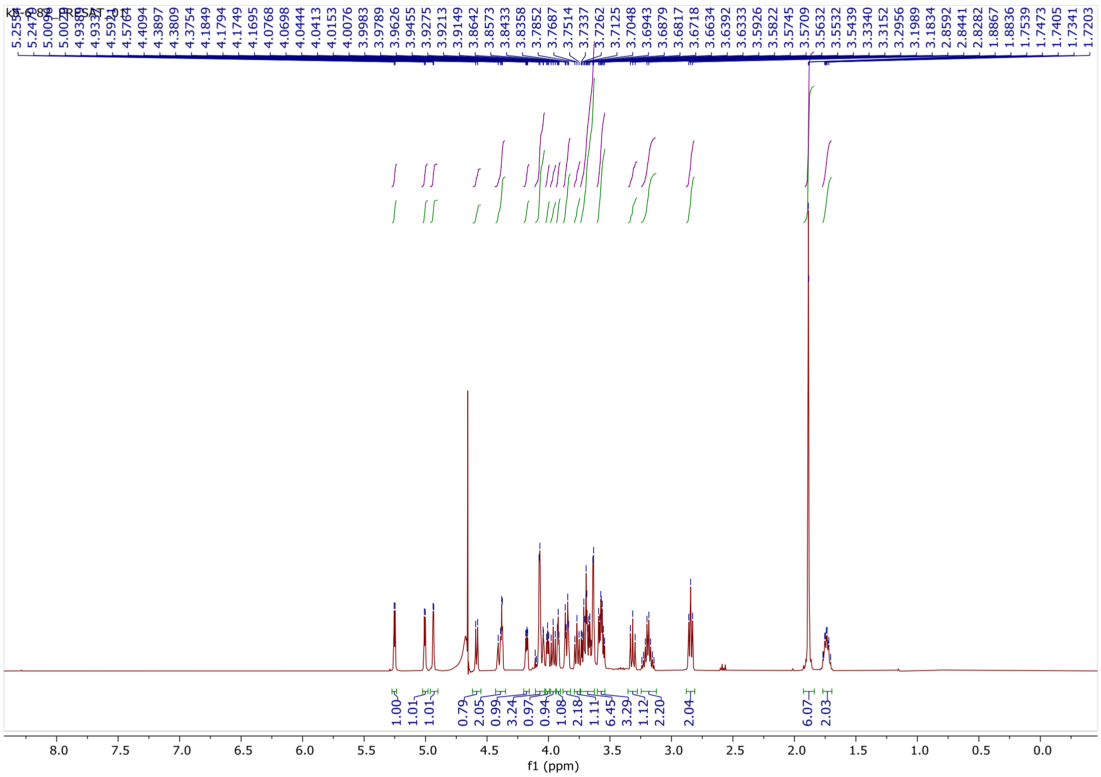

^{1}HNMR spectrum of compound **48**

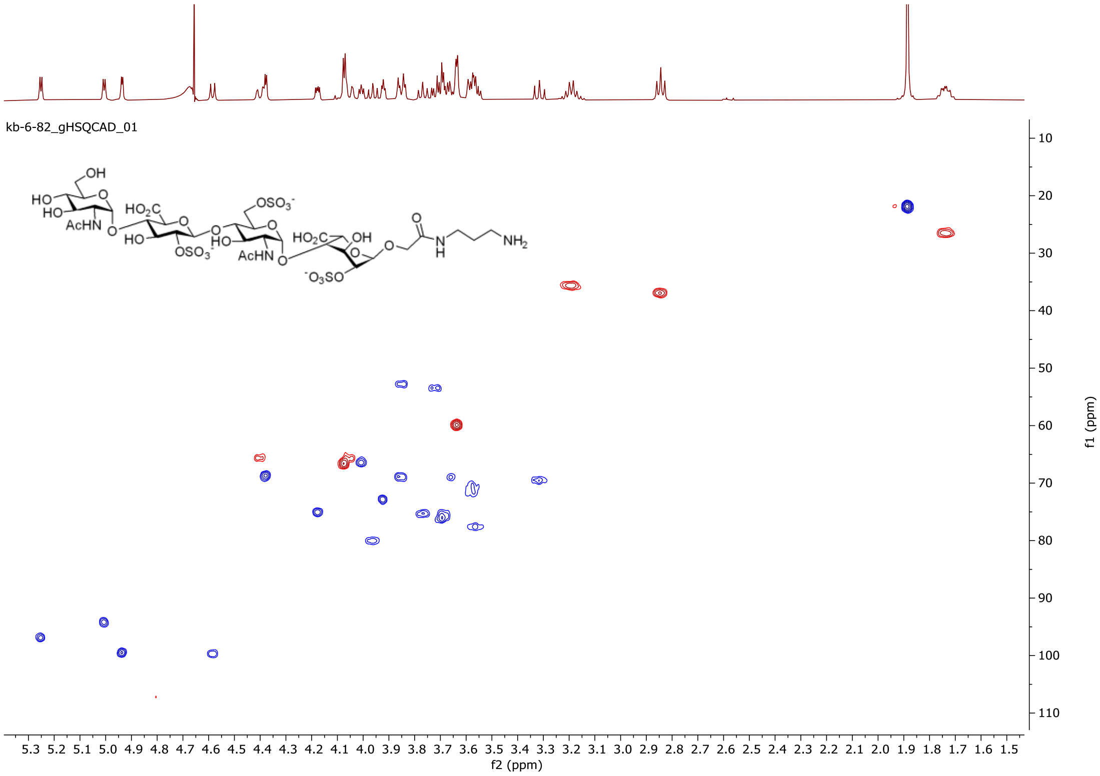

^{1}H-decoupled HSQC of compound **48**

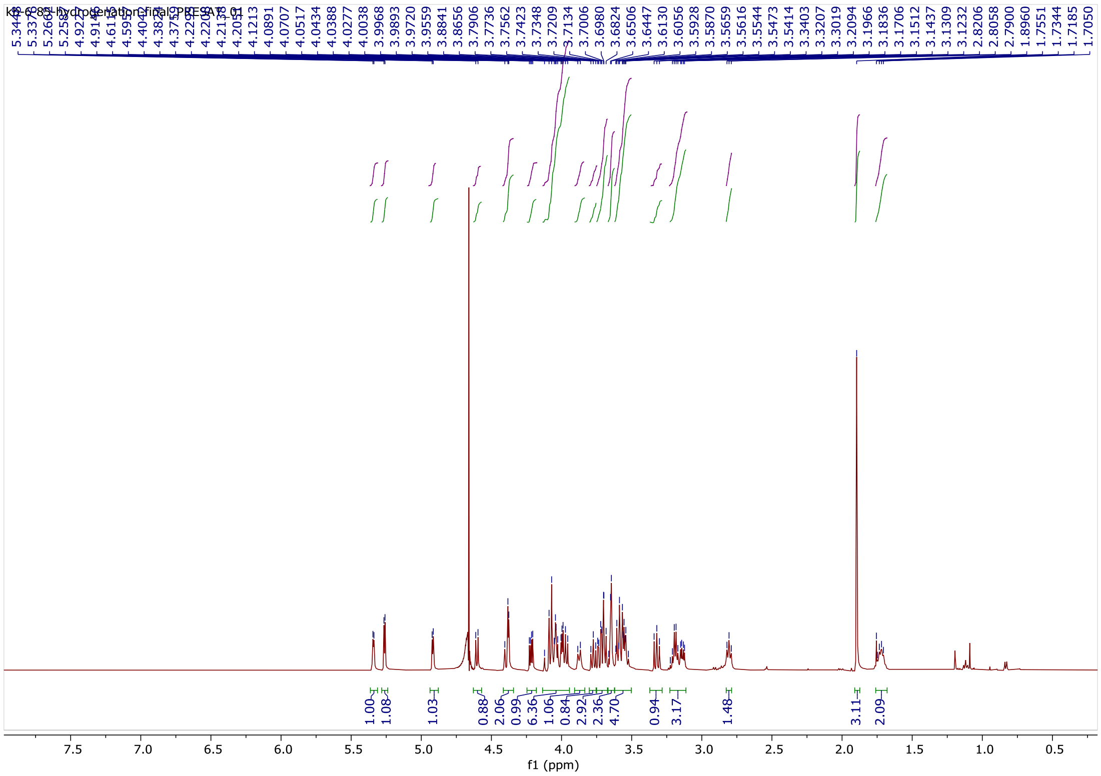

^{1}HNMR spectrum of compound **49**

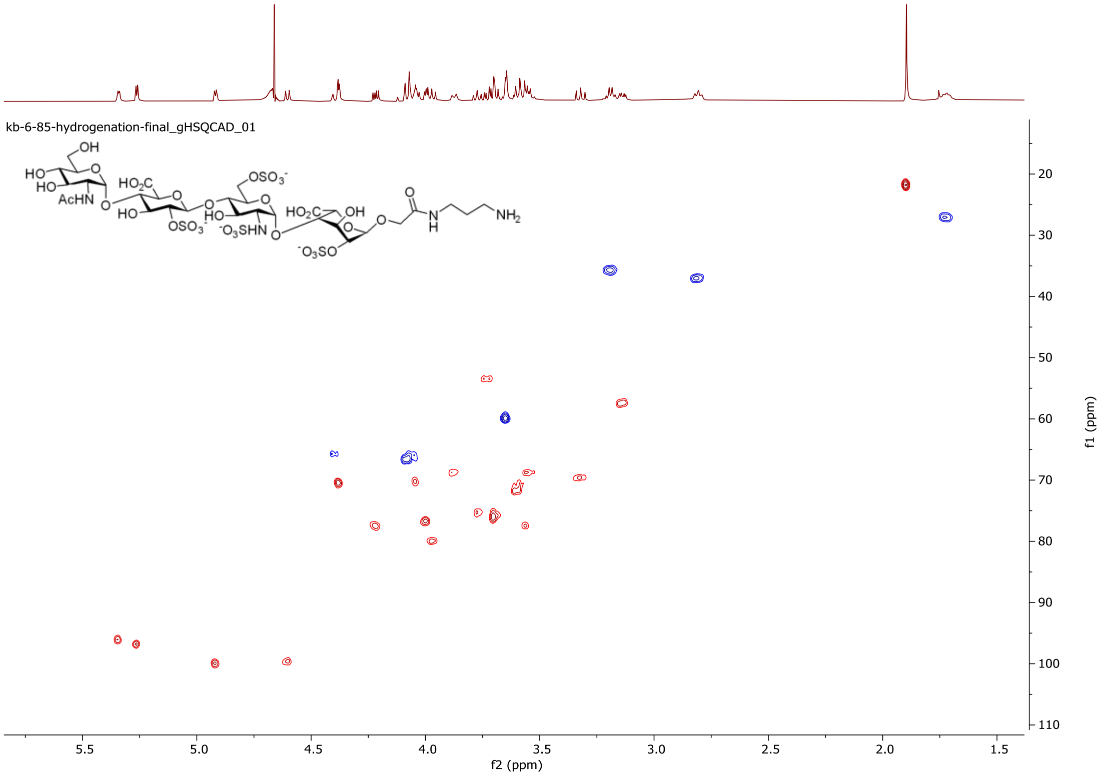

^{1}H-decoupled HSQC of compound **49**

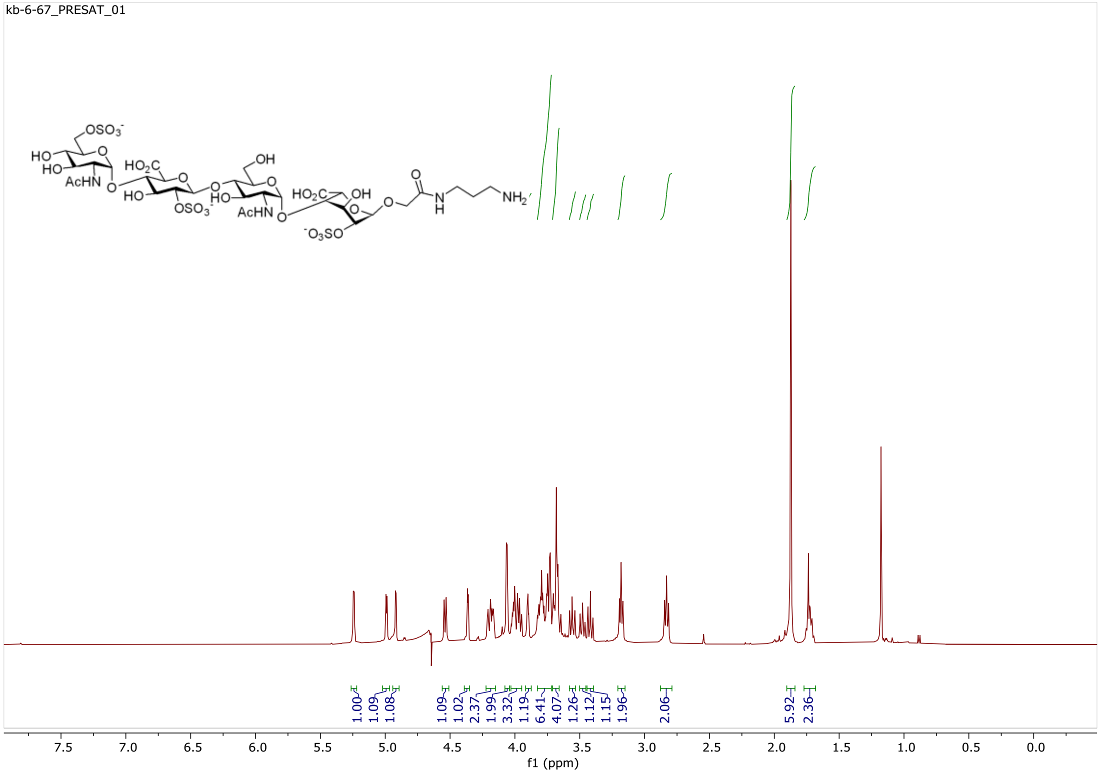

^{1}HNMR spectrum of compound **50**

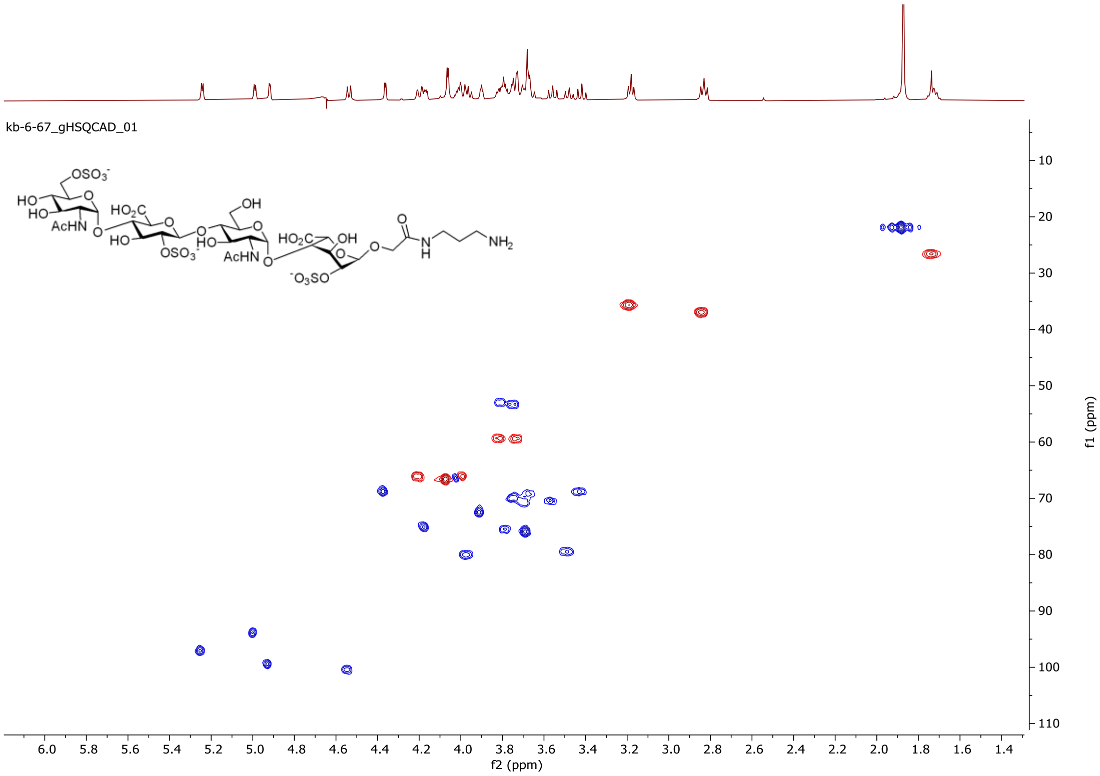

^{1}H-decoupled HSQC of compound **50**

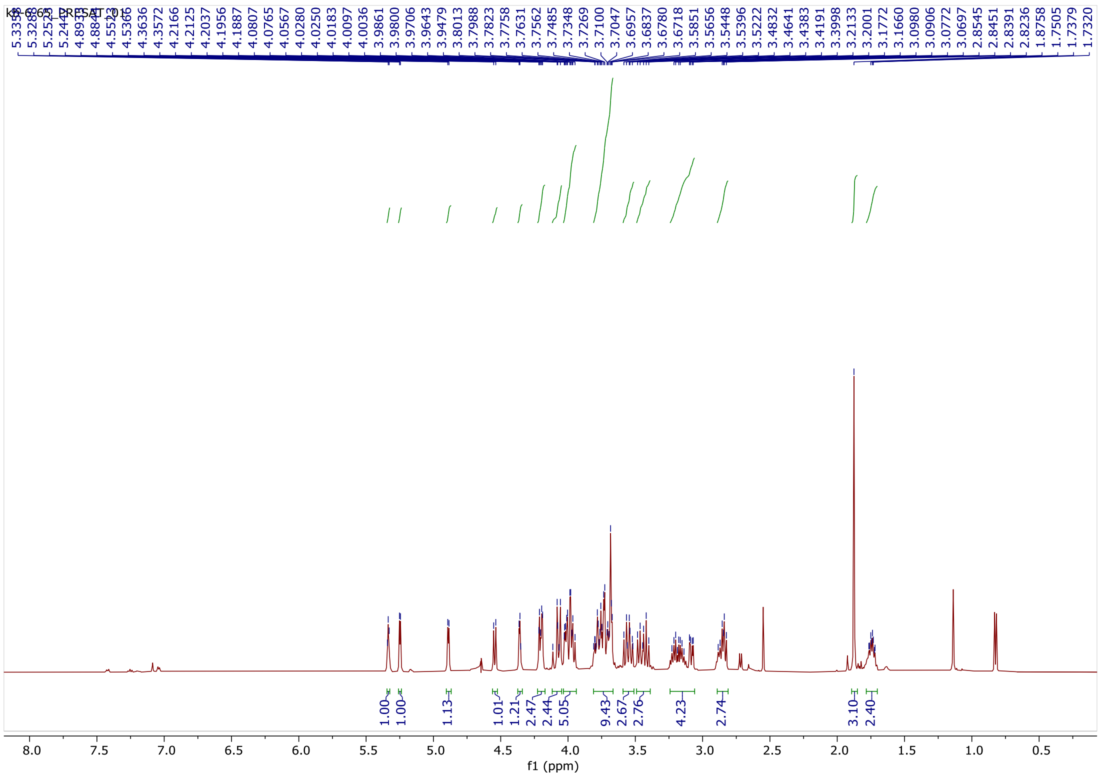

^{1}HNMR spectrum of compound **51**

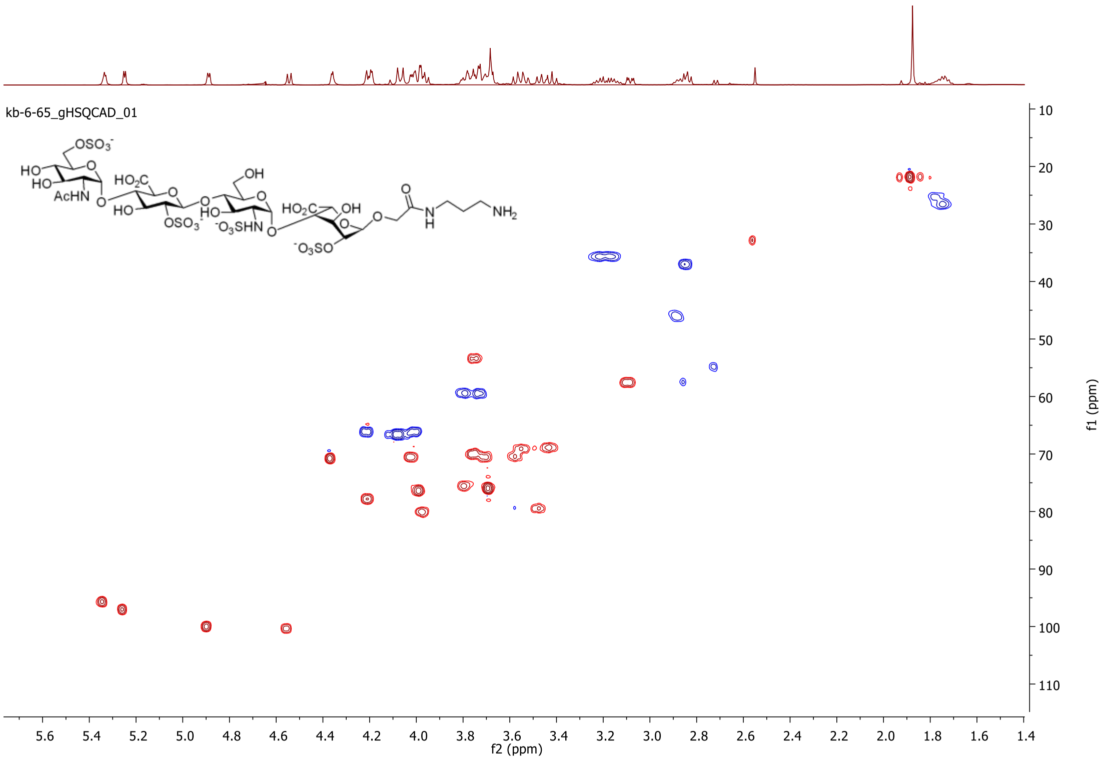

^{1}H-decoupled HSQC of compound **51**

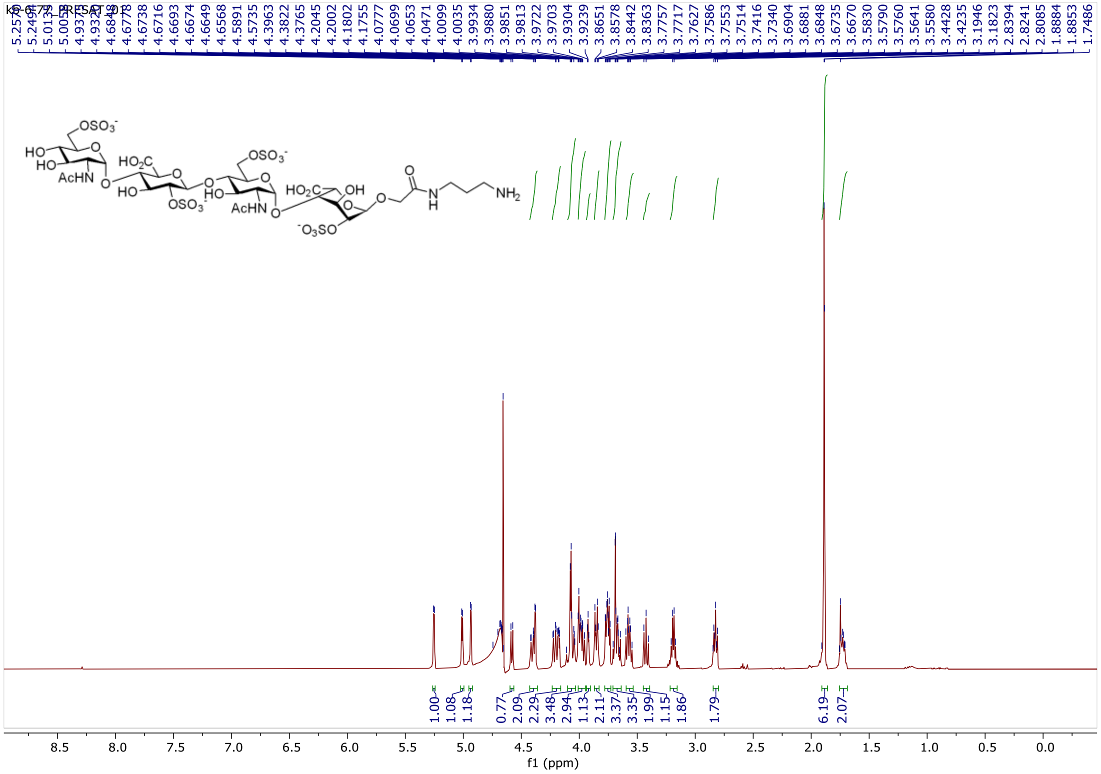

^{1}HNMR spectrum of compound **52**

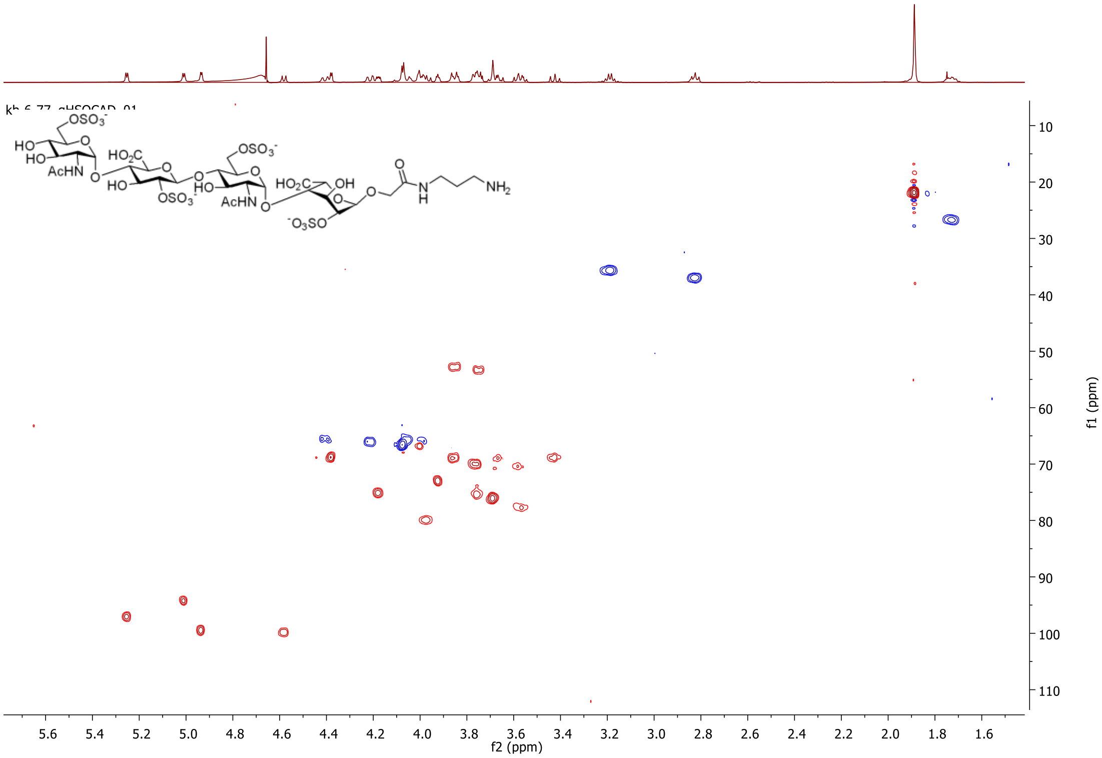

^{1}H-decoupled HSQC of compound **52**

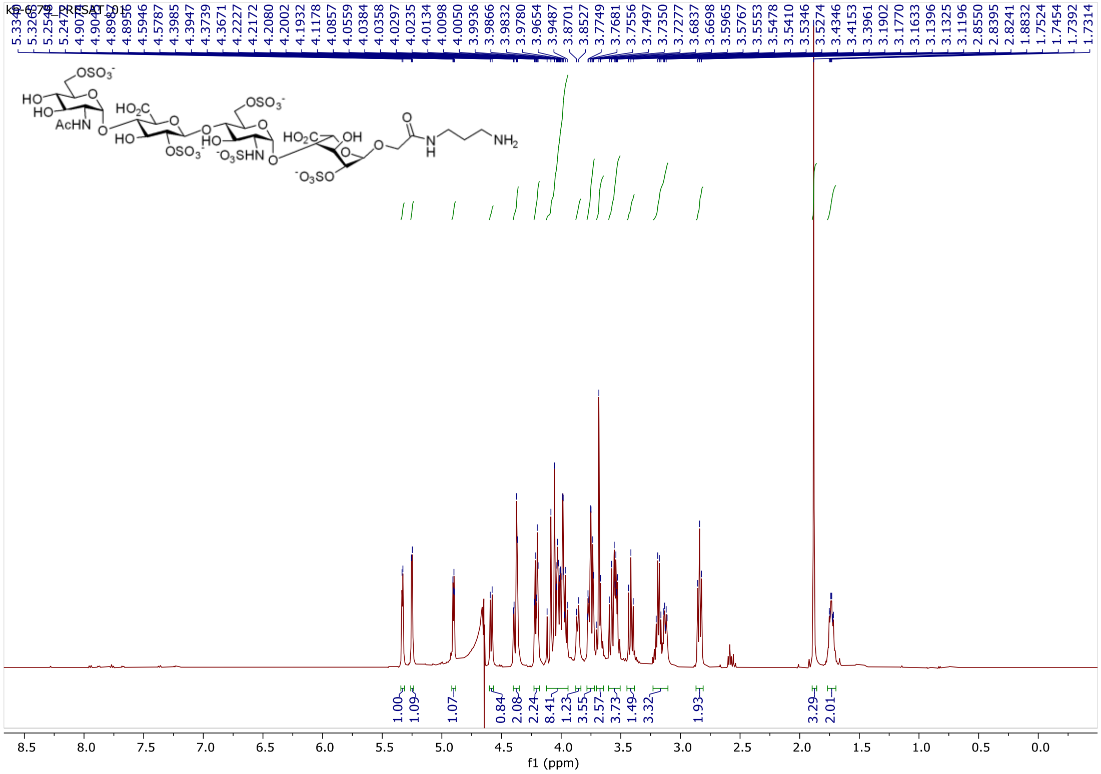

^{1}HNMR spectrum of compound **53**

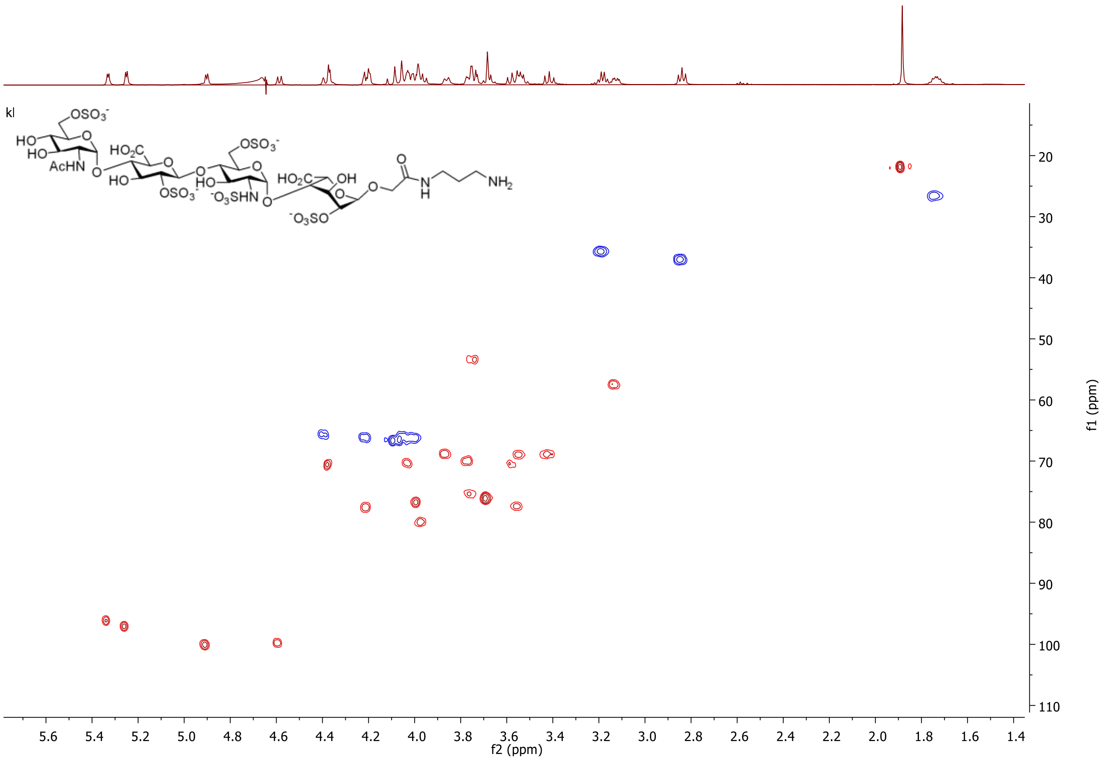

^{1}H-decoupled HSQC of compound **53**

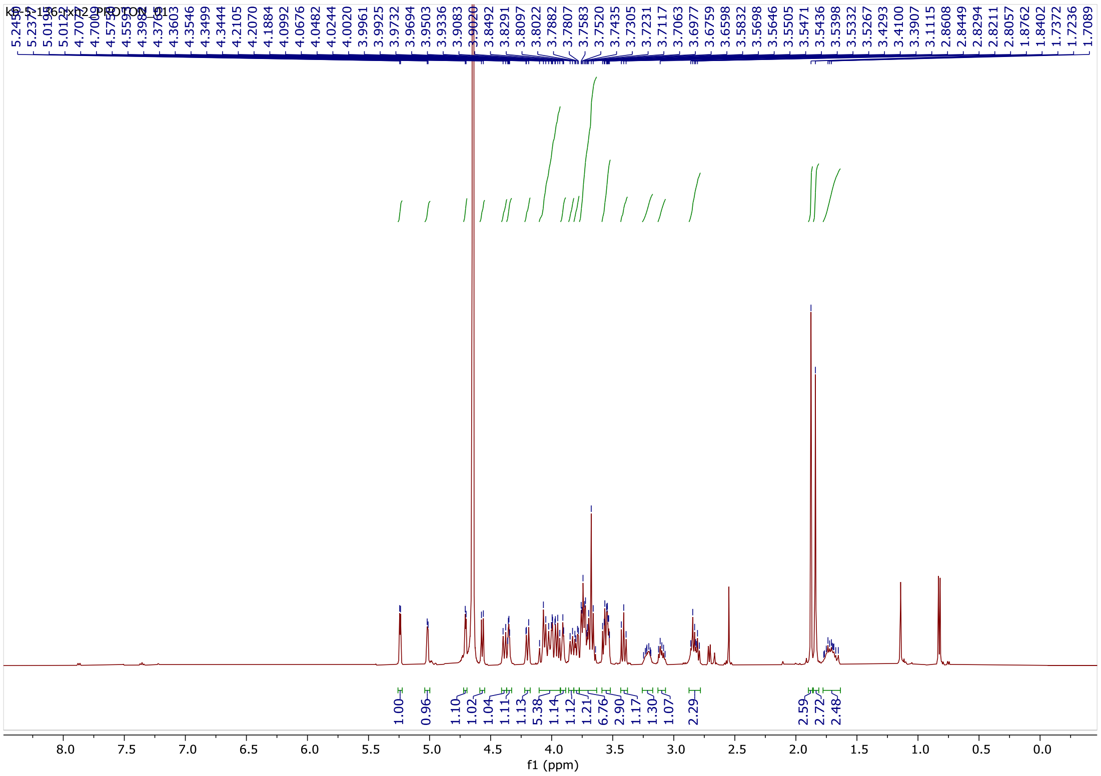

^{1}HNMR spectrum of compound **54**

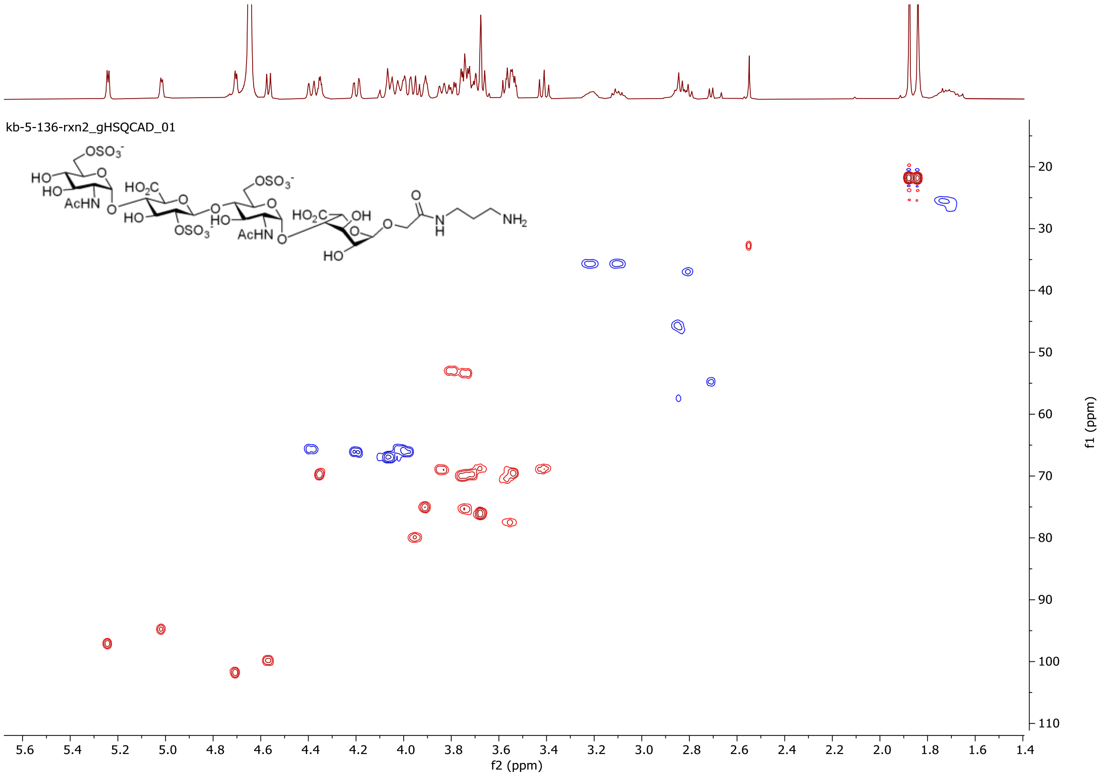

^{1}H-decoupled HSQC of compound **54**

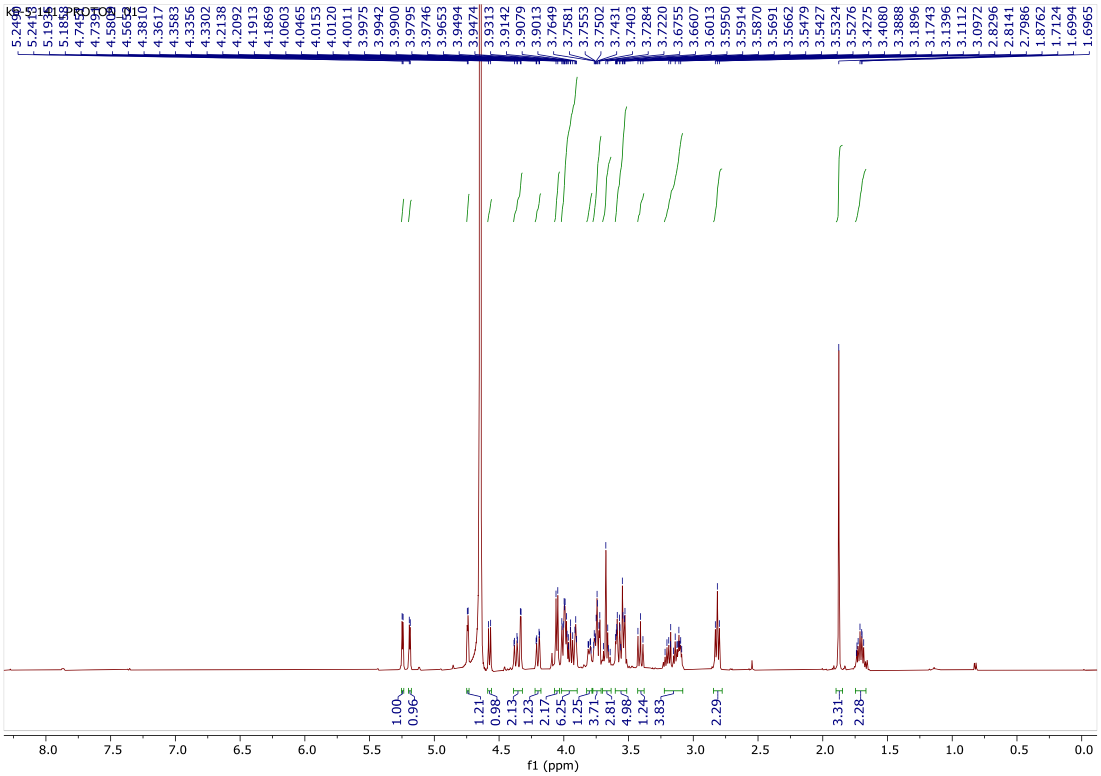

^{1}HNMR spectrum of compound **55**

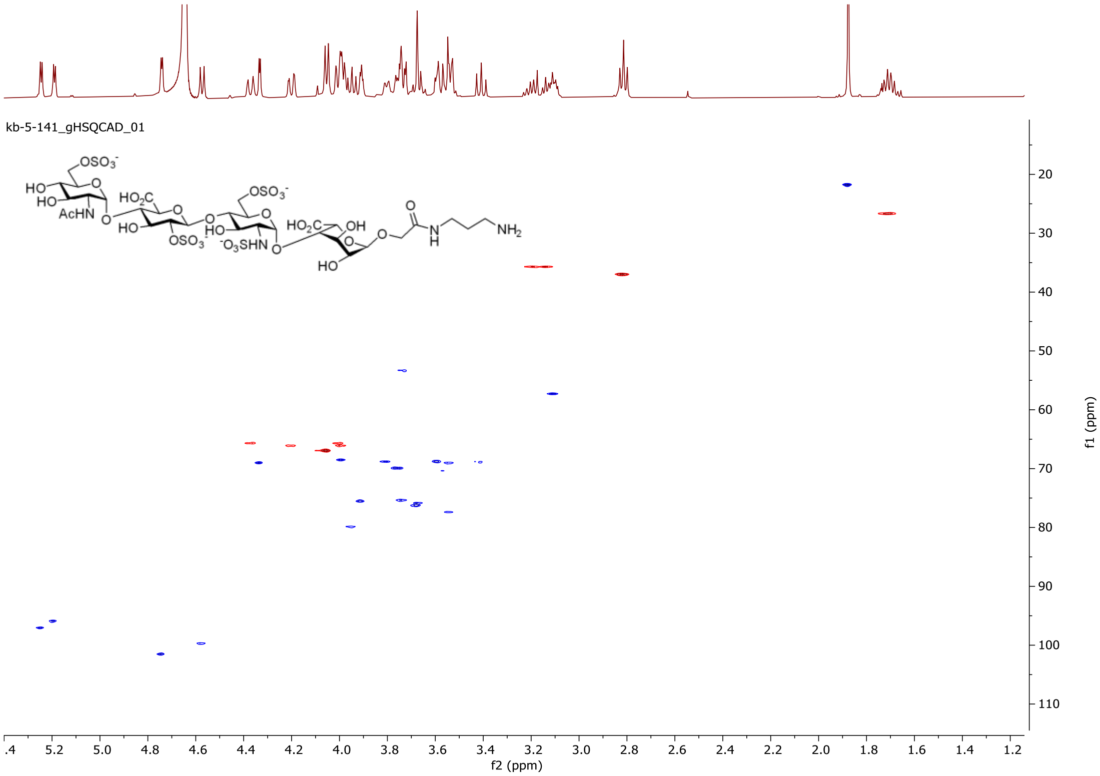

^{1}H-decoupled HSQC of compound **55**

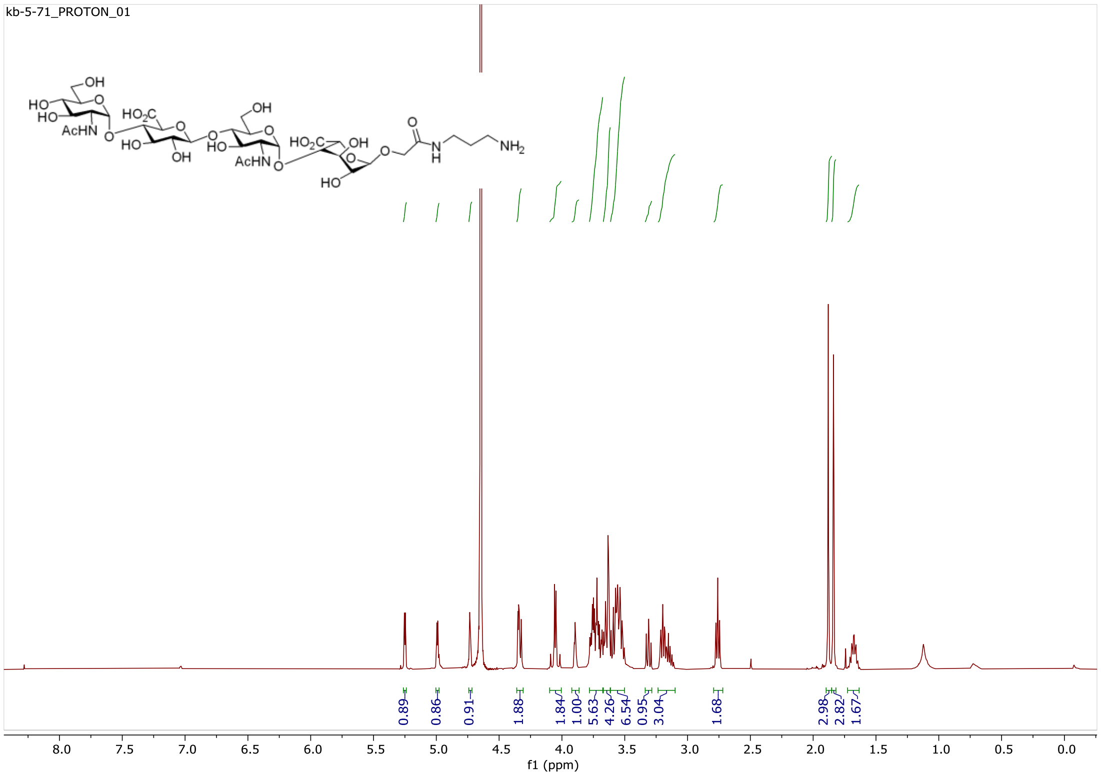

^{1}HNMR spectrum of compound **56**

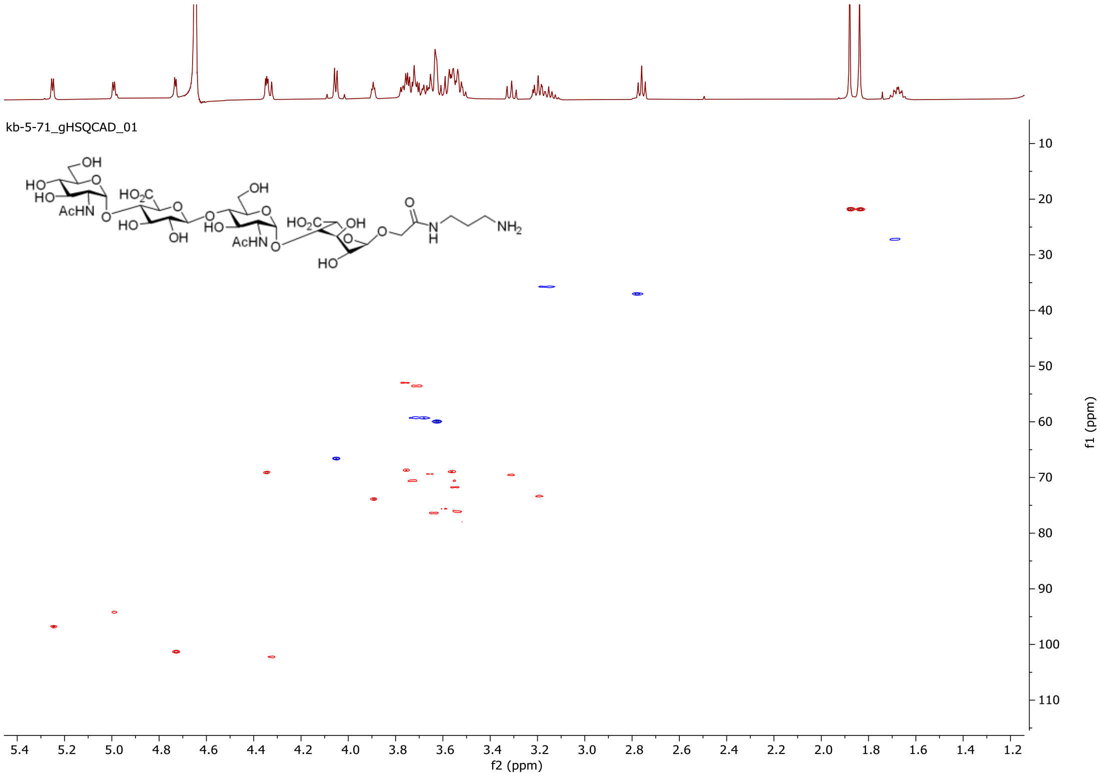

^{1}H-decoupled HSQC of compound **56**

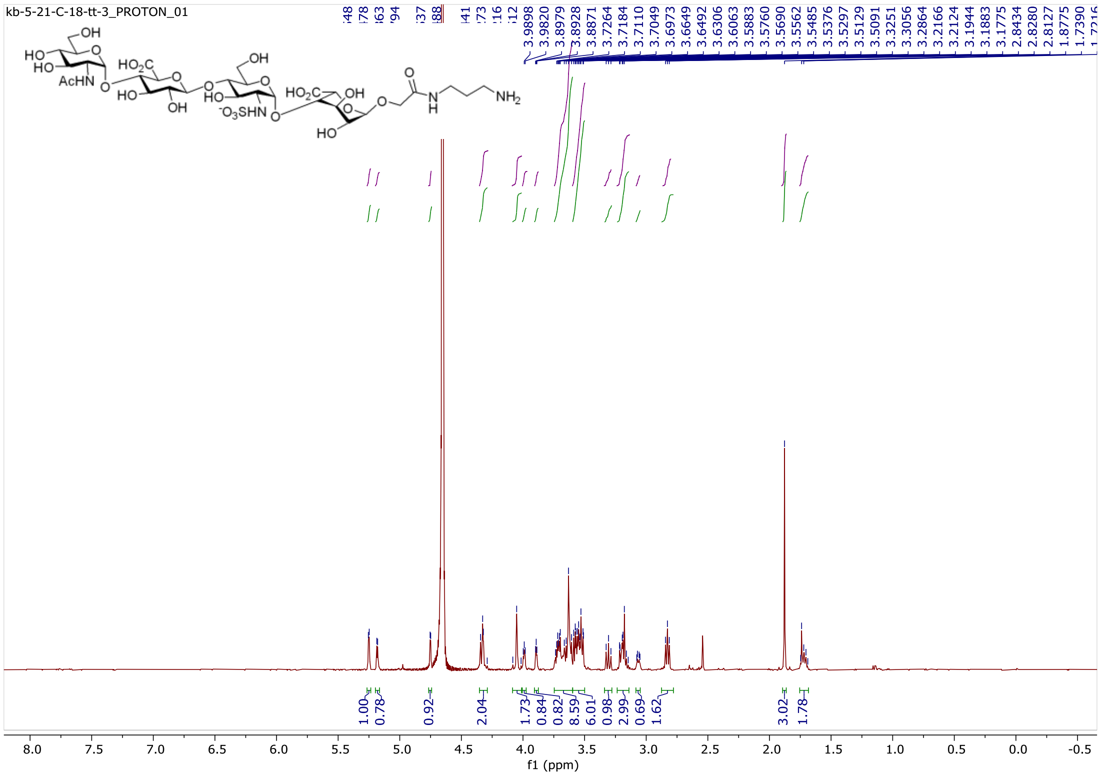

^{1}HNMR spectrum of compound **57**

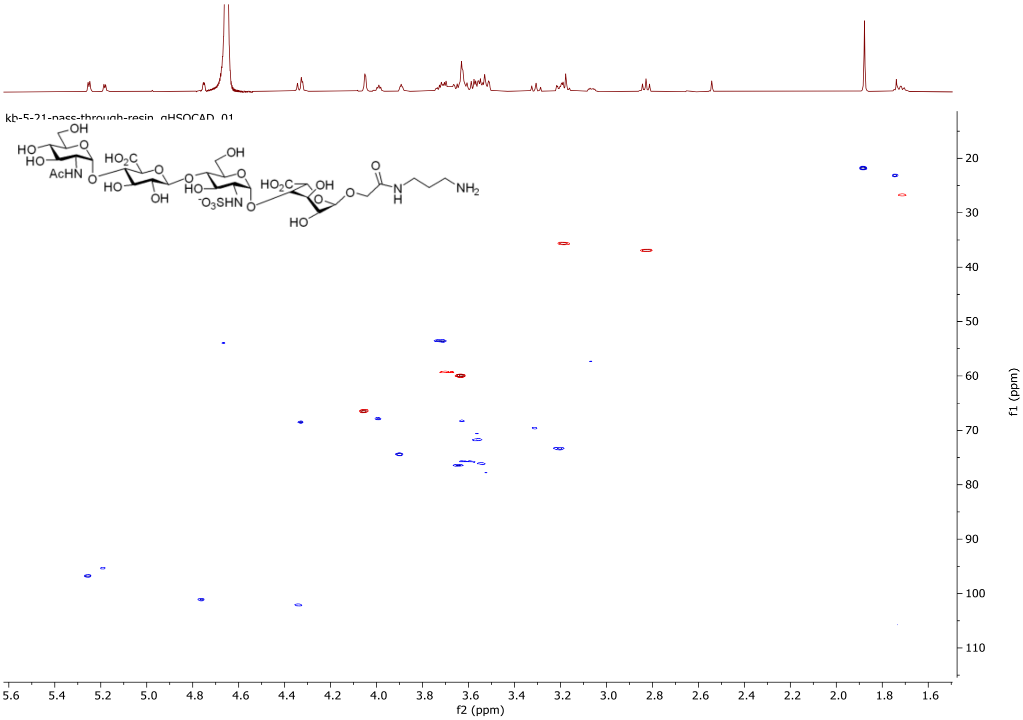

^{1}H-decoupled HSQC of compound **57**

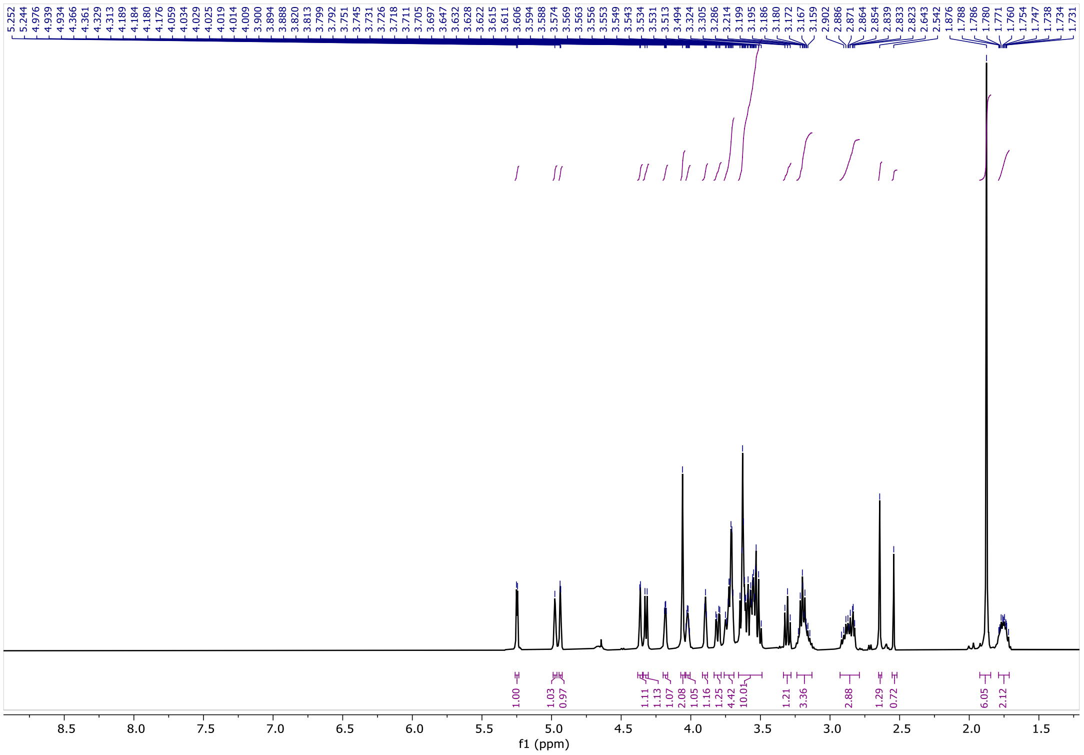

^{1}H NMR spectrum of compound **58**

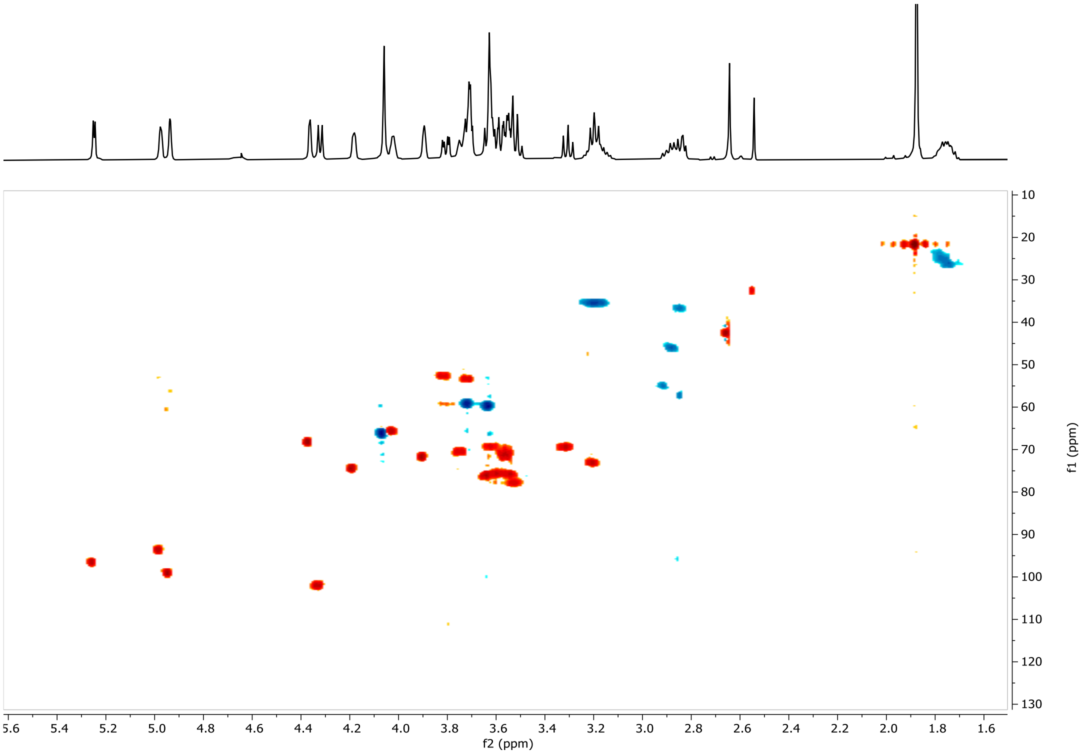

^{1}H-Coupled gHSQC NMR spectrum of compound **58**

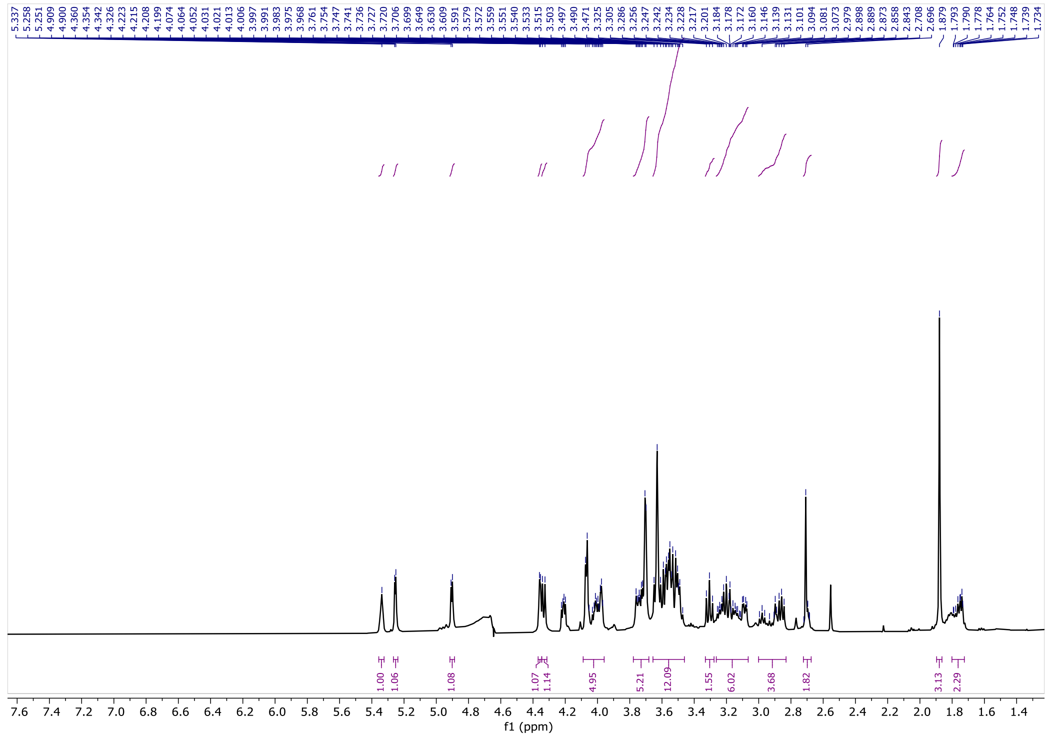

^{1}H NMR spectrum of compound **59**

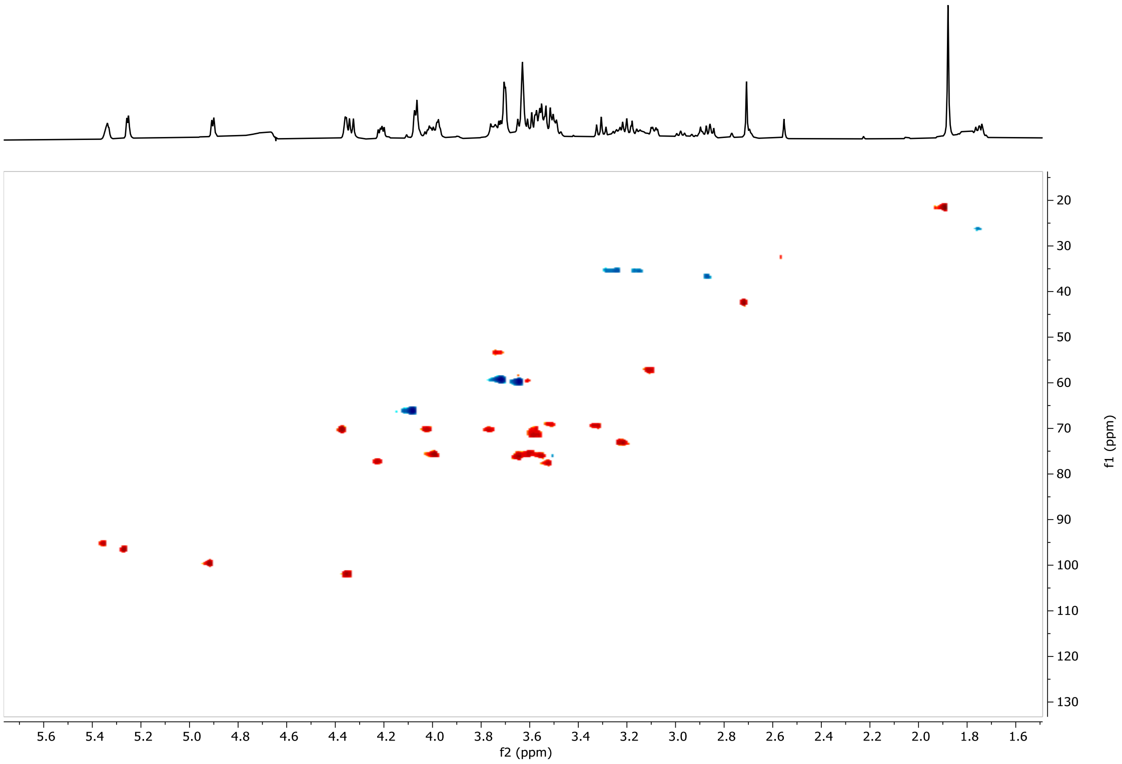

^{1}H-Coupled gHSQC NMR spectrum of compound **59**

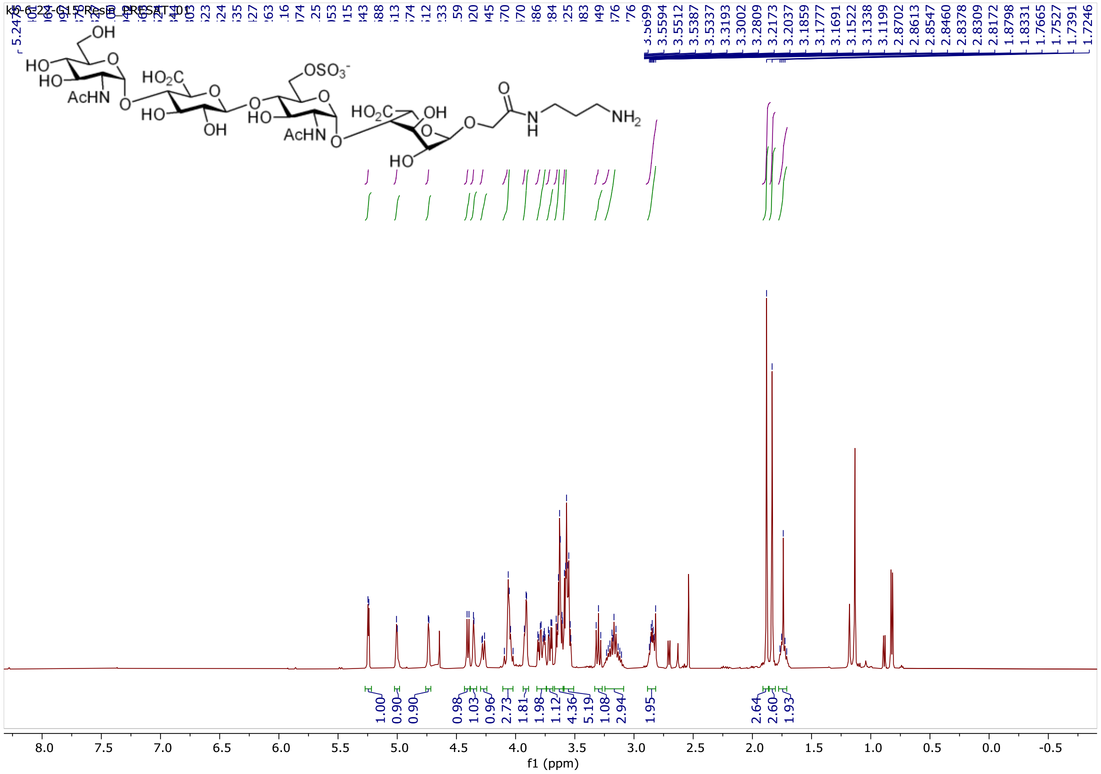

^{1}HNMR spectrum of compound **60**

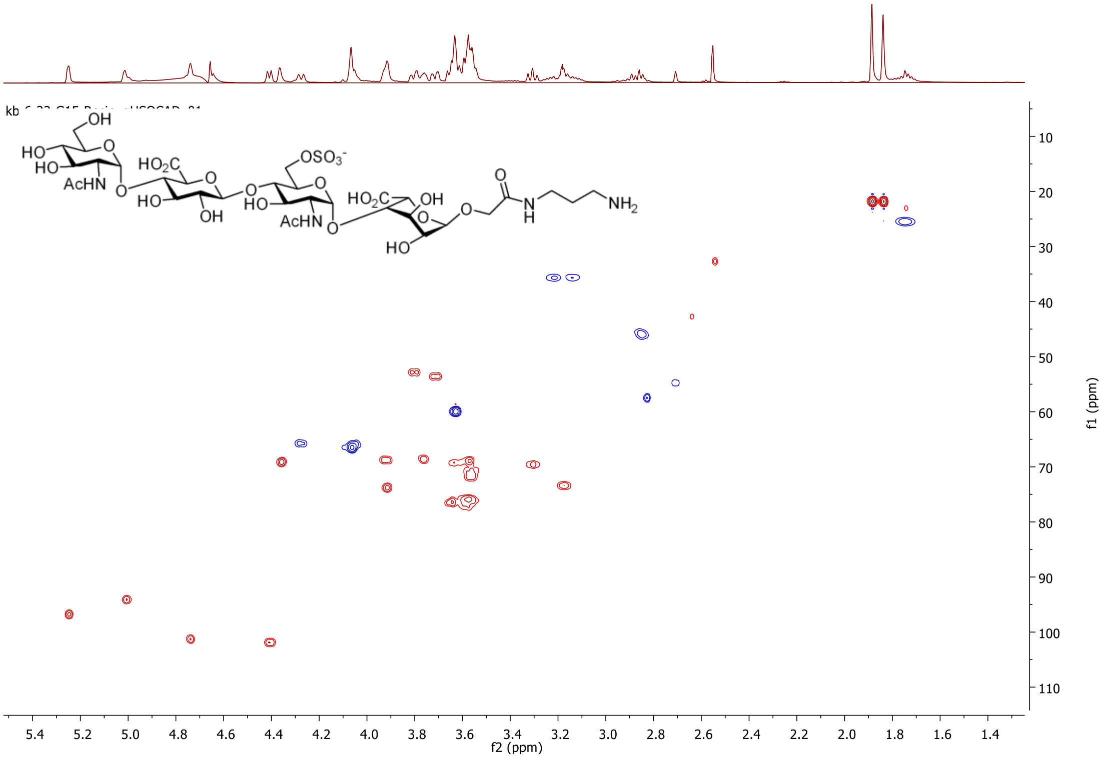

^{1}H-decoupled HSQC of compound **60**

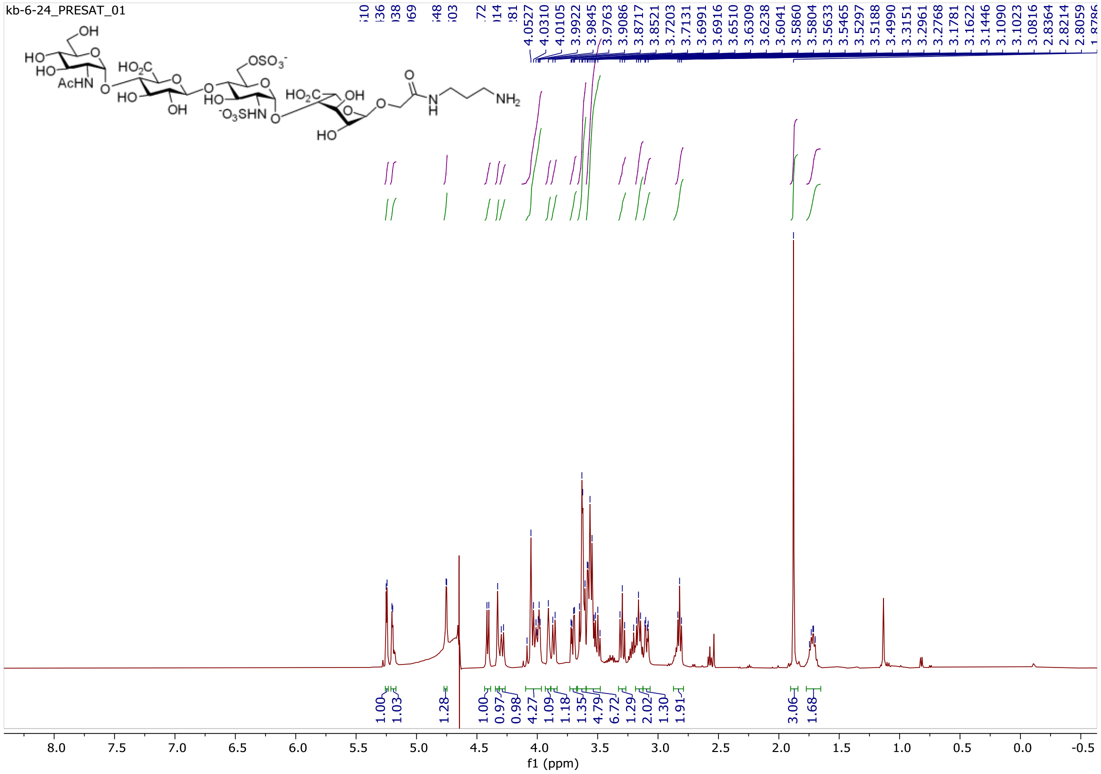

^{1}HNMR spectrum of compound **61**

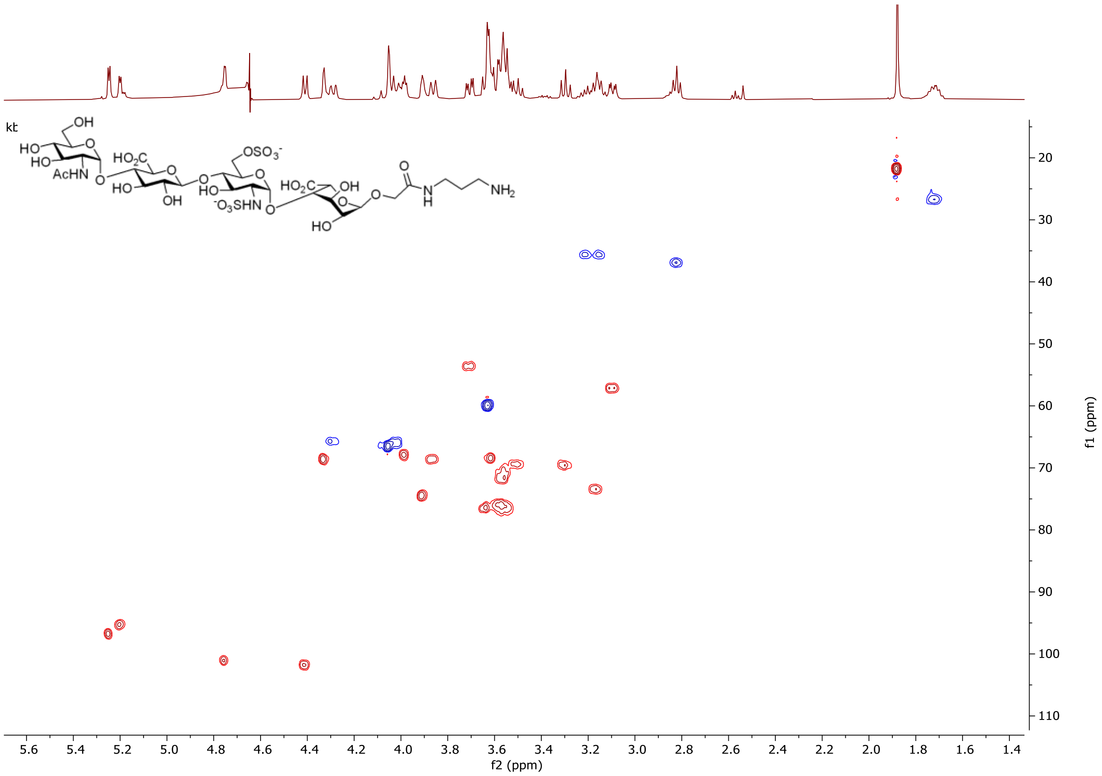

^{1}H-decoupled HSQC of compound **61**

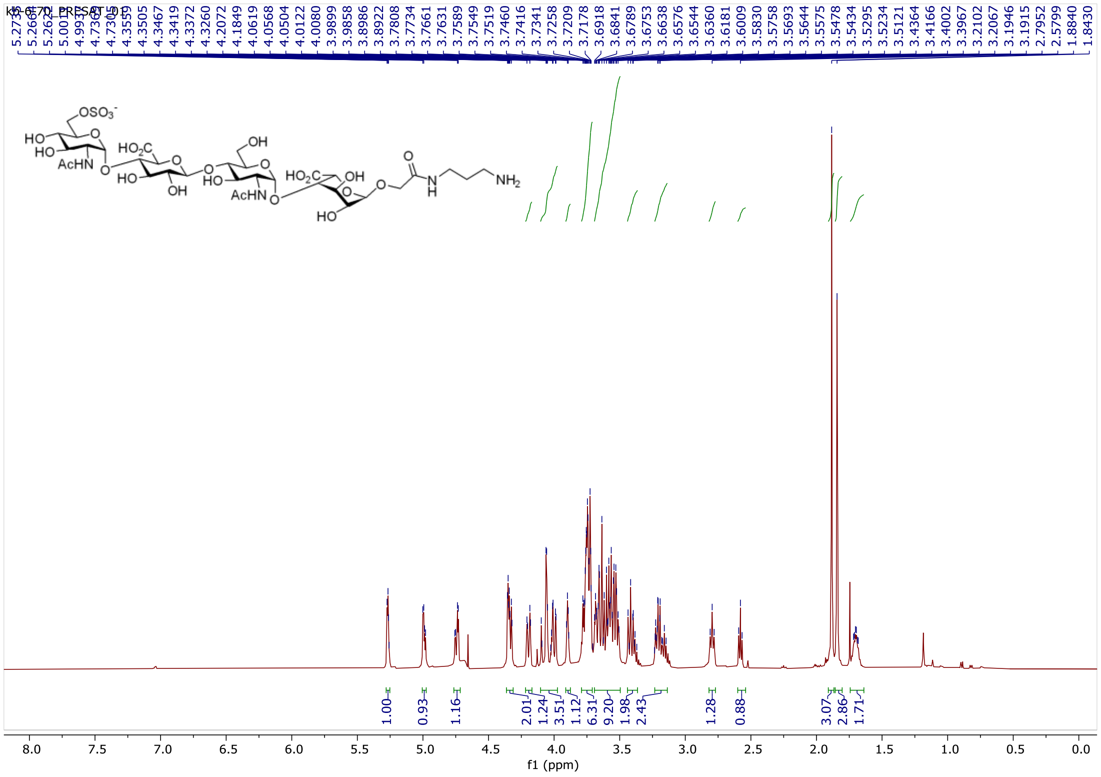

^{1}HNMR spectrum of compound **62**

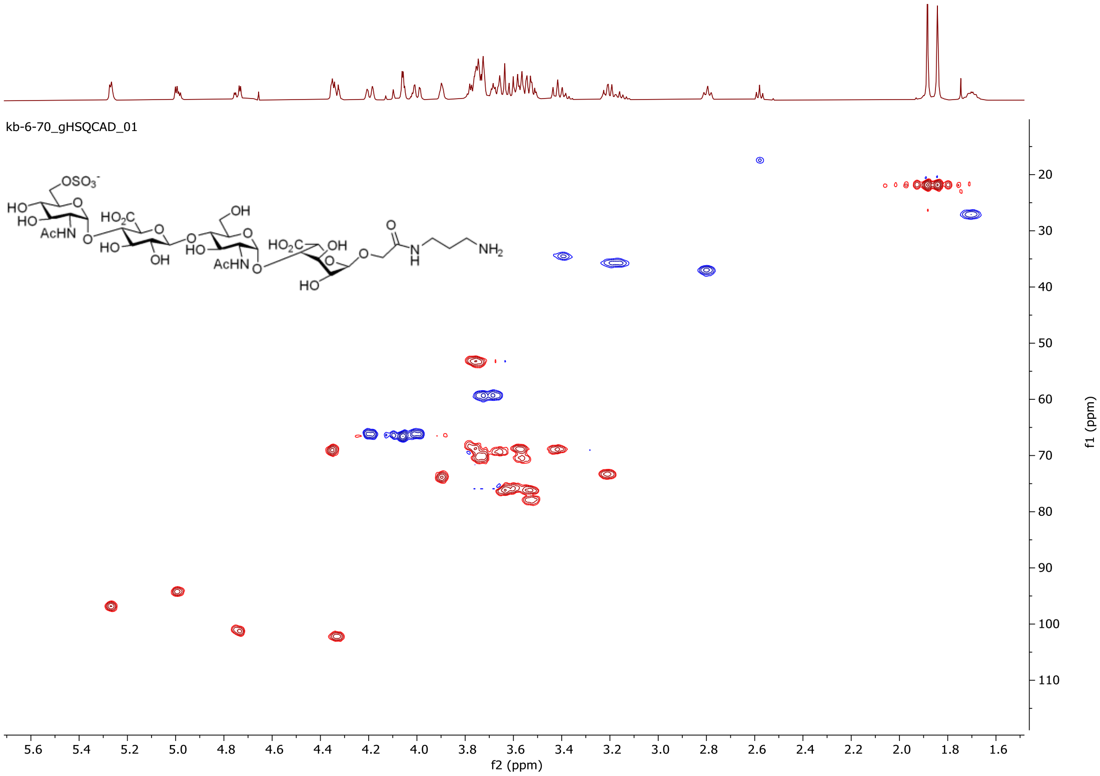

^{1}H-decoupled HSQC of compound **62**

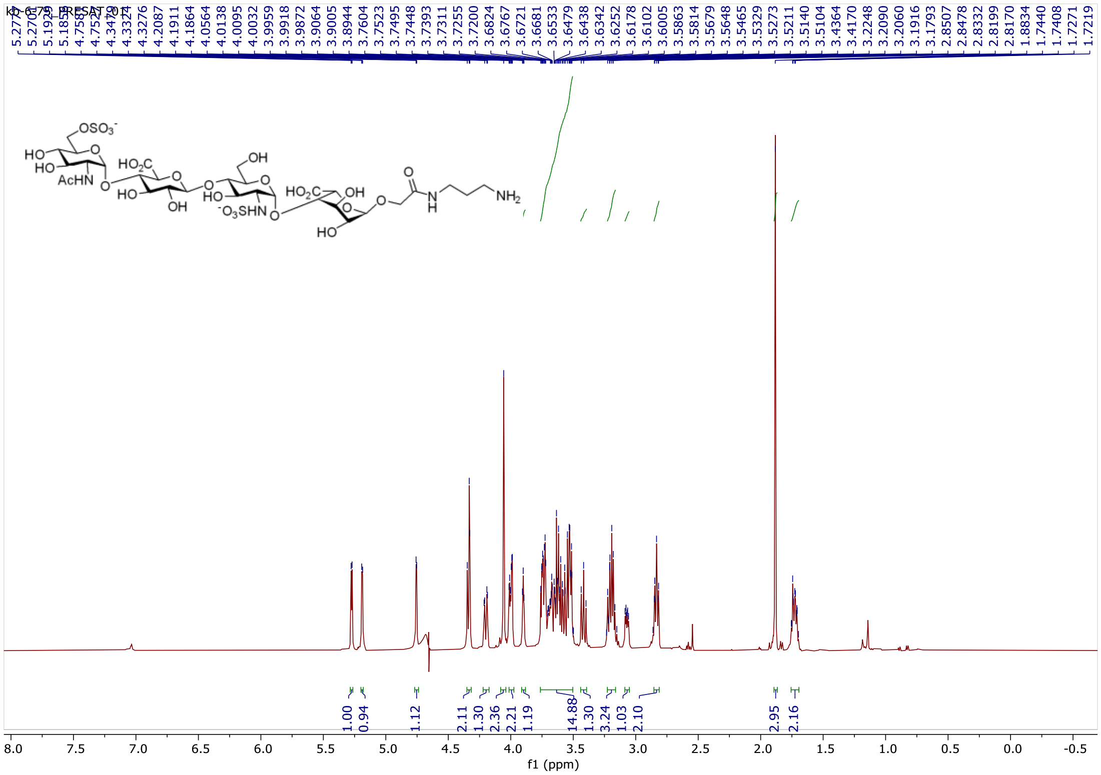

^{1}HNMR spectrum of compound **63**

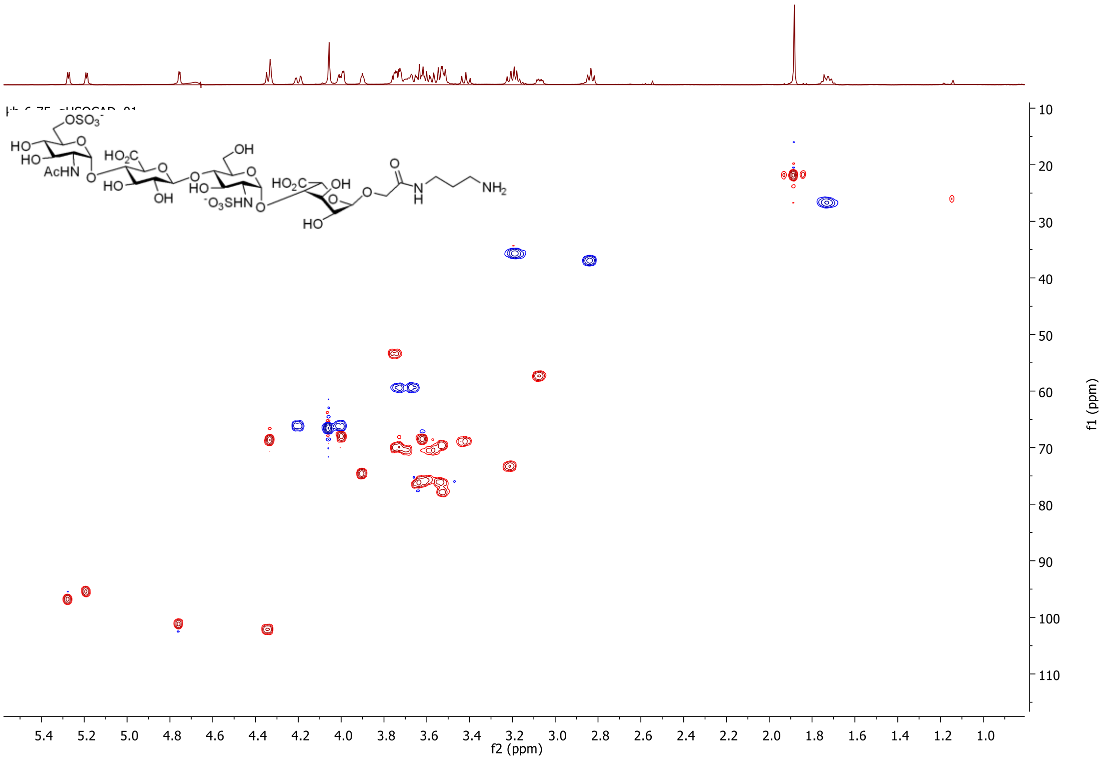

^{1}H-decoupled HSQC of compound **63**

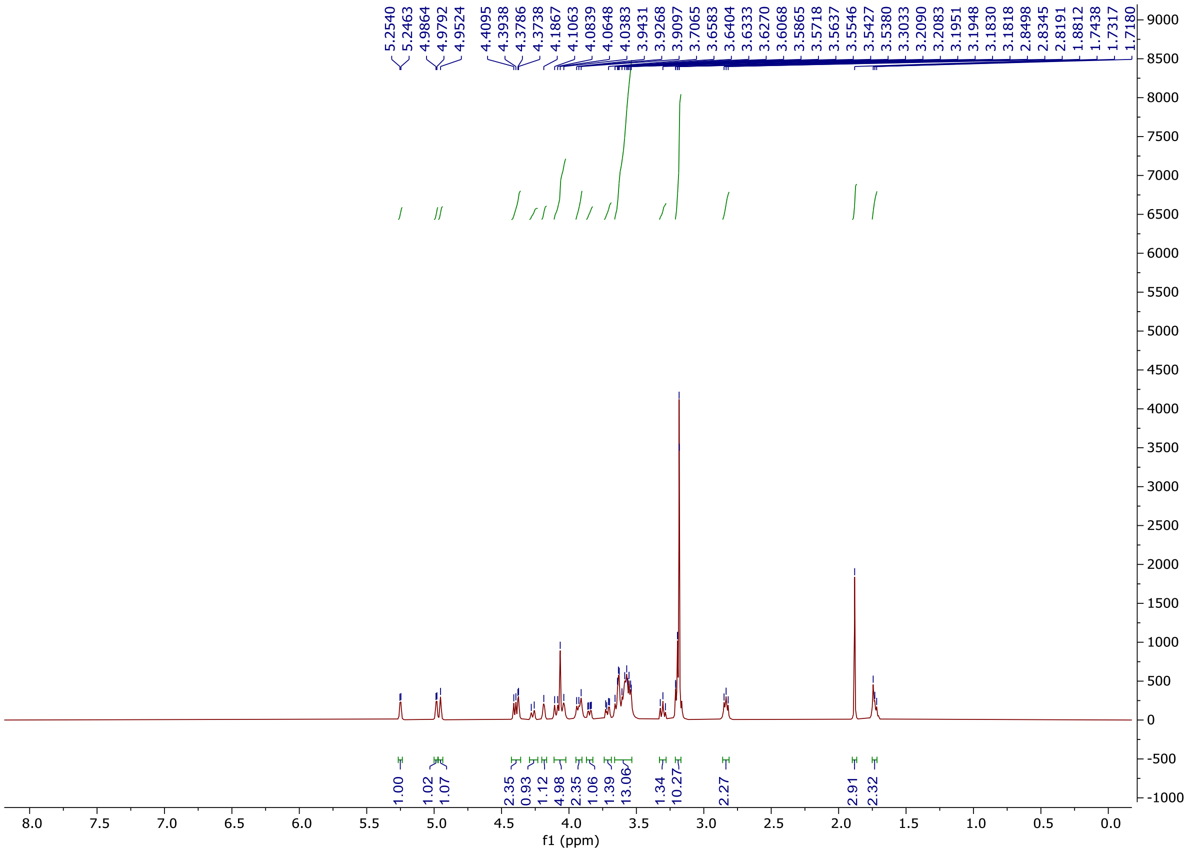

^{1}H NMR spectrum of compound **64**

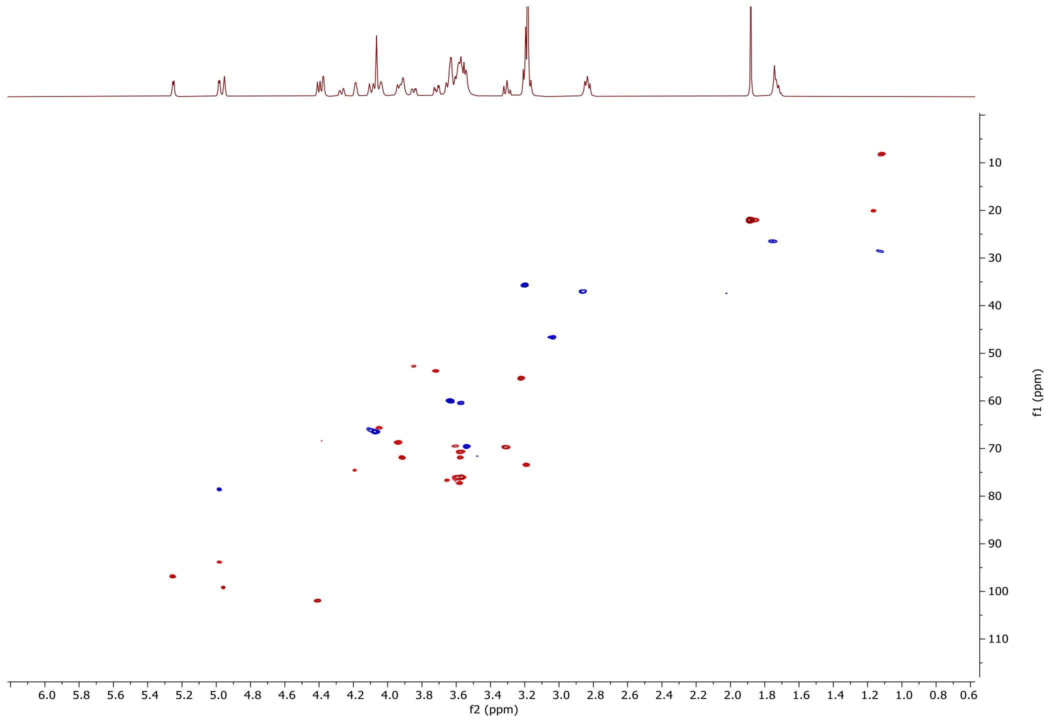

^{1}H-decoupled HSQC of compound **64**

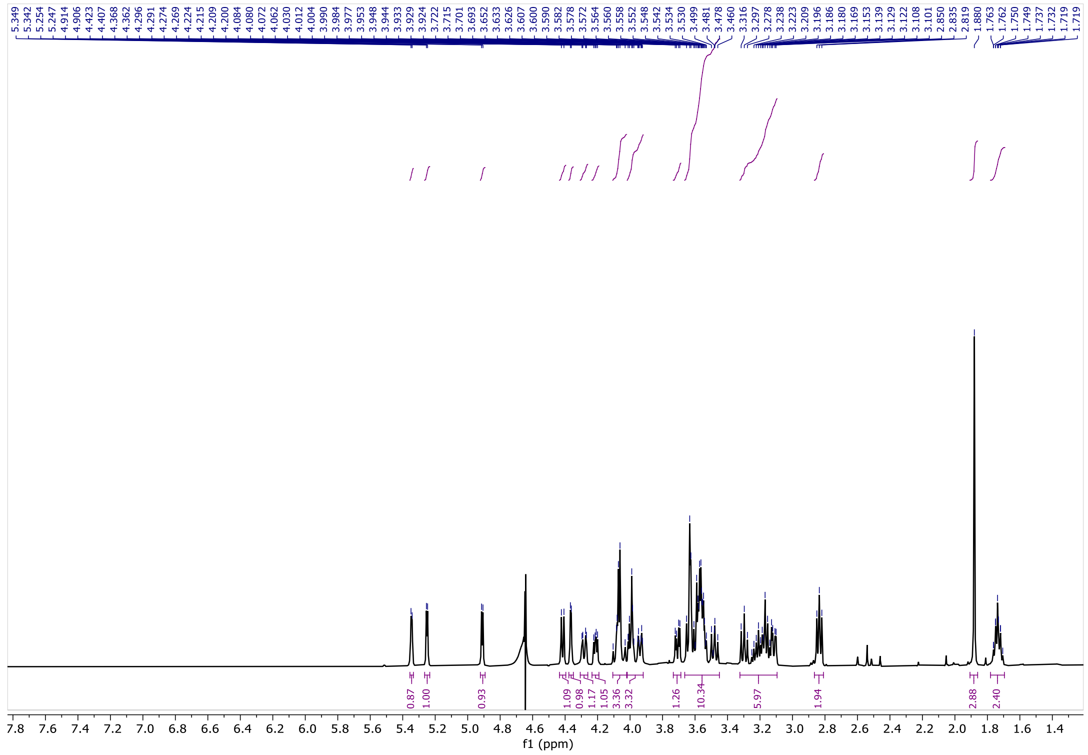

^{1}H NMR spectrum of compound **65**

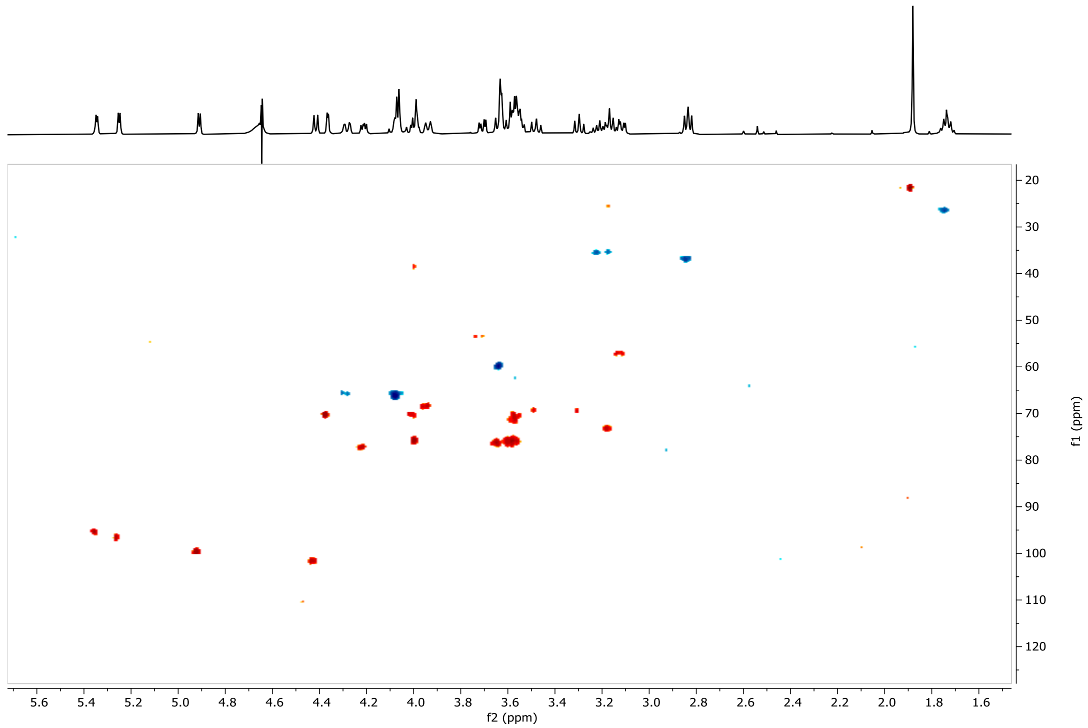

^{1}H-decoupled gHSQC NMR spectrum of compound **65**

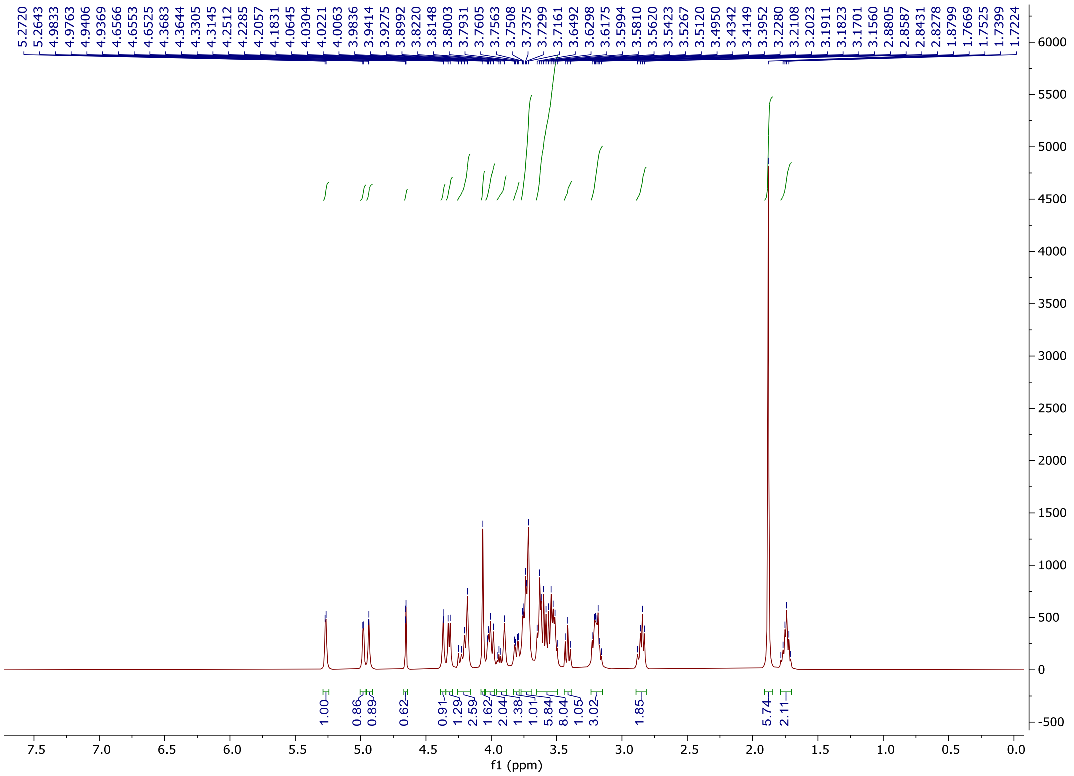

^{1}H NMR spectrum of compound **66**

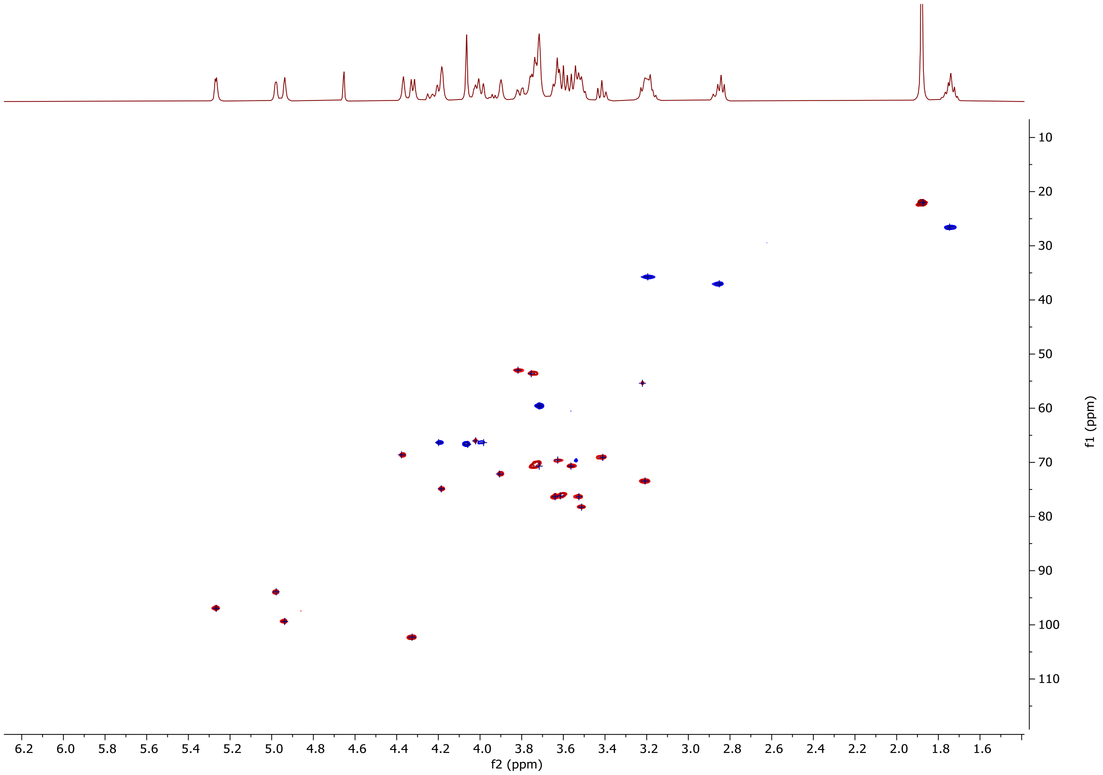

^{1}H-decoupled gHSQC NMR spectrum of compound **66**

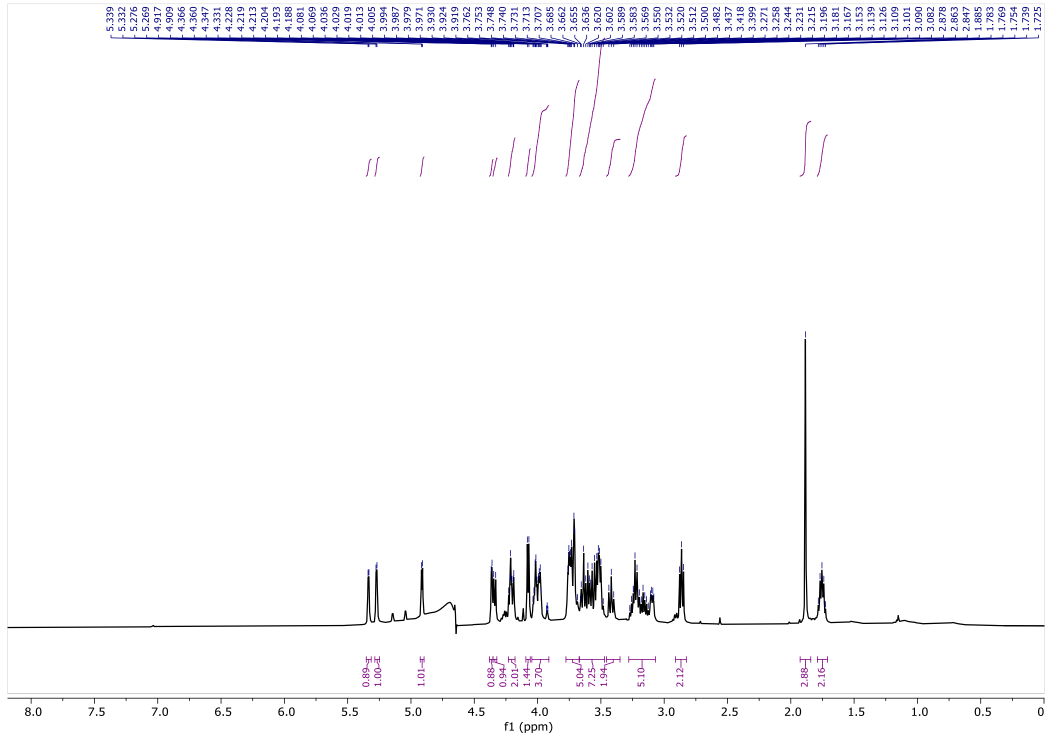

^{1}H NMR spectrum of compound **67**

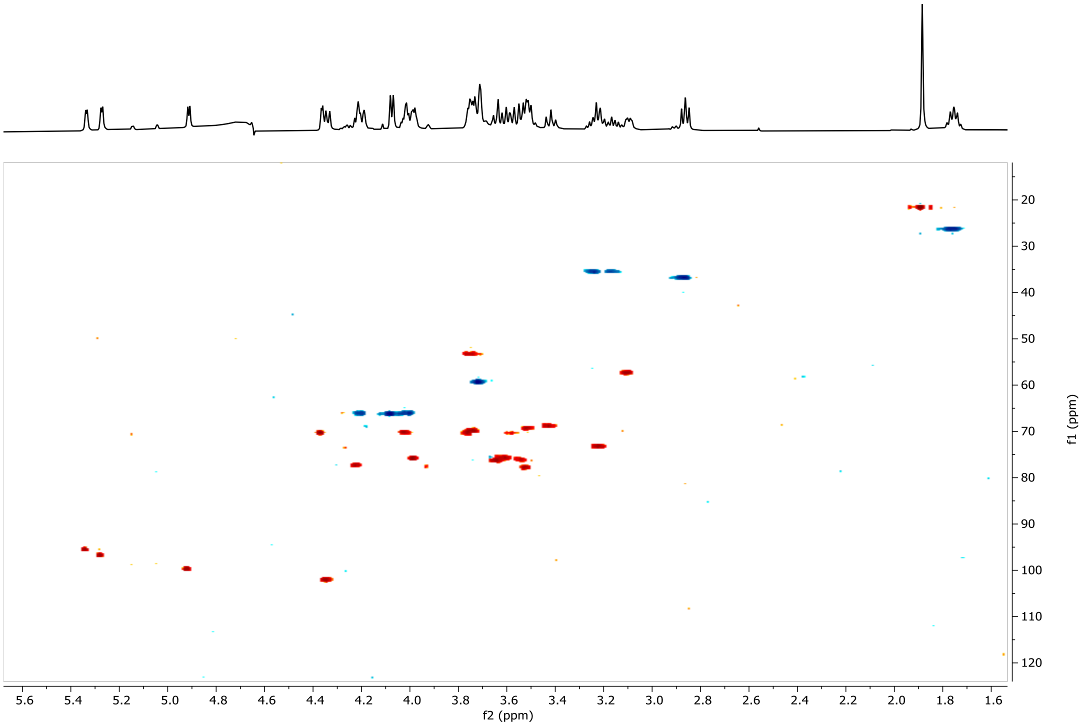

^{1}H-decoupled gHSQC NMR spectrum of compound **67**

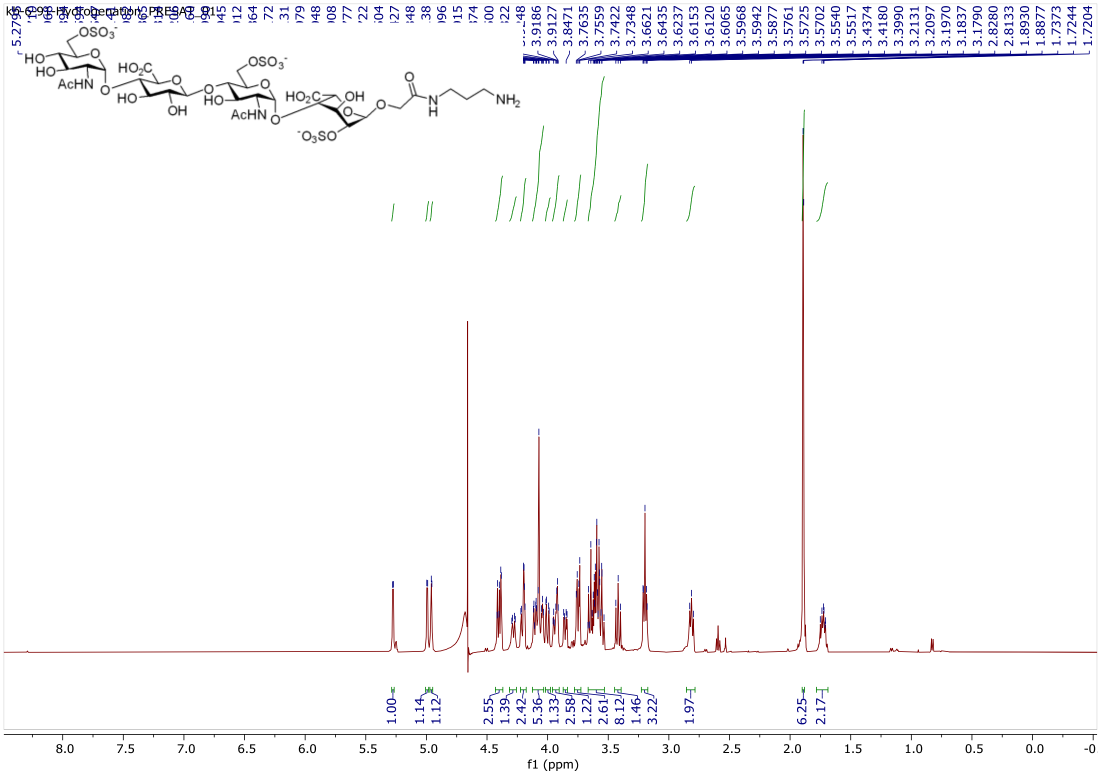

^{1}HNMR spectrum of compound **68**

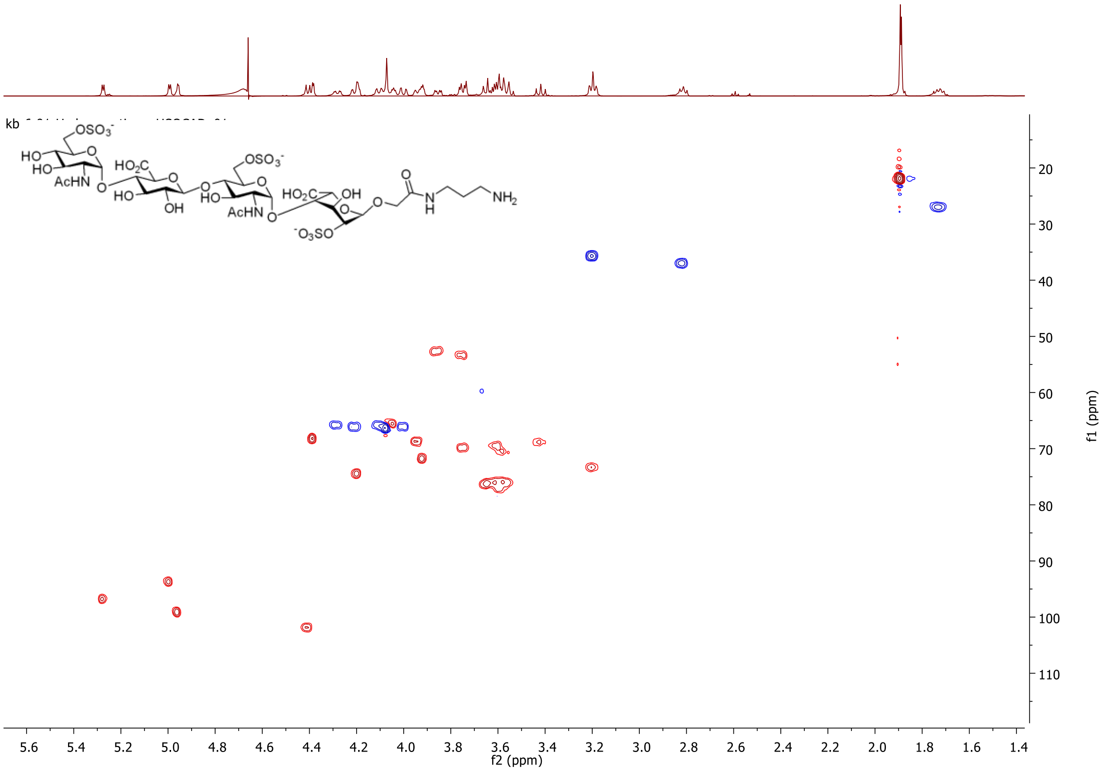

^{1}H-decoupled HSQC of compound **68**

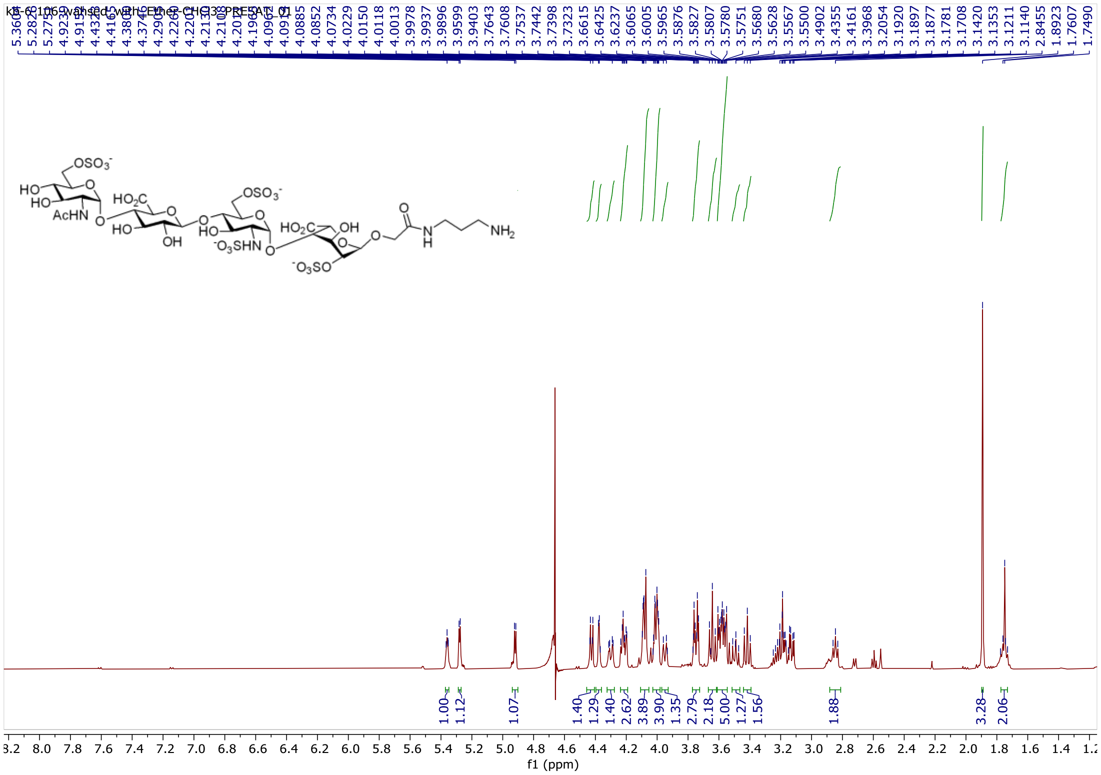

^{1}HNMR spectrum of compound **69**

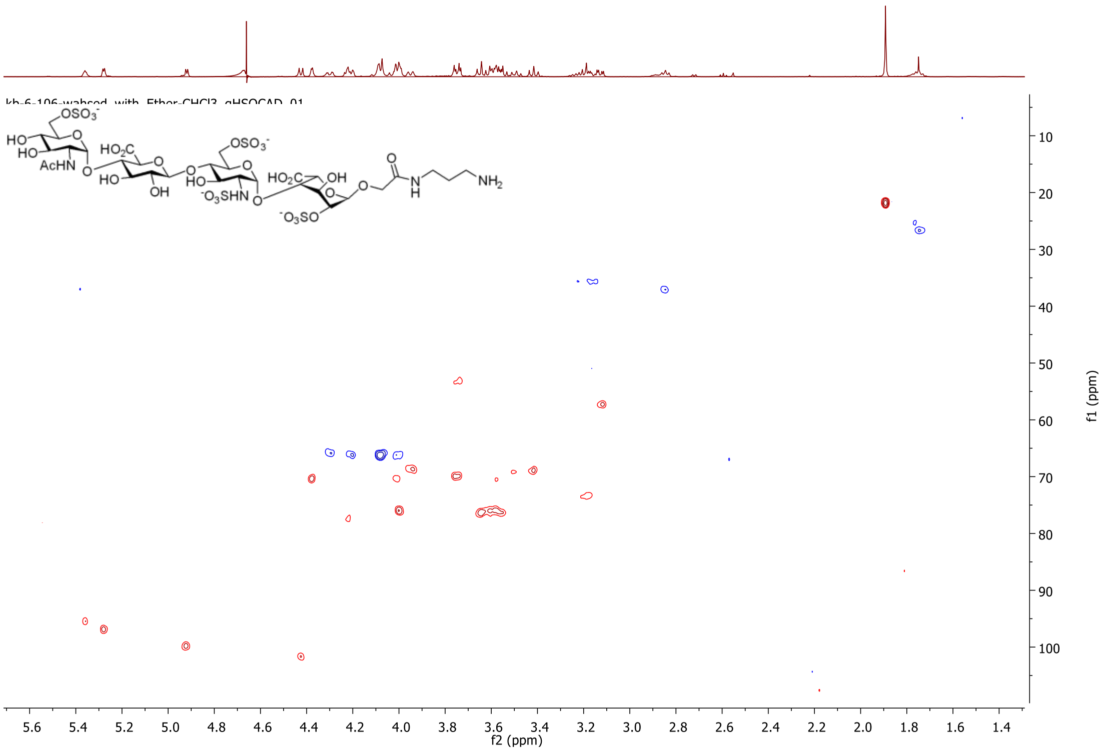

^{1}H-decoupled HSQC of compound **69**

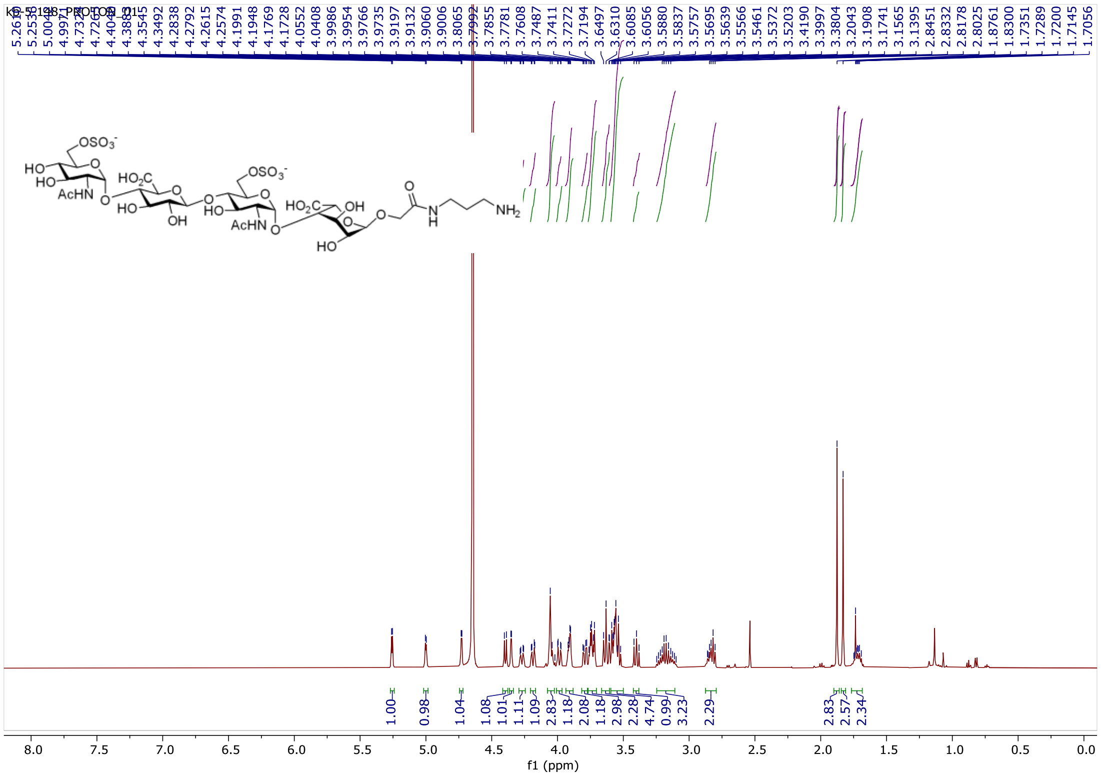

^{1}HNMR spectrum of compound **70**

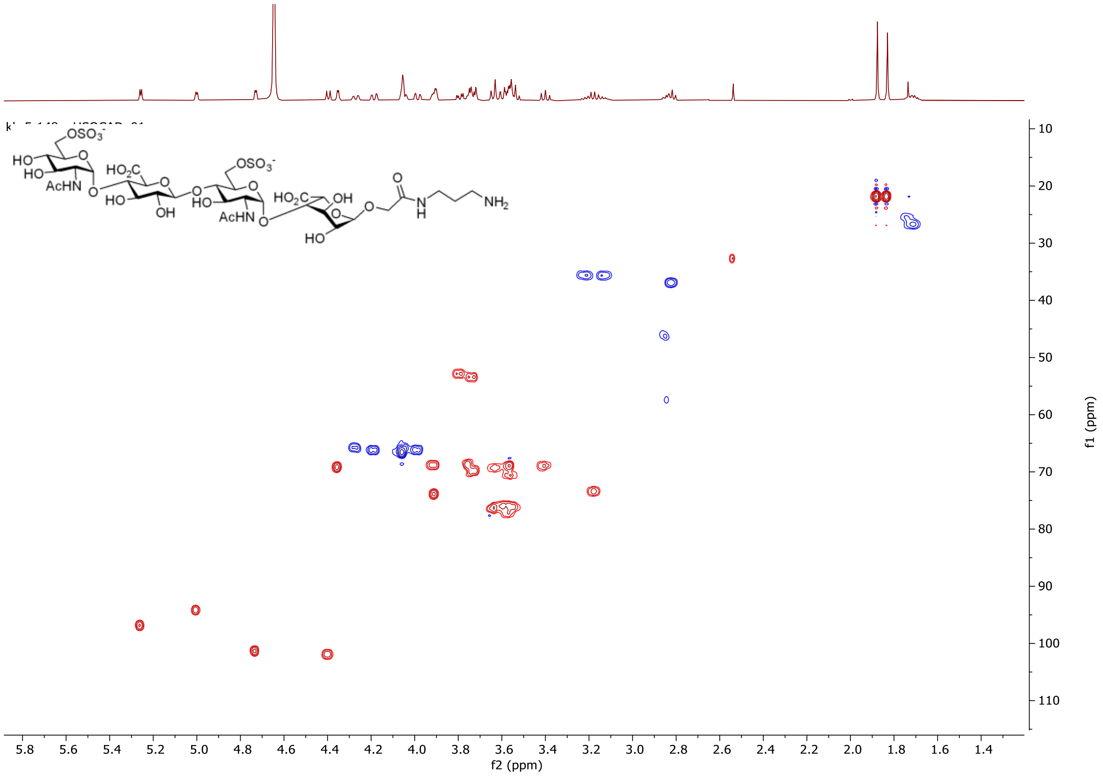

^{1}H-decoupled HSQC of compound **70**

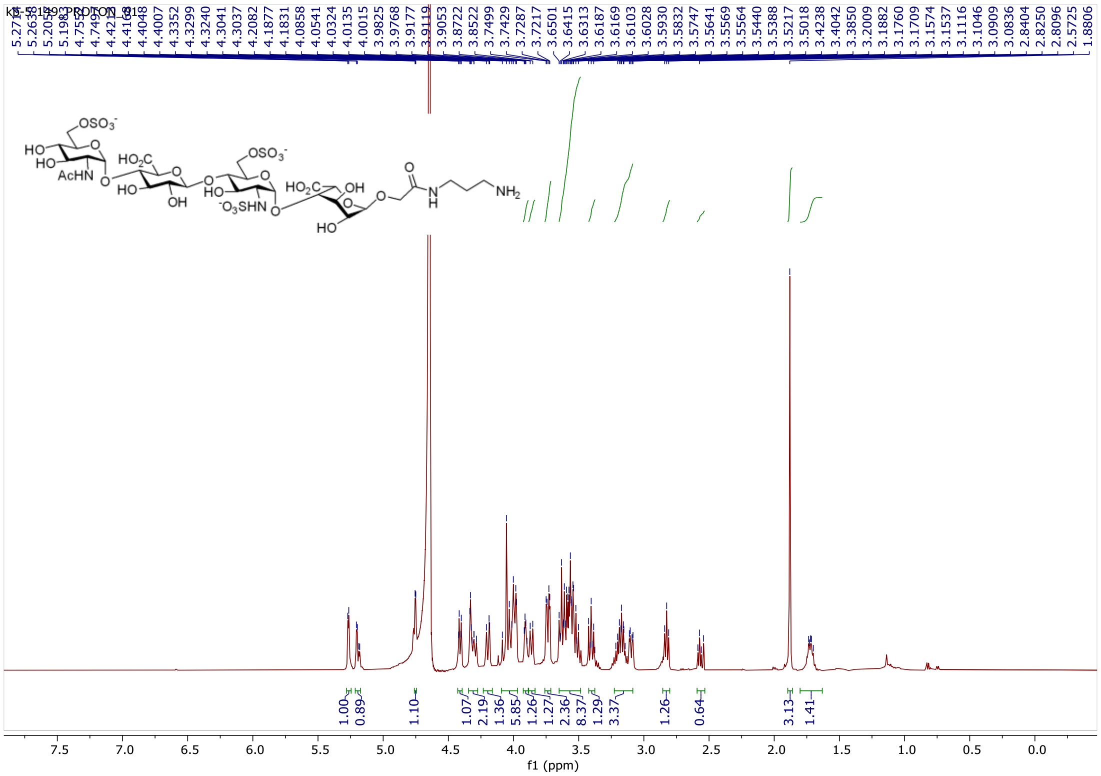

^{1}HNMR spectrum of compound **71**

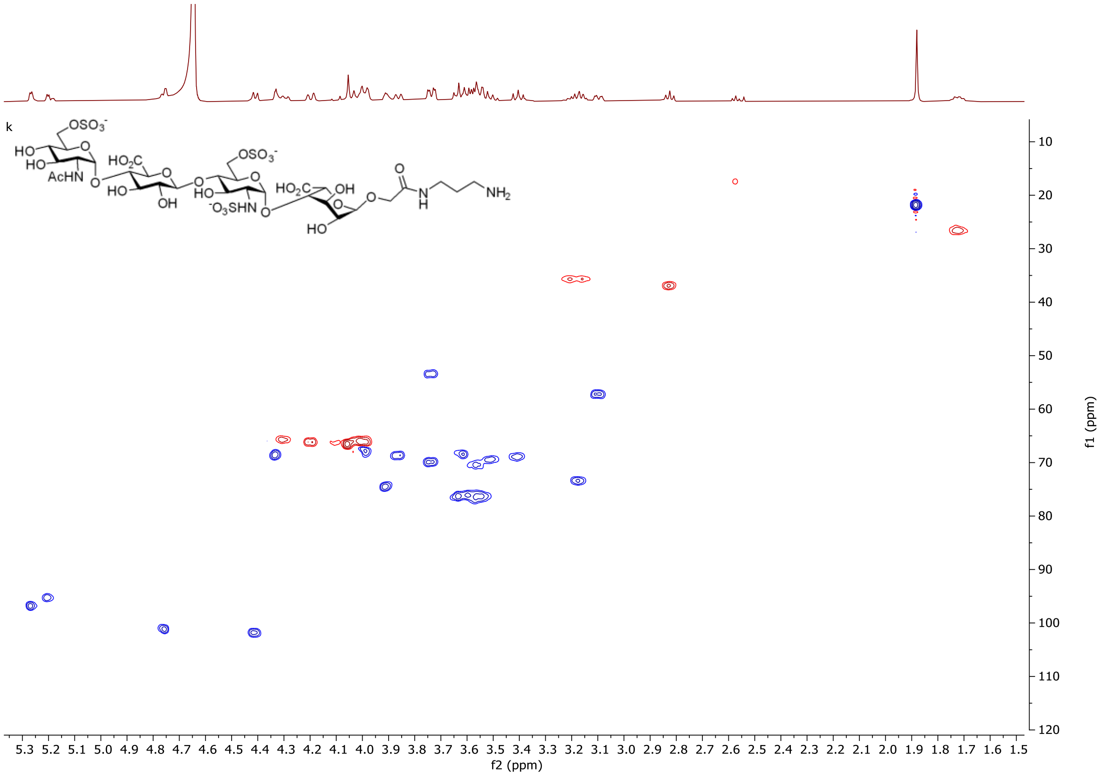

^{1}H-decoupled HSQC of compound **71**

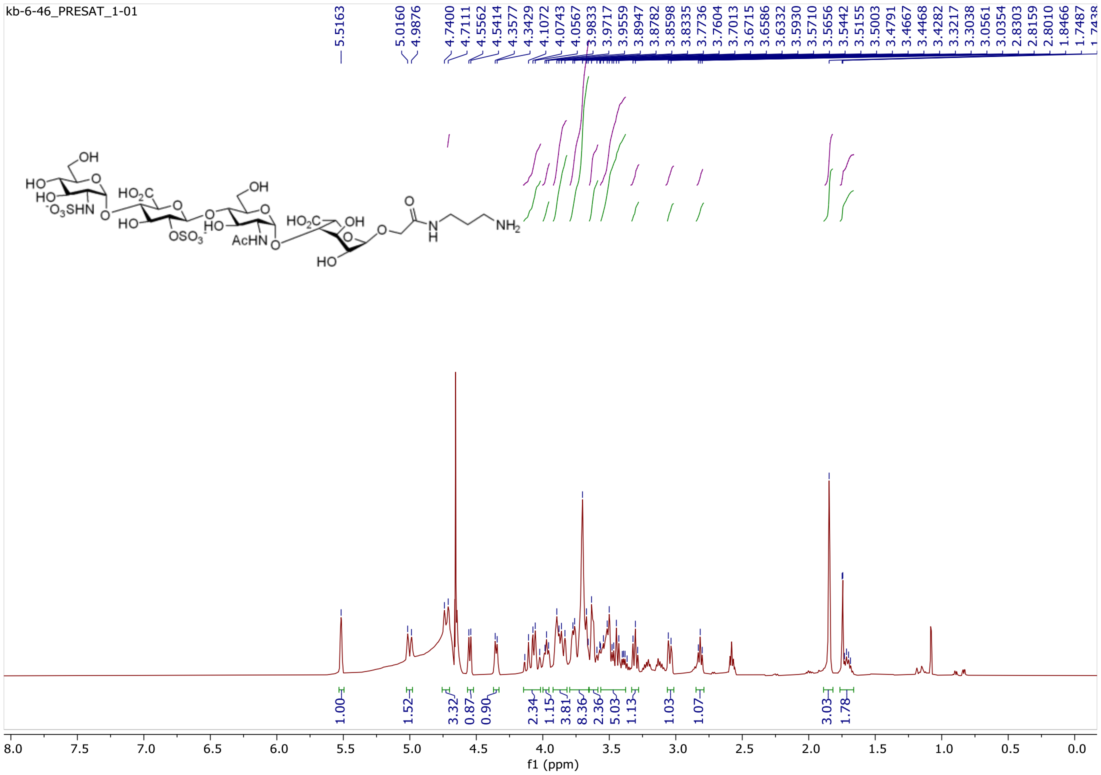

^{1}HNMR spectrum of compound **72**

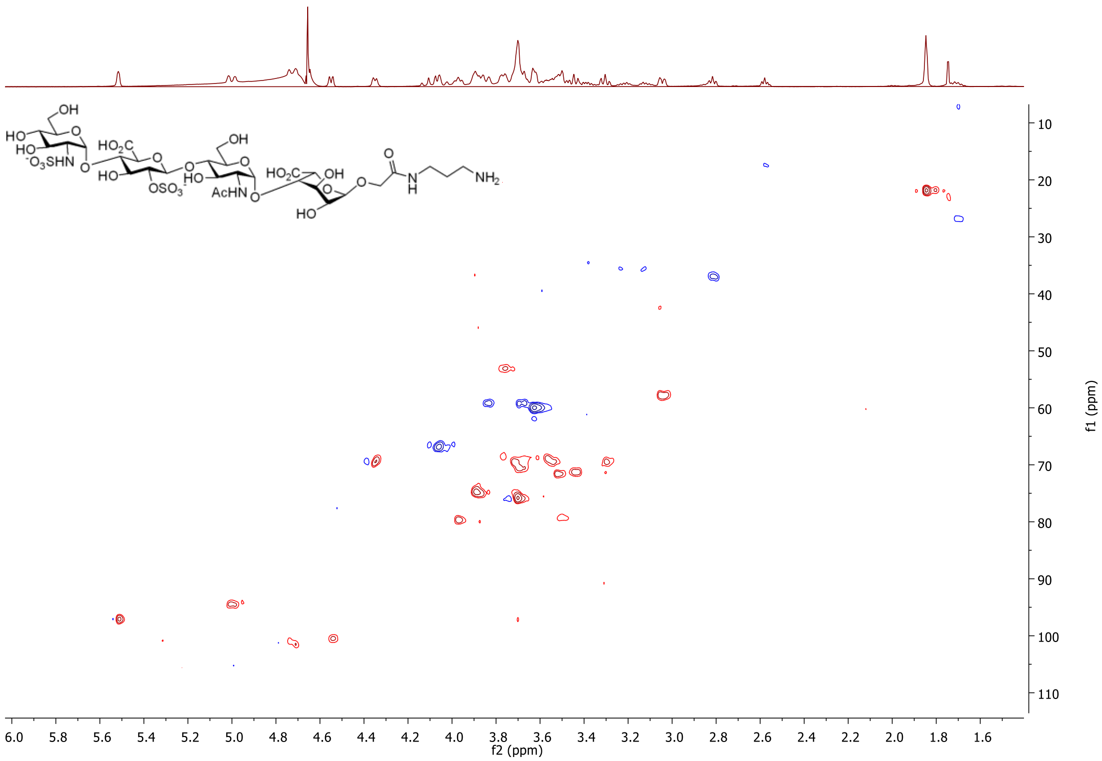

^{1}H-decoupled HSQC of compound **72**

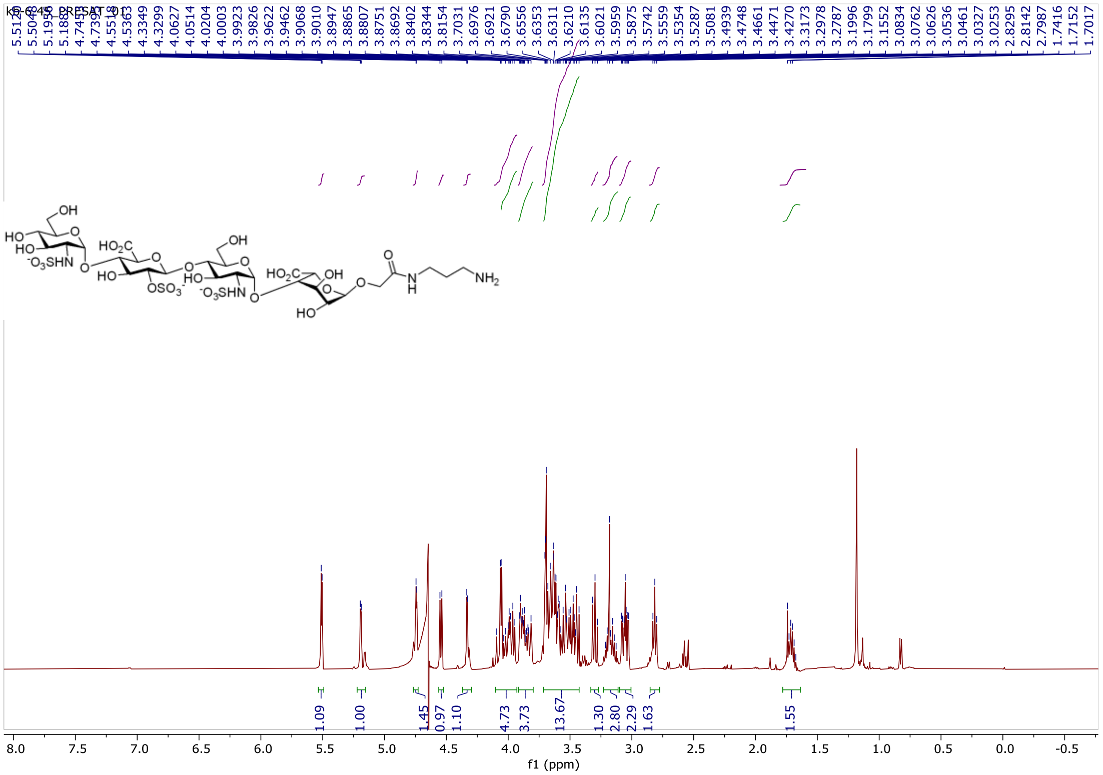

^{1}HNMR spectrum of compound **73**

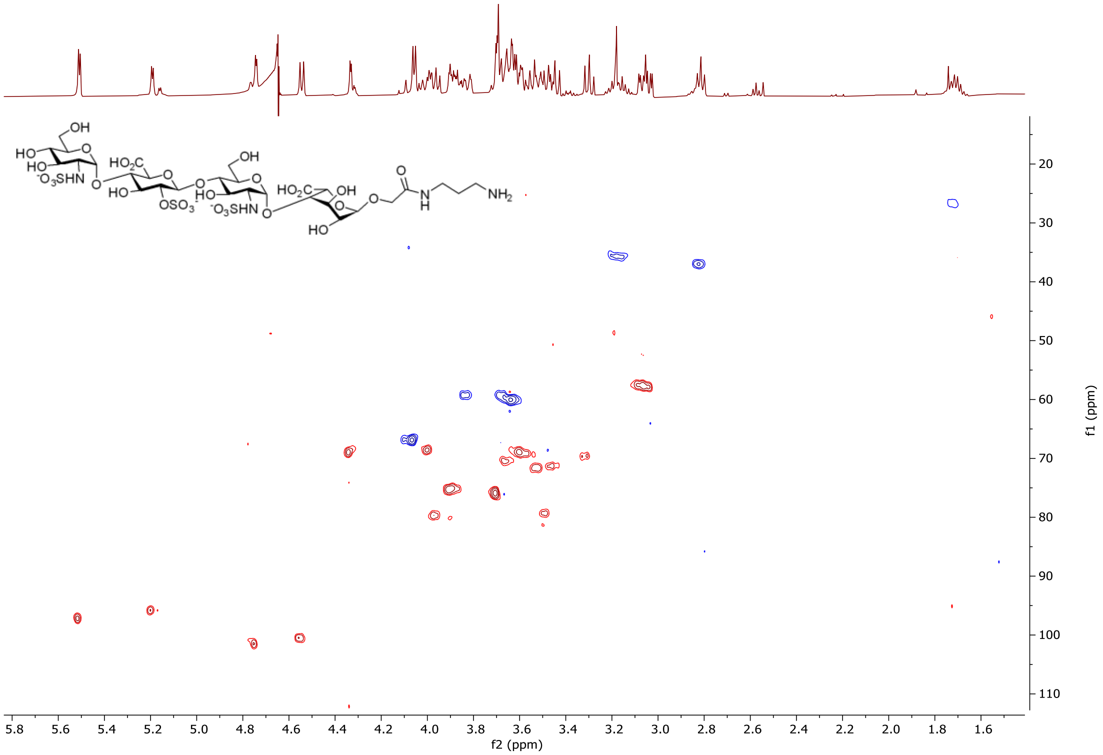

^{1}H-decoupled HSQC of compound **73**

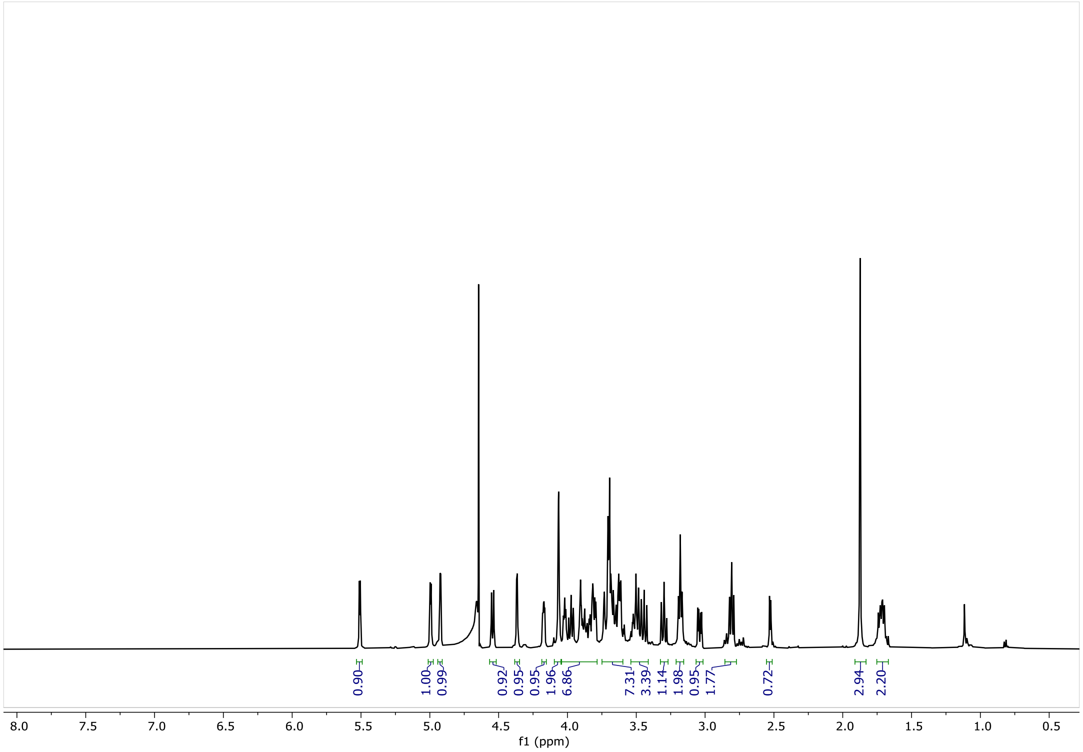

^{1}H NMR spectrum of compound **74**

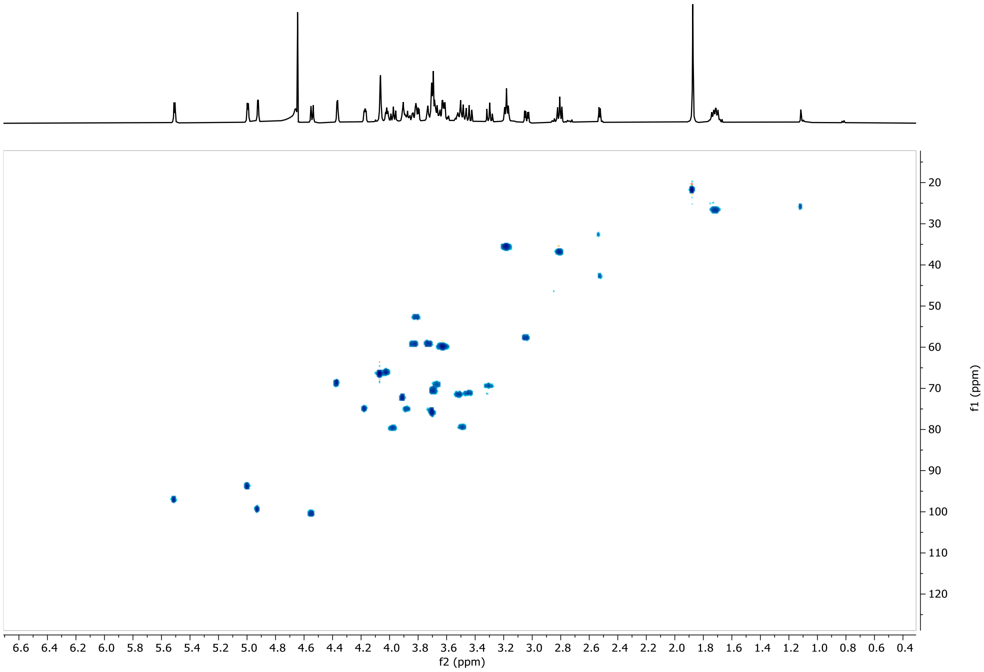

^{1}H-decoupled gHSQC NMR spectrum of compound **74**

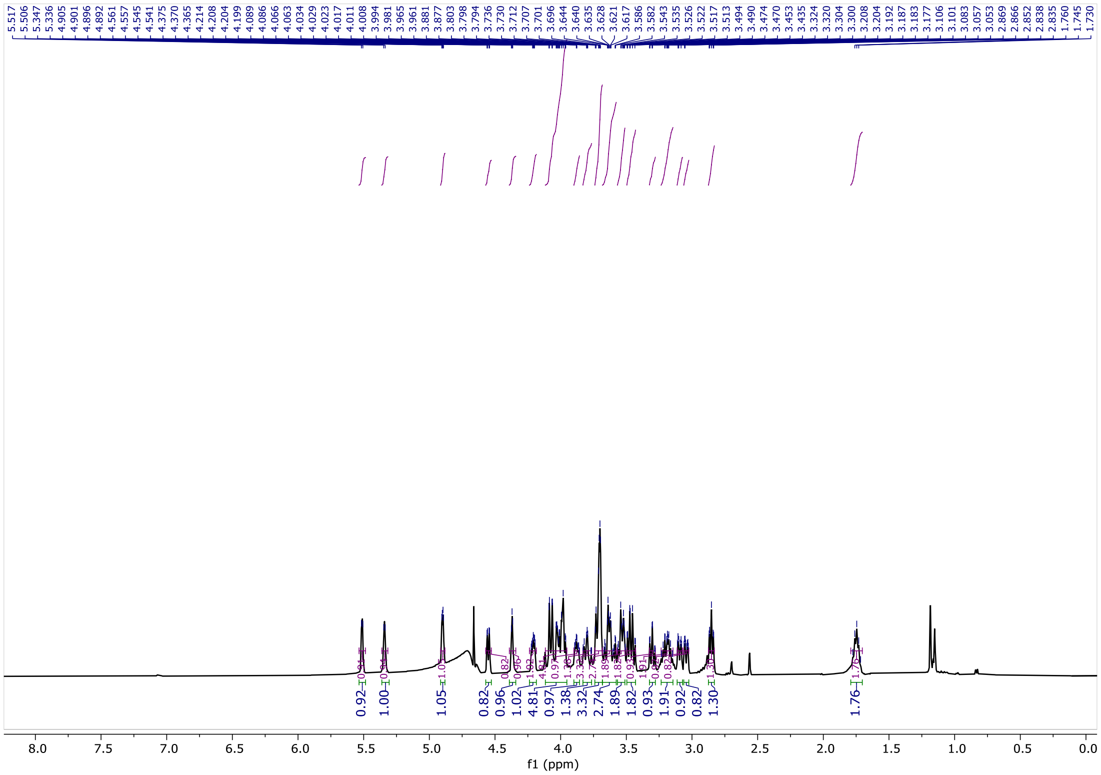

^{1}H NMR spectrum of compound **75**

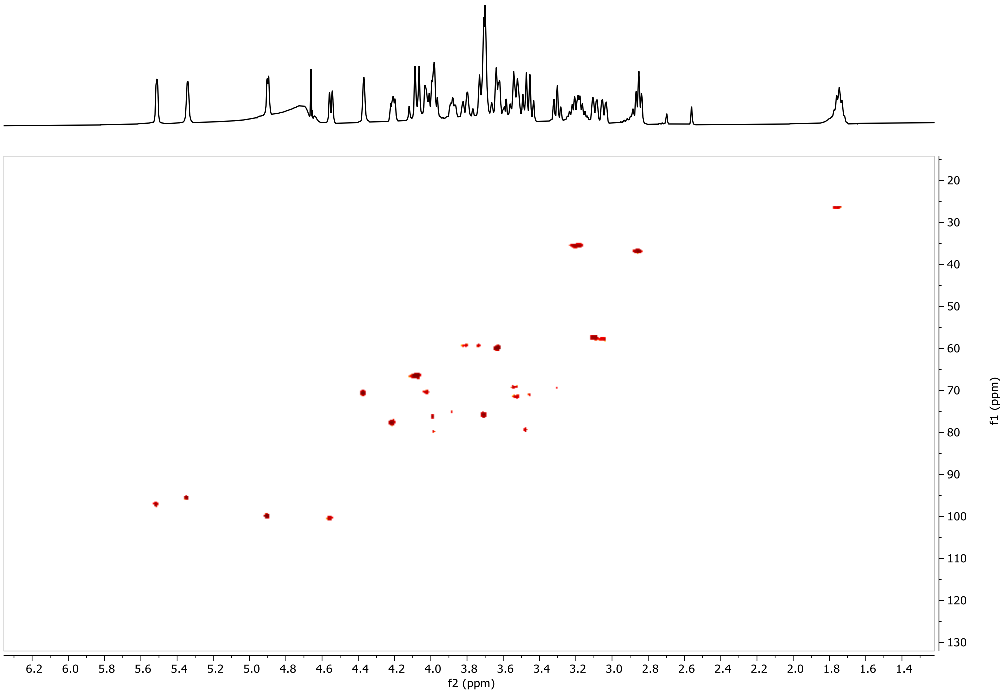

^{1}H-decoupled gHSQC NMR spectrum of compound **75**

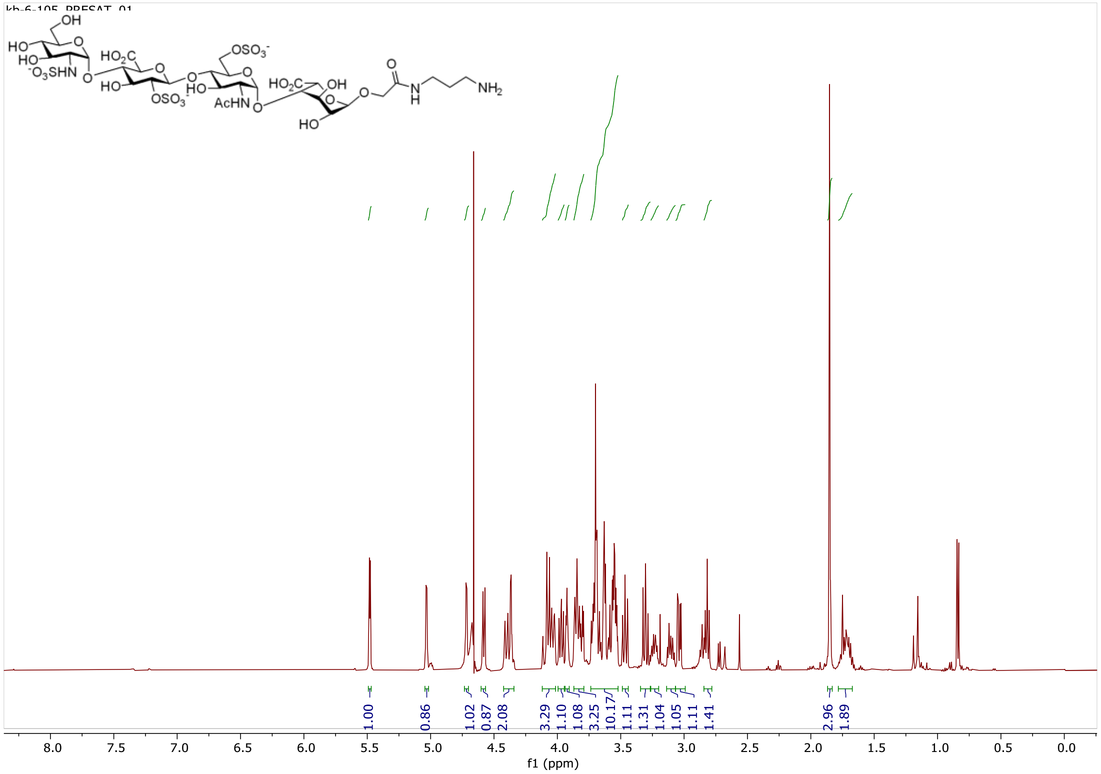

^{1}HNMR spectrum of compound **76**

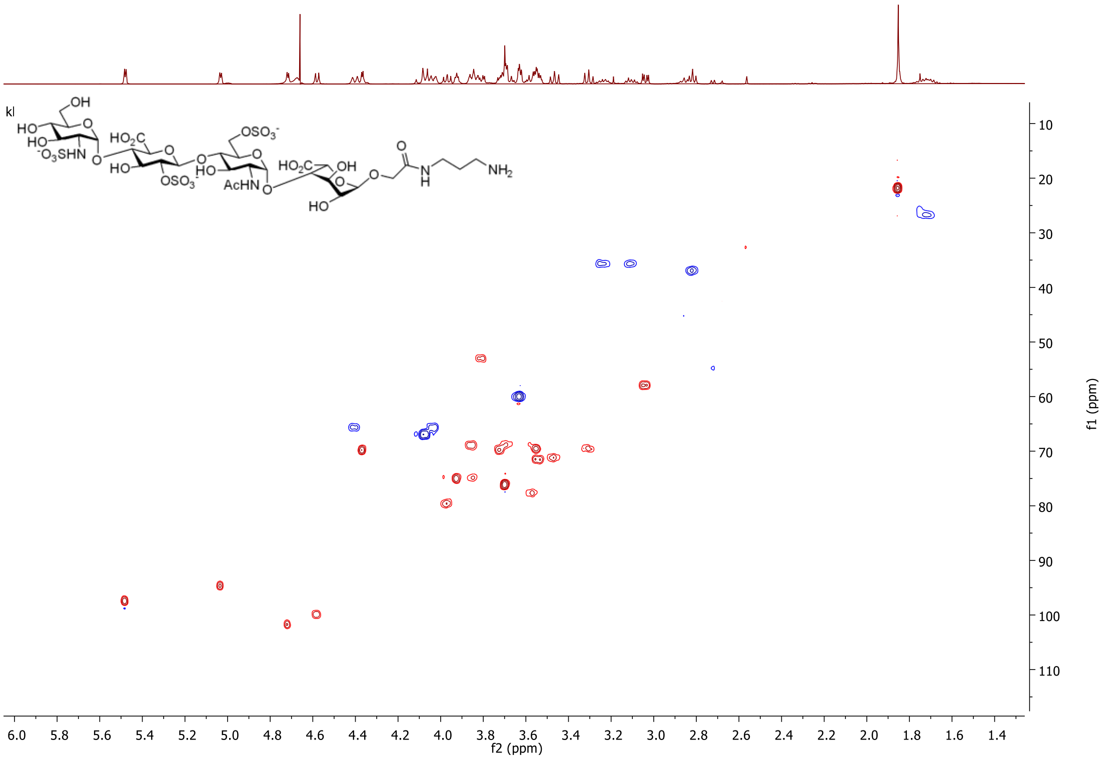

^{1}H-decoupled HSQC of compound **76**

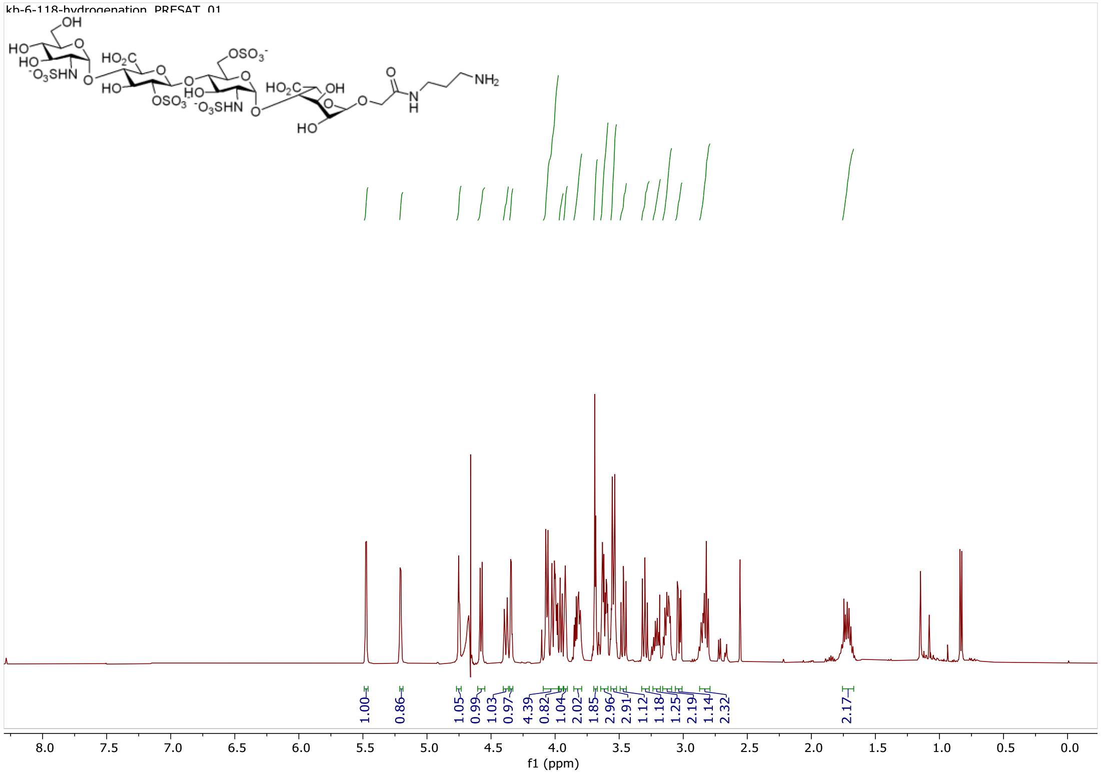

^{1}HNMR spectrum of compound **77**

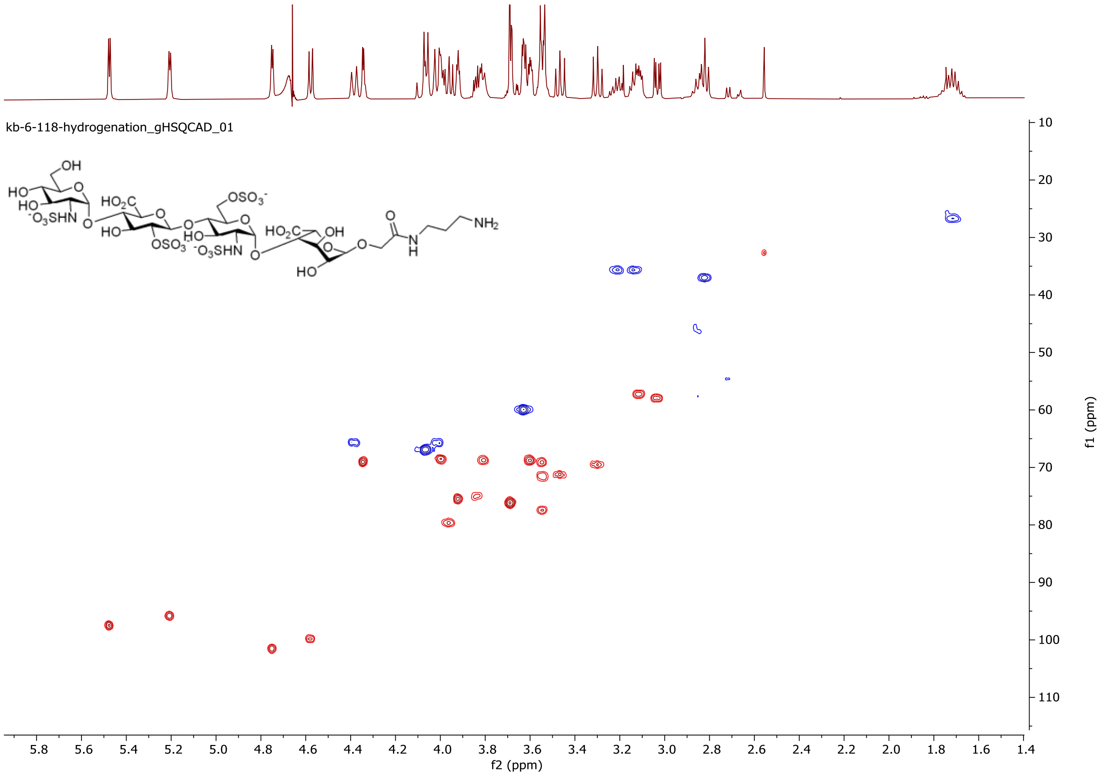

^{1}H-decoupled HSQC of compound **77**

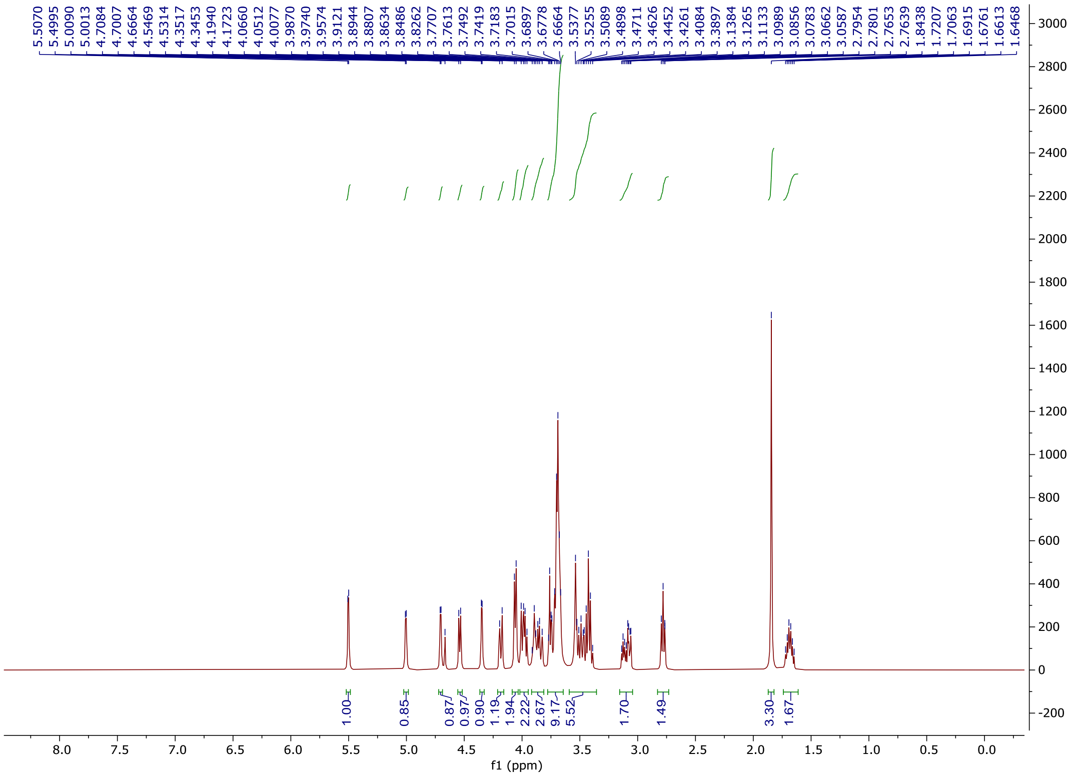

^{1}H NMR spectrum of compound **78**

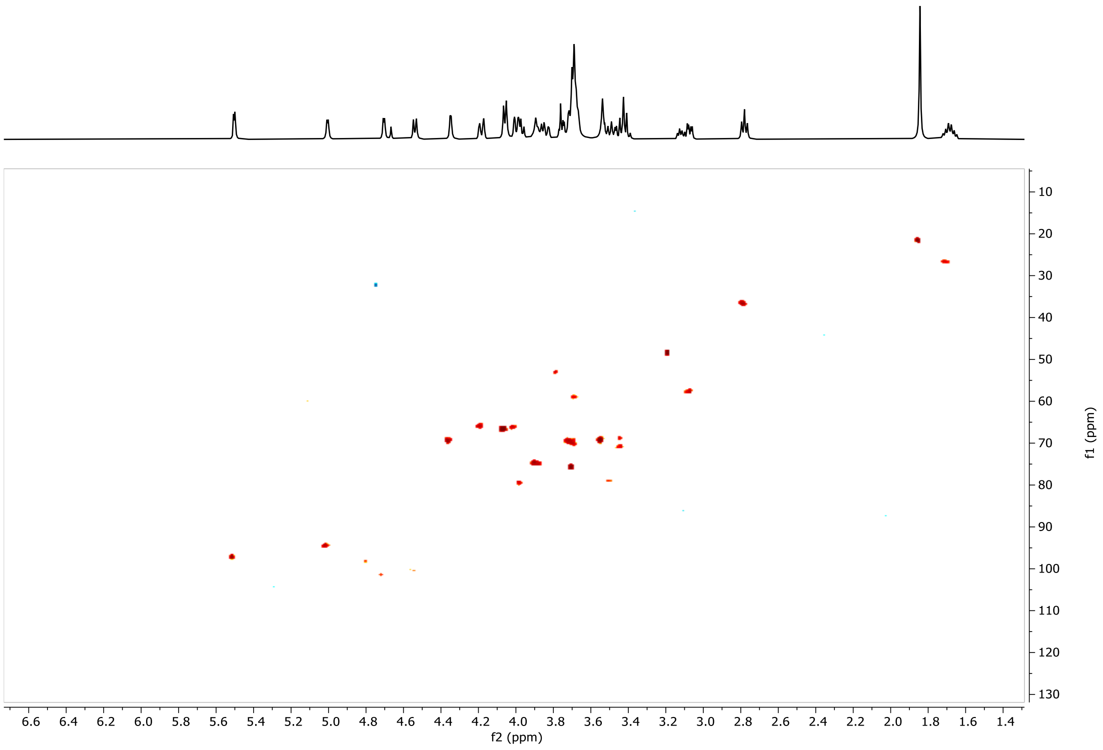

^{1}H-decoupled gHSQC NMR spectrum of compound **78**

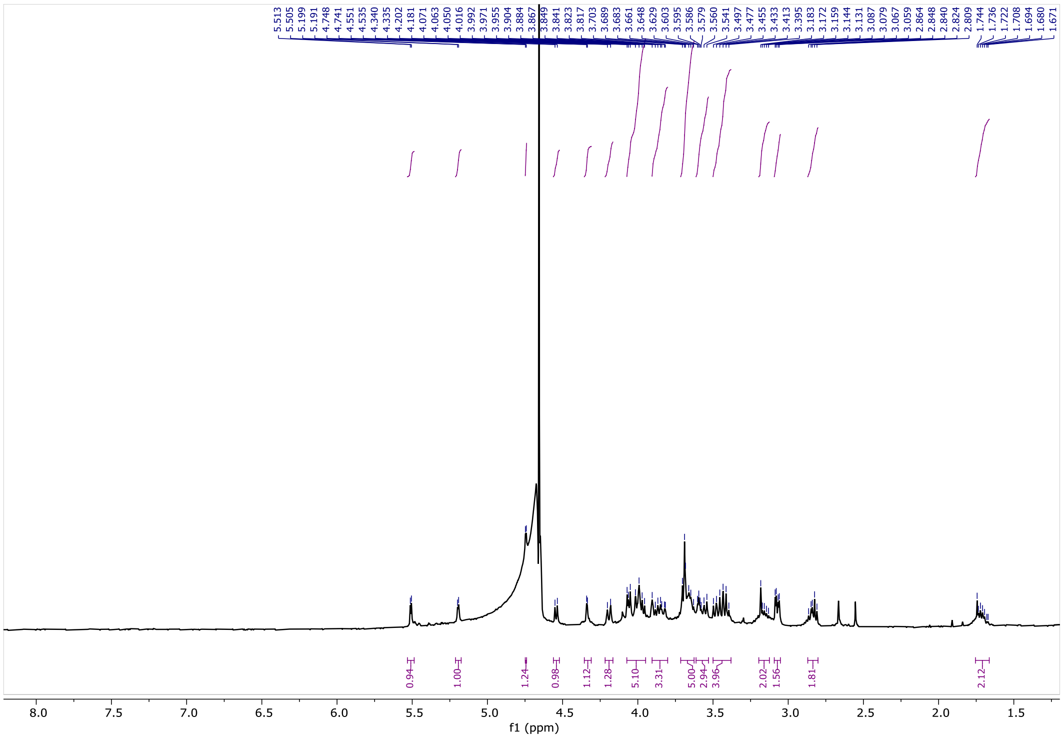

^{1}H NMR spectrum of compound **79**

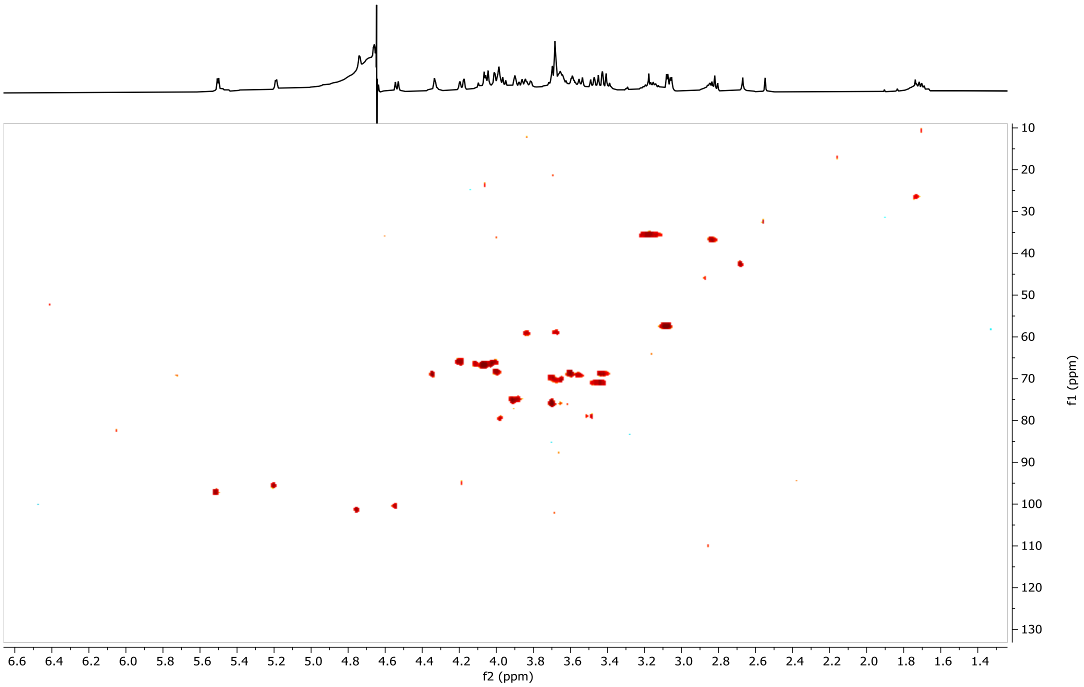

^{1}H-decoupled gHSQC NMR spectrum of compound **79**

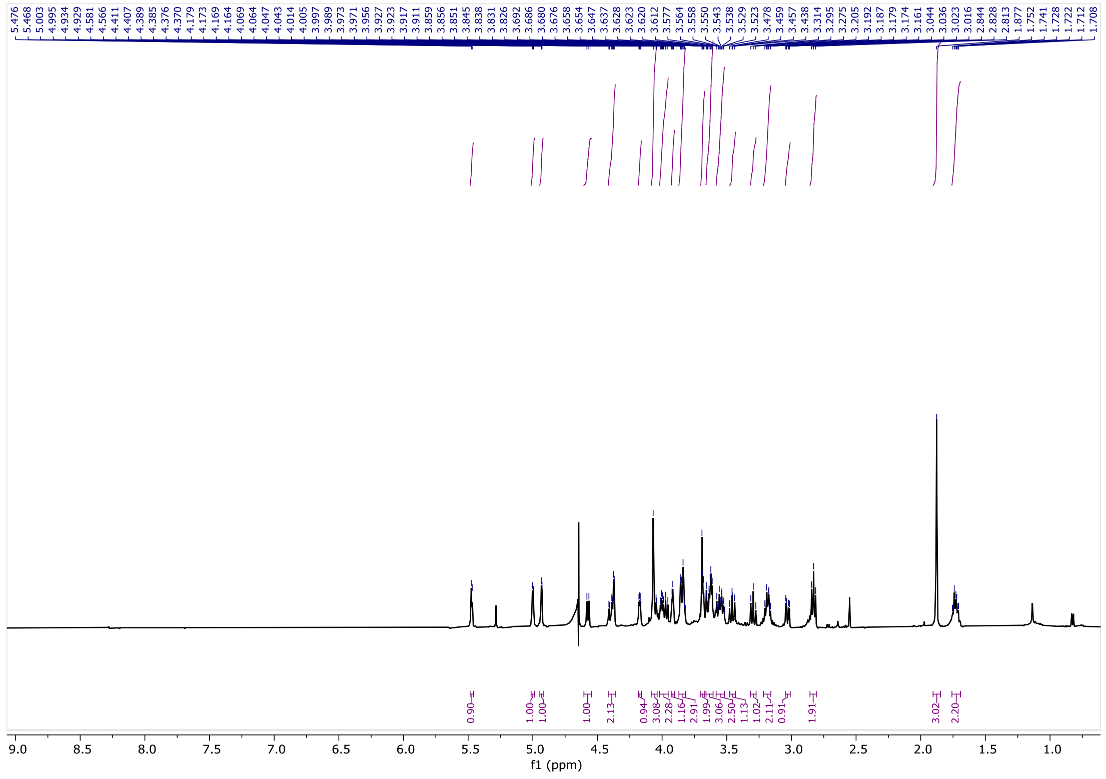

^{1}H NMR spectrum of compound **80**

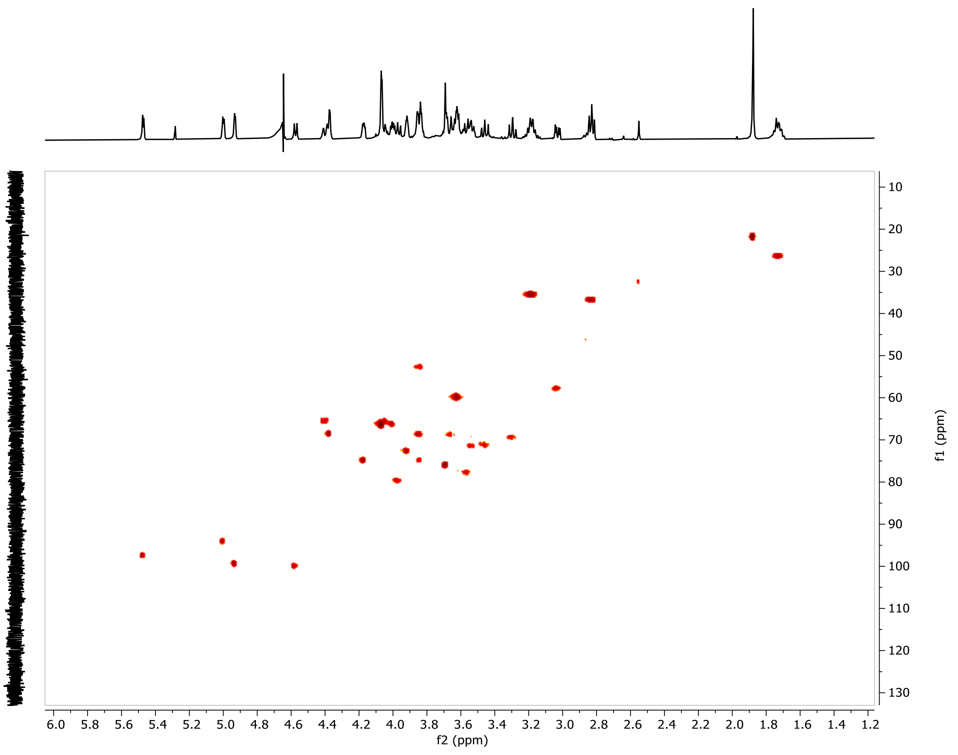

^{1}H-decoupled gHSQC NMR spectrum of compound **80**

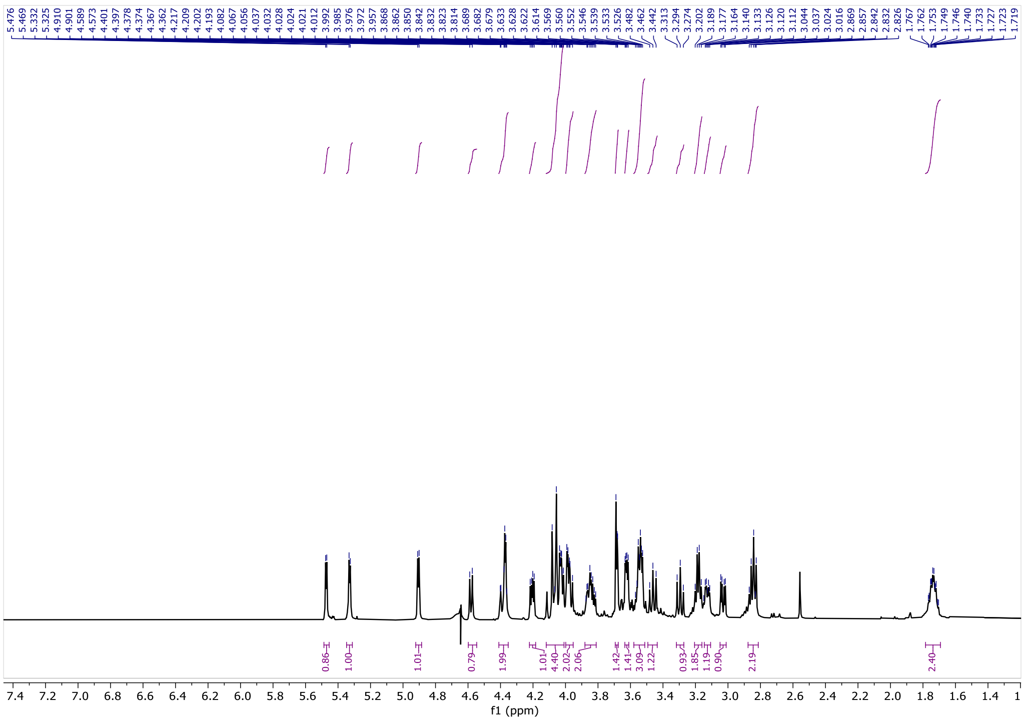

^{1}H NMR spectrum of compound **81**

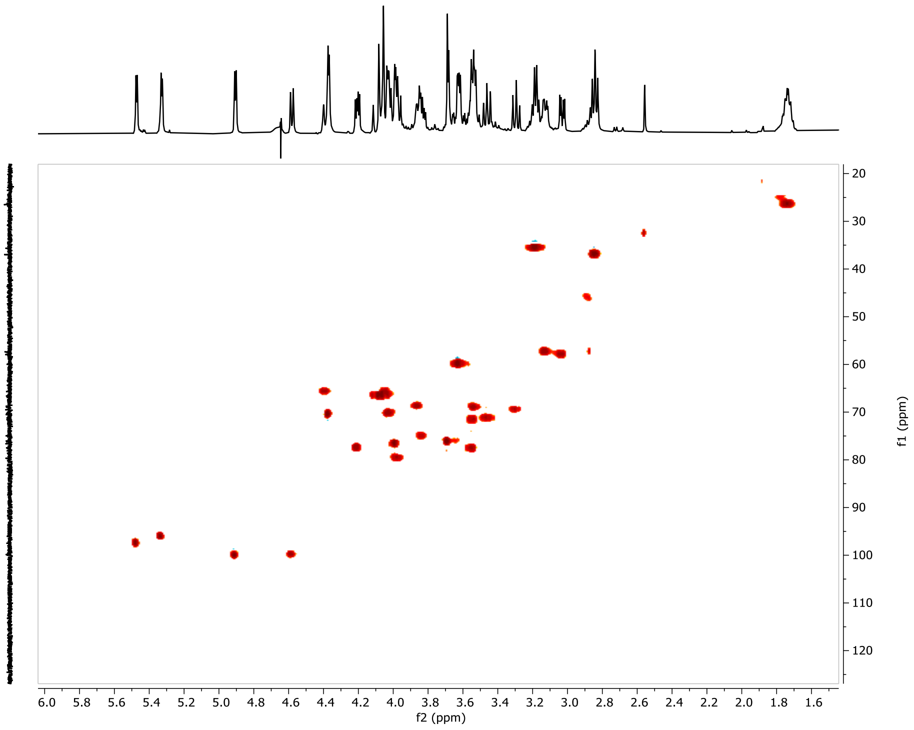

^{1}H-decoupled gHSQC NMR spectrum of compound **81**

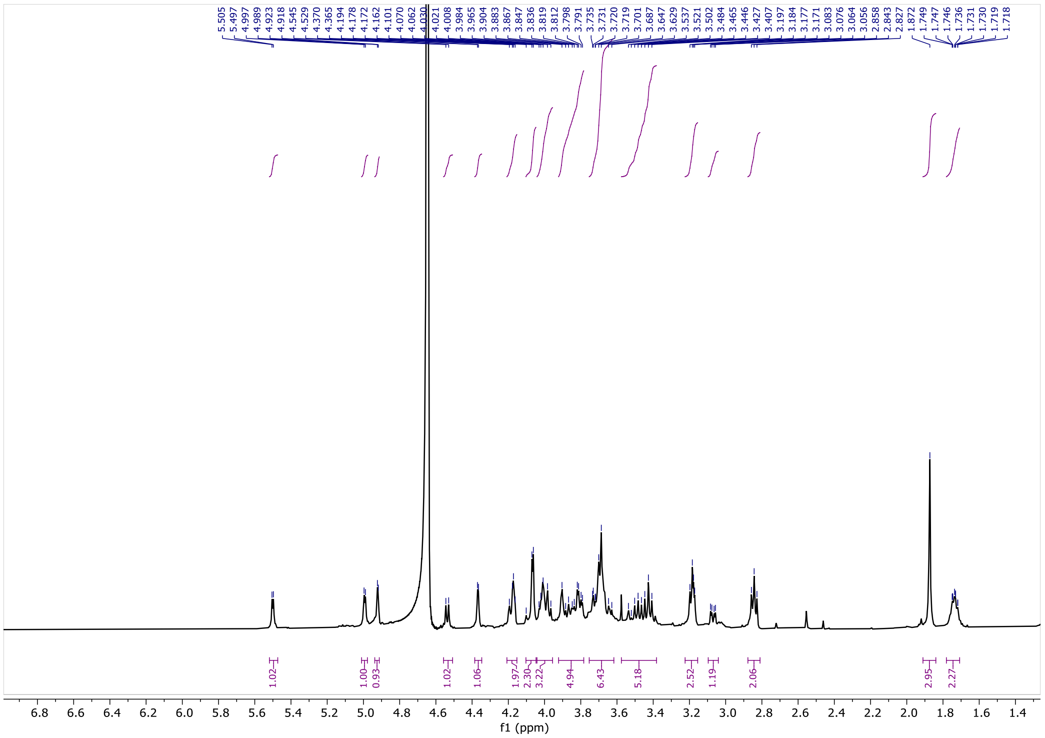

^{1}H NMR spectrum of compound **82**

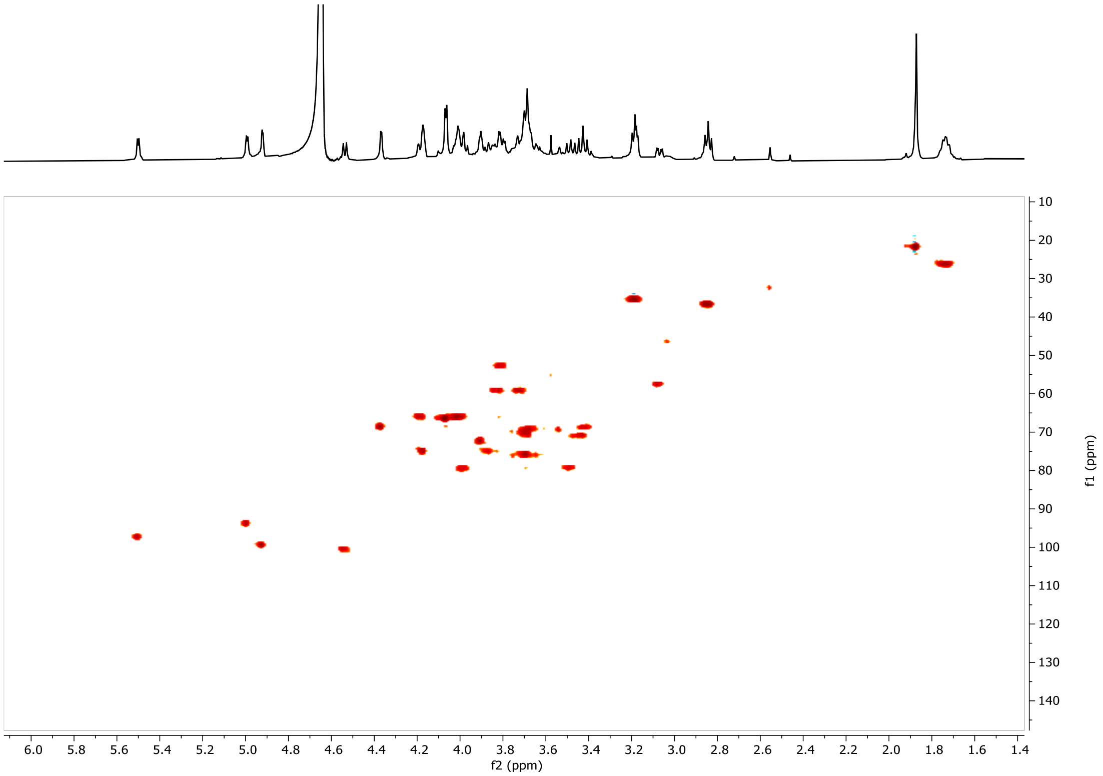

^{1}H-decoupled gHSQC NMR spectrum of compound **82**

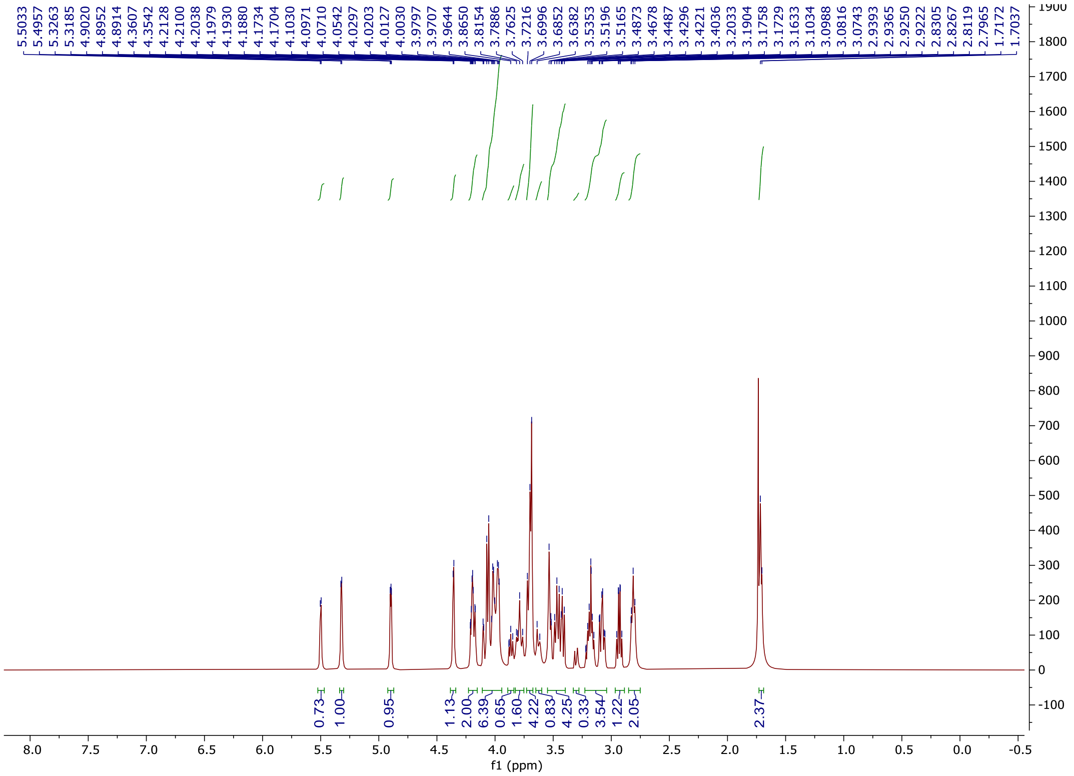

^{1}H NMR spectrum of compound **83**

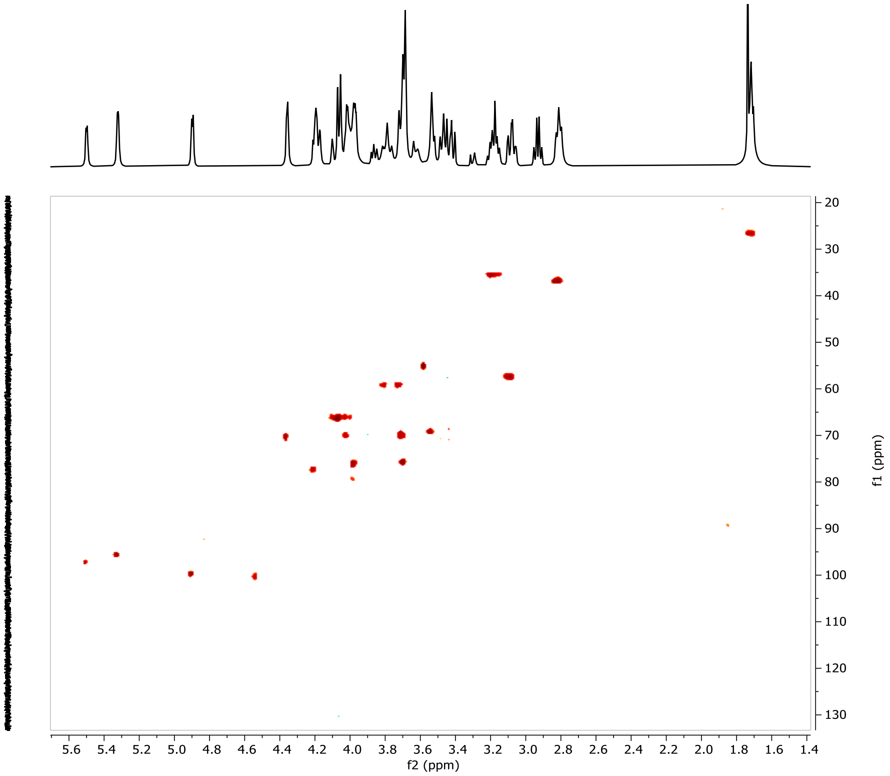

^{1}H-decoupled gHSQC NMR spectrum of compound **83**

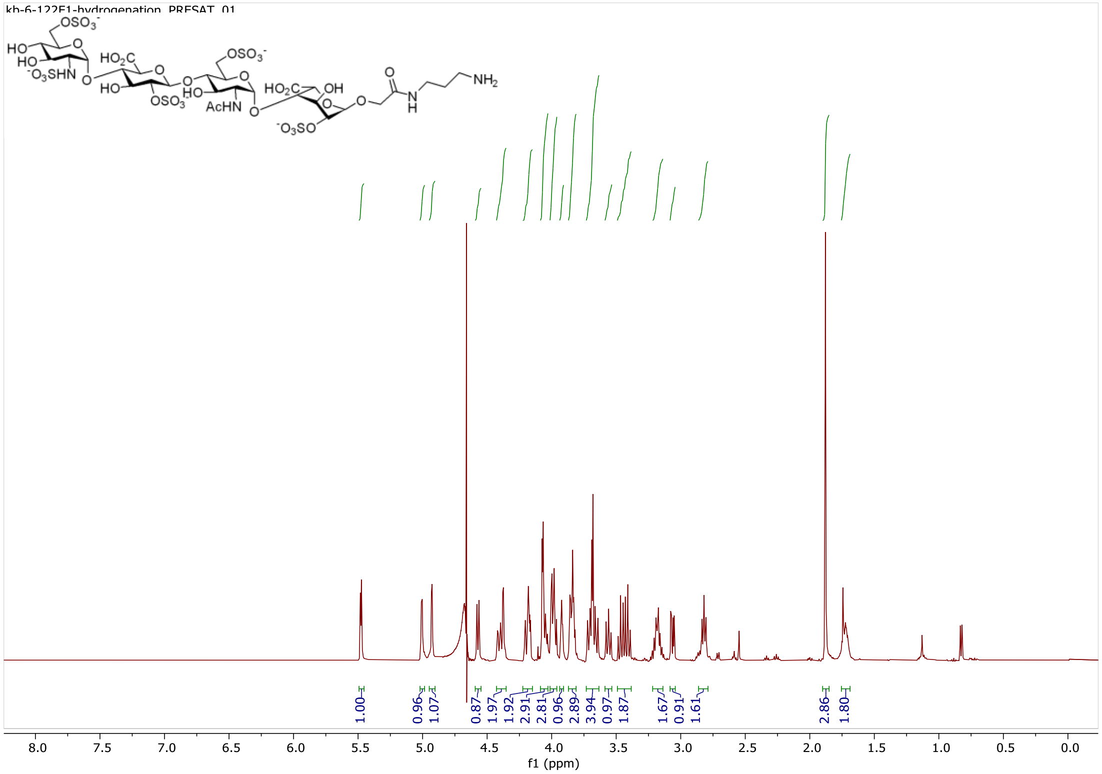

^{1}HNMR spectrum of compound **84**

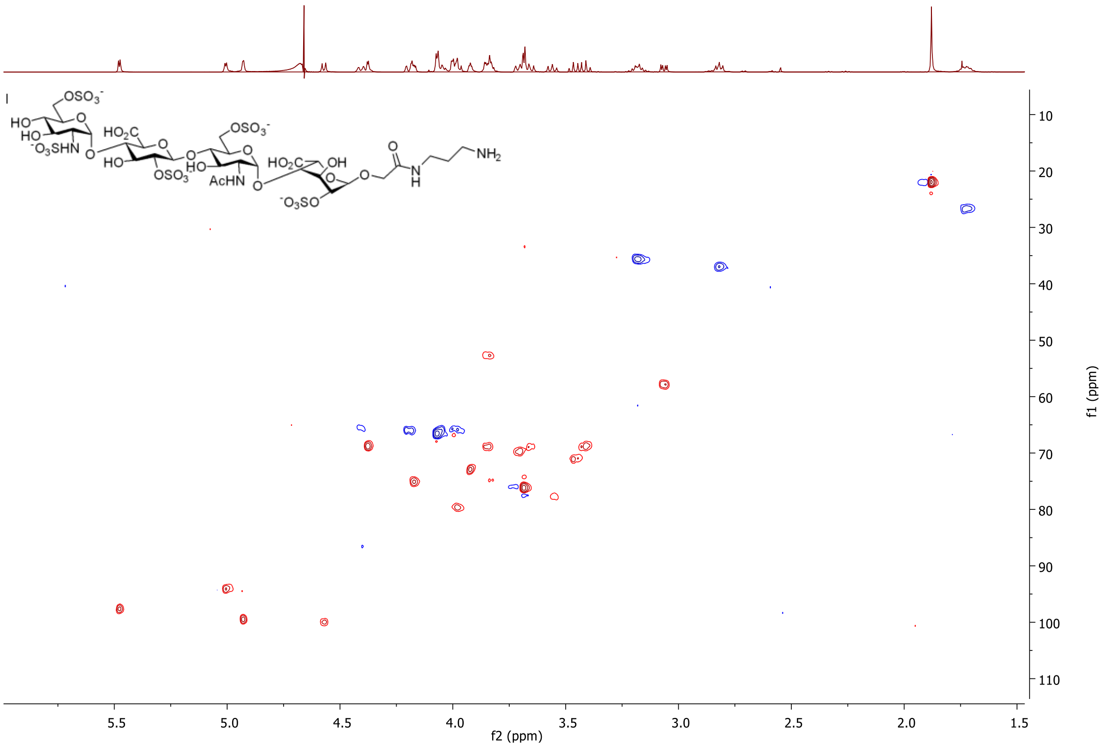

^{1}H-decoupled HSQC of compound **84**

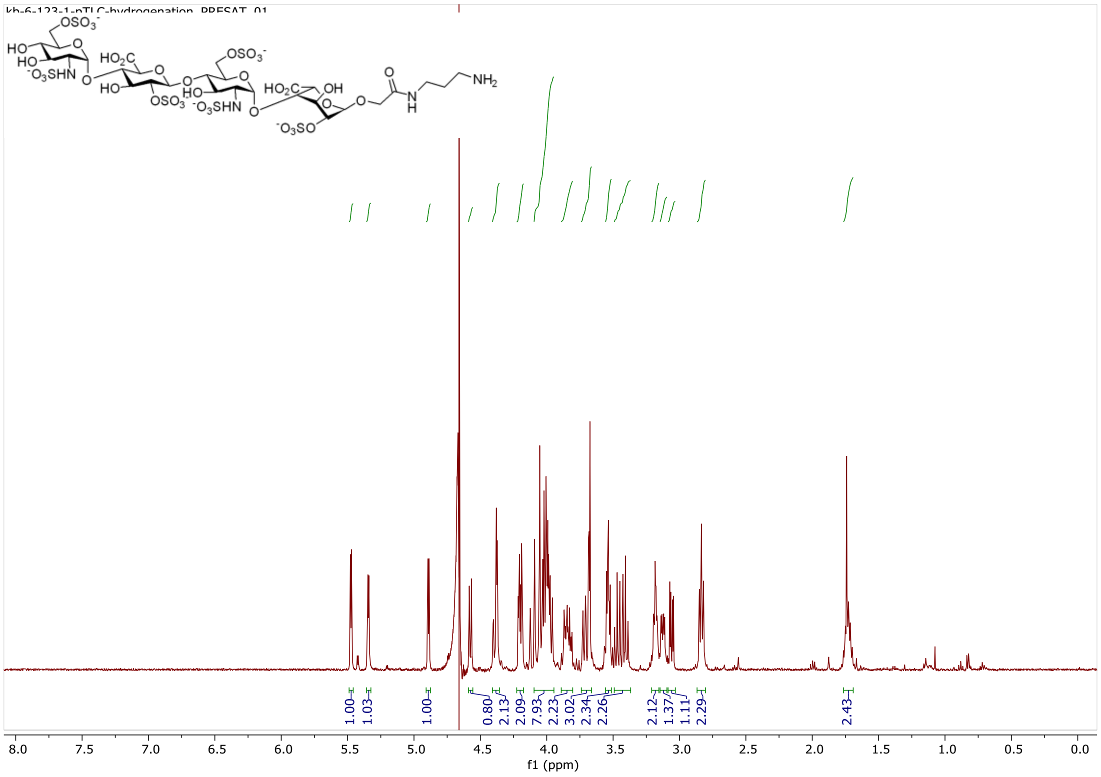

^{1}HNMR spectrum of compound **85**

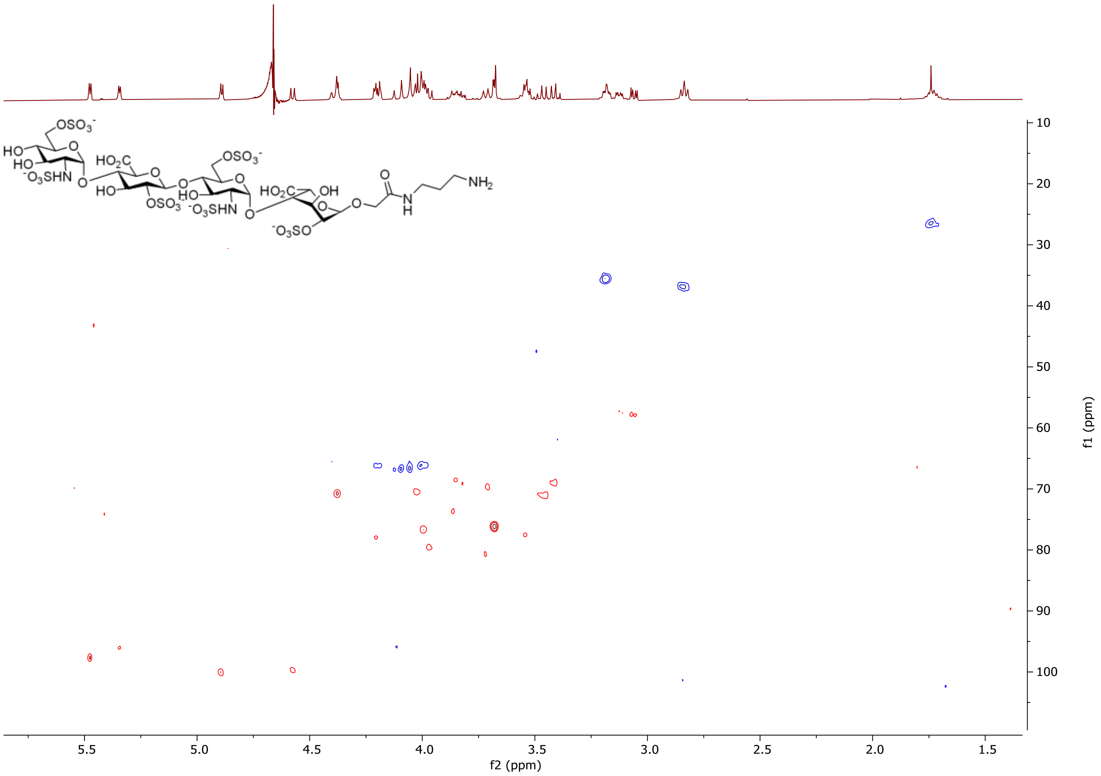

^{1}H-decoupled HSQC of compound **85**

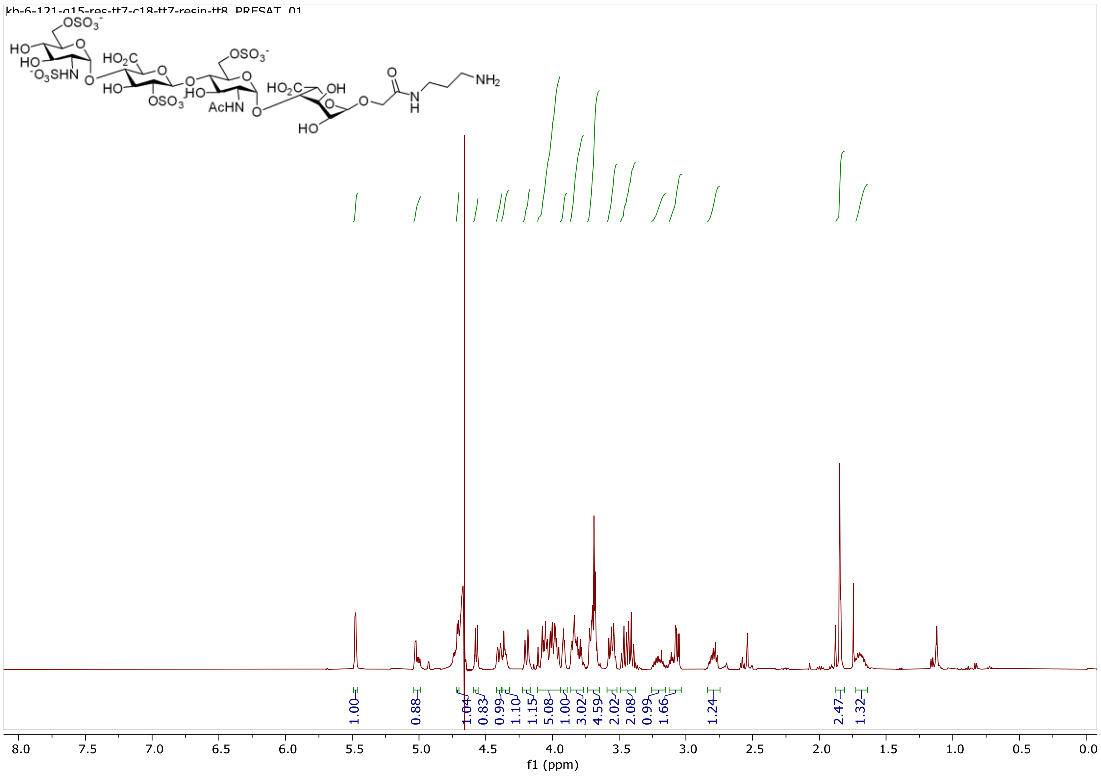

^{1}HNMR spectrum of compound **86**

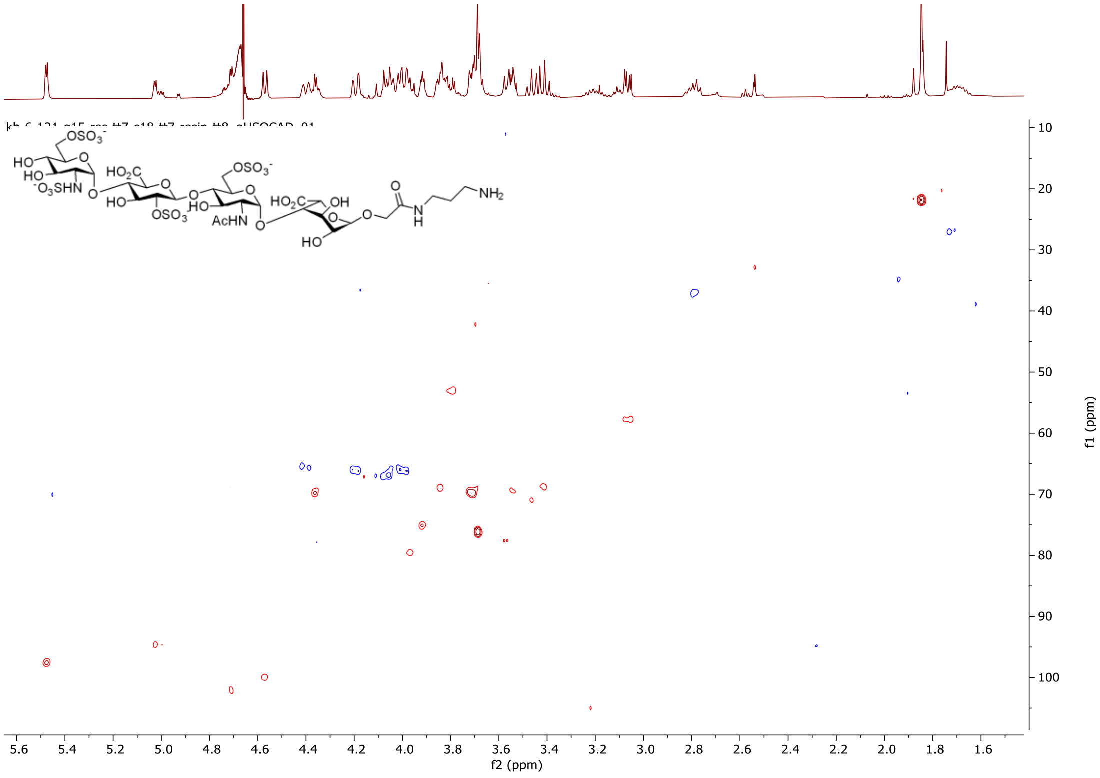

^{1}H-decoupled HSQC of compound **86**

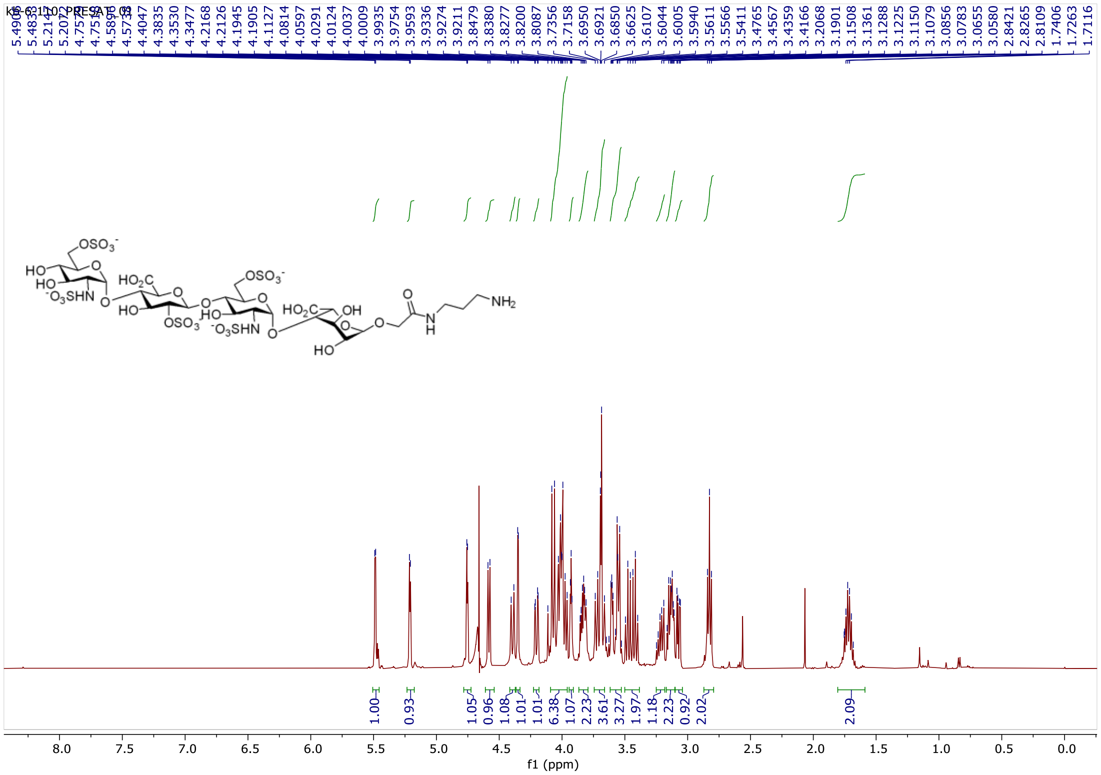

^{1}HNMR spectrum of compound **87**

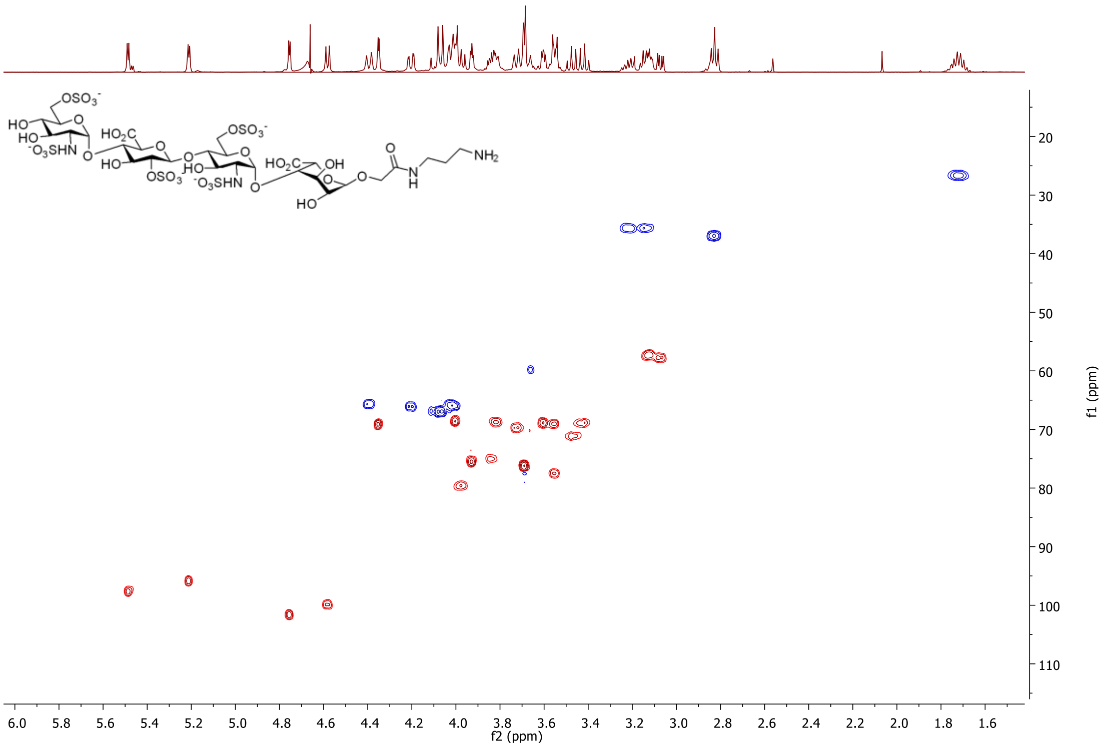

^{1}H-decoupled HSQC of compound **87**

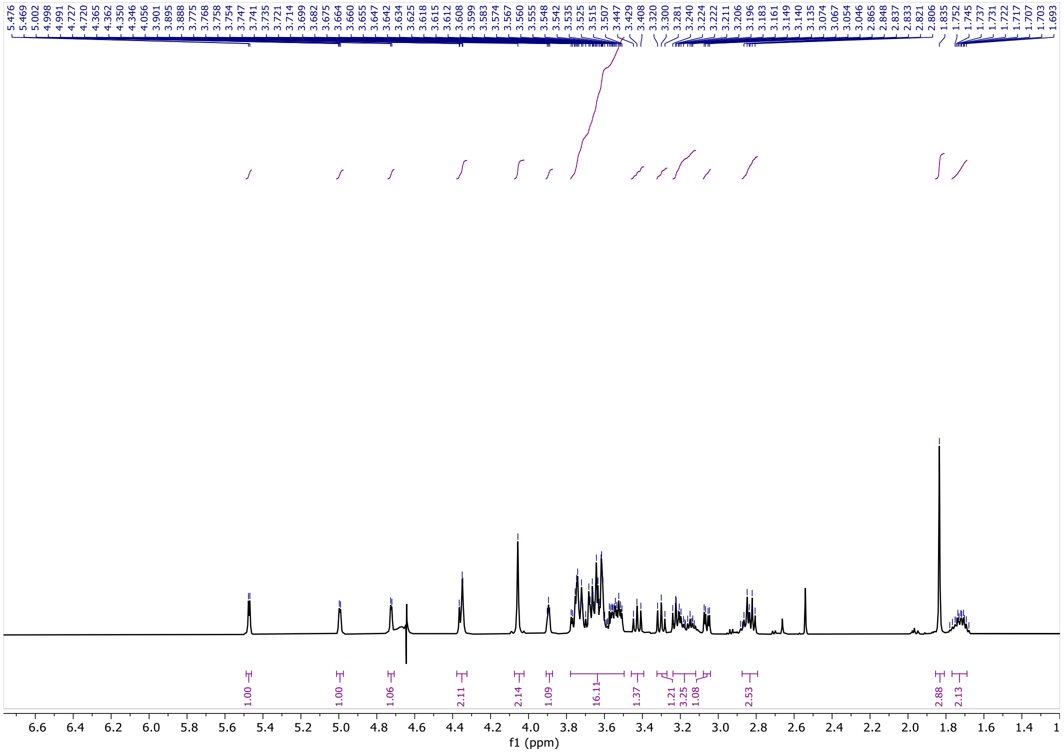

^{1}H NMR spectrum of compound **88**

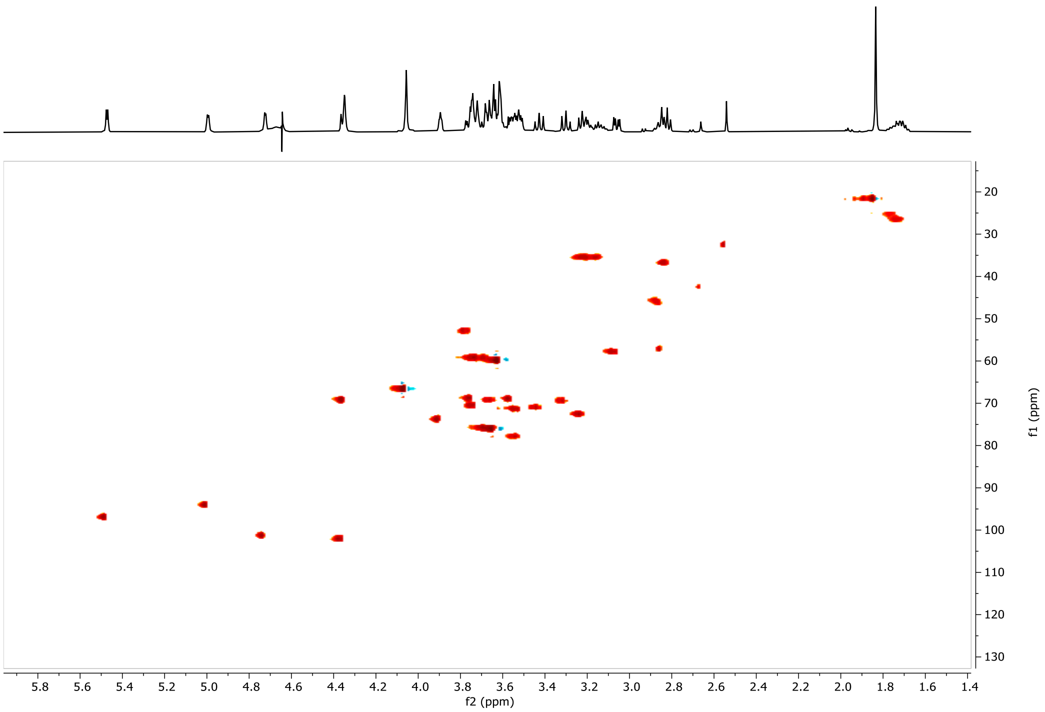

^{1}H-decoupled gHSQC NMR spectrum of compound **88**

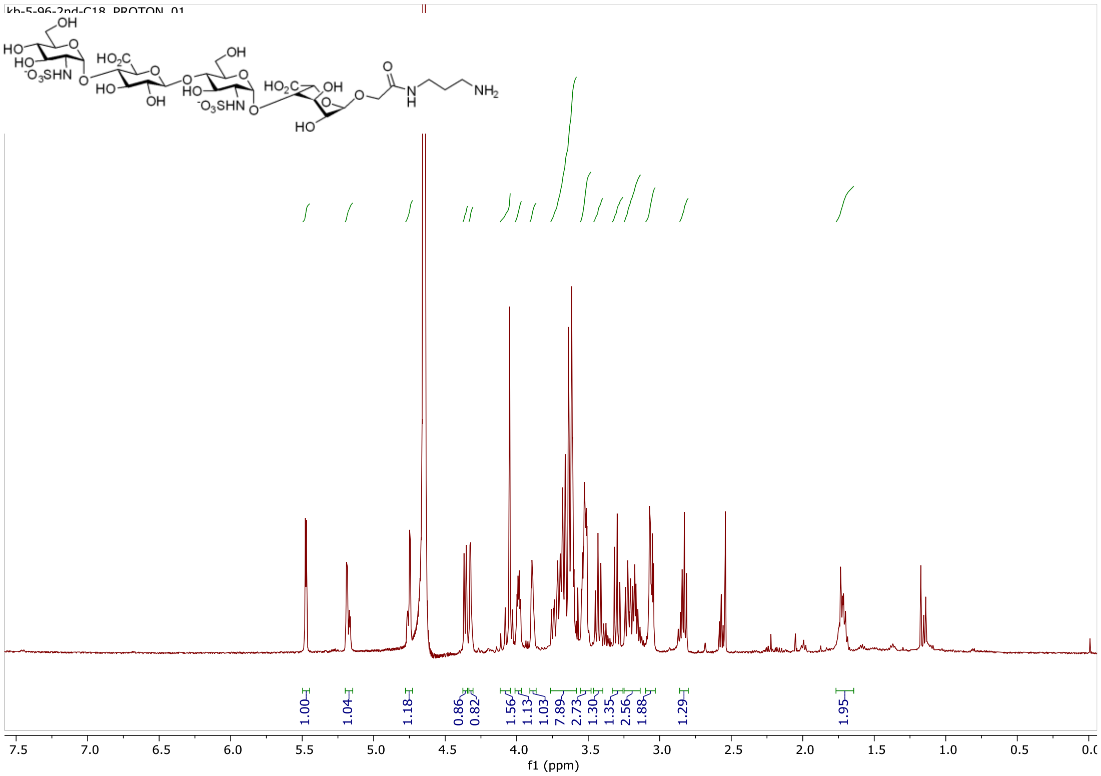

^{1}HNMR spectrum of compound **89**

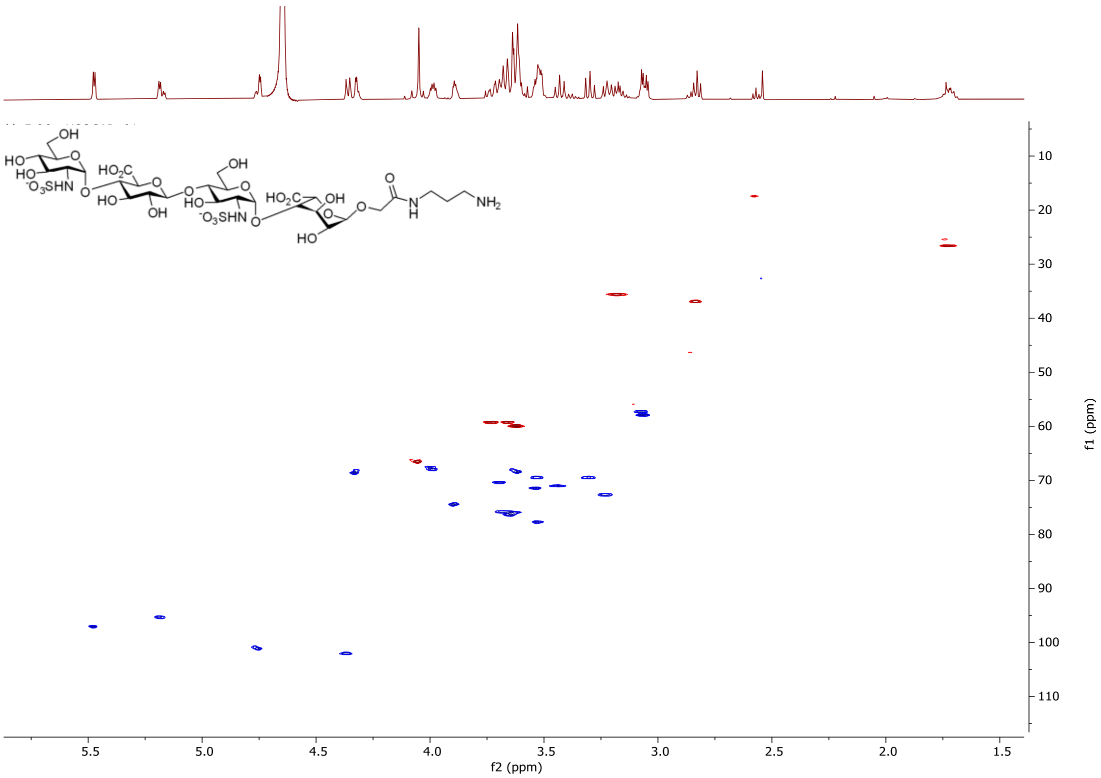

^{1}H-decoupled HSQC of compound **89**

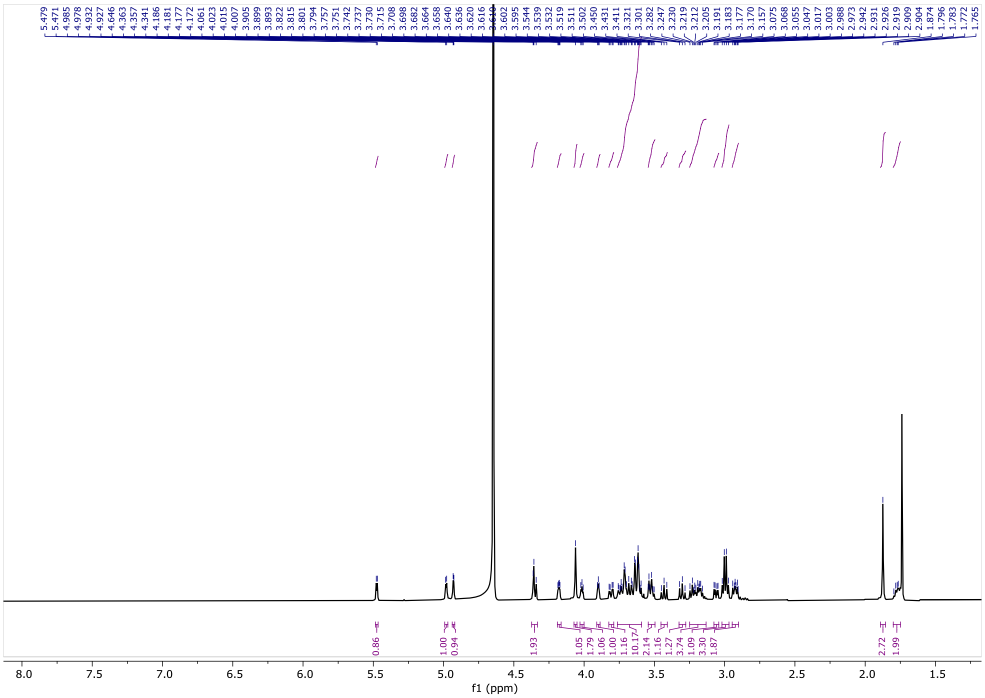

^{1}H NMR spectrum of compound **90**

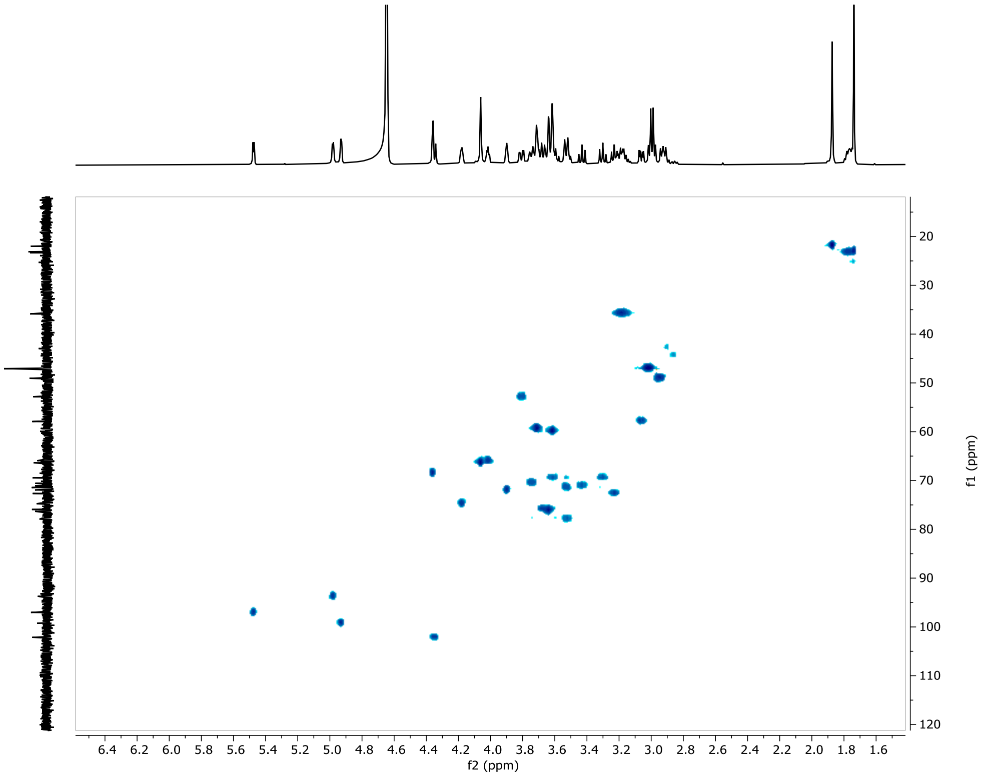

^{1}H-decoupled gHSQC NMR spectrum of compound **90**

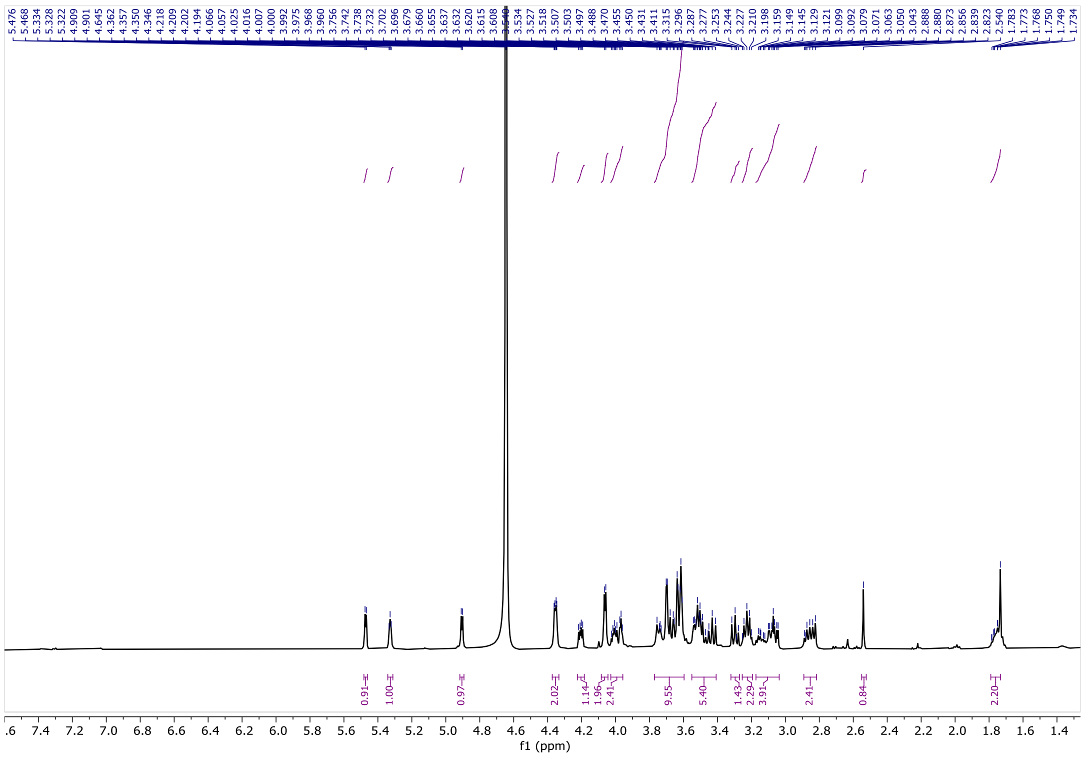

^{1}H NMR spectrum of compound **91**

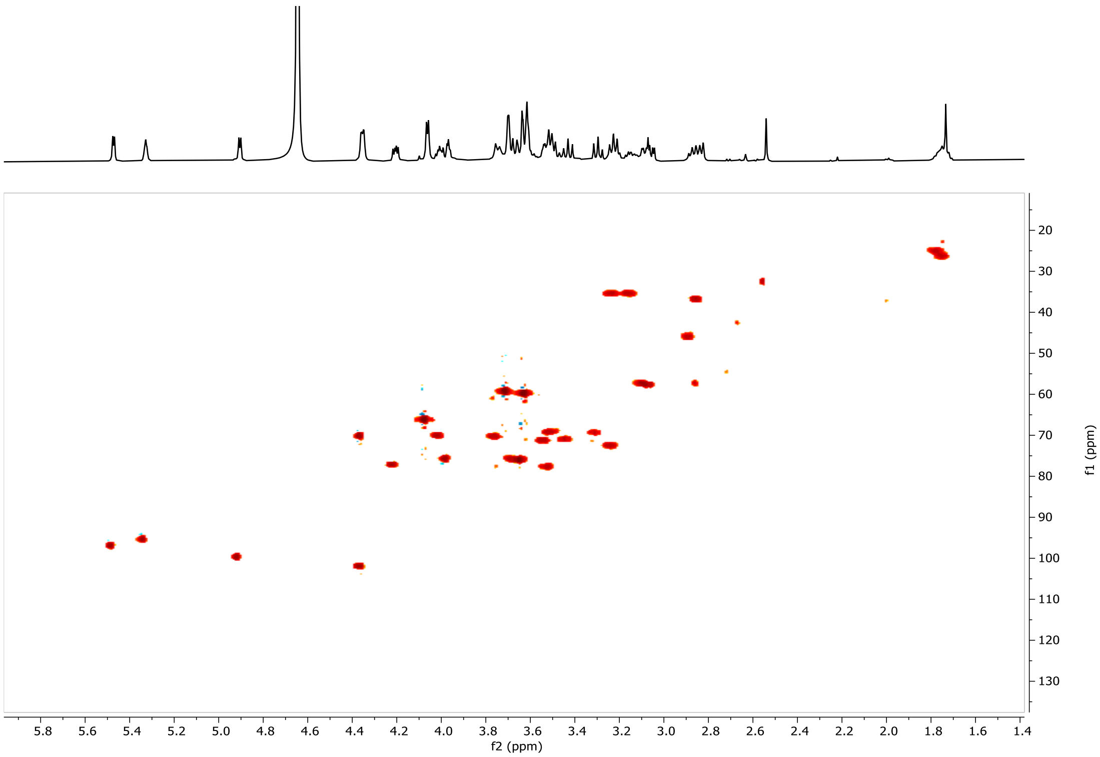

^{1}H-decoupled gHSQC NMR spectrum of compound **91**

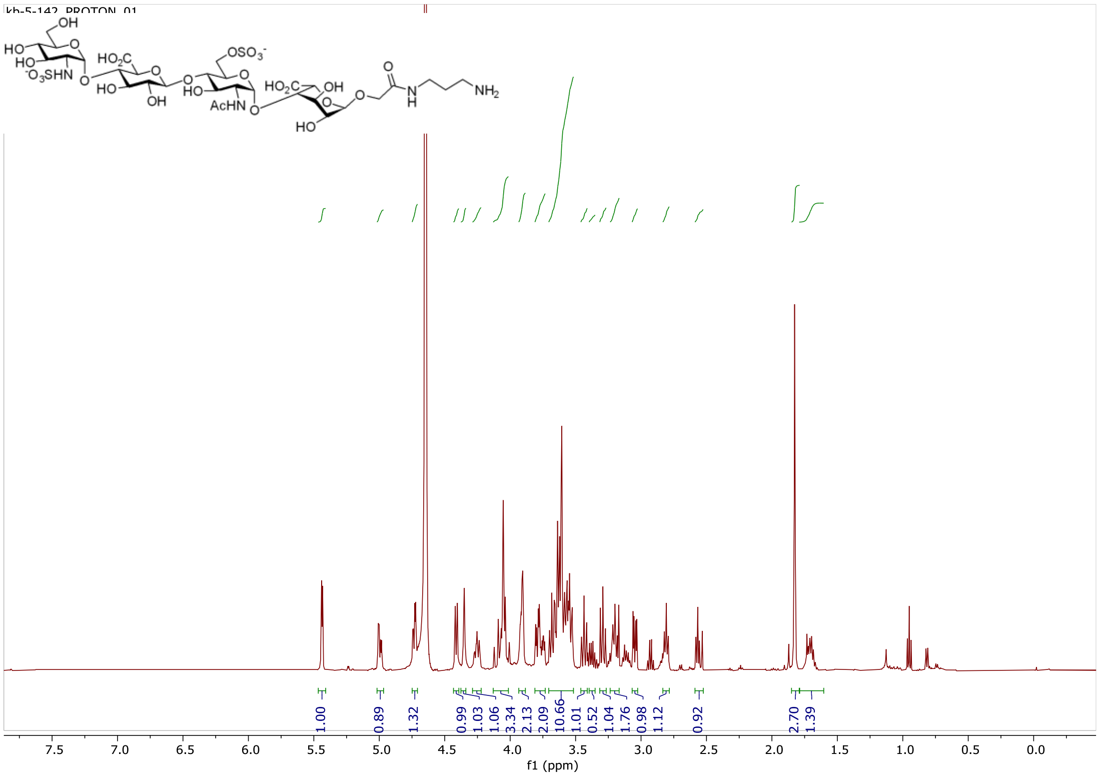

^{1}HNMR spectrum of compound **92**

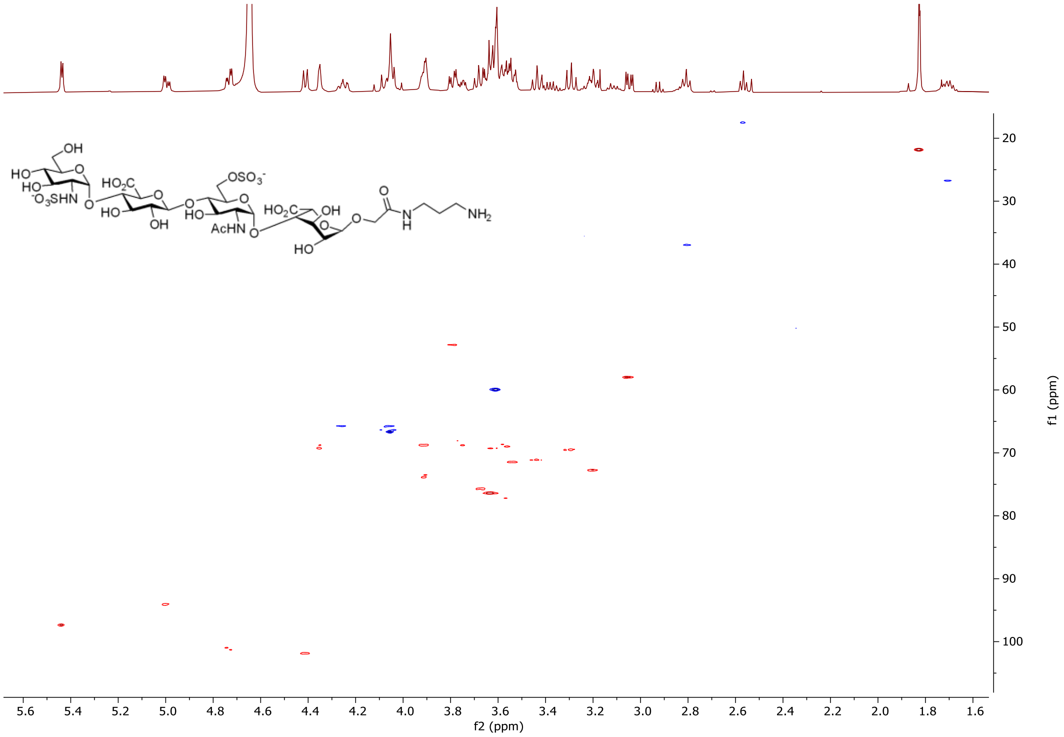

^{1}H-decoupled HSQC of compound **92**

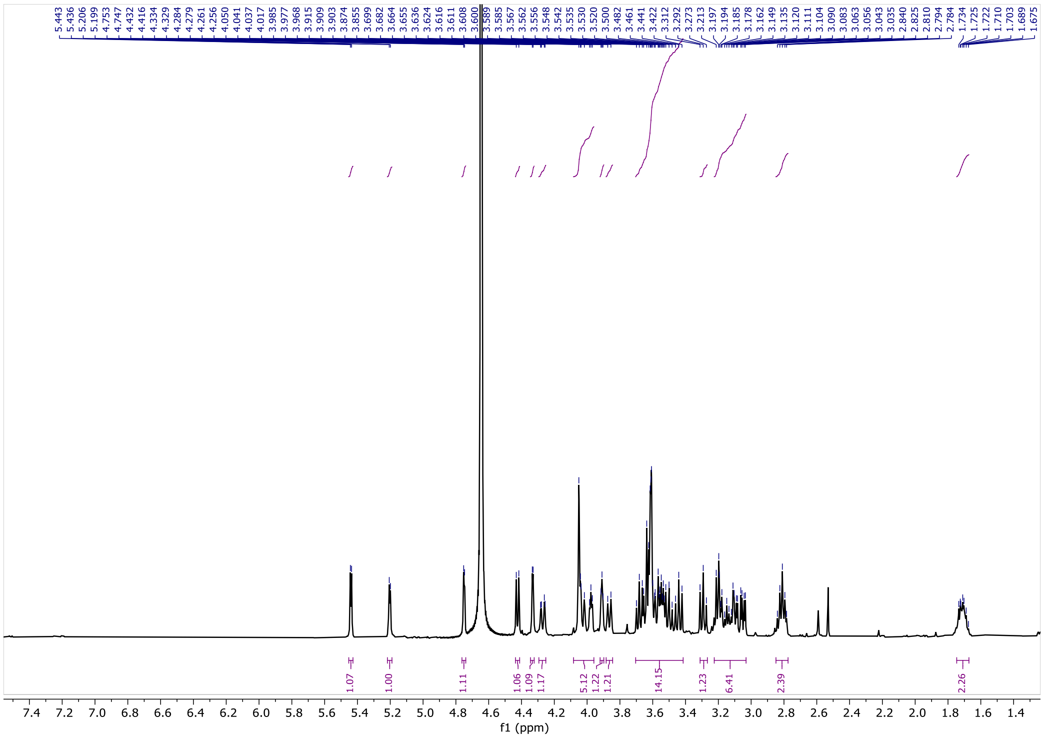

^{1}H NMR spectrum of compound **93**

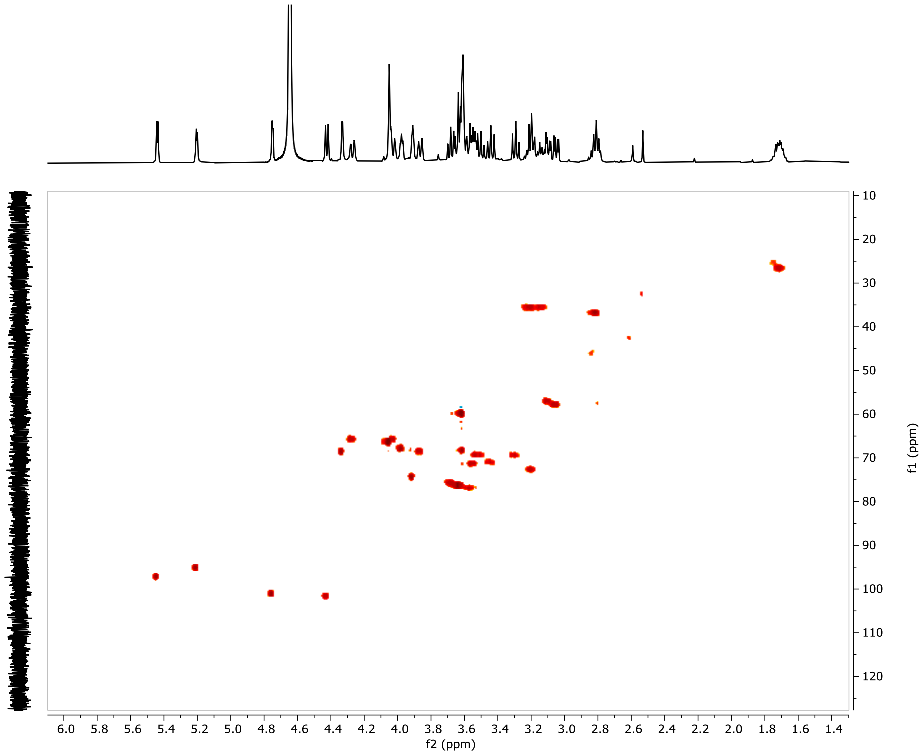

^{1}H-decoupled gHSQC NMR spectrum of compound **93**

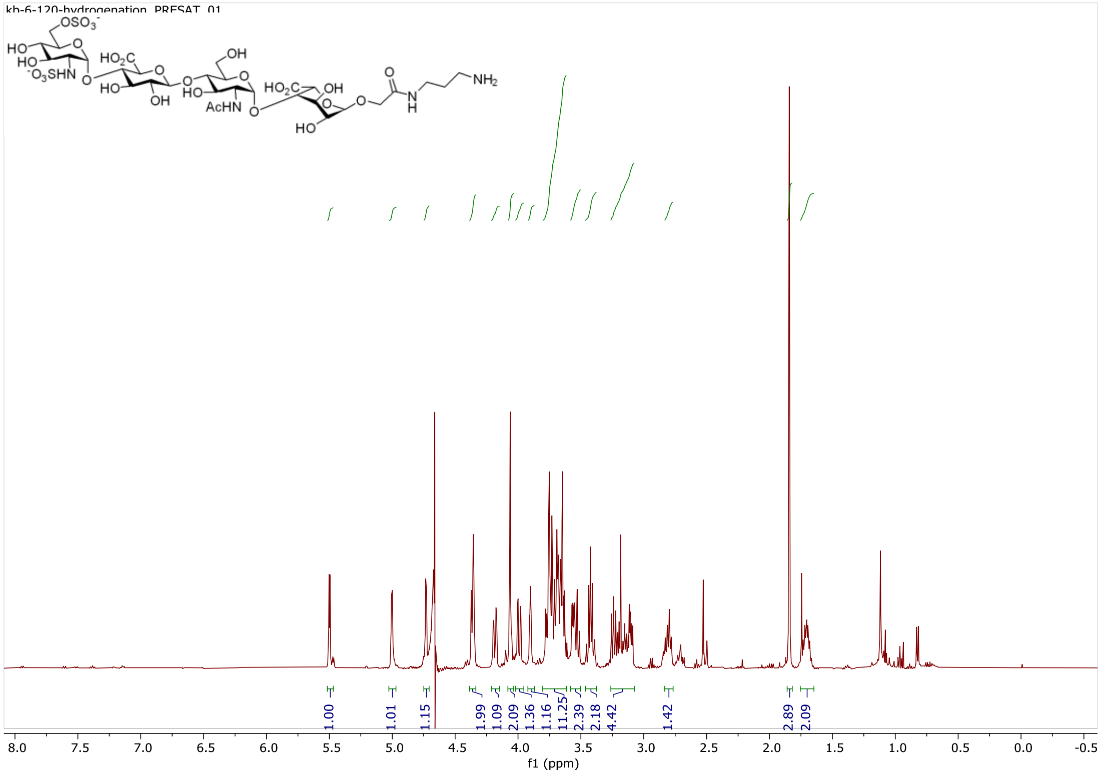

^{1}HNMR spectrum of compound **94**

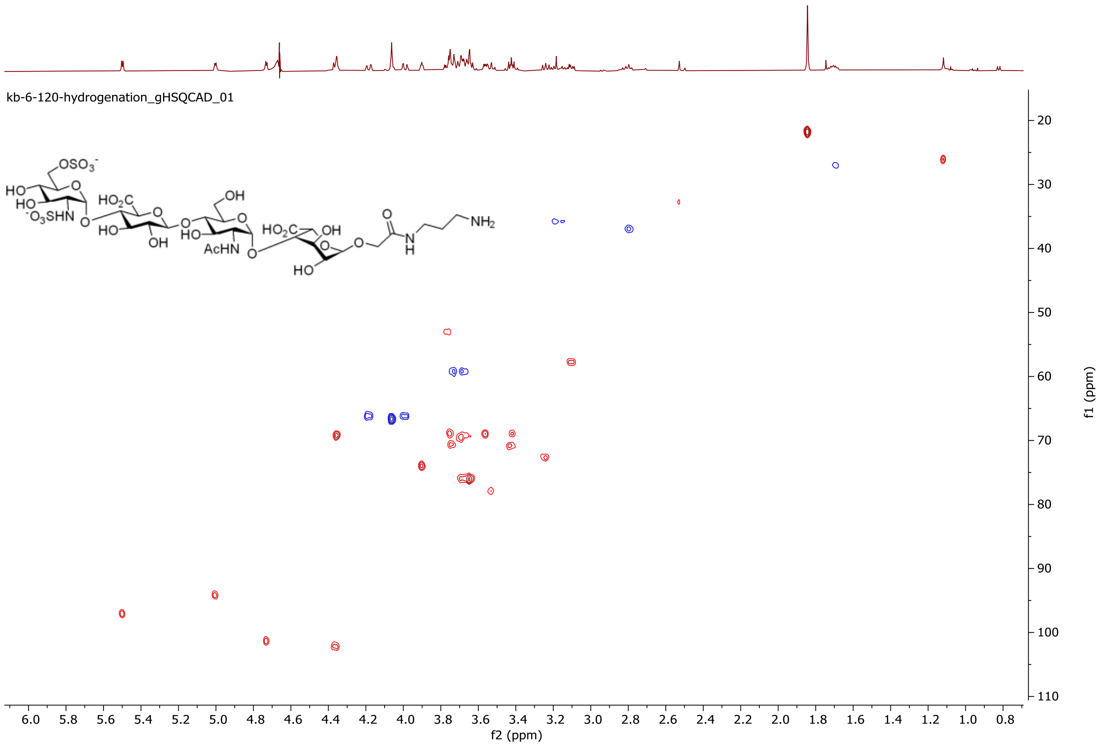

^{1}H-decoupled HSQC of compound **94**

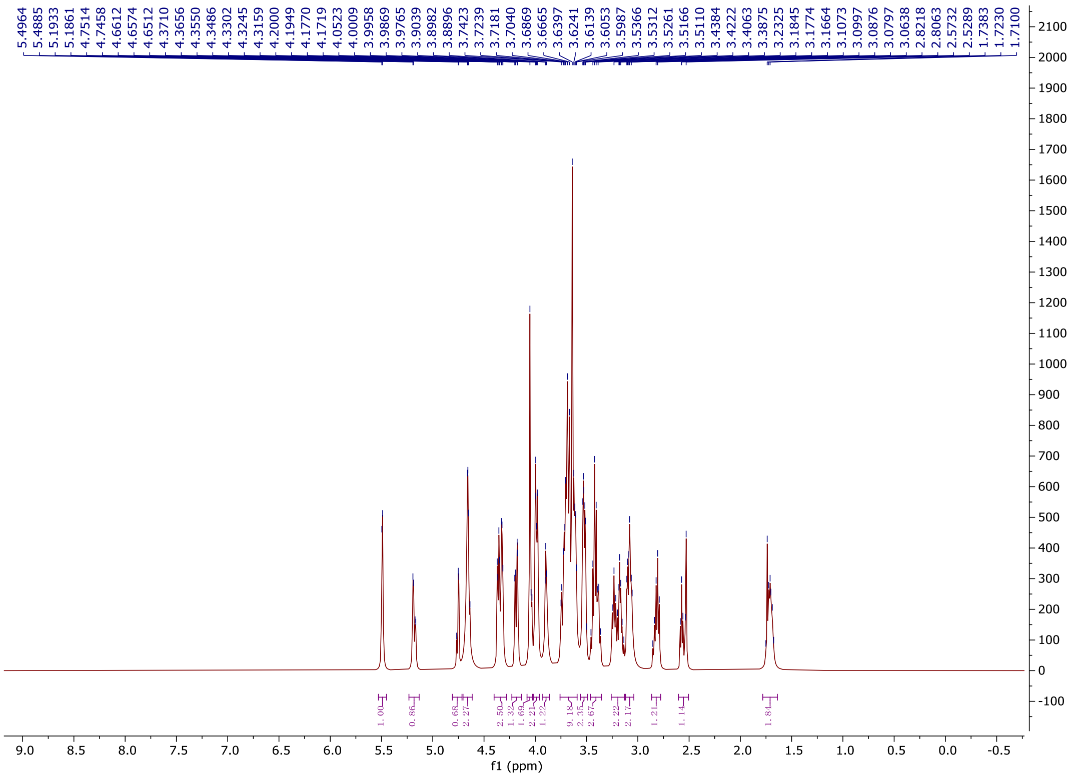

^{1}H NMR spectrum of compound **95**

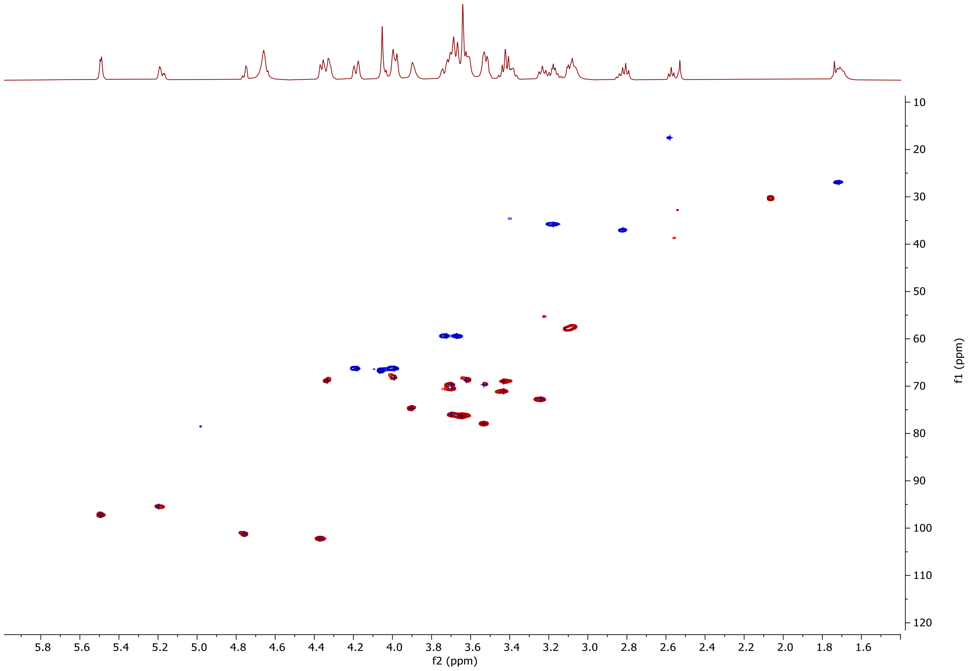

^{1}H-decoupled gHSQC NMR spectrum of compound **95**

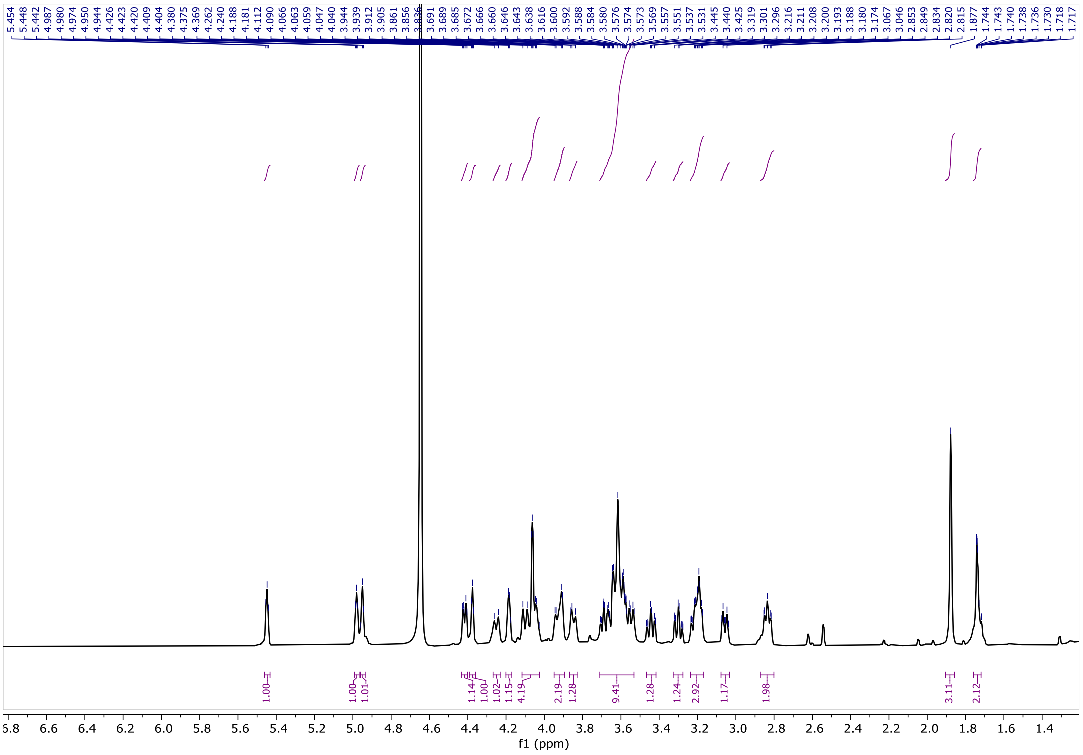

^{1}H NMR spectrum of compound **96**

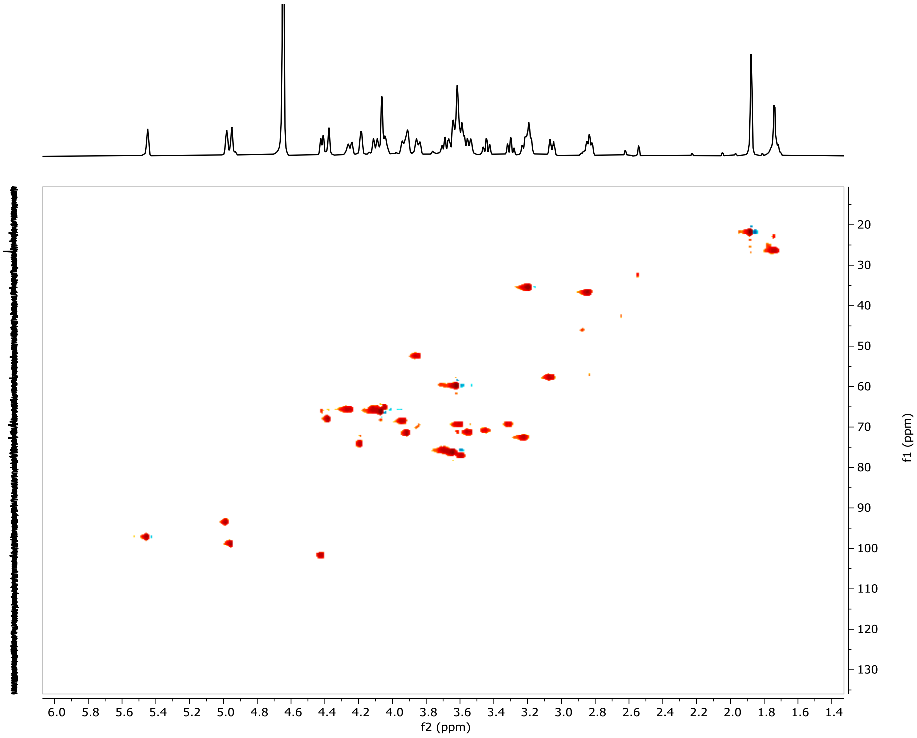

^{1}H-decoupled gHSQC NMR spectrum of compound **96**

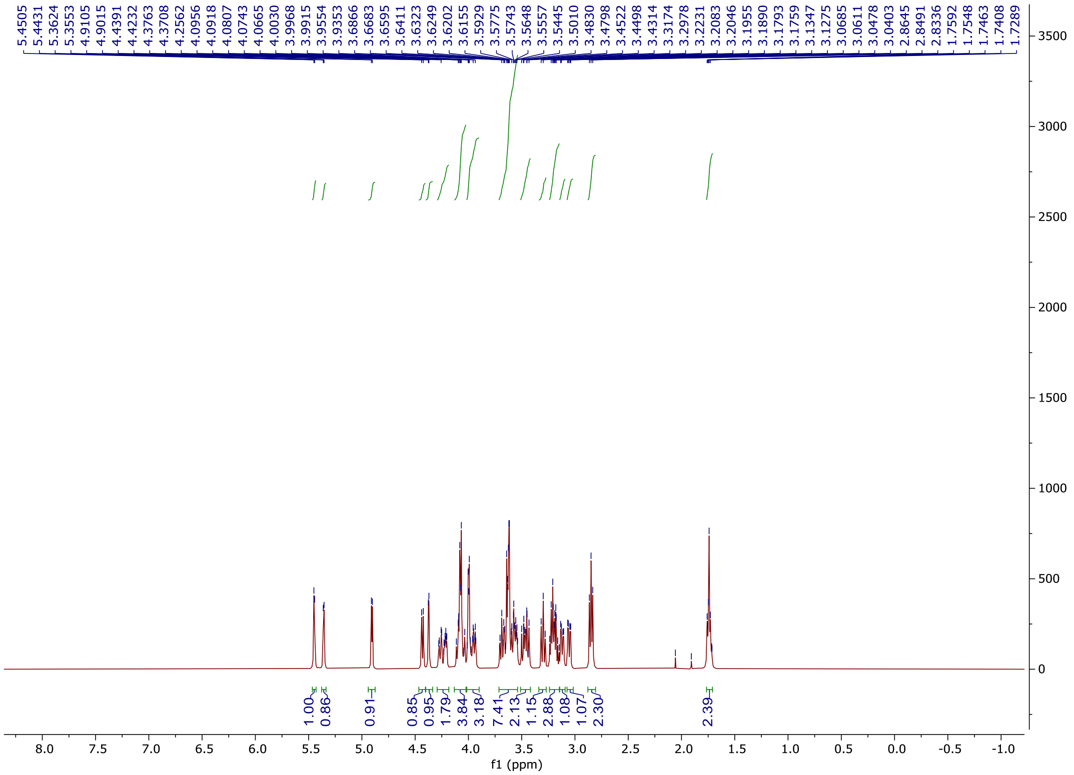

^{1}H NMR spectrum of compound **97**

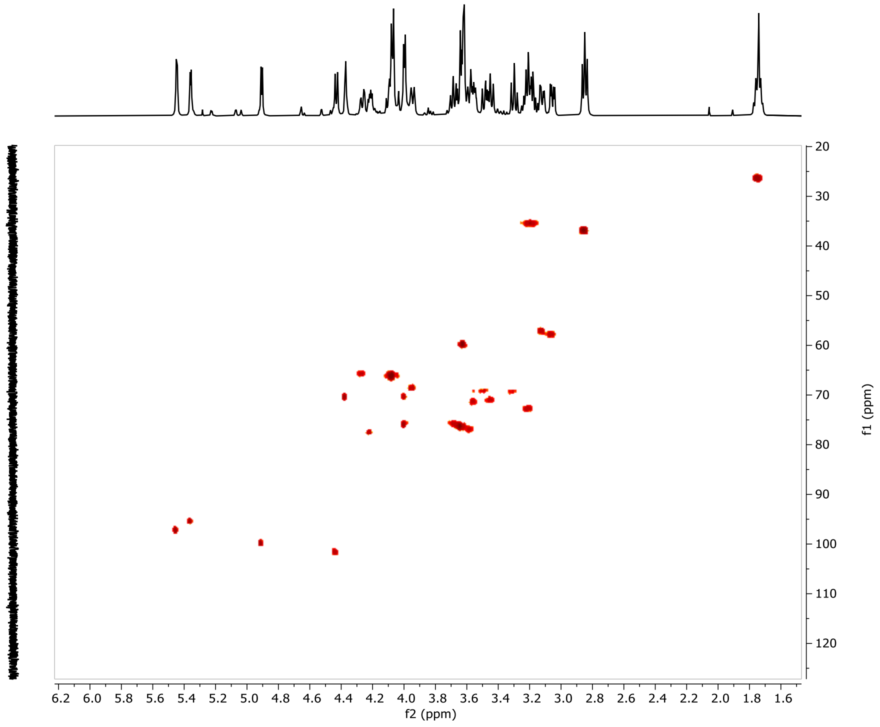

^{1}H-decoupled gHSQC NMR spectrum of compound **97**

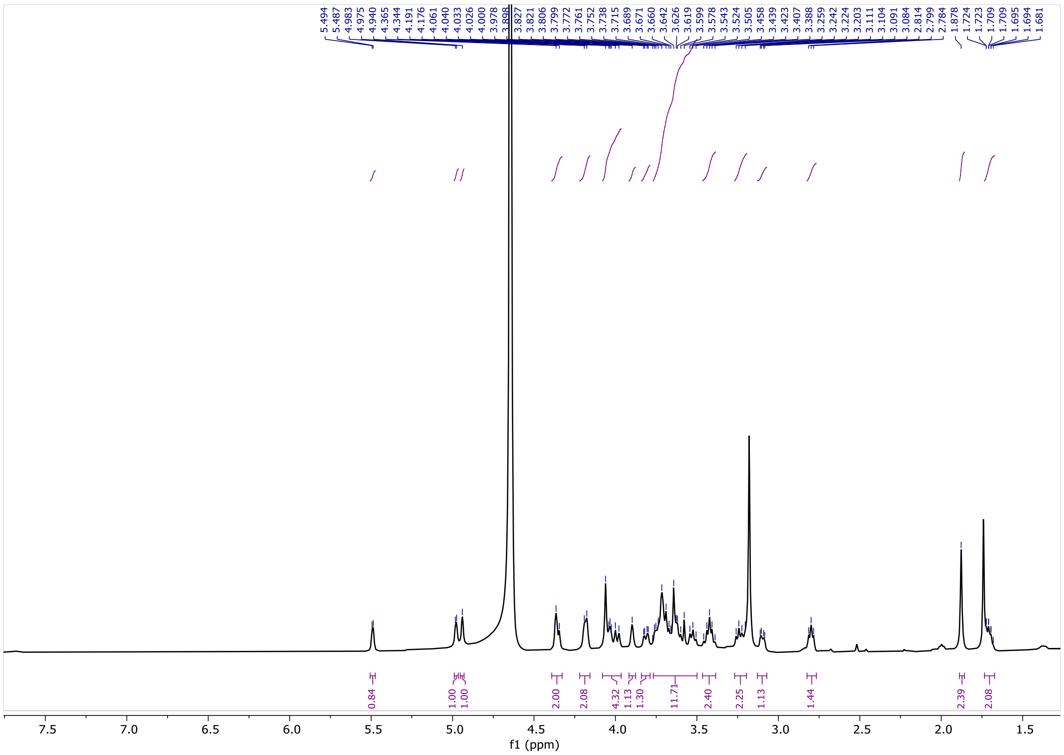

^{1}H NMR spectrum of compound **98**

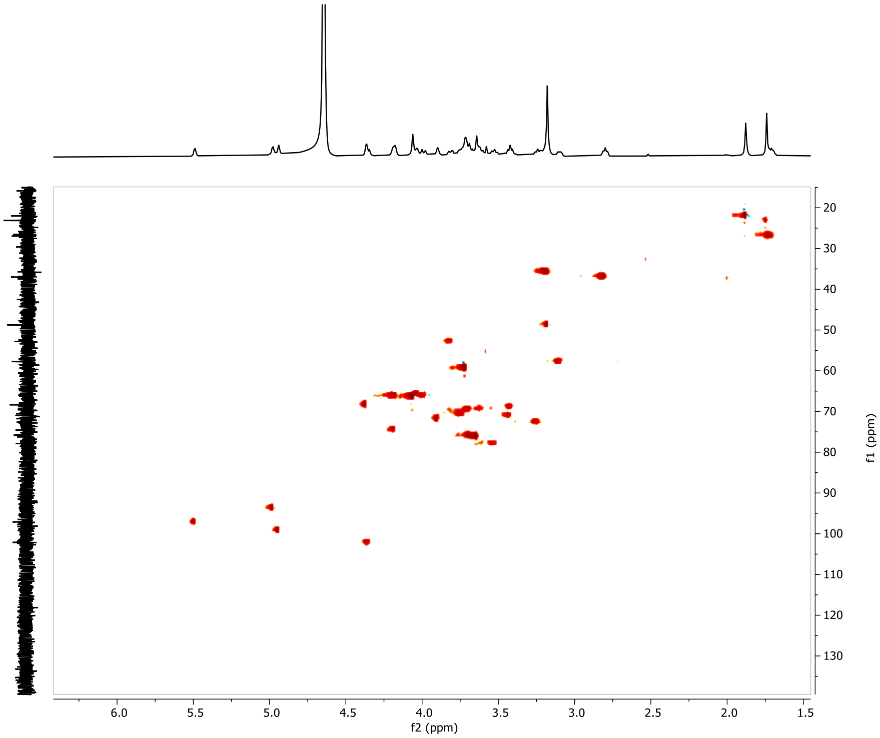

^{1}H-decoupled gHSQC NMR spectrum of compound **98**

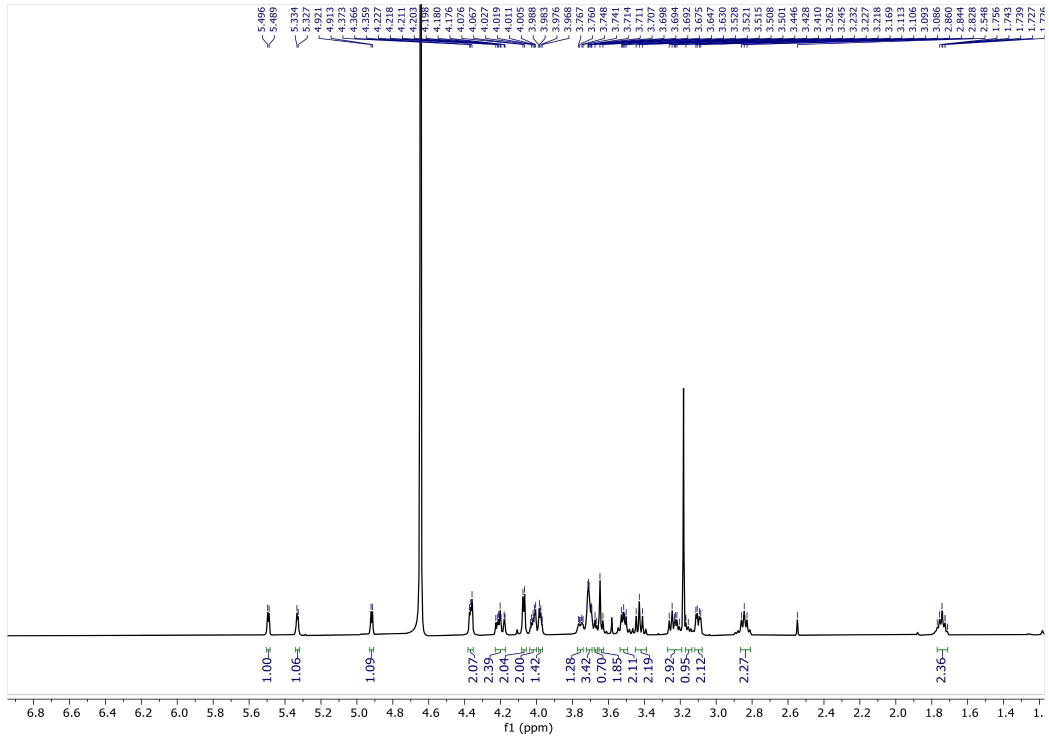

^{1}H NMR spectrum of compound **99**

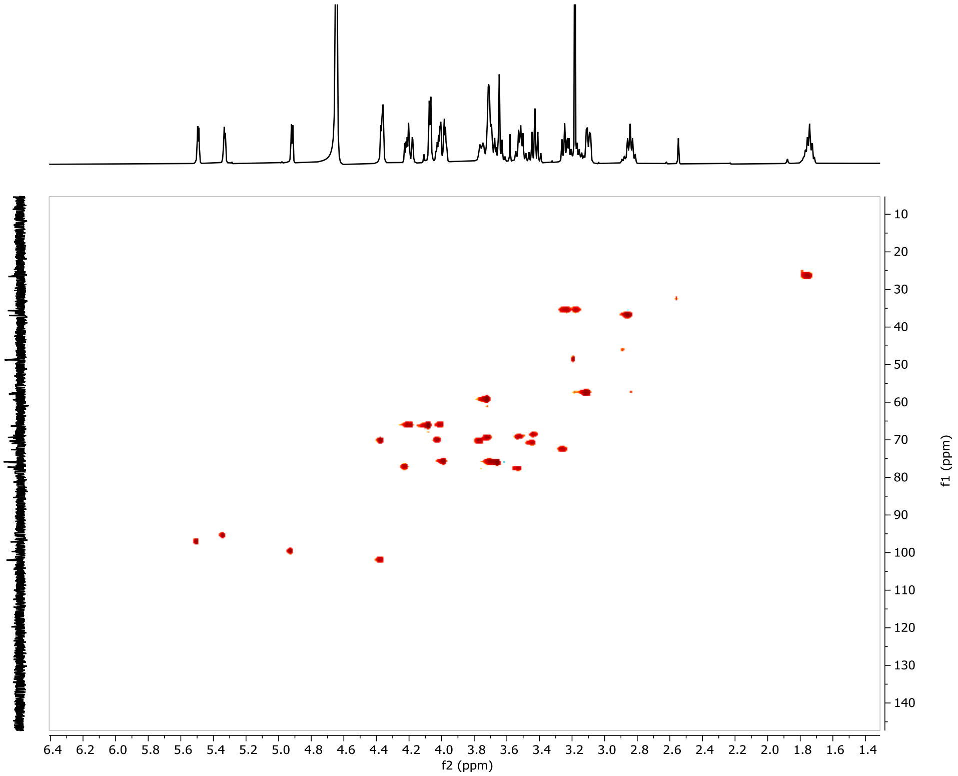

^{1}H-decoupled gHSQC NMR spectrum of compound **99**

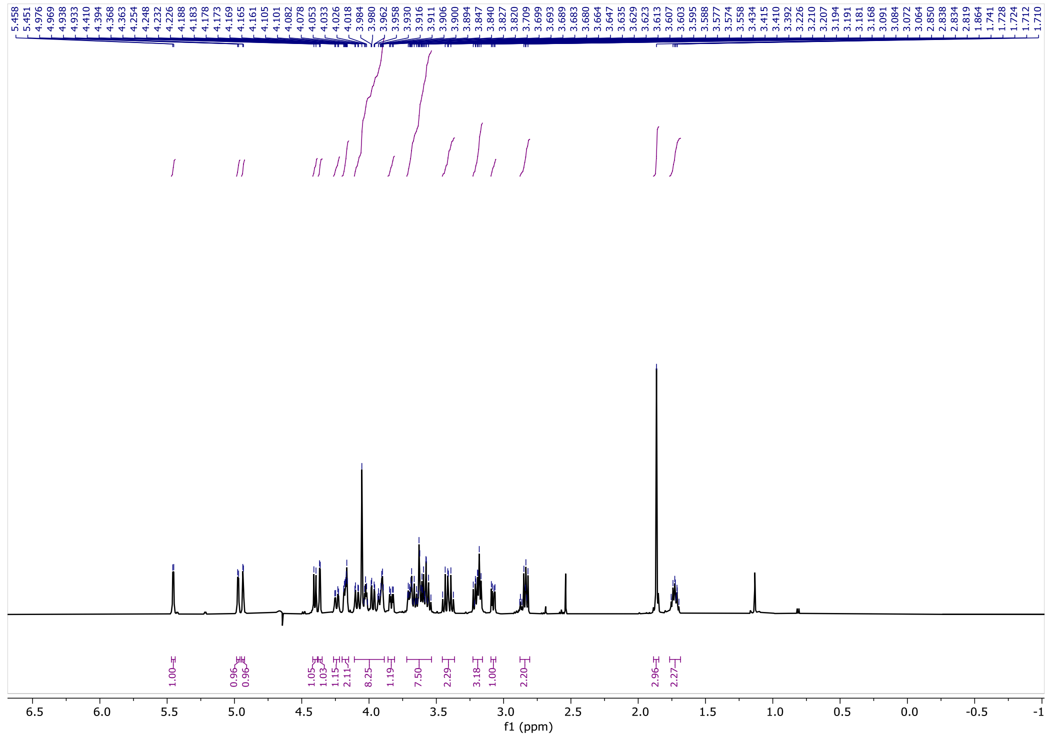

^{1}H NMR spectrum of compound **100**

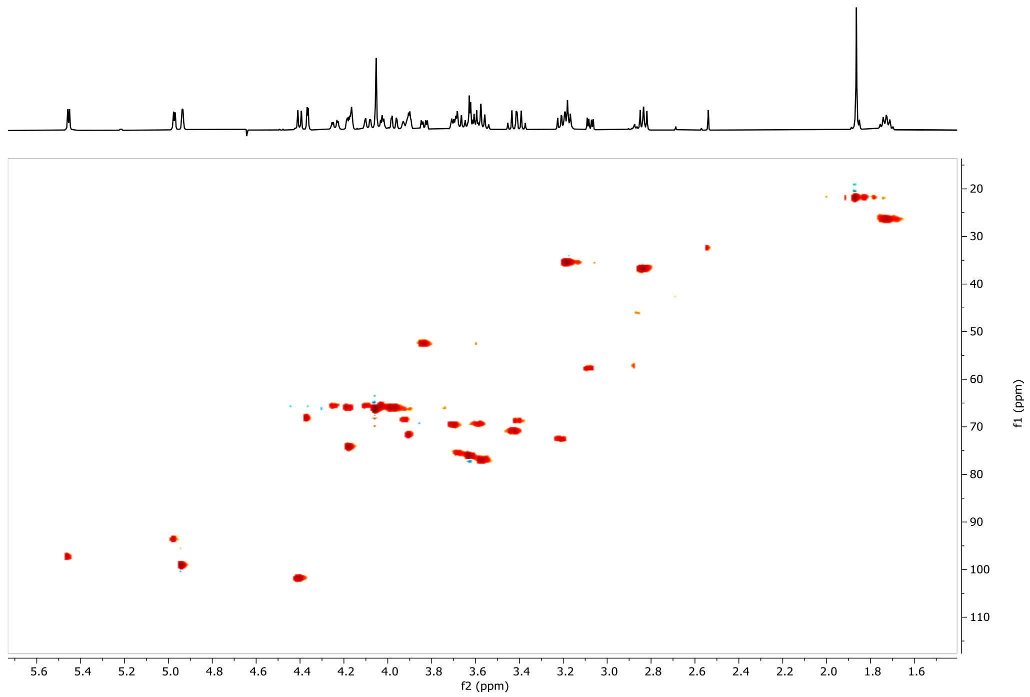

^{1}H-decoupled gHSQC NMR spectrum of compound **100**

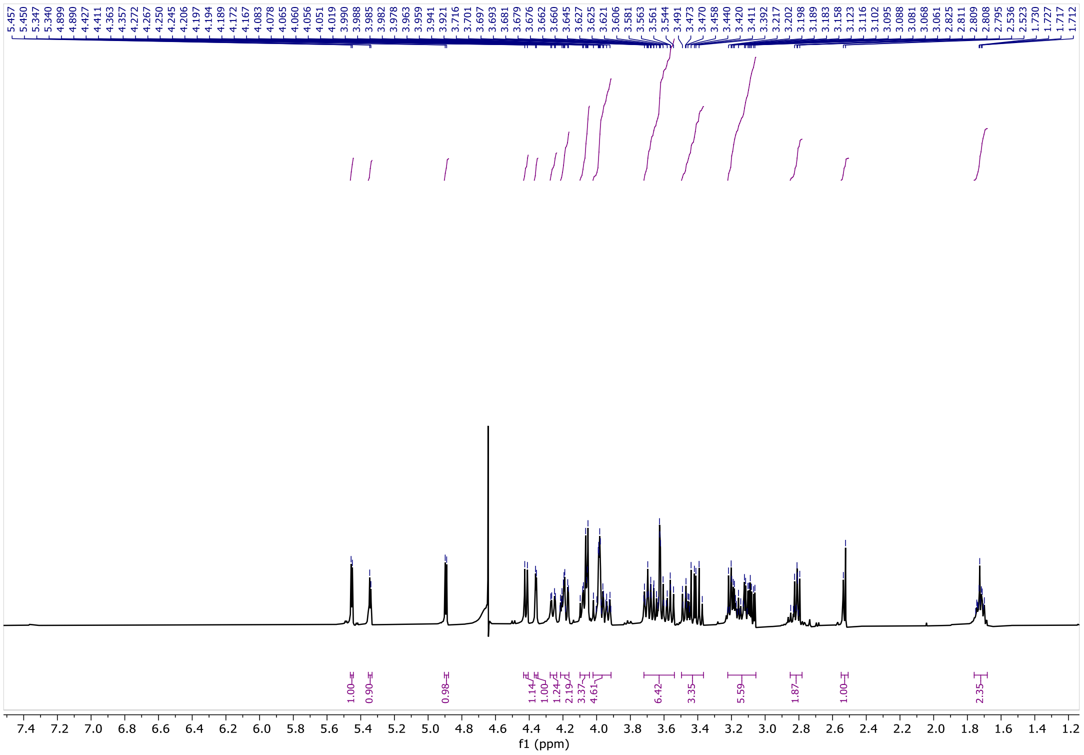

^{1}H NMR spectrum of compound **101**

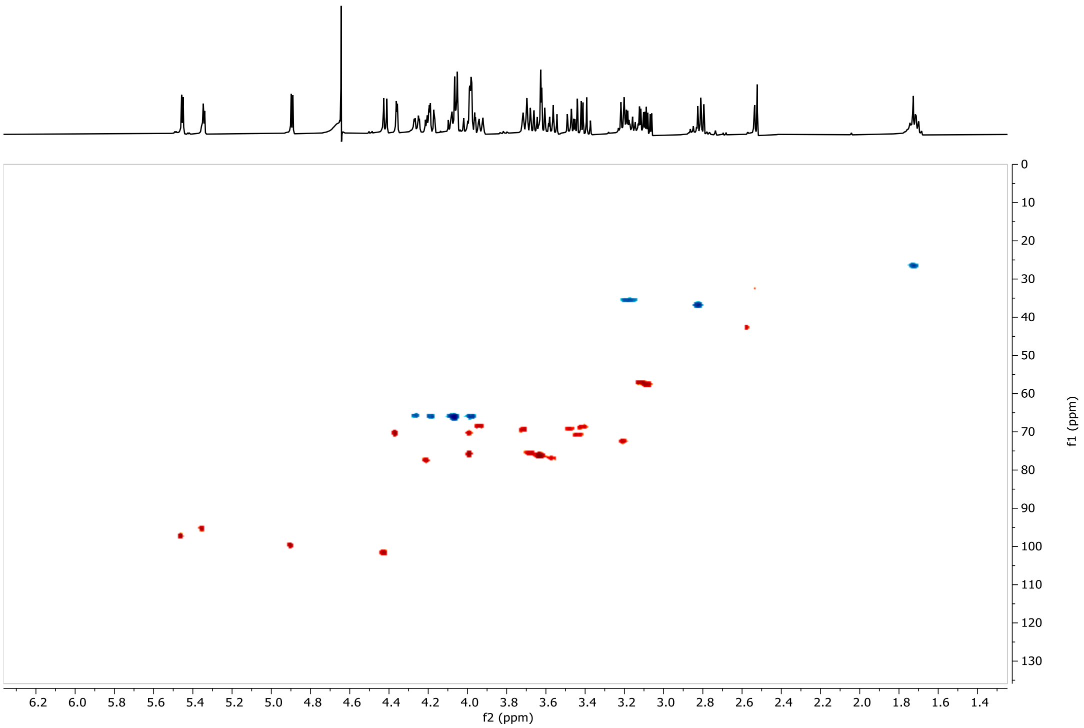

^{1}H-decoupled gHSQC NMR spectrum of compound **101**

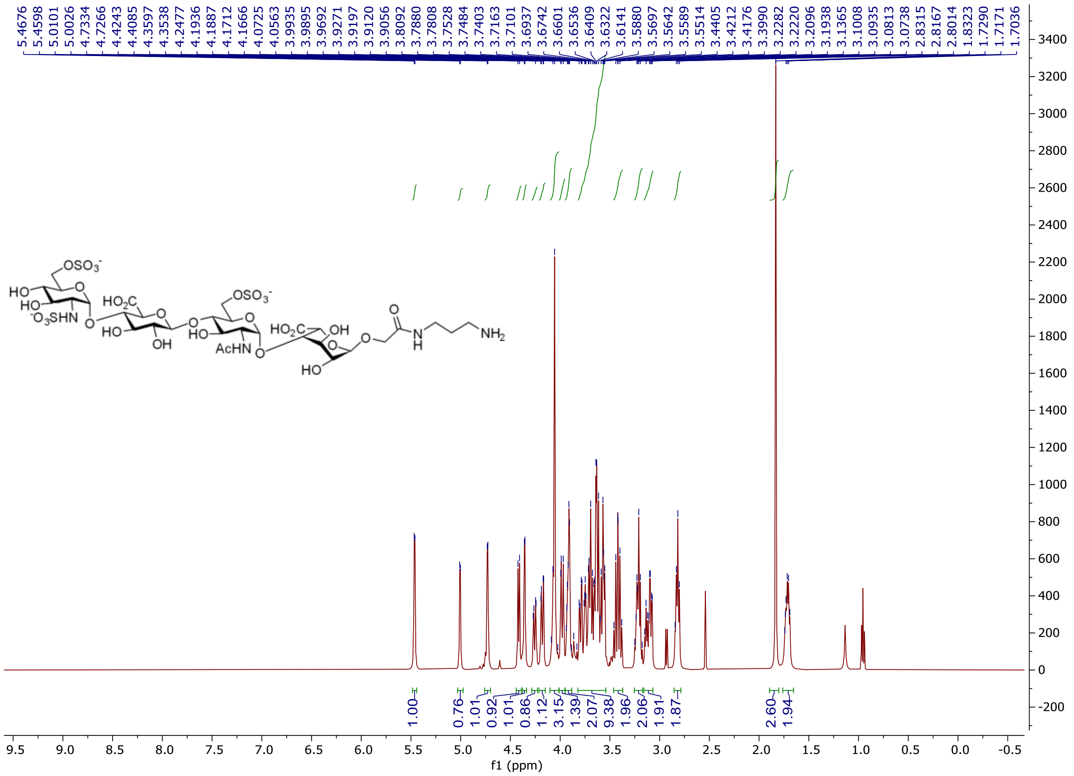

^{1}HNMR spectrum of compound **102**

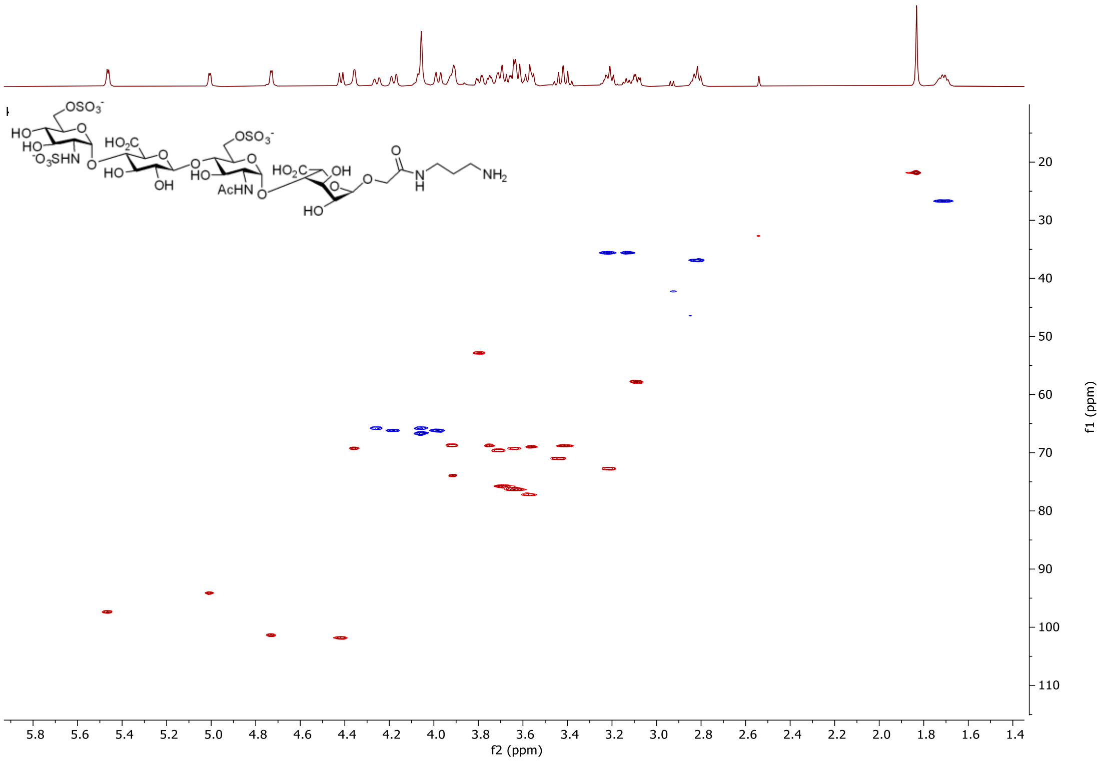

^{1}H-decoupled HSQC of compound **102**

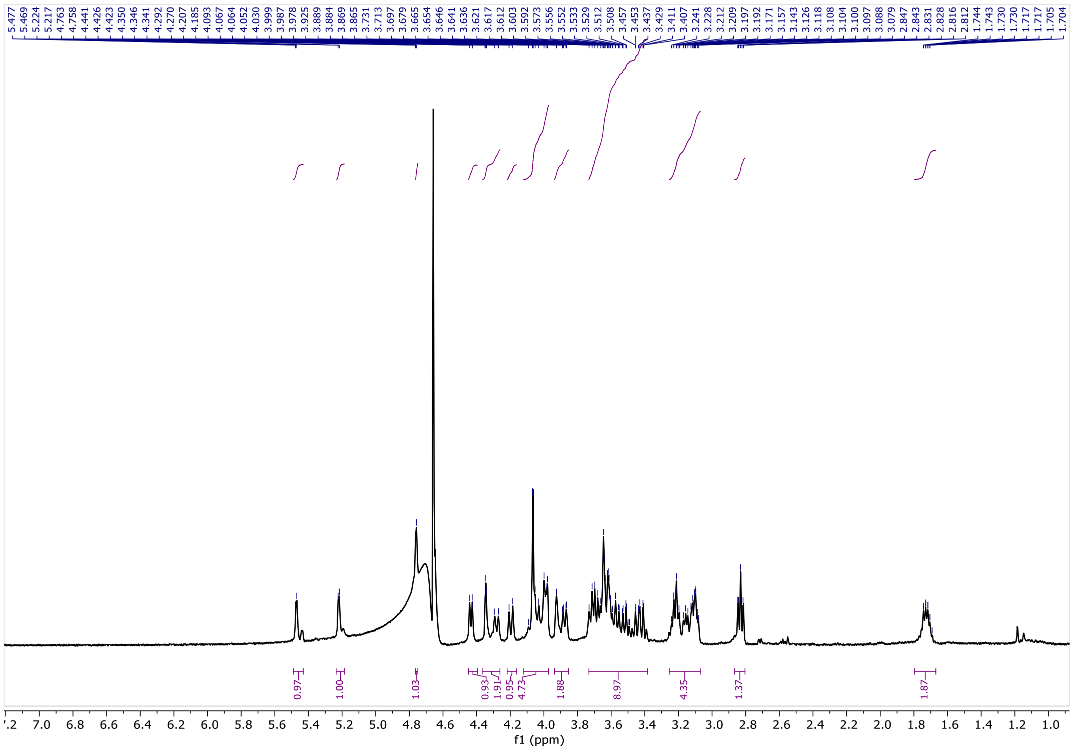

^{1}H NMR spectrum of compound **103**

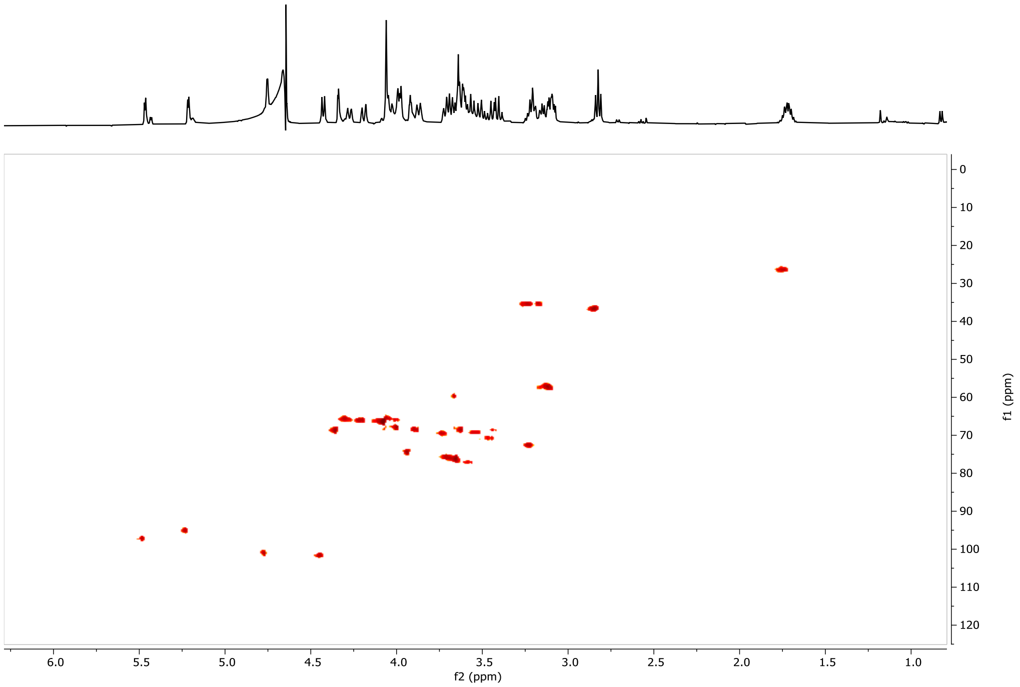

^{1}H-decoupled gHSQC NMR spectrum of compound **103**
